# Burden of respiratory tract cancers in China and its provinces, 1990–2021: a systematic analysis of the Global Burden of Disease Study 2021

**DOI:** 10.1016/j.lanwpc.2025.101485

**Published:** 2025-01-30

**Authors:** Xiaozhu Liu, Qizhi Yang, Liming Pan, Yanfang Ye, Lirong Kuang, Dandan Xu, Liuhua Wang, Shuang Hu, Yifeng Nie, Jian Huang, Jinxiu Qu, Chenan Liu, Wanyan Tang, Pengpeng Ye, Queran Lin, Ying Hu, Wenyi Jin

**Affiliations:** a Department of Orthopaedics, Renmin Hospital of Wuhan University, Wuhan University, Wuhan, 430060, China; b Emergency and Critical Care Medical Center, Beijing Shijitan Hospital, Capital Medical University, Beijing, 100038, China; c Northern Jiangsu People's Hospital Affiliated to Yangzhou University, Yangzhou, Jiangsu, 225009, China; d Department of Thoracic Surgery, No.6 People's Hospital of Xuzhou, Xuzhou, Jiangsu, 221006, China; e Xuzhou Tongshan District Huangji Town Health Center, Xuzhou, Jiangsu, 221145, China; f School of Cyber Science and Technology, University of Science and Technology of China, Hefei, 230026, China; g Clinical Research Design Division, Clinical Research Center, Sun Yat-Sen Memorial Hospital, Sun Yat-Sen University, Guangzhou, Guangdong, 510120, China; h Department of Ophthalmology, Wuhan Wuchang Hospital (Wuchang Hospital Affiliated to Wuhan University of Science and Technology), Wuhan, 430063, China; i Department of Emergency Intensive Care Unit, The Affiliated Hospital of Xuzhou Medical University, Xuzhou, Jiangsu, 221004, China; j The Yangzhou Clinical Medical College of Xuzhou Medical University, Xuzhou, Jiangsu, 221004, China; k Xiangya School of Nursing, Central South University, 410031, China; l National Center for Nanoscience and Technology, Beijing, 100190, China; m Department of Diagnostic Ultrasound, Sir Run Run Shaw Hospital, Zhejiang University College of Medicine, Hangzhou, 310030, China; n Departments of General Surgery, Beijing Shijitan Hospital, Capital Medical University, Beijing, 100038, China; o Department of Gastrointestinal Surgery, Beijing Shijitan Hospital, Capital Medical University, Beijing, 100038, China; p Department of Oncology, Chongqing Hospital of Traditional Chinese Medicine, Chongqing, 408499, China; q The George Institute for Global Health, University of New South Wales, Sydney, Australia; r National Centre for Non-Communicable Disease Control and Prevention, Chinese Centre for Disease Control and Prevention, Beijing, 100050, China; s Guangdong Provincial Key Laboratory of Malignant Tumor Epigenetics and Gene Regulation, Breast Tumor Center, Clinical Research Design Division, Clinical Research Center, Sun Yat-Sen Memorial Hospital, Sun Yat-Sen University, Guangzhou, China; t Department of Primary Care and Public Health, Imperial College London, London, UK; u Department of Medical Oncology, Beijing Chest Hospital, Capital Medical University, Beijing Tuberculosis and Thoracic Tumor Research Institute, Beijing, 101149, China; v Department of Biomedical Sciences, City University of Hong Kong, 999077, Hong Kong Special Administrative Region of China

**Keywords:** Respiratory tract cancers, Tracheal, bronchus, and lung cancer, Larynx cancer, Global Burden of Disease

## Abstract

**Background:**

Respiratory tract cancers emerged as a public health challenge with the highest incidence and mortality among all cancer types in China, despite many national policies in place, such as early cancer screening. It is of outmost importance to monitor the burden of respiratory tract cancers across China and its provinces for refining health strategies.

**Methods:**

Based on Global Burden of Disease (GBD) estimates, the present study investigated the age-sex specific pattern alterations of incidence, prevalence, mortality, and disability adjusted life years (DALYs) of respiratory tract cancers in China from 1990 to 2021, as well as its Estimated Annual Percentage Change (EAPC), Age-Standardized Incidence Rate (ASIR), and Age-Standardized Mortality Rate (ASMR).

**Findings:**

Between 1990 and 2021, China experienced an escalation in burdens of respiratory tract cancers, with the new cases surging from 274,752 (95% Uncertainty Interval (UI): 234,741–315,112) to 934,704 (750,040–1,136,938), marking an increase of 240.20% (156.05–342.29). Their attributed deaths similarly increased from 278,235 (238,518–322,013) to 814,121 (652,231–994,858). In 2021, the eastern and northeastern regions reported the highest incidence and mortality rates, particularly Shandong, with the highest new cases at 77,225 (58,842–101,352), while Tibet, Qinghai, and Macau observed the lowest. Regarding laryngeal cancer, Guangdong reported the highest incidence at 3466 (2230–4934), with Hainan exhibiting the highest ASIR at 3.46 (2.10–5.11) per 100,000 person-years and ASMR at 2.11 (1.37–3.09) per 100,000 person-years. Over the same timeframe, the EAPC for the ASIR of tracheal, bronchus, and lung cancer was 0.88 (0.63–1.14), and for ASMR, it was 0.29 (0.05–0.62), signifying an upward trend. Conversely, laryngeal cancer exhibited a stable ASIR with an EAPC of 0.04 (−0.22 to 0.30) and a declining ASMR with an EAPC of −1.69 (−1.80 to 1.59). Tracheal, bronchus, and lung cancer burdens exhibited notable sex differences, with their ASIR being 62.63 (46.50–79.90) per 100,000 person-years in males and 28.16 (22.22–34.90) per 100,000 person-years in females. For laryngeal cancer, the ASIR was 3.12 (2.34–4.04) per 100,000 person-years for males and 0.58 (0.35–0.79) per 100,000 person-years for females. Smoking and air pollution emerged as the predominant risk factors contributed to tracheal, bronchus, and lung cancer, accounting for 61.58% (30.00–82.95) and 25.98% (16.94–35.00) of deaths, respectively. In contrast, smoking contributed more to laryngeal cancer-caused deaths (76.70% [65.55–85.15]), followed by alcohol use (14.52% [7.70–20.99]).

**Interpretation:**

The burden of respiratory tract cancers in China has increased over the past three decades, and without intervention, the associated health losses could escalate further. This burden predominantly affected the eastern provinces, particularly impacting older males. Our findings advocate for the formulation of targeted prevention, screening, and intervention strategies based on regional and sex disparities.

**Funding:**

10.13039/100000865Bill & Melinda Gates Foundation.

**Research in context**

**Evidence before this study**

Respiratory tract cancers emerged as a public health concern in China. However, the burden of diseases, injuries, risk factors, and the spatiotemporal trajectory of respiratory tract cancers in China and its provinces remains to be elucidated. Understanding the subnational patterns of respiratory tract cancers burden and exploring potential interventions would support policymakers to address this escalating health challenge.

**Added value of this study**

Using data from the GBD China database, this study systematically analyzes the age-sex-specific spatiotemporal patterns of incidence, prevalence, mortality, and DALYs burden attributed to respiratory tract cancer from 1990 to 2021. The incidence, deaths and DALYs of respiratory tract cancers witnessed a notable increase during study period, mainly driven by tracheal, bronchial, and lung cancers. These burdens featured high heterogeneity depending on geographies, age, and sex. The eastern and northeastern regions of China showed the highest incidence and mortality burdens compared to other regions, with males and older population suffering the heavier burdens. Smoking and air pollution were identified as the dominant risk factors contributing to these burdens.

**Implications of all the available evidence**

The current investigation revealed increasing patterns of respiratory tract cancer burdens in China from 1990 to 2021, with tracheal, bronchus, and lung cancer featuring the highest incidences and mortality rates. These burdens varied across geographies, ages, and sexes. With these results, policymakers and stakeholders could be able to optimize health strategies and tailor new interventions for different populations, such as controlling smoking to reverse the increasing burdens of respiratory tract cancers in China and its 34 provinces, to alleviate health, social, and economic losses attributed to respiratory tract cancers.

## Introduction

Respiratory tract cancers in China, including Tracheal, bronchus, and lung cancer, and Larynx cancer, remain a notable public health concern, posing serious challenges.^1^, ^2^, ^3^ According to the latest national cancer statistics report, in 2022, the incidence of lung cancer in China reached 1.1 million cases, with 0.7 million deaths. Lung cancer ranks first in both standardized incidence and mortality rates in China and globally.^4^^,^^5^ Although Larynx cancer has a relatively lower incidence within respiratory tract cancers, its mortality rate is higher compared with Tracheal, bronchus, and lung cancer.^6^ As of now, Tracheal, bronchus, and lung cancer are among the most common cancers globally and are a leading cause of cancer-related deaths.^7^ China, being a populous country representing approximately 18% of the global population, has notable implications for both its own efforts and global initiatives to reduce the burden of respiratory tract cancers. China has already formulated a series of policies to alleviate the burden of respiratory tract cancers. Since 2005, China has funded four cancer screening programs and implemented a cancer registration system. Efforts are underway within the framework of cancer control in China to ensure the sustainable development of population-based cancer registries (PBCRs) to more effectively assess national cancer control policies.^8^ Preventing, controlling, and treating respiratory tract cancers are key steps in achieving this sustainable development goal, aligning with China's efforts to reduce the burden of respiratory tract cancers. The aforementioned data and policies highlight the urgency for China to dynamically monitor the burden of respiratory tract cancers. Despite China's implementation of measures to improve disease surveillance and reporting systems, the COVID-19 pandemic has posed unprecedented challenges to epidemiological monitoring between 2019 and 2021, resulting in significant data gaps for respiratory tract cancers in China. Given these research gaps, this study aims to symmetrically analyze the incidence, mortality, and risk factors of respiratory tract cancers in China and its provinces from 1990 to 2021, considering the latest public health emergency.

## Methods

### Overview

Using the 2021 Global Burden of Disease (GBD) assessment method, we analyzed the trends in incidence, prevalence, mortality rate, and Disability-Adjusted Life Years (DALYs) rates of respiratory tract cancers in 34 geographic units of China. The analysis was conducted at the provincial level, including 22 provinces, 5 autonomous regions, and 4 municipalities directly under the central government. Hong Kong, Macau, and Taiwan were also included in the analysis, totaling 34 provinces. We will refer to all these 34 geographic units as “province”. To ensure transparency and replicability, our study adhered to the “Guidelines for Accurate and Transparent Health Estimates Reporting (GATHER)”. The data used in this study are aggregated China GBD data and do not contain any personally identifiable information. Therefore, ethical approval or consent was not required for participation in the study. This comprehensive analysis aims to provide insights into the burden of respiratory tract cancers across different provinces in China, identifying key trends and risk factors. The findings from this study can serve as valuable evidence to guide policy formulation and public health interventions aimed at reducing the burden of respiratory tract cancers in China. By understanding the geographic variations and risk factors associated with respiratory tract cancers, targeted strategies can be developed to address the specific needs of different regions, ultimately contributing to improving public health outcomes and reducing the overall burden of cancer in China. Ethical approval was not required as the study uses data available in the public domain.

### Study population

The cohort of this study encompass all patients diagnosed with respiratory tract cancers in China from 1990 to 2021. The data is sourced from the Chinese Cancer Registry and includes hospital records, government reports, survey data, and other public health information. These data have undergone rigorous quality control and standardization processes to ensure their accuracy and comparability. Respiratory tract cancers, as defined in this study, include Tracheal, Bronchus, and Lung Cancer, as well as Larynx Cancer. Diagnoses adhere to the ICD-10 coding system, where Tracheal, Bronchus, and Lung Cancer correspond to ICD-10 codes C33-C34.9, D02.1-D02.3, D14.2-D14.3, D38.1, and Larynx Cancer corresponds to ICD-10 codes C32-C32.9, D02.0, D14.1, D38.0. The ICD-10 codes C33-C34.9 include non-small cell lung cancer (NSCLC) and small cell lung cancer (SCLC), covering all types of bronchial and lung malignancies as well as lung cancer histological sub-classes. The codes D02.1-D02.3 refer to in-situ cancers of the bronchus, lung, and trachea. The codes D14.2-D14.3 and D38.1 pertain to other tumors of the trachea, bronchus, and lung, with D14.2-D14.3 denoting benign tumors and D38.1 indicating other non-specific benign tumors. The data collection indicators correspond to the GBD database, with causes identified as “Tracheal, Bronchus, and Lung Cancer” and “Larynx Cancer”. Measurements include “incidence”, “prevalence”, “mortality” rate, and “DALYs”. Locations cover China and its 34 provinces. Metrics include “number”, “rate”, and “percent”. Risk factors include “smoking”, “air pollution”, “secondhand smoke”, “other environmental risks”, “high alcohol use”, “occupational exposure to sulfuric acid”, and “occupational exposure to asbestos”. Sex is categorized as “male”, “female”, and “both”, while age ranges from all ages to age-standardized, 0 to 95+ years, and corresponding 5-year bands. In the GBD database, data is first collected from a variety of sources. Then, the relationship between each risk factor and health outcome is analyzed to estimate their relative risks. Following this, researchers calculate the summary exposure values and theoretical minimum risk exposure levels for each risk factor. The population attributable fraction (PAF) is then used to predict the proportion of health risk that could be reduced if exposure to the risk factor is lowered to the minimum level. Finally, by multiplying the PAF with the disease burden associated with specific health outcomes (measured in DALYs), an estimate of the health loss attributable to these risk factors is derived.

### Statistical analysis

Using data from the GBD database specific to China, this study describes the incidence, prevalence, mortality rates, and DALY rates for respiratory tract cancers in China from 1990 to 2021. The Estimated Annual Percentage Change (EAPC) uses the year as the independent variable. After applying a logarithmic transformation to the incidence rates, the geometric mean for each year is taken, treating this sequence of geometric means as the dependent variable to fit a linear regression. It was calculated using the formula 100 × (exp (β) −1), with the 95% confidence interval (CI) derived from the linear regression model, assessing the EAPC for respiratory tract cancers across China and its 34 provinces. Visualizations include a map of China showing the Age-Standardized Incidence Rate (ASIR) and Age-Standardized Mortality Rate (ASMR) for respiratory tract cancers in 2021. Additionally, maps visualizing the percentage change (percentage change = ((New Value−Old Value)/Old Value) × 100%) in age-standardized incidence and mortality rates from 1990 to 2021, and horizontal bar charts comparing the ASIR and ASMR rates by sex across the provinces in 2021, were created. Dual-axis charts illustrate the differences in the number of cases and deaths, incidence and mortality rates across different age groups and sexes for 2021. Stacked area charts and percentage stacked area charts display the trends in mortality numbers by age group over time, cumulative effects, and their proportional contributions. Charts depicting the percentage contribution of various risk factors to respiratory tract cancer deaths across China and its provinces were also produced. All statistics were performed using the R program (Version 4.2.1).

### Role of the funding source

The funder of the study had no role in the study design, data collection, data analysis, data interpretation, or writing of the report.

## Results

### National alterations in burdens of respiratory tract cancers in China from 1990 to 2021

**Fig. 1:**
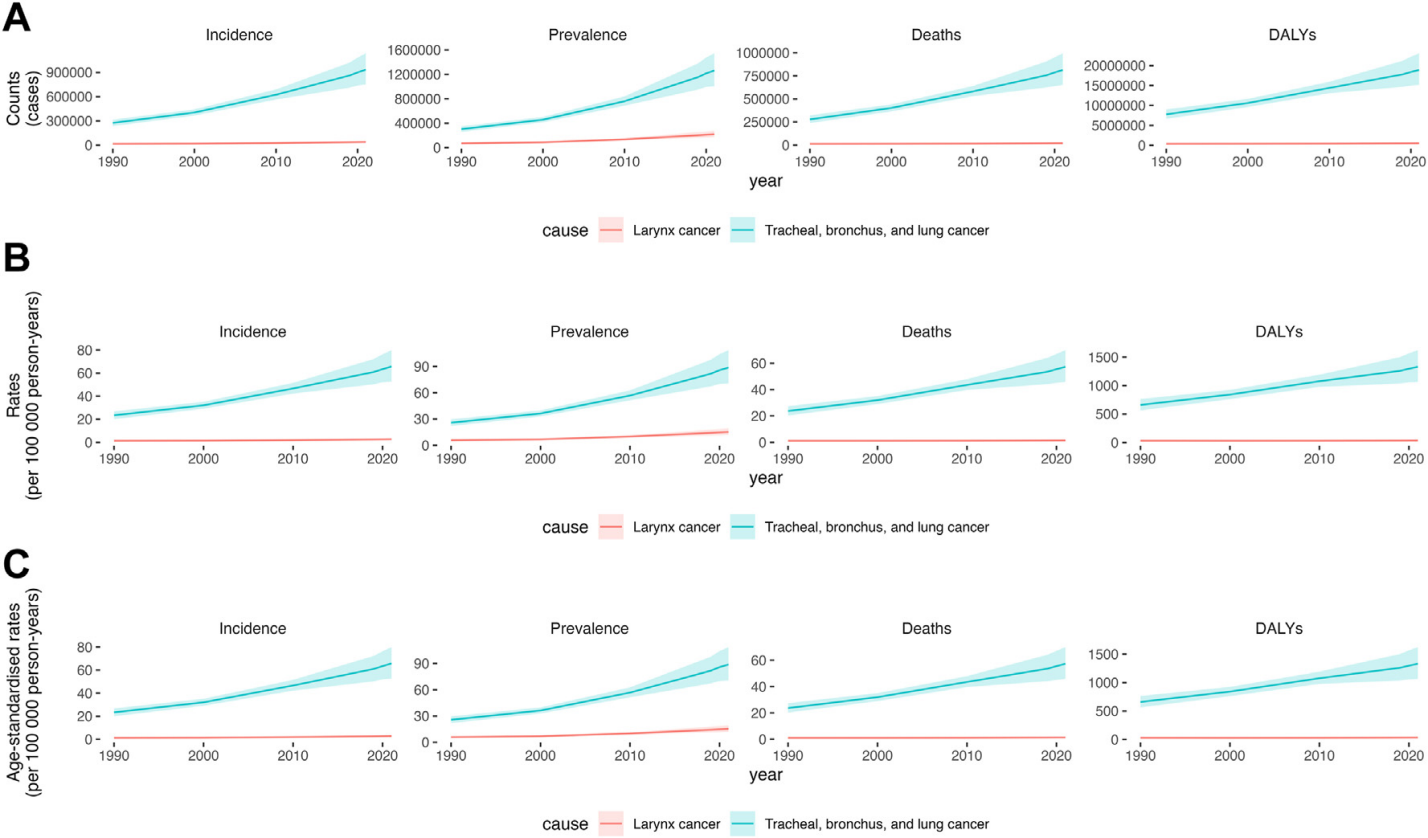
General trend of respiratory tract cancers in China, 1990–2021. (A) All-age counts. (B) All-age rates (per 100,000 person-years). (C) Age-standardized rates (per 100,000 person-years); DALYs = disability-adjusted life-years.

From 1990 to 2021, the burdens of respiratory tract cancers in China have continued to worsen, with the escalated number of new cases, prevalence, mortality, and DALYs (Fig. 1A). The new cases of Tracheal, bronchus, and lung cancer manifested a pronounced growth, rising from 274,752 cases in 1990 (95% UI: 234,740.75–315,111.78) to 934,704 cases in 2021 (95% UI: 750,040.14–1,136,937.93). The incidence of larynx cancer was raised from 15,434 cases in 1990 (95% UI: 12,624.19–18,174.02) to 38,905 cases in 2021 (95% UI: 30,369.67–49,486.18). From 1990 to 2021, both the all-age and age-standardized incidence, prevalence, mortality, and DALY rates for respiratory tract cancers in China have shown an escalating trend (Fig. 1A and B). The ASIR of tracheal, bronchus, and lung cancer increased from 33.11 (95% UI: 28.47–37.79) per 100,000 person-years in 1990 to 44.01 (95% UI: 35.45–53.35) per 100,000 person-years in 2021. Similarly, the ASMR exhibited an upward trend, rising from 34.74 (95% UI: 30.07–39.98) per 100,000 person-years in 1990 to 38.97 (95% UI: 31.39–47.34) per 100,000 person-years in 2021. In contrast, the ASIR for larynx cancer showed a slight decrease, declining from 1.82 (1.50–2.13) per 100,000 person-years in 1990 to 1.79 (95% UI: 1.40–2.26) per 100,000 person-years in 2021. Its ASMR displayed a downward trend, decreasing from 1.59 (95% UI: 1.31–1.86) per 100,000 person-years in 1990 to 0.94 (95% UI: 0.73–1.19) per 100,000 person-years in 2021 (Supplementary Material pp 2–23) (Fig. 1).

### Provincial burden distribution of respiratory tract cancers in China from 1990 to 2021

In 2021, the provinces of Shandong, Sichuan, and Jiangsu had the highest incidence of tracheal, bronchus, and lung cancer in China. Conversely, Qinghai, Tibet, and Macao had the lowest incidence of tracheal, bronchus, and lung cancer (Supplementary Material pp 24–27). For larynx cancer, the highest incidence was observed in Guangdong, Sichuan, and Liaoning provinces. Conversely, Qinghai, Macao, and Tibet reported the lowest incidence (Supplementary Material pp 28 and 29). In terms of mortality due to tracheal, bronchus, and lung cancer in 2021, the highest numbers were recorded in Shandong, Sichuan, and Jiangsu. Conversely, the lowest mortality rates were observed in Qinghai, Tibet, and Macao (Supplementary Material pp 30–33). Regarding mortality due to larynx cancer, the highest numbers were recorded in Hunan, Sichuan, and Guangdong. Conversely, the lowest mortality rates were observed in Qinghai, Tibet, and Macao (Supplementary Material pp 34–37) (Table 1).

**Table 1:** Province trend of respiratory tract cancers in all genders in China, 1990–2021.

Cause	Location	Incidence (95% UI)					Mortality (95% UI)				
		Case_1990	ASIR	Case_2021	ASIR	EAPC of ASIR	Case_1990	ASMR_1990	Case_2021	ASMR_2021	EAPC of ASMR
			(per 100,000), 1990		(per 100,000), 2021	(95% CI), 1990–2021		(per 100,000), 2021		(per 100,000), 2021	(95% CI), 1990–2021
Larynx cancer	China	15,434.2 (12,624.2–18,174)	1.8 (1.5–2.1)	38,904.9 (30,369.7–49,486.2)	1.8 (1.4–2.3)	0.04 (−0.22 to 0.3)	12,869 (10,580.7–15,234.5)	1.6 (1.3–1.9)	19,814.1 (15,420.2–25,279.4)	0.9 (0.7–1.2)	−1.69 (−1.8 to −1.59)
Tracheal, bronchus, and lung cancer	China	274,752 (234,740.7–315,111.8)	33.1 (28.5–37.8)	934,704.1 (750,040.1–1,136,937.9)	44 (35.4–53.3)	0.88 (0.63–1.14)	278,235.2 (238,517.8–322,012.8)	34.7 (30.1–40)	814,120.6 (652,231.4–994,858.2)	39 (31.4–47.3)	0.29 (−0.05 to 0.62)
Larynx cancer	Anhui	695.7 (518.2–965.8)	1.7 (1.3–2.3)	1592.6 (1127.2–2300.3)	1.6 (1.2–2.4)	0 (−0.45 to 0.45)	572.1 (424–763.2)	1.5 (1.1–1.9)	741.6 (527.6–1038.5)	0.8 (0.6–1.1)	−2.08 (−2.42 to −1.75)
Tracheal, bronchus, and lung cancer	Anhui	14,409.4 (11,179.2–18,214.1)	36 (28.2–44.9)	39,173.9 (29,010.2–51,540.5)	40.8 (30.4–53.8)	0.39 (0.32–0.46)	14,748.8 (11,523.2–18,520.3)	38.4 (30.3–47.7)	36,571.4 (27,488.4–48,315)	38.2 (28.8–50.4)	−0.07 (−0.2 to 0.05)
Larynx cancer	Beijing	147.1 (111–195.5)	1.6 (1.2–2.1)	476.5 (317–706.1)	1.4 (1–2.1)	−0.39 (−0.51 to −0.28)	111.2 (82.2–146.5)	1.3 (1–1.7)	201 (136–295.8)	0.6 (0.4–0.9)	−2.46 (−2.76 to −2.17)
Tracheal, bronchus, and lung cancer	Beijing	3799.7 (2938.1–4736.2)	42.9 (33.6–53)	14,671.6 (11,122.1–18,894.3)	45.6 (34.7–58.6)	0.04 (−0.36 to 0.43)	3701.4 (2902.7–4692.8)	43.6 (34.5–54.1)	12,110.2 (9182.7–15,538.1)	38.5 (29.2–49.2)	−0.62 (−1.1 to −0.13)
Larynx cancer	Chongqing	210.6 (154.1–280.3)	1.7 (1.3–2.2)	979.8 (642.9–1441.6)	1.7 (1.1–2.6)	0.22 (−0.21 to 0.65)	174.3 (128.8–229.9)	1.5 (1.1–1.9)	448.3 (302.5–635.3)	0.8 (0.5–1.1)	−1.91 (−2.15 to −1.66)
Tracheal, bronchus, and lung cancer	Chongqing	6284.3 (4809.9–8016.8)	51.2 (39.1–65.3)	33,549.4 (24,911.2–45,395.2)	60 (44.7–80.8)	0.55 (0.45–0.65)	6424.7 (4937.1–8154.4)	54.1 (41.9–67.7)	30,585.9 (22,362.3–41,003.1)	55.2 (40.3–74)	0.06 (0–0.12)
Larynx cancer	Fujian	277.6 (203.6–382.1)	1.3 (1–1.9)	801.1 (535.2–1166.3)	1.4 (0.9–2)	0.26 (−0.05 to 0.57)	230.2 (168.8–312.2)	1.2 (0.9–1.6)	358.5 (239–518.4)	0.7 (0.4–1)	−1.86 (−2.03 to −1.68)
Tracheal, bronchus, and lung cancer	Fujian	5799.6 (4483.3–7330.4)	28.6 (22.4–35.9)	20,713.8 (15,781.4–27,240.6)	37.6 (28.7–49.1)	0.9 (0.59–1.21)	5831.9 (4471.7–7389.1)	29.7 (23–37.3)	16,928.6 (12,640.1–22,741)	31.3 (23.5–41.6)	0.12 (−0.29 to 0.54)
Larynx cancer	Gansu	122.6 (92.1–160.9)	0.9 (0.7–1.2)	266.1 (186.6–385.3)	0.7 (0.5–1)	−0.73 (−1.07 to −0.38)	108.4 (81.4–139)	0.9 (0.7–1.1)	170.5 (120.2–236.9)	0.5 (0.3–0.7)	−1.91 (−2.08 to −1.74)
Tracheal, bronchus, and lung cancer	Gansu	2409.2 (1891.5–3017.1)	18.3 (14.5–22.6)	7397.9 (5593.6–9612.9)	20.8 (15.9–27)	0.35 (0.18–0.53)	2458.2 (1918.4–3078.6)	19.6 (15.5–24.3)	7027.5 (5326.1–9137.7)	20.3 (15.4–26.1)	0.01 (−0.19 to 0.2)
Larynx cancer	Guangdong	1114.6 (761–1512.5)	2.4 (1.6–3.2)	3466.3 (2229.6–4934.3)	2.5 (1.6–3.5)	0.19 (0.01–0.37)	889.7 (631.5–1177.8)	2 (1.4–2.6)	1369.8 (916.9–1930.6)	1 (0.7–1.5)	−2.13 (−2.41 to −1.86)
Tracheal, bronchus, and lung cancer	Guangdong	16,672.1 (12,806–20,992.3)	36 (27.9–44.7)	59,255.8 (44,211.4–76,684.3)	44.5 (33.3–57.6)	0.55 (0.13–0.96)	16,698.5 (12,834.8–21,222.2)	37 (28.7–46.2)	44,955.5 (34,058.9–58,611.9)	34.5 (26.1–44.7)	−0.44 (−1.02 to 0.14)
Larynx cancer	Guangxi	631 (440.5–810.8)	2.1 (1.5–2.7)	1491.8 (977.4–2053.5)	2.2 (1.4–3)	0.3 −0.23 to 0.84)	562.6 (407.4–720.8)	1.9 (1.4–2.5)	911.9 (609.5–1239.9)	1.3 (0.9–1.8)	−1.02 (−1.38 to −0.66)
Tracheal, bronchus, and lung cancer	Guangxi	6296.7 (4973.3–7842.7)	21.4 (16.9–26.5)	23,407.7 (17,066.4–30,455.8)	34.3 (25.2–44.5)	1.57 (1.27–1.88)	6400.7 (5002.2–8013)	22.2 (17.5–27.7)	20,043.8 (14,680.7–26,069.8)	29.6 (21.7–38.5)	0.91 (0.53–1.29)
Larynx cancer	Guizhou	650.3 (432.2–879.3)	3.1 (2.1–4.2)	1210.3 (769.8–1741.6)	2.4 (1.6–3.5)	−0.84 (−1.01 to −0.67)	603 (407.1–815)	3 (2.1–4)	841.8 (546.2–1211.7)	1.7 (1.1–2.5)	−1.88 (−2.08 to −1.69)
Tracheal, bronchus, and lung cancer	Guizhou	5853.3 (4514–7408.1)	28.3 (22–35.3)	17,595.9 (12,057.8–24,126.2)	36 (24.7–49.4)	0.69 (0.43–0.95)	6032 (4658.6–7652.9)	30.2 (23.6–37.7)	17,449.3 (11,933.6–23,695.9)	36.3 (24.7–49.2)	0.49 (0.14–0.84)
Larynx cancer	Hainan	180.2 (114.7–237.7)	3.9 (2.4–5.1)	441.6 (267.8–658.7)	3.5 (2.1–5.1)	−0.33 (−0.61 to −0.05)	154.3 (104.9–205.5)	3.5 (2.3–4.6)	263.5 (169.4–388.9)	2.1 (1.4–3.1)	−1.61 (−1.76 to −1.46)
Tracheal, bronchus, and lung cancer	Hainan	1351.3 (1045.4–1769.5)	29.8 (23.3–38.6)	4265 (3065.7–5657.7)	34.4 (25–45.5)	0.46 (0.44–0.48)	1381.6 (1071.7–1793.5)	31.2 (24.4–40)	3950.6 (2888.9–5261.2)	32.2 (23.7–42.6)	0.05 (−0.1 to 0.2)
Larynx cancer	Hebei	699.2 (494.5–940.4)	1.5 (1.1–2.1)	1829.5 (1309.2–2508.8)	1.6 (1.1–2.2)	0.11 (0.01–0.22)	578.5 (410.3–777.9)	1.3 (1–1.8)	1035.8 (736.6–1421.1)	0.9 (0.7–1.3)	−1.23 (−1.37 to −1.09)
Tracheal, bronchus, and lung cancer	Hebei	11,374 (8848–14,584)	25.6 (20.1–32.5)	41,429.7 (29,313.5–54,482.6)	37.1 (26.2–48.8)	1.09 (0.6–1.58)	11,632.8 (8971.5–14,841.6)	27.2 (21.3–34.3)	37,436.4 (26,535.6–50,075.1)	34.3 (24.2–45.4)	0.59 (0.02–1.17)
Larynx cancer	Heilongjiang	830.7 (564.7–1063.8)	4 (2.7–5)	1876.7 (1147–2623)	3.1 (1.9–4.3)	−0.72 (−0.88 to −0.56)	676.7 (465.9–869.3)	3.4 (2.4–4.4)	1032.9 (667–1453.3)	1.8 (1.2–2.5)	−2.1 (−2.27 to −1.93)
Tracheal, bronchus, and lung cancer	Heilongjiang	13,715.1 (10,975.2–16,711.7)	68.2 (55.4–82.8)	40,008.3 (30,695.2–51,333.2)	70.7 (55.3–90.1)	0.1 (−0.05 to 0.25)	13,647.7 (10,942.4–16,628.6)	71 (57.7–85.6)	35,737.6 (27,765.5–45,223.8)	64.9 (50.7–81.8)	−0.33 (−0.47 to −0.19)
Larynx cancer	Henan	640.3 (511.5–800.5)	1 (0.8–1.3)	1729.9 (1225.3–2405.7)	1.2 (0.8–1.6)	0.51 (0.35–0.67)	542 (430.7–681.4)	0.9 (0.7–1.1)	877.3 (614.7–1209.8)	0.6 (0.4–0.8)	−1.31 (−1.43 to −1.2)
Tracheal, bronchus, and lung cancer	Henan	13,494.5 (11,315.4–16,155.2)	22.1 (18.5–26.2)	54,030.2 (41,147.5–69,965.6)	37.9 (29.1–48.3)	1.77 (1.37–2.18)	13,844.1 (11,468.5–16,655.8)	23.5 (19.5–28)	49,873.3 (37,187–64,205.1)	35.4 (26.7–45.4)	1.31 (0.82–1.81)
Larynx cancer	Hong Kong	156.2 (118.3–188.5)	2.7 (2–3.2)	192.1 (134–266.7)	1.2 (0.9–1.7)	−2.55 (−2.68 to −2.43)	100.7 (78.1–121.7)	1.8 (1.4–2.1)	85.3 (59.1–117)	0.5 (0.4–0.7)	−3.84 (−4.12 to −3.57)
Tracheal, bronchus, and lung cancer	Hong Kong	2732.9 (2415.1–3093.5)	48.6 (42.9–54.8)	5255 (3930.4–6811.5)	33.5 (25.3–43.3)	−1.3 (−1.53 to −1.07)	2756.6 (2436.3–3124)	49.8 (43.9–56.5)	4783.9 (3523.4–6116.3)	29.9 (22.2–38.3)	−1.74 (−1.91 to −1.57)
Larynx cancer	Hubei	913.9 (684.1–1147)	2.4 (1.8–3)	2112.2 (1496.4–2991.2)	2.2 (1.6–3.1)	−0.03 (−0.59 to 0.54)	719.3 (545.7–913)	2 (1.5–2.5)	993.5 (692.6–1378.4)	1.1 (0.8–1.5)	−1.78 (−2.2 to −1.35)
Tracheal, bronchus, and lung cancer	Hubei	13,899.8 (11,326.3–16,737.5)	36.8 (30.2–43.7)	49,836.8 (35,925–65,312.9)	52.5 (38–68.5)	1.29 (0.9–1.68)	13,685.9 (11,249.2–16,570.1)	37.6 (31.2–44.9)	38,138.7 (28,061.1–50,569.5)	41.1 (30.1–54.1)	0.38 (0.02–0.75)
Larynx cancer	Hunan	964.2 (659.5–1219.6)	2.2 (1.5–2.7)	2392.9 (1572.7–3347.6)	2.2 (1.5–3.1)	0.17 (−0.05 to 0.38)	875.1 (608.9–1122.5)	2.1 (1.5–2.6)	1500.8 (1003.9–2151.5)	1.4 (1–2)	−1.21 (−1.27 to −1.14)
Tracheal, bronchus, and lung cancer	Hunan	13,104.9 (10,893.4–15,686.2)	29.4 (24.6–35)	43,692.2 (33,149.3–56,443.6)	40.9 (31.2–52.9)	1.09 (0.85–1.33)	13,292.8 (10,987.8–15,965.3)	30.9 (25.7–36.7)	37,618 (28,590.6–49,277)	35.6 (27.1–46.5)	0.42 (0.1–0.74)
Larynx cancer	Inner Mongolia	260.8 (187.8–347.9)	2.1 (1.5–2.8)	736.5 (520.2–1048.7)	1.9 (1.3–2.6)	−0.29 (−0.42 to −0.16)	216.8 (159.9–289.2)	1.9 (1.4–2.4)	383 (270.3–548.5)	1 (0.7–1.5)	−1.95 (−2.07 to −1.83)
Tracheal, bronchus, and lung cancer	Inner Mongolia	4923.5 (3716.2–6238.9)	40 (30.4–50.3)	17,491.9 (13,351–22,855.7)	47.6 (36.3–61.6)	0.44 (0.02–0.86)	4951.3 (3733.3–6322.9)	42.4 (32.6–53.7)	14,963.5 (11,415.5–19,384.1)	41.8 (32–53.8)	−0.22 (−0.76 to 0.32)
Larynx cancer	Jiangsu	630 (473.1–822.5)	1.1 (0.9–1.5)	1654.2 (1106–2370.3)	1.1 (0.8–1.6)	0.17 (−0.25 to 0.59)	520.2 (399.2–677.3)	1 (0.7–1.3)	794.4 (536.7–1133.5)	0.5 (0.4–0.8)	−1.78 (−1.97 to −1.6)
Tracheal, bronchus, and lung cancer	Jiangsu	18,676.5 (15,042.3–22,744.3)	33.5 (27.2–40.4)	65,468.5 (48,173.7–86,308.2)	44.5 (32.7–58.3)	0.92 (0.57–1.26)	18,766.1 (15,212.9–22,690.6)	34.7 (28.4–41.8)	53,035.8 (38,751.7–69,743.2)	36.5 (26.7–47.8)	0.12 (−0.22 to 0.46)
Larynx cancer	Jiangxi	602.4 (446.8–770)	2.4 (1.8–3.1)	1115.4 (771.2–1517)	1.8 (1.2–2.4)	−0.82 (−1.5 to −0.14)	544.2 (404.9–681.7)	2.3 (1.7–2.8)	633.2 (440.8–875.8)	1 (0.7–1.4)	−2.42 (−2.84 to −1.99)
Tracheal, bronchus, and lung cancer	Jiangxi	10,099.5 (8088.8–12,469.8)	41.1 (33.2–50.1)	24,742.7 (18,379.8–31,663.3)	40.5 (30.1–51.6)	−0.03 (−0.18 to 0.13)	10,370.8 (83,74.3–12,705.9)	43.5 (35.4–52.6)	22,240.8 (16,611.2–29,128.9)	37 (27.7–48)	−0.56 (−0.75 to −0.37)
Larynx cancer	Jilin	529.6 (361.8–680.9)	3.4 (2.3–4.3)	982.3 (611.4–1361.8)	2.3 (1.4–3.1)	−1.27 (−1.66 to −0.88)	450.7 (328.2–573)	3.1 (2.3–3.9)	522.4 (335.3–724.7)	1.3 (0.8–1.8)	−2.85 (−3.22 to −2.49)
Tracheal, bronchus, and lung cancer	Jilin	7275.1 (6002.3–8736.7)	47.6 (39.6–57)	21,747.5 (16,516.1–28,161.8)	52.4 (40.3–67.4)	0.24 (0.1–0.38)	7314.6 (6009.7–8849.1)	50.2 (41.8–60.4)	18,060 (13,756.3–23,354.5)	45 (34.7–57.8)	−0.48 (−0.74 to −0.22)
Larynx cancer	Liaoning	703.5 (468.7–1060.8)	2.4 (1.6–3.6)	2574.1 (1737.2–3598.5)	3.1 (2.1–4.3)	1.14 (0.5–1.78)	519.5 (351–789.9)	1.9 (1.3–2.9)	1094.2 (755.5–1510.1)	1.4 (0.9–1.9)	−0.75 (−1.33 to −0.18)
Tracheal, bronchus, and lung cancer	Liaoning	12,774.2 (9991.6–16,096.8)	45 (35.4–56.5)	49,364.7 (38,762–63,993)	61.5 (48.6–79.2)	1.19 (0.48–1.9)	13,091 (10,203.2–16,875.6)	48 (37.8–61.4)	44,619.6 (34,991.1–57,525.7)	56.9 (44.7–72.8)	0.73 (0.02–1.44)
Larynx cancer	Macao	5.7 (4.3–7.5)	2.1 (1.6–2.8)	18.3 (12.1–28.5)	1.8 (1.2–2.8)	−0.24 (−0.65 to 0.17)	4.1 (3.1–5.3)	1.5 (1.1–2)	7.4 (4.9–12)	0.8 (0.5–1.2)	−1.87 (−2.35 to −1.39)
Tracheal, bronchus, and lung cancer	Macao	116.4 (95.5–137.2)	42.5 (34.8–50.1)	300.8 (224.4–392.8)	31.2 (23.3–40.5)	−0.76 (−1.21 to −0.32)	122 (101–143.5)	44.6 (36.8–52.4)	272.2 (199.3–355.7)	28.7 (20.8–37.4)	−1.18 (−1.63 to −0.73)
Larynx cancer	Ningxia	24.1 (18–33.3)	1 (0.8–1.4)	75.6 (52.2–106.9)	0.9 (0.6–1.2)	−0.45 (−0.74 to −0.15)	20.9 (15.7–28.7)	1 (0.7–1.3)	41.6 (28.9–58.9)	0.5 (0.4–0.7)	−2.01 (−2.17 to −1.85)
Tracheal, bronchus, and lung cancer	Ningxia	635.2 (486.9–807.4)	27.6 (21.5–34.7)	2862.9 (2027.1–3898.4)	35.1 (24.9–47.2)	0.68 (0.47–0.9)	645.3 (493.4–826.1)	29.5 (23.1–37.4)	2466.6 (1776.8–3361.5)	31.3 (22.8–42.2)	0.03 (−0.36 to 0.43)
Larynx cancer	Qinghai	21.3 (15.5–29.7)	0.9 (0.7–1.2)	52.7 (36.7–79.3)	0.8 (0.5–1.2)	−0.46 (−0.57 to −0.35)	19.1 (14–26.5)	0.8 (0.6–1.2)	38.2 (26.8–54.8)	0.6 (0.4–0.8)	−1.23 (−1.42 to −1.03)
Tracheal, bronchus, and lung cancer	Qinghai	524.8 (410.5–681.7)	22.2 (17.5–28.2)	1954.3 (1415.1–2606.9)	30.1 (21.8–39.8)	0.84 (0.48–1.2)	529 (404.7–680.5)	23.6 (18.3–29.9)	1901.1 (1393.7–2516.9)	30.3 (22.4–39.9)	0.64 (0.19–1.1)
Larynx cancer	Shaanxi	334.3 (254.2–443.1)	1.5 (1.2–2)	734.4 (505.2–1047.8)	1.2 (0.8–1.7)	−0.6 (−1.02 to −0.17)	292.3 (222.1–385.8)	1.4 (1.1–1.8)	414.8 (289.9–580.6)	0.7 (0.5–1)	−2.15 (−2.4 to −1.9)
Tracheal, bronchus, and lung cancer	Shaanxi	5966.2 (4705.8–7478.9)	27.2 (21.7–33.7)	18,407.7 (13,288.8–25,028.3)	31.3 (22.7–42)	0.43 (0.39–0.47)	6086.7 (4748.9–7688)	28.9 (23.1–36)	16,664.1 (11,854.7–22,310.1)	28.9 (20.9–38.4)	−0.07 (−0.26 to 0.12)
Larynx cancer	Shandong	909.9 (668–1213.1)	1.4 (1–1.8)	2454.9 (1688.8–3491.8)	1.5 (1–2.1)	0.24 (−0.08 to 0.57)	759.5 (564.6–1012)	1.2 (0.9–1.6)	1191.8 (830.5–1704.3)	0.7 (0.5–1)	−1.69 (−1.95 to −1.42)
Tracheal, bronchus, and lung cancer	Shandong	21,604.4 (16,805.6–27,359.1)	33.4 (26.2–42.2)	77,225.2 (58,842.3–101,352.2)	46.7 (35.6–60.9)	0.95 (0.57–1.33)	22,361.6 (17,436.2–28,596.9)	35.8 (28.3–45)	71,354.1 (53,958.6–93,289.3)	43.7 (33.3–56.7)	0.46 (−0.03 to 0.95)
Larynx cancer	Shanghai	277.7 (197.1–378.7)	1.9 (1.3–2.5)	587.5 (377.4–903.2)	1.4 (0.9–2.1)	−1.24 (−1.6 to −0.87)	200 (142.2–278.1)	1.4 (1–1.9)	291.2 (187.7–438.1)	0.7 (0.5–1)	−2.52 (−2.9 to −2.15)
Tracheal, bronchus, and lung cancer	Shanghai	6219.5 (4871.5–7944.8)	42.8 (33.9–54.1)	18,519.8 (13,629.2–24,832.4)	43.4 (32.2–57.6)	−0.21 (−0.94 to 0.53)	5996.3 (4655.3–7552.7)	42.4 (33.3–53.4)	15,095.3 (11,207.2–19,901.2)	35.8 (26.7–47.3)	−0.83 (−1.61 to −0.05)
Larynx cancer	Shanxi	424.5 (303.9–578.2)	2.1 (1.5–2.8)	1016.5 (698.7–1489.9)	1.9 (1.3–2.7)	−0.24 (−0.58 to 0.09)	347.6 (250–463)	1.8 (1.3–2.4)	534.8 (376–764.4)	1 (0.7–1.4)	−1.77 (−2.11 to −1.42)
Tracheal, bronchus, and lung cancer	Shanxi	6087.3 (4613–7808.6)	30.6 (23.6–38.6)	20,403 (15,130.8–27,701.8)	40 (29.8–53.9)	0.87 (0.75–1)	6193.6 (4681.3–8064.9)	32.4 (25.1–41.6)	18,718.2 (13,730.4–25,159.8)	37.6 (27.7–49.8)	0.43 (0.23–0.64)
Larynx cancer	Sichuan	1260.1 (922.6–1636.8)	1.5 (1.1–1.9)	2600.5 (1852.8–3682.5)	1.8 (1.3–2.5)	0.69 (0.27–1.12)	1082.1 (801.4–1397.8)	1.4 (1–1.7)	1378.9 (975.3–1951.2)	1 (0.7–1.3)	−1.13 (−1.35 to −0.9)
Tracheal, bronchus, and lung cancer	Sichuan	23,682.3 (18,787.8–29,539.7)	28.8 (23–35.7)	72,290.6 (52,684.3–95,562.1)	50 (36.6–65.9)	1.78 (1.59–1.97)	23,923.8 (18,766.6–29,826.3)	30.2 (23.9–37.4)	62,948.5 (46,501.2–83,345.4)	43.7 (32.4–57.7)	1.13 (0.78–1.47)
Larynx cancer	Taiwan	351.8 (327–379.5)	2.1 (2–2.3)	715.9 (626.6–799.9)	1.7 (1.5–1.9)	−0.91 (−1.44 to −0.38)	186.1 (174–199.2)	1.2 (1.1–1.3)	250.3 (223.4–277.8)	0.6 (0.5–0.7)	−2.42 (−2.97 to −1.87)
Tracheal, bronchus, and lung cancer	Taiwan	4140.5 (3924.5–4340)	25.7 (24.3–26.9)	12,202.7 (10,969.6–13,235)	28.7 (25.8–31)	0.03 (−0.98 to 1.06)	4112.3 (3906.5–4319.2)	26.2 (24.8–27.6)	11,592.6 (10,418.7–12,597)	27 (24.4–29.3)	−0.18 (−1.02 to 0.67)
Larynx cancer	Tianjin	167.3 (124–218.9)	2.3 (1.7–2.9)	478.7 (330.7–669.6)	2.1 (1.5–2.9)	−0.22 (−0.33 to −0.1)	113.6 (83.5–147.4)	1.7 (1.2–2.1)	211.6 (147.8–299.9)	1 (0.7–1.4)	−1.76 (−1.89 to −1.64)
Tracheal, bronchus, and lung cancer	Tianjin	3558.3 (2768.9–4372.8)	49.6 (38.8–60.6)	13,906.4 (10,609.1–17,803.6)	63 (48.2–80.6)	0.68 (0.33–1.03)	3441.3 (2687.7–4279)	49.9 (39.2–61.4)	11,649.4 (8835.9–14,914.2)	54.2 (41.4–68.9)	0.16 (−0.22 to 0.54)
Larynx cancer	Tibet	13 (8.6–20.5)	0.9 (0.6–1.3)	14.5 (9.2–22.8)	0.4 (0.3–0.7)	−2.18 (−2.52 to −1.85)	12.1 (8.1–18.2)	0.8 (0.6–1.2)	11.8 (7.4–18.6)	0.4 (0.2–0.6)	−2.6 (−2.91 to −2.29)
Tracheal, bronchus, and lung cancer	Tibet	180.1 (135.8–229.4)	12 (9.1–15.1)	307.6 (216.5–425.9)	9.8 (7–13.5)	−0.79 (−1.09 to −0.5)	183.7 (138.5–234)	12.5 (9.5–15.8)	310 (220.2–424.5)	10.2 (7.3–13.8)	−0.83 (−1.15 to −0.5)
Larynx cancer	Xinjiang	91.3 (66.5–129.4)	1 (0.8–1.5)	202.4 (129–294.5)	0.7 (0.5–1)	−1.14 (−1.46 to −0.82)	83.8 (61.8–117.4)	1 (0.8–1.4)	141.7 (93.3–206.7)	0.5 (0.4–0.8)	−2.04 (−2.2 to −1.88)
Tracheal, bronchus, and lung cancer	Xinjiang	2013.7 (1589.2–2566.2)	23.1 (18.3–29.3)	6154.6 (4472.3–8421.5)	22.9 (16.8–31.1)	−0.03 (−0.09 to 0.04)	2068.8 (1642–2630.9)	24.6 (19.7–31.2)	6082.1 (4397.7–8279.2)	23.4 (17.1–31.4)	−0.19 (−0.34 to −0.05)
Larynx cancer	Yunnan	405.6 (298.9–536.8)	1.6 (1.2–2.2)	912.6 (639.1–1252)	1.5 (1–2)	−0.3 (−0.42 to −0.18)	374.3 (272.4–496.7)	1.6 (1.2–2.1)	630 (440–857.8)	1.1 (0.7–1.4)	−1.32 (−1.41 to −1.22)
Tracheal, bronchus, and lung cancer	Yunnan	6126.8 (4695–7879.7)	24.9 (19.4–31.7)	19,227.8 (13,329–26,015.7)	32 (22.4–43.2)	0.81 (0.63–0.99)	6276.9 (4769.3–8121.8)	26.4 (20.4–33.5)	17,684.1 (12,561.7–23,589.4)	30 (21.5–39.7)	0.37 (0.09–0.65)
Larynx cancer	Zhejiang	539.1 (385–726.6)	1.5 (1.1–2)	1834.4 (1254.5–2608.2)	1.8 (1.3–2.6)	0.61 (0.44–0.77)	410.1 (294.4–547.7)	1.2 (0.9–1.6)	660.6 (453.4–944.1)	0.7 (0.5–1)	−1.92 (−2.1 to −1.74)
Tracheal, bronchus, and lung cancer	Zhejiang	13,091.3 (10,148–16,324.1)	38 (29.9–47.3)	50,045 (36,628–67,277.2)	51.1 (37.6–68.3)	0.83 (0.41–1.26)	13,374.9 (10,369.5–16,906.4)	40.1 (31.5–50.2)	42,844.4 (31,204.7–56,520.4)	44.7 (32.7–58.3)	0.19 (−0.29 to 0.66)

Estimated annual percentage change = EAPC.

### ASIR and ASMR of respiratory tract cancers in China, 2021

**Fig. 2:**
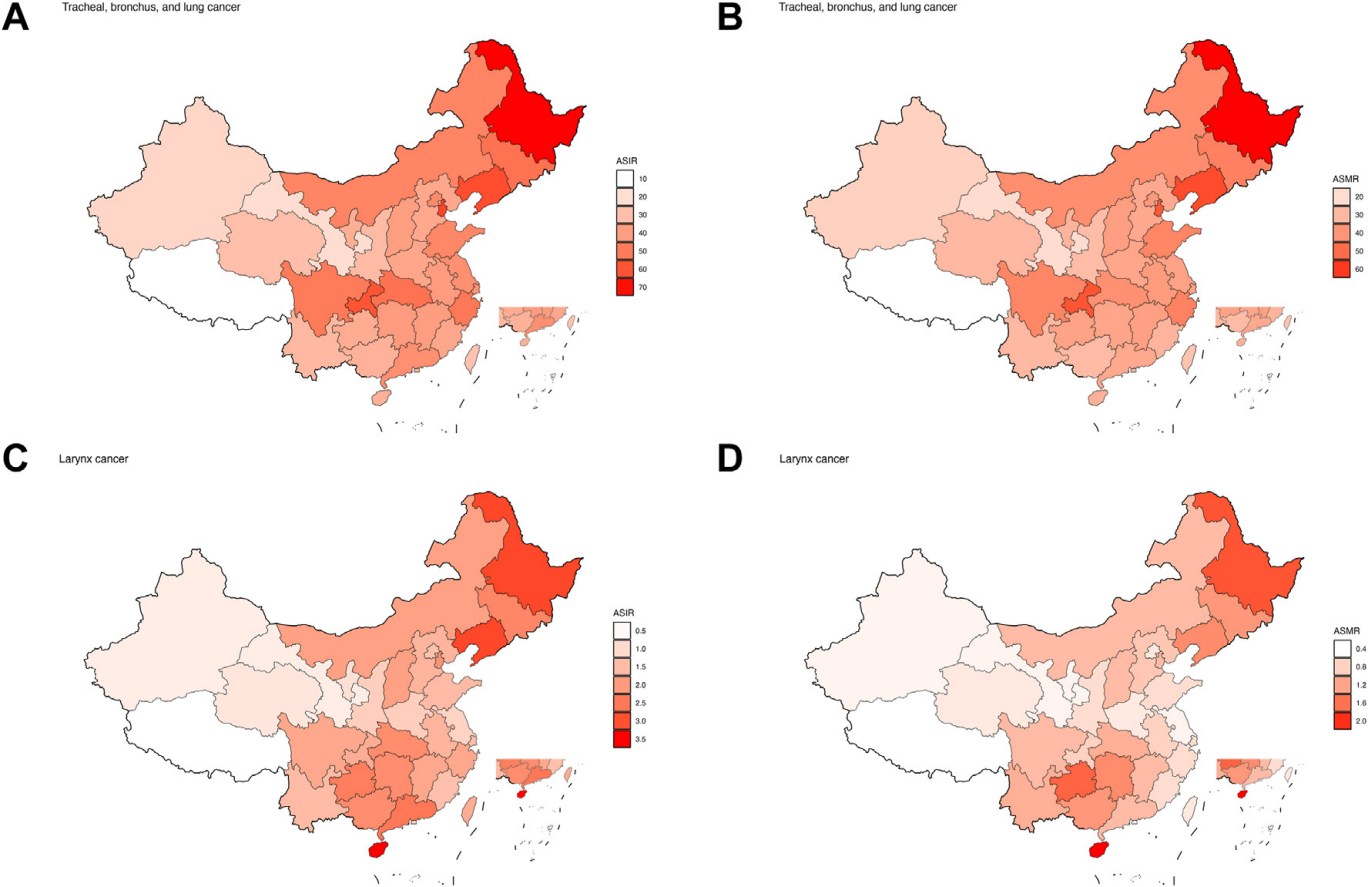
Burden of respiratory tract cancers by province in China, 2021. (A) ASIR of Tracheal, bronchus, and lung cancer. (B) ASMR of Tracheal, bronchus, and lung cancer. (C) ASIR of Larynx cancer. (D) ASMR of Larynx cancer; ASIR = Age-standardized incidence rate (per 100,000 person-years); ASMR = Age-standardized mortality rate (per 100,000 person-years).

In 2021, ASIR of respiratory tract cancers demonstrated a notable geographical heterogeneity, northeastern China reported the highest, while the western regions were the lowest. However, Hainan, a province in the southern China, did not adhered to the aforementioned geographical distribution pattern, showing the highest ASIR and ASMR of larynx cancer. Specifically, among the provinces in China, Heilongjiang, Tianjin, and Liaoning have the highest ASIR for tracheal, bronchus, and lung cancer. Conversely, the lowest ASIR for tracheal, bronchus, and lung cancer was documented in Xinjiang, Gansu, and Tibet (Supplementary Material pp 38–40). Hainan, Heilongjiang, and Liaoning have the highest ASIR for larynx cancer. Gansu, Xinjiang, and Tibet have the lowest ASIR (Supplementary Material pp 41–43). In 2021, the provinces with the highest ASMR for tracheal, bronchus, and lung cancer are Heilongjiang, Liaoning, and Chongqing. Xinjiang, Gansu, and Tibet reported the lowest ASMR (Supplementary Material pp 44–46). The provinces with the highest ASMR for larynx cancer are Hainan, Heilongjiang, and Guizhou. Ningxia, Gansu, and Tibet have the lowest ASMR (Supplementary Material pp 47–49) (Fig. 2, Table 1).

### EAPC of ASIR and ASMR of respiratory tract cancers in China, 1990–2021

From 1990 to 2021, the EAPC of ASIR for tracheal, bronchus, and lung cancer in China was 0.88 (95% UI: 0.63–1.14), and the EAPC of ASMR was 0.29 (95% UI: −0.05 to 0.62). Both ASIR and ASMR showed an overall increasing trend. Among the 34 provinces, 28 provinces demonstrated an increasing pattern in ASIR and EAPC, with the highest increases observed in Sichuan, Henan, and Guangxi. Conversely, six provinces, including Hong Kong, Tibet, Macao, Shanghai, Jiangxi, and Xinjiang, exhibited a decreasing trend in ASIR and EAPC. Among the 34 provinces, 20 provinces showed an increasing trend in ASMR EAPC, with the highest increases observed in Henan, Sichuan, and Guangxi. Conversely, 14 provinces demonstrated a decreasing trend in ASMR EAPC (Supplementary Material pp 50–56). From 1990 to 2021, for larynx cancer in China, the EAPC of ASIR was 0.04 (−0.22 to 0.3), and the EAPC of ASMR was −1.69 (−1.8 to −1.59). ASIR exhibited an overall decreasing trend, while ASMR showed an increasing trend. Among the 34 provinces, the EAPC of ASIR of 12 provinces were increased, with the highest observed in Liaoning, Sichuan, and Zhejiang. Anhui province showed no notable change of EAPC of ASIR. Additionally, 21 provinces demonstrated a reduction in EAPC of ASIR, with the most prominent declines reported in Hong Kong, Tibet and Jilin. All provinces exhibited a decrease in EAPC of ASMR, with the most notable decreases observed in Hong Kong, Jilin, and Tibet (Supplementary Material pp 57–64) (Table 1).

### Percentage change of ASIR and ASMR of respiratory tract cancers in China, 1990–2021

**Fig. 3:**
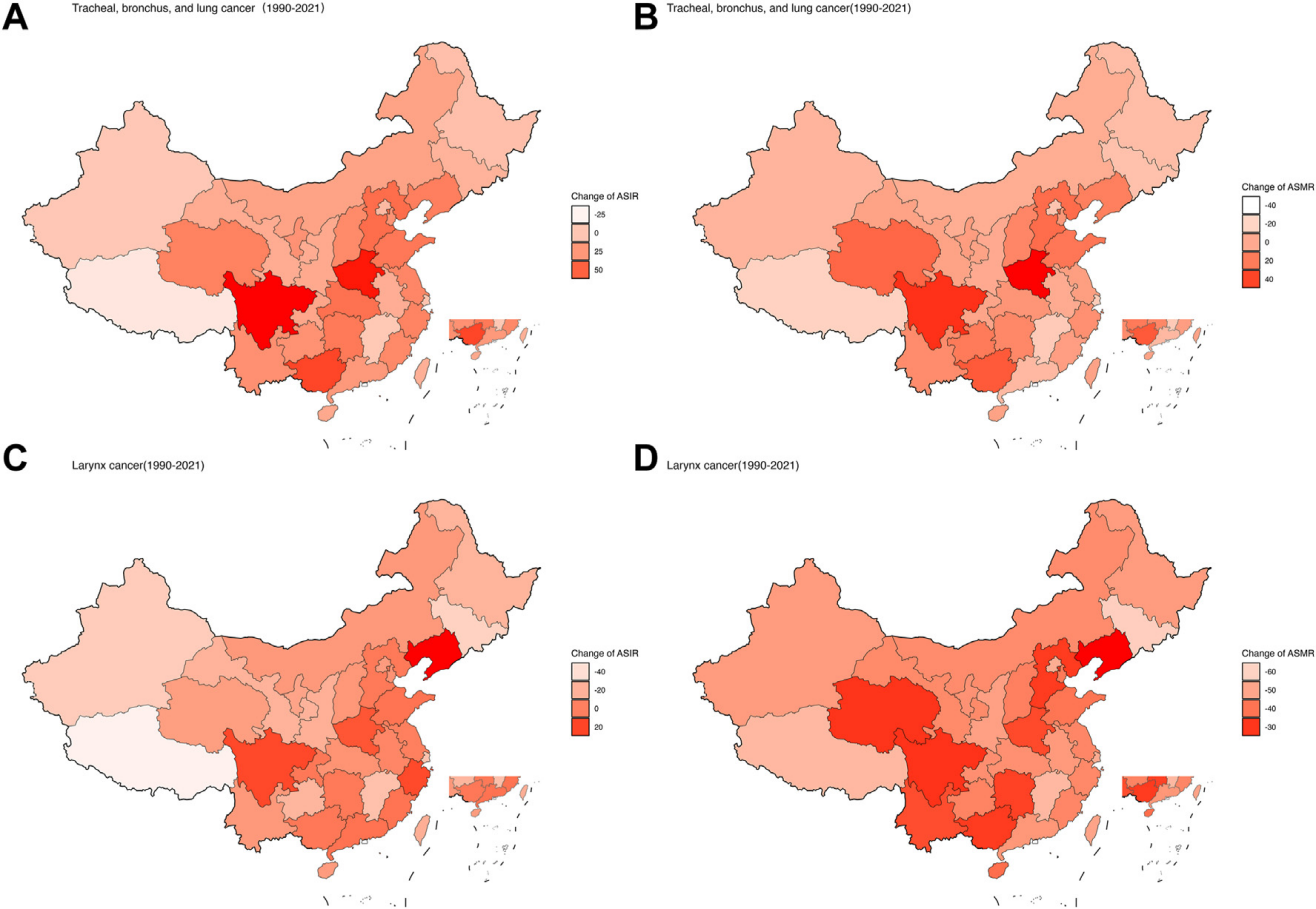
Changes of age-standardized percentage of respiratory cancer in provinces of China from 1990 to 2021. (A) Tracheal, bronchus, and lung cancer change of ASIR. (B) Tracheal, bronchus, and lung cancer change of ASMR. (C) Larynx cancer change of ASIR. (D) Larynx cancer change of ASMR.

From 1990 to 2021, the geographical distribution of respiratory tract cancers in China shows an increase mainly concentrated in the central and eastern regions, while decreases are primarily observed in western provinces. Specifically, the percentage change of ASIR for tracheal, bronchus, and lung cancer in China was 32.92% (95% UI: 1.21–71.31), and the percentage change of ASMR was 12.17% (95% UI: 13.83–43.73). Both ASIR and ASMR show an overall increasing trend. Among the 34 provinces, 29 provinces demonstrate an increasing trend in ASIR percentage change, with the highest increases observed in Sichuan, Henan, and Guangxi. Conversely, ASIR percentage change decreased in five provinces, including Xinjiang, Jiangxi, Tibet, Macao, and Hong Kong. Among the 34 provinces, 22 provinces show an increasing trend in ASMR percentage change, with the highest increases observed in Henan, Sichuan, and Guangxi. Conversely, 12 provinces demonstrate a decreasing trend in ASMR percentage change, with the most notable decreases seen in Hong Kong, Macao and Tibet (Supplementary Material pp 58–72). From 1990 to 2021, for larynx cancer in China, the percentage change of ASIR was −1.72% (95% UI: −26.16 to 29.69), and the percentage change of ASMR was −41.02% (95% UI: −55.62 to −23.44). ASIR and ASMR exhibit an overall decreasing trend. Among the 34 provinces, 12 provinces show an increasing trend in ASIR percentage change, with the most notable increases observed in Liaoning, Sichuan, and Zhejiang. 22 provinces demonstrate a decreasing trend in ASIR percentage change, with the most notable decreases seen in Hong Kong, Tibet, and Jilin. Among the 34 provinces, all provinces exhibit a decrease in ASMR percentage change, with the most notable decreases observed in Hong Kong, Jilin, and Tibet (Supplementary Material pp 73–80) (Fig. 3).

### Sex—specific burden of respiratory tract cancers by province in China, 2021

**Fig. 4:**
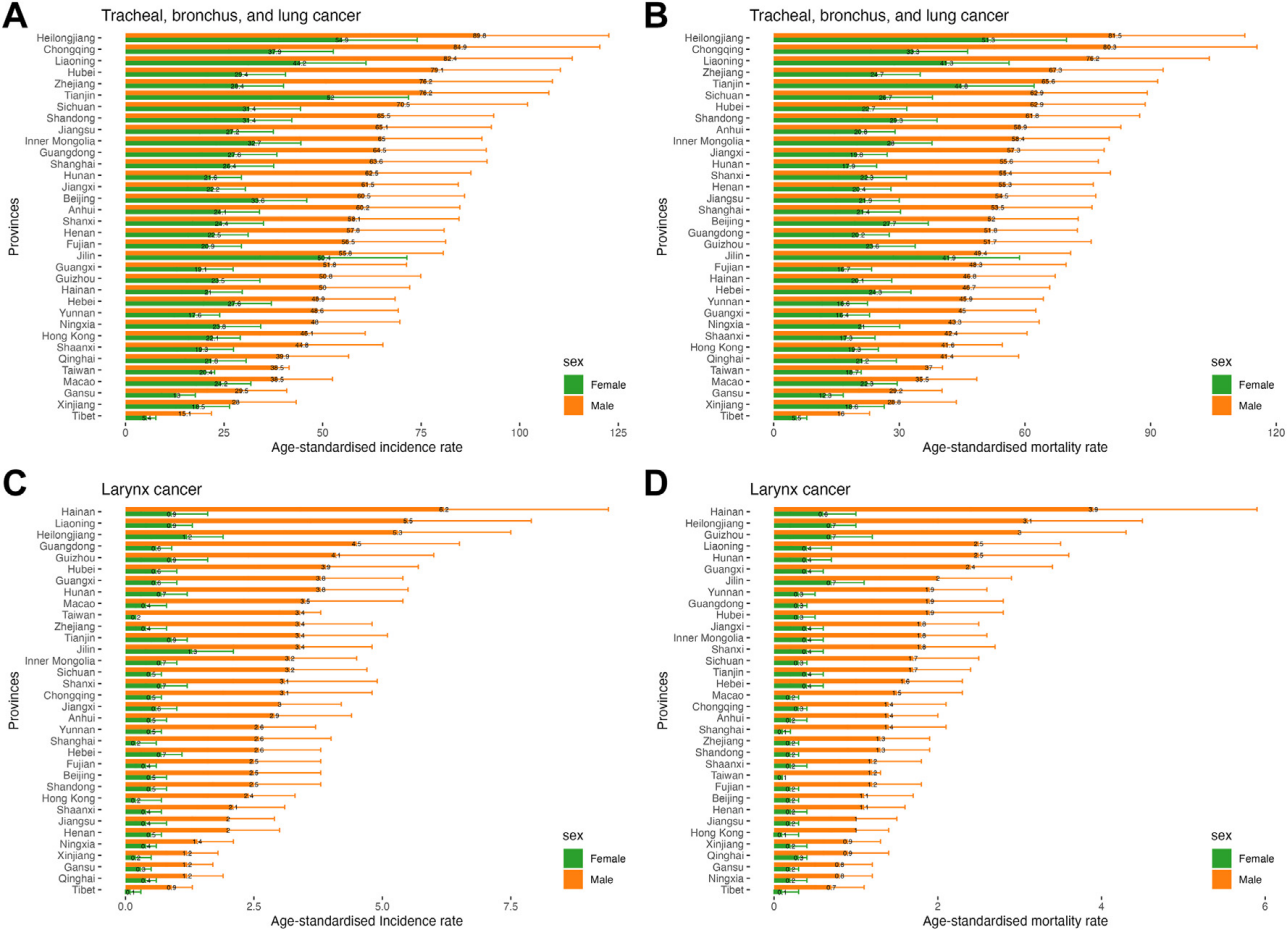
Female and male burden of respiratory tract cancers by province in China, 2021. (A) ASIR of tracheal, bronchus, and lung cancer. (B) ASMR of tracheal, bronchus, and lung cancer. (C) ASIR of larynx cancer. (D) ASMR of larynx cancer.

In 2021, the burden of respiratory cancer in China and its provinces was consistently higher in males than females. The ASIR for male tracheal, bronchus, and lung cancer was 62.63 (95% UI: 46.50–79.90) per 100,000 person-years, while females reported 28.16 (95% UI: 22.22–34.90) per 100,000 person-years. The ASIR for males was approximately 2.2 times higher than that for females nationally, with male ASIR notably higher than females across all provinces of China. The ASMR for male tracheal, bronchus, and lung cancer was 56.43 (95% UI: 42.27–72.41) per 100,000 person-years, compared to 24.42 (95% UI: 18.93–30.32) per 100,000 person-years for females. The ASMR for males was approximately 2.3 times higher than that for females nationally, with male ASMR notably higher than females across all provinces of China (Supplementary Material pp 81–93). For larynx cancer, the ASIR for males was 3.12 (95% UI: 2.34–4.04) per 100,000 person-years, compared to 0.58 (95% UI: 0.35–0.79) per 100,000 person-years for females. The ASIR for males was approximately 5.4 times higher than that for females nationally, with male ASIR notably higher than females across all provinces of China. The ASMR for males was 1.68 (95% UI: 1.27–2.15) per 100,000 person-years, while for females, it was 0.30 (95% UI: 0.18–0.43) per 100,000 person-years. The ASMR for males was approximately 5.6 times higher than that for females nationally, with male ASMR notably higher than females across all provinces of China (Supplementary Material pp 94–105) (Fig. 4).

### Age specific burden of respiratory tract cancers in China, 1990–2021

**Fig. 5:**
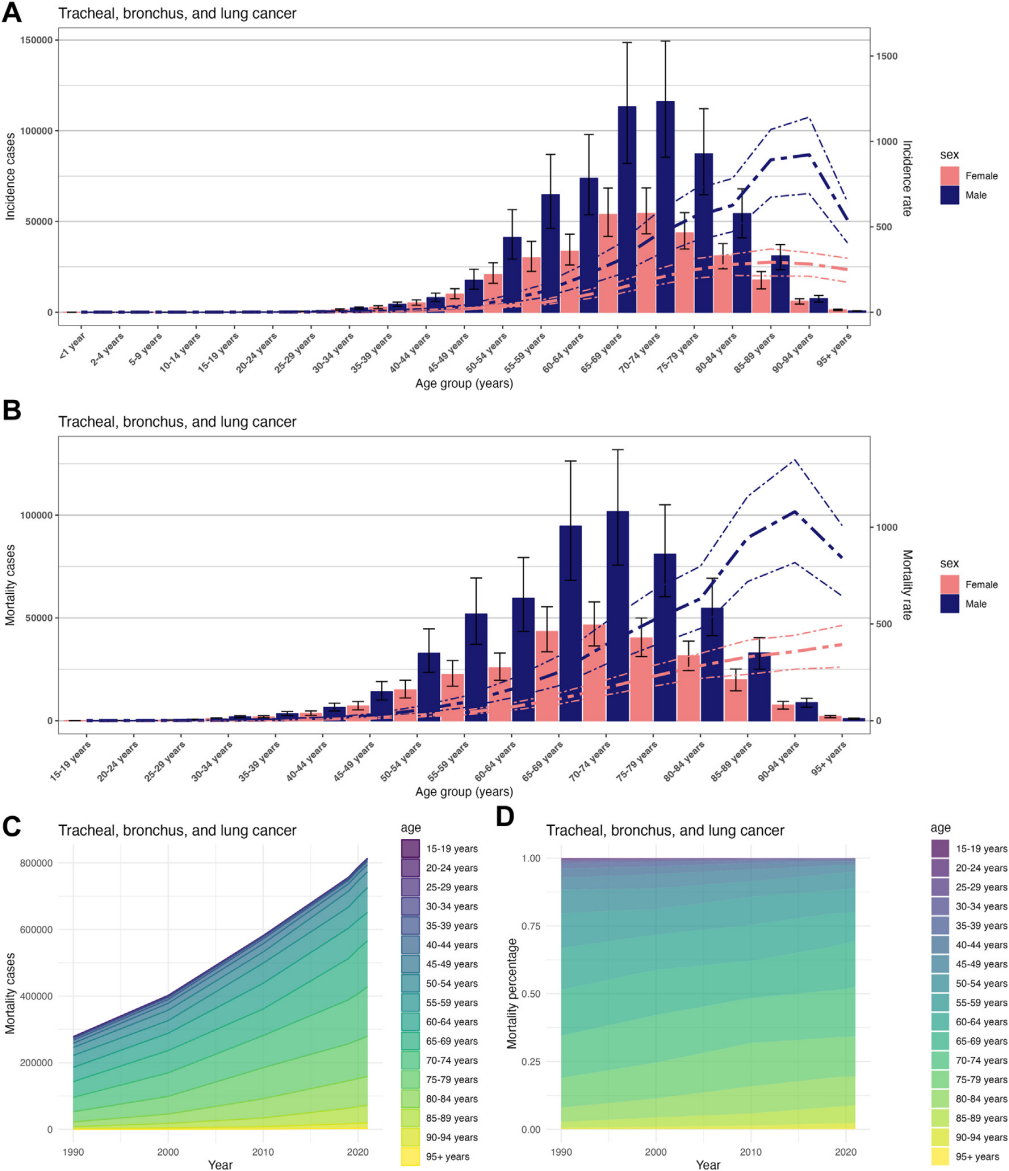
Age burden of tracheal, bronchus, and lung cancer in China from 1990 to 2021. (A) Incidence cases and incidence rate of tracheal, bronchus, and lung cancer in different age groups, 2021. (B) Mortality rate cases and mortality rate of tracheal, bronchus, and lung cancer in different age groups, 2021. (C) Changes of tracheal, bronchus, and lung cancer mortality rate cases in different age groups from 1990 to 2021. (D) Changes of tracheal, bronchus, and lung cancer mortality rate percentage in different age groups from 1990 to 2021.

**Fig. 6:**
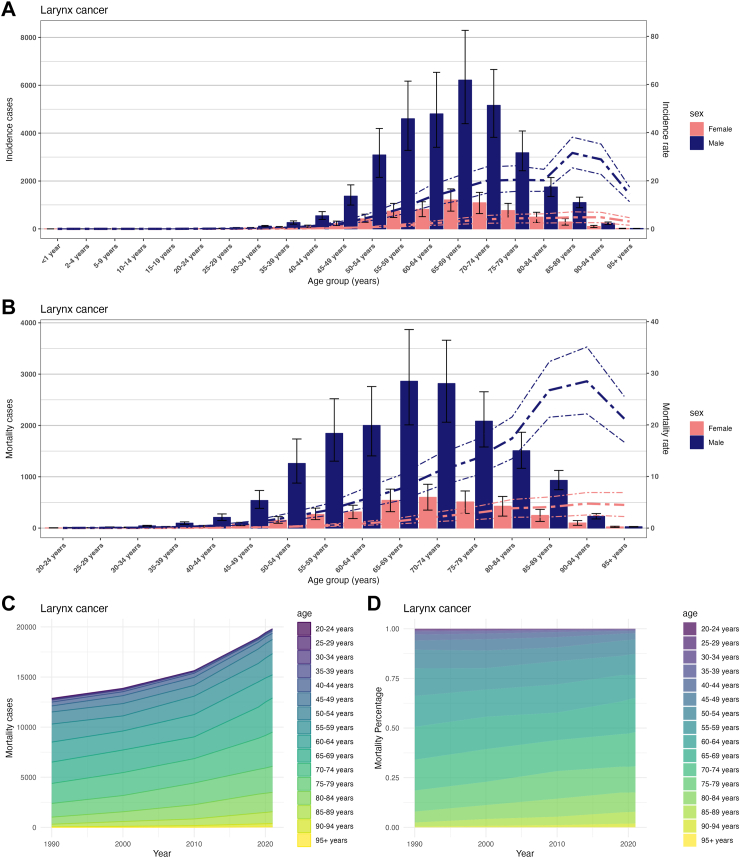
Age burden of larynx cancer in China from 1990 to 2021. (A) Incidence cases and incidence rate of larynx cancer in different age groups, 2021. (B) Mortality rate cases and mortality rate of larynx cancer in different age groups, 2021. (C) Changes of larynx cancer mortality rate cases in different age groups from 1990 to 2021. (D) Changes of larynx cancer mortality rate percentage in different age groups from 1990 to 2021.

In 2021, the incidence and mortality rates of tracheal, bronchus, and lung cancer were consistently higher in males than females across all age groups, showing a trend of initially increasing and then decreasing with age. The highest incidence and mortality rates were primarily observed in the age group of 75 years and above, with the 90–94 age group having the highest rates. Mortality and incidence rates were notably higher in males than in females across age groups, peaking and then declining as age increased. The highest number of incidence and deaths were concentrated in the 60–85 age group, with the 70–74 age group having the highest numbers. From 1990 to 2021, deaths from tracheal, bronchus, and lung cancer were predominantly in the 55–84 age group, showing a shift from younger (<44 years) to older (>80 years) age groups nationally (Supplementary Material pp 106–168) (Fig. 5). The incidence and mortality rates and numbers of larynx cancer displayed similar age and sex-dependent trends, consistently higher in males. These metrics peaked in the 85–89 age group. The most notable concentration of cases and deaths was observed in the 50–85 age range, particularly in the 65–69 age group. From 1990 to 2021, deaths from larynx cancer were predominantly in the 65–84 age group, showing a shift from younger (<44 years) to older (>80 years) age groups globally (Supplementary Material pp 169–229) (Fig. 6).

### Contribution of risk factors to all age mortality rate of respiratory tract cancers in 2021

In 2021, the contribution of risk factors to the overall mortality rate of respiratory tract cancers (combined for both sexes) varied. Across China, the primary risk factors contributing to tracheal, bronchus, and lung cancer mortality rates were smoking, air pollution, secondhand smoke, and other environmental risks (Supplementary Material pp 230–347). For larynx cancer, the main risk factors were Smoking, High alcohol use, occupational exposure to sulfuric acid and occupational exposure to asbestos (Supplementary Material pp 348–389, Supplementary Fig. S7).

## Discussion

Respiratory tract cancers, particularly tracheal, bronchus, lung, and larynx, are leading causes of health loss in China, with a rising trend in incidence and mortality.^9^ Cancer incidence varies by region, with high rates in eastern China and rising ASIR in some areas. Hainan has high larynx cancer rates. Successful control strategies in Hong Kong, Tibet, and Macao can inform other regions. Males and older adults bear a higher disease burden, calling for targeted interventions. Smoking and air pollution are key risk factors, necessitating public health measures to reduce cancer. Given the disparities in disease burden across regions, it is imperative for the Chinese government to augment public health initiatives, with a particular focus on rural areas where health infrastructure may be less robust.

Between 1990 and 2021, China experienced a marked increase in respiratory tract cancers, with tracheal, bronchial, and lung cancer cases increasing threefold and deaths nearly doubling. Laryngeal cancer rose less sharply. Despite medical progress, smoking, air pollution, and aging have contributed to higher cancer rates, underscoring the need for targeted interventions.^10^, ^11^, ^12^ Anti-smoking measures have been undermined by the large number of smokers.^13^ Industrialization and urbanization have worsened air pollution, especially PM2.5 and chemical pollutants, raising cancer risks.^14^ Additionally, the intensifying aging population in China has contributed to the rising incidence of respiratory tract cancers.^15^

Our study found a rise in respiratory tract cancer rates in China from 1990 to 2021, with eastern provinces showing higher burdens. Hainan has notably high larynx cancer rates, pointing to a need for in-depth analysis of contributing factors. Economically advanced provinces like Shandong, Sichuan, and Jiangsu face greater disease burdens, in contrast to areas with fewer cases such as Qinghai, Tibet, and Macao. Shandong, Sichuan, and Jiangsu are among the most economically developed provinces in China,^16^^,^^17^ with numerous industrial enterprises and dense populations.^18^ In the last three decades, China's industrialization and urbanization have caused severe pollution, especially air pollution with high emissions of PM2.5, sulfur dioxide, and nitrogen oxides, threatening respiratory health and increasing the risk of respiratory tract cancers. Economically developed provinces also face high smoking rates, particularly among males, exacerbating the respiratory cancer burden due to prolonged tobacco exposure and other factors.^19^ Additionally, the large population and high population density in these provinces imply not only higher pollution sources but also potential shortages or uneven distribution of public health facilities and medical resources, restricting early diagnosis and effective treatment and consequently increasing cancer mortality rates. Conversely, provinces with the lowest incidence and mortality rates, such as Qinghai and Tibet, have lower population densities and industrialization levels, which may explain the aforementioned disparities. Qinghai and Tibet are located on the Qinghai-Tibet Plateau, characterized by a relatively pristine natural environment and lower levels of industrialization,^20^ resulting in lighter atmospheric pollution, particularly with lower concentrations of harmful substances like PM2.5 compared to plain areas and industrial cities.^21^ Such an environment is beneficial for the respiratory health of residents, reducing the risk of respiratory tract cancers caused by environmental pollution.^22^ Moreover, residents in Qinghai and Tibet have unique lifestyle habits different from those in mainland China, such as engaging in more outdoor activities and having fewer habits of smoking and alcohol consumption. These healthy lifestyle habits contribute to lowering the risk of respiratory tract and other types of cancers.^23^ As a special administrative region of China, Macao has a high population density but a relatively high level of economic development, with higher standards and efficiency in public health, allocation of medical resources, and disease prevention, consequently reducing the incidence and mortality rates of respiratory tract cancers. Although medical resources in Qinghai and Tibet are not as abundant as those in coastal cities, the Chinese government has increased investment in healthcare in these provinces in recent years, further improving the overall health of local residents.^24^ Moreover, Heilongjiang, Tianjin, and Liaoning in northeastern China exhibit higher ASIR and ASMR. These provinces are early heavy industrial bases in China,^25^^,^^26^ with the early development of coal and other resource industries, leading to deteriorating air quality and long—term exposure of residents to carcinogenic substances. Moreover, smoking is widespread in these provinces,^27^ and the cold climate in winter increases the demand for coal—fired heating,^28^ further worsening air pollution conditions and consequently increasing the risk of respiratory tract cancers in these provinces. In 2021, regional differences in respiratory tract cancer burdens were evident in China, with Shandong, Sichuan, and Jiangsu having high incidences of tracheal, bronchus, and lung cancers. Northeastern provinces also reported high ASIR. Hainan showed high rates of larynx cancer, contrasting the norm. Studies, including one by,^29^ suggest spatial factors influence larynx cancer burdens, important for Hainan. Western regions like Qinghai, Tibet, and Macao saw fewer cases and deaths, with Xinjiang, Gansu, and Tibet having lower rates of tracheal, bronchus, and lung cancers, indicating a lighter burden.

This study from 1990 to 2021 used EAPC and changes in ASIR and ASMR to measure respiratory tract cancer burden in China, revealing regional differences. Most provinces showed increasing cancer rates, except Hong Kong, Tibet, and Macao, which had decreases, suggesting effective control. Larynx cancer incidence varied by region, but mortality rates fell nationwide. The study calls for policies to address cancer disparities, with higher burdens in central and eastern provinces. In 2021, male cancer rates exceeded females, consistent with global trends,^30^^,^^31^ necessitating a greater focus on understanding the impact of sex differences in tracheal, bronchus, and lung cancer within China. However, research by Stapelfeld et al.^32^ indicated that the risk of lung cancer among female smokers is twice that of male smokers. Yet, in reality, the incidence rate of lung cancer is notably higher in males than females. This discrepancy may be related to the influence of female sex hormones and the propensity for female smoking to cause greater damage to the lungs, warranting further detailed investigation into these mechanisms. Specifically, regarding sex differences in respiratory tract cancers in China, in terms of tracheal, bronchus, and lung cancer, the ASIR and ASMR for males are approximately 2.2 and 2.3 times higher than those for females, respectively. However, in Taiwan, a study by Chien et al.^33^ found that females in Taiwan generally have a higher incidence rate of lung adenocarcinoma than males, and this difference seems unaffected by smoking status. Sex-specific risk factors for lung adenocarcinoma in Taiwan may outweigh smoking, influencing prevention strategies. This contradicts our study on all lung cancer types in China, where a Taiwan-specific study showed varying incidence rates. Further research is needed to confirm these differences. Larynx cancer rates are significantly higher in males, with a 5.4× higher ASIR and 5.6× higher ASMR than females, aligning with global trends showing higher male incidence rates (5.8 vs. 1.2 per 100,000 person-years).^6^ Additionally, sex disparities in larynx cancer incidence and mortality rates in the United States also reflect this sex difference, with males notably higher than females.^34^ These results fully illustrate the influence of sex factors on the burden of larynx cancer, necessitating greater emphasis on the male population when formulating policies in China. Furthermore, although the sex differences between males and females in respiratory tract cancers are more pronounced in most provinces of China, with both ASIR and ASMR being notably higher in males, the province of Jilin shows little difference in the incidence and mortality rates of tracheal, bronchus, and lung cancer between males and females. This indicates the possible presence of specific influencing factors in the province, requiring more detailed epidemiological and etiological research to identify specific influencing factors and reduce the disease burden in the province. Research on future risk prediction models for lung cancer has also found sex to be an important influencing factor,^35^ Valencia et al.'s^36^ study found that estrogen may affect lung cancer development and treatment response by activating the epidermal growth factor receptor (EGFR) signaling pathway and possibly increasing aromatase expression and activity. However, the mechanisms by which sex influences lung cancer incidence and treatment involve multiple aspects,^37^^,^^38^ including the roles of estrogen and estrogen receptor α (ERα) in non-small cell lung cancer (NSCLC), as well as their interactions with PD-L1 expression. Future molecular research should address lung cancer disparities in China, considering sex differences in smoking, occupational exposure, biology, genetics, health awareness, and behaviors. Notably, higher smoking rates and male dominance in high-risk jobs contribute to respiratory tract cancer burdens. The US Preventive Services Task Force (USPSTF) has set lung cancer screening guidelines based on age and smoking history to ensure equitable access, with a focus on enhancing screening for women.^39^ Referring to these guidelines, China can formulate corresponding public health screening policies, especially taking measures to reduce male smoking rates and protect against high-risk occupations associated with cancer incidence, which will be crucial for reducing the overall burden of respiratory tract cancers.

In China, respiratory tract cancer rates initially rise with age and then decline after 2021, with males consistently more affected than females. Older people, especially those aged 75 and over, show the highest incidence and mortality, peaking in the 90–94 age group. This age-related trend is mirrored in countries like Japan, the U.S., and Serbia, suggesting the importance of age-specific screening and quality measures for these cancers.^40^, ^41^, ^42^ Larynx cancer rates peak in the oldest age groups, notably 85–89 years, with a shift from younger to older patients from 1990 to 2021. This trend reflects societal and environmental changes affecting respiratory tract cancer demographics in China. Factors include rising life expectancy and the aging population's increased cancer risk, compounded by long-term unhealthy habits like smoking and alcohol use, especially in men. In 2021, within China, smoking, air pollution, secondhand smoke, and other environmental risks^43^ were the primary risk factors contributing to mortality from tracheal, bronchus, and lung cancer. Although there were slight variations in these risk factors among different provinces, smoking consistently remained the major risk factor. Smoking contributed notably to respiratory tract cancer deaths in all provinces, accounting for over 40% in each, followed by air pollution, with a contribution exceeding 10%. Conversely, the contributions of secondhand smoke and other environmental risks were relatively small.^44^ In contrast, smoking and high alcohol use were the main risk factors for larynx cancer, with smoking contributing to over 60% of deaths in each province. While there were slight differences in the relative contributions of these risk factors among provinces, the overall trends remained consistent. Smoking remains the primary risk factor for respiratory tract cancers. A survey on the current smoking situation in China found that between 2007 and 2018, the smoking rate among adults decreased from 30.8% to 26.7%.^45^ Despite differing smoking rates in urban and rural areas, future strategies must target rural young males, tobacco-growing regions, and those with chronic diseases. Chan KH et al.^27^ highlighted smoking's significant role in China's disease burden, especially in respiratory cancers like lung and larynx. Quitting smoking reduces mortality from various diseases, with a 90%+ relative decrease in risk persisting even 30 years post-cessation.^46^ Based on these observations, the Chinese government needs to further strengthen public health campaigns and education to enhance awareness of smoking hazards among the population, especially rural youth, thereby reducing smoking rates and secondhand smoke exposure. If necessary, stricter tobacco control legislation should be implemented, such as comprehensive smoking bans in public places, increasing tobacco taxes, and banning all tobacco product advertising and promotion activities, to accelerate the reduction of smoking rates.^47^ Regarding the secondary risk factors contributing to different respiratory tract cancers, targeted measures need to be taken based on the specific secondary risk factors for each type of cancer.

This study presents data on China's respiratory tract cancer burden from 1990 to 2021 but acknowledges variations in data collection and coding across provinces, impacting result consistency. It lacks histological classification and specific data for smokers versus non-smokers, missing detailed burden comparisons between these groups. Future research should address these gaps. Additionally, the study's difficulty in precisely gauging the impact of risk factors like smoking and air pollution, along with varying prevention and treatment measures, complicates accurate disease burden estimation.

This study analyzes respiratory tract cancer trends in China from 1990 to 2021, showing a significant rise in cases and deaths, particularly for tracheal, bronchial, and lung cancers, and an increase in ASIR and ASMR. The findings offer insights for cancer prevention and control in China and globally. The study highlights gender and age disparities, with males and the older people bearing a higher burden, suggesting the need for targeted prevention and early intervention. Tailored strategies, such as differential screening for high-risk individuals, especially in polluted areas or with family history, are crucial for early detection and intervention. Secondly, the study's findings echo those from other regions worldwide, as described in the systematic review of “Promoting lung cancer awareness, help-seeking, and early detection interventions”.^48^ Successful increases in awareness of lung cancer have been achieved through educational campaigns and promotional activities, and in some studies, decision aids have reduced decision conflicts, thereby increasing the acceptance rate of lung cancer screening. This shows that raising public awareness of respiratory tract cancers through education and public campaigns is key to improving early diagnosis and treatment success rates. Furthermore, the study points out that smoking and air pollution are the two main risk factors for respiratory tract cancers. This discovery aligns with global research findings, emphasizing the importance of tobacco control and air pollution reduction in reducing the future burden of respiratory tract cancers. For example, implementing effective tobacco control policies and improving air quality can significantly reduce the incidence and mortality rates of lung cancer. According to data from the World Health Organization, the number of smokers in the Western Pacific region accounts for one-third of the global smoking population, so this region should pay special attention to tobacco control.^49^^,^^50^ At the same time, considering the severity of air pollution in Asia, especially in the South Asia subregion, these areas should be focal points for improving air quality. Lastly, the study also highlights the differences between regions, which may be related to varying smoking rates, levels of air pollution, and unequal distribution of medical resources. This provides important information for public health policymakers worldwide, indicating that regional differences and the needs of specific population groups should be considered when developing prevention and control strategies. In summary, this study not only provides new scientific evidence and perspectives for China but also for other countries globally on the prevention and control of respiratory tract cancers. Targeted screening strategies, educational campaigns, and environmental interventions can effectively reduce the burden of respiratory tract cancers. Additionally, this study corresponds with the World Health Organization's comprehensive monitoring framework for the prevention and control of non-communicable diseases, emphasizing the importance of surveillance, prevention, and healthcare, and providing a scientific basis for achieving the non-communicable disease control targets in the United Nations Sustainable Development Goals.

In conclusion, future research and health policy formulation regarding respiratory tract cancers in China should focus on reducing the overall burden while considering provincial and sex differences. Targeted screening, educational campaigns, and environmental interventions are essential to mitigate the impact of these cancers. In addition, implementing effective tobacco control policies and improving air quality can significantly reduce the incidence and mortality rates of respiratory tract cancers.

## Contributors

XZL and QZY contributed equally to this work and are joint first authors. PPY, QRL, YH, and WYJ are joint senior authors. YH, PPY, WYJ, and QRL conceived and designed the study. YH, PPY, WYJ, and QRL supervised the study. QZY, LMP, YFY, SH, DDX, LHW, LK, YFN, and WYJ performed the statistical analysis. LRK, JH, JXQ, and CNL contributed to the analysis, or interpretation of data. All authors verified the data and had access to raw data. QZY, XZL, WYT, and QRL drafted the manuscript. All authors revised the manuscript and approved the final version before submission.

## Data sharing statement

Some of the data presented here are publicly available on the Global Health Data Exchange website and the other data are available from the corresponding author upon reasonable request.

## Editor note

The Lancet Group takes a neutral position with respect to territorial claims in published maps and institutional affiliations.

## Declaration of interests

All the authors declare no conflicts of interest.
