# Supplementary material for: Burden of respiratory tract cancers in China and its provinces, 1990–2021: a systematic analysis of the Global Burden of Disease Study 2021
Source: Lancet Reg Health West Pac. 2025 Jan 30;55:101485. doi: 10.1016/j.lanwpc.2025.101485 (PMC11833622; doi:10.1016/j.lanwpc.2025.101485)
Supplement: Supplementary Materials [file mmc1.pdf]

Supplementary material

Figure 7 Percentage contribution of risk factors to all-age mortality rate of respiratory tract cancers in 2021, for both sexes, China and by provinces

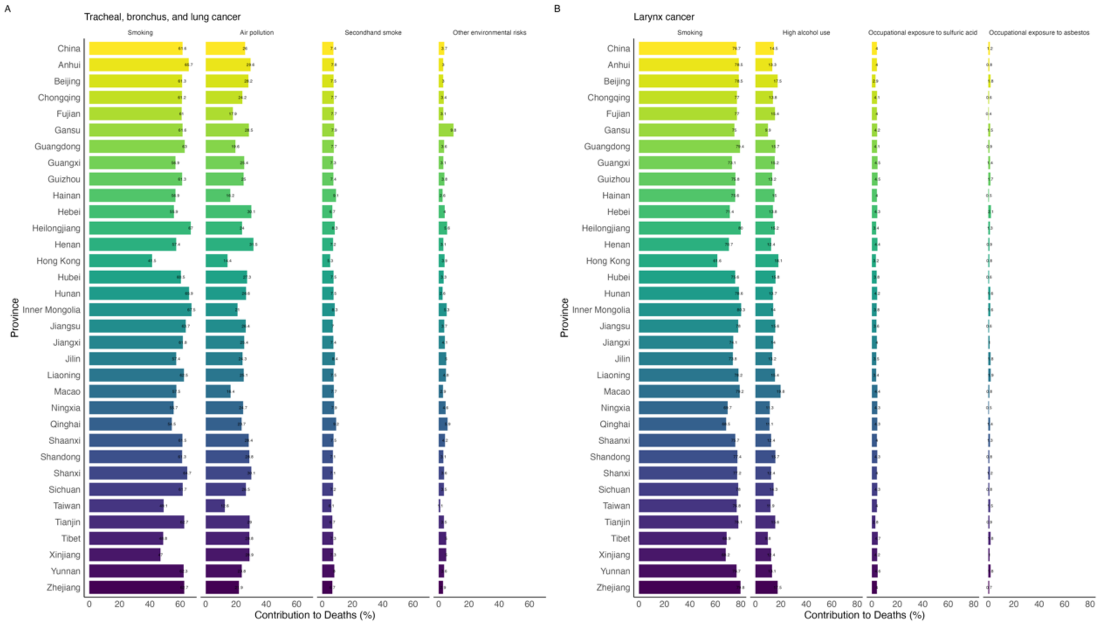

(A)Percentage contribution of risk factors of tracheal, bronchus, and lung cancer,2021. (B) Percentage contribution of risk factors of larynx cancer,2021

Appendix

Appendix 1：General trend of respiratory tract cancers in China, 1990–2021

measure	location	sex	age	cause	metric	year	val	upper	lower
Deaths	China	Both	All ages	Larynx cancer	Number	1990	12869.0357	15234.5474	10580.6819
Deaths	China	Both	All ages	Larynx cancer	Rate	1990	1.09388019	1.29495092	0.89936796
Deaths	China	Both	Age-standardized	Larynx cancer	Rate	1990	1.58992172	1.86499015	1.31467656
Deaths	China	Both	All ages	Tracheal, bronchus, and lung cancer	Number	1990	278235.229	322012.771	238517.798
Deaths	China	Both	All ages	Tracheal, bronchus, and lung cancer	Rate	1990	23.6502573	27.3713897	20.2742381
Deaths	China	Both	Age-standardized	Tracheal, bronchus, and lung cancer	Rate	1990	34.7395867	39.9812484	30.0689236
Deaths	China	Both	All ages	Larynx cancer	Number	2000	13867.6185	15619.5894	12088.0783
Deaths	China	Both	All ages	Larynx cancer	Rate	2000	1.10140375	1.24055002	0.96006786
Deaths	China	Both	Age-standardized	Larynx cancer	Rate	2000	1.32746686	1.48721158	1.15851043
Deaths	China	Both	All ages	Tracheal, bronchus,	Number	2000	402249.486	441062.17	364728.045

				and lung cancer					
Deaths	China	Both	All ages	Tracheal, bronchus, and lung cancer	Rate	2000	31.9477417	35.03035	28.9676874
Deaths	China	Both	Age-standardized	Tracheal, bronchus, and lung cancer	Rate	2000	38.8989308	42.553098	35.3066655
Deaths	China	Both	All ages	Larynx cancer	Number	2010	15633.967	17719.8956	13679.749
Deaths	China	Both	All ages	Larynx cancer	Rate	2010	1.16938633	1.32540919	1.02321512
Deaths	China	Both	Age-standardized	Larynx cancer	Rate	2010	1.09134742	1.22883983	0.95811174
Deaths	China	Both	All ages	Tracheal, bronchus, and lung cancer	Number	2010	581688.413	640349.275	530204.211
Deaths	China	Both	All ages	Tracheal, bronchus, and lung cancer	Rate	2010	43.5090132	47.8967166	39.6581081
Deaths	China	Both	Age-standardized	Tracheal, bronchus, and	Rate	2010	41.2956206	45.2685165	37.6245814

				lung cancer					
Deaths	China	Both	All ages	Larynx cancer	Number	2019	18932.3568	23867.0893	14943.0054
Deaths	China	Both	All ages	Larynx cancer	Rate	2019	1.33857198	1.68747174	1.05651338
Deaths	China	Both	Age-standardized	Larynx cancer	Rate	2019	0.9586724	1.19899201	0.76119605
Deaths	China	Both	All ages	Tracheal, bronchus, and lung cancer	Number	2019	757065.201	906536.779	624851.511
Deaths	China	Both	All ages	Tracheal, bronchus, and lung cancer	Rate	2019	53.5266833	64.094753	44.1787958
Deaths	China	Both	Age-standardized	Tracheal, bronchus, and lung cancer	Rate	2019	38.8387521	46.2165491	32.2126627
Deaths	China	Both	All ages	Larynx cancer	Number	2021	19814.0575	25279.3881	15420.2243
Deaths	China	Both	All ages	Larynx cancer	Rate	2021	1.39266307	1.77680267	1.0838354
Deaths	China	Both	Age-standardized	Larynx cancer	Rate	2021	0.93781119	1.18883941	0.73487808
Deaths	China	Both	All ages	Tracheal, bronchus, and lung cancer	Number	2021	814120.601	994858.212	652231.38

Deaths	China	Both	All ages	Tracheal, bronchus, and lung cancer	Rate	2021	57.221783	69.9252182	45.8431373
Deaths	China	Both	Age-standardized	Tracheal, bronchus, and lung cancer	Rate	2021	38.9684117	47.3442244	31.394936
Deaths	China	Both	All ages	Larynx cancer	Number	2020	19423.6696	23922.0367	14946.041
Deaths	China	Both	All ages	Larynx cancer	Rate	2020	1.36836054	1.68526194	1.05292013
Deaths	China	Both	Age-standardized	Larynx cancer	Rate	2020	0.95097791	1.16631921	0.73847843
Deaths	China	Both	All ages	Tracheal, bronchus, and lung cancer	Number	2020	787191.007	951670.391	639485.007
Deaths	China	Both	All ages	Tracheal, bronchus, and lung cancer	Rate	2020	55.4561076	67.043367	45.0505011
Deaths	China	Both	Age-standardized	Tracheal, bronchus, and lung cancer	Rate	2020	39.035346	46.955353	31.8382101
DALYs (Disabil	China	Both	All ages	Larynx cancer	Number	2010	410881.392	466902.113	357987.88

ity-Adjusted Life Years)									
DALYs (Disability-Adjusted Life Years)	China	Both	All ages	Larynx cancer	Rate	2010	30.7330239	34.9232505	26.7767056
DALYs (Disability-Adjusted Life Years)	China	Both	Age-standardized	Larynx cancer	Rate	2010	26.5030689	29.9716174	23.0769372
DALYs (Disability-Adjusted Life Years)	China	Both	All ages	Tracheal, bronchus, and lung cancer	Number	2010	14391425.7	15928929	13067135.6
DALYs (Disability-Adjusted Life Years)	China	Both	All ages	Tracheal, bronchus, and lung cancer	Rate	2010	1076.44697	1191.44883	977.392986
DALYs (Disability-Adjusted Life Years)	China	Both	Age-standardized	Tracheal, bronchus, and lung cancer	Rate	2010	941.130385	1039.99236	853.567051
DALYs (Disability-Adjusted Life Years)	China	Both	All ages	Larynx cancer	Number	2000	377345.407	425418.686	327189.025

DALYs (Disability-Adjusted Life Years)	China	Both	All ages	Larynx cancer	Rate	2000	29.9697924	33.7879022	25.9862369
DALYs (Disability-Adjusted Life Years)	China	Both	Age-standardized	Larynx cancer	Rate	2000	32.919505	37.1785283	28.6005211
DALYs (Disability-Adjusted Life Years)	China	Both	All ages	Tracheal, bronchus, and lung cancer	Number	2000	10599669	11637661.4	9595958.31
DALYs (Disability-Adjusted Life Years)	China	Both	All ages	Tracheal, bronchus, and lung cancer	Rate	2000	841.854369	924.294531	762.136952
DALYs (Disability-Adjusted Life Years)	China	Both	Age-standardized	Tracheal, bronchus, and lung cancer	Rate	2000	927.229684	1018.07195	839.31551
DALYs (Disability-Adjusted Life Years)	China	Both	All ages	Larynx cancer	Number	1990	362494.034	430009.612	296635.445
DALYs (Disability-Adjusted	China	Both	All ages	Larynx cancer	Rate	1990	30.8123353	36.5512232	25.214293

Life Years)									
DALYs (Disability-Adjusted Life Years)	China	Both	Age-standardized	Larynx cancer	Rate	1990	40.3705658	47.758985	33.2105554
DALYs (Disability-Adjusted Life Years)	China	Both	All ages	Tracheal, bronchus, and lung cancer	Number	1990	7762417.27	8989928.78	6632811.46
DALYs (Disability-Adjusted Life Years)	China	Both	All ages	Tracheal, bronchus, and lung cancer	Rate	1990	659.8128	764.152438	563.79524
DALYs (Disability-Adjusted Life Years)	China	Both	Age-standardized	Tracheal, bronchus, and lung cancer	Rate	1990	863.540123	996.208938	741.448221
DALYs (Disability-Adjusted Life Years)	China	Both	All ages	Larynx cancer	Number	2019	476667.986	608249.876	375724.657
DALYs (Disability-Adjusted Life Years)	China	Both	All ages	Larynx cancer	Rate	2019	33.7017951	43.0050125	26.564812
DALYs (Disability-Adjusted Life Years)	China	Both	Age-standardized	Larynx cancer	Rate	2019	23.186872	29.4456018	18.3477017

Adjusted Life Years)									
DALYs (Disability-Adjusted Life Years)	China	Both	All ages	Tracheal, bronchus, and lung cancer	Number	2019	17804470.2	21222042.3	14673037.6
DALYs (Disability-Adjusted Life Years)	China	Both	All ages	Tracheal, bronchus, and lung cancer	Rate	2019	1258.82716	1500.45932	1037.42589
DALYs (Disability-Adjusted Life Years)	China	Both	Age-standardized	Tracheal, bronchus, and lung cancer	Rate	2019	875.550121	1038.77185	724.763792
DALYs (Disability-Adjusted Life Years)	China	Both	All ages	Larynx cancer	Number	2020	487003.435	606752.962	371201.94
DALYs (Disability-Adjusted Life Years)	China	Both	All ages	Larynx cancer	Rate	2020	34.3084647	42.7445909	26.1504699
DALYs (Disability-Adjusted Life Years)	China	Both	Age-standardized	Larynx cancer	Rate	2020	23.0304598	28.5732182	17.6281167
DALYs (Disabil	China	Both	All ages	Tracheal,	Number	2020	18398789.3	22237788.5	15001121.9

ity-Adjusted Life Years)				bronchus, and lung cancer					
DALYs (Disability-Adjusted Life Years)	China	Both	All ages	Tracheal, bronchus, and lung cancer	Rate	2020	1296.15968	1566.60986	1056.80047
DALYs (Disability-Adjusted Life Years)	China	Both	Age-standardized	Tracheal, bronchus, and lung cancer	Rate	2020	879.056164	1058.28825	718.993267
DALYs (Disability-Adjusted Life Years)	China	Both	All ages	Larynx cancer	Number	2021	494204.198	633853.568	380811.521
DALYs (Disability-Adjusted Life Years)	China	Both	All ages	Larynx cancer	Rate	2021	34.7359412	44.5514231	26.7659535
DALYs (Disability-Adjusted Life Years)	China	Both	Age-standardized	Larynx cancer	Rate	2021	22.7417784	28.984331	17.6136482
DALYs (Disability-Adjusted Life Years)	China	Both	All ages	Tracheal, bronchus, and lung cancer	Number	2021	18913669.9	23090380.2	15142377.6

DALYs (Disability-Adjusted Life Years)	China	Both	All ages	Tracheal, bronchus, and lung cancer	Rate	2021	1329.37787	1622.94471	1064.3065
DALYs (Disability-Adjusted Life Years)	China	Both	Age-standardized	Tracheal, bronchus, and lung cancer	Rate	2021	877.944306	1067.30331	703.810667
Prevalence	China	Both	All ages	Tracheal, bronchus, and lung cancer	Number	1990	301999.002	347798.026	257252.112
Prevalence	China	Both	All ages	Tracheal, bronchus, and lung cancer	Rate	1990	25.6702005	29.5631607	21.8666726
Prevalence	China	Both	All ages	Tracheal, bronchus, and lung cancer	Number	2000	456623.646	501050.289	414176.28
Prevalence	China	Both	All ages	Tracheal, bronchus, and lung cancer	Rate	2000	36.2662845	39.7947685	32.8949999
Prevalence	China	Both	All ages	Tracheal,	Number	2010	758682.642	838268.7	686594.465

				bronchus, and lung cancer					
Prevalence	China	Both	All ages	Tracheal, bronchus, and lung cancer	Rate	2010	56.747792	62.700654	51.355755
Prevalence	China	Both	All ages	Tracheal, bronchus, and lung cancer	Number	2019	1154739.8	1379737.61	950781.811
Prevalence	China	Both	All ages	Tracheal, bronchus, and lung cancer	Rate	2019	81.6434195	97.5514101	67.2230038
Prevalence	China	Both	All ages	Tracheal, bronchus, and lung cancer	Number	2020	1217188.8	1477100.75	995149.492
Prevalence	China	Both	All ages	Tracheal, bronchus, and lung cancer	Rate	2020	85.748633	104.058936	70.1063869
Prevalence	China	Both	All ages	Tracheal, bronchus,	Number	2021	1262275.26	1545340.84	1005551.32

				and lung cancer					
Prevalence	China	Both	All ages	Tracheal, bronchus, and lung cancer	Rate	2021	88.7210578	108.61678	70.6768009
Prevalence	China	Both	Age-standardized	Tracheal, bronchus, and lung cancer	Rate	1990	33.7403115	38.6482755	28.9035262
Prevalence	China	Both	Age-standardized	Tracheal, bronchus, and lung cancer	Rate	2000	40.131122	44.0712201	36.4071217
Prevalence	China	Both	Age-standardized	Tracheal, bronchus, and lung cancer	Rate	2010	49.8519041	55.0156247	45.1855477
Prevalence	China	Both	Age-standardized	Tracheal, bronchus, and lung cancer	Rate	2019	56.3340911	67.1345784	46.4316259
Prevalence	China	Both	Age-standardized	Tracheal, bronchus, and	Rate	2020	57.5902399	69.5355697	47.2980837

				lung cancer					
Prevalence	China	Both	Age-standardized	Tracheal, bronchus, and lung cancer	Rate	2021	57.9473507	70.7772826	46.2040464
Prevalence	China	Both	All ages	Larynx cancer	Number	1990	69308.8214	81339.8697	56887.4001
Prevalence	China	Both	All ages	Larynx cancer	Rate	1990	5.89131529	6.91396576	4.83548274
Prevalence	China	Both	All ages	Larynx cancer	Number	2000	86501.9175	97071.6584	75891.3408
Prevalence	China	Both	All ages	Larynx cancer	Rate	2000	6.87021615	7.70969354	6.02749546
Prevalence	China	Both	All ages	Larynx cancer	Number	2010	134323.72	151600.784	117518.712
Prevalence	China	Both	All ages	Larynx cancer	Rate	2010	10.0471186	11.339405	8.79014104
Prevalence	China	Both	All ages	Larynx cancer	Number	2019	201409.834	253078.418	159804.731
Prevalence	China	Both	All ages	Larynx cancer	Rate	2019	14.2402535	17.8933707	11.2986533
Prevalence	China	Both	All ages	Larynx cancer	Number	2020	210452.15	261617.108	162832.888
Prevalence	China	Both	All ages	Larynx cancer	Rate	2020	14.8259532	18.4304271	11.471267
Prevalence	China	Both	All ages	Larynx cancer	Number	2021	217849.054	273850.854	171468.965
Prevalence	China	Both	All ages	Larynx cancer	Rate	2021	15.311873	19.2480501	12.0519735
Prevalence	China	Both	Age-standardized	Larynx cancer	Rate	1990	7.8304542	9.12512128	6.46588509
Prevalence	China	Both	Age-standardized	Larynx cancer	Rate	2000	7.62631908	8.53892465	6.715699
Prevalence	China	Both	Age-standardized	Larynx cancer	Rate	2010	8.66856816	9.7427273	7.58635461

Prevalence	China	Both	Age-standardized	Larynx cancer	Rate	2019	9.66973899	12.1023113	7.71064102
Prevalence	China	Both	Age-standardized	Larynx cancer	Rate	2020	9.80763043	12.1235734	7.62270318
Prevalence	China	Both	Age-standardized	Larynx cancer	Rate	2021	9.86420579	12.347578	7.81116743
Incidence	China	Both	All ages	Tracheal, bronchus, and lung cancer	Number	1990	274751.96	315111.78	234740.749
Incidence	China	Both	All ages	Tracheal, bronchus, and lung cancer	Rate	1990	23.3541761	26.784799	19.9531854
Incidence	China	Both	All ages	Tracheal, bronchus, and lung cancer	Number	2000	403535.149	442308.497	366520.244
Incidence	China	Both	All ages	Tracheal, bronchus, and lung cancer	Rate	2000	32.0498525	35.1293366	29.1100287
Incidence	China	Both	All ages	Tracheal, bronchus, and lung cancer	Number	2010	623928.517	689278.264	564445.163

Incidence	China	Both	All ages	Tracheal, bronchus, and lung cancer	Rate	2010	46.66848	51.5564973	42.2192561
Incidence	China	Both	All ages	Tracheal, bronchus, and lung cancer	Number	2019	861825.346	1021112.96	711347.544
Incidence	China	Both	All ages	Tracheal, bronchus, and lung cancer	Rate	2019	60.9335263	72.195618	50.2943137
Incidence	China	Both	All ages	Tracheal, bronchus, and lung cancer	Number	2020	900159.273	1083374.33	738850.474
Incidence	China	Both	All ages	Tracheal, bronchus, and lung cancer	Rate	2020	63.4145068	76.3216588	52.0506091
Incidence	China	Both	All ages	Tracheal, bronchus, and lung cancer	Number	2021	934704.064	1136937.93	750040.138
Incidence	China	Both	All ages	Tracheal,	Rate	2021	65.6971866	79.9115207	52.717784

				bronchus, and lung cancer					
Incidence	China	Both	Age-standardized	Tracheal, bronchus, and lung cancer	Rate	1990	33.1144866	37.7913722	28.4652058
Incidence	China	Both	Age-standardized	Tracheal, bronchus, and lung cancer	Rate	2000	37.8372153	41.3728004	34.4362202
Incidence	China	Both	Age-standardized	Tracheal, bronchus, and lung cancer	Rate	2010	43.1757399	47.5599705	39.1851091
Incidence	China	Both	Age-standardized	Tracheal, bronchus, and lung cancer	Rate	2019	43.4055478	51.2384151	36.019693
Incidence	China	Both	Age-standardized	Tracheal, bronchus, and lung cancer	Rate	2020	43.8602758	52.5849371	36.1361642
Incidence	China	Both	Age-standardized	Tracheal, bronchus,	Rate	2021	44.0143823	53.3495748	35.4476606

				and lung cancer					
Incidence	China	Both	All ages	Larynx cancer	Number	1990	15434.152	18174.015	12624.1898
Incidence	China	Both	All ages	Larynx cancer	Rate	1990	1.3119175	1.54480844	1.07306805
Incidence	China	Both	All ages	Larynx cancer	Number	2000	18185.3669	20512.2419	15802.7988
Incidence	China	Both	All ages	Larynx cancer	Rate	2000	1.444331	1.62913771	1.255101
Incidence	China	Both	All ages	Larynx cancer	Number	2010	25618.8977	29010.1793	22241.655
Incidence	China	Both	All ages	Larynx cancer	Rate	2010	1.91623717	2.16989757	1.66362685
Incidence	China	Both	All ages	Larynx cancer	Number	2019	36279.1521	45973.352	28681.9059
Incidence	China	Both	All ages	Larynx cancer	Rate	2019	2.56504021	3.25044798	2.02789309
Incidence	China	Both	All ages	Larynx cancer	Number	2020	37725.3295	46915.4739	28974.2055
Incidence	China	Both	All ages	Larynx cancer	Rate	2020	2.65767763	3.30510581	2.04117761
Incidence	China	Both	All ages	Larynx cancer	Number	2021	38904.8554	49486.1788	30369.6708
Incidence	China	Both	All ages	Larynx cancer	Rate	2021	2.73449067	3.4782161	2.13458142
Incidence	China	Both	Age-standardized	Larynx cancer	Rate	1990	1.82043963	2.13261868	1.50331836
Incidence	China	Both	Age-standardized	Larynx cancer	Rate	2000	1.6637984	1.87382726	1.45031901
Incidence	China	Both	Age-standardized	Larynx cancer	Rate	2010	1.70593029	1.92032672	1.48434744
Incidence	China	Both	Age-standardized	Larynx cancer	Rate	2019	1.7753872	2.23638384	1.4107009
Incidence	China	Both	Age-standardized	Larynx cancer	Rate	2020	1.78904939	2.21285579	1.37813013

Incidence	China	Both	Age-standardized	Larynx cancer	Rate	2021	1.78914368	2.26300814	1.40474029

Appendix 2：Percentage change of respiratory cancer burden in China , 1990–2021

measure	location	sex	age	cause	metric	year_start	year_end	val	upper	lower
Deaths	China	Both	All ages	Larynx cancer	Number	1990	2021	0.53966917	1.01923113	0.14142693
Deaths	China	Both	All ages	Larynx cancer	Percent	1990	2021	0.11399986	0.3287821	-0.0787465
Deaths	China	Both	All ages	Larynx cancer	Rate	1990	2021	0.27314041	0.66968645	-0.056163
Deaths	China	Both	Age-standardized	Larynx cancer	Rate	1990	2021	-0.4101526	-0.2343825	-0.5562333
Deaths	China	Both	All ages	Tracheal, bronchus, and lung cancer	Number	1990	2021	1.92601553	2.77307061	1.2280968
Deaths	China	Both	All ages	Tracheal, bronchus, and lung cancer	Percent	1990	2021	1.11782099	1.42804411	0.78585124
Deaths	China	Both	All ages	Tracheal, bronchus, and lung cancer	Rate	1990	2021	1.41949939	2.11992261	0.84239584
Deaths	China	Both	Age-standardized	Tracheal, bronchus, and lung cancer	Rate	1990	2021	0.12172928	0.43734777	-0.138304
DALYs (Disability-Adjusted Life Years)	China	Both	All ages	Larynx cancer	Number	1990	2021	0.36334437	0.82791576	-0.0015449
DALYs (Disability-Adjusted Life Years)	China	Both	All ages	Larynx cancer	Percent	1990	2021	0.41236506	0.7480832	0.1007014
DALYs (Disability-Adjusted Life Years)	China	Both	All ages	Larynx cancer	Rate	1990	2021	0.12733881	0.51148927	-0.1743853
DALYs (Disability-Adjusted Life Years)	China	Both	Age-standardized	Larynx cancer	Rate	1990	2021	-0.4366743	-0.2519964	-0.5833182
DALYs (Disability-Adjusted Life Years)	China	Both	All ages	Tracheal, bronchus, and lung cancer	Number	1990	2021	1.4365696	2.19285305	0.82880409

DALYs (Disability-Adjusted Life Years)	China	Both	All ages	Tracheal, bronchus, and lung cancer	Percent	1990	2021	1.52511679	2.01563302	1.05033497
DALYs (Disability-Adjusted Life Years)	China	Both	All ages	Tracheal, bronchus, and lung cancer	Rate	1990	2021	1.01478036	1.64014524	0.51222382
DALYs (Disability-Adjusted Life Years)	China	Both	Age-standardized	Tracheal, bronchus, and lung cancer	Rate	1990	2021	0.01668039	0.32559679	-0.2335338
YLDs (Years Lived with Disability)	China	Both	All ages	Larynx cancer	Number	1990	2021	1.85219996	2.77017708	1.12412294
YLDs (Years Lived with Disability)	China	Both	All ages	Larynx cancer	Percent	1990	2021	0.83549226	1.41933238	0.36824087
YLDs (Years Lived with Disability)	China	Both	All ages	Larynx cancer	Rate	1990	2021	1.35846187	2.11752998	0.75642067
YLDs (Years Lived with Disability)	China	Both	Age-standardized	Larynx cancer	Percent	1990	2021	0.19071495	0.56016588	-0.103852
YLDs (Years Lived with Disability)	China	Both	Age-standardized	Larynx cancer	Rate	1990	2021	0.12670204	0.48220897	-0.1519525
YLDs (Years Lived with Disability)	China	Both	All ages	Tracheal, bronchus, and lung cancer	Number	1990	2021	2.48120886	3.56142667	1.59326038
YLDs (Years Lived with Disability)	China	Both	All ages	Tracheal, bronchus, and lung cancer	Percent	1990	2021	1.24241429	1.92662991	0.66015004
YLDs (Years Lived with Disability)	China	Both	All ages	Tracheal, bronchus, and lung cancer	Rate	1990	2021	1.87858441	2.77180808	1.14434675
YLDs (Years Lived with Disability)	China	Both	Age-standardized	Tracheal, bronchus, and lung cancer	Percent	1990	2021	0.46631972	0.89611875	0.10168445
YLDs (Years Lived with Disability)	China	Both	Age-standardized	Tracheal, bronchus, and lung cancer	Rate	1990	2021	0.38635969	0.8157067	0.03971342
YLLs (Years of Life Lost)	China	Both	All ages	Larynx cancer	Number	1990	2021	0.33123546	0.77522653	-0.0294919

YLLs (Years of Life Lost)	China	Both	All ages	Larynx cancer	Percent	1990	2021	0.70396331	1.05272055	0.38784545
YLLs (Years of Life Lost)	China	Both	All ages	Larynx cancer	Rate	1990	2021	0.1007882	0.46792095	-0.1974944
YLLs (Years of Life Lost)	China	Both	Age-standardized	Larynx cancer	Rate	1990	2021	-0.449278	-0.2701156	-0.5944094
YLLs (Years of Life Lost)	China	Both	All ages	Tracheal, bronchus, and lung cancer	Number	1990	2021	1.42796711	2.17356805	0.82815112
YLLs (Years of Life Lost)	China	Both	All ages	Tracheal, bronchus, and lung cancer	Percent	1990	2021	2.10971754	2.62169701	1.58834544
YLLs (Years of Life Lost)	China	Both	All ages	Tracheal, bronchus, and lung cancer	Rate	1990	2021	1.00766704	1.62419863	0.51168388
YLLs (Years of Life Lost)	China	Both	Age-standardized	Tracheal, bronchus, and lung cancer	Rate	1990	2021	0.01347654	0.31658728	-0.2333112
Prevalence	China	Both	All ages	Larynx cancer	Number	1990	2021	2.14316489	3.13249585	1.35297258
Prevalence	China	Both	All ages	Larynx cancer	Percent	1990	2021	1.63780036	2.47112031	0.97336675
Prevalence	China	Both	All ages	Larynx cancer	Rate	1990	2021	1.5990585	2.41712854	0.94565464
Prevalence	China	Both	Age-standardized	Larynx cancer	Percent	1990	2021	0.31155402	0.71386835	-0.0127564
Prevalence	China	Both	Age-standardized	Larynx cancer	Rate	1990	2021	0.25972332	0.64464728	-0.0499373
Prevalence	China	Both	All ages	Tracheal, bronchus, and lung cancer	Number	1990	2021	3.17973321	4.49180835	2.1566487
Prevalence	China	Both	All ages	Tracheal, bronchus, and lung cancer	Percent	1990	2021	2.50765291	3.59387766	1.64166163

Prevalence	China	Both	All ages	Tracheal, bronchus, and lung cancer	Rate	1990	2021	2.45618874	3.54113342	1.61020815
Prevalence	China	Both	Age-standardized	Tracheal, bronchus, and lung cancer	Percent	1990	2021	0.78808411	1.32268322	0.35045067
Prevalence	China	Both	Age-standardized	Tracheal, bronchus, and lung cancer	Rate	1990	2021	0.71745156	1.24080478	0.30218119
Incidence	China	Both	All ages	Larynx cancer	Number	1990	2021	1.52069926	2.35450997	0.86828738
Incidence	China	Both	All ages	Larynx cancer	Percent	1990	2021	1.3124217	2.08728923	0.70884166
Incidence	China	Both	All ages	Larynx cancer	Rate	1990	2021	1.08434652	1.77381809	0.54487224
Incidence	China	Both	Age-standardized	Larynx cancer	Percent	1990	2021	0.06362141	0.400753	-0.2046448
Incidence	China	Both	Age-standardized	Larynx cancer	Rate	1990	2021	-0.0171914	0.29694891	-0.2615572
Incidence	China	Both	All ages	Tracheal, bronchus, and lung cancer	Number	1990	2021	2.40199235	3.42290813	1.56052045
Incidence	China	Both	All ages	Tracheal, bronchus, and lung cancer	Percent	1990	2021	2.12013923	3.06359784	1.34383564
Incidence	China	Both	All ages	Tracheal, bronchus, and lung cancer	Rate	1990	2021	1.8130809	2.65726818	1.11727435
Incidence	China	Both	Age-standardized	Tracheal, bronchus, and lung cancer	Percent	1990	2021	0.43802449	0.85830028	0.09724478
Incidence	China	Both	Age-standardized	Tracheal, bronchus, and lung cancer	Rate	1990	2021	0.32915792	0.71309171	0.01205423

Appendix 3：Number of Tracheal, bronchus, and lung cancer incidence cases by province in 2021

measure	location	sex	age	cause	metric	year	val	upper	lower
Incidence	Shandong	Both	All ages	Tracheal, bronchus, and lung cancer	Number	2021	77225.172	101352.201	58842.2726
Incidence	Sichuan	Both	All ages	Tracheal, bronchus, and lung cancer	Number	2021	72290.5901	95562.1297	52684.2693
Incidence	Jiangsu	Both	All ages	Tracheal, bronchus, and lung cancer	Number	2021	65468.4931	86308.2021	48173.7271
Incidence	Guangdong	Both	All ages	Tracheal, bronchus, and lung cancer	Number	2021	59255.7511	76684.3368	44211.3839
Incidence	Henan	Both	All ages	Tracheal, bronchus, and lung cancer	Number	2021	54030.239	69965.563	41147.5312
Incidence	Zhejiang	Both	All ages	Tracheal, bronchus, and lung cancer	Number	2021	50045.0205	67277.1619	36627.9507
Incidence	Hubei	Both	All ages	Tracheal, bronchus, and lung cancer	Number	2021	49836.7979	65312.9295	35924.9877
Incidence	Liaoning	Both	All ages	Tracheal, bronchus, and lung cancer	Number	2021	49364.6737	63992.9521	38762.0206
Incidence	Hunan	Both	All ages	Tracheal, bronchus, and lung cancer	Number	2021	43692.1701	56443.6359	33149.3204
Incidence	Hebei	Both	All ages	Tracheal, bronchus, and lung cancer	Number	2021	41429.7201	54482.6102	29313.508

Incidence	Heilongjiang	Both	All ages	Tracheal, bronchus, and lung cancer	Number	2021	40008.3441	51333.2102	30695.1816
Incidence	Anhui	Both	All ages	Tracheal, bronchus, and lung cancer	Number	2021	39173.8608	51540.5229	29010.1751
Incidence	Chongqing	Both	All ages	Tracheal, bronchus, and lung cancer	Number	2021	33549.4477	45395.1749	24911.191
Incidence	Jiangxi	Both	All ages	Tracheal, bronchus, and lung cancer	Number	2021	24742.7494	31663.3364	18379.7968
Incidence	Guangxi	Both	All ages	Tracheal, bronchus, and lung cancer	Number	2021	23407.6875	30455.7962	17066.3994
Incidence	Jilin	Both	All ages	Tracheal, bronchus, and lung cancer	Number	2021	21747.4621	28161.8271	16516.0616
Incidence	Fujian	Both	All ages	Tracheal, bronchus, and lung cancer	Number	2021	20713.7924	27240.5693	15781.3802
Incidence	Shanxi	Both	All ages	Tracheal, bronchus, and lung cancer	Number	2021	20402.9916	27701.7873	15130.803
Incidence	Yunnan	Both	All ages	Tracheal, bronchus, and lung cancer	Number	2021	19227.7635	26015.6672	13329.0304
Incidence	Shanghai	Both	All ages	Tracheal, bronchus, and lung cancer	Number	2021	18519.7512	24832.4083	13629.217
Incidence	Shaanxi	Both	All ages	Tracheal, bronchus, and lung cancer	Number	2021	18407.7426	25028.2512	13288.8253

Incidence	Guizhou	Both	All ages	Tracheal, bronchus, and lung cancer	Number	2021	17595.8863	24126.2262	12057.8305
Incidence	Inner Mongolia	Both	All ages	Tracheal, bronchus, and lung cancer	Number	2021	17491.879	22855.7345	13350.9596
Incidence	Beijing	Both	All ages	Tracheal, bronchus, and lung cancer	Number	2021	14671.5679	18894.2539	11122.1164
Incidence	Tianjin	Both	All ages	Tracheal, bronchus, and lung cancer	Number	2021	13906.4498	17803.5728	10609.101
Incidence	Taiwan (Province of China)	Both	All ages	Tracheal, bronchus, and lung cancer	Number	2021	12202.6741	13235.0077	10969.5874
Incidence	Gansu	Both	All ages	Tracheal, bronchus, and lung cancer	Number	2021	7397.8774	9612.9313	5593.5574
Incidence	Xinjiang	Both	All ages	Tracheal, bronchus, and lung cancer	Number	2021	6154.5643	8421.4721	4472.3137
Incidence	Hong Kong Special Administrative Region of China	Both	All ages	Tracheal, bronchus, and lung cancer	Number	2021	5255.0419	6811.5286	3930.4469
Incidence	Hainan	Both	All ages	Tracheal, bronchus, and lung cancer	Number	2021	4264.9644	5657.7423	3065.6595
Incidence	Ningxia	Both	All ages	Tracheal, bronchus, and lung cancer	Number	2021	2862.8751	3898.3810	2027.1089
Incidence	Qinghai	Both	All ages	Tracheal, bronchus, and lung cancer	Number	2021	1954.3219	2606.8694	1415.1438

Incidence	Tibet	Both	All ages	Tracheal, bronchus, and lung cancer	Number	2021	307.636309	425.908104	216.472645
Incidence	Macao Special Administrative Region of China	Both	All ages	Tracheal, bronchus, and lung cancer	Number	2021	300.778616	392.83471	224.374082

Appendix 4：Number of Larynx cancer incidence cases by province in 2021

measure	location	sex	age	cause	metric	year	val	upper	lower
Incidence	Guangdong	Both	All ages	Larynx cancer	Number	2021	3466.34103	4934.28532	2229.63996
Incidence	Sichuan	Both	All ages	Larynx cancer	Number	2021	2600.53643	3682.45827	1852.83989
Incidence	Liaoning	Both	All ages	Larynx cancer	Number	2021	2574.069	3598.45676	1737.17098
Incidence	Shandong	Both	All ages	Larynx cancer	Number	2021	2454.90967	3491.80153	1688.77389
Incidence	Hunan	Both	All ages	Larynx cancer	Number	2021	2392.88708	3347.55462	1572.74931
Incidence	Hubei	Both	All ages	Larynx cancer	Number	2021	2112.23153	2991.22698	1496.36457
Incidence	Heilongjiang	Both	All ages	Larynx cancer	Number	2021	1876.70711	2622.9717	1146.98431
Incidence	Zhejiang	Both	All ages	Larynx cancer	Number	2021	1834.37387	2608.24462	1254.54595
Incidence	Hebei	Both	All ages	Larynx cancer	Number	2021	1829.54367	2508.76725	1309.20686
Incidence	Henan	Both	All ages	Larynx cancer	Number	2021	1729.85597	2405.6787	1225.2847
Incidence	Jiangsu	Both	All ages	Larynx cancer	Number	2021	1654.16148	2370.28841	1105.97133
Incidence	Anhui	Both	All ages	Larynx cancer	Number	2021	1592.60753	2300.33285	1127.24611
Incidence	Guangxi	Both	All ages	Larynx cancer	Number	2021	1491.81749	2053.50187	977.389684
Incidence	Guizhou	Both	All ages	Larynx cancer	Number	2021	1210.32021	1741.55605	769.816595
Incidence	Jiangxi	Both	All ages	Larynx cancer	Number	2021	1115.36334	1517.00246	771.232134
Incidence	Shanxi	Both	All ages	Larynx cancer	Number	2021	1016.49857	1489.94837	698.698979
Incidence	Chongqing	Both	All ages	Larynx cancer	Number	2021	979.785218	1441.58369	642.895376
Incidence	Jilin	Both	All ages	Larynx cancer	Number	2021	982.290746	1361.82275	611.388812
Incidence	Yunnan	Both	All ages	Larynx cancer	Number	2021	912.571037	1252.02642	639.06242
Incidence	Fujian	Both	All ages	Larynx cancer	Number	2021	801.146377	1166.31247	535.240221

**Incidence**	**Inner Mongolia**	**Both**	**All ages**	**Larynx cancer**	**Number**	**2021**	**736.499832**	**1048.67883**	**520.205573**
**Incidence**	**Shaanxi**	**Both**	**All ages**	**Larynx cancer**	**Number**	**2021**	**734.410943**	**1047.76374**	**505.166459**
**Incidence**	**Shanghai**	**Both**	**All ages**	**Larynx cancer**	**Number**	**2021**	**587.538186**	**903.175681**	**377.354466**
**Incidence**	**Taiwan (Province of China)**	**Both**	**All ages**	**Larynx cancer**	**Number**	**2021**	**715.855335**	**799.919422**	**626.562026**
**Incidence**	**Beijing**	**Both**	**All ages**	**Larynx cancer**	**Number**	**2021**	**476.477851**	**706.07144**	**316.961**
**Incidence**	**Tianjin**	**Both**	**All ages**	**Larynx cancer**	**Number**	**2021**	**478.671397**	**669.637422**	**330.65962**
**Incidence**	**Hainan**	**Both**	**All ages**	**Larynx cancer**	**Number**	**2021**	**441.598885**	**658.665174**	**267.790271**
**Incidence**	**Gansu**	**Both**	**All ages**	**Larynx cancer**	**Number**	**2021**	**266.138906**	**385.267718**	**186.563865**
**Incidence**	**Xinjiang**	**Both**	**All ages**	**Larynx cancer**	**Number**	**2021**	**202.350517**	**294.459214**	**128.952705**
**Incidence**	**Hong Kong Special Administrative Region of China**	**Both**	**All ages**	**Larynx cancer**	**Number**	**2021**	**192.076796**	**266.732364**	**134.008937**
**Incidence**	**Ningxia**	**Both**	**All ages**	**Larynx cancer**	**Number**	**2021**	**75.5779217**	**106.932187**	**52.2256836**
**Incidence**	**Qinghai**	**Both**	**All ages**	**Larynx cancer**	**Number**	**2021**	**52.7109932**	**79.2604643**	**36.7057199**
**Incidence**	**Macao Special Administrative Region of China**	**Both**	**All ages**	**Larynx cancer**	**Number**	**2021**	**18.3227862**	**28.5048249**	**12.1076502**
**Incidence**	**Tibet**	**Both**	**All ages**	**Larynx cancer**	**Number**	**2021**	**14.4630067**	**22.7724416**	**9.15886327**

Appendix 5：Number of Tracheal, bronchus, and lung cancer mortality cases by province in 2021

measure	location	sex	age	cause	metric	year	val	upper	lower
Deaths	Shandong	Both	All ages	Tracheal, bronchus, and lung cancer	Number	2021	71354.1015	93289.279	53958.5712
Deaths	Sichuan	Both	All ages	Tracheal, bronchus, and lung cancer	Number	2021	62948.5163	83345.3581	46501.1583
Deaths	Jiangsu	Both	All ages	Tracheal, bronchus, and lung cancer	Number	2021	53035.7731	69743.2192	38751.7299
Deaths	Henan	Both	All ages	Tracheal, bronchus, and lung cancer	Number	2021	49873.3487	64205.1125	37186.9917
Deaths	Guangdong	Both	All ages	Tracheal, bronchus, and lung cancer	Number	2021	44955.5332	58611.949	34058.9479
Deaths	Liaoning	Both	All ages	Tracheal, bronchus, and lung cancer	Number	2021	44619.6438	57525.7296	34991.1317
Deaths	Zhejiang	Both	All ages	Tracheal, bronchus, and lung cancer	Number	2021	42844.3666	56520.4412	31204.7039
Deaths	Hubei	Both	All ages	Tracheal, bronchus, and lung cancer	Number	2021	38138.7377	50569.4663	28061.0543
Deaths	Hunan	Both	All ages	Tracheal, bronchus, and lung cancer	Number	2021	37617.9564	49276.9687	28590.5918
Deaths	Hebei	Both	All ages	Tracheal, bronchus, and lung cancer	Number	2021	37436.4272	50075.1395	26535.6247

Deaths	Anhui	Both	All ages	Tracheal, bronchus, and lung cancer	Number	2021	36571.3965	48315.0202	27488.3842
Deaths	Heilongjiang	Both	All ages	Tracheal, bronchus, and lung cancer	Number	2021	35737.6474	45223.7614	27765.4816
Deaths	Chongqing	Both	All ages	Tracheal, bronchus, and lung cancer	Number	2021	30585.9412	41003.1222	22362.2858
Deaths	Jiangxi	Both	All ages	Tracheal, bronchus, and lung cancer	Number	2021	22240.7929	29128.9406	16611.1563
Deaths	Guangxi	Both	All ages	Tracheal, bronchus, and lung cancer	Number	2021	20043.8315	26069.7722	14680.7494
Deaths	Shanxi	Both	All ages	Tracheal, bronchus, and lung cancer	Number	2021	18718.2378	25159.7963	13730.4191
Deaths	Jilin	Both	All ages	Tracheal, bronchus, and lung cancer	Number	2021	18059.9677	23354.4876	13756.2636
Deaths	Yunnan	Both	All ages	Tracheal, bronchus, and lung cancer	Number	2021	17684.0806	23589.4395	12561.6864
Deaths	Guizhou	Both	All ages	Tracheal, bronchus, and lung cancer	Number	2021	17449.2678	23695.8885	11933.5507
Deaths	Fujian	Both	All ages	Tracheal, bronchus, and lung cancer	Number	2021	16928.6029	22741.0093	12640.101
Deaths	Shaanxi	Both	All ages	Tracheal, bronchus, and lung cancer	Number	2021	16664.0971	22310.1039	11854.7321

Deaths	Shanghai	Both	All ages	Tracheal, bronchus, and lung cancer	Number	2021	15095.3286	19901.166	11207.2282
Deaths	Inner Mongolia	Both	All ages	Tracheal, bronchus, and lung cancer	Number	2021	14963.544	19384.1231	11415.5212
Deaths	Beijing	Both	All ages	Tracheal, bronchus, and lung cancer	Number	2021	12110.191	15538.0554	9182.73362
Deaths	Tianjin	Both	All ages	Tracheal, bronchus, and lung cancer	Number	2021	11649.3856	14914.236	8835.86138
Deaths	Taiwan (Province of China)	Both	All ages	Tracheal, bronchus, and lung cancer	Number	2021	11592.6359	12596.9765	10418.7351
Deaths	Gansu	Both	All ages	Tracheal, bronchus, and lung cancer	Number	2021	7027.4586	9137.7423	5326.0963
Deaths	Xinjiang	Both	All ages	Tracheal, bronchus, and lung cancer	Number	2021	6082.05312	8279.17097	4397.66264
Deaths	Hong Kong Special Administrative Region of China	Both	All ages	Tracheal, bronchus, and lung cancer	Number	2021	4783.90878	6116.33134	3523.35782
Deaths	Hainan	Both	All ages	Tracheal, bronchus, and lung cancer	Number	2021	3950.62148	5261.18761	2888.94608
Deaths	Ningxia	Both	All ages	Tracheal, bronchus, and lung cancer	Number	2021	2466.60682	3361.51441	1776.75444
Deaths	Qinghai	Both	All ages	Tracheal, bronchus, and lung cancer	Number	2021	1901.0571	2516.86289	1393.74256

Deaths	Tibet	Both	All ages	Tracheal, bronchus, and lung cancer	Number	2021	310.006209	424.509122	220.194955
Deaths	Macao Special Administrative Region of China	Both	All ages	Tracheal, bronchus, and lung cancer	Number	2021	272.171645	355.693541	199.333058

Appendix 6：Number of Larynx cancer mortality cases by province in 2021

measure	location	sex	age	cause	metric	year	val	upper	lower
Deaths	Hunan	Both	All ages	Larynx cancer	Number	2021	1500.7659	2151.4538	1003.92667
Deaths	Sichuan	Both	All ages	Larynx cancer	Number	2021	1378.92315	1951.2248	975.279746
Deaths	Guangdong	Both	All ages	Larynx cancer	Number	2021	1369.78699	1930.62794	916.887453
Deaths	Shandong	Both	All ages	Larynx cancer	Number	2021	1191.81041	1704.31664	830.493093
Deaths	Liaoning	Both	All ages	Larynx cancer	Number	2021	1094.2016	1510.11037	755.510244
Deaths	Hebei	Both	All ages	Larynx cancer	Number	2021	1035.7943	1421.12207	736.580575
Deaths	Heilongjiang	Both	All ages	Larynx cancer	Number	2021	1032.88436	1453.33891	667.012528
Deaths	Hubei	Both	All ages	Larynx cancer	Number	2021	993.479522	1378.44613	692.634605
Deaths	Guangxi	Both	All ages	Larynx cancer	Number	2021	911.943691	1239.86909	609.492295
Deaths	Henan	Both	All ages	Larynx cancer	Number	2021	877.271755	1209.76972	614.719677

Deaths	Guizhou	Both	All ages	Larynx cancer	Number	2021	841.800767	1211.73043	546.189992
Deaths	Jiangsu	Both	All ages	Larynx cancer	Number	2021	794.405791	1133.50443	536.6786
Deaths	Anhui	Both	All ages	Larynx cancer	Number	2021	741.618266	1038.51175	527.646801
Deaths	Zhejiang	Both	All ages	Larynx cancer	Number	2021	660.569766	944.103156	453.440591
Deaths	Jiangxi	Both	All ages	Larynx cancer	Number	2021	633.203507	875.773093	440.754842
Deaths	Yunnan	Both	All ages	Larynx cancer	Number	2021	630.010024	857.831527	439.959766
Deaths	Shanxi	Both	All ages	Larynx cancer	Number	2021	534.780597	764.369241	376.049197
Deaths	Jilin	Both	All ages	Larynx cancer	Number	2021	522.412099	724.661292	335.298098
Deaths	Chongqing	Both	All ages	Larynx cancer	Number	2021	448.320337	635.334177	302.4627
Deaths	Shaanxi	Both	All ages	Larynx cancer	Number	2021	414.817742	580.616931	289.939928
Deaths	Inner Mongolia	Both	All ages	Larynx cancer	Number	2021	382.959325	548.512109	270.337885

Deaths	Fujian	Both	All ages	Larynx cancer	Number	2021	358.463386	518.381247	238.998879
Deaths	Shanghai	Both	All ages	Larynx cancer	Number	2021	291.228318	438.077197	187.740806
Deaths	Hainan	Both	All ages	Larynx cancer	Number	2021	263.517677	388.908698	169.419061
Deaths	Taiwan (Province of China)	Both	All ages	Larynx cancer	Number	2021	250.340248	277.75591	223.447423
Deaths	Tianjin	Both	All ages	Larynx cancer	Number	2021	211.578974	299.916428	147.807135
Deaths	Beijing	Both	All ages	Larynx cancer	Number	2021	201.037395	295.790609	136.002362
Deaths	Gansu	Both	All ages	Larynx cancer	Number	2021	170.486449	236.86759	120.248134
Deaths	Xinjiang	Both	All ages	Larynx cancer	Number	2021	141.69764	206.723929	93.2618685
Deaths	Hong Kong Special Administrative Region of China	Both	All ages	Larynx cancer	Number	2021	85.3430273	116.991079	59.0738272
Deaths	Ningxia	Both	All ages	Larynx cancer	Number	2021	41.5559272	58.8949991	28.8878969
Deaths	Qinghai	Both	All ages	Larynx cancer	Number	2021	38.2419352	54.8392912	26.7827243

Deaths	Tibet	Both	All ages	Larynx cancer	Number	2021	11.756241 3	18.616998 3	7.3931559 9
Deaths	Macao Special Administrative Region of China	Both	All ages	Larynx cancer	Number	2021	7.3906682 6	11.965309 4	4.8727317 3

Appendix 7：ASIR of Tracheal, bronchus, and lung cancer by province in 2021

measure	location	sex	age	cause	metric	year	val	upper	lower
Incidence	Heilongjiang	Both	Age-standardized	Tracheal, bronchus, and lung cancer	Rate	2021	70.687857 8	90.067612 1	55.272638 7
Incidence	Tianjin	Both	Age-standardized	Tracheal, bronchus, and lung cancer	Rate	2021	62.990737 3	80.579003 2	48.185621 3
Incidence	Liaoning	Both	Age-standardized	Tracheal, bronchus, and lung cancer	Rate	2021	61.450181 7	79.230813 2	48.626879
Incidence	Chongqing	Both	Age-standardized	Tracheal, bronchus, and lung cancer	Rate	2021	60.029052 6	80.835212 7	44.650464 3
Incidence	Hubei	Both	Age-standardized	Tracheal, bronchus, and lung cancer	Rate	2021	52.512150 8	68.529869	38.017213 7
Incidence	Jilin	Both	Age-standardized	Tracheal, bronchus, and lung cancer	Rate	2021	52.366529 9	67.415046 7	40.3431
Incidence	Zhejiang	Both	Age-standardized	Tracheal, bronchus, and lung cancer	Rate	2021	51.107261 6	68.339707 8	37.564272 5
Incidence	Sichuan	Both	Age-standardized	Tracheal, bronchus, and lung cancer	Rate	2021	49.983814 1	65.856173 3	36.553312 6
Incidence	Inner Mongolia	Both	Age-standardized	Tracheal, bronchus, and lung cancer	Rate	2021	47.553501 1	61.558822 3	36.305962 1
Incidence	Shandong	Both	Age-standardized	Tracheal, bronchus, and lung cancer	Rate	2021	46.689489 3	60.853775 6	35.634272 6
Incidence	Beijing	Both	Age-standardized	Tracheal, bronchus, and lung cancer	Rate	2021	45.550896 6	58.616891 9	34.729655 8
Incidence	Guangdong	Both	Age-standardized	Tracheal, bronchus, and lung cancer	Rate	2021	44.509428 7	57.555682 7	33.334190 5
Incidence	Jiangsu	Both	Age-standardized	Tracheal, bronchus, and lung cancer	Rate	2021	44.455566 3	58.269657 7	32.660496

Incidence	Shanghai	Both	Age-standardized	Tracheal, bronchus, and lung cancer	Rate	2021	43.38 0732 8	57.64 7047	32.24 2388 1
Incidence	Hunan	Both	Age-standardized	Tracheal, bronchus, and lung cancer	Rate	2021	40.93 3590 8	52.91 1737	31.15 1140 6
Incidence	Anhui	Both	Age-standardized	Tracheal, bronchus, and lung cancer	Rate	2021	40.77 9098 2	53.80 0675 1	30.41 4421 7
Incidence	Jiangxi	Both	Age-standardized	Tracheal, bronchus, and lung cancer	Rate	2021	40.50 3129	51.62 8272 9	30.13 4098 5
Incidence	Shanxi	Both	Age-standardized	Tracheal, bronchus, and lung cancer	Rate	2021	40.03 0272 6	53.85 1199 4	29.84 2734 8
Incidence	Henan	Both	Age-standardized	Tracheal, bronchus, and lung cancer	Rate	2021	37.91 1315 3	48.31 9033 8	29.12 5232 6
Incidence	Fujian	Both	Age-standardized	Tracheal, bronchus, and lung cancer	Rate	2021	37.59 5469 5	49.10 5473 8	28.71 9658 1
Incidence	Hebei	Both	Age-standardized	Tracheal, bronchus, and lung cancer	Rate	2021	37.10 0517 7	48.75 3282 2	26.18 7092 5
Incidence	Guizhou	Both	Age-standardized	Tracheal, bronchus, and lung cancer	Rate	2021	36.01 6527 4	49.36 5558 1	24.68 1163 8
Incidence	Ningxia	Both	Age-standardized	Tracheal, bronchus, and lung cancer	Rate	2021	35.11 0948 5	47.24 6126 7	24.88 0470 2
Incidence	Hainan	Both	Age-standardized	Tracheal, bronchus, and lung cancer	Rate	2021	34.42 6027	45.53 2672 7	24.96 4907
Incidence	Guangxi	Both	Age-standardized	Tracheal, bronchus, and lung cancer	Rate	2021	34.31 4184 3	44.46 5912 6	25.15 5204 9
Incidence	Hong Kong Special Administrative Region of China	Both	Age-standardized	Tracheal, bronchus, and lung cancer	Rate	2021	33.51 6267 9	43.34 8511 6	25.26 7287 3
Incidence	Yunnan	Both	Age-standardized	Tracheal, bronchus, and lung cancer	Rate	2021	32.00 0919 7	43.22 5887 8	22.44 1756 9

Incidence	Shaanxi	Both	Age-standardized	Tracheal, bronchus, and lung cancer	Rate	2021	31.277511 7	42.032573 7	22.650551 1
Incidence	Macao Special Administrative Region of China	Both	Age-standardized	Tracheal, bronchus, and lung cancer	Rate	2021	31.152838 6	40.489386 7	23.280127 9
Incidence	Qinghai	Both	Age-standardized	Tracheal, bronchus, and lung cancer	Rate	2021	30.07769	39.818705 7	21.781333 6
Incidence	Taiwan (Province of China)	Both	Age-standardized	Tracheal, bronchus, and lung cancer	Rate	2021	28.653809 3	30.994966 2	25.780297 2
Incidence	Xinjiang	Both	Age-standardized	Tracheal, bronchus, and lung cancer	Rate	2021	22.946290 8	31.129642 4	16.803673 4
Incidence	Gansu	Both	Age-standardized	Tracheal, bronchus, and lung cancer	Rate	2021	20.834758 3	27.045179 7	15.855428 3
Incidence	Tibet	Both	Age-standardized	Tracheal, bronchus, and lung cancer	Rate	2021	9.8020859 9	13.536550 3	6.9544686 2

Appendix 8：ASIR of Larynx cancer by province in 2021

measure	location	sex	age	cause	metric	year	val	upper	lower
Incidence	Hainan	Both	Age-standardized	Larynx cancer	Rate	2021	3.46087821	5.10686464	2.10093753
Incidence	Heilongjiang	Both	Age-standardized	Larynx cancer	Rate	2021	3.1279053	4.33435868	1.93651671
Incidence	Liaoning	Both	Age-standardized	Larynx cancer	Rate	2021	3.075019	4.27543157	2.08937828
Incidence	Guangdong	Both	Age-standardized	Larynx cancer	Rate	2021	2.48226319	3.49578453	1.61376749
Incidence	Guizhou	Both	Age-standardized	Larynx cancer	Rate	2021	2.43460534	3.51683098	1.55182642
Incidence	Jilin	Both	Age-standardized	Larynx cancer	Rate	2021	2.25528116	3.11214669	1.41572343
Incidence	Hunan	Both	Age-standardized	Larynx cancer	Rate	2021	2.22741785	3.10455805	1.47070788
Incidence	Hubei	Both	Age-standardized	Larynx cancer	Rate	2021	2.18554197	3.06337684	1.56049881
Incidence	Guangxi	Both	Age-standardized	Larynx cancer	Rate	2021	2.16863233	2.97973438	1.42409622
Incidence	Tianjin	Both	Age-standardized	Larynx cancer	Rate	2021	2.09681288	2.89864844	1.46337942
Incidence	Shanxi	Both	Age-standardized	Larynx cancer	Rate	2021	1.89957715	2.74865387	1.31600356
Incidence	Inner Mongolia	Both	Age-standardized	Larynx cancer	Rate	2021	1.87682757	2.6168148	1.33292426
Incidence	Zhejiang	Both	Age-standardized	Larynx cancer	Rate	2021	1.81685236	2.57558447	1.25575023

Incidence	Macao Special Administrative Region of China	Both	Age-standardized	Larynx cancer	Rate	2021	1.8097869	2.78444782	1.21780702
Incidence	Sichuan	Both	Age-standardized	Larynx cancer	Rate	2021	1.78636399	2.53763254	1.27379056
Incidence	Jiangxi	Both	Age-standardized	Larynx cancer	Rate	2021	1.78040564	2.40981631	1.23327498
Incidence	Chongqing	Both	Age-standardized	Larynx cancer	Rate	2021	1.73608215	2.55434611	1.14580312
Incidence	Taiwan (Province of China)	Both	Age-standardized	Larynx cancer	Rate	2021	1.6993651	1.89355613	1.48142611
Incidence	Anhui	Both	Age-standardized	Larynx cancer	Rate	2021	1.64643899	2.39802129	1.1810519
Incidence	Hebei	Both	Age-standardized	Larynx cancer	Rate	2021	1.58450834	2.1548497	1.13806676
Incidence	Yunnan	Both	Age-standardized	Larynx cancer	Rate	2021	1.47957457	2.01922162	1.04636954
Incidence	Shandong	Both	Age-standardized	Larynx cancer	Rate	2021	1.45090625	2.06019846	1.01301421
Incidence	Beijing	Both	Age-standardized	Larynx cancer	Rate	2021	1.41611835	2.08791484	0.95253254
Incidence	Fujian	Both	Age-standardized	Larynx cancer	Rate	2021	1.41119885	2.04589625	0.94892335
Incidence	Shanghai	Both	Age-standardized	Larynx cancer	Rate	2021	1.3591579	2.0712149	0.88533946
Incidence	Hong Kong Special Administrative Region of China	Both	Age-standardized	Larynx cancer	Rate	2021	1.23694458	1.70425931	0.86896075
Incidence	Shaanxi	Both	Age-standardized	Larynx cancer	Rate	2021	1.21282423	1.71888133	0.84074084

Incidence	Henan	Both	Age-standardized	Larynx cancer	Rate	2021	1.18247469	1.64100557	0.84674938
Incidence	Jiangsu	Both	Age-standardized	Larynx cancer	Rate	2021	1.12450825	1.59319246	0.76353473
Incidence	Ningxia	Both	Age-standardized	Larynx cancer	Rate	2021	0.87833393	1.22364693	0.61640824
Incidence	Qinghai	Both	Age-standardized	Larynx cancer	Rate	2021	0.77467398	1.15844472	0.54824314
Incidence	Gansu	Both	Age-standardized	Larynx cancer	Rate	2021	0.72861411	1.04861947	0.51578104
Incidence	Xinjiang	Both	Age-standardized	Larynx cancer	Rate	2021	0.7155964	1.03061971	0.46409508
Incidence	Tibet	Both	Age-standardized	Larynx cancer	Rate	2021	0.4418052	0.70268724	0.27991874

Appendix 9：ASMR of Tracheal, bronchus, and lung cancer by province in 2021

measure	location	sex	age	cause	metric	year	val	upper	lower
Deaths	Heilongjiang	Both	Age-standardized	Tracheal, bronchus, and lung cancer	Rate	2021	64.884473 6	81.760051 7	50.665989 2
Deaths	Liaoning	Both	Age-standardized	Tracheal, bronchus, and lung cancer	Rate	2021	56.891270 7	72.772350 8	44.700481
Deaths	Chongqing	Both	Age-standardized	Tracheal, bronchus, and lung cancer	Rate	2021	55.234091 6	73.967732 6	40.285839
Deaths	Tianjin	Both	Age-standardized	Tracheal, bronchus, and lung cancer	Rate	2021	54.217559 7	68.941334 6	41.40323
Deaths	Jilin	Both	Age-standardized	Tracheal, bronchus, and lung cancer	Rate	2021	44.959559 6	57.763700 3	34.684518
Deaths	Zhejiang	Both	Age-standardized	Tracheal, bronchus, and lung cancer	Rate	2021	44.668233	58.346208 2	32.713512 7
Deaths	Sichuan	Both	Age-standardized	Tracheal, bronchus, and lung cancer	Rate	2021	43.735495 5	57.688051 4	32.367555 9
Deaths	Shandong	Both	Age-standardized	Tracheal, bronchus, and lung cancer	Rate	2021	43.686288 7	56.659768 6	33.304811 4
Deaths	Inner Mongolia	Both	Age-standardized	Tracheal, bronchus, and lung cancer	Rate	2021	41.839438 9	53.830328 4	32.031686 7
Deaths	Hubei	Both	Age-standardized	Tracheal, bronchus, and lung cancer	Rate	2021	41.131998 9	54.094672 6	30.089865 4
Deaths	Beijing	Both	Age-standardized	Tracheal, bronchus, and lung cancer	Rate	2021	38.455282 3	49.162524 7	29.174306 3
Deaths	Anhui	Both	Age-standardized	Tracheal, bronchus, and lung cancer	Rate	2021	38.201239 6	50.425490 1	28.797868 1
Deaths	Shanxi	Both	Age-standardized	Tracheal, bronchus, and lung cancer	Rate	2021	37.569926 6	49.805874 3	27.709678 2

Deaths	Jiangxi	Both	Age-standardized	Tracheal, bronchus, and lung cancer	Rate	2021	37.0179906	47.993164	27.7087567
Deaths	Jiangsu	Both	Age-standardized	Tracheal, bronchus, and lung cancer	Rate	2021	36.4850238	47.7643233	26.7441357
Deaths	Guizhou	Both	Age-standardized	Tracheal, bronchus, and lung cancer	Rate	2021	36.2901565	49.1850529	24.7087491
Deaths	Shanghai	Both	Age-standardized	Tracheal, bronchus, and lung cancer	Rate	2021	35.8093326	47.2557679	26.7028521
Deaths	Hunan	Both	Age-standardized	Tracheal, bronchus, and lung cancer	Rate	2021	35.5668906	46.4694227	27.0895778
Deaths	Henan	Both	Age-standardized	Tracheal, bronchus, and lung cancer	Rate	2021	35.4391411	45.3702679	26.662789
Deaths	Guangdong	Both	Age-standardized	Tracheal, bronchus, and lung cancer	Rate	2021	34.5352167	44.650678	26.143623
Deaths	Hebei	Both	Age-standardized	Tracheal, bronchus, and lung cancer	Rate	2021	34.2669226	45.352887	24.1738896
Deaths	Hainan	Both	Age-standardized	Tracheal, bronchus, and lung cancer	Rate	2021	32.245615	42.632149	23.6678178
Deaths	Fujian	Both	Age-standardized	Tracheal, bronchus, and lung cancer	Rate	2021	31.3054076	41.6274813	23.5237818
Deaths	Ningxia	Both	Age-standardized	Tracheal, bronchus, and lung cancer	Rate	2021	31.2914445	42.1659922	22.8288805
Deaths	Qinghai	Both	Age-standardized	Tracheal, bronchus, and lung cancer	Rate	2021	30.2918185	39.9158063	22.375814
Deaths	Yunnan	Both	Age-standardized	Tracheal, bronchus, and lung cancer	Rate	2021	30.020036	39.7263697	21.4632882
Deaths	Hong Kong Special Administrative Region of China	Both	Age-standardized	Tracheal, bronchus, and lung cancer	Rate	2021	29.8543274	38.2880141	22.2442097

Deaths	Guangxi	Both	Age-standardized	Tracheal, bronchus, and lung cancer	Rate	2021	29.576738 7	38.494703 8	21.693409
Deaths	Shaanxi	Both	Age-standardized	Tracheal, bronchus, and lung cancer	Rate	2021	28.926103 8	38.425937 9	20.898936
Deaths	Macao Special Administrative Region of China	Both	Age-standardized	Tracheal, bronchus, and lung cancer	Rate	2021	28.664203 1	37.438273 9	20.761149 1
Deaths	Taiwan (Province of China)	Both	Age-standardized	Tracheal, bronchus, and lung cancer	Rate	2021	27.020284 9	29.312704 9	24.367662 1
Deaths	Xinjiang	Both	Age-standardized	Tracheal, bronchus, and lung cancer	Rate	2021	23.392606 5	31.378662 5	17.116122 7
Deaths	Gansu	Both	Age-standardized	Tracheal, bronchus, and lung cancer	Rate	2021	20.262750 2	26.149324 2	15.398099 8
Deaths	Tibet	Both	Age-standardized	Tracheal, bronchus, and lung cancer	Rate	2021	10.192462 2	13.794465 2	7.2662129 5

Appendix 9：ASMR of Larynx cancer by province in 2021

measure	location	sex	age	cause	metric	year	val	upper	lower
Deaths	Hainan	Both	Age-standardized	Larynx cancer	Rate	2021	2.11442275	3.09388473	1.36575519
Deaths	Heilongjiang	Both	Age-standardized	Larynx cancer	Rate	2021	1.79994995	2.50339879	1.17290041
Deaths	Guizhou	Both	Age-standardized	Larynx cancer	Rate	2021	1.72711881	2.46361739	1.12719651
Deaths	Hunan	Both	Age-standardized	Larynx cancer	Rate	2021	1.41697816	2.01067025	0.9540835
Deaths	Liaoning	Both	Age-standardized	Larynx cancer	Rate	2021	1.36454377	1.88353057	0.94688237
Deaths	Guangxi	Both	Age-standardized	Larynx cancer	Rate	2021	1.33812867	1.80953636	0.90072943
Deaths	Jilin	Both	Age-standardized	Larynx cancer	Rate	2021	1.27463242	1.7556214	0.81993344
Deaths	Hubei	Both	Age-standardized	Larynx cancer	Rate	2021	1.07238882	1.47553943	0.75810568
Deaths	Yunnan	Both	Age-standardized	Larynx cancer	Rate	2021	1.05648123	1.42641757	0.74742978
Deaths	Shanxi	Both	Age-standardized	Larynx cancer	Rate	2021	1.04939363	1.44507788	0.74050065
Deaths	Jiangxi	Both	Age-standardized	Larynx cancer	Rate	2021	1.04710605	1.43158497	0.73078282
Deaths	Guangdong	Both	Age-standardized	Larynx cancer	Rate	2021	1.03984692	1.45488225	0.70255927
Deaths	Inner Mongolia	Both	Age-standardized	Larynx cancer	Rate	2021	1.03904199	1.45867403	0.74631218

Deaths	Tianjin	Both	Age-standardized	Larynx cancer	Rate	2021	0.97774975	1.37461503	0.68794126
Deaths	Sichuan	Both	Age-standardized	Larynx cancer	Rate	2021	0.95001904	1.33373314	0.67596705
Deaths	Hebei	Both	Age-standardized	Larynx cancer	Rate	2021	0.93218949	1.27110029	0.66942304
Deaths	Chongqing	Both	Age-standardized	Larynx cancer	Rate	2021	0.80026485	1.13391214	0.54414921
Deaths	Anhui	Both	Age-standardized	Larynx cancer	Rate	2021	0.77195377	1.07641703	0.55097469
Deaths	Macao Special Administrative Region of China	Both	Age-standardized	Larynx cancer	Rate	2021	0.76882726	1.21186102	0.51017502
Deaths	Shandong	Both	Age-standardized	Larynx cancer	Rate	2021	0.72184409	1.02467911	0.51281573
Deaths	Shaanxi	Both	Age-standardized	Larynx cancer	Rate	2021	0.71224159	0.98964213	0.50578067
Deaths	Shanghai	Both	Age-standardized	Larynx cancer	Rate	2021	0.69096237	1.02428432	0.45186941
Deaths	Zhejiang	Both	Age-standardized	Larynx cancer	Rate	2021	0.68555016	0.98428477	0.4735628
Deaths	Fujian	Both	Age-standardized	Larynx cancer	Rate	2021	0.66065042	0.95170924	0.44371963
Deaths	Beijing	Both	Age-standardized	Larynx cancer	Rate	2021	0.62209922	0.90673409	0.42260726
Deaths	Henan	Both	Age-standardized	Larynx cancer	Rate	2021	0.6133999	0.84287161	0.43186833
Deaths	Qinghai	Both	Age-standardized	Larynx cancer	Rate	2021	0.59244388	0.84911262	0.42183177

Deaths	Taiwan (Province of China)	Both	Age-standardized	Larynx cancer	Rate	2021	0.5893054	0.65443833	0.5259513
Deaths	Jiangsu	Both	Age-standardized	Larynx cancer	Rate	2021	0.54802985	0.77784311	0.37290997
Deaths	Hong Kong Special Administrative Region of China	Both	Age-standardized	Larynx cancer	Rate	2021	0.53053471	0.72671375	0.36893059
Deaths	Xinjiang	Both	Age-standardized	Larynx cancer	Rate	2021	0.52734625	0.75307677	0.35505554
Deaths	Ningxia	Both	Age-standardized	Larynx cancer	Rate	2021	0.51490685	0.72123235	0.36238814
Deaths	Gansu	Both	Age-standardized	Larynx cancer	Rate	2021	0.48432918	0.6704142	0.34702368
Deaths	Tibet	Both	Age-standardized	Larynx cancer	Rate	2021	0.37485558	0.59437331	0.23613538

Appendix 10：Tracheal, bronchus, and lung cancer：EAPC_both_Results for ASIR

cause	location	Sex	Num_1990	ASR_1990	Num_2021	ASR_2021	EAPC_CI
Tracheal, bronchus, and lung cancer	Sichuan	Both	23682.3 (18787.8-29539.7)	28.8 (23-35.7)	72290.6 (52684.3-95562.1)	50 (36.6-65.9)	1.78 (1.59-1.97)
Tracheal, bronchus, and lung cancer	Henan	Both	13494.5 (11315.4-16155.2)	22.1 (18.5-26.2)	54030.2 (41147.5-69965.6)	37.9 (29.1-48.3)	1.77 (1.37-2.18)
Tracheal, bronchus, and lung cancer	Guangxi	Both	6296.7 (4973.3-7842.7)	21.4 (16.9-26.5)	23407.7 (17066.4-30455.8)	34.3 (25.2-44.5)	1.57 (1.27-1.88)
Tracheal, bronchus, and lung cancer	Hong Kong Special Administrative Region of China	Both	2732.9 (2415.1-3093.5)	48.6 (42.9-54.8)	5255 (3930.4-6811.5)	33.5 (25.3-43.3)	-1.3 (-1.53--1.07)
Tracheal, bronchus, and lung cancer	Hubei	Both	13899.8 (11326.3-16737.5)	36.8 (30.2-43.7)	49836.8 (35925-65312.9)	52.5 (38-68.5)	1.29 (0.9-1.68)
Tracheal, bronchus, and lung cancer	Liaoning	Both	12774.2 (9991.6-16096.8)	45 (35.4-56.5)	49364.7 (38762-63993)	61.5 (48.6-79.2)	1.19 (0.48-1.9)
Tracheal, bronchus, and lung cancer	Hunan	Both	13104.9 (10893.4-15686.2)	29.4 (24.6-35)	43692.2 (33149.3-56443.6)	40.9 (31.2-52.9)	1.09 (0.85-1.33)
Tracheal, bronchus, and lung cancer	Hebei	Both	11374 (8848-14584)	25.6 (20.1-32.5)	41429.7 (29313.5-54482.6)	37.1 (26.2-48.8)	1.09 (0.6-1.58)
Tracheal, bronchus, and lung cancer	Shandong	Both	21604.4 (16805.6-27359.1)	33.4 (26.2-42.2)	77225.2 (58842.3-101352.2)	46.7 (35.6-60.9)	0.95 (0.57-1.33)
Tracheal, bronchus, and lung cancer	Jiangsu	Both	18676.5 (15042.3-22744.3)	33.5 (27.2-40.4)	65468.5 (48173.7-86308.2)	44.5 (32.7-58.3)	0.92 (0.57-1.26)
Tracheal, bronchus, and lung cancer	Fujian	Both	5799.6 (4483.3-7330.4)	28.6 (22.4-35.9)	20713.8 (15781.4-27240.6)	37.6 (28.7-49.1)	0.9 (0.59-1.21)
Tracheal, bronchus, and lung cancer	China	Both	274752 (234740.7-315111.8)	33.1 (28.5-37.8)	934704.1 (750040.1-1136937.9)	44 (35.4-53.3)	0.88 (0.63-1.14)
Tracheal, bronchus, and lung cancer	Shanxi	Both	6087.3 (4613-7808.6)	30.6 (23.6-38.6)	20403 (15130.8-27701.8)	40 (29.8-53.9)	0.87 (0.75-1)

Tracheal, bronchus, and lung cancer	Qinghai	Both	524.8 (410.5-681.7)	22.2 (17.5-28.2)	1954.3 (1415.1-2606.9)	30.1 (21.8-39.8)	0.84 (0.48-1.2)
Tracheal, bronchus, and lung cancer	Zhejiang	Both	13091.3 (10148-16324.1)	38 (29.9-47.3)	50045 (36628-67277.2)	51.1 (37.6-68.3)	0.83 (0.41-1.26)
Tracheal, bronchus, and lung cancer	Yunnan	Both	6126.8 (4695-7879.7)	24.9 (19.4-31.7)	19227.8 (13329-26015.7)	32 (22.4-43.2)	0.81 (0.63-0.99)
Tracheal, bronchus, and lung cancer	Tibet	Both	180.1 (135.8-229.4)	12 (9.1-15.1)	307.6 (216.5-425.9)	9.8 (7-13.5)	-0.79 (-1.09--0.5)
Tracheal, bronchus, and lung cancer	Macao Special Administrative Region of China	Both	116.4 (95.5-137.2)	42.5 (34.8-50.1)	300.8 (224.4-392.8)	31.2 (23.3-40.5)	-0.76 (-1.21--0.32)
Tracheal, bronchus, and lung cancer	Guizhou	Both	5853.3 (4514-7408.1)	28.3 (22-35.3)	17595.9 (12057.8-24126.2)	36 (24.7-49.4)	0.69 (0.43-0.95)
Tracheal, bronchus, and lung cancer	Ningxia	Both	635.2 (486.9-807.4)	27.6 (21.5-34.7)	2862.9 (2027.1-3898.4)	35.1 (24.9-47.2)	0.68 (0.47-0.9)
Tracheal, bronchus, and lung cancer	Tianjin	Both	3558.3 (2768.9-4372.8)	49.6 (38.8-60.6)	13906.4 (10609.1-17803.6)	63 (48.2-80.6)	0.68 (0.33-1.03)
Tracheal, bronchus, and lung cancer	Chongqing	Both	6284.3 (4809.9-8016.8)	51.2 (39.1-65.3)	33549.4 (24911.2-45395.2)	60 (44.7-80.8)	0.55 (0.45-0.65)
Tracheal, bronchus, and lung cancer	Guangdong	Both	16672.1 (12806-20992.3)	36 (27.9-44.7)	59255.8 (44211.4-76684.3)	44.5 (33.3-57.6)	0.55 (0.13-0.96)
Tracheal, bronchus, and lung cancer	Hainan	Both	1351.3 (1045.4-1769.5)	29.8 (23.3-38.6)	4265 (3065.7-5657.7)	34.4 (25-45.5)	0.46 (0.44-0.48)
Tracheal, bronchus, and lung cancer	Inner Mongolia	Both	4923.5 (3716.2-6238.9)	40 (30.4-50.3)	17491.9 (13351-22855.7)	47.6 (36.3-61.6)	0.44 (0.02-0.86)
Tracheal, bronchus, and lung cancer	Shaanxi	Both	5966.2 (4705.8-7478.9)	27.2 (21.7-33.7)	18407.7 (13288.8-25028.3)	31.3 (22.7-42)	0.43 (0.39-0.47)
Tracheal, bronchus, and lung cancer	Anhui	Both	14409.4 (11179.2-18214.1)	36 (28.2-44.9)	39173.9 (29010.2-51540.5)	40.8 (30.4-53.8)	0.39 (0.32-0.46)

**Tracheal, bronchus, and lung cancer**	**Gansu**	**Both**	**2409.2 (1891.5-3017.1)**	**18.3 (14.5-22.6)**	**7397.9 (5593.6-9612.9)**	**20.8 (15.9-27)**	**0.35 (0.18-0.53)**
**Tracheal, bronchus, and lung cancer**	**Jilin**	**Both**	**7275.1 (6002.3-8736.7)**	**47.6 (39.6-57)**	**21747.5 (16516.1-28161.8)**	**52.4 (40.3-67.4)**	**0.24 (0.1-0.38)**
**Tracheal, bronchus, and lung cancer**	**Shanghai**	**Both**	**6219.5 (4871.5-7944.8)**	**42.8 (33.9-54.1)**	**18519.8 (13629.2-24832.4)**	**43.4 (32.2-57.6)**	**-0.21 (-0.94-0.53)**
**Tracheal, bronchus, and lung cancer**	**Heilongjiang**	**Both**	**13715.1 (10975.2-16711.7)**	**68.2 (55.4-82.8)**	**40008.3 (30695.2-51333.2)**	**70.7 (55.3-90.1)**	**0.1 (-0.05-0.25)**
**Tracheal, bronchus, and lung cancer**	**Beijing**	**Both**	**3799.7 (2938.1-4736.2)**	**42.9 (33.6-53)**	**14671.6 (11122.1-18894.3)**	**45.6 (34.7-58.6)**	**0.04 (-0.36-0.43)**
**Tracheal, bronchus, and lung cancer**	**Taiwan (Province of China)**	**Both**	**4140.5 (3924.5-4340)**	**25.7 (24.3-26.9)**	**12202.7 (10969.6-13235)**	**28.7 (25.8-31)**	**0.03 (-0.98-1.06)**
**Tracheal, bronchus, and lung cancer**	**Jiangxi**	**Both**	**10099.5 (8088.8-12469.8)**	**41.1 (33.2-50.1)**	**24742.7 (18379.8-31663.3)**	**40.5 (30.1-51.6)**	**-0.03 (-0.18-0.13)**
**Tracheal, bronchus, and lung cancer**	**Xinjiang**	**Both**	**2013.7 (1589.2-2566.2)**	**23.1 (18.3-29.3)**	**6154.6 (4472.3-8421.5)**	**22.9 (16.8-31.1)**	**-0.03 (-0.09-0.04)**

Appendix 11：Tracheal, bronchus, and lung cancer：EAPC_both_Results for ASMR

cause	location	Sex	Num_1990	ASR_1990	Num_2021	ASR_2021	EAPC_CI
Tracheal, bronchus, and lung cancer	Hong Kong Special Administrative Region of China	Both	2756.6 (2436.3-3124)	49.8 (43.9-56.5)	4783.9 (3523.4-6116.3)	29.9 (22.2-38.3)	-1.74 (-1.91--1.57)
Tracheal, bronchus, and lung cancer	Henan	Both	13844.1 (11468.5-16655.8)	23.5 (19.5-28)	49873.3 (37187-64205.1)	35.4 (26.7-45.4)	1.31 (0.82-1.81)
Tracheal, bronchus, and lung cancer	Macao Special Administrative Region of China	Both	122 (101-143.5)	44.6 (36.8-52.4)	272.2 (199.3-355.7)	28.7 (20.8-37.4)	-1.18 (-1.63--0.73)
Tracheal, bronchus, and lung cancer	Sichuan	Both	23923.8 (18766.6-29826.3)	30.2 (23.9-37.4)	62948.5 (46501.2-83345.4)	43.7 (32.4-57.7)	1.13 (0.78-1.47)
Tracheal, bronchus, and lung cancer	Guangxi	Both	6400.7 (5002.2-8013)	22.2 (17.5-27.7)	20043.8 (14680.7-26069.8)	29.6 (21.7-38.5)	0.91 (0.53-1.29)
Tracheal, bronchus, and lung cancer	Shanghai	Both	5996.3 (4655.3-7552.7)	42.4 (33.3-53.4)	15095.3 (11207.2-19901.2)	35.8 (26.7-47.3)	-0.83 (-1.61--0.05)
Tracheal, bronchus, and lung cancer	Tibet	Both	183.7 (138.5-234)	12.5 (9.5-15.8)	310 (220.2-424.5)	10.2 (7.3-13.8)	-0.83 (-1.15--0.5)
Tracheal, bronchus, and lung cancer	Liaoning	Both	13091 (10203.2-16875.6)	48 (37.8-61.4)	44619.6 (34991.1-57525.7)	56.9 (44.7-72.8)	0.73 (0.02-1.44)
Tracheal, bronchus, and lung cancer	Qinghai	Both	529 (404.7-680.5)	23.6 (18.3-29.9)	1901.1 (1393.7-2516.9)	30.3 (22.4-39.9)	0.64 (0.19-1.1)
Tracheal, bronchus, and lung cancer	Beijing	Both	3701.4 (2902.7-4692.8)	43.6 (34.5-54.1)	12110.2 (9182.7-15538.1)	38.5 (29.2-49.2)	-0.62 (-1.1--0.13)

Tracheal, bronchus, and lung cancer	Hebei	Both	11632.8 (8971.5-14841.6)	27.2 (21.3-34.3)	37436.4 (26535.6-50075.1)	34.3 (24.2-45.4)	0.59 (0.02-1.17)
Tracheal, bronchus, and lung cancer	Jiangxi	Both	10370.8 (8374.3-12705.9)	43.5 (35.4-52.6)	22240.8 (16611.2-29128.9)	37 (27.7-48)	-0.56 (-0.75--0.37)
Tracheal, bronchus, and lung cancer	Guizhou	Both	6032 (4658.6-7652.9)	30.2 (23.6-37.7)	17449.3 (11933.6-23695.9)	36.3 (24.7-49.2)	0.49 (0.14-0.84)
Tracheal, bronchus, and lung cancer	Jilin	Both	7314.6 (6009.7-8849.1)	50.2 (41.8-60.4)	18060 (13756.3-23354.5)	45 (34.7-57.8)	-0.48 (-0.74--0.22)
Tracheal, bronchus, and lung cancer	Shandong	Both	22361.6 (17436.2-28596.9)	35.8 (28.3-45)	71354.1 (53958.6-93289.3)	43.7 (33.3-56.7)	0.46 (-0.03-0.95)
Tracheal, bronchus, and lung cancer	Guangdong	Both	16698.5 (12834.8-21222.2)	37 (28.7-46.2)	44955.5 (34058.9-58611.9)	34.5 (26.1-44.7)	-0.44 (-1.02-0.14)
Tracheal, bronchus, and lung cancer	Shanxi	Both	6193.6 (4681.3-8064.9)	32.4 (25.1-41.6)	18718.2 (13730.4-25159.8)	37.6 (27.7-49.8)	0.43 (0.23-0.64)
Tracheal, bronchus, and lung cancer	Hunan	Both	13292.8 (10987.8-15965.3)	30.9 (25.7-36.7)	37618 (28590.6-49277)	35.6 (27.1-46.5)	0.42 (0.1-0.74)
Tracheal, bronchus, and lung cancer	Hubei	Both	13685.9 (11249.2-16570.1)	37.6 (31.2-44.9)	38138.7 (28061.1-50569.5)	41.1 (30.1-54.1)	0.38 (0.02-0.75)
Tracheal, bronchus, and lung cancer	Yunnan	Both	6276.9 (4769.3-8121.8)	26.4 (20.4-33.5)	17684.1 (12561.7-23589.4)	30 (21.5-39.7)	0.37 (0.09-0.65)
Tracheal, bronchus, and lung cancer	Heilongjiang	Both	13647.7 (10942.4-16628.6)	71 (57.7-85.6)	35737.6 (27765.5-45223.8)	64.9 (50.7-81.8)	-0.33 (-0.47--0.19)

Tracheal, bronchus, and lung cancer	China	Both	278235.2 (238517.8-322012.8)	34.7 (30.1-40)	814120.6 (652231.4-994858.2)	39 (31.4-47.3)	0.29 (-0.05-0.62)
Tracheal, bronchus, and lung cancer	Inner Mongolia	Both	4951.3 (3733.3-6322.9)	42.4 (32.6-53.7)	14963.5 (11415.5-19384.1)	41.8 (32-53.8)	-0.22 (-0.76-0.32)
Tracheal, bronchus, and lung cancer	Xinjiang	Both	2068.8 (1642-2630.9)	24.6 (19.7-31.2)	6082.1 (4397.7-8279.2)	23.4 (17.1-31.4)	-0.19 (-0.34--0.05)
Tracheal, bronchus, and lung cancer	Zhejiang	Both	13374.9 (10369.5-16906.4)	40.1 (31.5-50.2)	42844.4 (31204.7-56520.4)	44.7 (32.7-58.3)	0.19 (-0.29-0.66)
Tracheal, bronchus, and lung cancer	Taiwan (Province of China)	Both	4112.3 (3906.5-4319.2)	26.2 (24.8-27.6)	11592.6 (10418.7-12597)	27 (24.4-29.3)	-0.18 (-1.02-0.67)
Tracheal, bronchus, and lung cancer	Tianjin	Both	3441.3 (2687.7-4279)	49.9 (39.2-61.4)	11649.4 (8835.9-14914.2)	54.2 (41.4-68.9)	0.16 (-0.22-0.54)
Tracheal, bronchus, and lung cancer	Fujian	Both	5831.9 (4471.7-7389.1)	29.7 (23-37.3)	16928.6 (12640.1-22741)	31.3 (23.5-41.6)	0.12 (-0.29-0.54)
Tracheal, bronchus, and lung cancer	Jiangsu	Both	18766.1 (15212.9-22690.6)	34.7 (28.4-41.8)	53035.8 (38751.7-69743.2)	36.5 (26.7-47.8)	0.12 (-0.22-0.46)
Tracheal, bronchus, and lung cancer	Shaanxi	Both	6086.7 (4748.9-7688)	28.9 (23.1-36)	16664.1 (11854.7-22310.1)	28.9 (20.9-38.4)	-0.07 (-0.26-0.12)
Tracheal, bronchus, and lung cancer	Anhui	Both	14748.8 (11523.2-18520.3)	38.4 (30.3-47.7)	36571.4 (27488.4-48315)	38.2 (28.8-50.4)	-0.07 (-0.2-0.05)
Tracheal, bronchus, and lung cancer	Chongqing	Both	6424.7 (4937.1-8154.4)	54.1 (41.9-67.7)	30585.9 (22362.3-41003.1)	55.2 (40.3-74)	0.06 (0-0.12)

Tracheal, bronchus, and lung cancer	Hainan	Both	1381.6 (1071.7-1793.5)	31.2 (24.4-40)	3950.6 (2888.9-5261.2)	32.2 (23.7-42.6)	0.05 (-0.1-0.2)
Tracheal, bronchus, and lung cancer	Ningxia	Both	645.3 (493.4-826.1)	29.5 (23.1-37.4)	2466.6 (1776.8-3361.5)	31.3 (22.8-42.2)	0.03 (-0.36-0.43)
Tracheal, bronchus, and lung cancer	Gansu	Both	2458.2 (1918.4-3078.6)	19.6 (15.5-24.3)	7027.5 (5326.1-9137.7)	20.3 (15.4-26.1)	0.01 (-0.19-0.2)

Appendix 12：Larynx cancer：EAPC_both_Results for ASIR

cause	location	Sex	Num_1990	ASR_1990	Num_2021	ASR_2021	EAPC_CI
Larynx cancer	Hong Kong Special Administrative Region of China	Both	156.2 (118.3-188.5)	2.7 (2-3.2)	192.1 (134-266.7)	1.2 (0.9-1.7)	-2.55 (-2.68--2.43)
Larynx cancer	Tibet	Both	13 (8.6-20.5)	0.9 (0.6-1.3)	14.5 (9.2-22.8)	0.4 (0.3-0.7)	-2.18 (-2.52--1.85)
Larynx cancer	Jilin	Both	529.6 (361.8-680.9)	3.4 (2.3-4.3)	982.3 (611.4-1361.8)	2.3 (1.4-3.1)	-1.27 (-1.66--0.88)
Larynx cancer	Shanghai	Both	277.7 (197.1-378.7)	1.9 (1.3-2.5)	587.5 (377.4-903.2)	1.4 (0.9-2.1)	-1.24 (-1.6--0.87)
Larynx cancer	Xinjiang	Both	91.3 (66.5-129.4)	1 (0.8-1.5)	202.4 (129-294.5)	0.7 (0.5-1)	-1.14 (-1.46--0.82)
Larynx cancer	Liaoning	Both	703.5 (468.7-1060.8)	2.4 (1.6-3.6)	2574.1 (1737.2-3598.5)	3.1 (2.1-4.3)	1.14 (0.5-1.78)
Larynx cancer	Taiwan (Province of China)	Both	351.8 (327-379.5)	2.1 (2-2.3)	715.9 (626.6-799.9)	1.7 (1.5-1.9)	-0.91 (-1.44--0.38)
Larynx cancer	Guizhou	Both	650.3 (432.2-879.3)	3.1 (2.1-4.2)	1210.3 (769.8-1741.6)	2.4 (1.6-3.5)	-0.84 (-1.01--0.67)
Larynx cancer	Jiangxi	Both	602.4 (446.8-770)	2.4 (1.8-3.1)	1115.4 (771.2-1517)	1.8 (1.2-2.4)	-0.82 (-1.5--0.14)
Larynx cancer	Gansu	Both	122.6 (92.1-160.9)	0.9 (0.7-1.2)	266.1 (186.6-385.3)	0.7 (0.5-1)	-0.73 (-1.07--0.38)

Larynx cancer	Heilongjiang	Both	830.7 (564.7-1063.8)	4 (2.7-5)	1876.7 (1147-2623)	3.1 (1.9-4.3)	-0.72 (-0.88--0.56)
Larynx cancer	Sichuan	Both	1260.1 (922.6-1636.8)	1.5 (1.1-1.9)	2600.5 (1852.8-3682.5)	1.8 (1.3-2.5)	0.69 (0.27-1.12)
Larynx cancer	Zhejiang	Both	539.1 (385-726.6)	1.5 (1.1-2)	1834.4 (1254.5-2608.2)	1.8 (1.3-2.6)	0.61 (0.44-0.77)
Larynx cancer	Shaanxi	Both	334.3 (254.2-443.1)	1.5 (1.2-2)	734.4 (505.2-1047.8)	1.2 (0.8-1.7)	-0.6 (-1.02--0.17)
Larynx cancer	Henan	Both	640.3 (511.5-800.5)	1 (0.8-1.3)	1729.9 (1225.3-2405.7)	1.2 (0.8-1.6)	0.51 (0.35-0.67)
Larynx cancer	Qinghai	Both	21.3 (15.5-29.7)	0.9 (0.7-1.2)	52.7 (36.7-79.3)	0.8 (0.5-1.2)	-0.46 (-0.57--0.35)
Larynx cancer	Ningxia	Both	24.1 (18-33.3)	1 (0.8-1.4)	75.6 (52.2-106.9)	0.9 (0.6-1.2)	-0.45 (-0.74--0.15)
Larynx cancer	Beijing	Both	147.1 (111-195.5)	1.6 (1.2-2.1)	476.5 (317-706.1)	1.4 (1-2.1)	-0.39 (-0.51--0.28)
Larynx cancer	Hainan	Both	180.2 (114.7-237.7)	3.9 (2.4-5.1)	441.6 (267.8-658.7)	3.5 (2.1-5.1)	-0.33 (-0.61--0.05)
Larynx cancer	Yunnan	Both	405.6 (298.9-536.8)	1.6 (1.2-2.2)	912.6 (639.1-1252)	1.5 (1-2)	-0.3 (-0.42--0.18)
Larynx cancer	Guangxi	Both	631 (440.5-810.8)	2.1 (1.5-2.7)	1491.8 (977.4-2053.5)	2.2 (1.4-3)	0.3 (-0.23-0.84)

Larynx cancer	Inner Mongolia	Both	260.8 (187.8-347.9)	2.1 (1.5-2.8)	736.5 (520.2-1048.7)	1.9 (1.3-2.6)	-0.29 (-0.42--0.16)
Larynx cancer	Fujian	Both	277.6 (203.6-382.1)	1.3 (1-1.9)	801.1 (535.2-1166.3)	1.4 (0.9-2)	0.26 (-0.05-0.57)
Larynx cancer	Macao Special Administrative Region of China	Both	5.7 (4.3-7.5)	2.1 (1.6-2.8)	18.3 (12.1-28.5)	1.8 (1.2-2.8)	-0.24 (-0.65-0.17)
Larynx cancer	Shanxi	Both	424.5 (303.9-578.2)	2.1 (1.5-2.8)	1016.5 (698.7-1489.9)	1.9 (1.3-2.7)	-0.24 (-0.58-0.09)
Larynx cancer	Shandong	Both	909.9 (668-1213.1)	1.4 (1-1.8)	2454.9 (1688.8-3491.8)	1.5 (1-2.1)	0.24 (-0.08-0.57)
Larynx cancer	Tianjin	Both	167.3 (124-218.9)	2.3 (1.7-2.9)	478.7 (330.7-669.6)	2.1 (1.5-2.9)	-0.22 (-0.33--0.1)
Larynx cancer	Chongqing	Both	210.6 (154.1-280.3)	1.7 (1.3-2.2)	979.8 (642.9-1441.6)	1.7 (1.1-2.6)	0.22 (-0.21-0.65)
Larynx cancer	Guangdong	Both	1114.6 (761-1512.5)	2.4 (1.6-3.2)	3466.3 (2229.6-4934.3)	2.5 (1.6-3.5)	0.19 (0.01-0.37)
Larynx cancer	Jiangsu	Both	630 (473.1-822.5)	1.1 (0.9-1.5)	1654.2 (1106-2370.3)	1.1 (0.8-1.6)	0.17 (-0.25-0.59)
Larynx cancer	Hunan	Both	964.2 (659.5-1219.6)	2.2 (1.5-2.7)	2392.9 (1572.7-3347.6)	2.2 (1.5-3.1)	0.17 (-0.05-0.38)
Larynx cancer	Hebei	Both	699.2 (494.5-940.4)	1.5 (1.1-2.1)	1829.5 (1309.2-2508.8)	1.6 (1.1-2.2)	0.11 (0.01-0.22)

**Larynx cancer**	**China**	**Both**	**15434.2 (12624.2-18174)**	**1.8 (1.5-2.1)**	**38904.9 (30369.7-49486.2)**	**1.8 (1.4-2.3)**	**0.04 (-0.22-0.3)**
**Larynx cancer**	**Hubei**	**Both**	**913.9 (684.1-1147)**	**2.4 (1.8-3)**	**2112.2 (1496.4-2991.2)**	**2.2 (1.6-3.1)**	**-0.03 (-0.59-0.54)**
**Larynx cancer**	**Anhui**	**Both**	**695.7 (518.2-965.8)**	**1.7 (1.3-2.3)**	**1592.6 (1127.2-2300.3)**	**1.6 (1.2-2.4)**	**0 (-0.45-0.45)**

Appendix 13：Larynx cancer：EAPC_both_Results for ASMR

cause	location	Sex	Num_1990	ASR_1990	Num_2021	ASR_2021	EAPC_CI
Larynx cancer	Hong Kong Special Administrative Region of China	Both	100.7 (78.1-121.7)	1.8 (1.4-2.1)	85.3 (59.1-117)	0.5 (0.4-0.7)	-3.84 (-4.12--3.57)
Larynx cancer	Jilin	Both	450.7 (328.2-573)	3.1 (2.3-3.9)	522.4 (335.3-724.7)	1.3 (0.8-1.8)	-2.85 (-3.22--2.49)
Larynx cancer	Tibet	Both	12.1 (8.1-18.2)	0.8 (0.6-1.2)	11.8 (7.4-18.6)	0.4 (0.2-0.6)	-2.6 (-2.91--2.29)
Larynx cancer	Shanghai	Both	200 (142.2-278.1)	1.4 (1-1.9)	291.2 (187.7-438.1)	0.7 (0.5-1)	-2.52 (-2.9--2.15)
Larynx cancer	Beijing	Both	111.2 (82.2-146.5)	1.3 (1-1.7)	201 (136-295.8)	0.6 (0.4-0.9)	-2.46 (-2.76--2.17)
Larynx cancer	Taiwan (Province of China)	Both	186.1 (174-199.2)	1.2 (1.1-1.3)	250.3 (223.4-277.8)	0.6 (0.5-0.7)	-2.42 (-2.97--1.87)
Larynx cancer	Jiangxi	Both	544.2 (404.9-681.7)	2.3 (1.7-2.8)	633.2 (440.8-875.8)	1 (0.7-1.4)	-2.42 (-2.84--1.99)
Larynx cancer	Shaanxi	Both	292.3 (222.1-385.8)	1.4 (1.1-1.8)	414.8 (289.9-580.6)	0.7 (0.5-1)	-2.15 (-2.4--1.9)
Larynx cancer	Guangdong	Both	889.7 (631.5-1177.8)	2 (1.4-2.6)	1369.8 (916.9-1930.6)	1 (0.7-1.5)	-2.13 (-2.41--1.86)
Larynx cancer	Heilongjiang	Both	676.7 (465.9-869.3)	3.4 (2.4-4.4)	1032.9 (667-1453.3)	1.8 (1.2-2.5)	-2.1 (-2.27--1.93)

Larynx cancer	Anhui	Both	572.1 (424-763.2)	1.5 (1.1-1.9)	741.6 (527.6-1038.5)	0.8 (0.6-1.1)	-2.08 (-2.42--1.75)
Larynx cancer	Xinjiang	Both	83.8 (61.8-117.4)	1 (0.8-1.4)	141.7 (93.3-206.7)	0.5 (0.4-0.8)	-2.04 (-2.2--1.88)
Larynx cancer	Ningxia	Both	20.9 (15.7-28.7)	1 (0.7-1.3)	41.6 (28.9-58.9)	0.5 (0.4-0.7)	-2.01 (-2.17--1.85)
Larynx cancer	Inner Mongolia	Both	216.8 (159.9-289.2)	1.9 (1.4-2.4)	383 (270.3-548.5)	1 (0.7-1.5)	-1.95 (-2.07--1.83)
Larynx cancer	Zhejiang	Both	410.1 (294.4-547.7)	1.2 (0.9-1.6)	660.6 (453.4-944.1)	0.7 (0.5-1)	-1.92 (-2.1--1.74)
Larynx cancer	Chongqing	Both	174.3 (128.8-229.9)	1.5 (1.1-1.9)	448.3 (302.5-635.3)	0.8 (0.5-1.1)	-1.91 (-2.15--1.66)
Larynx cancer	Gansu	Both	108.4 (81.4-139)	0.9 (0.7-1.1)	170.5 (120.2-236.9)	0.5 (0.3-0.7)	-1.91 (-2.08--1.74)
Larynx cancer	Guizhou	Both	603 (407.1-815)	3 (2.1-4)	841.8 (546.2-1211.7)	1.7 (1.1-2.5)	-1.88 (-2.08--1.69)
Larynx cancer	Macao Special Administrative Region of China	Both	4.1 (3.1-5.3)	1.5 (1.1-2)	7.4 (4.9-12)	0.8 (0.5-1.2)	-1.87 (-2.35--1.39)
Larynx cancer	Fujian	Both	230.2 (168.8-312.2)	1.2 (0.9-1.6)	358.5 (239-518.4)	0.7 (0.4-1)	-1.86 (-2.03--1.68)
Larynx cancer	Hubei	Both	719.3 (545.7-913)	2 (1.5-2.5)	993.5 (692.6-1378.4)	1.1 (0.8-1.5)	-1.78 (-2.2--1.35)

Larynx cancer	Jiangsu	Both	520.2 (399.2-677.3)	1 (0.7-1.3)	794.4 (536.7-1133.5)	0.5 (0.4-0.8)	-1.78 (-1.97--1.6)
Larynx cancer	Shanxi	Both	347.6 (250-463)	1.8 (1.3-2.4)	534.8 (376-764.4)	1 (0.7-1.4)	-1.77 (-2.11--1.42)
Larynx cancer	Tianjin	Both	113.6 (83.5-147.4)	1.7 (1.2-2.1)	211.6 (147.8-299.9)	1 (0.7-1.4)	-1.76 (-1.89--1.64)
Larynx cancer	Shandong	Both	759.5 (564.6-1012)	1.2 (0.9-1.6)	1191.8 (830.5-1704.3)	0.7 (0.5-1)	-1.69 (-1.95--1.42)
Larynx cancer	China	Both	12869 (10580.7-15234.5)	1.6 (1.3-1.9)	19814.1 (15420.2-25279.4)	0.9 (0.7-1.2)	-1.69 (-1.8--1.59)
Larynx cancer	Hainan	Both	154.3 (104.9-205.5)	3.5 (2.3-4.6)	263.5 (169.4-388.9)	2.1 (1.4-3.1)	-1.61 (-1.76--1.46)
Larynx cancer	Yunnan	Both	374.3 (272.4-496.7)	1.6 (1.2-2.1)	630 (440-857.8)	1.1 (0.7-1.4)	-1.32 (-1.41--1.22)
Larynx cancer	Henan	Both	542 (430.7-681.4)	0.9 (0.7-1.1)	877.3 (614.7-1209.8)	0.6 (0.4-0.8)	-1.31 (-1.43--1.2)
Larynx cancer	Qinghai	Both	19.1 (14-26.5)	0.8 (0.6-1.2)	38.2 (26.8-54.8)	0.6 (0.4-0.8)	-1.23 (-1.42--1.03)
Larynx cancer	Hebei	Both	578.5 (410.3-777.9)	1.3 (1-1.8)	1035.8 (736.6-1421.1)	0.9 (0.7-1.3)	-1.23 (-1.37--1.09)
Larynx cancer	Hunan	Both	875.1 (608.9-1122.5)	2.1 (1.5-2.6)	1500.8 (1003.9-2151.5)	1.4 (1-2)	-1.21 (-1.27--1.14)

**Larynx cancer**	**Sichuan**	**Both**	**1082.1 (801.4-1397.8)**	**1.4 (1-1.7)**	**1378.9 (975.3-1951.2)**	**1 (0.7-1.3)**	**-1.13 (-1.35--0.9)**
**Larynx cancer**	**Guangxi**	**Both**	**562.6 (407.4-720.8)**	**1.9 (1.4-2.5)**	**911.9 (609.5-1239.9)**	**1.3 (0.9-1.8)**	**-1.02 (-1.38--0.66)**
**Larynx cancer**	**Liaoning**	**Both**	**519.5 (351-789.9)**	**1.9 (1.3-2.9)**	**1094.2 (755.5-1510.1)**	**1.4 (0.9-1.9)**	**-0.75 (-1.33--0.18)**

Appendix 14：Percentage change in ASIR for Tracheal, bronchus, and lung cancer；1990-2021

measure	location	sex	age	cause	metric	year_start	year_end	val	upper	lower
Incidence	Sichuan	Both	Age-standardized	Tracheal, bronchus, and lung cancer	Rate	1990	2021	0.73566974	1.53547243	0.19183133
Incidence	Henan	Both	Age-standardized	Tracheal, bronchus, and lung cancer	Rate	1990	2021	0.71376057	1.34528836	0.22493703
Incidence	Guangxi	Both	Age-standardized	Tracheal, bronchus, and lung cancer	Rate	1990	2021	0.60622059	1.35106396	0.10703263
Incidence	Hebei	Both	Age-standardized	Tracheal, bronchus, and lung cancer	Rate	1990	2021	0.44731108	1.08443744	-0.0566524
Incidence	Hubei	Both	Age-standardized	Tracheal, bronchus, and lung cancer	Rate	1990	2021	0.42783328	0.95917244	-0.0338741
Incidence	Shandong	Both	Age-standardized	Tracheal, bronchus, and lung cancer	Rate	1990	2021	0.39761716	1.02030326	-0.0446668
Incidence	Hunan	Both	Age-standardized	Tracheal, bronchus, and lung cancer	Rate	1990	2021	0.39278569	0.8884983	0.00381318
Incidence	Liaoning	Both	Age-standardized	Tracheal, bronchus, and lung cancer	Rate	1990	2021	0.36668337	0.89190241	-0.0259277
Incidence	Qinghai	Both	Age-standardized	Tracheal, bronchus, and lung cancer	Rate	1990	2021	0.35413825	0.95416716	-0.0757135
Incidence	Zhejiang	Both	Age-standardized	Tracheal, bronchus, and lung cancer	Rate	1990	2021	0.34434084	0.96795275	-0.049158
Incidence	China	Both	Age-standardized	Tracheal, bronchus, and lung cancer	Rate	1990	2021	0.32915792	0.71309171	0.01205423

Incidence	Jiangsu	Both	Age-standardized	Tracheal, bronchus, and lung cancer	Rate	1990	2021	0.32833641	0.84580542	-0.0592699
Incidence	Fujian	Both	Age-standardized	Tracheal, bronchus, and lung cancer	Rate	1990	2021	0.31423924	0.9018898	-0.0985843
Incidence	Shanxi	Both	Age-standardized	Tracheal, bronchus, and lung cancer	Rate	1990	2021	0.30933891	0.91818827	-0.1167168
Incidence	Yunnan	Both	Age-standardized	Tracheal, bronchus, and lung cancer	Rate	1990	2021	0.28496687	0.9324493	-0.1575101
Incidence	Guizhou	Both	Age-standardized	Tracheal, bronchus, and lung cancer	Rate	1990	2021	0.27467514	0.82872834	-0.1911497
Incidence	Ningxia	Both	Age-standardized	Tracheal, bronchus, and lung cancer	Rate	1990	2021	0.27316723	0.88946644	-0.1657854
Incidence	Tianjin	Both	Age-standardized	Tracheal, bronchus, and lung cancer	Rate	1990	2021	0.27069444	0.82726098	-0.1036323
Incidence	Guangdong	Both	Age-standardized	Tracheal, bronchus, and lung cancer	Rate	1990	2021	0.23599774	0.78323461	-0.1289114
Incidence	Inner Mongolia	Both	Age-standardized	Tracheal, bronchus, and lung cancer	Rate	1990	2021	0.1880764	0.73702001	-0.1795636
Incidence	Chongqing	Both	Age-standardized	Tracheal, bronchus, and lung cancer	Rate	1990	2021	0.17165339	0.71950787	-0.2271516
Incidence	Hainan	Both	Age-standardized	Tracheal, bronchus, and lung cancer	Rate	1990	2021	0.15702683	0.71910453	-0.2181061

Incidence	Shaanxi	Both	Age-standardized	Tracheal, bronchus, and lung cancer	Rate	1990	2021	0.15162255	0.64758881	-0.251177 1
Incidence	Gansu	Both	Age-standardized	Tracheal, bronchus, and lung cancer	Rate	1990	2021	0.13813678	0.61971996	-0.214075 9
Incidence	Anhui	Both	Age-standardized	Tracheal, bronchus, and lung cancer	Rate	1990	2021	0.13238267	0.62310068	-0.205053 9
Incidence	Taiwan (Province of China)	Both	Age-standardized	Tracheal, bronchus, and lung cancer	Rate	1990	2021	0.11680395	0.20923003	0.00337649
Incidence	Jilin	Both	Age-standardized	Tracheal, bronchus, and lung cancer	Rate	1990	2021	0.10119175	0.49984752	-0.201693 6
Incidence	Beijing	Both	Age-standardized	Tracheal, bronchus, and lung cancer	Rate	1990	2021	0.06290234	0.53494254	-0.250574 3
Incidence	Heilongjiang	Both	Age-standardized	Tracheal, bronchus, and lung cancer	Rate	1990	2021	0.03660733	0.44282099	-0.279865 8
Incidence	Shanghai	Both	Age-standardized	Tracheal, bronchus, and lung cancer	Rate	1990	2021	0.01417324	0.48383933	-0.292628 6
Incidence	Xinjiang	Both	Age-standardized	Tracheal, bronchus, and lung cancer	Rate	1990	2021	-0.005661	0.46710888	-0.332060 5
Incidence	Jiangxi	Both	Age-standardized	Tracheal, bronchus, and lung cancer	Rate	1990	2021	-0.013375 5	0.3793703	-0.316695 6
Incidence	Tibet	Both	Age-standardized	Tracheal, bronchus, and lung cancer	Rate	1990	2021	-0.181198 3	0.23128016	-0.471313 3

**Incidence**	**Macao Special Administrative Region of China**	**Both**	**Age-standardized**	**Tracheal, bronchus, and lung cancer**	**Rate**	**1990**	**2021**	**-0.263617**	**-0.014743**	**-0.462087 5**
**Incidence**	**Hong Kong Special Administrative Region of China**	**Both**	**Age-standardized**	**Tracheal, bronchus, and lung cancer**	**Rate**	**1990**	**2021**	**-0.310063 3**	**-0.095906 7**	**-0.483985 2**

Appendix 15：Percentage change in ASMR for Tracheal, bronchus, and lung cancer；1990-2021

measure	location	sex	age	cause	metric	year_start	year_end	val	upper	lower
Deaths	Henan	Both	Age-standardized	Tracheal, bronchus, and lung cancer	Rate	1990	2021	0.50956738	1.06029004	0.08253582
Deaths	Sichuan	Both	Age-standardized	Tracheal, bronchus, and lung cancer	Rate	1990	2021	0.44728458	1.06209558	-0.0013848
Deaths	Guangxi	Both	Age-standardized	Tracheal, bronchus, and lung cancer	Rate	1990	2021	0.32981768	0.90235747	-0.0759196
Deaths	Qinghai	Both	Age-standardized	Tracheal, bronchus, and lung cancer	Rate	1990	2021	0.28443138	0.86066012	-0.1389022
Deaths	Hebei	Both	Age-standardized	Tracheal, bronchus, and lung cancer	Rate	1990	2021	0.26076567	0.8477176	-0.1849395
Deaths	Shandong	Both	Age-standardized	Tracheal, bronchus, and lung cancer	Rate	1990	2021	0.22159782	0.7489761	-0.1541477
Deaths	Guizhou	Both	Age-standardized	Tracheal, bronchus, and lung cancer	Rate	1990	2021	0.20358976	0.79597797	-0.24998
Deaths	Liaoning	Both	Age-standardized	Tracheal, bronchus, and lung cancer	Rate	1990	2021	0.18505792	0.64952953	-0.1620124
Deaths	Shanxi	Both	Age-standardized	Tracheal, bronchus, and lung cancer	Rate	1990	2021	0.15826555	0.67855548	-0.2102534
Deaths	Hunan	Both	Age-standardized	Tracheal, bronchus, and lung cancer	Rate	1990	2021	0.15174212	0.57397716	-0.1724971

**Deaths**	**Yunnan**	**Both**	**Age-standardized**	**Tracheal, bronchus, and lung cancer**	**Rate**	**1990**	**2021**	**0.13565132**	**0.67934492**	**-0.2402268**
**Deaths**	**China**	**Both**	**Age-standardized**	**Tracheal, bronchus, and lung cancer**	**Rate**	**1990**	**2021**	**0.12172928**	**0.43734777**	**-0.138304**
**Deaths**	**Zhejiang**	**Both**	**Age-standardized**	**Tracheal, bronchus, and lung cancer**	**Rate**	**1990**	**2021**	**0.11387022**	**0.61138726**	**-0.2150441**
**Deaths**	**Hubei**	**Both**	**Age-standardized**	**Tracheal, bronchus, and lung cancer**	**Rate**	**1990**	**2021**	**0.09479563**	**0.51731101**	**-0.2336774**
**Deaths**	**Tianjin**	**Both**	**Age-standardized**	**Tracheal, bronchus, and lung cancer**	**Rate**	**1990**	**2021**	**0.08755983**	**0.53227772**	**-0.2348094**
**Deaths**	**Ningxia**	**Both**	**Age-standardized**	**Tracheal, bronchus, and lung cancer**	**Rate**	**1990**	**2021**	**0.06132293**	**0.58628462**	**-0.2779895**
**Deaths**	**Fujian**	**Both**	**Age-standardized**	**Tracheal, bronchus, and lung cancer**	**Rate**	**1990**	**2021**	**0.05448928**	**0.53441855**	**-0.2726363**
**Deaths**	**Jiangsu**	**Both**	**Age-standardized**	**Tracheal, bronchus, and lung cancer**	**Rate**	**1990**	**2021**	**0.05216429**	**0.46214423**	**-0.2433905**
**Deaths**	**Gansu**	**Both**	**Age-standardized**	**Tracheal, bronchus, and lung cancer**	**Rate**	**1990**	**2021**	**0.0332725**	**0.45251428**	**-0.2790397**
**Deaths**	**Hainan**	**Both**	**Age-standardized**	**Tracheal, bronchus, and lung cancer**	**Rate**	**1990**	**2021**	**0.03260665**	**0.49426234**	**-0.2930773**
**Deaths**	**Taiwan (Province of China)**	**Both**	**Age-standardized**	**Tracheal, bronchus, and lung cancer**	**Rate**	**1990**	**2021**	**0.03123015**	**0.1231417**	**-0.0727267**

Deaths	Chongqing	Both	Age-standardized	Tracheal, bronchus, and lung cancer	Rate	1990	2021	0.02180843	0.50211492	-0.32179 66
Deaths	Shaanxi	Both	Age-standardized	Tracheal, bronchus, and lung cancer	Rate	1990	2021	0.00087959	0.44093845	-0.335863
Deaths	Anhui	Both	Age-standardized	Tracheal, bronchus, and lung cancer	Rate	1990	2021	-0.0039183	0.42954384	-0.3095333
Deaths	Inner Mongolia	Both	Age-standardized	Tracheal, bronchus, and lung cancer	Rate	1990	2021	-0.0125358	0.42103894	-0.3047487
Deaths	Xinjiang	Both	Age-standardized	Tracheal, bronchus, and lung cancer	Rate	1990	2021	-0.0504839	0.38754609	-0.3584122
Deaths	Guangdong	Both	Age-standardized	Tracheal, bronchus, and lung cancer	Rate	1990	2021	-0.0666804	0.35084384	-0.3383502
Deaths	Heilongjiang	Both	Age-standardized	Tracheal, bronchus, and lung cancer	Rate	1990	2021	-0.0860788	0.26482791	-0.339378
Deaths	Jilin	Both	Age-standardized	Tracheal, bronchus, and lung cancer	Rate	1990	2021	-0.1044901	0.22490841	-0.3599331
Deaths	Beijing	Both	Age-standardized	Tracheal, bronchus, and lung cancer	Rate	1990	2021	-0.1177553	0.25664311	-0.3792608
Deaths	Jiangxi	Both	Age-standardized	Tracheal, bronchus, and lung cancer	Rate	1990	2021	-0.14996	0.18343927	-0.3910648
Deaths	Shanghai	Both	Age-standardized	Tracheal, bronchus, and lung cancer	Rate	1990	2021	-0.1563062	0.20130482	-0.4074052

**Deaths**	**Tibet**	**Both**	**Age-standardized**	**Tracheal, bronchus, and lung cancer**	**Rate**	**1990**	**2021**	**-0.186464 5**	**0.19 542011**	**-0.47 44313**
**Deaths**	**Macao Special Administrative Region of China**	**Both**	**Age-standardized**	**Tracheal, bronchus, and lung cancer**	**Rate**	**1990**	**2021**	**-0.357038 8**	**-0.128949 3**	**-0.531527 7**
**Deaths**	**Hong Kong Special Administrative Region of China**	**Both**	**Age-standardized**	**Tracheal, bronchus, and lung cancer**	**Rate**	**1990**	**2021**	**-0.400225 7**	**-0.216547 5**	**-0.558808 4**

Appendix 16：Percentage change in ASIR for Larynx cancer；1990-2021

measure	location	sex	age	cause	metric	year_start	year_end	val	upper	lower
Incidence	Liaoning	Both	Age-standardized	Larynx cancer	Rate	1990	2021	0.3024136 7	1.0167681 1	-0.1909763
Incidence	Sichuan	Both	Age-standardized	Larynx cancer	Rate	1990	2021	0.1885714	0.8419529 6	-0.2190753
Incidence	Zhejiang	Both	Age-standardized	Larynx cancer	Rate	1990	2021	0.1832864 3	0.8198180 6	-0.2599037
Incidence	Henan	Both	Age-standardized	Larynx cancer	Rate	1990	2021	0.1477157	0.6724222 4	-0.2020612
Incidence	Guangdong	Both	Age-standardized	Larynx cancer	Rate	1990	2021	0.0507824 8	0.6612648	-0.3499488
Incidence	Shandong	Both	Age-standardized	Larynx cancer	Rate	1990	2021	0.0478448 3	0.5933211 9	-0.3219188
Incidence	Fujian	Both	Age-standardized	Larynx cancer	Rate	1990	2021	0.0462784 7	0.6459233 5	-0.3450904
Incidence	Guangxi	Both	Age-standardized	Larynx cancer	Rate	1990	2021	0.0282939 6	0.5263783 5	-0.3076928
Incidence	Chongqing	Both	Age-standardized	Larynx cancer	Rate	1990	2021	0.0267452 4	0.6722582	-0.3829421
Incidence	Hebei	Both	Age-standardized	Larynx cancer	Rate	1990	2021	0.0257780 9	0.6445156 1	-0.3365298

Incidence	Hunan	Both	Age-standardized	Larynx cancer	Rate	1990	2021	0.0233468 7	0.4451970 1	-0.3183182
Incidence	Jiangsu	Both	Age-standardized	Larynx cancer	Rate	1990	2021	0.0034966 2	0.5316152	-0.3481508
Incidence	China	Both	Age-standardized	Larynx cancer	Rate	1990	2021	-0.0171914	0.2969489 1	-0.2615572
Incidence	Anhui	Both	Age-standardized	Larynx cancer	Rate	1990	2021	-0.0269833	0.5075943 1	-0.3758419
Incidence	Tianjin	Both	Age-standardized	Larynx cancer	Rate	1990	2021	-0.0735881	0.3430758 9	-0.3673641
Incidence	Hubei	Both	Age-standardized	Larynx cancer	Rate	1990	2021	-0.0769238	0.3811202 6	-0.3819975
Incidence	Shanxi	Both	Age-standardized	Larynx cancer	Rate	1990	2021	-0.0886812	0.3993962	-0.4160014
Incidence	Yunnan	Both	Age-standardized	Larynx cancer	Rate	1990	2021	-0.0975859	0.3303139 1	-0.4255015
Incidence	Inner Mongolia	Both	Age-standardized	Larynx cancer	Rate	1990	2021	-0.1001441	0.3596359 9	-0.3848941
Incidence	Beijing	Both	Age-standardized	Larynx cancer	Rate	1990	2021	-0.1098843	0.4394113 6	-0.4340901
Incidence	Hainan	Both	Age-standardized	Larynx cancer	Rate	1990	2021	-0.1123079	0.4204796 6	-0.4369801

Incidence	Qinghai	Both	Age-standardized	Larynx cancer	Rate	1990	2021	-0.121614	0.3387763	-0.404469
Incidence	Ningxia	Both	Age-standardized	Larynx cancer	Rate	1990	2021	-0.142057	0.2709104 2	-0.4590893
Incidence	Macao Special Administrative Region of China	Both	Age-standardized	Larynx cancer	Rate	1990	2021	-0.1465377	0.4386769 1	-0.4591431
Incidence	Taiwan (Province of China)	Both	Age-standardized	Larynx cancer	Rate	1990	2021	-0.1908209	-0.078216	-0.2997881
Incidence	Shaanxi	Both	Age-standardized	Larynx cancer	Rate	1990	2021	-0.1928047	0.2766229 2	-0.482492
Incidence	Gansu	Both	Age-standardized	Larynx cancer	Rate	1990	2021	-0.2084733	0.1225170 9	-0.4581326
Incidence	Heilongjiang	Both	Age-standardized	Larynx cancer	Rate	1990	2021	-0.2100176	0.1086008 2	-0.4534389
Incidence	Guizhou	Both	Age-standardized	Larynx cancer	Rate	1990	2021	-0.2209629	0.2362208 7	-0.5111386
Incidence	Jiangxi	Both	Age-standardized	Larynx cancer	Rate	1990	2021	-0.2659807	0.0659240 8	-0.5110582
Incidence	Shanghai	Both	Age-standardized	Larynx cancer	Rate	1990	2021	-0.2751812	0.1196169 9	-0.5375121
Incidence	Xinjiang	Both	Age-standardized	Larynx cancer	Rate	1990	2021	-0.3162743	0.0840700 9	-0.5762837

Incidence	Jilin	Both	Age-standardized	Larynx cancer	Rate	1990	2021	-0.3395184	-0.0316893	-0.566068
Incidence	Tibet	Both	Age-standardized	Larynx cancer	Rate	1990	2021	-0.4832008	-0.1663217	-0.6722929
Incidence	Hong Kong Special Administrative Region of China	Both	Age-standardized	Larynx cancer	Rate	1990	2021	-0.5387694	-0.3191703	-0.6792108

Appendix 17：Percentage change in ASIR for Larynx cancer；1990-2021

measure	location	sex	age	cause	metric	year_start	year_end	val	upper	lower
Deaths	Liaoning	Both	Age-standardized	Larynx cancer	Rate	1990	2021	-0.2656124	0.1181134 6	-0.5276513
Deaths	Sichuan	Both	Age-standardized	Larynx cancer	Rate	1990	2021	-0.2971236	0.0832174 2	-0.5363951
Deaths	Qinghai	Both	Age-standardized	Larynx cancer	Rate	1990	2021	-0.299158	0.0626710 1	-0.5307863
Deaths	Hebei	Both	Age-standardized	Larynx cancer	Rate	1990	2021	-0.3065392	0.0851064	-0.5520997
Deaths	Guangxi	Both	Age-standardized	Larynx cancer	Rate	1990	2021	-0.307091	0.0246375 5	-0.5310088
Deaths	Hunan	Both	Age-standardized	Larynx cancer	Rate	1990	2021	-0.3154891	-0.0179916	-0.5465866
Deaths	Henan	Both	Age-standardized	Larynx cancer	Rate	1990	2021	-0.32614	-0.0401774	-0.5261573
Deaths	Yunnan	Both	Age-standardized	Larynx cancer	Rate	1990	2021	-0.33183	-0.0164403	-0.5686571
Deaths	Hainan	Both	Age-standardized	Larynx cancer	Rate	1990	2021	-0.3875847	-0.039613	-0.6139568
Deaths	Shandong	Both	Age-standardized	Larynx cancer	Rate	1990	2021	-0.4022012	-0.1090881	-0.6161842

Deaths	Tianjin	Both	Age-standardized	Larynx cancer	Rate	1990	2021	-0.410131	-0.1434719	-0.5930723
Deaths	China	Both	Age-standardized	Larynx cancer	Rate	1990	2021	-0.4101526	-0.2343825	-0.5562333
Deaths	Shanxi	Both	Age-standardized	Larynx cancer	Rate	1990	2021	-0.4208828	-0.1339921	-0.61686
Deaths	Guizhou	Both	Age-standardized	Larynx cancer	Rate	1990	2021	-0.4278491	-0.0940173	-0.6427866
Deaths	Fujian	Both	Age-standardized	Larynx cancer	Rate	1990	2021	-0.4335933	-0.1174995	-0.6440719
Deaths	Jiangsu	Both	Age-standardized	Larynx cancer	Rate	1990	2021	-0.4341358	-0.1473598	-0.6233619
Deaths	Zhejiang	Both	Age-standardized	Larynx cancer	Rate	1990	2021	-0.4392475	-0.1516392	-0.6422123
Deaths	Gansu	Both	Age-standardized	Larynx cancer	Rate	1990	2021	-0.4416781	-0.2119437	-0.6113284
Deaths	Inner Mongolia	Both	Age-standardized	Larynx cancer	Rate	1990	2021	-0.4451898	-0.1807334	-0.6175699
Deaths	Chongqing	Both	Age-standardized	Larynx cancer	Rate	1990	2021	-0.4524891	-0.1574683	-0.6526601
Deaths	Hubei	Both	Age-standardized	Larynx cancer	Rate	1990	2021	-0.4550594	-0.1922625	-0.6387334

Deaths	Ningxia	Both	Age-standardized	Larynx cancer	Rate	1990	2021	-0.4584058	-0.2021252	-0.6459633
Deaths	Guangdong	Both	Age-standardized	Larynx cancer	Rate	1990	2021	-0.4668391	-0.1675253	-0.6577501
Deaths	Anhui	Both	Age-standardized	Larynx cancer	Rate	1990	2021	-0.4746553	-0.2067148	-0.6593672
Deaths	Xinjiang	Both	Age-standardized	Larynx cancer	Rate	1990	2021	-0.4769612	-0.1813436	-0.6687131
Deaths	Heilongjiang	Both	Age-standardized	Larynx cancer	Rate	1990	2021	-0.4775698	-0.2374601	-0.6363048
Deaths	Shaanxi	Both	Age-standardized	Larynx cancer	Rate	1990	2021	-0.4866487	-0.2353081	-0.6640981
Deaths	Macao Special Administrative Region of China	Both	Age-standardized	Larynx cancer	Rate	1990	2021	-0.4924815	-0.159219	-0.6802729
Deaths	Taiwan (Province of China)	Both	Age-standardized	Larynx cancer	Rate	1990	2021	-0.4961372	-0.4308117	-0.5565691
Deaths	Shanghai	Both	Age-standardized	Larynx cancer	Rate	1990	2021	-0.5122773	-0.2564789	-0.6879691
Deaths	Beijing	Both	Age-standardized	Larynx cancer	Rate	1990	2021	-0.514061	-0.242747	-0.6908809
Deaths	Jiangxi	Both	Age-standardized	Larynx cancer	Rate	1990	2021	-0.540888	-0.3268262	-0.6875518

**Deaths**	**Tibet**	**Both**	**Age-standardized**	**Larynx cancer**	**Rate**	**1990**	**2021**	**-0.5423573**	**-0.2493071**	**-0.7100033**
**Deaths**	**Jilin**	**Both**	**Age-standardized**	**Larynx cancer**	**Rate**	**1990**	**2021**	**-0.589018**	**-0.4019938**	**-0.7244579**
**Deaths**	**Hong Kong Special Administrative Region of China**	**Both**	**Age-standardized**	**Larynx cancer**	**Rate**	**1990**	**2021**	**-0.6993916**	**-0.5470509**	**-0.7926055**

Appendix 18：ASIR of tracheal, bronchus, and lung cancer，2021

measure	location	sex	age	cause	metric	year	val	upper	lower
Incidence	Hebei	Male	Age-standardized	Tracheal, bronchus, and lung cancer	Rate	2021	48.8923442	68.3832501	30.2700847
Incidence	Hebei	Female	Age-standardized	Tracheal, bronchus, and lung cancer	Rate	2021	27.553332	37.04378	19.6831607
Incidence	Qinghai	Male	Age-standardized	Tracheal, bronchus, and lung cancer	Rate	2021	39.9410732	56.6268096	26.9321943
Incidence	Qinghai	Female	Age-standardized	Tracheal, bronchus, and lung cancer	Rate	2021	21.8275158	30.5598468	15.1747426
Incidence	Shandong	Male	Age-standardized	Tracheal, bronchus, and lung cancer	Rate	2021	65.4894842	93.4452793	45.2567188
Incidence	Shandong	Female	Age-standardized	Tracheal, bronchus, and lung cancer	Rate	2021	31.3718178	42.1935922	22.8063059
Incidence	Henan	Male	Age-standardized	Tracheal, bronchus, and lung cancer	Rate	2021	57.7604533	80.8337284	40.3456641
Incidence	Henan	Female	Age-standardized	Tracheal, bronchus, and lung cancer	Rate	2021	22.5417818	31.1199556	15.8699309
Incidence	Anhui	Male	Age-standardized	Tracheal, bronchus, and lung cancer	Rate	2021	60.1982506	84.8460931	42.5118544
Incidence	Anhui	Female	Age-standardized	Tracheal, bronchus, and lung cancer	Rate	2021	24.1209668	33.9572394	16.8562577

Incidence	Hunan	Male	Age-standardized	Tracheal, bronchus, and lung cancer	Rate	2021	62.4938368	87.5950603	43.312923
Incidence	Hunan	Female	Age-standardized	Tracheal, bronchus, and lung cancer	Rate	2021	21.5681287	29.3572745	15.6063975
Incidence	Shanxi	Male	Age-standardized	Tracheal, bronchus, and lung cancer	Rate	2021	58.0562016	84.6097043	39.5655099
Incidence	Shanxi	Female	Age-standardized	Tracheal, bronchus, and lung cancer	Rate	2021	24.3609097	35.024983	17.0336557
Incidence	Jilin	Male	Age-standardized	Tracheal, bronchus, and lung cancer	Rate	2021	55.7571143	80.5888689	38.3982459
Incidence	Jilin	Female	Age-standardized	Tracheal, bronchus, and lung cancer	Rate	2021	50.3577146	71.4405941	34.1852523
Incidence	Tianjin	Male	Age-standardized	Tracheal, bronchus, and lung cancer	Rate	2021	76.208621	107.365911	52.6014767
Incidence	Tianjin	Female	Age-standardized	Tracheal, bronchus, and lung cancer	Rate	2021	52.0099506	71.8258774	36.8547142
Incidence	Gansu	Male	Age-standardized	Tracheal, bronchus, and lung cancer	Rate	2021	29.5222453	40.889218	20.7193953
Incidence	Gansu	Female	Age-standardized	Tracheal, bronchus, and lung cancer	Rate	2021	12.9889116	17.6695663	9.24280959
Incidence	Chongqing	Male	Age-standardized	Tracheal, bronchus, and lung cancer	Rate	2021	84.8572473	120.303179	55.4074412

Incidence	Chongqing	Female	Age-standardized	Tracheal, bronchus, and lung cancer	Rate	2021	37.9408895	52.692162	26.1941567
Incidence	Jiangsu	Male	Age-standardized	Tracheal, bronchus, and lung cancer	Rate	2021	65.090232	92.8078834	42.9505053
Incidence	Jiangsu	Female	Age-standardized	Tracheal, bronchus, and lung cancer	Rate	2021	27.2303007	37.498462	18.7901356
Incidence	Hong Kong Special Administrative Region of China	Male	Age-standardized	Tracheal, bronchus, and lung cancer	Rate	2021	46.1305208	60.8181936	34.1652815
Incidence	Hong Kong Special Administrative Region of China	Female	Age-standardized	Tracheal, bronchus, and lung cancer	Rate	2021	22.0537137	29.1325983	15.9830594
Incidence	Xinjiang	Male	Age-standardized	Tracheal, bronchus, and lung cancer	Rate	2021	27.951567	43.275021	16.3564827
Incidence	Xinjiang	Female	Age-standardized	Tracheal, bronchus, and lung cancer	Rate	2021	18.4903651	26.350233	12.7405489
Incidence	Guangxi	Male	Age-standardized	Tracheal, bronchus, and lung cancer	Rate	2021	51.7867261	71.2879852	34.6088726
Incidence	Guangxi	Female	Age-standardized	Tracheal, bronchus, and lung cancer	Rate	2021	19.093998	27.2877722	13.0482623
Incidence	Zhejiang	Male	Age-standardized	Tracheal, bronchus, and lung cancer	Rate	2021	76.173294	108.336315	50.7613873
Incidence	Zhejiang	Female	Age-standardized	Tracheal, bronchus, and lung cancer	Rate	2021	28.3914266	40.0655716	19.8522727

Incidence	Ningxia	Male	Age-standardized	Tracheal, bronchus, and lung cancer	Rate	2021	48.0239547	69.5886516	31.6968288
Incidence	Ningxia	Female	Age-standardized	Tracheal, bronchus, and lung cancer	Rate	2021	23.8002855	34.3112723	15.7187964
Incidence	Heilongjiang	Male	Age-standardized	Tracheal, bronchus, and lung cancer	Rate	2021	89.8375296	122.635871	61.9619579
Incidence	Heilongjiang	Female	Age-standardized	Tracheal, bronchus, and lung cancer	Rate	2021	54.9173979	74.0461591	38.8242995
Incidence	Shanghai	Male	Age-standardized	Tracheal, bronchus, and lung cancer	Rate	2021	63.6117153	91.7144783	41.6840831
Incidence	Shanghai	Female	Age-standardized	Tracheal, bronchus, and lung cancer	Rate	2021	26.3985607	37.5771531	17.9181865
Incidence	Hainan	Male	Age-standardized	Tracheal, bronchus, and lung cancer	Rate	2021	49.9963521	72.0516845	31.6372739
Incidence	Hainan	Female	Age-standardized	Tracheal, bronchus, and lung cancer	Rate	2021	20.9705684	29.5722199	14.2873991
Incidence	Liaoning	Male	Age-standardized	Tracheal, bronchus, and lung cancer	Rate	2021	82.3705967	113.28118	58.7518037
Incidence	Liaoning	Female	Age-standardized	Tracheal, bronchus, and lung cancer	Rate	2021	44.1687332	60.9643502	31.0672797
Incidence	Shaanxi	Male	Age-standardized	Tracheal, bronchus, and lung cancer	Rate	2021	44.8415373	65.3111911	29.6266706

Incidence	Shaanxi	Female	Age-standardized	Tracheal, bronchus, and lung cancer	Rate	2021	19.2898588	27.425818	13.0856461
Incidence	Beijing	Male	Age-standardized	Tracheal, bronchus, and lung cancer	Rate	2021	60.4985396	85.9779613	41.6120594
Incidence	Beijing	Female	Age-standardized	Tracheal, bronchus, and lung cancer	Rate	2021	33.5729713	45.9606973	23.4243534
Incidence	Inner Mongolia	Male	Age-standardized	Tracheal, bronchus, and lung cancer	Rate	2021	64.9813891	90.4090754	44.2295843
Incidence	Inner Mongolia	Female	Age-standardized	Tracheal, bronchus, and lung cancer	Rate	2021	32.6610355	44.4739764	22.96845
Incidence	Guangdong	Male	Age-standardized	Tracheal, bronchus, and lung cancer	Rate	2021	64.5267438	91.4830406	44.4697354
Incidence	Guangdong	Female	Age-standardized	Tracheal, bronchus, and lung cancer	Rate	2021	27.5695969	38.3747081	19.2771042
Incidence	Jiangxi	Male	Age-standardized	Tracheal, bronchus, and lung cancer	Rate	2021	61.4762551	84.3889895	42.2344926
Incidence	Jiangxi	Female	Age-standardized	Tracheal, bronchus, and lung cancer	Rate	2021	22.2293854	30.4457426	15.7996313
Incidence	Sichuan	Male	Age-standardized	Tracheal, bronchus, and lung cancer	Rate	2021	70.4829273	101.966431	46.9022179
Incidence	Sichuan	Female	Age-standardized	Tracheal, bronchus, and lung cancer	Rate	2021	31.3872945	44.3796725	20.810503

Incidence	Hubei	Male	Age-standardized	Tracheal, bronchus, and lung cancer	Rate	2021	79.0726006	110.31554	52.3639296
Incidence	Hubei	Female	Age-standardized	Tracheal, bronchus, and lung cancer	Rate	2021	29.4218623	40.5548656	20.6760501
Incidence	Fujian	Male	Age-standardized	Tracheal, bronchus, and lung cancer	Rate	2021	56.4517317	81.233623	38.5716306
Incidence	Fujian	Female	Age-standardized	Tracheal, bronchus, and lung cancer	Rate	2021	20.9412522	29.4148063	14.5291292
Incidence	Macao Special Administrative Region of China	Male	Age-standardized	Tracheal, bronchus, and lung cancer	Rate	2021	38.534804	52.5349849	25.3281928
Incidence	Macao Special Administrative Region of China	Female	Age-standardized	Tracheal, bronchus, and lung cancer	Rate	2021	24.2274384	31.8269389	17.8763927
Incidence	China	Male	Age-standardized	Tracheal, bronchus, and lung cancer	Rate	2021	62.6314425	79.9041084	46.4990674
Incidence	China	Female	Age-standardized	Tracheal, bronchus, and lung cancer	Rate	2021	28.1634322	34.9005548	22.2219998
Incidence	Taiwan (Province of China)	Male	Age-standardized	Tracheal, bronchus, and lung cancer	Rate	2021	38.4754658	41.4928395	35.0275186
Incidence	Taiwan (Province of China)	Female	Age-standardized	Tracheal, bronchus, and lung cancer	Rate	2021	20.3861055	22.6210595	17.8643507
Incidence	Tibet	Male	Age-standardized	Tracheal, bronchus, and lung cancer	Rate	2021	15.1360394	21.8482741	9.9009213

Incidence	Tibet	Female	Age-standardized	Tracheal, bronchus, and lung cancer	Rate	2021	5.36946393	7.66570312	3.76592097
Incidence	Yunnan	Male	Age-standardized	Tracheal, bronchus, and lung cancer	Rate	2021	48.6125574	69.1806416	31.0610143
Incidence	Yunnan	Female	Age-standardized	Tracheal, bronchus, and lung cancer	Rate	2021	17.6045503	23.880541	12.3205581
Incidence	Guizhou	Male	Age-standardized	Tracheal, bronchus, and lung cancer	Rate	2021	50.8140256	74.9372742	30.8214676
Incidence	Guizhou	Female	Age-standardized	Tracheal, bronchus, and lung cancer	Rate	2021	23.5027961	34.1026497	15.593496

Appendix 19：ASMR of tracheal, bronchus, and lung cancer，2021

measure	location	sex	age	cause	metric	year	val	upper	lower
Deaths	Fujian	Male	Age-standardized	Tracheal, bronchus, and lung cancer	Rate	2021	48.3059538	69.781988	32.8955202
Deaths	Fujian	Female	Age-standardized	Tracheal, bronchus, and lung cancer	Rate	2021	16.7164386	23.3854135	11.6129635
Deaths	Shandong	Male	Age-standardized	Tracheal, bronchus, and lung cancer	Rate	2021	61.7837258	87.4459068	42.8207522
Deaths	Shandong	Female	Age-standardized	Tracheal, bronchus, and lung cancer	Rate	2021	29.3061528	39.0009102	21.3104785
Deaths	Guizhou	Male	Age-standardized	Tracheal, bronchus, and lung cancer	Rate	2021	51.7107222	75.8168365	31.1751794
Deaths	Guizhou	Female	Age-standardized	Tracheal, bronchus, and lung cancer	Rate	2021	23.6213099	33.8036654	15.7601259
Deaths	Hainan	Male	Age-standardized	Tracheal, bronchus, and lung cancer	Rate	2021	46.8474885	67.2272183	31.1131853
Deaths	Hainan	Female	Age-standardized	Tracheal, bronchus, and lung cancer	Rate	2021	20.0823464	28.1632915	13.9129729
Deaths	Tianjin	Male	Age-standardized	Tracheal, bronchus, and lung cancer	Rate	2021	65.599043	91.7325778	45.0957293
Deaths	Tianjin	Female	Age-standardized	Tracheal, bronchus, and lung cancer	Rate	2021	44.7991354	62.2191102	31.7629303
Deaths	Hubei	Male	Age-standardized	Tracheal, bronchus, and lung cancer	Rate	2021	62.8544967	88.7174393	41.9655242
Deaths	Hubei	Female	Age-standardized	Tracheal, bronchus, and lung cancer	Rate	2021	22.6947259	31.8223936	15.7106368
Deaths	Jilin	Male	Age-standardized	Tracheal, bronchus, and lung cancer	Rate	2021	49.3815398	70.947447	34.3553913

Deaths	Jilin	Female	Age-standardized	Tracheal, bronchus, and lung cancer	Rate	2021	41.940287	58.6846424	28.5844675
Deaths	China	Male	Age-standardized	Tracheal, bronchus, and lung cancer	Rate	2021	56.431345	72.4125875	42.2671221
Deaths	China	Female	Age-standardized	Tracheal, bronchus, and lung cancer	Rate	2021	24.4231069	30.3225106	18.9317569
Deaths	Taiwan (Province of China)	Male	Age-standardized	Tracheal, bronchus, and lung cancer	Rate	2021	37.0135384	40.2997789	33.470385
Deaths	Taiwan (Province of China)	Female	Age-standardized	Tracheal, bronchus, and lung cancer	Rate	2021	18.7450245	20.9380245	16.3278053
Deaths	Ningxia	Male	Age-standardized	Tracheal, bronchus, and lung cancer	Rate	2021	43.2806079	63.3835624	29.2717603
Deaths	Ningxia	Female	Age-standardized	Tracheal, bronchus, and lung cancer	Rate	2021	20.9521468	30.1413599	14.3204874
Deaths	Guangdong	Male	Age-standardized	Tracheal, bronchus, and lung cancer	Rate	2021	51.8134432	72.4754929	35.5512651
Deaths	Guangdong	Female	Age-standardized	Tracheal, bronchus, and lung cancer	Rate	2021	20.2034828	27.592893	14.1604693
Deaths	Gansu	Male	Age-standardized	Tracheal, bronchus, and lung cancer	Rate	2021	29.2340519	40.1766181	20.3697692
Deaths	Gansu	Female	Age-standardized	Tracheal, bronchus, and lung cancer	Rate	2021	12.2995924	16.6275484	8.76994216
Deaths	Shanxi	Male	Age-standardized	Tracheal, bronchus, and lung cancer	Rate	2021	55.4408268	80.4013241	37.423117
Deaths	Shanxi	Female	Age-standardized	Tracheal, bronchus, and lung cancer	Rate	2021	22.3395881	31.7374131	15.4872607
Deaths	Guangxi	Male	Age-standardized	Tracheal, bronchus, and lung cancer	Rate	2021	45.0070859	62.5863667	29.8729065

Deaths	Guangxi	Female	Age-standardized	Tracheal, bronchus, and lung cancer	Rate	2021	16.44371577	22.92351699	11.27781511
Deaths	Shanghai	Male	Age-standardized	Tracheal, bronchus, and lung cancer	Rate	2021	53.48037533	75.98082486	35.490476
Deaths	Shanghai	Female	Age-standardized	Tracheal, bronchus, and lung cancer	Rate	2021	21.42452777	30.28642677	14.57731477
Deaths	Qinghai	Male	Age-standardized	Tracheal, bronchus, and lung cancer	Rate	2021	41.390635	58.51457444	27.95257111
Deaths	Qinghai	Female	Age-standardized	Tracheal, bronchus, and lung cancer	Rate	2021	21.21014666	29.25942044	14.48128011
Deaths	Shaanxi	Male	Age-standardized	Tracheal, bronchus, and lung cancer	Rate	2021	42.43578522	60.50381877	27.92839455
Deaths	Shaanxi	Female	Age-standardized	Tracheal, bronchus, and lung cancer	Rate	2021	17.288813	24.22056866	11.61247388
Deaths	Heilongjiang	Male	Age-standardized	Tracheal, bronchus, and lung cancer	Rate	2021	81.52946899	112.535234	56.86217
Deaths	Heilongjiang	Female	Age-standardized	Tracheal, bronchus, and lung cancer	Rate	2021	51.33274355	69.88139099	36.51014255
Deaths	Hebei	Male	Age-standardized	Tracheal, bronchus, and lung cancer	Rate	2021	46.69345988	65.86819999	29.03984211
Deaths	Hebei	Female	Age-standardized	Tracheal, bronchus, and lung cancer	Rate	2021	24.32624799	32.75603399	17.32536955
Deaths	Yunnan	Male	Age-standardized	Tracheal, bronchus, and lung cancer	Rate	2021	45.89955966	64.43973711	29.81442066
Deaths	Yunnan	Female	Age-standardized	Tracheal, bronchus, and lung cancer	Rate	2021	16.64116466	22.38780788	11.57495588
Deaths	Sichuan	Male	Age-standardized	Tracheal, bronchus, and lung cancer	Rate	2021	62.90714499	89.229234	43.13110855

Deaths	Sichuan	Female	Age-standardized	Tracheal, bronchus, and lung cancer	Rate	2021	26.691187	37.859242 2	17.97 1856 1
Deaths	Tibet	Male	Age-standardized	Tracheal, bronchus, and lung cancer	Rate	2021	15.97962	22.940111 5	10.531123 9
Deaths	Tibet	Female	Age-standardized	Tracheal, bronchus, and lung cancer	Rate	2021	5.5367315 4	7.8960971	3.8281906 8
Deaths	Henan	Male	Age-standardized	Tracheal, bronchus, and lung cancer	Rate	2021	55.309296 9	76.320293 8	38.490459 7
Deaths	Henan	Female	Age-standardized	Tracheal, bronchus, and lung cancer	Rate	2021	20.371778 8	27.994088 2	14.108071 1
Deaths	Hong Kong Special Administrative Region of China	Male	Age-standardized	Tracheal, bronchus, and lung cancer	Rate	2021	41.586766 1	54.647030 6	30.786252 7
Deaths	Hong Kong Special Administrative Region of China	Female	Age-standardized	Tracheal, bronchus, and lung cancer	Rate	2021	19.267538 5	25.047274 4	13.897994 5
Deaths	Xinjiang	Male	Age-standardized	Tracheal, bronchus, and lung cancer	Rate	2021	28.847459	43.562520 5	16.922330 9
Deaths	Xinjiang	Female	Age-standardized	Tracheal, bronchus, and lung cancer	Rate	2021	18.601738 9	26.429793 7	12.970812 1
Deaths	Inner Mongolia	Male	Age-standardized	Tracheal, bronchus, and lung cancer	Rate	2021	58.395079 6	80.098776 5	39.753529 6
Deaths	Inner Mongolia	Female	Age-standardized	Tracheal, bronchus, and lung cancer	Rate	2021	27.956007 8	37.813613 3	19.853686
Deaths	Hunan	Male	Age-standardized	Tracheal, bronchus, and lung cancer	Rate	2021	55.579445 4	77.474159 6	39.077402 2
Deaths	Hunan	Female	Age-standardized	Tracheal, bronchus, and lung cancer	Rate	2021	17.927435 6	24.565391 4	12.978479 7
Deaths	Zhejiang	Male	Age-standardized	Tracheal, bronchus, and lung cancer	Rate	2021	67.265444 4	92.965612 4	45.363821 9

Deaths	Zhejiang	Female	Age-standardized	Tracheal, bronchus, and lung cancer	Rate	2021	24.6945045	35.0112316	17.3228924
Deaths	Jiangsu	Male	Age-standardized	Tracheal, bronchus, and lung cancer	Rate	2021	54.5268238	76.8831844	35.8713593
Deaths	Jiangsu	Female	Age-standardized	Tracheal, bronchus, and lung cancer	Rate	2021	21.893062	29.9831577	14.9130321
Deaths	Jiangxi	Male	Age-standardized	Tracheal, bronchus, and lung cancer	Rate	2021	57.2739145	78.8960086	39.4332999
Deaths	Jiangxi	Female	Age-standardized	Tracheal, bronchus, and lung cancer	Rate	2021	19.7791191	27.060205	14.0527809
Deaths	Macao Special Administrative Region of China	Male	Age-standardized	Tracheal, bronchus, and lung cancer	Rate	2021	35.5258093	48.4949578	23.2179567
Deaths	Macao Special Administrative Region of China	Female	Age-standardized	Tracheal, bronchus, and lung cancer	Rate	2021	22.2696222	29.4524844	16.6309645
Deaths	Anhui	Male	Age-standardized	Tracheal, bronchus, and lung cancer	Rate	2021	58.8661712	82.8820388	41.0782553
Deaths	Anhui	Female	Age-standardized	Tracheal, bronchus, and lung cancer	Rate	2021	20.7577196	29.0224317	14.4715622
Deaths	Chongqing	Male	Age-standardized	Tracheal, bronchus, and lung cancer	Rate	2021	80.2864787	115.402178	52.6834853
Deaths	Chongqing	Female	Age-standardized	Tracheal, bronchus, and lung cancer	Rate	2021	33.2770556	46.2653538	23.1594019
Deaths	Liaoning	Male	Age-standardized	Tracheal, bronchus, and lung cancer	Rate	2021	76.2494271	103.999787	54.6972939
Deaths	Liaoning	Female	Age-standardized	Tracheal, bronchus, and lung cancer	Rate	2021	41.2723102	56.1646919	28.8168048
Deaths	Beijing	Male	Age-standardized	Tracheal, bronchus, and lung cancer	Rate	2021	52.0118029	72.7450893	35.6238971

**Deaths**	**Beijing**	**Female**	**Age-standardized**	**Tracheal, bronchus, and lung cancer**	**Rate**	**2021**	**27.717475 5**	**36.942894 7**	**19.418017 4**

Appendix 20：ASIR of larynx cancer，2021

measure	location	sex	age	cause	metric	year	val	upper	lower
Incidence	Hebei	Male	Age-standardized	Larynx cancer	Rate	2021	2.62177107	3.83806601	1.64446351
Incidence	Hebei	Female	Age-standardized	Larynx cancer	Rate	2021	0.66888569	1.05082798	0.33308947
Incidence	Qinghai	Male	Age-standardized	Larynx cancer	Rate	2021	1.20276067	1.85109305	0.75066024
Incidence	Qinghai	Female	Age-standardized	Larynx cancer	Rate	2021	0.38557491	0.63298002	0.23927317
Incidence	Anhui	Male	Age-standardized	Larynx cancer	Rate	2021	2.92079271	4.38803285	1.98742306
Incidence	Anhui	Female	Age-standardized	Larynx cancer	Rate	2021	0.47454925	0.75604557	0.30150695
Incidence	Shandong	Male	Age-standardized	Larynx cancer	Rate	2021	2.54972002	3.81661916	1.69014889
Incidence	Shandong	Female	Age-standardized	Larynx cancer	Rate	2021	0.48693686	0.762653	0.31341893
Incidence	Henan	Male	Age-standardized	Larynx cancer	Rate	2021	2.02016474	2.98605763	1.33871695
Incidence	Henan	Female	Age-standardized	Larynx cancer	Rate	2021	0.46969765	0.71411974	0.30116975
Incidence	Hunan	Male	Age-standardized	Larynx cancer	Rate	2021	3.82330135	5.48264146	2.36957074
Incidence	Hunan	Female	Age-standardized	Larynx cancer	Rate	2021	0.74536169	1.21336694	0.2692336
Incidence	Jiangsu	Male	Age-standardized	Larynx cancer	Rate	2021	1.96901749	2.92803587	1.28202866

Incidence	Jiangsu	Female	Age-standardized	Larynx cancer	Rate	2021	0.38044367	0.8059098	0.22853618
Incidence	Chongqing	Male	Age-standardized	Larynx cancer	Rate	2021	3.07904617	4.83308716	1.91289255
Incidence	Chongqing	Female	Age-standardized	Larynx cancer	Rate	2021	0.47179418	0.71512514	0.30211523
Incidence	Shanxi	Male	Age-standardized	Larynx cancer	Rate	2021	3.13724546	4.89858495	2.10304883
Incidence	Shanxi	Female	Age-standardized	Larynx cancer	Rate	2021	0.73912806	1.19948124	0.36046744
Incidence	Hong Kong Special Administrative Region of China	Male	Age-standardized	Larynx cancer	Rate	2021	2.36764597	3.27292517	1.5654232
Incidence	Hong Kong Special Administrative Region of China	Female	Age-standardized	Larynx cancer	Rate	2021	0.2274243	0.71232896	0.12371735
Incidence	Jilin	Male	Age-standardized	Larynx cancer	Rate	2021	3.37231191	4.84783474	2.10017492
Incidence	Jilin	Female	Age-standardized	Larynx cancer	Rate	2021	1.28536726	2.07482229	0.41671371
Incidence	Tianjin	Male	Age-standardized	Larynx cancer	Rate	2021	3.449921	5.13964089	2.21561154
Incidence	Tianjin	Female	Age-standardized	Larynx cancer	Rate	2021	0.87134413	1.24697442	0.48697192
Incidence	Gansu	Male	Age-standardized	Larynx cancer	Rate	2021	1.19595773	1.67946744	0.80913979
Incidence	Gansu	Female	Age-standardized	Larynx cancer	Rate	2021	0.28783605	0.53632978	0.1766541
Incidence	Hainan	Male	Age-standardized	Larynx cancer	Rate	2021	6.17287575	9.35074474	3.42472718

Incidence	Hainan	Female	Age-standardized	Larynx cancer	Rate	2021	0.92923973	1.61431747	0.25682116
Incidence	Xinjiang	Male	Age-standardized	Larynx cancer	Rate	2021	1.20577786	1.78053109	0.76658004
Incidence	Xinjiang	Female	Age-standardized	Larynx cancer	Rate	2021	0.24733094	0.51844328	0.12401233
Incidence	Guangxi	Male	Age-standardized	Larynx cancer	Rate	2021	3.82948416	5.39964576	2.44690693
Incidence	Guangxi	Female	Age-standardized	Larynx cancer	Rate	2021	0.63031833	1.00967267	0.24074818
Incidence	Zhejiang	Male	Age-standardized	Larynx cancer	Rate	2021	3.35311141	4.84851267	2.25151679
Incidence	Zhejiang	Female	Age-standardized	Larynx cancer	Rate	2021	0.35903859	0.84001699	0.19893558
Incidence	Ningxia	Male	Age-standardized	Larynx cancer	Rate	2021	1.39028507	2.12837309	0.92043026
Incidence	Ningxia	Female	Age-standardized	Larynx cancer	Rate	2021	0.40139824	0.64486849	0.25112869
Incidence	Heilongjiang	Male	Age-standardized	Larynx cancer	Rate	2021	5.28159598	7.54861693	3.10986405
Incidence	Heilongjiang	Female	Age-standardized	Larynx cancer	Rate	2021	1.20201066	1.90745479	0.47323483
Incidence	Hubei	Male	Age-standardized	Larynx cancer	Rate	2021	3.87262645	5.73930511	2.57749032
Incidence	Hubei	Female	Age-standardized	Larynx cancer	Rate	2021	0.64899004	0.97696033	0.35597734
Incidence	Beijing	Male	Age-standardized	Larynx cancer	Rate	2021	2.49765649	3.84400311	1.56712409

Incidence	Beijing	Female	Age-standardized	Larynx cancer	Rate	2021	0.46653927	0.82491983	0.28602933
Incidence	Shaanxi	Male	Age-standardized	Larynx cancer	Rate	2021	2.05150803	3.1173043	1.35093933
Incidence	Shaanxi	Female	Age-standardized	Larynx cancer	Rate	2021	0.43344438	0.66193874	0.27348943
Incidence	Sichuan	Male	Age-standardized	Larynx cancer	Rate	2021	3.18504951	4.69568086	2.09071243
Incidence	Sichuan	Female	Age-standardized	Larynx cancer	Rate	2021	0.4660661	0.67712239	0.30024564
Incidence	Macao Special Administrative Region of China	Male	Age-standardized	Larynx cancer	Rate	2021	3.46692524	5.4465628	2.2523283
Incidence	Macao Special Administrative Region of China	Female	Age-standardized	Larynx cancer	Rate	2021	0.40901786	0.80005589	0.23102275
Incidence	Jiangxi	Male	Age-standardized	Larynx cancer	Rate	2021	3.03682156	4.18479842	2.02164168
Incidence	Jiangxi	Female	Age-standardized	Larynx cancer	Rate	2021	0.64109202	1.0215522	0.28483599
Incidence	Inner Mongolia	Male	Age-standardized	Larynx cancer	Rate	2021	3.16890506	4.53895308	2.14247454
Incidence	Inner Mongolia	Female	Age-standardized	Larynx cancer	Rate	2021	0.69219931	1.03836317	0.40269129
Incidence	Shanghai	Male	Age-standardized	Larynx cancer	Rate	2021	2.59861779	4.00810912	1.63863696
Incidence	Shanghai	Female	Age-standardized	Larynx cancer	Rate	2021	0.24522655	0.60934152	0.1244642
Incidence	Liaoning	Male	Age-standardized	Larynx cancer	Rate	2021	5.54408776	7.92745969	3.61723867

Incidence	Liaoning	Female	Age-standardized	Larynx cancer	Rate	2021	0.88042905	1.33434362	0.42405315
Incidence	Fujian	Male	Age-standardized	Larynx cancer	Rate	2021	2.54663933	3.79440899	1.63463702
Incidence	Fujian	Female	Age-standardized	Larynx cancer	Rate	2021	0.35851444	0.63477482	0.2208375
Incidence	Yunnan	Male	Age-standardized	Larynx cancer	Rate	2021	2.57996896	3.68369682	1.72635298
Incidence	Yunnan	Female	Age-standardized	Larynx cancer	Rate	2021	0.47859608	0.73649434	0.27304362
Incidence	Taiwan (Province of China)	Male	Age-standardized	Larynx cancer	Rate	2021	3.42205871	3.82825952	2.9698108
Incidence	Taiwan (Province of China)	Female	Age-standardized	Larynx cancer	Rate	2021	0.16252928	0.18346036	0.14161669
Incidence	Tibet	Male	Age-standardized	Larynx cancer	Rate	2021	0.8578555	1.3153736	0.55665062
Incidence	Tibet	Female	Age-standardized	Larynx cancer	Rate	2021	0.07313876	0.32773505	0.01861098
Incidence	Guizhou	Male	Age-standardized	Larynx cancer	Rate	2021	4.08723565	6.04834684	2.4338178
Incidence	Guizhou	Female	Age-standardized	Larynx cancer	Rate	2021	0.9457817	1.64982294	0.2905438
Incidence	Guangdong	Male	Age-standardized	Larynx cancer	Rate	2021	4.49406226	6.48175024	2.71071142
Incidence	Guangdong	Female	Age-standardized	Larynx cancer	Rate	2021	0.61540404	0.93136264	0.35108606
Incidence	China	Male	Age-standardized	Larynx cancer	Rate	2021	3.12388357	4.04004787	2.34251674

Incidence	China	Female	Age-standardized	Larynx cancer	Rate	2021	0.57538871	0.79118223	0.34705861

Appendix 21：ASMR of larynx cancer，2021

measure	location	sex	age	cause	metric	year	val	upper	lower
Deaths	Fujian	Male	Age-standardized	Larynx cancer	Rate	2021	1.22377887	1.81093426	0.79380746
Deaths	Fujian	Female	Age-standardized	Larynx cancer	Rate	2021	0.17288191	0.30086328	0.1059828
Deaths	Shandong	Male	Age-standardized	Larynx cancer	Rate	2021	1.32280687	1.91237493	0.90623348
Deaths	Shandong	Female	Age-standardized	Larynx cancer	Rate	2021	0.22655745	0.34828218	0.14940558
Deaths	Guizhou	Male	Age-standardized	Larynx cancer	Rate	2021	2.95328087	4.3404415	1.81549841
Deaths	Guizhou	Female	Age-standardized	Larynx cancer	Rate	2021	0.67149361	1.19380805	0.20889968
Deaths	Hainan	Male	Age-standardized	Larynx cancer	Rate	2021	3.88375152	5.89869102	2.37489478
Deaths	Hainan	Female	Age-standardized	Larynx cancer	Rate	2021	0.57092859	0.96466309	0.1571237
Deaths	Tianjin	Male	Age-standardized	Larynx cancer	Rate	2021	1.66636154	2.42152943	1.11077578
Deaths	Tianjin	Female	Age-standardized	Larynx cancer	Rate	2021	0.39305888	0.57396271	0.20355878
Deaths	Hubei	Male	Age-standardized	Larynx cancer	Rate	2021	1.94219406	2.80337064	1.31078896
Deaths	Hubei	Female	Age-standardized	Larynx cancer	Rate	2021	0.33104727	0.51915585	0.17578912
Deaths	Jilin	Male	Age-standardized	Larynx cancer	Rate	2021	2.00598447	2.92612971	1.27510125

Deaths	Jilin	Female	Age-standardized	Larynx cancer	Rate	2021	0.66956976	1.08700803	0.21410097
Deaths	China	Male	Age-standardized	Larynx cancer	Rate	2021	1.67599657	2.15022341	1.26503282
Deaths	China	Female	Age-standardized	Larynx cancer	Rate	2021	0.30430743	0.42762664	0.17915218
Deaths	Taiwan (Province of China)	Male	Age-standardized	Larynx cancer	Rate	2021	1.19219943	1.33172876	1.05832003
Deaths	Taiwan (Province of China)	Female	Age-standardized	Larynx cancer	Rate	2021	0.07118336	0.07989793	0.06169399
Deaths	Ningxia	Male	Age-standardized	Larynx cancer	Rate	2021	0.8345602	1.24616936	0.55746294
Deaths	Ningxia	Female	Age-standardized	Larynx cancer	Rate	2021	0.22922783	0.36507266	0.14478273
Deaths	Guangdong	Male	Age-standardized	Larynx cancer	Rate	2021	1.93732572	2.80846206	1.24939802
Deaths	Guangdong	Female	Age-standardized	Larynx cancer	Rate	2021	0.27004397	0.41153238	0.15416609
Deaths	Gansu	Male	Age-standardized	Larynx cancer	Rate	2021	0.81004444	1.15179162	0.54714477
Deaths	Gansu	Female	Age-standardized	Larynx cancer	Rate	2021	0.18903747	0.32807323	0.11900877
Deaths	Shanxi	Male	Age-standardized	Larynx cancer	Rate	2021	1.78153251	2.676975	1.1994008
Deaths	Shanxi	Female	Age-standardized	Larynx cancer	Rate	2021	0.40065646	0.63430881	0.18765197
Deaths	Guangxi	Male	Age-standardized	Larynx cancer	Rate	2021	2.44070522	3.37107337	1.56619318

Deaths	Guangxi	Female	Age-standardized	Larynx cancer	Rate	2021	0.37426753	0.60048743	0.13883736
Deaths	Shanghai	Male	Age-standardized	Larynx cancer	Rate	2021	1.40464738	2.11770982	0.89803456
Deaths	Shanghai	Female	Age-standardized	Larynx cancer	Rate	2021	0.10631224	0.23681255	0.05684033
Deaths	Qinghai	Male	Age-standardized	Larynx cancer	Rate	2021	0.93762024	1.43132181	0.59676154
Deaths	Qinghai	Female	Age-standardized	Larynx cancer	Rate	2021	0.29099052	0.44843393	0.18032694
Deaths	Shaanxi	Male	Age-standardized	Larynx cancer	Rate	2021	1.23587871	1.76356514	0.83085844
Deaths	Shaanxi	Female	Age-standardized	Larynx cancer	Rate	2021	0.24918946	0.37301136	0.15633166
Deaths	Heilongjiang	Male	Age-standardized	Larynx cancer	Rate	2021	3.14265491	4.52652812	1.99677352
Deaths	Heilongjiang	Female	Age-standardized	Larynx cancer	Rate	2021	0.65246695	1.03467165	0.24916134
Deaths	Hebei	Male	Age-standardized	Larynx cancer	Rate	2021	1.58816561	2.30796689	1.01589558
Deaths	Hebei	Female	Age-standardized	Larynx cancer	Rate	2021	0.38602677	0.61953454	0.19470888
Deaths	Yunnan	Male	Age-standardized	Larynx cancer	Rate	2021	1.88358596	2.63625321	1.25199788
Deaths	Yunnan	Female	Age-standardized	Larynx cancer	Rate	2021	0.33972542	0.52815327	0.19428742
Deaths	Sichuan	Male	Age-standardized	Larynx cancer	Rate	2021	1.70213173	2.49855462	1.13829199

Deaths	Sichuan	Female	Age-standardized	Larynx cancer	Rate	2021	0.27138952	0.41265976	0.16936639
Deaths	Tibet	Male	Age-standardized	Larynx cancer	Rate	2021	0.74482456	1.12462091	0.48175668
Deaths	Tibet	Female	Age-standardized	Larynx cancer	Rate	2021	0.06174602	0.26656786	0.01569547
Deaths	Henan	Male	Age-standardized	Larynx cancer	Rate	2021	1.09405785	1.5678707	0.71499161
Deaths	Henan	Female	Age-standardized	Larynx cancer	Rate	2021	0.23130206	0.35725058	0.15141455
Deaths	Hong Kong Special Administrative Region of China	Male	Age-standardized	Larynx cancer	Rate	2021	1.02757651	1.40547908	0.70464591
Deaths	Hong Kong Special Administrative Region of China	Female	Age-standardized	Larynx cancer	Rate	2021	0.08983215	0.27367932	0.04705034
Deaths	Xinjiang	Male	Age-standardized	Larynx cancer	Rate	2021	0.90311501	1.33859961	0.58792969
Deaths	Xinjiang	Female	Age-standardized	Larynx cancer	Rate	2021	0.17883847	0.37099997	0.09122246
Deaths	Hunan	Male	Age-standardized	Larynx cancer	Rate	2021	2.52968985	3.61204095	1.63739473
Deaths	Hunan	Female	Age-standardized	Larynx cancer	Rate	2021	0.43097419	0.69133542	0.15408503
Deaths	Zhejiang	Male	Age-standardized	Larynx cancer	Rate	2021	1.2882477	1.85900893	0.87262472
Deaths	Zhejiang	Female	Age-standardized	Larynx cancer	Rate	2021	0.15138088	0.31645924	0.08638378
Deaths	Jiangsu	Male	Age-standardized	Larynx cancer	Rate	2021	1.0105712	1.4709607	0.67156237

Deaths	Jiangsu	Female	Age-standardized	Larynx cancer	Rate	2021	0.16767822	0.33645157	0.10133757
Deaths	Inner Mongolia	Male	Age-standardized	Larynx cancer	Rate	2021	1.79712555	2.63685673	1.23654528
Deaths	Inner Mongolia	Female	Age-standardized	Larynx cancer	Rate	2021	0.37754873	0.56620345	0.21683542
Deaths	Jiangxi	Male	Age-standardized	Larynx cancer	Rate	2021	1.83557152	2.53123613	1.2605048
Deaths	Jiangxi	Female	Age-standardized	Larynx cancer	Rate	2021	0.3734782	0.60272279	0.15807305
Deaths	Macao Special Administrative Region of China	Male	Age-standardized	Larynx cancer	Rate	2021	1.489558	2.30900398	0.97897707
Deaths	Macao Special Administrative Region of China	Female	Age-standardized	Larynx cancer	Rate	2021	0.1648704	0.31760563	0.0934018
Deaths	Anhui	Male	Age-standardized	Larynx cancer	Rate	2021	1.38799825	2.0248472	0.9545026
Deaths	Anhui	Female	Age-standardized	Larynx cancer	Rate	2021	0.24119492	0.37846437	0.15426522
Deaths	Chongqing	Male	Age-standardized	Larynx cancer	Rate	2021	1.4198398	2.10547023	0.91489288
Deaths	Chongqing	Female	Age-standardized	Larynx cancer	Rate	2021	0.25018774	0.37867804	0.16075382
Deaths	Liaoning	Male	Age-standardized	Larynx cancer	Rate	2021	2.47219186	3.48146175	1.65248304
Deaths	Liaoning	Female	Age-standardized	Larynx cancer	Rate	2021	0.43491058	0.6667142	0.19953346
Deaths	Beijing	Male	Age-standardized	Larynx cancer	Rate	2021	1.14630991	1.73916814	0.72428755

**Deaths**	**Beijing**	**Female**	**Age-standardized**	**Larynx cancer**	**Rate**	**2021**	**0.19110384**	**0.32949484**	**0.11914517**

Appendix 22:Age burden of tracheal, bronchus, and lung cancer in China from 1990 to 2021

measure	location	sex	age	cause	metric	year	val	upper	lower
Deaths	China	Male	15-19 years	Tracheal, bronchus, and lung cancer	Number	1990	346.939653	436.068635	270.253861
Deaths	China	Female	15-19 years	Tracheal, bronchus, and lung cancer	Number	1990	189.872086	233.874641	156.480305
Deaths	China	Both	15-19 years	Tracheal, bronchus, and lung cancer	Number	1990	536.811739	646.991946	447.33875
Deaths	China	Male	15-19 years	Tracheal, bronchus, and lung cancer	Rate	1990	0.53349536	0.67055061	0.41557424
Deaths	China	Female	15-19 years	Tracheal, bronchus, and lung cancer	Rate	1990	0.30806498	0.37945856	0.25388725
Deaths	China	Both	15-19 years	Tracheal, bronchus, and lung cancer	Rate	1990	0.42380363	0.51078901	0.35316625
Deaths	China	Male	20-24 years	Tracheal, bronchus, and lung cancer	Number	1990	496.307923	614.632141	394.175335
Deaths	China	Female	20-24 years	Tracheal, bronchus, and lung cancer	Number	1990	301.839094	396.284352	231.048713
Deaths	China	Both	20-24 years	Tracheal, bronchus, and lung cancer	Number	1990	798.147017	954.076355	665.554634
Deaths	China	Male	20-24 years	Tracheal, bronchus, and lung cancer	Rate	1990	0.73511877	0.91037762	0.58384256
Deaths	China	Female	20-24 years	Tracheal, bronchus, and lung cancer	Rate	1990	0.46806244	0.61451888	0.35828767
Deaths	China	Both	20-24 years	Tracheal, bronchus, and lung cancer	Rate	1990	0.60465268	0.72278015	0.50420459
Deaths	China	Male	25-29 years	Tracheal, bronchus, and lung cancer	Number	1990	731.208829	881.692527	600.957001
Deaths	China	Female	25-29 years	Tracheal, bronchus, and lung cancer	Number	1990	472.136084	624.055749	356.397635
Deaths	China	Both	25-29 years	Tracheal, bronchus, and lung cancer	Number	1990	1203.34491	1426.25299	1020.0455
Deaths	China	Male	25-29 years	Tracheal, bronchus, and lung cancer	Rate	1990	1.29586057	1.56255031	1.06502609
Deaths	China	Female	25-29 years	Tracheal, bronchus, and lung cancer	Rate	1990	0.88310888	1.16726764	0.66662542
Deaths	China	Both	25-29 years	Tracheal, bronchus, and lung cancer	Rate	1990	1.09505037	1.29789793	0.92824691
Deaths	China	Male	30-34 years	Tracheal, bronchus, and lung cancer	Number	1990	1485.79792	1791.83238	1213.73606
Deaths	China	Female	30-34 years	Tracheal, bronchus, and lung cancer	Number	1990	926.13362	1193.64884	705.515115

Deaths	China	Both	30-34 years	Tracheal, bronchus, and lung cancer	Number	1990	2411.93154	2829.25112	2054.42025
Deaths	China	Male	30-34 years	Tracheal, bronchus, and lung cancer	Rate	1990	3.22825718	3.89319145	2.63713666
Deaths	China	Female	30-34 years	Tracheal, bronchus, and lung cancer	Rate	1990	2.19361306	2.82724181	1.67106251
Deaths	China	Both	30-34 years	Tracheal, bronchus, and lung cancer	Rate	1990	2.73324276	3.20615657	2.32810475
Deaths	China	Male	35-39 years	Tracheal, bronchus, and lung cancer	Number	1990	3547.04742	4365.41413	2846.38934
Deaths	China	Female	35-39 years	Tracheal, bronchus, and lung cancer	Number	1990	2114.23309	2658.83193	1652.92761
Deaths	China	Both	35-39 years	Tracheal, bronchus, and lung cancer	Number	1990	5661.28052	6566.87471	4820.38073
Deaths	China	Male	35-39 years	Tracheal, bronchus, and lung cancer	Rate	1990	7.5107775	9.243647	6.02715285
Deaths	China	Female	35-39 years	Tracheal, bronchus, and lung cancer	Rate	1990	4.79277145	6.0273268	3.74703446
Deaths	China	Both	35-39 years	Tracheal, bronchus, and lung cancer	Rate	1990	6.19809394	7.1895583	5.27745843
Deaths	China	Male	40-44 years	Tracheal, bronchus, and lung cancer	Number	1990	6111.8038	7600.50014	4855.8198
Deaths	China	Female	40-44 years	Tracheal, bronchus, and lung cancer	Number	1990	3260.25012	4059.85527	2548.62655
Deaths	China	Both	40-44 years	Tracheal, bronchus, and lung cancer	Number	1990	9372.05392	10965.8081	7905.80086
Deaths	China	Male	40-44 years	Tracheal, bronchus, and lung cancer	Rate	1990	17.3729235	21.6045724	13.8027641
Deaths	China	Female	40-44 years	Tracheal, bronchus, and lung cancer	Rate	1990	10.2156462	12.7211237	7.98584961
Deaths	China	Both	40-44 years	Tracheal, bronchus, and lung cancer	Rate	1990	13.9684727	16.3438657	11.7831122
Deaths	China	Male	45-49 years	Tracheal, bronchus, and lung cancer	Number	1990	8758.03949	10980.7352	6846.81481
Deaths	China	Female	45-49 years	Tracheal, bronchus, and lung cancer	Number	1990	3781.47599	4775.30722	2949.85108
Deaths	China	Both	45-49 years	Tracheal, bronchus, and lung cancer	Number	1990	12539.5155	14874.0086	10484.9783
Deaths	China	Male	45-49 years	Tracheal, bronchus, and lung cancer	Rate	1990	32.1527726	40.3127987	25.1362282
Deaths	China	Female	45-49 years	Tracheal, bronchus, and lung cancer	Rate	1990	15.5104867	19.586886	12.0994094
Deaths	China	Both	45-49 years	Tracheal, bronchus, and lung cancer	Rate	1990	24.2924625	28.8150125	20.3122634

Deaths	China	Male	50-54 years	Tracheal, bronchus, and lung cancer	Number	1990	17221.137	21605.9799	13439.5813
Deaths	China	Female	50-54 years	Tracheal, bronchus, and lung cancer	Number	1990	6744.57086	8398.62294	5343.13375
Deaths	China	Both	50-54 years	Tracheal, bronchus, and lung cancer	Number	1990	23965.7078	28655.5056	19749.7794
Deaths	China	Male	50-54 years	Tracheal, bronchus, and lung cancer	Rate	1990	68.1874294	85.5493011	53.2142856
Deaths	China	Female	50-54 years	Tracheal, bronchus, and lung cancer	Rate	1990	30.035558	37.4015384	23.7945463
Deaths	China	Both	50-54 years	Tracheal, bronchus, and lung cancer	Rate	1990	50.2311205	60.0607402	41.394711
Deaths	China	Male	55-59 years	Tracheal, bronchus, and lung cancer	Number	1990	25896.6499	32490.974	20059.2922
Deaths	China	Female	55-59 years	Tracheal, bronchus, and lung cancer	Number	1990	9974.80246	12222.8525	7970.40019
Deaths	China	Both	55-59 years	Tracheal, bronchus, and lung cancer	Number	1990	35871.4524	42654.2933	29739.4852
Deaths	China	Male	55-59 years	Tracheal, bronchus, and lung cancer	Rate	1990	113.937872	142.951017	88.2551634
Deaths	China	Female	55-59 years	Tracheal, bronchus, and lung cancer	Rate	1990	48.326698	59.2182257	38.6156141
Deaths	China	Both	55-59 years	Tracheal, bronchus, and lung cancer	Rate	1990	82.7120013	98.3518015	68.5729788
Deaths	China	Male	60-64 years	Tracheal, bronchus, and lung cancer	Number	1990	31124.8575	38692.4726	24464.3644
Deaths	China	Female	60-64 years	Tracheal, bronchus, and lung cancer	Number	1990	11638.9194	14151.7281	9451.57513
Deaths	China	Both	60-64 years	Tracheal, bronchus, and lung cancer	Number	1990	42763.7769	50435.5573	35922.9304
Deaths	China	Male	60-64 years	Tracheal, bronchus, and lung cancer	Rate	1990	170.988296	212.561935	134.398044
Deaths	China	Female	60-64 years	Tracheal, bronchus, and lung cancer	Rate	1990	67.9266331	82.5917945	55.1609349
Deaths	China	Both	60-64 years	Tracheal, bronchus, and lung cancer	Rate	1990	121.015417	142.725467	101.656793
Deaths	China	Male	65-69 years	Tracheal, bronchus, and lung cancer	Number	1990	33639.4928	41252.7944	26850.1621
Deaths	China	Female	65-69 years	Tracheal, bronchus, and lung cancer	Number	1990	13497.26	16379.731	11126.5688
Deaths	China	Both	65-69 years	Tracheal, bronchus, and lung cancer	Number	1990	47136.7528	55287.4027	39883.5073
Deaths	China	Male	65-69 years	Tracheal, bronchus, and lung cancer	Rate	1990	250.87988	307.659101	200.24575

Deaths	China	Female	65-69 years	Tracheal, bronchus, and lung cancer	Rate	1990	97.2901276	118.06738	80.2018555
Deaths	China	Both	65-69 years	Tracheal, bronchus, and lung cancer	Rate	1990	172.777211	202.652976	146.190833
Deaths	China	Male	70-74 years	Tracheal, bronchus, and lung cancer	Number	1990	30057.8661	36243.0783	24451.1818
Deaths	China	Female	70-74 years	Tracheal, bronchus, and lung cancer	Number	1990	13234.1667	16028.9401	11001.5984
Deaths	China	Both	70-74 years	Tracheal, bronchus, and lung cancer	Number	1990	43292.0328	49739.4114	37197.1626
Deaths	China	Male	70-74 years	Tracheal, bronchus, and lung cancer	Rate	1990	344.490251	415.37836	280.232593
Deaths	China	Female	70-74 years	Tracheal, bronchus, and lung cancer	Rate	1990	131.130883	158.822926	109.009456
Deaths	China	Both	70-74 years	Tracheal, bronchus, and lung cancer	Rate	1990	230.060771	264.32317	197.671658
Deaths	China	Male	75-79 years	Tracheal, bronchus, and lung cancer	Number	1990	19968.7379	23955.3755	16385.5896
Deaths	China	Female	75-79 years	Tracheal, bronchus, and lung cancer	Number	1990	10531.9437	12772.1695	8791.00483
Deaths	China	Both	75-79 years	Tracheal, bronchus, and lung cancer	Number	1990	30500.6817	34999.4344	26594.879
Deaths	China	Male	75-79 years	Tracheal, bronchus, and lung cancer	Rate	1990	407.771545	489.180664	334.601878
Deaths	China	Female	75-79 years	Tracheal, bronchus, and lung cancer	Rate	1990	162.438334	196.990223	135.587145
Deaths	China	Both	75-79 years	Tracheal, bronchus, and lung cancer	Rate	1990	268.003633	307.533309	233.684094
Deaths	China	Male	80-84	Tracheal, bronchus, and lung cancer	Number	1990	8750.8503	10578.6556	7187.28706
Deaths	China	Female	80-84	Tracheal, bronchus, and lung cancer	Number	1990	5858.61499	6912.49795	4910.92109
Deaths	China	Both	80-84	Tracheal, bronchus, and lung cancer	Number	1990	14609.4653	16749.1353	12598.4405
Deaths	China	Male	80-84	Tracheal, bronchus, and lung cancer	Rate	1990	428.995054	518.599994	352.344115
Deaths	China	Female	80-84	Tracheal, bronchus, and lung cancer	Rate	1990	179.862365	212.21709	150.767696
Deaths	China	Both	80-84	Tracheal, bronchus, and lung cancer	Rate	1990	275.79987	316.192909	237.835415
Deaths	China	Male	85-89	Tracheal, bronchus, and lung cancer	Number	1990	3451.78258	4116.52486	2880.12339
Deaths	China	Female	85-89	Tracheal, bronchus, and lung cancer	Number	1990	2594.47977	3121.84576	2136.43031

Deaths	China	Both	85-89	Tracheal, bronchus, and lung cancer	Number	1990	6046.26235	6945.30229	5218.32884
Deaths	China	Male	85-89	Tracheal, bronchus, and lung cancer	Rate	1990	629.177103	750.343662	524.977355
Deaths	China	Female	85-89	Tracheal, bronchus, and lung cancer	Rate	1990	227.938456	274.270284	187.696444
Deaths	China	Both	85-89	Tracheal, bronchus, and lung cancer	Rate	1990	358.43391	411.730705	309.35244
Deaths	China	Male	90-94	Tracheal, bronchus, and lung cancer	Number	1990	775.646013	927.869624	657.495171
Deaths	China	Female	90-94	Tracheal, bronchus, and lung cancer	Number	1990	605.656302	735.710209	488.069678
Deaths	China	Both	90-94	Tracheal, bronchus, and lung cancer	Number	1990	1381.30232	1580.9564	1183.02951
Deaths	China	Male	90-94	Tracheal, bronchus, and lung cancer	Rate	1990	951.957263	1138.78266	806.949683
Deaths	China	Female	90-94	Tracheal, bronchus, and lung cancer	Rate	1990	268.770861	326.484618	216.589685
Deaths	China	Both	90-94	Tracheal, bronchus, and lung cancer	Rate	1990	450.196528	515.268146	385.5751
Deaths	China	Male	95+ years	Tracheal, bronchus, and lung cancer	Number	1990	38.9304644	47.4895205	31.6351806
Deaths	China	Female	95+ years	Tracheal, bronchus, and lung cancer	Number	1990	105.778968	128.959395	83.4183717
Deaths	China	Both	95+ years	Tracheal, bronchus, and lung cancer	Number	1990	144.709432	172.294568	115.92644
Deaths	China	Male	95+ years	Tracheal, bronchus, and lung cancer	Rate	1990	501.083762	611.249519	407.184337
Deaths	China	Female	95+ years	Tracheal, bronchus, and lung cancer	Rate	1990	323.257363	394.096057	254.92405
Deaths	China	Both	95+ years	Tracheal, bronchus, and lung cancer	Rate	1990	357.377074	425.501832	286.294068
Deaths	China	Male	15-19 years	Tracheal, bronchus, and lung cancer	Number	2000	231.702523	266.070202	198.918576
Deaths	China	Female	15-19 years	Tracheal, bronchus, and lung cancer	Number	2000	146.479502	172.392453	125.044504
Deaths	China	Both	15-19 years	Tracheal, bronchus, and lung cancer	Number	2000	378.182025	425.10147	335.563848
Deaths	China	Male	15-19 years	Tracheal, bronchus, and lung cancer	Rate	2000	0.43483711	0.49933508	0.37331133
Deaths	China	Female	15-19 years	Tracheal, bronchus, and lung cancer	Rate	2000	0.28955741	0.34078156	0.24718519
Deaths	China	Both	15-19 years	Tracheal, bronchus, and lung cancer	Rate	2000	0.36408369	0.40925401	0.32305429

Deaths	China	Male	20-24 years	Tracheal, bronchus, and lung cancer	Number	2000	335.274113	383.972329	289.446008
Deaths	China	Female	20-24 years	Tracheal, bronchus, and lung cancer	Number	2000	212.15369	254.621818	176.641557
Deaths	China	Both	20-24 years	Tracheal, bronchus, and lung cancer	Number	2000	547.427802	616.749963	479.85423
Deaths	China	Male	20-24 years	Tracheal, bronchus, and lung cancer	Rate	2000	0.69460464	0.79549524	0.5996602
Deaths	China	Female	20-24 years	Tracheal, bronchus, and lung cancer	Rate	2000	0.45248278	0.54305908	0.37674227
Deaths	China	Both	20-24 years	Tracheal, bronchus, and lung cancer	Rate	2000	0.57530165	0.64815355	0.50428738
Deaths	China	Male	25-29 years	Tracheal, bronchus, and lung cancer	Number	2000	807.741683	932.898542	692.206015
Deaths	China	Female	25-29 years	Tracheal, bronchus, and lung cancer	Number	2000	473.08173	571.798449	383.650578
Deaths	China	Both	25-29 years	Tracheal, bronchus, and lung cancer	Number	2000	1280.82341	1450.93513	1127.34455
Deaths	China	Male	25-29 years	Tracheal, bronchus, and lung cancer	Rate	2000	1.33356641	1.54019804	1.14281918
Deaths	China	Female	25-29 years	Tracheal, bronchus, and lung cancer	Rate	2000	0.8215735	0.99300908	0.66626363
Deaths	China	Both	25-29 years	Tracheal, bronchus, and lung cancer	Rate	2000	1.08404316	1.22801964	0.95414413
Deaths	China	Male	30-34 years	Tracheal, bronchus, and lung cancer	Number	2000	2221.78962	2540.29943	1885.98342
Deaths	China	Female	30-34 years	Tracheal, bronchus, and lung cancer	Number	2000	1245.61187	1535.34679	988.397139
Deaths	China	Both	30-34 years	Tracheal, bronchus, and lung cancer	Number	2000	3467.40149	3931.25453	2988.50175
Deaths	China	Male	30-34 years	Tracheal, bronchus, and lung cancer	Rate	2000	3.37260555	3.85609326	2.86286249
Deaths	China	Female	30-34 years	Tracheal, bronchus, and lung cancer	Rate	2000	1.99621355	2.4605418	1.58400205
Deaths	China	Both	30-34 years	Tracheal, bronchus, and lung cancer	Rate	2000	2.70307321	3.06467793	2.32973858
Deaths	China	Male	35-39 years	Tracheal, bronchus, and lung cancer	Number	2000	4180.81131	4783.09039	3626.90627
Deaths	China	Female	35-39 years	Tracheal, bronchus, and lung cancer	Number	2000	2498.60027	2939.78511	2123.97167
Deaths	China	Both	35-39 years	Tracheal, bronchus, and lung cancer	Number	2000	6679.41159	7476.48286	5938.66967
Deaths	China	Male	35-39 years	Tracheal, bronchus, and lung cancer	Rate	2000	7.40101778	8.46719318	6.42047578

Deaths	China	Female	35-39 years	Tracheal, bronchus, and lung cancer	Rate	2000	4.67955804	5.50584069	3.97792668
Deaths	China	Both	35-39 years	Tracheal, bronchus, and lung cancer	Rate	2000	6.07862366	6.80400137	5.40450869
Deaths	China	Male	40-44 years	Tracheal, bronchus, and lung cancer	Number	2000	7314.12025	8465.69167	6318.60679
Deaths	China	Female	40-44 years	Tracheal, bronchus, and lung cancer	Number	2000	4124.69898	4764.92441	3573.2732
Deaths	China	Both	40-44 years	Tracheal, bronchus, and lung cancer	Number	2000	11438.8192	12801.7128	10261.3179
Deaths	China	Male	40-44 years	Tracheal, bronchus, and lung cancer	Rate	2000	17.1381073	19.8364161	14.8054663
Deaths	China	Female	40-44 years	Tracheal, bronchus, and lung cancer	Rate	2000	10.4606727	12.0843521	9.06219866
Deaths	China	Both	40-44 years	Tracheal, bronchus, and lung cancer	Rate	2000	13.9314208	15.5912987	12.4973334
Deaths	China	Male	45-49 years	Tracheal, bronchus, and lung cancer	Number	2000	14323.7573	16581.941	12362.0975
Deaths	China	Female	45-49 years	Tracheal, bronchus, and lung cancer	Number	2000	6521.56122	7734.62087	5444.23903
Deaths	China	Both	45-49 years	Tracheal, bronchus, and lung cancer	Number	2000	20845.3185	23400.4669	18376.5991
Deaths	China	Male	45-49 years	Tracheal, bronchus, and lung cancer	Rate	2000	32.3521571	37.4525727	27.9214812
Deaths	China	Female	45-49 years	Tracheal, bronchus, and lung cancer	Rate	2000	15.5634865	18.4584126	12.9924934
Deaths	China	Both	45-49 years	Tracheal, bronchus, and lung cancer	Rate	2000	24.1888284	27.1538128	21.3241358
Deaths	China	Male	50-54 years	Tracheal, bronchus, and lung cancer	Number	2000	22143.6612	25421.4815	19242.8025
Deaths	China	Female	50-54 years	Tracheal, bronchus, and lung cancer	Number	2000	9147.90553	10613.276	7900.02369
Deaths	China	Both	50-54 years	Tracheal, bronchus, and lung cancer	Number	2000	31291.5667	35128.6619	27786.8218
Deaths	China	Male	50-54 years	Tracheal, bronchus, and lung cancer	Rate	2000	66.9979554	76.9153425	58.221105
Deaths	China	Female	50-54 years	Tracheal, bronchus, and lung cancer	Rate	2000	29.7784848	34.5485943	25.7163494
Deaths	China	Both	50-54 years	Tracheal, bronchus, and lung cancer	Rate	2000	49.0685712	55.0855527	43.5727511
Deaths	China	Male	55-59 years	Tracheal, bronchus, and lung cancer	Number	2000	26756.8664	30639.378	23065.1215
Deaths	China	Female	55-59 years	Tracheal, bronchus, and lung cancer	Number	2000	11018.6791	12892.7081	9379.05249

Deaths	China	Both	55-59 years	Tracheal, bronchus, and lung cancer	Number	2000	37775.5455	42469.8327	33609.0507
Deaths	China	Male	55-59 years	Tracheal, bronchus, and lung cancer	Rate	2000	110.188309	126.177004	94.9852159
Deaths	China	Female	55-59 years	Tracheal, bronchus, and lung cancer	Rate	2000	48.9972315	57.3305562	41.7062336
Deaths	China	Both	55-59 years	Tracheal, bronchus, and lung cancer	Rate	2000	80.7666369	90.8033361	71.8583929
Deaths	China	Male	60-64 years	Tracheal, bronchus, and lung cancer	Number	2000	37392.2578	42227.3688	32513.9141
Deaths	China	Female	60-64 years	Tracheal, bronchus, and lung cancer	Number	2000	14658.5807	16729.6156	12829.3961
Deaths	China	Both	60-64 years	Tracheal, bronchus, and lung cancer	Number	2000	52050.8385	57390.6911	46664.1567
Deaths	China	Male	60-64 years	Tracheal, bronchus, and lung cancer	Rate	2000	171.369362	193.528759	149.011828
Deaths	China	Female	60-64 years	Tracheal, bronchus, and lung cancer	Rate	2000	72.6930568	82.9634821	63.6219863
Deaths	China	Both	60-64 years	Tracheal, bronchus, and lung cancer	Rate	2000	123.975665	136.694226	111.145565
Deaths	China	Male	65-69 years	Tracheal, bronchus, and lung cancer	Number	2000	47597.8206	53417.403	41677.1882
Deaths	China	Female	65-69 years	Tracheal, bronchus, and lung cancer	Number	2000	19388.6748	22115.0487	17012.9152
Deaths	China	Both	65-69 years	Tracheal, bronchus, and lung cancer	Number	2000	66986.4954	73895.3323	60462.2385
Deaths	China	Male	65-69 years	Tracheal, bronchus, and lung cancer	Rate	2000	267.315784	299.999344	234.064714
Deaths	China	Female	65-69 years	Tracheal, bronchus, and lung cancer	Rate	2000	111.16274	126.794092	97.5415952
Deaths	China	Both	65-69 years	Tracheal, bronchus, and lung cancer	Rate	2000	190.045859	209.646763	171.536038
Deaths	China	Male	70-74 years	Tracheal, bronchus, and lung cancer	Number	2000	49525.209	55767.4878	43579.5783
Deaths	China	Female	70-74 years	Tracheal, bronchus, and lung cancer	Number	2000	21022.0498	23938.0506	18568.9996
Deaths	China	Both	70-74 years	Tracheal, bronchus, and lung cancer	Number	2000	70547.2589	77643.846	63898.351
Deaths	China	Male	70-74 years	Tracheal, bronchus, and lung cancer	Rate	2000	394.592035	444.327383	347.22023
Deaths	China	Female	70-74 years	Tracheal, bronchus, and lung cancer	Rate	2000	158.563686	180.558298	140.060985
Deaths	China	Both	70-74 years	Tracheal, bronchus, and lung cancer	Rate	2000	273.345897	300.842685	247.58371

Deaths	China	Male	75-79 years	Tracheal, bronchus, and lung cancer	Number	2000	35429.565	39821.0123	31272.2467
Deaths	China	Female	75-79 years	Tracheal, bronchus, and lung cancer	Number	2000	17730.9476	20102.1366	15488.594
Deaths	China	Both	75-79 years	Tracheal, bronchus, and lung cancer	Number	2000	53160.5126	58796.9621	48121.5797
Deaths	China	Male	75-79 years	Tracheal, bronchus, and lung cancer	Rate	2000	487.041121	547.409218	429.891535
Deaths	China	Female	75-79 years	Tracheal, bronchus, and lung cancer	Rate	2000	199.802597	226.52253	174.534457
Deaths	China	Both	75-79 years	Tracheal, bronchus, and lung cancer	Rate	2000	329.194101	364.097563	297.990734
Deaths	China	Male	80-84	Tracheal, bronchus, and lung cancer	Number	2000	17497.4759	19834.509	15427.0007
Deaths	China	Female	80-84	Tracheal, bronchus, and lung cancer	Number	2000	10650.0192	12162.0777	9067.64189
Deaths	China	Both	80-84	Tracheal, bronchus, and lung cancer	Number	2000	28147.4951	31447.851	24965.6973
Deaths	China	Male	80-84	Tracheal, bronchus, and lung cancer	Rate	2000	533.196233	604.412062	470.103158
Deaths	China	Female	80-84	Tracheal, bronchus, and lung cancer	Rate	2000	219.938505	251.164728	187.260094
Deaths	China	Both	80-84	Tracheal, bronchus, and lung cancer	Rate	2000	346.477972	387.103278	307.312041
Deaths	China	Male	85-89	Tracheal, bronchus, and lung cancer	Number	2000	8374.51325	9363.16791	7460.44287
Deaths	China	Female	85-89	Tracheal, bronchus, and lung cancer	Number	2000	5328.51332	6245.18492	4394.79464
Deaths	China	Both	85-89	Tracheal, bronchus, and lung cancer	Number	2000	13703.0266	15218.2763	12115.4342
Deaths	China	Male	85-89	Tracheal, bronchus, and lung cancer	Rate	2000	839.41581	938.513193	747.79435
Deaths	China	Female	85-89	Tracheal, bronchus, and lung cancer	Rate	2000	277.19951	324.886529	228.625669
Deaths	China	Both	85-89	Tracheal, bronchus, and lung cancer	Rate	2000	469.293593	521.18702	414.922616
Deaths	China	Male	90-94	Tracheal, bronchus, and lung cancer	Number	2000	2017.13653	2298.04366	1768.69741
Deaths	China	Female	90-94	Tracheal, bronchus, and lung cancer	Number	2000	1594.4909	1873.6164	1286.65342
Deaths	China	Both	90-94	Tracheal, bronchus, and lung cancer	Number	2000	3611.62743	4098.17854	3117.75303
Deaths	China	Male	90-94	Tracheal, bronchus, and lung cancer	Rate	2000	1197.82439	1364.63382	1050.29524

Deaths	China	Female	90-94	Tracheal, bronchus, and lung cancer	Rate	2000	334.046193	392.523047	269.554174
Deaths	China	Both	90-94	Tracheal, bronchus, and lung cancer	Rate	2000	559.312259	634.661671	482.828732
Deaths	China	Male	95+ years	Tracheal, bronchus, and lung cancer	Number	2000	69.3922769	88.8325969	53.3932295
Deaths	China	Female	95+ years	Tracheal, bronchus, and lung cancer	Number	2000	268.343044	327.42282	203.816997
Deaths	China	Both	95+ years	Tracheal, bronchus, and lung cancer	Number	2000	337.735321	405.032841	259.935076
Deaths	China	Male	95+ years	Tracheal, bronchus, and lung cancer	Rate	2000	451.3092	577.743951	347.255585
Deaths	China	Female	95+ years	Tracheal, bronchus, and lung cancer	Rate	2000	383.351178	467.751733	291.170156
Deaths	China	Both	95+ years	Tracheal, bronchus, and lung cancer	Rate	2000	395.5902	474.41595	304.462584
Deaths	China	Male	15-19 years	Tracheal, bronchus, and lung cancer	Number	2010	157.62942	181.773127	135.747398
Deaths	China	Female	15-19 years	Tracheal, bronchus, and lung cancer	Number	2010	85.8730734	97.5958146	75.1424552
Deaths	China	Both	15-19 years	Tracheal, bronchus, and lung cancer	Number	2010	243.502493	272.740039	218.379887
Deaths	China	Male	15-19 years	Tracheal, bronchus, and lung cancer	Rate	2010	0.30292003	0.34931754	0.26086885
Deaths	China	Female	15-19 years	Tracheal, bronchus, and lung cancer	Rate	2010	0.17863671	0.20302284	0.15631443
Deaths	China	Both	15-19 years	Tracheal, bronchus, and lung cancer	Rate	2010	0.24323981	0.27244582	0.21814431
Deaths	China	Male	20-24 years	Tracheal, bronchus, and lung cancer	Number	2010	376.174968	437.77238	321.103473
Deaths	China	Female	20-24 years	Tracheal, bronchus, and lung cancer	Number	2010	222.481759	265.470772	184.650729
Deaths	China	Both	20-24 years	Tracheal, bronchus, and lung cancer	Number	2010	598.656727	680.050073	520.422678
Deaths	China	Male	20-24 years	Tracheal, bronchus, and lung cancer	Rate	2010	0.58571718	0.68162644	0.49996899
Deaths	China	Female	20-24 years	Tracheal, bronchus, and lung cancer	Rate	2010	0.34887389	0.41628501	0.28955101
Deaths	China	Both	20-24 years	Tracheal, bronchus, and lung cancer	Rate	2010	0.46771491	0.53130541	0.40659268
Deaths	China	Male	25-29 years	Tracheal, bronchus, and lung cancer	Number	2010	609.296547	689.7887	539.234454
Deaths	China	Female	25-29 years	Tracheal, bronchus, and lung cancer	Number	2010	392.104913	458.452056	336.254999

Deaths	China	Both	25-29 years	Tracheal, bronchus, and lung cancer	Number	2010	1001.40146	1110.75928	908.676507
Deaths	China	Male	25-29 years	Tracheal, bronchus, and lung cancer	Rate	2010	1.19275681	1.35032798	1.0556035
Deaths	China	Female	25-29 years	Tracheal, bronchus, and lung cancer	Rate	2010	0.77530014	0.90648683	0.66486937
Deaths	China	Both	25-29 years	Tracheal, bronchus, and lung cancer	Rate	2010	0.98507245	1.09264707	0.89385949
Deaths	China	Male	30-34 years	Tracheal, bronchus, and lung cancer	Number	2010	1591.56652	1793.06866	1410.6343
Deaths	China	Female	30-34 years	Tracheal, bronchus, and lung cancer	Number	2010	882.638062	1021.30006	755.78827
Deaths	China	Both	30-34 years	Tracheal, bronchus, and lung cancer	Number	2010	2474.20458	2739.17217	2235.13693
Deaths	China	Male	30-34 years	Tracheal, bronchus, and lung cancer	Rate	2010	3.20254774	3.60801004	2.8384762
Deaths	China	Female	30-34 years	Tracheal, bronchus, and lung cancer	Rate	2010	1.84092436	2.13013264	1.57635286
Deaths	China	Both	30-34 years	Tracheal, bronchus, and lung cancer	Rate	2010	2.53394849	2.80531417	2.28910814
Deaths	China	Male	35-39 years	Tracheal, bronchus, and lung cancer	Number	2010	3682.24923	4245.60374	3225.66859
Deaths	China	Female	35-39 years	Tracheal, bronchus, and lung cancer	Number	2010	2123.24744	2442.64746	1826.57956
Deaths	China	Both	35-39 years	Tracheal, bronchus, and lung cancer	Number	2010	5805.49668	6467.02299	5223.56749
Deaths	China	Male	35-39 years	Tracheal, bronchus, and lung cancer	Rate	2010	6.07406739	7.00335084	5.32091317
Deaths	China	Female	35-39 years	Tracheal, bronchus, and lung cancer	Rate	2010	3.6619256	4.2127889	3.15026799
Deaths	China	Both	35-39 years	Tracheal, bronchus, and lung cancer	Rate	2010	4.89484987	5.45260955	4.40420175
Deaths	China	Male	40-44 years	Tracheal, bronchus, and lung cancer	Number	2010	9309.84485	10730.3716	8020.22411
Deaths	China	Female	40-44 years	Tracheal, bronchus, and lung cancer	Number	2010	5064.83782	5824.34466	4371.66134
Deaths	China	Both	40-44 years	Tracheal, bronchus, and lung cancer	Number	2010	14374.6827	16081.3379	12905.6894
Deaths	China	Male	40-44 years	Tracheal, bronchus, and lung cancer	Rate	2010	14.5789304	16.8034316	12.559424
Deaths	China	Female	40-44 years	Tracheal, bronchus, and lung cancer	Rate	2010	8.23768325	9.47297982	7.1102694
Deaths	China	Both	40-44 years	Tracheal, bronchus, and lung cancer	Rate	2010	11.4683704	12.8299695	10.2963822

Deaths	China	Male	45-49 years	Tracheal, bronchus, and lung cancer	Number	2010	17177.1502	19960.6615	14924.8788
Deaths	China	Female	45-49 years	Tracheal, bronchus, and lung cancer	Number	2010	7768.56316	8910.45741	6716.64837
Deaths	China	Both	45-49 years	Tracheal, bronchus, and lung cancer	Number	2010	24945.7133	28105.0592	22373.7522
Deaths	China	Male	45-49 years	Tracheal, bronchus, and lung cancer	Rate	2010	31.7551951	36.9010396	27.5914476
Deaths	China	Female	45-49 years	Tracheal, bronchus, and lung cancer	Rate	2010	14.8849796	17.0729096	12.869455
Deaths	China	Both	45-49 years	Tracheal, bronchus, and lung cancer	Rate	2010	23.4710221	26.4436	21.051105
Deaths	China	Male	50-54 years	Tracheal, bronchus, and lung cancer	Number	2010	23894.1771	27753.1128	20723.6846
Deaths	China	Female	50-54 years	Tracheal, bronchus, and lung cancer	Number	2010	9519.64094	10978.276	8164.3929
Deaths	China	Both	50-54 years	Tracheal, bronchus, and lung cancer	Number	2010	33413.818	37527.3222	29918.5121
Deaths	China	Male	50-54 years	Tracheal, bronchus, and lung cancer	Rate	2010	58.7450759	68.2324698	50.9502554
Deaths	China	Female	50-54 years	Tracheal, bronchus, and lung cancer	Rate	2010	24.5941546	28.3625632	21.0928482
Deaths	China	Both	50-54 years	Tracheal, bronchus, and lung cancer	Rate	2010	42.0928217	47.2747796	37.6896347
Deaths	China	Male	55-59 years	Tracheal, bronchus, and lung cancer	Number	2010	43477.2278	50370.5229	37603.7137
Deaths	China	Female	55-59 years	Tracheal, bronchus, and lung cancer	Number	2010	16803.6185	19230.1993	14505.5859
Deaths	China	Both	55-59 years	Tracheal, bronchus, and lung cancer	Number	2010	60280.8463	67960.2328	53913.3277
Deaths	China	Male	55-59 years	Tracheal, bronchus, and lung cancer	Rate	2010	105.205552	121.885845	90.9929093
Deaths	China	Female	55-59 years	Tracheal, bronchus, and lung cancer	Rate	2010	41.5121094	47.5067995	35.8349881
Deaths	China	Both	55-59 years	Tracheal, bronchus, and lung cancer	Rate	2010	73.6886259	83.0760761	65.9048318
Deaths	China	Male	60-64 years	Tracheal, bronchus, and lung cancer	Number	2010	56965.9172	65478.9447	49328.6484
Deaths	China	Female	60-64 years	Tracheal, bronchus, and lung cancer	Number	2010	20393.381	23069.4809	17917.9727
Deaths	China	Both	60-64 years	Tracheal, bronchus, and lung cancer	Number	2010	77359.2982	86297.959	69670.3601
Deaths	China	Male	60-64 years	Tracheal, bronchus, and lung cancer	Rate	2010	189.85728	218.229688	164.40362

Deaths	China	Female	60-64 years	Tracheal, bronchus, and lung cancer	Rate	2010	70.3262499	79.5547377	61.7898437
Deaths	China	Both	60-64 years	Tracheal, bronchus, and lung cancer	Rate	2010	131.111124	146.260665	118.079655
Deaths	China	Male	65-69 years	Tracheal, bronchus, and lung cancer	Number	2010	58348.7901	66843.4269	51281.2149
Deaths	China	Female	65-69 years	Tracheal, bronchus, and lung cancer	Number	2010	21380.1311	24063.3006	18820.4584
Deaths	China	Both	65-69 years	Tracheal, bronchus, and lung cancer	Number	2010	79728.9211	89165.303	72001.0925
Deaths	China	Male	65-69 years	Tracheal, bronchus, and lung cancer	Rate	2010	279.719453	320.442066	245.838061
Deaths	China	Female	65-69 years	Tracheal, bronchus, and lung cancer	Rate	2010	104.429188	117.534871	91.9267137
Deaths	China	Both	65-69 years	Tracheal, bronchus, and lung cancer	Rate	2010	192.89372	215.723815	174.197247
Deaths	China	Male	70-74 years	Tracheal, bronchus, and lung cancer	Number	2010	69382.6624	78364.736	61494.2554
Deaths	China	Female	70-74 years	Tracheal, bronchus, and lung cancer	Number	2010	27127.7053	30298.8423	23980.1133
Deaths	China	Both	70-74 years	Tracheal, bronchus, and lung cancer	Number	2010	96510.3677	106114.328	87999.003
Deaths	China	Male	70-74 years	Tracheal, bronchus, and lung cancer	Rate	2010	419.580996	473.898706	371.877065
Deaths	China	Female	70-74 years	Tracheal, bronchus, and lung cancer	Rate	2010	162.477926	181.471046	143.625826
Deaths	China	Both	70-74 years	Tracheal, bronchus, and lung cancer	Rate	2010	290.4103	319.309671	264.798668
Deaths	China	Male	75-79 years	Tracheal, bronchus, and lung cancer	Number	2010	64641.9309	72824.2365	57395.8095
Deaths	China	Female	75-79 years	Tracheal, bronchus, and lung cancer	Number	2010	28273.6101	31747.9787	24780.448
Deaths	China	Both	75-79 years	Tracheal, bronchus, and lung cancer	Number	2010	92915.541	101660.135	84356.1741
Deaths	China	Male	75-79 years	Tracheal, bronchus, and lung cancer	Rate	2010	571.163003	643.460198	507.137743
Deaths	China	Female	75-79 years	Tracheal, bronchus, and lung cancer	Rate	2010	223.370209	250.818788	195.773155
Deaths	China	Both	75-79 years	Tracheal, bronchus, and lung cancer	Rate	2010	387.546402	424.019696	351.845681
Deaths	China	Male	80-84	Tracheal, bronchus, and lung cancer	Number	2010	38453.5414	42800.5364	33665.124
Deaths	China	Female	80-84	Tracheal, bronchus, and lung cancer	Number	2010	19567.053	21971.1537	16822.7751

Deaths	China	Both	80-84	Tracheal, bronchus, and lung cancer	Number	2010	58020.5944	63662.7582	51675.229
Deaths	China	Male	80-84	Tracheal, bronchus, and lung cancer	Rate	2010	721.455176	803.012347	631.616155
Deaths	China	Female	80-84	Tracheal, bronchus, and lung cancer	Rate	2010	269.058517	302.116319	231.323078
Deaths	China	Both	80-84	Tracheal, bronchus, and lung cancer	Rate	2010	460.39275	505.163255	410.042349
Deaths	China	Male	85-89	Tracheal, bronchus, and lung cancer	Number	2010	16815.1949	18650.1483	14918.666
Deaths	China	Female	85-89	Tracheal, bronchus, and lung cancer	Number	2010	9424.63974	10807.3077	7746.78233
Deaths	China	Both	85-89	Tracheal, bronchus, and lung cancer	Number	2010	26239.8347	28893.9726	22999.2499
Deaths	China	Male	85-89	Tracheal, bronchus, and lung cancer	Rate	2010	998.373501	1107.32072	885.770332
Deaths	China	Female	85-89	Tracheal, bronchus, and lung cancer	Rate	2010	298.606051	342.413882	245.445571
Deaths	China	Both	85-89	Tracheal, bronchus, and lung cancer	Rate	2010	542.092608	596.924834	475.14489
Deaths	China	Male	90-94	Tracheal, bronchus, and lung cancer	Number	2010	4115.48927	4586.77752	3518.73373
Deaths	China	Female	90-94	Tracheal, bronchus, and lung cancer	Number	2010	2889.28371	3412.16334	2270.33311
Deaths	China	Both	90-94	Tracheal, bronchus, and lung cancer	Number	2010	7004.77299	7825.97161	5953.19467
Deaths	China	Male	90-94	Tracheal, bronchus, and lung cancer	Rate	2010	1282.10031	1428.92096	1096.19277
Deaths	China	Female	90-94	Tracheal, bronchus, and lung cancer	Rate	2010	309.538088	365.555833	243.227955
Deaths	China	Both	90-94	Tracheal, bronchus, and lung cancer	Rate	2010	558.410105	623.87484	474.579842
Deaths	China	Male	95+ years	Tracheal, bronchus, and lung cancer	Number	2010	195.952698	222.604653	154.073224
Deaths	China	Female	95+ years	Tracheal, bronchus, and lung cancer	Number	2010	574.807843	680.759085	437.498209
Deaths	China	Both	95+ years	Tracheal, bronchus, and lung cancer	Number	2010	770.760541	894.58431	604.243541
Deaths	China	Male	95+ years	Tracheal, bronchus, and lung cancer	Rate	2010	660.400305	750.222792	519.25799
Deaths	China	Female	95+ years	Tracheal, bronchus, and lung cancer	Rate	2010	351.668987	416.490243	267.662584
Deaths	China	Both	95+ years	Tracheal, bronchus, and lung cancer	Rate	2010	399.10304	463.219507	312.879839

**Deaths**	**China**	**Male**	**15-19 years**	**Tracheal, bronchus, and lung cancer**	**Number**	**2019**	**110.749976**	**145.357501**	**81.2908511**
**Deaths**	**China**	**Female**	**15-19 years**	**Tracheal, bronchus, and lung cancer**	**Number**	**2019**	**64.2011129**	**78.0846483**	**52.7229825**
**Deaths**	**China**	**Both**	**15-19 years**	**Tracheal, bronchus, and lung cancer**	**Number**	**2019**	**174.951089**	**212.722646**	**142.338182**
**Deaths**	**China**	**Male**	**15-19 years**	**Tracheal, bronchus, and lung cancer**	**Rate**	**2019**	**0.28508221**	**0.37416565**	**0.20925129**
**Deaths**	**China**	**Female**	**15-19 years**	**Tracheal, bronchus, and lung cancer**	**Rate**	**2019**	**0.19152299**	**0.23293996**	**0.15728174**
**Deaths**	**China**	**Both**	**15-19 years**	**Tracheal, bronchus, and lung cancer**	**Rate**	**2019**	**0.241746**	**0.29393843**	**0.19668175**
**Deaths**	**China**	**Male**	**20-24 years**	**Tracheal, bronchus, and lung cancer**	**Number**	**2019**	**262.537662**	**346.21327**	**199.534995**
**Deaths**	**China**	**Female**	**20-24 years**	**Tracheal, bronchus, and lung cancer**	**Number**	**2019**	**138.559613**	**177.274007**	**104.519841**
**Deaths**	**China**	**Both**	**20-24 years**	**Tracheal, bronchus, and lung cancer**	**Number**	**2019**	**401.097275**	**490.653836**	**321.275351**
**Deaths**	**China**	**Male**	**20-24 years**	**Tracheal, bronchus, and lung cancer**	**Rate**	**2019**	**0.63696214**	**0.83997376**	**0.48410669**
**Deaths**	**China**	**Female**	**20-24 years**	**Tracheal, bronchus, and lung cancer**	**Rate**	**2019**	**0.37510663**	**0.47991369**	**0.28295464**
**Deaths**	**China**	**Both**	**20-24 years**	**Tracheal, bronchus, and lung cancer**	**Rate**	**2019**	**0.51320166**	**0.62778877**	**0.41106997**
**Deaths**	**China**	**Male**	**25-29 years**	**Tracheal, bronchus, and lung cancer**	**Number**	**2019**	**645.663881**	**800.527927**	**506.239269**
**Deaths**	**China**	**Female**	**25-29 years**	**Tracheal, bronchus, and lung cancer**	**Number**	**2019**	**339.525915**	**442.013878**	**253.776629**
**Deaths**	**China**	**Both**	**25-29 years**	**Tracheal, bronchus, and lung cancer**	**Number**	**2019**	**985.189796**	**1183.0478**	**799.371577**
**Deaths**	**China**	**Male**	**25-29 years**	**Tracheal, bronchus, and lung cancer**	**Rate**	**2019**	**1.23898725**	**1.53616134**	**0.97144043**
**Deaths**	**China**	**Female**	**25-29 years**	**Tracheal, bronchus, and lung cancer**	**Rate**	**2019**	**0.70423967**	**0.9168187**	**0.52637976**
**Deaths**	**China**	**Both**	**25-29 years**	**Tracheal, bronchus, and lung cancer**	**Rate**	**2019**	**0.98200878**	**1.17922793**	**0.79679054**
**Deaths**	**China**	**Male**	**30-34 years**	**Tracheal, bronchus, and lung cancer**	**Number**	**2019**	**1910.79944**	**2368.42044**	**1506.84717**
**Deaths**	**China**	**Female**	**30-34 years**	**Tracheal, bronchus, and lung cancer**	**Number**	**2019**	**991.0084**	**1278.40368**	**755.135418**
**Deaths**	**China**	**Both**	**30-34 years**	**Tracheal, bronchus, and lung cancer**	**Number**	**2019**	**2901.80784**	**3456.90768**	**2386.72712**
**Deaths**	**China**	**Male**	**30-34 years**	**Tracheal, bronchus, and lung cancer**	**Rate**	**2019**	**3.00282266**	**3.72197438**	**2.36801138**

Deaths	China	Female	30-34 years	Tracheal, bronchus, and lung cancer	Rate	2019	1.62536729	2.09672848	1.23850858
Deaths	China	Both	30-34 years	Tracheal, bronchus, and lung cancer	Rate	2019	2.32880915	2.7742975	1.91543764
Deaths	China	Male	35-39 years	Tracheal, bronchus, and lung cancer	Number	2019	3096.0936	3918.67754	2415.20579
Deaths	China	Female	35-39 years	Tracheal, bronchus, and lung cancer	Number	2019	1647.39913	2119.42351	1263.12801
Deaths	China	Both	35-39 years	Tracheal, bronchus, and lung cancer	Number	2019	4743.49273	5708.24323	3843.55388
Deaths	China	Male	35-39 years	Tracheal, bronchus, and lung cancer	Rate	2019	6.31387532	7.99137385	4.92533825
Deaths	China	Female	35-39 years	Tracheal, bronchus, and lung cancer	Rate	2019	3.55020459	4.56743417	2.72208646
Deaths	China	Both	35-39 years	Tracheal, bronchus, and lung cancer	Rate	2019	4.97016825	5.98102093	4.02722436
Deaths	China	Male	40-44 years	Tracheal, bronchus, and lung cancer	Number	2019	6821.28625	8845.86306	5159.41509
Deaths	China	Female	40-44 years	Tracheal, bronchus, and lung cancer	Number	2019	3703.58156	4762.28632	2801.58092
Deaths	China	Both	40-44 years	Tracheal, bronchus, and lung cancer	Number	2019	10524.8678	12785.327	8449.01111
Deaths	China	Male	40-44 years	Tracheal, bronchus, and lung cancer	Rate	2019	13.8126329	17.912261	10.4474588
Deaths	China	Female	40-44 years	Tracheal, bronchus, and lung cancer	Rate	2019	7.82285711	10.0590968	5.91761431
Deaths	China	Both	40-44 years	Tracheal, bronchus, and lung cancer	Rate	2019	10.8809487	13.2178845	8.7348609
Deaths	China	Male	45-49 years	Tracheal, bronchus, and lung cancer	Number	2019	15156.1008	19865.7072	11175.987
Deaths	China	Female	45-49 years	Tracheal, bronchus, and lung cancer	Number	2019	7299.53742	9334.3746	5533.63541
Deaths	China	Both	45-49 years	Tracheal, bronchus, and lung cancer	Number	2019	22455.6383	27618.4278	17984.4385
Deaths	China	Male	45-49 years	Tracheal, bronchus, and lung cancer	Rate	2019	25.1643714	32.9839475	18.5560054
Deaths	China	Female	45-49 years	Tracheal, bronchus, and lung cancer	Rate	2019	12.6132448	16.1293442	9.56185218
Deaths	China	Both	45-49 years	Tracheal, bronchus, and lung cancer	Rate	2019	19.0140217	23.3855471	15.2280911
Deaths	China	Male	50-54 years	Tracheal, bronchus, and lung cancer	Number	2019	33145.7117	43648.3456	24546.1431
Deaths	China	Female	50-54 years	Tracheal, bronchus, and lung cancer	Number	2019	14736.704	18845.6575	11212.5494

**Deaths**	**China**	**Both**	**50-54 years**	**Tracheal, bronchus, and lung cancer**	**Number**	**2019**	**47882.4157**	**59138.067**	**38399.0923**
**Deaths**	**China**	**Male**	**50-54 years**	**Tracheal, bronchus, and lung cancer**	**Rate**	**2019**	**53.9002707**	**70.9792465**	**39.9159858**
**Deaths**	**China**	**Female**	**50-54 years**	**Tracheal, bronchus, and lung cancer**	**Rate**	**2019**	**24.2250535**	**30.9795909**	**18.4318425**
**Deaths**	**China**	**Both**	**50-54 years**	**Tracheal, bronchus, and lung cancer**	**Rate**	**2019**	**39.1429621**	**48.3442425**	**31.390526**
**Deaths**	**China**	**Male**	**55-59 years**	**Tracheal, bronchus, and lung cancer**	**Number**	**2019**	**44519.5016**	**58591.6593**	**33154.6042**
**Deaths**	**China**	**Female**	**55-59 years**	**Tracheal, bronchus, and lung cancer**	**Number**	**2019**	**18434.8515**	**23369.6059**	**14115.1812**
**Deaths**	**China**	**Both**	**55-59 years**	**Tracheal, bronchus, and lung cancer**	**Number**	**2019**	**62954.3531**	**78277.3981**	**50209.9933**
**Deaths**	**China**	**Male**	**55-59 years**	**Tracheal, bronchus, and lung cancer**	**Rate**	**2019**	**95.204072**	**125.297103**	**70.9004641**
**Deaths**	**China**	**Female**	**55-59 years**	**Tracheal, bronchus, and lung cancer**	**Rate**	**2019**	**39.7790642**	**50.4273685**	**30.4579994**
**Deaths**	**China**	**Both**	**55-59 years**	**Tracheal, bronchus, and lung cancer**	**Rate**	**2019**	**67.6163066**	**84.0740678**	**53.9281898**
**Deaths**	**China**	**Male**	**60-64 years**	**Tracheal, bronchus, and lung cancer**	**Number**	**2019**	**64825.8261**	**85171.0194**	**49321.7491**
**Deaths**	**China**	**Female**	**60-64 years**	**Tracheal, bronchus, and lung cancer**	**Number**	**2019**	**26772.7426**	**33484.6628**	**21117.7127**
**Deaths**	**China**	**Both**	**60-64 years**	**Tracheal, bronchus, and lung cancer**	**Number**	**2019**	**91598.5687**	**112361.089**	**74812.1743**
**Deaths**	**China**	**Male**	**60-64 years**	**Tracheal, bronchus, and lung cancer**	**Rate**	**2019**	**167.320442**	**219.83295**	**127.303227**
**Deaths**	**China**	**Female**	**60-64 years**	**Tracheal, bronchus, and lung cancer**	**Rate**	**2019**	**69.7414325**	**87.225593**	**55.0104095**
**Deaths**	**China**	**Both**	**60-64 years**	**Tracheal, bronchus, and lung cancer**	**Rate**	**2019**	**118.755454**	**145.673588**	**96.992277**
**Deaths**	**China**	**Male**	**65-69 years**	**Tracheal, bronchus, and lung cancer**	**Number**	**2019**	**86023.4991**	**112021.794**	**65454.9084**
**Deaths**	**China**	**Female**	**65-69 years**	**Tracheal, bronchus, and lung cancer**	**Number**	**2019**	**37567.9496**	**46790.9561**	**29959.4296**
**Deaths**	**China**	**Both**	**65-69 years**	**Tracheal, bronchus, and lung cancer**	**Number**	**2019**	**123591.449**	**150752.913**	**101427.945**
**Deaths**	**China**	**Male**	**65-69 years**	**Tracheal, bronchus, and lung cancer**	**Rate**	**2019**	**251.583236**	**327.61752**	**191.428596**
**Deaths**	**China**	**Female**	**65-69 years**	**Tracheal, bronchus, and lung cancer**	**Rate**	**2019**	**105.879043**	**131.872559**	**84.4356893**
**Deaths**	**China**	**Both**	**65-69 years**	**Tracheal, bronchus, and lung cancer**	**Rate**	**2019**	**177.383262**	**216.366454**	**145.573338**

**Deaths**	**China**	**Male**	**70-74 years**	**Tracheal, bronchus, and lung cancer**	**Number**	**2019**	**92342.2736**	**117702.621**	**71036.1408**
**Deaths**	**China**	**Female**	**70-74 years**	**Tracheal, bronchus, and lung cancer**	**Number**	**2019**	**39673.1243**	**48161.1671**	**32047.7727**
**Deaths**	**China**	**Both**	**70-74 years**	**Tracheal, bronchus, and lung cancer**	**Number**	**2019**	**132015.398**	**158856.494**	**108122.234**
**Deaths**	**China**	**Male**	**70-74 years**	**Tracheal, bronchus, and lung cancer**	**Rate**	**2019**	**395.495495**	**504.112087**	**304.242819**
**Deaths**	**China**	**Female**	**70-74 years**	**Tracheal, bronchus, and lung cancer**	**Rate**	**2019**	**162.535647**	**197.310058**	**131.29557**
**Deaths**	**China**	**Both**	**70-74 years**	**Tracheal, bronchus, and lung cancer**	**Rate**	**2019**	**276.429326**	**332.632362**	**226.399017**
**Deaths**	**China**	**Male**	**75-79 years**	**Tracheal, bronchus, and lung cancer**	**Number**	**2019**	**75392.9221**	**95522.1413**	**58911.7683**
**Deaths**	**China**	**Female**	**75-79 years**	**Tracheal, bronchus, and lung cancer**	**Number**	**2019**	**35670.7842**	**43608.2778**	**28469.2683**
**Deaths**	**China**	**Both**	**75-79 years**	**Tracheal, bronchus, and lung cancer**	**Number**	**2019**	**111063.706**	**131687.921**	**92311.0748**
**Deaths**	**China**	**Male**	**75-79 years**	**Tracheal, bronchus, and lung cancer**	**Rate**	**2019**	**524.500227**	**664.536981**	**409.842662**
**Deaths**	**China**	**Female**	**75-79 years**	**Tracheal, bronchus, and lung cancer**	**Rate**	**2019**	**223.299305**	**272.988058**	**178.217776**
**Deaths**	**China**	**Both**	**75-79 years**	**Tracheal, bronchus, and lung cancer**	**Rate**	**2019**	**365.959092**	**433.916656**	**304.168466**
**Deaths**	**China**	**Male**	**80-84**	**Tracheal, bronchus, and lung cancer**	**Number**	**2019**	**52901.5192**	**65047.944**	**41678.2375**
**Deaths**	**China**	**Female**	**80-84**	**Tracheal, bronchus, and lung cancer**	**Number**	**2019**	**29457.6728**	**35972.7323**	**23416.0964**
**Deaths**	**China**	**Both**	**80-84**	**Tracheal, bronchus, and lung cancer**	**Number**	**2019**	**82359.192**	**96956.8426**	**68715.0693**
**Deaths**	**China**	**Male**	**80-84**	**Tracheal, bronchus, and lung cancer**	**Rate**	**2019**	**642.712329**	**790.281948**	**506.358182**
**Deaths**	**China**	**Female**	**80-84**	**Tracheal, bronchus, and lung cancer**	**Rate**	**2019**	**277.700889**	**339.119109**	**220.74625**
**Deaths**	**China**	**Both**	**80-84**	**Tracheal, bronchus, and lung cancer**	**Rate**	**2019**	**437.181383**	**514.669043**	**364.755267**
**Deaths**	**China**	**Male**	**85-89**	**Tracheal, bronchus, and lung cancer**	**Number**	**2019**	**29274.7602**	**35028.8197**	**23201.3296**
**Deaths**	**China**	**Female**	**85-89**	**Tracheal, bronchus, and lung cancer**	**Number**	**2019**	**17537.7978**	**22251.2141**	**13229.0158**
**Deaths**	**China**	**Both**	**85-89**	**Tracheal, bronchus, and lung cancer**	**Number**	**2019**	**46812.558**	**54720.7855**	**39658.6844**
**Deaths**	**China**	**Male**	**85-89**	**Tracheal, bronchus, and lung cancer**	**Rate**	**2019**	**944.452969**	**1130.0886**	**748.513892**

Deaths	China	Female	85-89	Tracheal, bronchus, and lung cancer	Rate	2019	323.728208	410.732622	244.192893
Deaths	China	Both	85-89	Tracheal, bronchus, and lung cancer	Rate	2019	549.630406	642.481608	465.636141
Deaths	China	Male	90-94	Tracheal, bronchus, and lung cancer	Number	2019	7643.32003	9325.68962	6116.41897
Deaths	China	Female	90-94	Tracheal, bronchus, and lung cancer	Number	2019	6459.08489	7848.30021	4955.64379
Deaths	China	Both	90-94	Tracheal, bronchus, and lung cancer	Number	2019	14102.4049	16440.3776	11607.478
Deaths	China	Male	90-94	Tracheal, bronchus, and lung cancer	Rate	2019	1079.35578	1316.93255	863.733576
Deaths	China	Female	90-94	Tracheal, bronchus, and lung cancer	Rate	2019	348.069835	422.932444	267.05178
Deaths	China	Both	90-94	Tracheal, bronchus, and lung cancer	Rate	2019	550.053612	641.244465	452.740877
Deaths	China	Male	95+ years	Tracheal, bronchus, and lung cancer	Number	2019	876.39588	1028.77286	700.127218
Deaths	China	Female	95+ years	Tracheal, bronchus, and lung cancer	Number	2019	1621.71464	2014.67072	1164.5621
Deaths	China	Both	95+ years	Tracheal, bronchus, and lung cancer	Number	2019	2498.11052	2994.98264	1910.72751
Deaths	China	Male	95+ years	Tracheal, bronchus, and lung cancer	Rate	2019	836.778468	982.267257	668.478018
Deaths	China	Female	95+ years	Tracheal, bronchus, and lung cancer	Rate	2019	372.34753	462.570694	267.384785
Deaths	China	Both	95+ years	Tracheal, bronchus, and lung cancer	Rate	2019	462.379795	554.346754	353.660011
Deaths	China	Male	15-19 years	Tracheal, bronchus, and lung cancer	Number	2021	111.099356	148.001446	77.6895433
Deaths	China	Female	15-19 years	Tracheal, bronchus, and lung cancer	Number	2021	67.404197	83.4879935	54.0753249
Deaths	China	Both	15-19 years	Tracheal, bronchus, and lung cancer	Number	2021	178.503552	218.935427	141.746267
Deaths	China	Male	15-19 years	Tracheal, bronchus, and lung cancer	Rate	2021	0.27717455	0.36923918	0.19382259
Deaths	China	Female	15-19 years	Tracheal, bronchus, and lung cancer	Rate	2021	0.19486984	0.24136912	0.15633522
Deaths	China	Both	15-19 years	Tracheal, bronchus, and lung cancer	Rate	2021	0.23904969	0.29319554	0.1898248
Deaths	China	Male	20-24 years	Tracheal, bronchus, and lung cancer	Number	2021	246.196128	325.42159	177.812066
Deaths	China	Female	20-24 years	Tracheal, bronchus, and lung cancer	Number	2021	131.270665	174.174758	95.5715745

**Deaths**	**China**	**Both**	**20-24 years**	**Tracheal, bronchus, and lung cancer**	**Number**	**2021**	**377.466792**	**477.244617**	**288.595282**
**Deaths**	**China**	**Male**	**20-24 years**	**Tracheal, bronchus, and lung cancer**	**Rate**	**2021**	**0.63342408**	**0.83725879**	**0.4574826**
**Deaths**	**China**	**Female**	**20-24 years**	**Tracheal, bronchus, and lung cancer**	**Rate**	**2021**	**0.38263908**	**0.50769964**	**0.27858028**
**Deaths**	**China**	**Both**	**20-24 years**	**Tracheal, bronchus, and lung cancer**	**Rate**	**2021**	**0.51584713**	**0.65220377**	**0.39439509**
**Deaths**	**China**	**Male**	**25-29 years**	**Tracheal, bronchus, and lung cancer**	**Number**	**2021**	**564.882344**	**715.883508**	**421.765725**
**Deaths**	**China**	**Female**	**25-29 years**	**Tracheal, bronchus, and lung cancer**	**Number**	**2021**	**296.862392**	**401.609715**	**213.760191**
**Deaths**	**China**	**Both**	**25-29 years**	**Tracheal, bronchus, and lung cancer**	**Number**	**2021**	**861.744736**	**1052.17259**	**692.812481**
**Deaths**	**China**	**Male**	**25-29 years**	**Tracheal, bronchus, and lung cancer**	**Rate**	**2021**	**1.23834972**	**1.56937839**	**0.92460575**
**Deaths**	**China**	**Female**	**25-29 years**	**Tracheal, bronchus, and lung cancer**	**Rate**	**2021**	**0.72642285**	**0.98273976**	**0.52307161**
**Deaths**	**China**	**Both**	**25-29 years**	**Tracheal, bronchus, and lung cancer**	**Rate**	**2021**	**0.99644322**	**1.21663667**	**0.80110533**
**Deaths**	**China**	**Male**	**30-34 years**	**Tracheal, bronchus, and lung cancer**	**Number**	**2021**	**1910.08799**	**2426.81247**	**1447.6089**
**Deaths**	**China**	**Female**	**30-34 years**	**Tracheal, bronchus, and lung cancer**	**Number**	**2021**	**990.564411**	**1307.98864**	**725.38443**
**Deaths**	**China**	**Both**	**30-34 years**	**Tracheal, bronchus, and lung cancer**	**Number**	**2021**	**2900.6524**	**3532.07698**	**2327.54727**
**Deaths**	**China**	**Male**	**30-34 years**	**Tracheal, bronchus, and lung cancer**	**Rate**	**2021**	**3.04717991**	**3.87151494**	**2.30938301**
**Deaths**	**China**	**Female**	**30-34 years**	**Tracheal, bronchus, and lung cancer**	**Rate**	**2021**	**1.69415621**	**2.23704491**	**1.24062052**
**Deaths**	**China**	**Both**	**30-34 years**	**Tracheal, bronchus, and lung cancer**	**Rate**	**2021**	**2.39420048**	**2.91537876**	**1.92115911**
**Deaths**	**China**	**Male**	**35-39 years**	**Tracheal, bronchus, and lung cancer**	**Number**	**2021**	**3426.551**	**4444.04542**	**2543.49002**
**Deaths**	**China**	**Female**	**35-39 years**	**Tracheal, bronchus, and lung cancer**	**Number**	**2021**	**1855.52221**	**2456.08614**	**1361.68664**
**Deaths**	**China**	**Both**	**35-39 years**	**Tracheal, bronchus, and lung cancer**	**Number**	**2021**	**5282.07321**	**6565.19252**	**4171.51375**
**Deaths**	**China**	**Male**	**35-39 years**	**Tracheal, bronchus, and lung cancer**	**Rate**	**2021**	**6.30273394**	**8.17429417**	**4.67844805**
**Deaths**	**China**	**Female**	**35-39 years**	**Tracheal, bronchus, and lung cancer**	**Rate**	**2021**	**3.59617191**	**4.7601198**	**2.63907337**
**Deaths**	**China**	**Both**	**35-39 years**	**Tracheal, bronchus, and lung cancer**	**Rate**	**2021**	**4.98481589**	**6.19572556**	**3.93675499**

Deaths	China	Male	40-44 years	Tracheal, bronchus, and lung cancer	Number	2021	6451.57456	8524.53178	4720.86839
Deaths	China	Female	40-44 years	Tracheal, bronchus, and lung cancer	Number	2021	3567.64402	4764.67439	2613.23358
Deaths	China	Both	40-44 years	Tracheal, bronchus, and lung cancer	Number	2021	10019.2186	12586.4398	7812.44667
Deaths	China	Male	40-44 years	Tracheal, bronchus, and lung cancer	Rate	2021	13.7486039	18.1661716	10.060389
Deaths	China	Female	40-44 years	Tracheal, bronchus, and lung cancer	Rate	2021	7.99769953	10.6811201	5.85816771
Deaths	China	Both	40-44 years	Tracheal, bronchus, and lung cancer	Rate	2021	10.945936	13.7506098	8.53505101
Deaths	China	Male	45-49 years	Tracheal, bronchus, and lung cancer	Number	2021	14090.2206	19065.6253	10052.619
Deaths	China	Female	45-49 years	Tracheal, bronchus, and lung cancer	Number	2021	7090.6489	9323.90509	5268.38403
Deaths	China	Both	45-49 years	Tracheal, bronchus, and lung cancer	Number	2021	21180.8695	26603.2212	16361.834
Deaths	China	Male	45-49 years	Tracheal, bronchus, and lung cancer	Rate	2021	25.1335145	34.0084222	17.9314188
Deaths	China	Female	45-49 years	Tracheal, bronchus, and lung cancer	Rate	2021	13.0678926	17.1837291	9.70950298
Deaths	China	Both	45-49 years	Tracheal, bronchus, and lung cancer	Rate	2021	19.1992112	24.1142538	14.8310392
Deaths	China	Male	50-54 years	Tracheal, bronchus, and lung cancer	Number	2021	32763.767	44677.9401	23492.9075
Deaths	China	Female	50-54 years	Tracheal, bronchus, and lung cancer	Number	2021	14995.106	19663.6744	11056.9889
Deaths	China	Both	50-54 years	Tracheal, bronchus, and lung cancer	Number	2021	47758.873	60436.7287	37323.9533
Deaths	China	Male	50-54 years	Tracheal, bronchus, and lung cancer	Rate	2021	53.5871959	73.0735733	38.4241238
Deaths	China	Female	50-54 years	Tracheal, bronchus, and lung cancer	Rate	2021	25.1098609	32.9275517	18.5153378
Deaths	China	Both	50-54 years	Tracheal, bronchus, and lung cancer	Rate	2021	39.5161798	50.0059673	30.8822206
Deaths	China	Male	55-59 years	Tracheal, bronchus, and lung cancer	Number	2021	51850.0826	69460.535	37152.6677
Deaths	China	Female	55-59 years	Tracheal, bronchus, and lung cancer	Number	2021	22435.774	29227.9312	16773.4463
Deaths	China	Both	55-59 years	Tracheal, bronchus, and lung cancer	Number	2021	74285.8566	94318.2431	56416.3469
Deaths	China	Male	55-59 years	Tracheal, bronchus, and lung cancer	Rate	2021	94.4728835	126.559818	67.6936172

Deaths	China	Female	55-59 years	Tracheal, bronchus, and lung cancer	Rate	2021	40.748 7422	53.084 9273	30.464 598
Deaths	China	Both	55-59 years	Tracheal, bronchus, and lung cancer	Rate	2021	67.567 9936	85.788 7993	51.314 4701
Deaths	China	Male	60-64 years	Tracheal, bronchus, and lung cancer	Numbe r	2021	59577. 723	79403. 3753	43328. 0291
Deaths	China	Female	60-64 years	Tracheal, bronchus, and lung cancer	Numbe r	2021	25758. 3259	32904. 3004	19619. 8871
Deaths	China	Both	60-64 years	Tracheal, bronchus, and lung cancer	Numbe r	2021	85336. 0488	106068 .15	67370. 7528
Deaths	China	Male	60-64 years	Tracheal, bronchus, and lung cancer	Rate	2021	162.66 8007	216.79 8967	118.30 0664
Deaths	China	Female	60-64 years	Tracheal, bronchus, and lung cancer	Rate	2021	70.803 8872	90.446 5759	53.930 6896
Deaths	China	Both	60-64 years	Tracheal, bronchus, and lung cancer	Rate	2021	116.89 0425	145.28 8554	92.282 1716
Deaths	China	Male	65-69 years	Tracheal, bronchus, and lung cancer	Numbe r	2021	94684. 291	126288 .216	68263. 354
Deaths	China	Female	65-69 years	Tracheal, bronchus, and lung cancer	Numbe r	2021	43354. 6123	55485. 7362	33494. 1674
Deaths	China	Both	65-69 years	Tracheal, bronchus, and lung cancer	Numbe r	2021	138038 .903	171595 .716	109566 .149
Deaths	China	Male	65-69 years	Tracheal, bronchus, and lung cancer	Rate	2021	250.88 5209	334.62 621	180.87 7584
Deaths	China	Female	65-69 years	Tracheal, bronchus, and lung cancer	Rate	2021	111.27 0018	142.40 4661	85.963 0941
Deaths	China	Both	65-69 years	Tracheal, bronchus, and lung cancer	Rate	2021	179.96 4254	223.71 2984	142.84 3719
Deaths	China	Male	70-74 years	Tracheal, bronchus, and lung cancer	Numbe r	2021	101679 .452	131845 .801	75667. 5474
Deaths	China	Female	70-74 years	Tracheal, bronchus, and lung cancer	Numbe r	2021	46520. 2353	57812. 8335	36374. 0365
Deaths	China	Both	70-74 years	Tracheal, bronchus, and lung cancer	Numbe r	2021	148199 .688	181356 .342	118367 .668
Deaths	China	Male	70-74 years	Tracheal, bronchus, and lung cancer	Rate	2021	393.24 5347	509.91 3717	292.64 4288
Deaths	China	Female	70-74 years	Tracheal, bronchus, and lung cancer	Rate	2021	169.53 4694	210.68 8553	132.55 8684
Deaths	China	Both	70-74 years	Tracheal, bronchus, and lung cancer	Rate	2021	278.06 6756	340.27 8515	222.09 3001
Deaths	China	Male	75-79 years	Tracheal, bronchus, and lung cancer	Numbe r	2021	80976. 7969	105058 .62	60375. 6732
Deaths	China	Female	75-79 years	Tracheal, bronchus, and lung cancer	Numbe r	2021	40153. 6411	49963. 0489	31178. 2506

Deaths	China	Both	75-79 years	Tracheal, bronchus, and lung cancer	Number	2021	121130.438	147265.029	97989.9471
Deaths	China	Male	75-79 years	Tracheal, bronchus, and lung cancer	Rate	2021	519.001153	673.34776	386.963244
Deaths	China	Female	75-79 years	Tracheal, bronchus, and lung cancer	Rate	2021	229.233166	285.234105	177.993549
Deaths	China	Both	75-79 years	Tracheal, bronchus, and lung cancer	Rate	2021	365.743738	444.655062	295.872864
Deaths	China	Male	80-84	Tracheal, bronchus, and lung cancer	Number	2021	54713.3262	69292.9808	41346.7366
Deaths	China	Female	80-84	Tracheal, bronchus, and lung cancer	Number	2021	31602.5469	38695.5977	24328.7026
Deaths	China	Both	80-84	Tracheal, bronchus, and lung cancer	Number	2021	86315.8731	103918.591	70908.8789
Deaths	China	Male	80-84	Tracheal, bronchus, and lung cancer	Rate	2021	630.702971	798.768635	476.620806
Deaths	China	Female	80-84	Tracheal, bronchus, and lung cancer	Rate	2021	284.275306	348.079632	218.844684
Deaths	China	Both	80-84	Tracheal, bronchus, and lung cancer	Rate	2021	436.118135	525.057332	358.273014
Deaths	China	Male	85-89	Tracheal, bronchus, and lung cancer	Number	2021	32922.9351	40365.1356	25039.7046
Deaths	China	Female	85-89	Tracheal, bronchus, and lung cancer	Number	2021	19946.7392	25126.5935	14501.35
Deaths	China	Both	85-89	Tracheal, bronchus, and lung cancer	Number	2021	52869.6743	62327.136	43174.5047
Deaths	China	Male	85-89	Tracheal, bronchus, and lung cancer	Rate	2021	945.812575	1159.61268	719.342531
Deaths	China	Female	85-89	Tracheal, bronchus, and lung cancer	Rate	2021	329.979841	415.670413	239.896511
Deaths	China	Both	85-89	Tracheal, bronchus, and lung cancer	Rate	2021	555.018434	654.301542	453.239902
Deaths	China	Male	90-94	Tracheal, bronchus, and lung cancer	Number	2021	8740.06353	10909.5612	6610.75307
Deaths	China	Female	90-94	Tracheal, bronchus, and lung cancer	Number	2021	7586.05655	9372.47976	5652.06922
Deaths	China	Both	90-94	Tracheal, bronchus, and lung cancer	Number	2021	16326.1201	19588.3257	12994.6504
Deaths	China	Male	90-94	Tracheal, bronchus, and lung cancer	Rate	2021	1080.57478	1348.79988	817.318206
Deaths	China	Female	90-94	Tracheal, bronchus, and lung cancer	Rate	2021	357.301485	441.4416	266.211135
Deaths	China	Both	90-94	Tracheal, bronchus, and lung cancer	Rate	2021	556.827753	668.090357	443.202791

Deaths	China	Male	95+ years	Tracheal, bronchus, and lung cancer	Number	2021	1017.61858	1220.16873	779.88282
Deaths	China	Female	95+ years	Tracheal, bronchus, and lung cancer	Number	2021	2040.97889	2551.69456	1433.31309
Deaths	China	Both	95+ years	Tracheal, bronchus, and lung cancer	Number	2021	3058.59746	3712.19299	2267.92198
Deaths	China	Male	95+ years	Tracheal, bronchus, and lung cancer	Rate	2021	841.398679	1008.87344	644.831365
Deaths	China	Female	95+ years	Tracheal, bronchus, and lung cancer	Rate	2021	393.893683	492.457994	276.618721
Deaths	China	Both	95+ years	Tracheal, bronchus, and lung cancer	Rate	2021	478.580015	580.848379	354.862693
Deaths	China	Male	15-19 years	Tracheal, bronchus, and lung cancer	Number	2020	110.495098	145.998276	79.3234994
Deaths	China	Female	15-19 years	Tracheal, bronchus, and lung cancer	Number	2020	65.3596053	80.5076636	53.1041887
Deaths	China	Both	15-19 years	Tracheal, bronchus, and lung cancer	Number	2020	175.854703	214.954933	142.098721
Deaths	China	Male	15-19 years	Tracheal, bronchus, and lung cancer	Rate	2020	0.28157465	0.37204739	0.20214007
Deaths	China	Female	15-19 years	Tracheal, bronchus, and lung cancer	Rate	2020	0.19327059	0.23806392	0.15703091
Deaths	China	Both	15-19 years	Tracheal, bronchus, and lung cancer	Rate	2020	0.24070061	0.29421894	0.19449721
Deaths	China	Male	20-24 years	Tracheal, bronchus, and lung cancer	Number	2020	252.958973	329.848094	190.170838
Deaths	China	Female	20-24 years	Tracheal, bronchus, and lung cancer	Number	2020	132.900812	175.552391	99.4581771
Deaths	China	Both	20-24 years	Tracheal, bronchus, and lung cancer	Number	2020	385.859785	478.381594	303.80363
Deaths	China	Male	20-24 years	Tracheal, bronchus, and lung cancer	Rate	2020	0.63470998	0.8276357	0.47716563
Deaths	China	Female	20-24 years	Tracheal, bronchus, and lung cancer	Rate	2020	0.37495053	0.49528262	0.28059946
Deaths	China	Both	20-24 years	Tracheal, bronchus, and lung cancer	Rate	2020	0.51243575	0.63530806	0.40346221
Deaths	China	Male	25-29 years	Tracheal, bronchus, and lung cancer	Number	2020	600.952836	751.049023	462.278115
Deaths	China	Female	25-29 years	Tracheal, bronchus, and lung cancer	Number	2020	316.938596	419.154409	233.098604
Deaths	China	Both	25-29 years	Tracheal, bronchus, and lung cancer	Number	2020	917.891432	1105.97068	744.03869
Deaths	China	Male	25-29 years	Tracheal, bronchus, and lung cancer	Rate	2020	1.23941223	1.54897237	0.95340784

Deaths	China	Female	25-29 years	Tracheal, bronchus, and lung cancer	Rate	2020	0.71872381	0.9505193	0.52859929
Deaths	China	Both	25-29 years	Tracheal, bronchus, and lung cancer	Rate	2020	0.99141118	1.19455488	0.80363347
Deaths	China	Male	30-34 years	Tracheal, bronchus, and lung cancer	Number	2020	1938.64325	2414.45604	1531.54778
Deaths	China	Female	30-34 years	Tracheal, bronchus, and lung cancer	Number	2020	1011.08946	1337.23891	749.194812
Deaths	China	Both	30-34 years	Tracheal, bronchus, and lung cancer	Number	2020	2949.73271	3555.3312	2418.8491
Deaths	China	Male	30-34 years	Tracheal, bronchus, and lung cancer	Rate	2020	3.02402709	3.76623211	2.389012
Deaths	China	Female	30-34 years	Tracheal, bronchus, and lung cancer	Rate	2020	1.66689627	2.20459083	1.23513308
Deaths	China	Both	30-34 years	Tracheal, bronchus, and lung cancer	Rate	2020	2.36423073	2.84962202	1.93872392
Deaths	China	Male	35-39 years	Tracheal, bronchus, and lung cancer	Number	2020	3221.10472	4126.61228	2475.14745
Deaths	China	Female	35-39 years	Tracheal, bronchus, and lung cancer	Number	2020	1725.944	2254.29287	1298.43649
Deaths	China	Both	35-39 years	Tracheal, bronchus, and lung cancer	Number	2020	4947.04871	5979.41875	3984.85528
Deaths	China	Male	35-39 years	Tracheal, bronchus, and lung cancer	Rate	2020	6.28893867	8.05686678	4.83251924
Deaths	China	Female	35-39 years	Tracheal, bronchus, and lung cancer	Rate	2020	3.55724777	4.64619844	2.67613569
Deaths	China	Both	35-39 years	Tracheal, bronchus, and lung cancer	Rate	2020	4.96006108	5.99514659	3.99533676
Deaths	China	Male	40-44 years	Tracheal, bronchus, and lung cancer	Number	2020	6594.60494	8537.28018	5046.62723
Deaths	China	Female	40-44 years	Tracheal, bronchus, and lung cancer	Number	2020	3606.33737	4655.89788	2705.78116
Deaths	China	Both	40-44 years	Tracheal, bronchus, and lung cancer	Number	2020	10200.9423	12443.5153	8128.81255
Deaths	China	Male	40-44 years	Tracheal, bronchus, and lung cancer	Rate	2020	13.7646026	17.8194555	10.533583
Deaths	China	Female	40-44 years	Tracheal, bronchus, and lung cancer	Rate	2020	7.88701107	10.1823857	5.91750683
Deaths	China	Both	40-44 years	Tracheal, bronchus, and lung cancer	Rate	2020	10.8943803	13.2893985	8.68139162
Deaths	China	Male	45-49 years	Tracheal, bronchus, and lung cancer	Number	2020	14712.3137	19835.8	10752.5045
Deaths	China	Female	45-49 years	Tracheal, bronchus, and lung cancer	Number	2020	7261.04912	9482.96751	5470.78472

Deaths	China	Both	45-49 years	Tracheal, bronchus, and lung cancer	Number	2020	21973.3629	27202.6285	17211.6646
Deaths	China	Male	45-49 years	Tracheal, bronchus, and lung cancer	Rate	2020	25.1680521	33.9326947	18.394088
Deaths	China	Female	45-49 years	Tracheal, bronchus, and lung cancer	Rate	2020	12.8728239	16.8119742	9.69893565
Deaths	China	Both	45-49 years	Tracheal, bronchus, and lung cancer	Rate	2020	19.130172	23.6828093	14.9846023
Deaths	China	Male	50-54 years	Tracheal, bronchus, and lung cancer	Number	2020	33031.4281	44298.2714	24130.7021
Deaths	China	Female	50-54 years	Tracheal, bronchus, and lung cancer	Number	2020	15006.3144	19548.8153	11314.1434
Deaths	China	Both	50-54 years	Tracheal, bronchus, and lung cancer	Number	2020	48037.7425	60064.9082	37688.6124
Deaths	China	Male	50-54 years	Tracheal, bronchus, and lung cancer	Rate	2020	53.8061962	72.1592017	39.3074525
Deaths	China	Female	50-54 years	Tracheal, bronchus, and lung cancer	Rate	2020	24.8291198	32.3450425	18.7201343
Deaths	China	Both	50-54 years	Tracheal, bronchus, and lung cancer	Rate	2020	39.4307889	49.3030394	30.9359191
Deaths	China	Male	55-59 years	Tracheal, bronchus, and lung cancer	Number	2020	48383.1202	65124.3566	35484.9555
Deaths	China	Female	55-59 years	Tracheal, bronchus, and lung cancer	Number	2020	20425.7727	26301.1687	15442.2479
Deaths	China	Both	55-59 years	Tracheal, bronchus, and lung cancer	Number	2020	68808.8929	87058.3555	53614.8077
Deaths	China	Male	55-59 years	Tracheal, bronchus, and lung cancer	Rate	2020	94.6296718	127.372862	69.4029174
Deaths	China	Female	55-59 years	Tracheal, bronchus, and lung cancer	Rate	2020	40.087293	51.6182507	30.3067074
Deaths	China	Both	55-59 years	Tracheal, bronchus, and lung cancer	Rate	2020	67.4054139	85.2826464	52.521239
Deaths	China	Male	60-64 years	Tracheal, bronchus, and lung cancer	Number	2020	61628.6238	82057.0056	45496.3995
Deaths	China	Female	60-64 years	Tracheal, bronchus, and lung cancer	Number	2020	26155.2106	33280.3933	20320.3976
Deaths	China	Both	60-64 years	Tracheal, bronchus, and lung cancer	Number	2020	87783.8344	108740.262	69987.7615
Deaths	China	Male	60-64 years	Tracheal, bronchus, and lung cancer	Rate	2020	165.576237	220.46071	122.234153
Deaths	China	Female	60-64 years	Tracheal, bronchus, and lung cancer	Rate	2020	70.9444447	90.2710768	55.1178634
Deaths	China	Both	60-64 years	Tracheal, bronchus, and lung cancer	Rate	2020	118.486116	146.772026	94.4658896

Deaths	China	Male	65-69 years	Tracheal, bronchus, and lung cancer	Number	2020	92145.7577	121478.454	68224.4585
Deaths	China	Female	65-69 years	Tracheal, bronchus, and lung cancer	Number	2020	41561.8886	52338.4872	32784.8717
Deaths	China	Both	65-69 years	Tracheal, bronchus, and lung cancer	Number	2020	133707.646	164635.98	107831.474
Deaths	China	Male	65-69 years	Tracheal, bronchus, and lung cancer	Rate	2020	251.995552	332.213125	186.576794
Deaths	China	Female	65-69 years	Tracheal, bronchus, and lung cancer	Rate	2020	109.63696	138.064771	86.4838866
Deaths	China	Both	65-69 years	Tracheal, bronchus, and lung cancer	Rate	2020	179.533429	221.061868	144.788684
Deaths	China	Male	70-74 years	Tracheal, bronchus, and lung cancer	Number	2020	96497.9671	124611.665	72670.2767
Deaths	China	Female	70-74 years	Tracheal, bronchus, and lung cancer	Number	2020	42996.6663	53113.7985	34084.5712
Deaths	China	Both	70-74 years	Tracheal, bronchus, and lung cancer	Number	2020	139494.633	169727.018	112712.373
Deaths	China	Male	70-74 years	Tracheal, bronchus, and lung cancer	Rate	2020	396.310729	511.771815	298.45199
Deaths	China	Female	70-74 years	Tracheal, bronchus, and lung cancer	Rate	2020	167.776173	207.253971	133.000518
Deaths	China	Both	70-74 years	Tracheal, bronchus, and lung cancer	Rate	2020	279.12065	339.613894	225.530904
Deaths	China	Male	75-79 years	Tracheal, bronchus, and lung cancer	Number	2020	77543.9905	99357.6462	58718.5727
Deaths	China	Female	75-79 years	Tracheal, bronchus, and lung cancer	Number	2020	37752.5281	46628.1068	29705.8956
Deaths	China	Both	75-79 years	Tracheal, bronchus, and lung cancer	Number	2020	115296.519	138417.436	93751.7459
Deaths	China	Male	75-79 years	Tracheal, bronchus, and lung cancer	Rate	2020	522.921319	670.022668	395.971284
Deaths	China	Female	75-79 years	Tracheal, bronchus, and lung cancer	Rate	2020	227.927908	281.513514	179.347
Deaths	China	Both	75-79 years	Tracheal, bronchus, and lung cancer	Rate	2020	367.275713	440.927124	298.645091
Deaths	China	Male	80-84	Tracheal, bronchus, and lung cancer	Number	2020	53873.1565	67521.1413	41691.9963
Deaths	China	Female	80-84	Tracheal, bronchus, and lung cancer	Number	2020	30732.0947	38420.5566	24163.3537
Deaths	China	Both	80-84	Tracheal, bronchus, and lung cancer	Number	2020	84605.2512	99936.2847	70097.6121
Deaths	China	Male	80-84	Tracheal, bronchus, and lung cancer	Rate	2020	637.10542	798.506861	493.050687

Deaths	China	Female	80-84	Tracheal, bronchus, and lung cancer	Rate	2020	282.891698	353.664681	222.425845
Deaths	China	Both	80-84	Tracheal, bronchus, and lung cancer	Rate	2020	437.927169	517.282482	362.833848
Deaths	China	Male	85-89	Tracheal, bronchus, and lung cancer	Number	2020	31118.7249	37924.6229	24345.9468
Deaths	China	Female	85-89	Tracheal, bronchus, and lung cancer	Number	2020	18757.7178	23720.3638	14102.0149
Deaths	China	Both	85-89	Tracheal, bronchus, and lung cancer	Number	2020	49876.4428	58540.4088	41682.292
Deaths	China	Male	85-89	Tracheal, bronchus, and lung cancer	Rate	2020	944.987059	1151.6628	739.317075
Deaths	China	Female	85-89	Tracheal, bronchus, and lung cancer	Rate	2020	326.778539	413.232883	245.671454
Deaths	China	Both	85-89	Tracheal, bronchus, and lung cancer	Rate	2020	552.144391	648.056609	461.433143
Deaths	China	Male	90-94	Tracheal, bronchus, and lung cancer	Number	2020	8191.13149	10051.9515	6417.50432
Deaths	China	Female	90-94	Tracheal, bronchus, and lung cancer	Number	2020	7032.13551	8681.72591	5268.35316
Deaths	China	Both	90-94	Tracheal, bronchus, and lung cancer	Number	2020	15223.267	17843.2648	12350.4701
Deaths	China	Male	90-94	Tracheal, bronchus, and lung cancer	Rate	2020	1081.19553	1326.81608	847.08407
Deaths	China	Female	90-94	Tracheal, bronchus, and lung cancer	Rate	2020	353.87149	436.882264	265.114343
Deaths	China	Both	90-94	Tracheal, bronchus, and lung cancer	Rate	2020	554.622123	650.075269	449.958867
Deaths	China	Male	95+ years	Tracheal, bronchus, and lung cancer	Number	2020	949.402371	1129.07262	736.151659
Deaths	China	Female	95+ years	Tracheal, bronchus, and lung cancer	Number	2020	1856.68298	2367.67108	1335.89578
Deaths	China	Both	95+ years	Tracheal, bronchus, and lung cancer	Number	2020	2806.08535	3377.32832	2126.22369
Deaths	China	Male	95+ years	Tracheal, bronchus, and lung cancer	Rate	2020	842.163743	1001.53955	653.000514
Deaths	China	Female	95+ years	Tracheal, bronchus, and lung cancer	Rate	2020	389.821644	497.106637	280.479271
Deaths	China	Both	95+ years	Tracheal, bronchus, and lung cancer	Rate	2020	476.395693	573.376952	360.973984
Incidence	China	Female	45-49 years	Tracheal, bronchus, and lung cancer	Number	1990	4157.9976	5224.8699	3258.10806
Incidence	China	Both	45-49 years	Tracheal, bronchus, and lung cancer	Number	1990	13634.2028	16006.6956	11436.0161

Incidence	China	Male	45-49 years	Tracheal, bronchus, and lung cancer	Rate	1990	34.7893236	43.157004	26.9046375
Incidence	China	Female	45-49 years	Tracheal, bronchus, and lung cancer	Rate	1990	17.0548661	21.4308581	13.3637876
Incidence	China	Both	45-49 years	Tracheal, bronchus, and lung cancer	Rate	1990	26.4131704	31.0093362	22.1546832
Incidence	China	Male	45-49 years	Tracheal, bronchus, and lung cancer	Number	2000	15860.3879	18305.1218	13744.7842
Incidence	China	Female	45-49 years	Tracheal, bronchus, and lung cancer	Number	2000	7436.57792	8726.52674	6195.74818
Incidence	China	Both	45-49 years	Tracheal, bronchus, and lung cancer	Number	2000	23296.9658	26156.4362	20533.5805
Incidence	China	Male	45-49 years	Tracheal, bronchus, and lung cancer	Rate	2000	35.8228466	41.3446112	31.0444675
Incidence	China	Female	45-49 years	Tracheal, bronchus, and lung cancer	Rate	2000	17.7471431	20.8255626	14.7859447
Incidence	China	Both	45-49 years	Tracheal, bronchus, and lung cancer	Rate	2000	27.0337106	30.3518292	23.8270887
Incidence	China	Male	45-49 years	Tracheal, bronchus, and lung cancer	Number	2010	20177.6261	23459.8644	17437.4718
Incidence	China	Female	45-49 years	Tracheal, bronchus, and lung cancer	Number	2010	9780.89605	11324.3972	8447.10729
Incidence	China	Both	45-49 years	Tracheal, bronchus, and lung cancer	Number	2010	29958.5221	33897.5202	26915.096
Incidence	China	Male	45-49 years	Tracheal, bronchus, and lung cancer	Rate	2010	37.3021395	43.3699747	32.2364485
Incidence	China	Female	45-49 years	Tracheal, bronchus, and lung cancer	Rate	2010	18.7407163	21.6981464	16.1851062
Incidence	China	Both	45-49 years	Tracheal, bronchus, and lung cancer	Rate	2010	28.1874935	31.8936338	25.3239826
Incidence	China	Male	45-49 years	Tracheal, bronchus, and lung cancer	Number	2019	18731.699	24577.883	13899.2985
Incidence	China	Female	45-49 years	Tracheal, bronchus, and lung cancer	Number	2019	10054.1282	12654.8346	7594.50409
Incidence	China	Both	45-49 years	Tracheal, bronchus, and lung cancer	Number	2019	28785.8271	34830.121	23162.9654
Incidence	China	Male	45-49 years	Tracheal, bronchus, and lung cancer	Rate	2019	31.1011015	40.8077898	23.0776448
Incidence	China	Female	45-49 years	Tracheal, bronchus, and lung cancer	Rate	2019	17.3730432	21.8669371	13.1229328
Incidence	China	Both	45-49 years	Tracheal, bronchus, and lung cancer	Rate	2019	24.3740274	29.4919552	19.6129418
Incidence	China	Male	45-49 years	Tracheal, bronchus, and lung cancer	Number	2020	18256.7352	24397.0216	13393.4201

Incidence	China	Female	45-49 years	Tracheal, bronchus, and lung cancer	Number	2020	10068.4811	13055.1087	7614.79075
Incidence	China	Both	45-49 years	Tracheal, bronchus, and lung cancer	Number	2020	28325.2163	34677.4912	22529.8522
Incidence	China	Male	45-49 years	Tracheal, bronchus, and lung cancer	Rate	2020	31.2314209	41.7354824	22.9118479
Incidence	China	Female	45-49 years	Tracheal, bronchus, and lung cancer	Rate	2020	17.8500079	23.1448806	13.4999583
Incidence	China	Both	45-49 years	Tracheal, bronchus, and lung cancer	Rate	2020	24.6601425	30.1904799	19.6146557
Incidence	China	Male	45-49 years	Tracheal, bronchus, and lung cancer	Number	2021	17557.0968	23681.2434	12633.9564
Incidence	China	Female	45-49 years	Tracheal, bronchus, and lung cancer	Number	2021	9892.88877	12954.7698	7439.15942
Incidence	China	Both	45-49 years	Tracheal, bronchus, and lung cancer	Number	2021	27449.9856	34219.5193	21242.7738
Incidence	China	Male	45-49 years	Tracheal, bronchus, and lung cancer	Rate	2021	31.3175755	42.2415584	22.5358946
Incidence	China	Female	45-49 years	Tracheal, bronchus, and lung cancer	Rate	2021	18.2323522	23.875324	13.710189
Incidence	China	Both	45-49 years	Tracheal, bronchus, and lung cancer	Rate	2021	24.8817959	31.0179796	19.2553238
Incidence	China	Male	50-54 years	Tracheal, bronchus, and lung cancer	Number	1990	18454.0858	22942.8604	14237.1126
Incidence	China	Female	50-54 years	Tracheal, bronchus, and lung cancer	Number	1990	7328.07207	9158.80098	5756.51618
Incidence	China	Both	50-54 years	Tracheal, bronchus, and lung cancer	Number	1990	25782.1579	30519.1906	21145.0343
Incidence	China	Male	50-54 years	Tracheal, bronchus, and lung cancer	Rate	1990	73.0693147	90.8427055	56.3721266
Incidence	China	Female	50-54 years	Tracheal, bronchus, and lung cancer	Rate	1990	32.6340605	40.7868348	25.6354598
Incidence	China	Both	50-54 years	Tracheal, bronchus, and lung cancer	Rate	1990	54.0383238	63.966946	44.3191069
Incidence	China	Male	50-54 years	Tracheal, bronchus, and lung cancer	Number	2000	24312.2611	27914.7837	21161.3202
Incidence	China	Female	50-54 years	Tracheal, bronchus, and lung cancer	Number	2000	10318.5701	12042.9653	8854.10711
Incidence	China	Both	50-54 years	Tracheal, bronchus, and lung cancer	Number	2000	34630.8312	38868.3577	30993.1165
Incidence	China	Male	50-54 years	Tracheal, bronchus, and lung cancer	Rate	2000	73.5592806	84.4590884	64.0257805
Incidence	China	Female	50-54 years	Tracheal, bronchus, and lung cancer	Rate	2000	33.5892606	39.2025537	28.822105

Incidence	China	Both	50-54 years	Tracheal, bronchus, and lung cancer	Rate	2000	54.3049003	60.9498015	48.600569
Incidence	China	Male	50-54 years	Tracheal, bronchus, and lung cancer	Number	2010	28140.2571	32566.4701	24430.963
Incidence	China	Female	50-54 years	Tracheal, bronchus, and lung cancer	Number	2010	11965.3978	13859.5144	10082.8135
Incidence	China	Both	50-54 years	Tracheal, bronchus, and lung cancer	Number	2010	40105.6549	44990.2431	36031.0043
Incidence	China	Male	50-54 years	Tracheal, bronchus, and lung cancer	Rate	2010	69.1842843	80.0663589	60.064792
Incidence	China	Female	50-54 years	Tracheal, bronchus, and lung cancer	Rate	2010	30.9128091	35.8062916	26.0491204
Incidence	China	Both	50-54 years	Tracheal, bronchus, and lung cancer	Rate	2010	50.522816	56.6761416	45.3898037
Incidence	China	Male	50-54 years	Tracheal, bronchus, and lung cancer	Number	2019	41248.8682	55144.545	30767.9661
Incidence	China	Female	50-54 years	Tracheal, bronchus, and lung cancer	Number	2019	20113.0746	25457.8923	15180.8323
Incidence	China	Both	50-54 years	Tracheal, bronchus, and lung cancer	Number	2019	61361.9427	75536.1516	49112.464
Incidence	China	Male	50-54 years	Tracheal, bronchus, and lung cancer	Rate	2019	67.0773095	89.6739201	50.0336731
Incidence	China	Female	50-54 years	Tracheal, bronchus, and lung cancer	Rate	2019	33.0630449	41.849168	24.9551373
Incidence	China	Both	50-54 years	Tracheal, bronchus, and lung cancer	Rate	2019	50.1622185	61.7493641	40.1485032
Incidence	China	Male	50-54 years	Tracheal, bronchus, and lung cancer	Number	2020	41298.2313	54048.1827	30060.7053
Incidence	China	Female	50-54 years	Tracheal, bronchus, and lung cancer	Number	2020	20623.0926	26699.5177	15871.1948
Incidence	China	Both	50-54 years	Tracheal, bronchus, and lung cancer	Number	2020	61921.3239	77036.8482	49059.9744
Incidence	China	Male	50-54 years	Tracheal, bronchus, and lung cancer	Rate	2020	67.2723181	88.0412168	48.9670687
Incidence	China	Female	50-54 years	Tracheal, bronchus, and lung cancer	Rate	2020	34.1225182	44.1764384	26.260132
Incidence	China	Both	50-54 years	Tracheal, bronchus, and lung cancer	Rate	2020	50.8268399	63.2341059	40.2698668
Incidence	China	Male	50-54 years	Tracheal, bronchus, and lung cancer	Number	2021	41148.3103	56517.8259	29294.3151
Incidence	China	Female	50-54 years	Tracheal, bronchus, and lung cancer	Number	2021	20764.1405	27296.2956	15936.3652
Incidence	China	Both	50-54 years	Tracheal, bronchus, and lung cancer	Number	2021	61912.4509	77912.3328	48850.0813

Incidence	China	Male	50-54 years	Tracheal, bronchus, and lung cancer	Rate	2021	67.3006424	92.4384491	47.9126898
Incidence	China	Female	50-54 years	Tracheal, bronchus, and lung cancer	Rate	2021	34.7703231	45.708659	26.6860343
Incidence	China	Both	50-54 years	Tracheal, bronchus, and lung cancer	Rate	2021	51.2269948	64.4654607	40.4190567
Incidence	China	Male	55-59 years	Tracheal, bronchus, and lung cancer	Number	1990	27087.7076	33628.8859	20644.4976
Incidence	China	Female	55-59 years	Tracheal, bronchus, and lung cancer	Number	1990	10538.8139	12982.8281	8461.59537
Incidence	China	Both	55-59 years	Tracheal, bronchus, and lung cancer	Number	1990	37626.5215	44454.1332	30976.4284
Incidence	China	Male	55-59 years	Tracheal, bronchus, and lung cancer	Rate	1990	119.178186	147.957505	90.8299003
Incidence	China	Female	55-59 years	Tracheal, bronchus, and lung cancer	Rate	1990	51.0592647	62.9002142	40.9953947
Incidence	China	Both	55-59 years	Tracheal, bronchus, and lung cancer	Rate	1990	86.7588205	102.501853	71.4251088
Incidence	China	Male	55-59 years	Tracheal, bronchus, and lung cancer	Number	2000	28751.0662	32801.9601	24763.5935
Incidence	China	Female	55-59 years	Tracheal, bronchus, and lung cancer	Number	2000	12057.4898	14070.6878	10201.4881
Incidence	China	Both	55-59 years	Tracheal, bronchus, and lung cancer	Number	2000	40808.556	45926.5599	36330.2723
Incidence	China	Male	55-59 years	Tracheal, bronchus, and lung cancer	Rate	2000	118.400687	135.082803	101.979748
Incidence	China	Female	55-59 years	Tracheal, bronchus, and lung cancer	Rate	2000	53.6165552	62.568729	45.363393
Incidence	China	Both	55-59 years	Tracheal, bronchus, and lung cancer	Rate	2000	87.2514157	98.1940496	77.6765464
Incidence	China	Male	55-59 years	Tracheal, bronchus, and lung cancer	Number	2010	50479.2419	58489.0789	43421.0107
Incidence	China	Female	55-59 years	Tracheal, bronchus, and lung cancer	Number	2010	20342.9753	23395.3758	17592.0487
Incidence	China	Both	55-59 years	Tracheal, bronchus, and lung cancer	Number	2010	70822.2172	79660.8556	63230.445
Incidence	China	Male	55-59 years	Tracheal, bronchus, and lung cancer	Rate	2010	122.148922	141.531007	105.069519
Incidence	China	Female	55-59 years	Tracheal, bronchus, and lung cancer	Rate	2010	50.2558312	57.7965632	43.4598685
Incidence	China	Both	55-59 years	Tracheal, bronchus, and lung cancer	Rate	2010	86.5746285	97.3791735	77.29428
Incidence	China	Male	55-59 years	Tracheal, bronchus, and lung cancer	Number	2019	55099.3395	73362.782	41449.341

Incidence	China	Female	55-59 years	Tracheal, bronchus, and lung cancer	Number	2019	24332.4792	30372.2477	18572.177
Incidence	China	Both	55-59 years	Tracheal, bronchus, and lung cancer	Number	2019	79431.8187	97056.9032	63792.8407
Incidence	China	Male	55-59 years	Tracheal, bronchus, and lung cancer	Rate	2019	117.828846	156.884856	88.6385946
Incidence	China	Female	55-59 years	Tracheal, bronchus, and lung cancer	Rate	2019	52.5050743	65.5377989	40.0753875
Incidence	China	Both	55-59 years	Tracheal, bronchus, and lung cancer	Rate	2019	85.3139766	104.24425	68.5168867
Incidence	China	Male	55-59 years	Tracheal, bronchus, and lung cancer	Number	2020	60141.6596	80679.067	44181.2356
Incidence	China	Female	55-59 years	Tracheal, bronchus, and lung cancer	Number	2020	27121.892	35008.1663	20510.1456
Incidence	China	Both	55-59 years	Tracheal, bronchus, and lung cancer	Number	2020	87263.5515	108131.053	68521.8132
Incidence	China	Male	55-59 years	Tracheal, bronchus, and lung cancer	Rate	2020	117.627501	157.795396	86.4114551
Incidence	China	Female	55-59 years	Tracheal, bronchus, and lung cancer	Rate	2020	53.228989	68.7064641	40.2528819
Incidence	China	Both	55-59 years	Tracheal, bronchus, and lung cancer	Rate	2020	85.4836572	105.925529	67.1241898
Incidence	China	Male	55-59 years	Tracheal, bronchus, and lung cancer	Number	2021	64737.1582	86933.64	46196.0732
Incidence	China	Female	55-59 years	Tracheal, bronchus, and lung cancer	Number	2021	29964.8502	38993.2259	22529.9842
Incidence	China	Both	55-59 years	Tracheal, bronchus, and lung cancer	Number	2021	94702.0083	119137.915	72339.6316
Incidence	China	Male	55-59 years	Tracheal, bronchus, and lung cancer	Rate	2021	117.95364	158.396501	84.1710566
Incidence	China	Female	55-59 years	Tracheal, bronchus, and lung cancer	Rate	2021	54.4233487	70.8210426	40.9198504
Incidence	China	Both	55-59 years	Tracheal, bronchus, and lung cancer	Rate	2021	86.1378596	108.363964	65.7977707
Incidence	China	Male	60-64 years	Tracheal, bronchus, and lung cancer	Number	1990	31807.1719	39137.6827	24802.7875
Incidence	China	Female	60-64 years	Tracheal, bronchus, and lung cancer	Number	1990	11975.1115	14542.7193	9603.976
Incidence	China	Both	60-64 years	Tracheal, bronchus, and lung cancer	Number	1990	43782.2835	51068.5822	36643.3898
Incidence	China	Male	60-64 years	Tracheal, bronchus, and lung cancer	Rate	1990	174.736675	215.007752	136.257214
Incidence	China	Female	60-64 years	Tracheal, bronchus, and lung cancer	Rate	1990	69.8887051	84.873683	56.0503713

Incidence	China	Both	60-64 years	Tracheal, bronchus, and lung cancer	Rate	1990	123.897646	144.516837	103.695591
Incidence	China	Male	60-64 years	Tracheal, bronchus, and lung cancer	Number	2000	39335.7015	44369.6492	34320.1419
Incidence	China	Female	60-64 years	Tracheal, bronchus, and lung cancer	Number	2000	15618.7146	17857.1317	13614.657
Incidence	China	Both	60-64 years	Tracheal, bronchus, and lung cancer	Number	2000	54954.4161	60749.4941	49491.3422
Incidence	China	Male	60-64 years	Tracheal, bronchus, and lung cancer	Rate	2000	180.276198	203.346867	157.289801
Incidence	China	Female	60-64 years	Tracheal, bronchus, and lung cancer	Rate	2000	77.4544364	88.5549231	67.5161566
Incidence	China	Both	60-64 years	Tracheal, bronchus, and lung cancer	Rate	2000	130.891461	144.694286	117.879408
Incidence	China	Male	60-64 years	Tracheal, bronchus, and lung cancer	Number	2010	64963.1297	74480.4625	56409.4349
Incidence	China	Female	60-64 years	Tracheal, bronchus, and lung cancer	Number	2010	24065.5098	27363.9424	21171.0468
Incidence	China	Both	60-64 years	Tracheal, bronchus, and lung cancer	Number	2010	89028.6395	99599.6536	80071.4095
Incidence	China	Male	60-64 years	Tracheal, bronchus, and lung cancer	Rate	2010	216.510568	248.230147	188.002624
Incidence	China	Female	60-64 years	Tracheal, bronchus, and lung cancer	Rate	2010	82.9895276	94.3641199	73.0080179
Incidence	China	Both	60-64 years	Tracheal, bronchus, and lung cancer	Rate	2010	150.888714	168.804821	135.707701
Incidence	China	Male	60-64 years	Tracheal, bronchus, and lung cancer	Number	2019	79157.9809	104725.988	60567.4173
Incidence	China	Female	60-64 years	Tracheal, bronchus, and lung cancer	Number	2019	34350.4778	42305.46	26559.6575
Incidence	China	Both	60-64 years	Tracheal, bronchus, and lung cancer	Number	2019	113508.459	138836.046	92559.1097
Incidence	China	Male	60-64 years	Tracheal, bronchus, and lung cancer	Rate	2019	204.312835	270.305827	156.329161
Incidence	China	Female	60-64 years	Tracheal, bronchus, and lung cancer	Rate	2019	89.4809907	110.203255	69.1863581
Incidence	China	Both	60-64 years	Tracheal, bronchus, and lung cancer	Rate	2019	147.161127	179.997766	120.000774
Incidence	China	Male	60-64 years	Tracheal, bronchus, and lung cancer	Number	2020	75772.7754	100076.517	56122.5081
Incidence	China	Female	60-64 years	Tracheal, bronchus, and lung cancer	Number	2020	33795.4827	42909.1397	26332.717
Incidence	China	Both	60-64 years	Tracheal, bronchus, and lung cancer	Number	2020	109568.258	135677.733	88468.6849

Incidence	China	Male	60-64 years	Tracheal, bronchus, and lung cancer	Rate	2020	203.577011	268.873326	150.78308
Incidence	China	Female	60-64 years	Tracheal, bronchus, and lung cancer	Rate	2020	91.6682258	116.388476	71.4259202
Incidence	China	Both	60-64 years	Tracheal, bronchus, and lung cancer	Rate	2020	147.889613	183.130843	119.410492
Incidence	China	Male	60-64 years	Tracheal, bronchus, and lung cancer	Number	2021	73717.5339	97880.4688	53693.07
Incidence	China	Female	60-64 years	Tracheal, bronchus, and lung cancer	Number	2021	33516.8339	42999.5907	26024.6205
Incidence	China	Both	60-64 years	Tracheal, bronchus, and lung cancer	Number	2021	107234.368	132324.296	84923.1546
Incidence	China	Male	60-64 years	Tracheal, bronchus, and lung cancer	Rate	2021	201.274633	267.247891	146.600848
Incidence	China	Female	60-64 years	Tracheal, bronchus, and lung cancer	Rate	2021	92.1302937	118.196275	71.5358717
Incidence	China	Both	60-64 years	Tracheal, bronchus, and lung cancer	Rate	2021	146.885999	181.253332	116.324856
Incidence	China	Male	65-69 years	Tracheal, bronchus, and lung cancer	Number	1990	33183.6289	40474.388	26283.3919
Incidence	China	Female	65-69 years	Tracheal, bronchus, and lung cancer	Number	1990	13361.1696	16012.6557	10986.3407
Incidence	China	Both	65-69 years	Tracheal, bronchus, and lung cancer	Number	1990	46544.7985	54300.6399	39355.5867
Incidence	China	Male	65-69 years	Tracheal, bronchus, and lung cancer	Rate	1990	247.480094	301.853826	196.018836
Incidence	China	Female	65-69 years	Tracheal, bronchus, and lung cancer	Rate	1990	96.3091692	115.42145	79.1910717
Incidence	China	Both	65-69 years	Tracheal, bronchus, and lung cancer	Rate	1990	170.607434	199.03605	144.255768
Incidence	China	Male	65-69 years	Tracheal, bronchus, and lung cancer	Number	2000	48490.5908	54703.3893	42407.2599
Incidence	China	Female	65-69 years	Tracheal, bronchus, and lung cancer	Number	2000	19959.1523	22578.7521	17365.8104
Incidence	China	Both	65-69 years	Tracheal, bronchus, and lung cancer	Number	2000	68449.7432	75482.5386	61713.8433
Incidence	China	Male	65-69 years	Tracheal, bronchus, and lung cancer	Rate	2000	272.329702	307.221617	238.164895
Incidence	China	Female	65-69 years	Tracheal, bronchus, and lung cancer	Rate	2000	114.433507	129.452682	99.5648796
Incidence	China	Both	65-69 years	Tracheal, bronchus, and lung cancer	Rate	2000	194.197206	214.149789	175.086938
Incidence	China	Male	65-69 years	Tracheal, bronchus, and lung cancer	Number	2010	64152.0043	73265.9015	56250.2781

Incidence	China	Female	65-69 years	Tracheal, bronchus, and lung cancer	Number	2010	24269.8435	27188.4972	21110.2441
Incidence	China	Both	65-69 years	Tracheal, bronchus, and lung cancer	Number	2010	88421.8478	98806.1314	79468.9504
Incidence	China	Male	65-69 years	Tracheal, bronchus, and lung cancer	Rate	2010	307.5396	351.230898	269.659354
Incidence	China	Female	65-69 years	Tracheal, bronchus, and lung cancer	Rate	2010	118.54371	132.799592	103.110951
Incidence	China	Both	65-69 years	Tracheal, bronchus, and lung cancer	Rate	2010	213.925121	239.04854	192.264755
Incidence	China	Male	65-69 years	Tracheal, bronchus, and lung cancer	Number	2019	101662.848	132496.408	77409.0581
Incidence	China	Female	65-69 years	Tracheal, bronchus, and lung cancer	Number	2019	46150.2752	56647.8134	36371.8985
Incidence	China	Both	65-69 years	Tracheal, bronchus, and lung cancer	Number	2019	147813.124	177374.251	120879.915
Incidence	China	Male	65-69 years	Tracheal, bronchus, and lung cancer	Rate	2019	297.321878	387.497317	226.389551
Incidence	China	Female	65-69 years	Tracheal, bronchus, and lung cancer	Rate	2019	130.066905	159.652478	102.508171
Incidence	China	Both	65-69 years	Tracheal, bronchus, and lung cancer	Rate	2019	212.147153	254.574436	173.491563
Incidence	China	Male	65-69 years	Tracheal, bronchus, and lung cancer	Number	2020	109571.695	141677.764	80862.3215
Incidence	China	Female	65-69 years	Tracheal, bronchus, and lung cancer	Number	2020	51373.5922	64590.5359	40549.3875
Incidence	China	Both	65-69 years	Tracheal, bronchus, and lung cancer	Number	2020	160945.287	195676.561	131559.026
Incidence	China	Male	65-69 years	Tracheal, bronchus, and lung cancer	Rate	2020	299.651122	387.453174	221.138182
Incidence	China	Female	65-69 years	Tracheal, bronchus, and lung cancer	Rate	2020	135.519454	170.384701	106.966062
Incidence	China	Both	65-69 years	Tracheal, bronchus, and lung cancer	Rate	2020	216.106259	262.741024	176.64841
Incidence	China	Male	65-69 years	Tracheal, bronchus, and lung cancer	Number	2021	113171.595	148598.131	81942.5521
Incidence	China	Female	65-69 years	Tracheal, bronchus, and lung cancer	Number	2021	53857.7178	68439.0609	41730.6362
Incidence	China	Both	65-69 years	Tracheal, bronchus, and lung cancer	Number	2021	167029.313	202929.525	132932.342
Incidence	China	Male	65-69 years	Tracheal, bronchus, and lung cancer	Rate	2021	299.871065	393.740848	217.123391
Incidence	China	Female	65-69 years	Tracheal, bronchus, and lung cancer	Rate	2021	138.226337	175.64949	107.102068

Incidence	China	Both	65-69 years	Tracheal, bronchus, and lung cancer	Rate	2021	217.759667	264.563538	173.306721
Incidence	China	Male	70-74 years	Tracheal, bronchus, and lung cancer	Number	1990	28180.6332	33624.5278	22669.1491
Incidence	China	Female	70-74 years	Tracheal, bronchus, and lung cancer	Number	1990	12435.4255	14972.8411	10321.8482
Incidence	China	Both	70-74 years	Tracheal, bronchus, and lung cancer	Number	1990	40616.0587	46285.9434	34747.4095
Incidence	China	Male	70-74 years	Tracheal, bronchus, and lung cancer	Rate	1990	322.975469	385.367411	259.808891
Incidence	China	Female	70-74 years	Tracheal, bronchus, and lung cancer	Rate	1990	123.216547	148.358557	102.274144
Incidence	China	Both	70-74 years	Tracheal, bronchus, and lung cancer	Rate	1990	215.840218	245.97089	184.65328
Incidence	China	Male	70-74 years	Tracheal, bronchus, and lung cancer	Number	2000	48041.2041	53934.1216	42509.3746
Incidence	China	Female	70-74 years	Tracheal, bronchus, and lung cancer	Number	2000	20493.1336	23421.7975	18140.1941
Incidence	China	Both	70-74 years	Tracheal, bronchus, and lung cancer	Number	2000	68534.3377	75452.6305	62221.8132
Incidence	China	Male	70-74 years	Tracheal, bronchus, and lung cancer	Rate	2000	382.768228	429.72004	338.693384
Incidence	China	Female	70-74 years	Tracheal, bronchus, and lung cancer	Rate	2000	154.574212	176.664339	136.826621
Incidence	China	Both	70-74 years	Tracheal, bronchus, and lung cancer	Rate	2000	265.546533	292.352493	241.087714
Incidence	China	Male	70-74 years	Tracheal, bronchus, and lung cancer	Number	2010	72833.63	82278.0418	64530.8538
Incidence	China	Female	70-74 years	Tracheal, bronchus, and lung cancer	Number	2010	29184.5139	32664.7985	25757.526
Incidence	China	Both	70-74 years	Tracheal, bronchus, and lung cancer	Number	2010	102018.144	112676.687	92145.2274
Incidence	China	Male	70-74 years	Tracheal, bronchus, and lung cancer	Rate	2010	440.450192	497.563822	390.240428
Incidence	China	Female	70-74 years	Tracheal, bronchus, and lung cancer	Rate	2010	174.796919	195.641639	154.271413
Incidence	China	Both	70-74 years	Tracheal, bronchus, and lung cancer	Rate	2010	306.983804	339.056531	277.275113
Incidence	China	Male	70-74 years	Tracheal, bronchus, and lung cancer	Number	2019	104256.121	133899.894	80322.4949
Incidence	China	Female	70-74 years	Tracheal, bronchus, and lung cancer	Number	2019	46015.0722	55060.223	36881.6037
Incidence	China	Both	70-74 years	Tracheal, bronchus, and lung cancer	Number	2019	150271.193	179516.726	122751.198

Incidence	China	Male	70-74 years	Tracheal, bronchus, and lung cancer	Rate	2019	446.521667	573.483876	344.015624
Incidence	China	Female	70-74 years	Tracheal, bronchus, and lung cancer	Rate	2019	188.517785	225.574595	151.099149
Incidence	China	Both	70-74 years	Tracheal, bronchus, and lung cancer	Rate	2019	314.655451	375.893179	257.030857
Incidence	China	Male	70-74 years	Tracheal, bronchus, and lung cancer	Number	2020	109510.901	140992.931	82398.4253
Incidence	China	Female	70-74 years	Tracheal, bronchus, and lung cancer	Number	2020	50077.8992	61527.331	39813.1163
Incidence	China	Both	70-74 years	Tracheal, bronchus, and lung cancer	Number	2020	159588.8	192743.923	129802.682
Incidence	China	Male	70-74 years	Tracheal, bronchus, and lung cancer	Rate	2020	449.753981	579.048584	338.404849
Incidence	China	Female	70-74 years	Tracheal, bronchus, and lung cancer	Rate	2020	195.407667	240.084197	155.353725
Incidence	China	Both	70-74 years	Tracheal, bronchus, and lung cancer	Rate	2020	319.327909	385.669382	259.727618
Incidence	China	Male	70-74 years	Tracheal, bronchus, and lung cancer	Number	2021	116018.069	149431.128	85436.7099
Incidence	China	Female	70-74 years	Tracheal, bronchus, and lung cancer	Number	2021	54426.9985	68522.8591	43232.3825
Incidence	China	Both	70-74 years	Tracheal, bronchus, and lung cancer	Number	2021	170445.067	207533.518	135364.659
Incidence	China	Male	70-74 years	Tracheal, bronchus, and lung cancer	Rate	2021	448.699954	577.924981	330.426529
Incidence	China	Female	70-74 years	Tracheal, bronchus, and lung cancer	Rate	2021	198.349482	249.71933	157.552702
Incidence	China	Both	70-74 years	Tracheal, bronchus, and lung cancer	Rate	2021	319.805713	389.394693	253.984419
Incidence	China	Male	75-79 years	Tracheal, bronchus, and lung cancer	Number	1990	17628.7195	21005.933	14416.0707
Incidence	China	Female	75-79 years	Tracheal, bronchus, and lung cancer	Number	1990	9286.6364	11266.3953	7660.4816
Incidence	China	Both	75-79 years	Tracheal, bronchus, and lung cancer	Number	1990	26915.356	30740.1789	23369.9519
Incidence	China	Male	75-79 years	Tracheal, bronchus, and lung cancer	Rate	1990	359.987208	428.951584	294.383324
Incidence	China	Female	75-79 years	Tracheal, bronchus, and lung cancer	Rate	1990	143.231467	173.766071	118.150638
Incidence	China	Both	75-79 years	Tracheal, bronchus, and lung cancer	Rate	1990	236.500064	270.108049	205.34728
Incidence	China	Male	75-79 years	Tracheal, bronchus, and lung cancer	Number	2000	32311.3688	36267.9919	28609.1741

Incidence	China	Female	75-79 years	Tracheal, bronchus, and lung cancer	Number	2000	16172.3205	18366.297	14160.8178
Incidence	China	Both	75-79 years	Tracheal, bronchus, and lung cancer	Number	2000	48483.6893	53608.2217	44093.3903
Incidence	China	Male	75-79 years	Tracheal, bronchus, and lung cancer	Rate	2000	444.17608	498.56676	393.28296
Incidence	China	Female	75-79 years	Tracheal, bronchus, and lung cancer	Rate	2000	182.239083	206.962083	159.572305
Incidence	China	Both	75-79 years	Tracheal, bronchus, and lung cancer	Rate	2000	300.233081	331.96652	273.046351
Incidence	China	Male	75-79 years	Tracheal, bronchus, and lung cancer	Number	2010	64015.6799	72510.7161	56731.1965
Incidence	China	Female	75-79 years	Tracheal, bronchus, and lung cancer	Number	2010	28326.2364	32025.2722	24680.3712
Incidence	China	Both	75-79 years	Tracheal, bronchus, and lung cancer	Number	2010	92341.9164	101104.633	82790.9716
Incidence	China	Male	75-79 years	Tracheal, bronchus, and lung cancer	Rate	2010	565.629577	640.689995	501.265357
Incidence	China	Female	75-79 years	Tracheal, bronchus, and lung cancer	Rate	2010	223.785973	253.009492	194.982517
Incidence	China	Both	75-79 years	Tracheal, bronchus, and lung cancer	Rate	2010	385.153841	421.702722	345.317294
Incidence	China	Male	75-79 years	Tracheal, bronchus, and lung cancer	Number	2019	80210.937	101807.75	62749.4082
Incidence	China	Female	75-79 years	Tracheal, bronchus, and lung cancer	Number	2019	38374.6909	46610.461	30140.2753
Incidence	China	Both	75-79 years	Tracheal, bronchus, and lung cancer	Number	2019	118585.628	139899.929	98588.2555
Incidence	China	Male	75-79 years	Tracheal, bronchus, and lung cancer	Rate	2019	558.018624	708.265266	436.540698
Incidence	China	Female	75-79 years	Tracheal, bronchus, and lung cancer	Rate	2019	240.225776	291.781742	188.678289
Incidence	China	Both	75-79 years	Tracheal, bronchus, and lung cancer	Rate	2019	390.744107	460.97553	324.852012
Incidence	China	Male	75-79 years	Tracheal, bronchus, and lung cancer	Number	2020	83016.6919	104971.859	62407.6757
Incidence	China	Female	75-79 years	Tracheal, bronchus, and lung cancer	Number	2020	40820.1614	50399.977	31798.7945
Incidence	China	Both	75-79 years	Tracheal, bronchus, and lung cancer	Number	2020	123836.853	148107.857	102208.18
Incidence	China	Male	75-79 years	Tracheal, bronchus, and lung cancer	Rate	2020	559.826722	707.882358	420.848913
Incidence	China	Female	75-79 years	Tracheal, bronchus, and lung cancer	Rate	2020	246.448503	304.285883	191.982712

Incidence	China	Both	75-79 years	Tracheal, bronchus, and lung cancer	Rate	2020	394.480849	471.795848	325.582962
Incidence	China	Male	75-79 years	Tracheal, bronchus, and lung cancer	Number	2021	87273.6629	112122.022	64769.2244
Incidence	China	Female	75-79 years	Tracheal, bronchus, and lung cancer	Number	2021	43650.2739	54837.5711	34826.2078
Incidence	China	Both	75-79 years	Tracheal, bronchus, and lung cancer	Number	2021	130923.937	158243.925	106199.605
Incidence	China	Male	75-79 years	Tracheal, bronchus, and lung cancer	Rate	2021	559.35939	718.618923	415.122645
Incidence	China	Female	75-79 years	Tracheal, bronchus, and lung cancer	Rate	2021	249.195097	313.06227	198.819377
Incidence	China	Both	75-79 years	Tracheal, bronchus, and lung cancer	Rate	2021	395.31443	477.804964	320.661275
Incidence	China	Male	<1 year	Tracheal, bronchus, and lung cancer	Number	1990	0	0	0
Incidence	China	Female	<1 year	Tracheal, bronchus, and lung cancer	Number	1990	0	0	0
Incidence	China	Both	<1 year	Tracheal, bronchus, and lung cancer	Number	1990	0	0	0
Incidence	China	Male	<1 year	Tracheal, bronchus, and lung cancer	Rate	1990	0	0	0
Incidence	China	Female	<1 year	Tracheal, bronchus, and lung cancer	Rate	1990	0	0	0
Incidence	China	Both	<1 year	Tracheal, bronchus, and lung cancer	Rate	1990	0	0	0
Incidence	China	Male	<1 year	Tracheal, bronchus, and lung cancer	Number	2000	0	0	0
Incidence	China	Female	<1 year	Tracheal, bronchus, and lung cancer	Number	2000	0	0	0
Incidence	China	Both	<1 year	Tracheal, bronchus, and lung cancer	Number	2000	0	0	0
Incidence	China	Male	<1 year	Tracheal, bronchus, and lung cancer	Rate	2000	0	0	0
Incidence	China	Female	<1 year	Tracheal, bronchus, and lung cancer	Rate	2000	0	0	0
Incidence	China	Both	<1 year	Tracheal, bronchus, and lung cancer	Rate	2000	0	0	0
Incidence	China	Male	<1 year	Tracheal, bronchus, and lung cancer	Number	2010	0	0	0
Incidence	China	Female	<1 year	Tracheal, bronchus, and lung cancer	Number	2010	0	0	0
Incidence	China	Both	<1 year	Tracheal, bronchus, and lung cancer	Number	2010	0	0	0

Incidence	China	Male	<1 year	Tracheal, bronchus, and lung cancer	Rate	2010	0	0	0
Incidence	China	Female	<1 year	Tracheal, bronchus, and lung cancer	Rate	2010	0	0	0
Incidence	China	Both	<1 year	Tracheal, bronchus, and lung cancer	Rate	2010	0	0	0
Incidence	China	Male	<1 year	Tracheal, bronchus, and lung cancer	Number	2019	0	0	0
Incidence	China	Female	<1 year	Tracheal, bronchus, and lung cancer	Number	2019	0	0	0
Incidence	China	Both	<1 year	Tracheal, bronchus, and lung cancer	Number	2019	0	0	0
Incidence	China	Male	<1 year	Tracheal, bronchus, and lung cancer	Rate	2019	0	0	0
Incidence	China	Female	<1 year	Tracheal, bronchus, and lung cancer	Rate	2019	0	0	0
Incidence	China	Both	<1 year	Tracheal, bronchus, and lung cancer	Rate	2019	0	0	0
Incidence	China	Male	<1 year	Tracheal, bronchus, and lung cancer	Number	2020	0	0	0
Incidence	China	Female	<1 year	Tracheal, bronchus, and lung cancer	Number	2020	0	0	0
Incidence	China	Both	<1 year	Tracheal, bronchus, and lung cancer	Number	2020	0	0	0
Incidence	China	Male	<1 year	Tracheal, bronchus, and lung cancer	Rate	2020	0	0	0
Incidence	China	Female	<1 year	Tracheal, bronchus, and lung cancer	Rate	2020	0	0	0
Incidence	China	Both	<1 year	Tracheal, bronchus, and lung cancer	Rate	2020	0	0	0
Incidence	China	Male	<1 year	Tracheal, bronchus, and lung cancer	Number	2021	0	0	0
Incidence	China	Female	<1 year	Tracheal, bronchus, and lung cancer	Number	2021	0	0	0
Incidence	China	Both	<1 year	Tracheal, bronchus, and lung cancer	Number	2021	0	0	0
Incidence	China	Male	<1 year	Tracheal, bronchus, and lung cancer	Rate	2021	0	0	0
Incidence	China	Female	<1 year	Tracheal, bronchus, and lung cancer	Rate	2021	0	0	0
Incidence	China	Both	<1 year	Tracheal, bronchus, and lung cancer	Rate	2021	0	0	0
Incidence	China	Male	80-84	Tracheal, bronchus, and lung cancer	Number	1990	7151.54602	8639.00977	5896.06038

**Incidence**	**China**	**Female**	**80-84**	**Tracheal, bronchus, and lung cancer**	**Number**	**1990**	**4777.43258**	**5671.35222**	**3997.60174**
**Incidence**	**China**	**Both**	**80-84**	**Tracheal, bronchus, and lung cancer**	**Number**	**1990**	**11928.9786**	**13590.8832**	**10301.7442**
**Incidence**	**China**	**Male**	**80-84**	**Tracheal, bronchus, and lung cancer**	**Rate**	**1990**	**350.591973**	**423.512268**	**289.043996**
**Incidence**	**China**	**Female**	**80-84**	**Tracheal, bronchus, and lung cancer**	**Rate**	**1990**	**146.669532**	**174.113306**	**122.728342**
**Incidence**	**China**	**Both**	**80-84**	**Tracheal, bronchus, and lung cancer**	**Rate**	**1990**	**225.197205**	**256.570912**	**194.478008**
**Incidence**	**China**	**Male**	**80-84**	**Tracheal, bronchus, and lung cancer**	**Number**	**2000**	**14711.8916**	**16559.0232**	**12985.2862**
**Incidence**	**China**	**Female**	**80-84**	**Tracheal, bronchus, and lung cancer**	**Number**	**2000**	**8974.17011**	**10235.3944**	**7653.89182**
**Incidence**	**China**	**Both**	**80-84**	**Tracheal, bronchus, and lung cancer**	**Number**	**2000**	**23686.0617**	**26318.2269**	**20959.9522**
**Incidence**	**China**	**Male**	**80-84**	**Tracheal, bronchus, and lung cancer**	**Rate**	**2000**	**448.311815**	**504.598998**	**395.6974**
**Incidence**	**China**	**Female**	**80-84**	**Tracheal, bronchus, and lung cancer**	**Rate**	**2000**	**185.329764**	**211.375894**	**158.064083**
**Incidence**	**China**	**Both**	**80-84**	**Tracheal, bronchus, and lung cancer**	**Rate**	**2000**	**291.560531**	**323.96083**	**258.003837**
**Incidence**	**China**	**Male**	**80-84**	**Tracheal, bronchus, and lung cancer**	**Number**	**2010**	**35242.3467**	**39179.0602**	**31117.1853**
**Incidence**	**China**	**Female**	**80-84**	**Tracheal, bronchus, and lung cancer**	**Number**	**2010**	**17991.8999**	**20236.8412**	**15403.7414**
**Incidence**	**China**	**Both**	**80-84**	**Tracheal, bronchus, and lung cancer**	**Number**	**2010**	**53234.2466**	**58343.1587**	**47102.3988**
**Incidence**	**China**	**Male**	**80-84**	**Tracheal, bronchus, and lung cancer**	**Rate**	**2010**	**661.20759**	**735.067169**	**583.812404**
**Incidence**	**China**	**Female**	**80-84**	**Tracheal, bronchus, and lung cancer**	**Rate**	**2010**	**247.399233**	**278.268499**	**211.810527**
**Incidence**	**China**	**Both**	**80-84**	**Tracheal, bronchus, and lung cancer**	**Rate**	**2010**	**422.413135**	**462.952294**	**373.756994**
**Incidence**	**China**	**Male**	**80-84**	**Tracheal, bronchus, and lung cancer**	**Number**	**2019**	**52068.9173**	**64775.497**	**40711.2693**
**Incidence**	**China**	**Female**	**80-84**	**Tracheal, bronchus, and lung cancer**	**Number**	**2019**	**28787.9445**	**34852.9244**	**22747.5016**
**Incidence**	**China**	**Both**	**80-84**	**Tracheal, bronchus, and lung cancer**	**Number**	**2019**	**80856.8619**	**94766.9283**	**67717.3419**
**Incidence**	**China**	**Male**	**80-84**	**Tracheal, bronchus, and lung cancer**	**Rate**	**2019**	**632.596864**	**786.971928**	**494.610271**
**Incidence**	**China**	**Female**	**80-84**	**Tracheal, bronchus, and lung cancer**	**Rate**	**2019**	**271.387284**	**328.562551**	**214.443329**

Inciden ce	China	Both	80-84	Tracheal, bronchus, and lung cancer	Rate	2019	429.20 6672	503.04 4479	359.45 9102
Inciden ce	China	Male	80-84	Tracheal, bronchus, and lung cancer	Numbe r	2020	53258. 5549	67004. 6447	41098. 7588
Inciden ce	China	Female	80-84	Tracheal, bronchus, and lung cancer	Numbe r	2020	30115. 3123	37680. 1608	23449. 8948
Inciden ce	China	Both	80-84	Tracheal, bronchus, and lung cancer	Numbe r	2020	83373. 8672	99632. 8426	68142. 6351
Inciden ce	China	Male	80-84	Tracheal, bronchus, and lung cancer	Rate	2020	629.83 7125	792.39 8758	486.03 5044
Inciden ce	China	Female	80-84	Tracheal, bronchus, and lung cancer	Rate	2020	277.21 416	346.84 9271	215.85 8392
Inciden ce	China	Both	80-84	Tracheal, bronchus, and lung cancer	Rate	2020	431.55 3375	515.71 1828	352.71 4647
Inciden ce	China	Male	80-84	Tracheal, bronchus, and lung cancer	Numbe r	2021	54317. 1234	67963. 4326	40930. 3113
Inciden ce	China	Female	80-84	Tracheal, bronchus, and lung cancer	Numbe r	2021	31055. 2979	37796. 1043	23879. 7526
Inciden ce	China	Both	80-84	Tracheal, bronchus, and lung cancer	Numbe r	2021	85372. 4214	101518 .634	70010. 291
Inciden ce	China	Male	80-84	Tracheal, bronchus, and lung cancer	Rate	2021	626.13 5779	783.44 2387	471.82 05
Inciden ce	China	Female	80-84	Tracheal, bronchus, and lung cancer	Rate	2021	279.35 2622	339.98 8393	214.80 6231
Inciden ce	China	Both	80-84	Tracheal, bronchus, and lung cancer	Rate	2021	431.35 1267	512.93 135	353.73 2824
Inciden ce	China	Male	85-89	Tracheal, bronchus, and lung cancer	Numbe r	1990	2638.0 0946	3135.4 6369	2208.8 2974
Inciden ce	China	Female	85-89	Tracheal, bronchus, and lung cancer	Numbe r	1990	1971.5 1338	2381.9 277	1632.6 5218
Inciden ce	China	Both	85-89	Tracheal, bronchus, and lung cancer	Numbe r	1990	4609.5 2284	5269.7 1507	3959.4 7422
Inciden ce	China	Male	85-89	Tracheal, bronchus, and lung cancer	Rate	1990	480.84 5798	571.51 9762	402.61 6637
Inciden ce	China	Female	85-89	Tracheal, bronchus, and lung cancer	Rate	1990	173.20 7639	209.26 4658	143.43 6932
Inciden ce	China	Both	85-89	Tracheal, bronchus, and lung cancer	Rate	1990	273.26 1264	312.39 8713	234.72 5148
Inciden ce	China	Male	85-89	Tracheal, bronchus, and lung cancer	Numbe r	2000	6589.0 4925	7347.8 2737	5880.0 0696
Inciden ce	China	Female	85-89	Tracheal, bronchus, and lung cancer	Numbe r	2000	4165.1 4529	4882.0 5591	3381.6 3902
Inciden ce	China	Both	85-89	Tracheal, bronchus, and lung cancer	Numbe r	2000	10754. 1945	11907. 6525	9379.6 5038

Incidence	China	Male	85-89	Tracheal, bronchus, and lung cancer	Rate	2000	660.450577	736.506383	589.380022
Incidence	China	Female	85-89	Tracheal, bronchus, and lung cancer	Rate	2000	216.678868	253.973937	175.919365
Incidence	China	Both	85-89	Tracheal, bronchus, and lung cancer	Rate	2000	368.303642	407.806625	321.229022
Incidence	China	Male	85-89	Tracheal, bronchus, and lung cancer	Number	2010	14519.3243	15949.6999	12768.4988
Incidence	China	Female	85-89	Tracheal, bronchus, and lung cancer	Number	2010	7907.86601	9072.58855	6427.40313
Incidence	China	Both	85-89	Tracheal, bronchus, and lung cancer	Number	2010	22427.1903	24633.4375	19641.6804
Incidence	China	Male	85-89	Tracheal, bronchus, and lung cancer	Rate	2010	862.060102	946.986211	758.107825
Incidence	China	Female	85-89	Tracheal, bronchus, and lung cancer	Rate	2010	250.549274	287.451819	203.642953
Incidence	China	Both	85-89	Tracheal, bronchus, and lung cancer	Rate	2010	463.326628	508.905814	405.780368
Incidence	China	Male	85-89	Tracheal, bronchus, and lung cancer	Number	2019	27353.6576	32844.5557	21731.4159
Incidence	China	Female	85-89	Tracheal, bronchus, and lung cancer	Number	2019	15438.0893	19591.5358	11541.6486
Incidence	China	Both	85-89	Tracheal, bronchus, and lung cancer	Number	2019	42791.7469	49414.2	36095.6427
Incidence	China	Male	85-89	Tracheal, bronchus, and lung cancer	Rate	2019	882.474971	1059.62057	701.092004
Incidence	China	Female	85-89	Tracheal, bronchus, and lung cancer	Rate	2019	284.969929	361.637922	213.045974
Incidence	China	Both	85-89	Tracheal, bronchus, and lung cancer	Rate	2019	502.421706	580.176517	423.802151
Incidence	China	Male	85-89	Tracheal, bronchus, and lung cancer	Number	2020	29226.3315	35469.4002	22926.6858
Incidence	China	Female	85-89	Tracheal, bronchus, and lung cancer	Number	2020	16584.026	21152.4345	12379.262
Incidence	China	Both	85-89	Tracheal, bronchus, and lung cancer	Number	2020	45810.3574	53228.6341	37986.3767
Incidence	China	Male	85-89	Tracheal, bronchus, and lung cancer	Rate	2020	887.520458	1077.10468	696.218159
Incidence	China	Female	85-89	Tracheal, bronchus, and lung cancer	Rate	2020	288.910614	368.496941	215.659346
Incidence	China	Both	85-89	Tracheal, bronchus, and lung cancer	Rate	2020	507.131834	589.253967	420.518458
Incidence	China	Male	85-89	Tracheal, bronchus, and lung cancer	Number	2021	31071.5345	37242.8995	23452.3317

Incidence	China	Female	85-89	Tracheal, bronchus, and lung cancer	Number	2021	17707.8072	22404.197	12824.741
Incidence	China	Both	85-89	Tracheal, bronchus, and lung cancer	Number	2021	48779.3418	57116.5169	40268.5615
Incidence	China	Male	85-89	Tracheal, bronchus, and lung cancer	Rate	2021	892.625399	1069.91684	673.740364
Incidence	China	Female	85-89	Tracheal, bronchus, and lung cancer	Rate	2021	292.941085	370.63368	212.160291
Incidence	China	Both	85-89	Tracheal, bronchus, and lung cancer	Rate	2021	512.078696	599.601193	422.733718
Incidence	China	Male	90-94	Tracheal, bronchus, and lung cancer	Number	1990	544.732606	650.903909	461.965835
Incidence	China	Female	90-94	Tracheal, bronchus, and lung cancer	Number	1990	421.66956	514.129378	337.693187
Incidence	China	Both	90-94	Tracheal, bronchus, and lung cancer	Number	1990	966.402166	1106.03226	829.26178
Incidence	China	Male	90-94	Tracheal, bronchus, and lung cancer	Rate	1990	668.555182	798.860169	566.974786
Incidence	China	Female	90-94	Tracheal, bronchus, and lung cancer	Rate	1990	187.12344	228.154145	149.857416
Incidence	China	Both	90-94	Tracheal, bronchus, and lung cancer	Rate	1990	314.971527	360.48002	270.274487
Incidence	China	Male	90-94	Tracheal, bronchus, and lung cancer	Number	2000	1449.09085	1652.52633	1263.75215
Incidence	China	Female	90-94	Tracheal, bronchus, and lung cancer	Number	2000	1129.99862	1352.59675	923.290991
Incidence	China	Both	90-94	Tracheal, bronchus, and lung cancer	Number	2000	2579.08947	2920.79722	2203.42023
Incidence	China	Male	90-94	Tracheal, bronchus, and lung cancer	Rate	2000	860.50514	981.310045	750.446548
Incidence	China	Female	90-94	Tracheal, bronchus, and lung cancer	Rate	2000	236.734959	283.369316	193.429665
Incidence	China	Both	90-94	Tracheal, bronchus, and lung cancer	Rate	2000	399.408961	452.327302	341.231196
Incidence	China	Male	90-94	Tracheal, bronchus, and lung cancer	Number	2010	3199.91393	3570.02452	2741.968
Incidence	China	Female	90-94	Tracheal, bronchus, and lung cancer	Number	2010	2155.87359	2535.48842	1685.60516
Incidence	China	Both	90-94	Tracheal, bronchus, and lung cancer	Number	2010	5355.78752	5967.66746	4538.26764
Incidence	China	Male	90-94	Tracheal, bronchus, and lung cancer	Rate	2010	996.870687	1112.17141	854.206579
Incidence	China	Female	90-94	Tracheal, bronchus, and lung cancer	Rate	2010	230.965545	271.63488	180.584203

Incidence	China	Both	90-94	Tracheal, bronchus, and lung cancer	Rate	2010	426.955431	475.733592	361.78396
Incidence	China	Male	90-94	Tracheal, bronchus, and lung cancer	Number	2019	6445.12305	7831.49664	5175.24907
Incidence	China	Female	90-94	Tracheal, bronchus, and lung cancer	Number	2019	5066.96569	6105.48942	3809.21944
Incidence	China	Both	90-94	Tracheal, bronchus, and lung cancer	Number	2019	11512.0887	13323.8173	9429.15743
Incidence	China	Male	90-94	Tracheal, bronchus, and lung cancer	Rate	2019	910.151709	1105.92924	730.825734
Incidence	China	Female	90-94	Tracheal, bronchus, and lung cancer	Rate	2019	273.050741	329.015136	205.272791
Incidence	China	Both	90-94	Tracheal, bronchus, and lung cancer	Rate	2019	449.020293	519.685392	367.777137
Incidence	China	Male	90-94	Tracheal, bronchus, and lung cancer	Number	2020	6947.34195	8554.05458	5419.58174
Incidence	China	Female	90-94	Tracheal, bronchus, and lung cancer	Number	2020	5537.22879	6848.23107	4109.27874
Incidence	China	Both	90-94	Tracheal, bronchus, and lung cancer	Number	2020	12484.5707	14787.1603	9929.43267
Incidence	China	Male	90-94	Tracheal, bronchus, and lung cancer	Rate	2020	917.02045	1129.09988	715.36241
Incidence	China	Female	90-94	Tracheal, bronchus, and lung cancer	Rate	2020	278.644716	344.617042	206.787339
Incidence	China	Both	90-94	Tracheal, bronchus, and lung cancer	Rate	2020	454.844491	538.733653	361.754348
Incidence	China	Male	90-94	Tracheal, bronchus, and lung cancer	Number	2021	7457.83929	9239.82725	5621.31475
Incidence	China	Female	90-94	Tracheal, bronchus, and lung cancer	Number	2021	5997.32882	7406.87953	4479.36348
Incidence	China	Both	90-94	Tracheal, bronchus, and lung cancer	Number	2021	13455.1681	16030.3402	10769.6352
Incidence	China	Male	90-94	Tracheal, bronchus, and lung cancer	Rate	2021	922.047423	1142.3629	694.989336
Incidence	China	Female	90-94	Tracheal, bronchus, and lung cancer	Rate	2021	282.472782	348.862289	210.97697
Incidence	China	Both	90-94	Tracheal, bronchus, and lung cancer	Rate	2021	458.909465	546.739719	367.315181
Incidence	China	Male	2-4 years	Tracheal, bronchus, and lung cancer	Number	1990	0	0	0
Incidence	China	Female	2-4 years	Tracheal, bronchus, and lung cancer	Number	1990	0	0	0
Incidence	China	Both	2-4 years	Tracheal, bronchus, and lung cancer	Number	1990	0	0	0

Inciden ce	China	Male	2-4 years	Tracheal, bronchus, and lung cancer	Rate	1990	0	0	0
Inciden ce	China	Female	2-4 years	Tracheal, bronchus, and lung cancer	Rate	1990	0	0	0
Inciden ce	China	Both	2-4 years	Tracheal, bronchus, and lung cancer	Rate	1990	0	0	0
Inciden ce	China	Male	2-4 years	Tracheal, bronchus, and lung cancer	Numbe r	2000	0	0	0
Inciden ce	China	Female	2-4 years	Tracheal, bronchus, and lung cancer	Numbe r	2000	0	0	0
Inciden ce	China	Both	2-4 years	Tracheal, bronchus, and lung cancer	Numbe r	2000	0	0	0
Inciden ce	China	Male	2-4 years	Tracheal, bronchus, and lung cancer	Rate	2000	0	0	0
Inciden ce	China	Female	2-4 years	Tracheal, bronchus, and lung cancer	Rate	2000	0	0	0
Inciden ce	China	Both	2-4 years	Tracheal, bronchus, and lung cancer	Rate	2000	0	0	0
Inciden ce	China	Male	2-4 years	Tracheal, bronchus, and lung cancer	Numbe r	2010	0	0	0
Inciden ce	China	Female	2-4 years	Tracheal, bronchus, and lung cancer	Numbe r	2010	0	0	0
Inciden ce	China	Both	2-4 years	Tracheal, bronchus, and lung cancer	Numbe r	2010	0	0	0
Inciden ce	China	Male	2-4 years	Tracheal, bronchus, and lung cancer	Rate	2010	0	0	0
Inciden ce	China	Female	2-4 years	Tracheal, bronchus, and lung cancer	Rate	2010	0	0	0
Inciden ce	China	Both	2-4 years	Tracheal, bronchus, and lung cancer	Rate	2010	0	0	0
Inciden ce	China	Male	2-4 years	Tracheal, bronchus, and lung cancer	Numbe r	2019	0	0	0
Inciden ce	China	Female	2-4 years	Tracheal, bronchus, and lung cancer	Numbe r	2019	0	0	0
Inciden ce	China	Both	2-4 years	Tracheal, bronchus, and lung cancer	Numbe r	2019	0	0	0
Inciden ce	China	Male	2-4 years	Tracheal, bronchus, and lung cancer	Rate	2019	0	0	0
Inciden ce	China	Female	2-4 years	Tracheal, bronchus, and lung cancer	Rate	2019	0	0	0
Inciden ce	China	Both	2-4 years	Tracheal, bronchus, and lung cancer	Rate	2019	0	0	0
Inciden ce	China	Male	2-4 years	Tracheal, bronchus, and lung cancer	Numbe r	2020	0	0	0

Incidence	China	Female	2-4 years	Tracheal, bronchus, and lung cancer	Number	2020	0	0	0
Incidence	China	Both	2-4 years	Tracheal, bronchus, and lung cancer	Number	2020	0	0	0
Incidence	China	Male	2-4 years	Tracheal, bronchus, and lung cancer	Rate	2020	0	0	0
Incidence	China	Female	2-4 years	Tracheal, bronchus, and lung cancer	Rate	2020	0	0	0
Incidence	China	Both	2-4 years	Tracheal, bronchus, and lung cancer	Rate	2020	0	0	0
Incidence	China	Male	2-4 years	Tracheal, bronchus, and lung cancer	Number	2021	0	0	0
Incidence	China	Female	2-4 years	Tracheal, bronchus, and lung cancer	Number	2021	0	0	0
Incidence	China	Both	2-4 years	Tracheal, bronchus, and lung cancer	Number	2021	0	0	0
Incidence	China	Male	2-4 years	Tracheal, bronchus, and lung cancer	Rate	2021	0	0	0
Incidence	China	Female	2-4 years	Tracheal, bronchus, and lung cancer	Rate	2021	0	0	0
Incidence	China	Both	2-4 years	Tracheal, bronchus, and lung cancer	Rate	2021	0	0	0
Incidence	China	Male	95+ years	Tracheal, bronchus, and lung cancer	Number	1990	24.2591528	29.7817921	19.7572038
Incidence	China	Female	95+ years	Tracheal, bronchus, and lung cancer	Number	1990	65.8379831	79.788325	51.9963819
Incidence	China	Both	95+ years	Tracheal, bronchus, and lung cancer	Number	1990	90.0971359	106.769833	72.4410652
Incidence	China	Male	95+ years	Tracheal, bronchus, and lung cancer	Rate	1990	312.245635	383.328909	254.299921
Incidence	China	Female	95+ years	Tracheal, bronchus, and lung cancer	Rate	1990	201.198908	243.830736	158.899389
Incidence	China	Both	95+ years	Tracheal, bronchus, and lung cancer	Rate	1990	222.505543	263.680743	178.901786
Incidence	China	Male	95+ years	Tracheal, bronchus, and lung cancer	Number	2000	43.3950242	55.4555542	33.5800989
Incidence	China	Female	95+ years	Tracheal, bronchus, and lung cancer	Number	2000	167.499434	206.787635	127.557672
Incidence	China	Both	95+ years	Tracheal, bronchus, and lung cancer	Number	2000	210.894458	256.854791	162.226929
Incidence	China	Male	95+ years	Tracheal, bronchus, and lung cancer	Rate	2000	282.229875	360.668405	218.396171
Incidence	China	Female	95+ years	Tracheal, bronchus, and lung cancer	Rate	2000	239.287385	295.413969	182.227135

Incidence	China	Both	95+ years	Tracheal, bronchus, and lung cancer	Rate	2000	247.02119	300.85464	190.016795
Incidence	China	Male	95+ years	Tracheal, bronchus, and lung cancer	Number	2010	123.977059	140.962149	97.353772
Incidence	China	Female	95+ years	Tracheal, bronchus, and lung cancer	Number	2010	361.412271	428.00081	278.116198
Incidence	China	Both	95+ years	Tracheal, bronchus, and lung cancer	Number	2010	485.389331	565.229026	382.886153
Incidence	China	Male	95+ years	Tracheal, bronchus, and lung cancer	Rate	2010	417.827814	475.07101	328.101941
Incidence	China	Female	95+ years	Tracheal, bronchus, and lung cancer	Rate	2010	221.113002	261.852049	170.152239
Incidence	China	Both	95+ years	Tracheal, bronchus, and lung cancer	Rate	2010	251.336631	292.67796	198.260055
Incidence	China	Male	95+ years	Tracheal, bronchus, and lung cancer	Number	2019	562.048574	661.835227	439.734167
Incidence	China	Female	95+ years	Tracheal, bronchus, and lung cancer	Number	2019	1028.09946	1273.43301	727.566747
Incidence	China	Both	95+ years	Tracheal, bronchus, and lung cancer	Number	2019	1590.14804	1909.19649	1225.97652
Incidence	China	Male	95+ years	Tracheal, bronchus, and lung cancer	Rate	2019	536.64121	631.917014	419.856016
Incidence	China	Female	95+ years	Tracheal, bronchus, and lung cancer	Rate	2019	236.052809	292.38167	167.050154
Incidence	China	Both	95+ years	Tracheal, bronchus, and lung cancer	Rate	2019	294.323376	353.376632	226.918212
Incidence	China	Male	95+ years	Tracheal, bronchus, and lung cancer	Number	2020	609.440186	723.45427	470.654973
Incidence	China	Female	95+ years	Tracheal, bronchus, and lung cancer	Number	2020	1177.75136	1500.94409	810.830272
Incidence	China	Both	95+ years	Tracheal, bronchus, and lung cancer	Number	2020	1787.19154	2156.44652	1338.52015
Incidence	China	Male	95+ years	Tracheal, bronchus, and lung cancer	Rate	2020	540.601587	641.737344	417.492694
Incidence	China	Female	95+ years	Tracheal, bronchus, and lung cancer	Rate	2020	247.275908	315.132146	170.238642
Incidence	China	Both	95+ years	Tracheal, bronchus, and lung cancer	Rate	2020	303.415701	366.104985	227.243706
Incidence	China	Male	95+ years	Tracheal, bronchus, and lung cancer	Number	2021	653.881846	780.511594	491.190489
Incidence	China	Female	95+ years	Tracheal, bronchus, and lung cancer	Number	2021	1295.51311	1637.32643	911.187495
Incidence	China	Both	95+ years	Tracheal, bronchus, and lung cancer	Number	2021	1949.39496	2363.859	1458.71207

Incidence	China	Male	95+ years	Tracheal, bronchus, and lung cancer	Rate	2021	540.649842	645.351255	406.131569
Incidence	China	Female	95+ years	Tracheal, bronchus, and lung cancer	Rate	2021	250.024355	315.991773	175.852382
Incidence	China	Both	95+ years	Tracheal, bronchus, and lung cancer	Rate	2021	305.022638	369.873999	228.245283
Incidence	China	Male	5-9 years	Tracheal, bronchus, and lung cancer	Number	1990	0	0	0
Incidence	China	Female	5-9 years	Tracheal, bronchus, and lung cancer	Number	1990	0	0	0
Incidence	China	Both	5-9 years	Tracheal, bronchus, and lung cancer	Number	1990	0	0	0
Incidence	China	Male	5-9 years	Tracheal, bronchus, and lung cancer	Rate	1990	0	0	0
Incidence	China	Female	5-9 years	Tracheal, bronchus, and lung cancer	Rate	1990	0	0	0
Incidence	China	Both	5-9 years	Tracheal, bronchus, and lung cancer	Rate	1990	0	0	0
Incidence	China	Male	5-9 years	Tracheal, bronchus, and lung cancer	Number	2000	0	0	0
Incidence	China	Female	5-9 years	Tracheal, bronchus, and lung cancer	Number	2000	0	0	0
Incidence	China	Both	5-9 years	Tracheal, bronchus, and lung cancer	Number	2000	0	0	0
Incidence	China	Male	5-9 years	Tracheal, bronchus, and lung cancer	Rate	2000	0	0	0
Incidence	China	Female	5-9 years	Tracheal, bronchus, and lung cancer	Rate	2000	0	0	0
Incidence	China	Both	5-9 years	Tracheal, bronchus, and lung cancer	Rate	2000	0	0	0
Incidence	China	Male	5-9 years	Tracheal, bronchus, and lung cancer	Number	2010	0	0	0
Incidence	China	Female	5-9 years	Tracheal, bronchus, and lung cancer	Number	2010	0	0	0
Incidence	China	Both	5-9 years	Tracheal, bronchus, and lung cancer	Number	2010	0	0	0
Incidence	China	Male	5-9 years	Tracheal, bronchus, and lung cancer	Rate	2010	0	0	0
Incidence	China	Female	5-9 years	Tracheal, bronchus, and lung cancer	Rate	2010	0	0	0
Incidence	China	Both	5-9 years	Tracheal, bronchus, and lung cancer	Rate	2010	0	0	0
Incidence	China	Male	5-9 years	Tracheal, bronchus, and lung cancer	Number	2019	0	0	0

Inciden ce	China	Female	5-9 years	Tracheal, bronchus, and lung cancer	Numbe r	2019	0	0	0
Inciden ce	China	Both	5-9 years	Tracheal, bronchus, and lung cancer	Numbe r	2019	0	0	0
Inciden ce	China	Male	5-9 years	Tracheal, bronchus, and lung cancer	Rate	2019	0	0	0
Inciden ce	China	Female	5-9 years	Tracheal, bronchus, and lung cancer	Rate	2019	0	0	0
Inciden ce	China	Both	5-9 years	Tracheal, bronchus, and lung cancer	Rate	2019	0	0	0
Inciden ce	China	Male	5-9 years	Tracheal, bronchus, and lung cancer	Numbe r	2020	0	0	0
Inciden ce	China	Female	5-9 years	Tracheal, bronchus, and lung cancer	Numbe r	2020	0	0	0
Inciden ce	China	Both	5-9 years	Tracheal, bronchus, and lung cancer	Numbe r	2020	0	0	0
Inciden ce	China	Male	5-9 years	Tracheal, bronchus, and lung cancer	Rate	2020	0	0	0
Inciden ce	China	Female	5-9 years	Tracheal, bronchus, and lung cancer	Rate	2020	0	0	0
Inciden ce	China	Both	5-9 years	Tracheal, bronchus, and lung cancer	Rate	2020	0	0	0
Inciden ce	China	Male	5-9 years	Tracheal, bronchus, and lung cancer	Numbe r	2021	0	0	0
Inciden ce	China	Female	5-9 years	Tracheal, bronchus, and lung cancer	Numbe r	2021	0	0	0
Inciden ce	China	Both	5-9 years	Tracheal, bronchus, and lung cancer	Numbe r	2021	0	0	0
Inciden ce	China	Male	5-9 years	Tracheal, bronchus, and lung cancer	Rate	2021	0	0	0
Inciden ce	China	Female	5-9 years	Tracheal, bronchus, and lung cancer	Rate	2021	0	0	0
Inciden ce	China	Both	5-9 years	Tracheal, bronchus, and lung cancer	Rate	2021	0	0	0
Inciden ce	China	Male	10-14 years	Tracheal, bronchus, and lung cancer	Numbe r	1990	0	0	0
Inciden ce	China	Female	10-14 years	Tracheal, bronchus, and lung cancer	Numbe r	1990	0	0	0
Inciden ce	China	Both	10-14 years	Tracheal, bronchus, and lung cancer	Numbe r	1990	0	0	0
Inciden ce	China	Male	10-14 years	Tracheal, bronchus, and lung cancer	Rate	1990	0	0	0
Inciden ce	China	Female	10-14 years	Tracheal, bronchus, and lung cancer	Rate	1990	0	0	0

Incidence	China	Both	10-14 years	Tracheal, bronchus, and lung cancer	Rate	1990	0	0	0
Incidence	China	Male	10-14 years	Tracheal, bronchus, and lung cancer	Number	2000	0	0	0
Incidence	China	Female	10-14 years	Tracheal, bronchus, and lung cancer	Number	2000	0	0	0
Incidence	China	Both	10-14 years	Tracheal, bronchus, and lung cancer	Number	2000	0	0	0
Incidence	China	Male	10-14 years	Tracheal, bronchus, and lung cancer	Rate	2000	0	0	0
Incidence	China	Female	10-14 years	Tracheal, bronchus, and lung cancer	Rate	2000	0	0	0
Incidence	China	Both	10-14 years	Tracheal, bronchus, and lung cancer	Rate	2000	0	0	0
Incidence	China	Male	10-14 years	Tracheal, bronchus, and lung cancer	Number	2010	0	0	0
Incidence	China	Female	10-14 years	Tracheal, bronchus, and lung cancer	Number	2010	0	0	0
Incidence	China	Both	10-14 years	Tracheal, bronchus, and lung cancer	Number	2010	0	0	0
Incidence	China	Male	10-14 years	Tracheal, bronchus, and lung cancer	Rate	2010	0	0	0
Incidence	China	Female	10-14 years	Tracheal, bronchus, and lung cancer	Rate	2010	0	0	0
Incidence	China	Both	10-14 years	Tracheal, bronchus, and lung cancer	Rate	2010	0	0	0
Incidence	China	Male	10-14 years	Tracheal, bronchus, and lung cancer	Number	2019	0	0	0
Incidence	China	Female	10-14 years	Tracheal, bronchus, and lung cancer	Number	2019	0	0	0
Incidence	China	Both	10-14 years	Tracheal, bronchus, and lung cancer	Number	2019	0	0	0
Incidence	China	Male	10-14 years	Tracheal, bronchus, and lung cancer	Rate	2019	0	0	0
Incidence	China	Female	10-14 years	Tracheal, bronchus, and lung cancer	Rate	2019	0	0	0
Incidence	China	Both	10-14 years	Tracheal, bronchus, and lung cancer	Rate	2019	0	0	0
Incidence	China	Male	10-14 years	Tracheal, bronchus, and lung cancer	Number	2020	0	0	0
Incidence	China	Female	10-14 years	Tracheal, bronchus, and lung cancer	Number	2020	0	0	0
Incidence	China	Both	10-14 years	Tracheal, bronchus, and lung cancer	Number	2020	0	0	0

Incidence	China	Male	10-14 years	Tracheal, bronchus, and lung cancer	Rate	2020	0	0	0
Incidence	China	Female	10-14 years	Tracheal, bronchus, and lung cancer	Rate	2020	0	0	0
Incidence	China	Both	10-14 years	Tracheal, bronchus, and lung cancer	Rate	2020	0	0	0
Incidence	China	Male	10-14 years	Tracheal, bronchus, and lung cancer	Number	2021	0	0	0
Incidence	China	Female	10-14 years	Tracheal, bronchus, and lung cancer	Number	2021	0	0	0
Incidence	China	Both	10-14 years	Tracheal, bronchus, and lung cancer	Number	2021	0	0	0
Incidence	China	Male	10-14 years	Tracheal, bronchus, and lung cancer	Rate	2021	0	0	0
Incidence	China	Female	10-14 years	Tracheal, bronchus, and lung cancer	Rate	2021	0	0	0
Incidence	China	Both	10-14 years	Tracheal, bronchus, and lung cancer	Rate	2021	0	0	0
Incidence	China	Male	15-19 years	Tracheal, bronchus, and lung cancer	Number	1990	353.586069	441.145302	277.401944
Incidence	China	Female	15-19 years	Tracheal, bronchus, and lung cancer	Number	1990	196.869056	238.638692	162.609219
Incidence	China	Both	15-19 years	Tracheal, bronchus, and lung cancer	Number	1990	550.455125	660.204484	461.830287
Incidence	China	Male	15-19 years	Tracheal, bronchus, and lung cancer	Rate	1990	0.54371568	0.67835709	0.42656597
Incidence	China	Female	15-19 years	Tracheal, bronchus, and lung cancer	Rate	1990	0.31941748	0.38718817	0.26383134
Incidence	China	Both	15-19 years	Tracheal, bronchus, and lung cancer	Rate	1990	0.43457485	0.52122008	0.36460706
Incidence	China	Male	15-19 years	Tracheal, bronchus, and lung cancer	Number	2000	239.540619	277.250647	206.396131
Incidence	China	Female	15-19 years	Tracheal, bronchus, and lung cancer	Number	2000	156.021694	183.758169	133.909308
Incidence	China	Both	15-19 years	Tracheal, bronchus, and lung cancer	Number	2000	395.562314	446.334786	353.208991
Incidence	China	Male	15-19 years	Tracheal, bronchus, and lung cancer	Rate	2000	0.44954689	0.52031746	0.38734449
Incidence	China	Female	15-19 years	Tracheal, bronchus, and lung cancer	Rate	2000	0.30842021	0.36324905	0.26470893
Incidence	China	Both	15-19 years	Tracheal, bronchus, and lung cancer	Rate	2000	0.38081605	0.42969577	0.34004163
Incidence	China	Male	15-19 years	Tracheal, bronchus, and lung cancer	Number	2010	168.997181	196.314712	145.774259

Incidence	China	Female	15-19 years	Tracheal, bronchus, and lung cancer	Number	2010	99.4227935	112.986897	86.9402667
Incidence	China	Both	15-19 years	Tracheal, bronchus, and lung cancer	Number	2010	268.419974	300.105422	240.745845
Incidence	China	Male	15-19 years	Tracheal, bronchus, and lung cancer	Rate	2010	0.32476571	0.37726243	0.2801377
Incidence	China	Female	15-19 years	Tracheal, bronchus, and lung cancer	Rate	2010	0.20682339	0.23504	0.18085673
Incidence	China	Both	15-19 years	Tracheal, bronchus, and lung cancer	Rate	2010	0.26813041	0.29978168	0.24048614
Incidence	China	Male	15-19 years	Tracheal, bronchus, and lung cancer	Number	2019	122.750031	162.737848	90.4120393
Incidence	China	Female	15-19 years	Tracheal, bronchus, and lung cancer	Number	2019	80.4404677	98.3367218	65.8298144
Incidence	China	Both	15-19 years	Tracheal, bronchus, and lung cancer	Number	2019	203.190499	244.671321	167.223722
Incidence	China	Male	15-19 years	Tracheal, bronchus, and lung cancer	Rate	2019	0.31597162	0.41890451	0.2327302
Incidence	China	Female	15-19 years	Tracheal, bronchus, and lung cancer	Rate	2019	0.23996778	0.29335539	0.19638168
Incidence	China	Both	15-19 years	Tracheal, bronchus, and lung cancer	Rate	2019	0.28076699	0.33808485	0.23106839
Incidence	China	Male	15-19 years	Tracheal, bronchus, and lung cancer	Number	2020	122.861839	162.683296	88.73298
Incidence	China	Female	15-19 years	Tracheal, bronchus, and lung cancer	Number	2020	82.4739411	101.129214	67.2733307
Incidence	China	Both	15-19 years	Tracheal, bronchus, and lung cancer	Number	2020	205.33578	250.814502	167.120918
Incidence	China	Male	15-19 years	Tracheal, bronchus, and lung cancer	Rate	2020	0.31308881	0.41456583	0.22611824
Incidence	China	Female	15-19 years	Tracheal, bronchus, and lung cancer	Rate	2020	0.24387827	0.29904255	0.19892955
Incidence	China	Both	15-19 years	Tracheal, bronchus, and lung cancer	Rate	2020	0.28105275	0.34330162	0.22874627
Incidence	China	Male	15-19 years	Tracheal, bronchus, and lung cancer	Number	2021	123.948575	163.062111	86.277001
Incidence	China	Female	15-19 years	Tracheal, bronchus, and lung cancer	Number	2021	85.6556683	105.511504	69.1361812
Incidence	China	Both	15-19 years	Tracheal, bronchus, and lung cancer	Number	2021	209.604244	254.337617	167.375244
Incidence	China	Male	15-19 years	Tracheal, bronchus, and lung cancer	Rate	2021	0.30923124	0.40681305	0.21524688
Incidence	China	Female	15-19 years	Tracheal, bronchus, and lung cancer	Rate	2021	0.24763601	0.3050405	0.19987712

Inciden ce	China	Both	15-19 years	Tracheal, bronchus, and lung cancer	Rate	2021	0.2806 9934	0.3406 057	0.2241 468
Inciden ce	China	Male	20-24 years	Tracheal, bronchus, and lung cancer	Numbe r	1990	487.12 7006	599.59 1235	385.57 8119
Inciden ce	China	Female	20-24 years	Tracheal, bronchus, and lung cancer	Numbe r	1990	301.55 0545	395.38 4729	226.43 4143
Inciden ce	China	Both	20-24 years	Tracheal, bronchus, and lung cancer	Numbe r	1990	788.67 7551	934.61 0692	659.91 5737
Inciden ce	China	Male	20-24 years	Tracheal, bronchus, and lung cancer	Rate	1990	0.7215 2023	0.8880 994	0.5711 0858
Inciden ce	China	Female	20-24 years	Tracheal, bronchus, and lung cancer	Rate	1990	0.4676 1499	0.6131 2383	0.3511 3185
Inciden ce	China	Both	20-24 years	Tracheal, bronchus, and lung cancer	Rate	1990	0.5974 7889	0.7080 3354	0.4999 3273
Inciden ce	China	Male	20-24 years	Tracheal, bronchus, and lung cancer	Numbe r	2000	333.56 4702	383.06 0056	287.65 9813
Inciden ce	China	Female	20-24 years	Tracheal, bronchus, and lung cancer	Numbe r	2000	217.77 1388	258.61 6152	180.09 8601
Inciden ce	China	Both	20-24 years	Tracheal, bronchus, and lung cancer	Numbe r	2000	551.33 609	621.87 6043	484.69 5938
Inciden ce	China	Male	20-24 years	Tracheal, bronchus, and lung cancer	Rate	2000	0.6910 6317	0.7936 0524	0.5959 5964
Inciden ce	China	Female	20-24 years	Tracheal, bronchus, and lung cancer	Rate	2000	0.4644 6424	0.5515 7822	0.3841 1547
Inciden ce	China	Both	20-24 years	Tracheal, bronchus, and lung cancer	Rate	2000	0.5794 0894	0.6535 4064	0.5093 7562
Inciden ce	China	Male	20-24 years	Tracheal, bronchus, and lung cancer	Numbe r	2010	388.26 791	451.16 205	328.92 3656
Inciden ce	China	Female	20-24 years	Tracheal, bronchus, and lung cancer	Numbe r	2010	248.56 2519	297.37 4871	207.69 7855
Inciden ce	China	Both	20-24 years	Tracheal, bronchus, and lung cancer	Numbe r	2010	636.83 043	720.00 1269	553.69 3971
Inciden ce	China	Male	20-24 years	Tracheal, bronchus, and lung cancer	Rate	2010	0.6045 463	0.7024 7461	0.5121 4528
Inciden ce	China	Female	20-24 years	Tracheal, bronchus, and lung cancer	Rate	2010	0.3897 7116	0.4663 1386	0.3256 9124
Inciden ce	China	Both	20-24 years	Tracheal, bronchus, and lung cancer	Rate	2010	0.4975 3903	0.5625 1824	0.4325 8668
Inciden ce	China	Male	20-24 years	Tracheal, bronchus, and lung cancer	Numbe r	2019	280.90 7869	365.23 72	213.76 7096
Inciden ce	China	Female	20-24 years	Tracheal, bronchus, and lung cancer	Numbe r	2019	168.32 3512	212.60 2239	127.33 0369
Inciden ce	China	Both	20-24 years	Tracheal, bronchus, and lung cancer	Numbe r	2019	449.23 1381	534.69 9895	364.64 2573

Incidence	China	Male	20-24 years	Tracheal, bronchus, and lung cancer	Rate	2019	0.68153147	0.88612913	0.51863625
Incidence	China	Female	20-24 years	Tracheal, bronchus, and lung cancer	Rate	2019	0.45568304	0.57555378	0.34470698
Incidence	China	Both	20-24 years	Tracheal, bronchus, and lung cancer	Rate	2019	0.57478898	0.68414545	0.46655808
Incidence	China	Male	20-24 years	Tracheal, bronchus, and lung cancer	Number	2020	271.459583	351.879689	203.897304
Incidence	China	Female	20-24 years	Tracheal, bronchus, and lung cancer	Number	2020	162.422871	212.02291	123.604809
Incidence	China	Both	20-24 years	Tracheal, bronchus, and lung cancer	Number	2020	433.882454	530.749312	348.021373
Incidence	China	Male	20-24 years	Tracheal, bronchus, and lung cancer	Rate	2020	0.68113064	0.8829161	0.51160729
Incidence	China	Female	20-24 years	Tracheal, bronchus, and lung cancer	Rate	2020	0.45824055	0.5981762	0.34872389
Incidence	China	Both	20-24 years	Tracheal, bronchus, and lung cancer	Rate	2020	0.57621159	0.70485429	0.46218497
Incidence	China	Male	20-24 years	Tracheal, bronchus, and lung cancer	Number	2021	264.987106	343.985647	193.977138
Incidence	China	Female	20-24 years	Tracheal, bronchus, and lung cancer	Number	2021	161.372424	214.937551	117.936028
Incidence	China	Both	20-24 years	Tracheal, bronchus, and lung cancer	Number	2021	426.359531	524.149917	330.82809
Incidence	China	Male	20-24 years	Tracheal, bronchus, and lung cancer	Rate	2021	0.68177033	0.8850212	0.4990728
Incidence	China	Female	20-24 years	Tracheal, bronchus, and lung cancer	Rate	2021	0.47038229	0.62651855	0.34377013
Incidence	China	Both	20-24 years	Tracheal, bronchus, and lung cancer	Rate	2021	0.58266408	0.71630468	0.45211055
Incidence	China	Male	25-29 years	Tracheal, bronchus, and lung cancer	Number	1990	786.039589	944.361186	643.055753
Incidence	China	Female	25-29 years	Tracheal, bronchus, and lung cancer	Number	1990	516.695618	682.971565	388.714455
Incidence	China	Both	25-29 years	Tracheal, bronchus, and lung cancer	Number	1990	1302.73521	1542.12466	1113.62265
Incidence	China	Male	25-29 years	Tracheal, bronchus, and lung cancer	Rate	1990	1.39303257	1.67361276	1.13963421
Incidence	China	Female	25-29 years	Tracheal, bronchus, and lung cancer	Rate	1990	0.96645544	1.27746697	0.72707255
Incidence	China	Both	25-29 years	Tracheal, bronchus, and lung cancer	Rate	1990	1.18549607	1.40334177	1.01340263
Incidence	China	Male	25-29 years	Tracheal, bronchus, and lung cancer	Number	2000	879.291734	1008.42064	757.06331

Incidence	China	Female	25-29 years	Tracheal, bronchus, and lung cancer	Number	2000	531.127001	637.542588	429.021171
Incidence	China	Both	25-29 years	Tracheal, bronchus, and lung cancer	Number	2000	1410.41873	1595.71883	1240.84365
Incidence	China	Male	25-29 years	Tracheal, bronchus, and lung cancer	Rate	2000	1.4516942	1.66488361	1.24989736
Incidence	China	Female	25-29 years	Tracheal, bronchus, and lung cancer	Rate	2000	0.92237735	1.1071831	0.74505609
Incidence	China	Both	25-29 years	Tracheal, bronchus, and lung cancer	Rate	2000	1.19372801	1.35055939	1.05020572
Incidence	China	Male	25-29 years	Tracheal, bronchus, and lung cancer	Number	2010	689.080143	780.68002	610.819327
Incidence	China	Female	25-29 years	Tracheal, bronchus, and lung cancer	Number	2010	479.670878	561.103382	407.01843
Incidence	China	Both	25-29 years	Tracheal, bronchus, and lung cancer	Number	2010	1168.75102	1303.23439	1065.65232
Incidence	China	Male	25-29 years	Tracheal, bronchus, and lung cancer	Rate	2010	1.34894091	1.52825651	1.1957378
Incidence	China	Female	25-29 years	Tracheal, bronchus, and lung cancer	Rate	2010	0.94844233	1.10945697	0.80478829
Incidence	China	Both	25-29 years	Tracheal, bronchus, and lung cancer	Rate	2010	1.14969319	1.28198366	1.04827563
Incidence	China	Male	25-29 years	Tracheal, bronchus, and lung cancer	Number	2019	756.878265	926.59245	597.215555
Incidence	China	Female	25-29 years	Tracheal, bronchus, and lung cancer	Number	2019	453.806979	582.42913	338.619715
Incidence	China	Both	25-29 years	Tracheal, bronchus, and lung cancer	Number	2019	1210.68524	1444.51688	988.953315
Incidence	China	Male	25-29 years	Tracheal, bronchus, and lung cancer	Rate	2019	1.45240046	1.77807101	1.14601804
Incidence	China	Female	25-29 years	Tracheal, bronchus, and lung cancer	Rate	2019	0.94127978	1.20806596	0.70236004
Incidence	China	Both	25-29 years	Tracheal, bronchus, and lung cancer	Rate	2019	1.20677614	1.43985277	0.98576015
Incidence	China	Male	25-29 years	Tracheal, bronchus, and lung cancer	Number	2020	706.860822	891.07945	542.688502
Incidence	China	Female	25-29 years	Tracheal, bronchus, and lung cancer	Number	2020	426.58816	564.624213	318.418273
Incidence	China	Both	25-29 years	Tracheal, bronchus, and lung cancer	Number	2020	1133.44898	1363.50299	928.735552
Incidence	China	Male	25-29 years	Tracheal, bronchus, and lung cancer	Rate	2020	1.45783811	1.83777278	1.11924718
Incidence	China	Female	25-29 years	Tracheal, bronchus, and lung cancer	Rate	2020	0.96737687	1.28040216	0.72207928

Inciden ce	China	Both	25-29 years	Tracheal, bronchus, and lung cancer	Rate	2020	1.2242 341	1.4727 146	1.0031 2387
Inciden ce	China	Male	25-29 years	Tracheal, bronchus, and lung cancer	Numbe r	2021	666.59 6827	848.91 8979	494.73 7867
Inciden ce	China	Female	25-29 years	Tracheal, bronchus, and lung cancer	Numbe r	2021	402.15 7327	531.03 0736	295.50 5895
Inciden ce	China	Both	25-29 years	Tracheal, bronchus, and lung cancer	Numbe r	2021	1068.7 5415	1292.5 9835	860.85 7417
Inciden ce	China	Male	25-29 years	Tracheal, bronchus, and lung cancer	Rate	2021	1.4613 3084	1.8610 2219	1.0845 7718
Inciden ce	China	Female	25-29 years	Tracheal, bronchus, and lung cancer	Rate	2021	0.9840 7976	1.2994 3325	0.7231 035
Inciden ce	China	Both	25-29 years	Tracheal, bronchus, and lung cancer	Rate	2021	1.2358 1009	1.4946 4315	0.9954 1721
Inciden ce	China	Male	30-34 years	Tracheal, bronchus, and lung cancer	Numbe r	1990	1620.9 7807	1952.0 0893	1324.1 7158
Inciden ce	China	Female	30-34 years	Tracheal, bronchus, and lung cancer	Numbe r	1990	1029.2 9624	1331.9 2779	784.41 7901
Inciden ce	China	Both	30-34 years	Tracheal, bronchus, and lung cancer	Numbe r	1990	2650.2 743	3100.4 1684	2247.5 9045
Inciden ce	China	Male	30-34 years	Tracheal, bronchus, and lung cancer	Rate	1990	3.5219 6892	4.2412 1394	2.8770 8468
Inciden ce	China	Female	30-34 years	Tracheal, bronchus, and lung cancer	Rate	1990	2.4379 6103	3.1547 6529	1.8579 4935
Inciden ce	China	Both	30-34 years	Tracheal, bronchus, and lung cancer	Rate	1990	3.0033 3693	3.5134 4628	2.5470 0858
Inciden ce	China	Male	30-34 years	Tracheal, bronchus, and lung cancer	Numbe r	2000	2454.0 611	2814.5 1629	2077.9 4433
Inciden ce	China	Female	30-34 years	Tracheal, bronchus, and lung cancer	Numbe r	2000	1420.0 156	1733.5 4122	1111.4 5898
Inciden ce	China	Both	30-34 years	Tracheal, bronchus, and lung cancer	Numbe r	2000	3874.0 767	4384.3 5554	3327.3 582
Inciden ce	China	Male	30-34 years	Tracheal, bronchus, and lung cancer	Rate	2000	3.7251 8623	4.2723 4567	3.1542 53
Inciden ce	China	Female	30-34 years	Tracheal, bronchus, and lung cancer	Rate	2000	2.2757 124	2.7781 6754	1.7812 2056
Inciden ce	China	Both	30-34 years	Tracheal, bronchus, and lung cancer	Rate	2000	3.0201 0396	3.4179 0071	2.5939 0003
Inciden ce	China	Male	30-34 years	Tracheal, bronchus, and lung cancer	Numbe r	2010	1826.0 4205	2061.4 3674	1618.1 6731
Inciden ce	China	Female	30-34 years	Tracheal, bronchus, and lung cancer	Numbe r	2010	1095.6 6723	1280.7 3126	941.00 4457
Inciden ce	China	Both	30-34 years	Tracheal, bronchus, and lung cancer	Numbe r	2010	2921.7 0927	3226.2 3162	2648.4 8475

Incidence	China	Male	30-34 years	Tracheal, bronchus, and lung cancer	Rate	2010	3.67435904	4.14801988	3.25607381
Incidence	China	Female	30-34 years	Tracheal, bronchus, and lung cancer	Rate	2010	2.28524077	2.67123012	1.96265955
Incidence	China	Both	30-34 years	Tracheal, bronchus, and lung cancer	Rate	2010	2.99225895	3.30413451	2.71243695
Incidence	China	Male	30-34 years	Tracheal, bronchus, and lung cancer	Number	2019	2272.10885	2801.8029	1808.74258
Incidence	China	Female	30-34 years	Tracheal, bronchus, and lung cancer	Number	2019	1343.56884	1715.96439	1011.75491
Incidence	China	Both	30-34 years	Tracheal, bronchus, and lung cancer	Number	2019	3615.67769	4258.64706	2976.75032
Incidence	China	Male	30-34 years	Tracheal, bronchus, and lung cancer	Rate	2019	3.57062066	4.40303523	2.84244023
Incidence	China	Female	30-34 years	Tracheal, bronchus, and lung cancer	Rate	2019	2.2036068	2.81437816	1.65939395
Incidence	China	Both	30-34 years	Tracheal, bronchus, and lung cancer	Rate	2019	2.90171636	3.41772329	2.38895329
Incidence	China	Male	30-34 years	Tracheal, bronchus, and lung cancer	Number	2020	2312.95872	2874.57179	1826.82397
Incidence	China	Female	30-34 years	Tracheal, bronchus, and lung cancer	Number	2020	1380.99453	1818.25077	1040.42642
Incidence	China	Both	30-34 years	Tracheal, bronchus, and lung cancer	Number	2020	3693.95325	4449.45263	3048.4613
Incidence	China	Male	30-34 years	Tracheal, bronchus, and lung cancer	Rate	2020	3.60790972	4.48395184	2.8496038
Incidence	China	Female	30-34 years	Tracheal, bronchus, and lung cancer	Rate	2020	2.27672696	2.99759373	1.7152616
Incidence	China	Both	30-34 years	Tracheal, bronchus, and lung cancer	Rate	2020	2.96072853	3.5662664	2.44336237
Incidence	China	Male	30-34 years	Tracheal, bronchus, and lung cancer	Number	2021	2286.87592	2891.13655	1727.12212
Incidence	China	Female	30-34 years	Tracheal, bronchus, and lung cancer	Number	2021	1363.17264	1828.58315	1005.03282
Incidence	China	Both	30-34 years	Tracheal, bronchus, and lung cancer	Number	2021	3650.04856	4395.81235	2911.01145
Incidence	China	Male	30-34 years	Tracheal, bronchus, and lung cancer	Rate	2021	3.64827296	4.61225518	2.75529287
Incidence	China	Female	30-34 years	Tracheal, bronchus, and lung cancer	Rate	2021	2.33142576	3.1274145	1.71890144
Incidence	China	Both	30-34 years	Tracheal, bronchus, and lung cancer	Rate	2021	3.01275258	3.62830653	2.40275085
Incidence	China	Male	35-39 years	Tracheal, bronchus, and lung cancer	Number	1990	4077.91964	4963.02843	3285.62041

Incidence	China	Female	35-39 years	Tracheal, bronchus, and lung cancer	Number	1990	2476.87471	3125.42327	1932.56455
Incidence	China	Both	35-39 years	Tracheal, bronchus, and lung cancer	Number	1990	6554.79435	7589.40247	5555.22918
Incidence	China	Male	35-39 years	Tracheal, bronchus, and lung cancer	Rate	1990	8.63488514	10.5090792	6.95721283
Incidence	China	Female	35-39 years	Tracheal, bronchus, and lung cancer	Rate	1990	5.61484656	7.08504632	4.38094561
Incidence	China	Both	35-39 years	Tracheal, bronchus, and lung cancer	Rate	1990	7.17633246	8.3090441	6.08198661
Incidence	China	Male	35-39 years	Tracheal, bronchus, and lung cancer	Number	2000	4873.04665	5578.18282	4249.29768
Incidence	China	Female	35-39 years	Tracheal, bronchus, and lung cancer	Number	2000	3007.10055	3507.76522	2531.32319
Incidence	China	Both	35-39 years	Tracheal, bronchus, and lung cancer	Number	2000	7880.1472	8829.1184	7004.02534
Incidence	China	Male	35-39 years	Tracheal, bronchus, and lung cancer	Rate	2000	8.62643688	9.87469349	7.52225473
Incidence	China	Female	35-39 years	Tracheal, bronchus, and lung cancer	Rate	2000	5.63191389	6.56959463	4.74084387
Incidence	China	Both	35-39 years	Tracheal, bronchus, and lung cancer	Rate	2000	7.17135763	8.03497244	6.37403963
Incidence	China	Male	35-39 years	Tracheal, bronchus, and lung cancer	Number	2010	4450.15012	5070.32744	3868.99221
Incidence	China	Female	35-39 years	Tracheal, bronchus, and lung cancer	Number	2010	2779.0155	3210.92651	2420.75256
Incidence	China	Both	35-39 years	Tracheal, bronchus, and lung cancer	Number	2010	7229.16563	8051.66103	6490.40563
Incidence	China	Male	35-39 years	Tracheal, bronchus, and lung cancer	Rate	2010	7.34076105	8.36377676	6.38210993
Incidence	China	Female	35-39 years	Tracheal, bronchus, and lung cancer	Rate	2010	4.79291664	5.53782557	4.17502716
Incidence	China	Both	35-39 years	Tracheal, bronchus, and lung cancer	Rate	2010	6.09520294	6.7886822	5.4723244
Incidence	China	Male	35-39 years	Tracheal, bronchus, and lung cancer	Number	2019	3876.75091	4907.00173	2996.72096
Incidence	China	Female	35-39 years	Tracheal, bronchus, and lung cancer	Number	2019	2356.14516	2962.21597	1788.77904
Incidence	China	Both	35-39 years	Tracheal, bronchus, and lung cancer	Number	2019	6232.89607	7364.58955	5120.54743
Incidence	China	Male	35-39 years	Tracheal, bronchus, and lung cancer	Rate	2019	7.90587271	10.0068671	6.11122432
Incidence	China	Female	35-39 years	Tracheal, bronchus, and lung cancer	Rate	2019	5.07757786	6.38368235	3.8548834

Incidence	China	Both	35-39 years	Tracheal, bronchus, and lung cancer	Rate	2019	6.53074515	7.71651846	5.36524112
Incidence	China	Male	35-39 years	Tracheal, bronchus, and lung cancer	Number	2020	4046.06595	5162.11867	3101.57082
Incidence	China	Female	35-39 years	Tracheal, bronchus, and lung cancer	Number	2020	2486.23858	3257.96964	1902.32741
Incidence	China	Both	35-39 years	Tracheal, bronchus, and lung cancer	Number	2020	6532.30452	7853.38577	5316.27252
Incidence	China	Male	35-39 years	Tracheal, bronchus, and lung cancer	Rate	2020	7.89960676	10.0786067	6.05555869
Incidence	China	Female	35-39 years	Tracheal, bronchus, and lung cancer	Rate	2020	5.12424891	6.71482115	3.92078188
Incidence	China	Both	35-39 years	Tracheal, bronchus, and lung cancer	Rate	2020	6.54948664	7.87404277	5.33025607
Incidence	China	Male	35-39 years	Tracheal, bronchus, and lung cancer	Number	2021	4317.4565	5604.26408	3247.46046
Incidence	China	Female	35-39 years	Tracheal, bronchus, and lung cancer	Number	2021	2691.60625	3581.12171	1987.13229
Incidence	China	Both	35-39 years	Tracheal, bronchus, and lung cancer	Number	2021	7009.06275	8630.54289	5587.06924
Incidence	China	Male	35-39 years	Tracheal, bronchus, and lung cancer	Rate	2021	7.94144888	10.3083787	5.97331815
Incidence	China	Female	35-39 years	Tracheal, bronchus, and lung cancer	Rate	2021	5.21657932	6.94054171	3.85124431
Incidence	China	Both	35-39 years	Tracheal, bronchus, and lung cancer	Rate	2021	6.61461627	8.14484497	5.27264778
Incidence	China	Male	40-44 years	Tracheal, bronchus, and lung cancer	Number	1990	6742.23005	8319.52753	5346.60115
Incidence	China	Female	40-44 years	Tracheal, bronchus, and lung cancer	Number	1990	3666.41344	4606.06367	2850.76067
Incidence	China	Both	40-44 years	Tracheal, bronchus, and lung cancer	Number	1990	10408.6435	12073.4931	8764.10339
Incidence	China	Male	40-44 years	Tracheal, bronchus, and lung cancer	Rate	1990	19.1649226	23.648422	15.1978198
Incidence	China	Female	40-44 years	Tracheal, bronchus, and lung cancer	Rate	1990	11.4883156	14.4326095	8.93255465
Incidence	China	Both	40-44 years	Tracheal, bronchus, and lung cancer	Rate	1990	15.513446	17.9948025	13.0623596
Incidence	China	Male	40-44 years	Tracheal, bronchus, and lung cancer	Number	2000	8233.63776	9512.12119	7083.53465
Incidence	China	Female	40-44 years	Tracheal, bronchus, and lung cancer	Number	2000	4801.19065	5525.04847	4125.99563
Incidence	China	Both	40-44 years	Tracheal, bronchus, and lung cancer	Number	2000	13034.8284	14491.147	11640.1322

Incidence	China	Male	40-44 years	Tracheal, bronchus, and lung cancer	Rate	2000	19.2926781	22.2883612	16.5978098
Incidence	China	Female	40-44 years	Tracheal, bronchus, and lung cancer	Rate	2000	12.1763271	14.0121071	10.4639612
Incidence	China	Both	40-44 years	Tracheal, bronchus, and lung cancer	Rate	2000	15.875212	17.648873	14.1766014
Incidence	China	Male	40-44 years	Tracheal, bronchus, and lung cancer	Number	2010	10961.8333	12657.9941	9445.25871
Incidence	China	Female	40-44 years	Tracheal, bronchus, and lung cancer	Number	2010	6542.25168	7535.46481	5701.63048
Incidence	China	Both	40-44 years	Tracheal, bronchus, and lung cancer	Number	2010	17504.085	19696.9324	15711.3492
Incidence	China	Male	40-44 years	Tracheal, bronchus, and lung cancer	Rate	2010	17.1658934	19.822029	14.7909844
Incidence	China	Female	40-44 years	Tracheal, bronchus, and lung cancer	Rate	2010	10.6406165	12.2560237	9.27339187
Incidence	China	Both	40-44 years	Tracheal, bronchus, and lung cancer	Rate	2010	13.9650617	15.7145533	12.5347861
Incidence	China	Male	40-44 years	Tracheal, bronchus, and lung cancer	Number	2019	8372.03202	10848.0181	6346.41687
Incidence	China	Female	40-44 years	Tracheal, bronchus, and lung cancer	Number	2019	5232.79608	6668.1728	3976.49335
Incidence	China	Both	40-44 years	Tracheal, bronchus, and lung cancer	Number	2019	13604.8281	16288.8393	11049.5575
Incidence	China	Male	40-44 years	Tracheal, bronchus, and lung cancer	Rate	2019	16.9527859	21.9664864	12.8510553
Incidence	China	Female	40-44 years	Tracheal, bronchus, and lung cancer	Rate	2019	11.0529269	14.0847885	8.39931261
Incidence	China	Both	40-44 years	Tracheal, bronchus, and lung cancer	Rate	2019	14.0651112	16.8399288	11.4233899
Incidence	China	Male	40-44 years	Tracheal, bronchus, and lung cancer	Number	2020	8123.72891	10490.706	6159.06173
Incidence	China	Female	40-44 years	Tracheal, bronchus, and lung cancer	Number	2020	5131.34205	6609.48026	3919.10869
Incidence	China	Both	40-44 years	Tracheal, bronchus, and lung cancer	Number	2020	13255.071	16137.5434	10584.8204
Incidence	China	Male	40-44 years	Tracheal, bronchus, and lung cancer	Rate	2020	16.9562698	21.8967476	12.8555142
Incidence	China	Female	40-44 years	Tracheal, bronchus, and lung cancer	Rate	2020	11.2221757	14.454844	8.57103772
Incidence	China	Both	40-44 years	Tracheal, bronchus, and lung cancer	Rate	2020	14.156122	17.2345387	11.3043536
Incidence	China	Male	40-44 years	Tracheal, bronchus, and lung cancer	Number	2021	7976.70937	10520.1907	5879.17709

Incidence	China	Female	40-44 years	Tracheal, bronchus, and lung cancer	Number	2021	5110.06976	6767.89489	3852.69712
Incidence	China	Both	40-44 years	Tracheal, bronchus, and lung cancer	Number	2021	13086.7791	16145.4704	10265.8357
Incidence	China	Male	40-44 years	Tracheal, bronchus, and lung cancer	Rate	2021	16.9987367	22.419013	12.5287984
Incidence	China	Female	40-44 years	Tracheal, bronchus, and lung cancer	Rate	2021	11.4554037	15.1718023	8.63671199
Incidence	China	Both	40-44 years	Tracheal, bronchus, and lung cancer	Rate	2021	14.2972275	17.6388293	11.2153638
Incidence	China	Male	45-49 years	Tracheal, bronchus, and lung cancer	Number	1990	9476.20517	11755.4635	7328.50882

Appendix 23：Age burden of larynx cancer in China from 1990 to 2021

measure	location	sex	age	cause	metric	year	val	upper	lower
Deaths	China	Male	20-24 years	Larynx cancer	Number	1990	27.9473907	34.0298173	22.2964237
Deaths	China	Female	20-24 years	Larynx cancer	Number	1990	14.375272	19.8707715	4.76786268
Deaths	China	Both	20-24 years	Larynx cancer	Number	1990	42.3226627	50.1345745	30.5981469
Deaths	China	Male	20-24 years	Larynx cancer	Rate	1990	0.04139497	0.05040411	0.0330249
Deaths	China	Female	20-24 years	Larynx cancer	Rate	1990	0.02229176	0.03081364	0.00739353
Deaths	China	Both	20-24 years	Larynx cancer	Rate	1990	0.0320624	0.03798048	0.02318026
Deaths	China	Male	25-29 years	Larynx cancer	Number	1990	36.3955967	45.4887994	28.8706416
Deaths	China	Female	25-29 years	Larynx cancer	Number	1990	17.2252349	24.3438712	6.30159088
Deaths	China	Both	25-29 years	Larynx cancer	Number	1990	53.6208316	63.7749084	39.0619663
Deaths	China	Male	25-29 years	Larynx cancer	Rate	1990	0.06450089	0.08061601	0.05116504
Deaths	China	Female	25-29 years	Larynx cancer	Rate	1990	0.03221901	0.04553409	0.01178684
Deaths	China	Both	25-29 years	Larynx cancer	Rate	1990	0.04879525	0.05803551	0.0355466
Deaths	China	Male	30-34 years	Larynx cancer	Number	1990	56.7899993	70.0888789	45.3477801
Deaths	China	Female	30-34 years	Larynx cancer	Number	1990	20.1428079	27.6671143	7.48249457
Deaths	China	Both	30-34 years	Larynx cancer	Number	1990	76.9328072	91.081707	59.5331488
Deaths	China	Male	30-34 years	Larynx cancer	Rate	1990	0.12339008	0.15228513	0.09852908
Deaths	China	Female	30-34 years	Larynx cancer	Rate	1990	0.04770967	0.06553152	0.01772282
Deaths	China	Both	30-34 years	Larynx cancer	Rate	1990	0.0871816	0.10321537	0.067464
Deaths	China	Male	35-39 years	Larynx cancer	Number	1990	158.678474	193.182878	126.1493
Deaths	China	Female	35-39 years	Larynx cancer	Number	1990	43.7316881	59.3634863	19.7004049

Deaths	China	Both	35-39 years	Larynx cancer	Number	1990	202.410162	241.375237	163.637196
Deaths	China	Male	35-39 years	Larynx cancer	Rate	1990	0.3359974	0.40905955	0.26711775
Deaths	China	Female	35-39 years	Larynx cancer	Rate	1990	0.0991357	0.13457155	0.044659
Deaths	China	Both	35-39 years	Larynx cancer	Rate	1990	0.22160308	0.2642629	0.17915359
Deaths	China	Male	40-44 years	Larynx cancer	Number	1990	327.09004	410.005973	253.06432
Deaths	China	Female	40-44 years	Larynx cancer	Number	1990	56.8220785	76.3094746	25.5250576
Deaths	China	Both	40-44 years	Larynx cancer	Number	1990	383.912119	465.816474	301.635179
Deaths	China	Male	40-44 years	Larynx cancer	Rate	1990	0.92975993	1.16545011	0.71934035
Deaths	China	Female	40-44 years	Larynx cancer	Rate	1990	0.17804593	0.2391076	0.07998005
Deaths	China	Both	40-44 years	Larynx cancer	Rate	1990	0.57219751	0.69427094	0.44956877
Deaths	China	Male	45-49 years	Larynx cancer	Number	1990	521.121349	650.22009	400.397639
Deaths	China	Female	45-49 years	Larynx cancer	Number	1990	80.0420996	106.759916	38.0121915
Deaths	China	Both	45-49 years	Larynx cancer	Number	1990	601.163448	729.918168	475.533789
Deaths	China	Male	45-49 years	Larynx cancer	Rate	1990	1.91315605	2.38710715	1.46995161
Deaths	China	Female	45-49 years	Larynx cancer	Rate	1990	0.32830882	0.43789734	0.15591467
Deaths	China	Both	45-49 years	Larynx cancer	Rate	1990	1.16461761	1.41405062	0.92123868
Deaths	China	Male	50-54 years	Larynx cancer	Number	1990	1060.19594	1325.48068	821.645448
Deaths	China	Female	50-54 years	Larynx cancer	Number	1990	141.070842	187.613992	72.6020011
Deaths	China	Both	50-54 years	Larynx cancer	Number	1990	1201.26678	1460.22432	952.315821
Deaths	China	Male	50-54 years	Larynx cancer	Rate	1990	4.19786662	5.24826675	3.25332126
Deaths	China	Female	50-54 years	Larynx cancer	Rate	1990	0.62822995	0.83550029	0.32331807
Deaths	China	Both	50-54 years	Larynx cancer	Rate	1990	2.5178049	3.06056905	1.9960141

Deaths	China	Male	55-59 years	Larynx cancer	Number	1990	1547.22139	1917.40674	1192.45271
Deaths	China	Female	55-59 years	Larynx cancer	Number	1990	252.474826	339.82008	133.996344
Deaths	China	Both	55-59 years	Larynx cancer	Number	1990	1799.69621	2183.55582	1433.41968
Deaths	China	Male	55-59 years	Larynx cancer	Rate	1990	6.80733273	8.4360427	5.24645177
Deaths	China	Female	55-59 years	Larynx cancer	Rate	1990	1.22320966	1.64638673	0.6491959
Deaths	China	Both	55-59 years	Larynx cancer	Rate	1990	4.14971978	5.03481905	3.30516336
Deaths	China	Male	60-64 years	Larynx cancer	Number	1990	1689.87222	2126.34322	1321.22476
Deaths	China	Female	60-64 years	Larynx cancer	Number	1990	308.495076	398.36978	176.900738
Deaths	China	Both	60-64 years	Larynx cancer	Number	1990	1998.36729	2436.21413	1597.11525
Deaths	China	Male	60-64 years	Larynx cancer	Rate	1990	9.28352429	11.6813323	7.25831338
Deaths	China	Female	60-64 years	Larynx cancer	Rate	1990	1.80042761	2.32495105	1.03242158
Deaths	China	Both	60-64 years	Larynx cancer	Rate	1990	5.65509573	6.89414013	4.51960941
Deaths	China	Male	65-69 years	Larynx cancer	Number	1990	1746.18648	2186.68721	1366.57496
Deaths	China	Female	65-69 years	Larynx cancer	Number	1990	383.75705	485.26372	216.244593
Deaths	China	Both	65-69 years	Larynx cancer	Number	1990	2129.94353	2571.32908	1727.1486
Deaths	China	Male	65-69 years	Larynx cancer	Rate	1990	13.0228793	16.3080885	10.1917756
Deaths	China	Female	65-69 years	Larynx cancer	Rate	1990	2.7661742	3.49784839	1.55872111
Deaths	China	Both	65-69 years	Larynx cancer	Rate	1990	7.80719247	9.42506726	6.33076952
Deaths	China	Male	70-74 years	Larynx cancer	Number	1990	1589.77136	1928.75884	1256.43455
Deaths	China	Female	70-74 years	Larynx cancer	Number	1990	405.222008	514.182315	224.560662
Deaths	China	Both	70-74 years	Larynx cancer	Number	1990	1994.99337	2351.7918	1633.85867
Deaths	China	Male	70-74 years	Larynx cancer	Rate	1990	18.2202134	22.1053155	14.399873

Deaths	China	Female	70-74 years	Larynx cancer	Rate	1990	4.01514663	5.094781	2.22506174
Deaths	China	Both	70-74 years	Larynx cancer	Rate	1990	10.6017132	12.4977969	8.68258572
Deaths	China	Male	75-79 years	Larynx cancer	Number	1990	1058.35593	1267.43862	859.959486
Deaths	China	Female	75-79 years	Larynx cancer	Number	1990	305.993674	384.944862	175.236359
Deaths	China	Both	75-79 years	Larynx cancer	Number	1990	1364.34961	1580.25132	1128.67381
Deaths	China	Male	75-79 years	Larynx cancer	Rate	1990	21.6121538	25.8817261	17.5607998
Deaths	China	Female	75-79 years	Larynx cancer	Rate	1990	4.71946149	5.9371569	2.7027397
Deaths	China	Both	75-79 years	Larynx cancer	Rate	1990	11.988278	13.8853649	9.91744005
Deaths	China	Male	80-84	Larynx cancer	Number	1990	519.286487	626.615984	428.413527
Deaths	China	Female	80-84	Larynx cancer	Number	1990	186.024455	230.422278	120.431905
Deaths	China	Both	80-84	Larynx cancer	Number	1990	705.310942	818.29463	581.058413
Deaths	China	Male	80-84	Larynx cancer	Rate	1990	25.4571072	30.7187472	21.0022201
Deaths	China	Female	80-84	Larynx cancer	Rate	1990	5.71104236	7.07407738	3.69731878
Deaths	China	Both	80-84	Larynx cancer	Rate	1990	13.3149751	15.4478996	10.9693156
Deaths	China	Male	85-89	Larynx cancer	Number	1990	196.105721	235.181796	163.050697
Deaths	China	Female	85-89	Larynx cancer	Number	1990	67.2945093	87.234497	43.4722306
Deaths	China	Both	85-89	Larynx cancer	Number	1990	263.40023	306.325983	219.307882
Deaths	China	Male	85-89	Larynx cancer	Rate	1990	35.7453652	42.8679958	29.720228
Deaths	China	Female	85-89	Larynx cancer	Rate	1990	5.91217043	7.66400139	3.81926013
Deaths	China	Both	85-89	Larynx cancer	Rate	1990	15.6148657	18.1595858	13.0009876
Deaths	China	Male	90-94	Larynx cancer	Number	1990	33.9098997	40.4072308	28.543559
Deaths	China	Female	90-94	Larynx cancer	Number	1990	13.862002	17.9406227	9.73420269

Deaths	China	Both	90-94	Larynx cancer	Number	1990	47.7719017	56.0197558	40.2808806
Deaths	China	Male	90-94	Larynx cancer	Rate	1990	41.6179221	49.5921545	35.0317643
Deaths	China	Female	90-94	Larynx cancer	Rate	1990	6.15151234	7.96147351	4.31972726
Deaths	China	Both	90-94	Larynx cancer	Rate	1990	15.5699039	18.2580593	13.1284169
Deaths	China	Male	95+ years	Larynx cancer	Number	1990	1.64002912	2.19393636	1.26898697
Deaths	China	Female	95+ years	Larynx cancer	Number	1990	1.93378514	2.64480671	1.34564434
Deaths	China	Both	95+ years	Larynx cancer	Number	1990	3.57381426	4.55398165	2.76410463
Deaths	China	Male	95+ years	Larynx cancer	Rate	1990	21.1092258	28.2387047	16.3334493
Deaths	China	Female	95+ years	Larynx cancer	Rate	1990	5.90958958	8.08245025	4.11224887
Deaths	China	Both	95+ years	Larynx cancer	Rate	1990	8.8259574	11.2465968	6.8262836
Deaths	China	Male	20-24 years	Larynx cancer	Number	2000	15.5572185	17.912727	13.0060812
Deaths	China	Female	20-24 years	Larynx cancer	Number	2000	8.22908552	10.2513594	3.73992903
Deaths	China	Both	20-24 years	Larynx cancer	Number	2000	23.786304	26.9646526	18.8118988
Deaths	China	Male	20-24 years	Larynx cancer	Rate	2000	0.03223069	0.03711072	0.02694537
Deaths	China	Female	20-24 years	Larynx cancer	Rate	2000	0.01755105	0.02186417	0.00797655
Deaths	China	Both	20-24 years	Larynx cancer	Rate	2000	0.02499745	0.02833764	0.01976976
Deaths	China	Male	25-29 years	Larynx cancer	Number	2000	30.817195	35.7619889	26.0403557
Deaths	China	Female	25-29 years	Larynx cancer	Number	2000	13.8120347	17.7559652	6.67956801
Deaths	China	Both	25-29 years	Larynx cancer	Number	2000	44.6292297	51.0343082	36.2579463
Deaths	China	Male	25-29 years	Larynx cancer	Rate	2000	0.05087861	0.05904237	0.04299214
Deaths	China	Female	25-29 years	Larynx cancer	Rate	2000	0.02398656	0.03083575	0.01160002
Deaths	China	Both	25-29 years	Larynx cancer	Rate	2000	0.03777259	0.04319361	0.03068743

Deaths	China	Male	30-34 years	Larynx cancer	Number	2000	61.461281	71.3526178	50.9910681
Deaths	China	Female	30-34 years	Larynx cancer	Number	2000	21.224467	27.2492938	10.0317997
Deaths	China	Both	30-34 years	Larynx cancer	Number	2000	82.685748	94.1926975	69.9405422
Deaths	China	Male	30-34 years	Larynx cancer	Rate	2000	0.09329626	0.10831099	0.07740281
Deaths	China	Female	30-34 years	Larynx cancer	Rate	2000	0.03401426	0.04366963	0.01607693
Deaths	China	Both	30-34 years	Larynx cancer	Rate	2000	0.06445911	0.07342956	0.05452337
Deaths	China	Male	35-39 years	Larynx cancer	Number	2000	149.160395	173.395065	127.450123
Deaths	China	Female	35-39 years	Larynx cancer	Number	2000	37.6244268	47.2088309	20.9556523
Deaths	China	Both	35-39 years	Larynx cancer	Number	2000	186.784822	213.043153	161.119047
Deaths	China	Male	35-39 years	Larynx cancer	Rate	2000	0.26404893	0.30694998	0.22561665
Deaths	China	Female	35-39 years	Larynx cancer	Rate	2000	0.07046573	0.08841609	0.03924725
Deaths	China	Both	35-39 years	Larynx cancer	Rate	2000	0.16998423	0.19388072	0.146627
Deaths	China	Male	40-44 years	Larynx cancer	Number	2000	340.510781	411.338185	280.191278
Deaths	China	Female	40-44 years	Larynx cancer	Number	2000	53.1610095	65.9885432	31.6335353
Deaths	China	Both	40-44 years	Larynx cancer	Number	2000	393.67179	467.625427	325.208893
Deaths	China	Male	40-44 years	Larynx cancer	Rate	2000	0.79786907	0.96382856	0.65653121
Deaths	China	Female	40-44 years	Larynx cancer	Rate	2000	0.13482194	0.16735392	0.08022599
Deaths	China	Both	40-44 years	Larynx cancer	Rate	2000	0.47945573	0.5695244	0.39607427
Deaths	China	Male	45-49 years	Larynx cancer	Number	2000	690.304356	811.383523	570.085222
Deaths	China	Female	45-49 years	Larynx cancer	Number	2000	98.2536563	121.669097	55.794836
Deaths	China	Both	45-49 years	Larynx cancer	Number	2000	788.558013	911.111135	661.090259
Deaths	China	Male	45-49 years	Larynx cancer	Rate	2000	1.55914643	1.83262022	1.28761513

Deaths	China	Female	45-49 years	Larynx cancer	Rate	2000	0.23447905	0.29035921	0.1331525
Deaths	China	Both	45-49 years	Larynx cancer	Rate	2000	0.91503972	1.0572499	0.76712662
Deaths	China	Male	50-54 years	Larynx cancer	Number	2000	1084.52168	1270.10398	907.380315
Deaths	China	Female	50-54 years	Larynx cancer	Number	2000	144.596044	179.199023	90.271888
Deaths	China	Both	50-54 years	Larynx cancer	Number	2000	1229.11773	1418.4867	1042.03074
Deaths	China	Male	50-54 years	Larynx cancer	Rate	2000	3.28133342	3.84283199	2.74537374
Deaths	China	Female	50-54 years	Larynx cancer	Rate	2000	0.47069256	0.58333302	0.29385525
Deaths	China	Both	50-54 years	Larynx cancer	Rate	2000	1.92738994	2.22434102	1.63401724
Deaths	China	Male	55-59 years	Larynx cancer	Number	2000	1289.99595	1556.09893	1067.04048
Deaths	China	Female	55-59 years	Larynx cancer	Number	2000	214.195135	272.197919	133.984235
Deaths	China	Both	55-59 years	Larynx cancer	Number	2000	1504.19109	1793.44608	1246.83337
Deaths	China	Male	55-59 years	Larynx cancer	Rate	2000	5.31237368	6.40822087	4.39421361
Deaths	China	Female	55-59 years	Larynx cancer	Rate	2000	0.95247066	1.21039412	0.59579343
Deaths	China	Both	55-59 years	Larynx cancer	Rate	2000	3.21606092	3.83450738	2.66581295
Deaths	China	Male	60-64 years	Larynx cancer	Number	2000	1612.17099	1877.84127	1355.49906
Deaths	China	Female	60-64 years	Larynx cancer	Number	2000	282.505769	341.456681	186.903528
Deaths	China	Both	60-64 years	Larynx cancer	Number	2000	1894.67676	2176.92073	1630.1643
Deaths	China	Male	60-64 years	Larynx cancer	Rate	2000	7.3886074	8.60617886	6.21227554
Deaths	China	Female	60-64 years	Larynx cancer	Rate	2000	1.40096837	1.69331059	0.92686932
Deaths	China	Both	60-64 years	Larynx cancer	Rate	2000	4.51277669	5.18503069	3.88275594
Deaths	China	Male	65-69 years	Larynx cancer	Number	2000	1872.86966	2165.09032	1592.00397
Deaths	China	Female	65-69 years	Larynx cancer	Number	2000	387.984618	467.21886	256.434439

Deaths	China	Both	65-69 years	Larynx cancer	Number	2000	2260.85428	2582.04487	1959.93619
Deaths	China	Male	65-69 years	Larynx cancer	Rate	2000	10.5182888	12.1594394	8.94090915
Deaths	China	Female	65-69 years	Larynx cancer	Rate	2000	2.22446525	2.67874567	1.47023741
Deaths	China	Both	65-69 years	Larynx cancer	Rate	2000	6.41421814	7.32546064	5.56049028
Deaths	China	Male	70-74 years	Larynx cancer	Number	2000	1864.79864	2123.17838	1596.80046
Deaths	China	Female	70-74 years	Larynx cancer	Number	2000	419.467844	506.715763	271.726445
Deaths	China	Both	70-74 years	Larynx cancer	Number	2000	2284.26649	2552.37743	1971.14555
Deaths	China	Male	70-74 years	Larynx cancer	Rate	2000	14.8577806	16.9164209	12.7225055
Deaths	China	Female	70-74 years	Larynx cancer	Rate	2000	3.16393349	3.82202115	2.04955972
Deaths	China	Both	70-74 years	Larynx cancer	Rate	2000	8.85073187	9.88956777	7.637498
Deaths	China	Male	75-79 years	Larynx cancer	Number	2000	1286.68303	1455.73759	1114.80653
Deaths	China	Female	75-79 years	Larynx cancer	Number	2000	334.915789	403.651021	230.932583
Deaths	China	Both	75-79 years	Larynx cancer	Number	2000	1621.59882	1800.98429	1421.44192
Deaths	China	Male	75-79 years	Larynx cancer	Rate	2000	17.6877009	20.0116503	15.3249587
Deaths	China	Female	75-79 years	Larynx cancer	Rate	2000	3.77402528	4.54857372	2.60228224
Deaths	China	Both	75-79 years	Larynx cancer	Rate	2000	10.0416783	11.1525148	8.80221564
Deaths	China	Male	80-84	Larynx cancer	Number	2000	737.367615	890.351939	621.219972
Deaths	China	Female	80-84	Larynx cancer	Number	2000	231.589373	296.553224	166.029296
Deaths	China	Both	80-84	Larynx cancer	Number	2000	968.956989	1141.28019	827.659458
Deaths	China	Male	80-84	Larynx cancer	Rate	2000	22.4696201	27.1314733	18.9302818
Deaths	China	Female	80-84	Larynx cancer	Rate	2000	4.78265996	6.12425867	3.42874828
Deaths	China	Both	80-84	Larynx cancer	Rate	2000	11.9272515	14.0484418	10.1879677

Deaths	China	Male	85-89	Larynx cancer	Number	2000	379.760419	446.59601	324.899793
Deaths	China	Female	85-89	Larynx cancer	Number	2000	99.3719156	132.329537	72.9706201
Deaths	China	Both	85-89	Larynx cancer	Number	2000	479.132335	561.08627	414.140512
Deaths	China	Male	85-89	Larynx cancer	Rate	2000	38.0651257	44.764363	32.566194
Deaths	China	Female	85-89	Larynx cancer	Rate	2000	5.16951815	6.88403702	3.79607199
Deaths	China	Both	85-89	Larynx cancer	Rate	2000	16.4090563	19.2157689	14.1832527
Deaths	China	Male	90-94	Larynx cancer	Number	2000	71.4705672	86.4676659	61.5928782
Deaths	China	Female	90-94	Larynx cancer	Number	2000	27.0415851	37.583175	19.5057125
Deaths	China	Both	90-94	Larynx cancer	Number	2000	98.5121523	118.729316	83.1517636
Deaths	China	Male	90-94	Larynx cancer	Rate	2000	42.4409488	51.3465882	36.5753386
Deaths	China	Female	90-94	Larynx cancer	Rate	2000	5.66521801	7.8736834	4.08645104
Deaths	China	Both	90-94	Larynx cancer	Rate	2000	15.2560184	18.3869358	12.8772421
Deaths	China	Male	95+ years	Larynx cancer	Number	2000	2.48488657	3.45479018	1.8333929
Deaths	China	Female	95+ years	Larynx cancer	Number	2000	3.71108991	5.64108246	2.58188762
Deaths	China	Both	95+ years	Larynx cancer	Number	2000	6.19597647	8.19525571	4.59391233
Deaths	China	Male	95+ years	Larynx cancer	Rate	2000	16.1610516	22.4690508	11.9239074
Deaths	China	Female	95+ years	Larynx cancer	Rate	2000	5.30161195	8.05877275	3.68844911
Deaths	China	Both	95+ years	Larynx cancer	Rate	2000	7.25736227	9.59912289	5.38086065
Deaths	China	Male	20-24 years	Larynx cancer	Number	2010	14.7003041	17.3444496	12.1698919
Deaths	China	Female	20-24 years	Larynx cancer	Number	2010	8.79413537	10.8245646	5.87794583
Deaths	China	Both	20-24 years	Larynx cancer	Number	2010	23.4944395	26.8259741	20.1141258
Deaths	China	Male	20-24 years	Larynx cancer	Rate	2010	0.02288887	0.0270059	0.01894893

**Deaths**	**China**	**Female**	**20-24 years**	**Larynx cancer**	**Rate**	**2010**	**0.01379009**	**0.01697401**	**0.00921721**
**Deaths**	**China**	**Both**	**20-24 years**	**Larynx cancer**	**Rate**	**2010**	**0.01835559**	**0.02095843**	**0.01571464**
**Deaths**	**China**	**Male**	**25-29 years**	**Larynx cancer**	**Number**	**2010**	**21.5422012**	**24.6713747**	**18.7500856**
**Deaths**	**China**	**Female**	**25-29 years**	**Larynx cancer**	**Number**	**2010**	**11.1729764**	**14.1663834**	**7.43884795**
**Deaths**	**China**	**Both**	**25-29 years**	**Larynx cancer**	**Number**	**2010**	**32.7151776**	**36.9847728**	**28.3472046**
**Deaths**	**China**	**Male**	**25-29 years**	**Larynx cancer**	**Rate**	**2010**	**0.04217094**	**0.0482966**	**0.0367051**
**Deaths**	**China**	**Female**	**25-29 years**	**Larynx cancer**	**Rate**	**2010**	**0.02209207**	**0.02801087**	**0.01470867**
**Deaths**	**China**	**Both**	**25-29 years**	**Larynx cancer**	**Rate**	**2010**	**0.03218172**	**0.03638169**	**0.02788497**
**Deaths**	**China**	**Male**	**30-34 years**	**Larynx cancer**	**Number**	**2010**	**38.3439068**	**44.0205622**	**32.858982**
**Deaths**	**China**	**Female**	**30-34 years**	**Larynx cancer**	**Number**	**2010**	**14.1510708**	**17.942773**	**9.24811926**
**Deaths**	**China**	**Both**	**30-34 years**	**Larynx cancer**	**Number**	**2010**	**52.4949777**	**59.4265262**	**46.402648**
**Deaths**	**China**	**Male**	**30-34 years**	**Larynx cancer**	**Rate**	**2010**	**0.07715555**	**0.08857811**	**0.06611879**
**Deaths**	**China**	**Female**	**30-34 years**	**Larynx cancer**	**Rate**	**2010**	**0.02951499**	**0.03742337**	**0.01928887**
**Deaths**	**China**	**Both**	**30-34 years**	**Larynx cancer**	**Rate**	**2010**	**0.05376256**	**0.06086148**	**0.04752312**
**Deaths**	**China**	**Male**	**35-39 years**	**Larynx cancer**	**Number**	**2010**	**116.899368**	**134.861345**	**101.103059**
**Deaths**	**China**	**Female**	**35-39 years**	**Larynx cancer**	**Number**	**2010**	**29.3973946**	**37.0589455**	**19.4712596**
**Deaths**	**China**	**Both**	**35-39 years**	**Larynx cancer**	**Number**	**2010**	**146.296763**	**165.694336**	**127.88104**
**Deaths**	**China**	**Male**	**35-39 years**	**Larynx cancer**	**Rate**	**2010**	**0.19283177**	**0.22246101**	**0.16677491**
**Deaths**	**China**	**Female**	**35-39 years**	**Larynx cancer**	**Rate**	**2010**	**0.05070114**	**0.06391488**	**0.03358172**
**Deaths**	**China**	**Both**	**35-39 years**	**Larynx cancer**	**Rate**	**2010**	**0.12334874**	**0.13970362**	**0.10782169**
**Deaths**	**China**	**Male**	**40-44 years**	**Larynx cancer**	**Number**	**2010**	**348.298735**	**409.109637**	**293.079539**
**Deaths**	**China**	**Female**	**40-44 years**	**Larynx cancer**	**Number**	**2010**	**59.3557017**	**73.0042566**	**40.9716304**

Deaths	China	Both	40-44 years	Larynx cancer	Number	2010	407.654437	469.462389	349.33432
Deaths	China	Male	40-44 years	Larynx cancer	Rate	2010	0.5454251	0.6406531	0.45895354
Deaths	China	Female	40-44 years	Larynx cancer	Rate	2010	0.09653882	0.11873745	0.06663813
Deaths	China	Both	40-44 years	Larynx cancer	Rate	2010	0.32523376	0.37454521	0.27870496
Deaths	China	Male	45-49 years	Larynx cancer	Number	2010	701.28835	830.758931	592.052205
Deaths	China	Female	45-49 years	Larynx cancer	Number	2010	103.331508	127.057514	70.9971761
Deaths	China	Both	45-49 years	Larynx cancer	Number	2010	804.619858	929.563887	691.339114
Deaths	China	Male	45-49 years	Larynx cancer	Rate	2010	1.29646351	1.53581424	1.09451993
Deaths	China	Female	45-49 years	Larynx cancer	Rate	2010	0.19798866	0.24344894	0.13603436
Deaths	China	Both	45-49 years	Larynx cancer	Rate	2010	0.75705393	0.87461177	0.6504699
Deaths	China	Male	50-54 years	Larynx cancer	Number	2010	957.830606	1142.45657	796.288857
Deaths	China	Female	50-54 years	Larynx cancer	Number	2010	122.123287	155.039824	81.2108597
Deaths	China	Both	50-54 years	Larynx cancer	Number	2010	1079.95389	1268.63902	908.934247
Deaths	China	Male	50-54 years	Larynx cancer	Rate	2010	2.35487631	2.80878885	1.95771753
Deaths	China	Female	50-54 years	Larynx cancer	Rate	2010	0.31550759	0.40054803	0.20980964
Deaths	China	Both	50-54 years	Larynx cancer	Rate	2010	1.3604643	1.59815906	1.14502351
Deaths	China	Male	55-59 years	Larynx cancer	Number	2010	1593.65223	1878.6307	1343.43557
Deaths	China	Female	55-59 years	Larynx cancer	Number	2010	242.522767	297.646791	157.448658
Deaths	China	Both	55-59 years	Larynx cancer	Number	2010	1836.175	2140.28424	1571.97295
Deaths	China	Male	55-59 years	Larynx cancer	Rate	2010	3.85629605	4.54588273	3.25082549
Deaths	China	Female	55-59 years	Larynx cancer	Rate	2010	0.59913474	0.73531461	0.38896538
Deaths	China	Both	55-59 years	Larynx cancer	Rate	2010	2.2445805	2.61633031	1.92161414

Deaths	China	Male	60-64 years	Larynx cancer	Number	2010	1908.04589	2226.36294	1619.7428
Deaths	China	Female	60-64 years	Larynx cancer	Number	2010	317.829404	389.158824	205.950987
Deaths	China	Both	60-64 years	Larynx cancer	Number	2010	2225.87529	2557.28166	1916.36602
Deaths	China	Male	60-64 years	Larynx cancer	Rate	2010	6.35917792	7.42007208	5.39831495
Deaths	China	Female	60-64 years	Larynx cancer	Rate	2010	1.09602964	1.34200801	0.7102187
Deaths	China	Both	60-64 years	Larynx cancer	Rate	2010	3.77248783	4.33416642	3.24792117
Deaths	China	Male	65-69 years	Larynx cancer	Number	2010	1820.05598	2128.92248	1559.70101
Deaths	China	Female	65-69 years	Larynx cancer	Number	2010	343.967313	427.177196	227.02262
Deaths	China	Both	65-69 years	Larynx cancer	Number	2010	2164.02329	2512.45864	1857.7205
Deaths	China	Male	65-69 years	Larynx cancer	Rate	2010	8.72520342	10.2058849	7.47708245
Deaths	China	Female	65-69 years	Larynx cancer	Rate	2010	1.68007517	2.08650581	1.10887009
Deaths	China	Both	65-69 years	Larynx cancer	Rate	2010	5.23557194	6.0785658	4.4945123
Deaths	China	Male	70-74 years	Larynx cancer	Number	2010	2006.05966	2315.33927	1712.48431
Deaths	China	Female	70-74 years	Larynx cancer	Number	2010	431.341392	524.650946	277.097916
Deaths	China	Both	70-74 years	Larynx cancer	Number	2010	2437.40105	2767.43377	2095.18437
Deaths	China	Male	70-74 years	Larynx cancer	Rate	2010	12.1313377	14.0016586	10.3559859
Deaths	China	Female	70-74 years	Larynx cancer	Rate	2010	2.58346418	3.14232983	1.65964258
Deaths	China	Both	70-74 years	Larynx cancer	Rate	2010	7.33440755	8.32751229	6.30463998
Deaths	China	Male	75-79 years	Larynx cancer	Number	2010	1742.33065	1990.07571	1497.74329
Deaths	China	Female	75-79 years	Larynx cancer	Number	2010	428.493611	523.782313	275.0952
Deaths	China	Both	75-79 years	Larynx cancer	Number	2010	2170.82426	2458.57081	1883.39603
Deaths	China	Male	75-79 years	Larynx cancer	Rate	2010	15.3948806	17.5839057	13.2337563

Deaths	China	Female	75-79 years	Larynx cancer	Rate	2010	3.38523121	4.13804124	2.17333662
Deaths	China	Both	75-79 years	Larynx cancer	Rate	2010	9.05440708	10.2545846	7.85555727
Deaths	China	Male	80-84	Larynx cancer	Number	2010	1104.0155	1272.13286	947.967935
Deaths	China	Female	80-84	Larynx cancer	Number	2010	310.020542	373.974249	183.961769
Deaths	China	Both	80-84	Larynx cancer	Number	2010	1414.03604	1603.45442	1227.09343
Deaths	China	Male	80-84	Larynx cancer	Rate	2010	20.7132469	23.8674203	17.7855238
Deaths	China	Female	80-84	Larynx cancer	Rate	2010	4.26296527	5.14236644	2.5295828
Deaths	China	Both	80-84	Larynx cancer	Rate	2010	11.2203597	12.7233924	9.73697225
Deaths	China	Male	85-89	Larynx cancer	Number	2010	527.991011	611.478206	428.714592
Deaths	China	Female	85-89	Larynx cancer	Number	2010	136.568656	171.292859	83.2123858
Deaths	China	Both	85-89	Larynx cancer	Number	2010	664.559667	760.888438	553.66015
Deaths	China	Male	85-89	Larynx cancer	Rate	2010	31.3485651	36.3054749	25.4541972
Deaths	China	Female	85-89	Larynx cancer	Rate	2010	4.32697994	5.42716597	2.63646385
Deaths	China	Both	85-89	Larynx cancer	Rate	2010	13.729236	15.7193063	11.4381466
Deaths	China	Male	90-94	Larynx cancer	Number	2010	115.518639	135.226699	89.8216737
Deaths	China	Female	90-94	Larynx cancer	Number	2010	45.3808577	56.6668396	28.2104752
Deaths	China	Both	90-94	Larynx cancer	Number	2010	160.899496	185.412534	132.782073
Deaths	China	Male	90-94	Larynx cancer	Rate	2010	35.9875758	42.1272371	27.9821882
Deaths	China	Female	90-94	Larynx cancer	Rate	2010	4.86179458	6.07089746	3.02227729
Deaths	China	Both	90-94	Larynx cancer	Rate	2010	12.826669	14.780812	10.5851897
Deaths	China	Male	95+ years	Larynx cancer	Number	2010	5.37889892	6.48324765	3.95315977
Deaths	China	Female	95+ years	Larynx cancer	Number	2010	7.56448751	9.61197605	4.4509151

Deaths	China	Both	95+ years	Larynx cancer	Number	2010	12.9433864	15.7242238	9.45900664
Deaths	China	Male	95+ years	Larynx cancer	Rate	2010	18.1279794	21.8498584	13.3229496
Deaths	China	Female	95+ years	Larynx cancer	Rate	2010	4.62797384	5.88063286	2.72308185
Deaths	China	Both	95+ years	Larynx cancer	Rate	2010	6.70213977	8.14206903	4.8979133
Deaths	China	Male	20-24 years	Larynx cancer	Number	2019	9.87701482	12.7917558	7.50087611
Deaths	China	Female	20-24 years	Larynx cancer	Number	2019	4.56428102	6.89528229	2.54712199
Deaths	China	Both	20-24 years	Larynx cancer	Number	2019	14.4412958	18.6477654	11.2297416
Deaths	China	Male	20-24 years	Larynx cancer	Rate	2019	0.02396336	0.03103503	0.01819843
Deaths	China	Female	20-24 years	Larynx cancer	Rate	2019	0.01235636	0.01866681	0.00689553
Deaths	China	Both	20-24 years	Larynx cancer	Rate	2019	0.01847756	0.02385971	0.01436839
Deaths	China	Male	25-29 years	Larynx cancer	Number	2019	20.6674063	25.0542067	16.4824342
Deaths	China	Female	25-29 years	Larynx cancer	Number	2019	7.44850373	10.7741616	4.50372488
Deaths	China	Both	25-29 years	Larynx cancer	Number	2019	28.1159101	33.7095667	23.1035314
Deaths	China	Male	25-29 years	Larynx cancer	Rate	2019	0.03965942	0.0480774	0.03162873
Deaths	China	Female	25-29 years	Larynx cancer	Rate	2019	0.01544958	0.02234761	0.00934156
Deaths	China	Both	25-29 years	Larynx cancer	Rate	2019	0.02802513	0.03360072	0.02302893
Deaths	China	Male	30-34 years	Larynx cancer	Number	2019	43.8898745	54.1840575	34.7946244
Deaths	China	Female	30-34 years	Larynx cancer	Number	2019	13.0026568	19.0506947	7.72085467
Deaths	China	Both	30-34 years	Larynx cancer	Number	2019	56.8925314	69.2374781	46.5859804
Deaths	China	Male	30-34 years	Larynx cancer	Rate	2019	0.06897297	0.08515028	0.05467978
Deaths	China	Female	30-34 years	Larynx cancer	Rate	2019	0.02132585	0.03124532	0.01266309
Deaths	China	Both	30-34 years	Larynx cancer	Rate	2019	0.04565838	0.05556566	0.03738699

Deaths	China	Male	35-39 years	Larynx cancer	Number	2019	89.9568945	112.794939	69.5678596
Deaths	China	Female	35-39 years	Larynx cancer	Number	2019	19.6831941	29.2803624	11.8010277
Deaths	China	Both	35-39 years	Larynx cancer	Number	2019	109.640089	135.973287	87.7673959
Deaths	China	Male	35-39 years	Larynx cancer	Rate	2019	0.18344943	0.23002314	0.14187
Deaths	China	Female	35-39 years	Larynx cancer	Rate	2019	0.04241799	0.06310024	0.02543164
Deaths	China	Both	35-39 years	Larynx cancer	Rate	2019	0.11487942	0.14247099	0.0919615
Deaths	China	Male	40-44 years	Larynx cancer	Number	2019	224.743027	292.036627	167.978275
Deaths	China	Female	40-44 years	Larynx cancer	Number	2019	38.0576482	54.7080244	23.6663628
Deaths	China	Both	40-44 years	Larynx cancer	Number	2019	262.800675	334.63279	204.489059
Deaths	China	Male	40-44 years	Larynx cancer	Rate	2019	0.45508909	0.59135398	0.34014439
Deaths	China	Female	40-44 years	Larynx cancer	Rate	2019	0.08038693	0.11555654	0.04998906
Deaths	China	Both	40-44 years	Larynx cancer	Rate	2019	0.27169184	0.3459542	0.2114074
Deaths	China	Male	45-49 years	Larynx cancer	Number	2019	581.779512	771.016104	421.145663
Deaths	China	Female	45-49 years	Larynx cancer	Number	2019	81.2313325	116.513575	52.4972961
Deaths	China	Both	45-49 years	Larynx cancer	Number	2019	663.010845	865.320367	499.266286
Deaths	China	Male	45-49 years	Larynx cancer	Rate	2019	0.96595529	1.28015351	0.69924752
Deaths	China	Female	45-49 years	Larynx cancer	Rate	2019	0.14036378	0.20132978	0.09071277
Deaths	China	Both	45-49 years	Larynx cancer	Rate	2019	0.56139587	0.73269885	0.42274728
Deaths	China	Male	50-54 years	Larynx cancer	Number	2019	1290.0516	1707.41226	935.545786
Deaths	China	Female	50-54 years	Larynx cancer	Number	2019	156.24819	215.55049	101.394919
Deaths	China	Both	50-54 years	Larynx cancer	Number	2019	1446.29979	1884.91333	1088.92227
Deaths	China	Male	50-54 years	Larynx cancer	Rate	2019	2.09783188	2.77652759	1.52134827

Deaths	China	Female	50-54 years	Larynx cancer	Rate	2019	0.256849 89	0.354334 47	0.166678 88
Deaths	China	Both	50-54 years	Larynx cancer	Rate	2019	1.182322 51	1.540880 72	0.890173 2
Deaths	China	Male	55-59 years	Larynx cancer	Number	2019	1612.899 09	2131.444 6	1176.849
Deaths	China	Female	55-59 years	Larynx cancer	Number	2019	229.1077 62	334.3827 41	142.8353 14
Deaths	China	Both	55-59 years	Larynx cancer	Number	2019	1842.006 85	2413.931 37	1385.862 5
Deaths	China	Male	55-59 years	Larynx cancer	Rate	2019	3.449152 75	4.558052 05	2.516668 27
Deaths	China	Female	55-59 years	Larynx cancer	Rate	2019	0.494372 97	0.721537 27	0.308212 69
Deaths	China	Both	55-59 years	Larynx cancer	Rate	2019	1.978412 83	2.592690 03	1.488489 66
Deaths	China	Male	60-64 years	Larynx cancer	Number	2019	2179.520 07	2952.682 15	1602.052 43
Deaths	China	Female	60-64 years	Larynx cancer	Number	2019	340.6803 96	468.5168 01	207.8547 41
Deaths	China	Both	60-64 years	Larynx cancer	Number	2019	2520.200 47	3289.062 1	1916.729 27
Deaths	China	Male	60-64 years	Larynx cancer	Rate	2019	5.625508 89	7.621099 66	4.135020 5
Deaths	China	Female	60-64 years	Larynx cancer	Rate	2019	0.887452 56	1.220458 93	0.541449 47
Deaths	China	Both	60-64 years	Larynx cancer	Rate	2019	3.267382 4	4.264193 96	2.484995 76
Deaths	China	Male	65-69 years	Larynx cancer	Number	2019	2615.345 74	3491.406 98	1932.883 43
Deaths	China	Female	65-69 years	Larynx cancer	Number	2019	489.1468 56	666.9645 9	294.1273 48
Deaths	China	Both	65-69 years	Larynx cancer	Number	2019	3104.492 6	4012.677 24	2400.001 05
Deaths	China	Male	65-69 years	Larynx cancer	Rate	2019	7.648807 01	10.21092 46	5.652886 38
Deaths	China	Female	65-69 years	Larynx cancer	Rate	2019	1.378579 38	1.879729 21	0.828949 21
Deaths	China	Both	65-69 years	Larynx cancer	Rate	2019	4.455688 72	5.759150 69	3.444575
Deaths	China	Male	70-74 years	Larynx cancer	Number	2019	2570.896 54	3356.487 26	1946.602 2
Deaths	China	Female	70-74 years	Larynx cancer	Number	2019	533.9554 14	740.4217 43	327.5642 11

Deaths	China	Both	70-74 years	Larynx cancer	Number	2019	3104.851 96	3967.381 35	2416.929 46
Deaths	China	Male	70-74 years	Larynx cancer	Rate	2019	11.01096 99	14.3756	8.337160 96
Deaths	China	Female	70-74 years	Larynx cancer	Rate	2019	2.187546 11	3.033411 89	1.341988 11
Deaths	China	Both	70-74 years	Larynx cancer	Rate	2019	6.501303 24	8.307368 46	5.060850 42
Deaths	China	Male	75-79 years	Larynx cancer	Number	2019	1986.644 54	2526.156 15	1549.180 28
Deaths	China	Female	75-79 years	Larynx cancer	Number	2019	479.7394 82	668.3279 11	277.5262 19
Deaths	China	Both	75-79 years	Larynx cancer	Number	2019	2466.384 02	3058.404 56	1989.111 3
Deaths	China	Male	75-79 years	Larynx cancer	Rate	2019	13.82086 65	17.57418 92	10.77747 6
Deaths	China	Female	75-79 years	Larynx cancer	Rate	2019	3.003171 79	4.183736 39	1.737315 65
Deaths	China	Both	75-79 years	Larynx cancer	Rate	2019	8.126828 18	10.07755 81	6.554196 6
Deaths	China	Male	80-84	Larynx cancer	Number	2019	1475.443 03	1810.692 62	1159.657 88
Deaths	China	Female	80-84	Larynx cancer	Number	2019	411.3280 89	564.4238 99	226.9913 01
Deaths	China	Both	80-84	Larynx cancer	Number	2019	1886.771 12	2274.527 63	1534.451 62
Deaths	China	Male	80-84	Larynx cancer	Rate	2019	17.92548 58	21.99850 76	14.08894 17
Deaths	China	Female	80-84	Larynx cancer	Rate	2019	3.877637 48	5.320889 4	2.139873 26
Deaths	China	Both	80-84	Larynx cancer	Rate	2019	10.01541 16	12.07371 17	8.145219 3
Deaths	China	Male	85-89	Larynx cancer	Number	2019	858.4214 52	1020.303 1	704.0951 68
Deaths	China	Female	85-89	Larynx cancer	Number	2019	227.2709 89	332.3156 45	124.3771 18
Deaths	China	Both	85-89	Larynx cancer	Number	2019	1085.692 44	1309.394 77	893.0819 62
Deaths	China	Male	85-89	Larynx cancer	Rate	2019	27.69411 89	32.91669 29	22.71529 36
Deaths	China	Female	85-89	Larynx cancer	Rate	2019	4.195169 25	6.134176 56	2.295863 02
Deaths	China	Both	85-89	Larynx cancer	Rate	2019	12.74721 15	15.37372 04	10.48575 47

Deaths	China	Male	90-94	Larynx cancer	Number	2019	209.084522	255.577126	168.026245
Deaths	China	Female	90-94	Larynx cancer	Number	2019	90.0852274	130.683845	49.0237889
Deaths	China	Both	90-94	Larynx cancer	Number	2019	299.169749	368.717728	237.255203
Deaths	China	Male	90-94	Larynx cancer	Rate	2019	29.5259894	36.0914689	23.7279216
Deaths	China	Female	90-94	Larynx cancer	Rate	2019	4.85454995	7.04234504	2.64181419
Deaths	China	Both	90-94	Larynx cancer	Rate	2019	11.6688892	14.3815554	9.25395931
Deaths	China	Male	95+ years	Larynx cancer	Number	2019	22.2355522	26.2942729	17.862119
Deaths	China	Female	95+ years	Larynx cancer	Number	2019	19.350927	28.1348209	9.8281533
Deaths	China	Both	95+ years	Larynx cancer	Number	2019	41.5864792	51.9317105	30.7310937
Deaths	China	Male	95+ years	Larynx cancer	Rate	2019	21.2303957	25.1056422	17.0546633
Deaths	China	Female	95+ years	Larynx cancer	Rate	2019	4.4429949	6.45978694	2.2565552
Deaths	China	Both	95+ years	Larynx cancer	Rate	2019	7.69731665	9.61213423	5.68807371
Deaths	China	Male	20-24 years	Larynx cancer	Number	2021	8.9933962	11.6583202	6.78781759
Deaths	China	Female	20-24 years	Larynx cancer	Number	2021	4.0986577	6.29991009	2.322357
Deaths	China	Both	20-24 years	Larynx cancer	Number	2021	13.0920539	16.6498012	10.2527589
Deaths	China	Male	20-24 years	Larynx cancer	Rate	2021	0.0231386	0.02999503	0.01746399
Deaths	China	Female	20-24 years	Larynx cancer	Rate	2021	0.01194712	0.01836352	0.00676941
Deaths	China	Both	20-24 years	Larynx cancer	Rate	2021	0.01789164	0.02275366	0.01401145
Deaths	China	Male	25-29 years	Larynx cancer	Number	2021	18.0386822	22.5900981	13.9375456
Deaths	China	Female	25-29 years	Larynx cancer	Number	2021	6.34430474	9.67127238	3.70415062
Deaths	China	Both	25-29 years	Larynx cancer	Number	2021	24.382987	29.4701249	19.661996
Deaths	China	Male	25-29 years	Larynx cancer	Rate	2021	0.03954487	0.0495226	0.03055425

Deaths	China	Female	25-29 years	Larynx cancer	Rate	2021	0.01552453	0.02366562	0.00906406
Deaths	China	Both	25-29 years	Larynx cancer	Rate	2021	0.02819427	0.03407657	0.02273534
Deaths	China	Male	30-34 years	Larynx cancer	Number	2021	43.0113199	53.790196	33.2083849
Deaths	China	Female	30-34 years	Larynx cancer	Number	2021	12.4815902	19.0849878	7.37065598
Deaths	China	Both	30-34 years	Larynx cancer	Number	2021	55.4929102	68.2805295	44.7243126
Deaths	China	Male	30-34 years	Larynx cancer	Rate	2021	0.06861633	0.08581197	0.05297762
Deaths	China	Female	30-34 years	Larynx cancer	Rate	2021	0.02134719	0.03264094	0.01260599
Deaths	China	Both	30-34 years	Larynx cancer	Rate	2021	0.04580389	0.0563588	0.03691548
Deaths	China	Male	35-39 years	Larynx cancer	Number	2021	95.9628707	122.659262	71.7129047
Deaths	China	Female	35-39 years	Larynx cancer	Number	2021	21.0559068	31.9174987	12.6816733
Deaths	China	Both	35-39 years	Larynx cancer	Number	2021	117.018778	144.813479	91.7096584
Deaths	China	Male	35-39 years	Larynx cancer	Rate	2021	0.17651231	0.22561716	0.13190738
Deaths	China	Female	35-39 years	Larynx cancer	Rate	2021	0.04080828	0.06185903	0.02457824
Deaths	China	Both	35-39 years	Larynx cancer	Rate	2021	0.11043335	0.13666386	0.08654855
Deaths	China	Male	40-44 years	Larynx cancer	Number	2021	208.880849	276.188029	149.411909
Deaths	China	Female	40-44 years	Larynx cancer	Number	2021	34.7439209	52.7128539	21.7292411
Deaths	China	Both	40-44 years	Larynx cancer	Number	2021	243.62477	312.821848	185.696151
Deaths	China	Male	40-44 years	Larynx cancer	Rate	2021	0.44513475	0.58856947	0.31840369
Deaths	China	Female	40-44 years	Larynx cancer	Rate	2021	0.07788654	0.11816806	0.04871112
Deaths	China	Both	40-44 years	Larynx cancer	Rate	2021	0.2661586	0.34175599	0.20287193
Deaths	China	Male	45-49 years	Larynx cancer	Number	2021	538.142818	733.17826	385.19694
Deaths	China	Female	45-49 years	Larynx cancer	Number	2021	76.2672482	113.102813	47.1324177

Deaths	China	Both	45-49 years	Larynx cancer	Number	2021	614.410066	809.100178	452.587612
Deaths	China	Male	45-49 years	Larynx cancer	Rate	2021	0.95991544	1.30781107	0.68709733
Deaths	China	Female	45-49 years	Larynx cancer	Rate	2021	0.14055867	0.20844572	0.08686389
Deaths	China	Both	45-49 years	Larynx cancer	Rate	2021	0.55692655	0.73340168	0.41024402
Deaths	China	Male	50-54 years	Larynx cancer	Number	2021	1259.35768	1737.2245	877.048774
Deaths	China	Female	50-54 years	Larynx cancer	Number	2021	152.799401	224.519706	94.3338164
Deaths	China	Both	50-54 years	Larynx cancer	Number	2021	1412.15709	1899.92059	1018.93776
Deaths	China	Male	50-54 years	Larynx cancer	Rate	2021	2.05975848	2.84133963	1.43446828
Deaths	China	Female	50-54 years	Larynx cancer	Rate	2021	0.25586826	0.37596657	0.15796547
Deaths	China	Both	50-54 years	Larynx cancer	Rate	2021	1.16843321	1.57201372	0.84307952
Deaths	China	Male	55-59 years	Larynx cancer	Number	2021	1845.99337	2520.40188	1303.97131
Deaths	China	Female	55-59 years	Larynx cancer	Number	2021	267.726817	390.188371	165.985641
Deaths	China	Both	55-59 years	Larynx cancer	Number	2021	2113.72018	2796.38493	1564.78394
Deaths	China	Male	55-59 years	Larynx cancer	Rate	2021	3.36347229	4.59227105	2.37588686
Deaths	China	Female	55-59 years	Larynx cancer	Rate	2021	0.48625606	0.70867559	0.3014697
Deaths	China	Both	55-59 years	Larynx cancer	Rate	2021	1.92257097	2.54350058	1.42327646
Deaths	China	Male	60-64 years	Larynx cancer	Number	2021	2000.78253	2757.97924	1406.14143
Deaths	China	Female	60-64 years	Larynx cancer	Number	2021	315.156667	446.267713	186.690644
Deaths	China	Both	60-64 years	Larynx cancer	Number	2021	2315.93919	3119.77138	1703.2523
Deaths	China	Male	60-64 years	Larynx cancer	Rate	2021	5.46283561	7.53024729	3.83925756
Deaths	China	Female	60-64 years	Larynx cancer	Rate	2021	0.86629532	1.22669031	0.5131709
Deaths	China	Both	60-64 years	Larynx cancer	Rate	2021	3.17229494	4.273357	2.33305721

Deaths	China	Male	65-69 years	Larynx cancer	Number	2021	2860.7762	3869.28324	2011.59456
Deaths	China	Female	65-69 years	Larynx cancer	Number	2021	541.294906	759.387403	319.936573
Deaths	China	Both	65-69 years	Larynx cancer	Number	2021	3402.07111	4492.26632	2529.52173
Deaths	China	Male	65-69 years	Larynx cancer	Rate	2021	7.58020604	10.2524497	5.33012727
Deaths	China	Female	65-69 years	Larynx cancer	Rate	2021	1.38923844	1.94897488	0.82112021
Deaths	China	Both	65-69 years	Larynx cancer	Rate	2021	4.43535246	5.85666315	3.29779128
Deaths	China	Male	70-74 years	Larynx cancer	Number	2021	2816.59309	3661.50087	2063.73415
Deaths	China	Female	70-74 years	Larynx cancer	Number	2021	600.012659	854.11166	350.933265
Deaths	China	Both	70-74 years	Larynx cancer	Number	2021	3416.60575	4373.08316	2633.72369
Deaths	China	Male	70-74 years	Larynx cancer	Rate	2021	10.8931755	14.1608569	7.98149312
Deaths	China	Female	70-74 years	Larynx cancer	Rate	2021	2.18663904	3.1126575	1.27891365
Deaths	China	Both	70-74 years	Larynx cancer	Rate	2021	6.41057002	8.20520655	4.9416501
Deaths	China	Male	75-79 years	Larynx cancer	Number	2021	2083.70339	2656.00349	1579.78265
Deaths	China	Female	75-79 years	Larynx cancer	Number	2021	510.438046	723.545791	286.447733
Deaths	China	Both	75-79 years	Larynx cancer	Number	2021	2594.14144	3262.99016	2058.74911
Deaths	China	Male	75-79 years	Larynx cancer	Rate	2021	13.3549919	17.0230106	10.125234
Deaths	China	Female	75-79 years	Larynx cancer	Rate	2021	2.91404033	4.13065137	1.63530178
Deaths	China	Both	75-79 years	Larynx cancer	Rate	2021	7.83280407	9.85233968	6.21622946
Deaths	China	Male	80-84	Larynx cancer	Number	2021	1507.01811	1867.80977	1164.81506
Deaths	China	Female	80-84	Larynx cancer	Number	2021	427.154471	616.823349	234.001738
Deaths	China	Both	80-84	Larynx cancer	Number	2021	1934.17258	2356.33151	1564.17937
Deaths	China	Male	80-84	Larynx cancer	Rate	2021	17.3720164	21.5310099	13.4273014

Deaths	China	Female	80-84	Larynx cancer	Rate	2021	3.842395	5.54852895	2.10492262
Deaths	China	Both	80-84	Larynx cancer	Rate	2021	9.772568	11.9055611	7.90314652
Deaths	China	Male	85-89	Larynx cancer	Number	2021	929.952153	1123.33173	747.969993
Deaths	China	Female	85-89	Larynx cancer	Number	2021	247.365589	364.798837	130.17631
Deaths	China	Both	85-89	Larynx cancer	Number	2021	1177.31774	1393.96236	948.903432
Deaths	China	Male	85-89	Larynx cancer	Rate	2021	26.715736	32.2711591	21.4877387
Deaths	China	Female	85-89	Larynx cancer	Rate	2021	4.09218053	6.03488424	2.15351279
Deaths	China	Both	85-89	Larynx cancer	Rate	2021	12.3593167	14.6336216	9.96145529
Deaths	China	Male	90-94	Larynx cancer	Number	2021	230.074279	283.937341	179.014546
Deaths	China	Female	90-94	Larynx cancer	Number	2021	100.875369	146.327667	54.0878799
Deaths	China	Both	90-94	Larynx cancer	Number	2021	330.949648	401.64164	259.142463
Deaths	China	Male	90-94	Larynx cancer	Rate	2021	28.4451551	35.1044965	22.1324025
Deaths	China	Female	90-94	Larynx cancer	Rate	2021	4.75120622	6.89199881	2.54752647
Deaths	China	Both	90-94	Larynx cancer	Rate	2021	11.2875532	13.6986137	8.8384573
Deaths	China	Male	95+ years	Larynx cancer	Number	2021	25.6379382	30.8625195	20.1486596
Deaths	China	Female	95+ years	Larynx cancer	Number	2021	23.3233006	35.6613833	11.6185021
Deaths	China	Both	95+ years	Larynx cancer	Number	2021	48.9612389	63.8394646	34.846193
Deaths	China	Male	95+ years	Larynx cancer	Rate	2021	21.1982445	25.5180908	16.6595383
Deaths	China	Female	95+ years	Larynx cancer	Rate	2021	4.50122284	6.88238064	2.24228414
Deaths	China	Both	95+ years	Larynx cancer	Rate	2021	7.66098538	9.98898754	5.45239829
Deaths	China	Male	20-24 years	Larynx cancer	Number	2020	9.303423	11.6986414	7.03164014
Deaths	China	Female	20-24 years	Larynx cancer	Number	2020	4.24260639	6.30885913	2.37849948

Deaths	China	Both	20-24 years	Larynx cancer	Number	2020	13.5460294	16.6206729	10.6546914
Deaths	China	Male	20-24 years	Larynx cancer	Rate	2020	0.02334361	0.02935355	0.01764338
Deaths	China	Female	20-24 years	Larynx cancer	Rate	2020	0.01196958	0.01779906	0.00671042
Deaths	China	Both	20-24 years	Larynx cancer	Rate	2020	0.01798962	0.02207285	0.01414982
Deaths	China	Male	25-29 years	Larynx cancer	Number	2020	19.2338885	23.6597303	15.2852242
Deaths	China	Female	25-29 years	Larynx cancer	Number	2020	6.86314152	10.1725293	4.2023951
Deaths	China	Both	25-29 years	Larynx cancer	Number	2020	26.09703	31.3915289	21.3762032
Deaths	China	Male	25-29 years	Larynx cancer	Rate	2020	0.0396682	0.04879611	0.03152443
Deaths	China	Female	25-29 years	Larynx cancer	Rate	2020	0.01556359	0.02306831	0.0095298
Deaths	China	Both	25-29 years	Larynx cancer	Rate	2020	0.02818731	0.03390588	0.02308836
Deaths	China	Male	30-34 years	Larynx cancer	Number	2020	44.0530929	54.6112542	34.818048
Deaths	China	Female	30-34 years	Larynx cancer	Number	2020	12.9989447	19.9627208	7.6090765
Deaths	China	Both	30-34 years	Larynx cancer	Number	2020	57.0520376	69.2651462	45.9702027
Deaths	China	Male	30-34 years	Larynx cancer	Rate	2020	0.068717	0.08518633	0.05431155
Deaths	China	Female	30-34 years	Larynx cancer	Rate	2020	0.02143024	0.03291082	0.01254443
Deaths	China	Both	30-34 years	Larynx cancer	Rate	2020	0.04572759	0.05551648	0.03684543
Deaths	China	Male	35-39 years	Larynx cancer	Number	2020	91.5827009	115.475734	69.8988431
Deaths	China	Female	35-39 years	Larynx cancer	Number	2020	20.0727405	30.516216	11.9794889
Deaths	China	Both	35-39 years	Larynx cancer	Number	2020	111.655441	137.134852	87.5527131
Deaths	China	Male	35-39 years	Larynx cancer	Rate	2020	0.1788076	0.22545675	0.13647167
Deaths	China	Female	35-39 years	Larynx cancer	Rate	2020	0.04137082	0.06289529	0.02469026
Deaths	China	Both	35-39 years	Larynx cancer	Rate	2020	0.11194913	0.13749556	0.08778301

Deaths	China	Male	40-44 years	Larynx cancer	Number	2020	214.738847	282.584499	159.029282
Deaths	China	Female	40-44 years	Larynx cancer	Number	2020	36.0182342	53.5809116	21.7542143
Deaths	China	Both	40-44 years	Larynx cancer	Number	2020	250.757081	317.550306	192.432259
Deaths	China	Male	40-44 years	Larynx cancer	Rate	2020	0.4482141	0.58982507	0.33193419
Deaths	China	Female	40-44 years	Larynx cancer	Rate	2020	0.07877139	0.11718073	0.04757617
Deaths	China	Both	40-44 years	Larynx cancer	Rate	2020	0.267803	0.33913669	0.20551339
Deaths	China	Male	45-49 years	Larynx cancer	Number	2020	564.845721	745.015374	400.560518
Deaths	China	Female	45-49 years	Larynx cancer	Number	2020	79.4176662	115.679112	49.3520278
Deaths	China	Both	45-49 years	Larynx cancer	Number	2020	644.263387	825.399897	475.021714
Deaths	China	Male	45-49 years	Larynx cancer	Rate	2020	0.96626994	1.27448246	0.68523063
Deaths	China	Female	45-49 years	Larynx cancer	Rate	2020	0.14079641	0.20508288	0.08749424
Deaths	China	Both	45-49 years	Larynx cancer	Rate	2020	0.56090046	0.71859925	0.41355741
Deaths	China	Male	50-54 years	Larynx cancer	Number	2020	1283.44311	1722.10456	896.630738
Deaths	China	Female	50-54 years	Larynx cancer	Number	2020	156.232259	227.732253	97.0295915
Deaths	China	Both	50-54 years	Larynx cancer	Number	2020	1439.67537	1876.80838	1041.1405
Deaths	China	Male	50-54 years	Larynx cancer	Rate	2020	2.09065111	2.80520406	1.46055718
Deaths	China	Female	50-54 years	Larynx cancer	Rate	2020	0.25849848	0.37680081	0.16054304
Deaths	China	Both	50-54 years	Larynx cancer	Rate	2020	1.18172779	1.5405394	0.85459867
Deaths	China	Male	55-59 years	Larynx cancer	Number	2020	1736.73176	2307.74526	1220.13258
Deaths	China	Female	55-59 years	Larynx cancer	Number	2020	248.212985	365.236047	150.944462
Deaths	China	Both	55-59 years	Larynx cancer	Number	2020	1984.94474	2552.97178	1461.59583
Deaths	China	Male	55-59 years	Larynx cancer	Rate	2020	3.39677051	4.51358191	2.38638486

Deaths	China	Female	55-59 years	Larynx cancer	Rate	2020	0.48713881	0.71680639	0.29624117
Deaths	China	Both	55-59 years	Larynx cancer	Rate	2020	1.94445829	2.50089941	1.431784
Deaths	China	Male	60-64 years	Larynx cancer	Number	2020	2080.17167	2778.43693	1457.99065
Deaths	China	Female	60-64 years	Larynx cancer	Number	2020	326.35011	472.874368	190.933978
Deaths	China	Both	60-64 years	Larynx cancer	Number	2020	2406.52178	3151.36302	1755.16287
Deaths	China	Male	60-64 years	Larynx cancer	Rate	2020	5.58875043	7.46476397	3.91715068
Deaths	China	Female	60-64 years	Larynx cancer	Rate	2020	0.88520516	1.28264345	0.517897
Deaths	China	Both	60-64 years	Larynx cancer	Rate	2020	3.24819963	4.25354811	2.36902879
Deaths	China	Male	65-69 years	Larynx cancer	Number	2020	2796.64014	3669.96032	1988.49545
Deaths	China	Female	65-69 years	Larynx cancer	Number	2020	529.35338	754.236594	314.383203
Deaths	China	Both	65-69 years	Larynx cancer	Number	2020	3325.99352	4282.37239	2482.98929
Deaths	China	Male	65-69 years	Larynx cancer	Rate	2020	7.64810983	10.0364216	5.43803665
Deaths	China	Female	65-69 years	Larynx cancer	Rate	2020	1.39639216	1.9896162	0.82931791
Deaths	China	Both	65-69 years	Larynx cancer	Rate	2020	4.46591528	5.75007502	3.33398719
Deaths	China	Male	70-74 years	Larynx cancer	Number	2020	2684.2771	3408.53401	1972.12828
Deaths	China	Female	70-74 years	Larynx cancer	Number	2020	566.608589	800.069191	339.803482
Deaths	China	Both	70-74 years	Larynx cancer	Number	2020	3250.88569	4060.38468	2494.33039
Deaths	China	Male	70-74 years	Larynx cancer	Rate	2020	11.0241474	13.9986224	8.0993996
Deaths	China	Female	70-74 years	Larynx cancer	Rate	2020	2.21094863	3.12192917	1.32593833
Deaths	China	Both	70-74 years	Larynx cancer	Rate	2020	6.50483323	8.12459363	4.99101006
Deaths	China	Male	75-79 years	Larynx cancer	Number	2020	2018.83653	2524.4175	1510.62847
Deaths	China	Female	75-79 years	Larynx cancer	Number	2020	494.005033	706.791764	278.881498

Deaths	China	Both	75-79 years	Larynx cancer	Number	2020	2512.84156	3065.85349	1965.60641
Deaths	China	Male	75-79 years	Larynx cancer	Rate	2020	13.6141131	17.0235207	10.1869897
Deaths	China	Female	75-79 years	Larynx cancer	Rate	2020	2.98251639	4.26719949	1.68372503
Deaths	China	Both	75-79 years	Larynx cancer	Rate	2020	8.00462743	9.76624045	6.26141624
Deaths	China	Male	80-84	Larynx cancer	Number	2020	1491.73006	1825.03626	1168.40843
Deaths	China	Female	80-84	Larynx cancer	Number	2020	422.482611	605.527118	222.561033
Deaths	China	Both	80-84	Larynx cancer	Number	2020	1914.21268	2279.41713	1523.66078
Deaths	China	Male	80-84	Larynx cancer	Rate	2020	17.6412405	21.5829287	13.81763
Deaths	China	Female	80-84	Larynx cancer	Rate	2020	3.88899046	5.57393162	2.04869434
Deaths	China	Both	80-84	Larynx cancer	Rate	2020	9.90819986	11.798543	7.88665529
Deaths	China	Male	85-89	Larynx cancer	Number	2020	888.629948	1064.3382	719.296331
Deaths	China	Female	85-89	Larynx cancer	Number	2020	237.080714	352.73239	128.157331
Deaths	China	Both	85-89	Larynx cancer	Number	2020	1125.71066	1323.09205	919.021137
Deaths	China	Male	85-89	Larynx cancer	Rate	2020	26.9851609	32.3209202	21.8429812
Deaths	China	Female	85-89	Larynx cancer	Rate	2020	4.13018738	6.1449573	2.23263116
Deaths	China	Both	85-89	Larynx cancer	Rate	2020	12.4618917	14.6469519	10.1737882
Deaths	China	Male	90-94	Larynx cancer	Number	2020	218.279642	264.543067	169.730882
Deaths	China	Female	90-94	Larynx cancer	Number	2020	95.398095	137.765389	50.4025483
Deaths	China	Both	90-94	Larynx cancer	Number	2020	313.677737	374.658009	245.120059
Deaths	China	Male	90-94	Larynx cancer	Rate	2020	28.8120114	34.9185925	22.4037756
Deaths	China	Female	90-94	Larynx cancer	Rate	2020	4.80062792	6.9326371	2.53635967
Deaths	China	Both	90-94	Larynx cancer	Rate	2020	11.4280734	13.649739	8.93034376

Deaths	China	Male	95+ years	Larynx cancer	Number	2020	24.1277913	28.5197641	19.1202197
Deaths	China	Female	95+ years	Larynx cancer	Number	2020	21.7070452	32.9235311	10.6695411
Deaths	China	Both	95+ years	Larynx cancer	Number	2020	45.8348365	59.1581596	32.9126341
Deaths	China	Male	95+ years	Larynx cancer	Rate	2020	21.402465	25.2983477	16.9605178
Deaths	China	Female	95+ years	Larynx cancer	Rate	2020	4.55752335	6.91249135	2.24013367
Deaths	China	Both	95+ years	Larynx cancer	Rate	2020	7.78148773	10.0434195	5.58765511
Incidence	China	Male	5-9 years	Larynx cancer	Number	1990	0	0	0
Incidence	China	Female	5-9 years	Larynx cancer	Number	1990	0	0	0
Incidence	China	Both	5-9 years	Larynx cancer	Number	1990	0	0	0
Incidence	China	Male	5-9 years	Larynx cancer	Rate	1990	0	0	0
Incidence	China	Female	5-9 years	Larynx cancer	Rate	1990	0	0	0
Incidence	China	Both	5-9 years	Larynx cancer	Rate	1990	0	0	0
Incidence	China	Male	5-9 years	Larynx cancer	Number	2000	0	0	0
Incidence	China	Female	5-9 years	Larynx cancer	Number	2000	0	0	0
Incidence	China	Both	5-9 years	Larynx cancer	Number	2000	0	0	0
Incidence	China	Male	5-9 years	Larynx cancer	Rate	2000	0	0	0
Incidence	China	Female	5-9 years	Larynx cancer	Rate	2000	0	0	0
Incidence	China	Both	5-9 years	Larynx cancer	Rate	2000	0	0	0
Incidence	China	Male	5-9 years	Larynx cancer	Number	2010	0	0	0
Incidence	China	Female	5-9 years	Larynx cancer	Number	2010	0	0	0
Incidence	China	Both	5-9 years	Larynx cancer	Number	2010	0	0	0
Incidence	China	Male	5-9 years	Larynx cancer	Rate	2010	0	0	0

Incidence	China	Female	5-9 years	Larynx cancer	Rate	2010	0	0	0
Incidence	China	Both	5-9 years	Larynx cancer	Rate	2010	0	0	0
Incidence	China	Male	5-9 years	Larynx cancer	Number	2019	0	0	0
Incidence	China	Female	5-9 years	Larynx cancer	Number	2019	0	0	0
Incidence	China	Both	5-9 years	Larynx cancer	Number	2019	0	0	0
Incidence	China	Male	5-9 years	Larynx cancer	Rate	2019	0	0	0
Incidence	China	Female	5-9 years	Larynx cancer	Rate	2019	0	0	0
Incidence	China	Both	5-9 years	Larynx cancer	Rate	2019	0	0	0
Incidence	China	Male	5-9 years	Larynx cancer	Number	2020	0	0	0
Incidence	China	Female	5-9 years	Larynx cancer	Number	2020	0	0	0
Incidence	China	Both	5-9 years	Larynx cancer	Number	2020	0	0	0
Incidence	China	Male	5-9 years	Larynx cancer	Rate	2020	0	0	0
Incidence	China	Female	5-9 years	Larynx cancer	Rate	2020	0	0	0
Incidence	China	Both	5-9 years	Larynx cancer	Rate	2020	0	0	0
Incidence	China	Male	5-9 years	Larynx cancer	Number	2021	0	0	0
Incidence	China	Female	5-9 years	Larynx cancer	Number	2021	0	0	0
Incidence	China	Both	5-9 years	Larynx cancer	Number	2021	0	0	0
Incidence	China	Male	5-9 years	Larynx cancer	Rate	2021	0	0	0
Incidence	China	Female	5-9 years	Larynx cancer	Rate	2021	0	0	0
Incidence	China	Both	5-9 years	Larynx cancer	Rate	2021	0	0	0
Incidence	China	Male	10-14 years	Larynx cancer	Number	1990	0	0	0
Incidence	China	Female	10-14 years	Larynx cancer	Number	1990	0	0	0

Incidence	China	Both	10-14 years	Larynx cancer	Number	1990	0	0	0
Incidence	China	Male	10-14 years	Larynx cancer	Rate	1990	0	0	0
Incidence	China	Female	10-14 years	Larynx cancer	Rate	1990	0	0	0
Incidence	China	Both	10-14 years	Larynx cancer	Rate	1990	0	0	0
Incidence	China	Male	10-14 years	Larynx cancer	Number	2000	0	0	0
Incidence	China	Female	10-14 years	Larynx cancer	Number	2000	0	0	0
Incidence	China	Both	10-14 years	Larynx cancer	Number	2000	0	0	0
Incidence	China	Male	10-14 years	Larynx cancer	Rate	2000	0	0	0
Incidence	China	Female	10-14 years	Larynx cancer	Rate	2000	0	0	0
Incidence	China	Both	10-14 years	Larynx cancer	Rate	2000	0	0	0
Incidence	China	Male	10-14 years	Larynx cancer	Number	2010	0	0	0
Incidence	China	Female	10-14 years	Larynx cancer	Number	2010	0	0	0
Incidence	China	Both	10-14 years	Larynx cancer	Number	2010	0	0	0
Incidence	China	Male	10-14 years	Larynx cancer	Rate	2010	0	0	0
Incidence	China	Female	10-14 years	Larynx cancer	Rate	2010	0	0	0
Incidence	China	Both	10-14 years	Larynx cancer	Rate	2010	0	0	0
Incidence	China	Male	10-14 years	Larynx cancer	Number	2019	0	0	0
Incidence	China	Female	10-14 years	Larynx cancer	Number	2019	0	0	0
Incidence	China	Both	10-14 years	Larynx cancer	Number	2019	0	0	0
Incidence	China	Male	10-14 years	Larynx cancer	Rate	2019	0	0	0
Incidence	China	Female	10-14 years	Larynx cancer	Rate	2019	0	0	0
Incidence	China	Both	10-14 years	Larynx cancer	Rate	2019	0	0	0

Incidence	China	Male	10-14 years	Larynx cancer	Number	2020	0	0	0
Incidence	China	Female	10-14 years	Larynx cancer	Number	2020	0	0	0
Incidence	China	Both	10-14 years	Larynx cancer	Number	2020	0	0	0
Incidence	China	Male	10-14 years	Larynx cancer	Rate	2020	0	0	0
Incidence	China	Female	10-14 years	Larynx cancer	Rate	2020	0	0	0
Incidence	China	Both	10-14 years	Larynx cancer	Rate	2020	0	0	0
Incidence	China	Male	10-14 years	Larynx cancer	Number	2021	0	0	0
Incidence	China	Female	10-14 years	Larynx cancer	Number	2021	0	0	0
Incidence	China	Both	10-14 years	Larynx cancer	Number	2021	0	0	0
Incidence	China	Male	10-14 years	Larynx cancer	Rate	2021	0	0	0
Incidence	China	Female	10-14 years	Larynx cancer	Rate	2021	0	0	0
Incidence	China	Both	10-14 years	Larynx cancer	Rate	2021	0	0	0
Incidence	China	Male	15-19 years	Larynx cancer	Number	1990	0	0	0
Incidence	China	Female	15-19 years	Larynx cancer	Number	1990	0	0	0
Incidence	China	Both	15-19 years	Larynx cancer	Number	1990	0	0	0
Incidence	China	Male	15-19 years	Larynx cancer	Rate	1990	0	0	0
Incidence	China	Female	15-19 years	Larynx cancer	Rate	1990	0	0	0
Incidence	China	Both	15-19 years	Larynx cancer	Rate	1990	0	0	0
Incidence	China	Male	15-19 years	Larynx cancer	Number	2000	0	0	0
Incidence	China	Female	15-19 years	Larynx cancer	Number	2000	0	0	0
Incidence	China	Both	15-19 years	Larynx cancer	Number	2000	0	0	0
Incidence	China	Male	15-19 years	Larynx cancer	Rate	2000	0	0	0

**Incidence**	**China**	**Female**	**15-19 years**	**Larynx cancer**	**Rate**	**2000**	**0**	**0**	**0**
**Incidence**	**China**	**Both**	**15-19 years**	**Larynx cancer**	**Rate**	**2000**	**0**	**0**	**0**
**Incidence**	**China**	**Male**	**15-19 years**	**Larynx cancer**	**Number**	**2010**	**0**	**0**	**0**
**Incidence**	**China**	**Female**	**15-19 years**	**Larynx cancer**	**Number**	**2010**	**0**	**0**	**0**
**Incidence**	**China**	**Both**	**15-19 years**	**Larynx cancer**	**Number**	**2010**	**0**	**0**	**0**
**Incidence**	**China**	**Male**	**15-19 years**	**Larynx cancer**	**Rate**	**2010**	**0**	**0**	**0**
**Incidence**	**China**	**Female**	**15-19 years**	**Larynx cancer**	**Rate**	**2010**	**0**	**0**	**0**
**Incidence**	**China**	**Both**	**15-19 years**	**Larynx cancer**	**Rate**	**2010**	**0**	**0**	**0**
**Incidence**	**China**	**Male**	**15-19 years**	**Larynx cancer**	**Number**	**2019**	**0**	**0**	**0**
**Incidence**	**China**	**Female**	**15-19 years**	**Larynx cancer**	**Number**	**2019**	**0**	**0**	**0**
**Incidence**	**China**	**Both**	**15-19 years**	**Larynx cancer**	**Number**	**2019**	**0**	**0**	**0**
**Incidence**	**China**	**Male**	**15-19 years**	**Larynx cancer**	**Rate**	**2019**	**0**	**0**	**0**
**Incidence**	**China**	**Female**	**15-19 years**	**Larynx cancer**	**Rate**	**2019**	**0**	**0**	**0**
**Incidence**	**China**	**Both**	**15-19 years**	**Larynx cancer**	**Rate**	**2019**	**0**	**0**	**0**
**Incidence**	**China**	**Male**	**15-19 years**	**Larynx cancer**	**Number**	**2020**	**0**	**0**	**0**
**Incidence**	**China**	**Female**	**15-19 years**	**Larynx cancer**	**Number**	**2020**	**0**	**0**	**0**
**Incidence**	**China**	**Both**	**15-19 years**	**Larynx cancer**	**Number**	**2020**	**0**	**0**	**0**
**Incidence**	**China**	**Male**	**15-19 years**	**Larynx cancer**	**Rate**	**2020**	**0**	**0**	**0**
**Incidence**	**China**	**Female**	**15-19 years**	**Larynx cancer**	**Rate**	**2020**	**0**	**0**	**0**
**Incidence**	**China**	**Both**	**15-19 years**	**Larynx cancer**	**Rate**	**2020**	**0**	**0**	**0**
**Incidence**	**China**	**Male**	**15-19 years**	**Larynx cancer**	**Number**	**2021**	**0**	**0**	**0**
**Incidence**	**China**	**Female**	**15-19 years**	**Larynx cancer**	**Number**	**2021**	**0**	**0**	**0**

Incidence	China	Both	15-19 years	Larynx cancer	Number	2021	0	0	0
Incidence	China	Male	15-19 years	Larynx cancer	Rate	2021	0	0	0
Incidence	China	Female	15-19 years	Larynx cancer	Rate	2021	0	0	0
Incidence	China	Both	15-19 years	Larynx cancer	Rate	2021	0	0	0
Incidence	China	Male	20-24 years	Larynx cancer	Number	1990	34.4586943	41.5873049	27.1444639
Incidence	China	Female	20-24 years	Larynx cancer	Number	1990	18.1972809	25.3566552	5.99469752
Incidence	China	Both	20-24 years	Larynx cancer	Number	1990	52.6559751	62.31343	37.4845118
Incidence	China	Male	20-24 years	Larynx cancer	Rate	1990	0.05103935	0.06159807	0.04020569
Incidence	China	Female	20-24 years	Larynx cancer	Rate	1990	0.02821856	0.03932061	0.00929599
Incidence	China	Both	20-24 years	Larynx cancer	Rate	1990	0.03989062	0.04720682	0.02839716
Incidence	China	Male	20-24 years	Larynx cancer	Number	2000	21.6039797	24.770408	17.6234799
Incidence	China	Female	20-24 years	Larynx cancer	Number	2000	11.9548041	14.8149998	5.5868211
Incidence	China	Both	20-24 years	Larynx cancer	Number	2000	33.5587838	37.9701013	26.4829106
Incidence	China	Male	20-24 years	Larynx cancer	Rate	2000	0.04475808	0.05131813	0.03651147
Incidence	China	Female	20-24 years	Larynx cancer	Rate	2000	0.02549728	0.03159753	0.01191561
Incidence	China	Both	20-24 years	Larynx cancer	Rate	2000	0.03526753	0.03990346	0.02783136
Incidence	China	Male	20-24 years	Larynx cancer	Number	2010	26.6572427	31.0937665	21.7975821
Incidence	China	Female	20-24 years	Larynx cancer	Number	2010	17.7144321	22.0680617	12.097085
Incidence	China	Both	20-24 years	Larynx cancer	Number	2010	44.3716748	50.2582237	37.6561787
Incidence	China	Male	20-24 years	Larynx cancer	Rate	2010	0.04150623	0.04841405	0.03393957
Incidence	China	Female	20-24 years	Larynx cancer	Rate	2010	0.02777802	0.03460495	0.01896945
Incidence	China	Both	20-24 years	Larynx cancer	Rate	2010	0.03466643	0.03926544	0.02941979

Incidence	China	Male	20-24 years	Larynx cancer	Number	2019	22.1606118	29.0864849	16.8226617
Incidence	China	Female	20-24 years	Larynx cancer	Number	2019	11.4181124	18.2512107	6.59230643
Incidence	China	Both	20-24 years	Larynx cancer	Number	2019	33.5787242	43.3128824	26.2592546
Incidence	China	Male	20-24 years	Larynx cancer	Rate	2019	0.05376551	0.07056888	0.04081471
Incidence	China	Female	20-24 years	Larynx cancer	Rate	2019	0.03091095	0.04940942	0.0178466
Incidence	China	Both	20-24 years	Larynx cancer	Rate	2019	0.04296378	0.05541858	0.03359857
Incidence	China	Male	20-24 years	Larynx cancer	Number	2020	21.1609635	26.8664419	16.0745925
Incidence	China	Female	20-24 years	Larynx cancer	Number	2020	10.7544266	16.2573111	6.01483321
Incidence	China	Both	20-24 years	Larynx cancer	Number	2020	31.9153901	39.1437091	25.4870767
Incidence	China	Male	20-24 years	Larynx cancer	Rate	2020	0.05309586	0.06741172	0.04033343
Incidence	China	Female	20-24 years	Larynx cancer	Rate	2020	0.03034126	0.04586644	0.01696953
Incidence	China	Both	20-24 years	Larynx cancer	Rate	2020	0.04238479	0.05198426	0.03384776
Incidence	China	Male	20-24 years	Larynx cancer	Number	2021	20.7183677	26.5011004	15.6767096
Incidence	China	Female	20-24 years	Larynx cancer	Number	2021	10.5407223	16.4725747	6.08153415
Incidence	China	Both	20-24 years	Larynx cancer	Number	2021	31.25909	38.7229773	24.5857371
Incidence	China	Male	20-24 years	Larynx cancer	Rate	2021	0.05330512	0.06818318	0.04033372
Incidence	China	Female	20-24 years	Larynx cancer	Rate	2021	0.03072501	0.04801568	0.01772698
Incidence	China	Both	20-24 years	Larynx cancer	Rate	2021	0.04271876	0.05291892	0.03359893
Incidence	China	Male	25-29 years	Larynx cancer	Number	1990	49.4895094	62.2365993	39.1300235
Incidence	China	Female	25-29 years	Larynx cancer	Number	1990	24.0270441	34.0920855	8.32694101
Incidence	China	Both	25-29 years	Larynx cancer	Number	1990	73.5165536	87.1210727	52.9262214
Incidence	China	Male	25-29 years	Larynx cancer	Rate	1990	0.08770614	0.11029675	0.06934689

Incidence	China	Female	25-29 years	Larynx cancer	Rate	1990	0.04494148	0.06376768	0.01557516
Incidence	China	Both	25-29 years	Larynx cancer	Rate	1990	0.06690046	0.07928065	0.04816315
Incidence	China	Male	25-29 years	Larynx cancer	Number	2000	46.55109	53.9510737	39.6161146
Incidence	China	Female	25-29 years	Larynx cancer	Number	2000	21.9164978	27.6871147	10.5279415
Incidence	China	Both	25-29 years	Larynx cancer	Number	2000	68.4675878	78.243833	55.3517881
Incidence	China	Male	25-29 years	Larynx cancer	Rate	2000	0.07685498	0.08907221	0.06540546
Incidence	China	Female	25-29 years	Larynx cancer	Rate	2000	0.03806111	0.0480826	0.01828326
Incidence	China	Both	25-29 years	Larynx cancer	Rate	2000	0.05794852	0.06622278	0.04684778
Incidence	China	Male	25-29 years	Larynx cancer	Number	2010	42.7753812	48.4623724	36.7709031
Incidence	China	Female	25-29 years	Larynx cancer	Number	2010	24.6694899	31.6655354	16.7511006
Incidence	China	Both	25-29 years	Larynx cancer	Number	2010	67.4448711	76.7168367	57.9887733
Incidence	China	Male	25-29 years	Larynx cancer	Rate	2010	0.08373694	0.09486977	0.07198259
Incidence	China	Female	25-29 years	Larynx cancer	Rate	2010	0.04877842	0.06261154	0.03312157
Incidence	China	Both	25-29 years	Larynx cancer	Rate	2010	0.0663451	0.07546588	0.0570432
Incidence	China	Male	25-29 years	Larynx cancer	Number	2019	51.2437243	62.4423899	40.4728017
Incidence	China	Female	25-29 years	Larynx cancer	Number	2019	20.710653	29.7115826	12.9349348
Incidence	China	Both	25-29 years	Larynx cancer	Number	2019	71.9543773	85.8838114	59.4414363
Incidence	China	Male	25-29 years	Larynx cancer	Rate	2019	0.09833339	0.11982291	0.07766469
Incidence	China	Female	25-29 years	Larynx cancer	Rate	2019	0.04295773	0.06162733	0.02682945
Incidence	China	Both	25-29 years	Larynx cancer	Rate	2019	0.07172205	0.08560651	0.05924951
Incidence	China	Male	25-29 years	Larynx cancer	Number	2020	48.4849067	60.6972245	38.1373247
Incidence	China	Female	25-29 years	Larynx cancer	Number	2020	19.3718836	28.6355351	11.638361

Incidence	China	Both	25-29 years	Larynx cancer	Number	2020	67.8567902	81.4598955	55.1509772
Incidence	China	Male	25-29 years	Larynx cancer	Rate	2020	0.09999584	0.12518267	0.07865487
Incidence	China	Female	25-29 years	Larynx cancer	Rate	2020	0.04392975	0.064937	0.02639239
Incidence	China	Both	25-29 years	Larynx cancer	Rate	2020	0.07329187	0.08798454	0.05956837
Incidence	China	Male	25-29 years	Larynx cancer	Number	2021	46.1748439	57.7764953	35.753059
Incidence	China	Female	25-29 years	Larynx cancer	Number	2021	18.1574953	28.2370723	10.7655752
Incidence	China	Both	25-29 years	Larynx cancer	Number	2021	64.3323392	77.4182872	52.4008716
Incidence	China	Male	25-29 years	Larynx cancer	Rate	2021	0.10122569	0.12665913	0.07837878
Incidence	China	Female	25-29 years	Larynx cancer	Rate	2021	0.04443143	0.06909617	0.02634338
Incidence	China	Both	25-29 years	Larynx cancer	Rate	2021	0.07438807	0.08951947	0.0605916
Incidence	China	Male	30-34 years	Larynx cancer	Number	1990	78.7824264	96.5051245	62.5100349
Incidence	China	Female	30-34 years	Larynx cancer	Number	1990	28.6936389	39.7687261	10.4275665
Incidence	China	Both	30-34 years	Larynx cancer	Number	1990	107.476065	129.314001	81.4559806
Incidence	China	Male	30-34 years	Larynx cancer	Rate	1990	0.17117397	0.20968084	0.13581825
Incidence	China	Female	30-34 years	Larynx cancer	Rate	1990	0.06796292	0.09419504	0.02469843
Incidence	China	Both	30-34 years	Larynx cancer	Rate	1990	0.12179375	0.14654087	0.09230733
Incidence	China	Male	30-34 years	Larynx cancer	Number	2000	94.871123	109.183498	79.7545748
Incidence	China	Female	30-34 years	Larynx cancer	Number	2000	34.4061268	44.1093891	16.7566861
Incidence	China	Both	30-34 years	Larynx cancer	Number	2000	129.27725	146.444323	108.084759
Incidence	China	Male	30-34 years	Larynx cancer	Rate	2000	0.14401133	0.16573706	0.12106489
Incidence	China	Female	30-34 years	Larynx cancer	Rate	2000	0.05513915	0.07068956	0.02685421
Incidence	China	Both	30-34 years	Larynx cancer	Rate	2000	0.10078033	0.11416322	0.08425936

Incidence	China	Male	30-34 years	Larynx cancer	Number	2010	77.2160597	87.8925976	64.8166604
Incidence	China	Female	30-34 years	Larynx cancer	Number	2010	31.7079989	40.8990403	21.1534933
Incidence	China	Both	30-34 years	Larynx cancer	Number	2010	108.924059	122.432014	95.50348
Incidence	China	Male	30-34 years	Larynx cancer	Rate	2010	0.15537404	0.17685735	0.13042399
Incidence	China	Female	30-34 years	Larynx cancer	Rate	2010	0.06613359	0.08530341	0.04411999
Incidence	China	Both	30-34 years	Larynx cancer	Rate	2010	0.11155422	0.12538834	0.09780958
Incidence	China	Male	30-34 years	Larynx cancer	Number	2019	110.324167	135.650219	88.0153495
Incidence	China	Female	30-34 years	Larynx cancer	Number	2019	37.0889534	53.5026922	22.3644498
Incidence	China	Both	30-34 years	Larynx cancer	Number	2019	147.41312	178.298456	119.98782
Incidence	China	Male	30-34 years	Larynx cancer	Rate	2019	0.17337451	0.21317441	0.13831618
Incidence	China	Female	30-34 years	Larynx cancer	Rate	2019	0.06083013	0.08775054	0.03668026
Incidence	China	Both	30-34 years	Larynx cancer	Rate	2019	0.11830453	0.14309117	0.09629471
Incidence	China	Male	30-34 years	Larynx cancer	Number	2020	112.621228	141.836298	88.232101
Incidence	China	Female	30-34 years	Larynx cancer	Number	2020	37.7154946	57.9663273	21.9084742
Incidence	China	Both	30-34 years	Larynx cancer	Number	2020	150.336723	184.268905	120.067444
Incidence	China	Male	30-34 years	Larynx cancer	Rate	2020	0.17567422	0.22124587	0.13763041
Incidence	China	Female	30-34 years	Larynx cancer	Rate	2020	0.06217829	0.0955641	0.03611862
Incidence	China	Both	30-34 years	Larynx cancer	Rate	2020	0.1204959	0.14769277	0.09623487
Incidence	China	Male	30-34 years	Larynx cancer	Number	2021	111.866277	141.491753	87.8151703
Incidence	China	Female	30-34 years	Larynx cancer	Number	2021	36.7910579	57.1731425	22.0302885
Incidence	China	Both	30-34 years	Larynx cancer	Number	2021	148.657335	180.848103	121.388888
Incidence	China	Male	30-34 years	Larynx cancer	Rate	2021	0.17846124	0.22572302	0.1400923

Incidence	China	Female	30-34 years	Larynx cancer	Rate	2021	0.06292352	0.09778287	0.03767827
Incidence	China	Both	30-34 years	Larynx cancer	Rate	2021	0.12270187	0.14927215	0.10019447
Incidence	China	Male	35-39 years	Larynx cancer	Number	1990	232.083521	284.319355	185.313502
Incidence	China	Female	35-39 years	Larynx cancer	Number	1990	65.559476	90.4240046	28.803716
Incidence	China	Both	35-39 years	Larynx cancer	Number	1990	297.642997	351.891123	237.782866
Incidence	China	Male	35-39 years	Larynx cancer	Rate	1990	0.49143061	0.60203859	0.39239635
Incidence	China	Female	35-39 years	Larynx cancer	Rate	1990	0.14861729	0.20498288	0.06529537
Incidence	China	Both	35-39 years	Larynx cancer	Rate	1990	0.32586607	0.38525811	0.26032989
Incidence	China	Male	35-39 years	Larynx cancer	Number	2000	245.128186	287.156247	210.492523
Incidence	China	Female	35-39 years	Larynx cancer	Number	2000	64.9712791	82.0330757	37.5091252
Incidence	China	Both	35-39 years	Larynx cancer	Number	2000	310.099465	350.979269	267.383278
Incidence	China	Male	35-39 years	Larynx cancer	Rate	2000	0.43393445	0.50833399	0.37262119
Incidence	China	Female	35-39 years	Larynx cancer	Rate	2000	0.12168288	0.15363744	0.07024978
Incidence	China	Both	35-39 years	Larynx cancer	Rate	2000	0.28220719	0.31941001	0.24333316
Incidence	China	Male	35-39 years	Larynx cancer	Number	2010	249.087991	283.022021	213.038423
Incidence	China	Female	35-39 years	Larynx cancer	Number	2010	69.4956038	88.8739801	45.6998998
Incidence	China	Both	35-39 years	Larynx cancer	Number	2010	318.583595	357.761791	277.541081
Incidence	China	Male	35-39 years	Larynx cancer	Rate	2010	0.41088399	0.46685999	0.35141829
Incidence	China	Female	35-39 years	Larynx cancer	Rate	2010	0.11985778	0.15327931	0.07881777
Incidence	China	Both	35-39 years	Larynx cancer	Rate	2010	0.26861076	0.30164349	0.23400615
Incidence	China	Male	35-39 years	Larynx cancer	Number	2019	238.09081	300.571475	186.941659
Incidence	China	Female	35-39 years	Larynx cancer	Number	2019	59.4360862	85.8136475	36.7040572

Incidence	China	Both	35-39 years	Larynx cancer	Number	2019	297.526896	369.646047	238.56338
Incidence	China	Male	35-39 years	Larynx cancer	Rate	2019	0.48553948	0.61295654	0.38123083
Incidence	China	Female	35-39 years	Larynx cancer	Rate	2019	0.12808691	0.18493151	0.07909857
Incidence	China	Both	35-39 years	Larynx cancer	Rate	2019	0.3117447	0.38731019	0.24996352
Incidence	China	Male	35-39 years	Larynx cancer	Number	2020	246.46667	313.514426	191.917639
Incidence	China	Female	35-39 years	Larynx cancer	Number	2020	61.6628244	92.2448983	36.6380881
Incidence	China	Both	35-39 years	Larynx cancer	Number	2020	308.129495	380.130484	245.901563
Incidence	China	Male	35-39 years	Larynx cancer	Rate	2020	0.48120565	0.6121108	0.37470321
Incidence	China	Female	35-39 years	Larynx cancer	Rate	2020	0.12708984	0.19012086	0.07551274
Incidence	China	Both	35-39 years	Larynx cancer	Rate	2020	0.30893998	0.38113035	0.24654837
Incidence	China	Male	35-39 years	Larynx cancer	Number	2021	262.484781	331.419444	195.812101
Incidence	China	Female	35-39 years	Larynx cancer	Number	2021	65.7860203	97.8629633	41.0370319
Incidence	China	Both	35-39 years	Larynx cancer	Number	2021	328.270801	398.336601	258.391087
Incidence	China	Male	35-39 years	Larynx cancer	Rate	2021	0.48280961	0.60960674	0.36017312
Incidence	China	Female	35-39 years	Larynx cancer	Rate	2021	0.12749933	0.18966738	0.07953352
Incidence	China	Both	35-39 years	Larynx cancer	Rate	2021	0.30979682	0.37591956	0.24384971
Incidence	China	Male	40-44 years	Larynx cancer	Number	1990	458.113708	572.45502	346.999985
Incidence	China	Female	40-44 years	Larynx cancer	Number	1990	81.3987458	109.053899	35.6706876
Incidence	China	Both	40-44 years	Larynx cancer	Number	1990	539.512454	655.030002	421.944853
Incidence	China	Male	40-44 years	Larynx cancer	Rate	1990	1.3021973	1.62721475	0.98635434
Incidence	China	Female	40-44 years	Larynx cancer	Rate	1990	0.25505429	0.34170877	0.1117703
Incidence	China	Both	40-44 years	Larynx cancer	Rate	1990	0.80411029	0.97628212	0.62888297

Incidence	China	Male	40-44 years	Larynx cancer	Number	2000	544.511362	658.898051	448.833582
Incidence	China	Female	40-44 years	Larynx cancer	Number	2000	88.7395117	109.176867	53.6807331
Incidence	China	Both	40-44 years	Larynx cancer	Number	2000	633.250874	750.613861	525.984777
Incidence	China	Male	40-44 years	Larynx cancer	Rate	2000	1.27587377	1.54389935	1.05168603
Incidence	China	Female	40-44 years	Larynx cancer	Rate	2000	0.22505278	0.27688408	0.13614002
Incidence	China	Both	40-44 years	Larynx cancer	Rate	2000	0.77124083	0.91417806	0.64060067
Incidence	China	Male	40-44 years	Larynx cancer	Number	2010	714.145639	846.09596	592.30918
Incidence	China	Female	40-44 years	Larynx cancer	Number	2010	135.369602	168.503488	95.0965952
Incidence	China	Both	40-44 years	Larynx cancer	Number	2010	849.515241	972.366465	722.446269
Incidence	China	Male	40-44 years	Larynx cancer	Rate	2010	1.11833009	1.32496023	0.92753794
Incidence	China	Female	40-44 years	Larynx cancer	Rate	2010	0.22017129	0.27406176	0.15466944
Incidence	China	Both	40-44 years	Larynx cancer	Rate	2010	0.67775795	0.77577078	0.57638013
Incidence	China	Male	40-44 years	Larynx cancer	Number	2019	565.828089	747.775383	420.57359
Incidence	China	Female	40-44 years	Larynx cancer	Number	2019	108.898193	152.856033	71.567432
Incidence	China	Both	40-44 years	Larynx cancer	Number	2019	674.726282	870.997361	537.203429
Incidence	China	Male	40-44 years	Larynx cancer	Rate	2019	1.14576275	1.51419344	0.85163244
Incidence	China	Female	40-44 years	Larynx cancer	Rate	2019	0.23001924	0.32286879	0.15116767
Incidence	China	Both	40-44 years	Larynx cancer	Rate	2019	0.69755385	0.90046524	0.55537828
Incidence	China	Male	40-44 years	Larynx cancer	Number	2020	550.51185	730.688099	405.049134
Incidence	China	Female	40-44 years	Larynx cancer	Number	2020	105.07294	158.140126	63.5947161
Incidence	China	Both	40-44 years	Larynx cancer	Number	2020	655.58479	827.705266	508.257663
Incidence	China	Male	40-44 years	Larynx cancer	Rate	2020	1.14905698	1.52513023	0.84543963

Incidence	China	Female	40-44 years	Larynx cancer	Rate	2020	0.2297931	0.34585032	0.13908078
Incidence	China	Both	40-44 years	Larynx cancer	Rate	2020	0.70015002	0.88397088	0.54280792
Incidence	China	Male	40-44 years	Larynx cancer	Number	2021	544.992264	719.385938	393.056903
Incidence	China	Female	40-44 years	Larynx cancer	Number	2021	103.27078	155.03069	63.2281586
Incidence	China	Both	40-44 years	Larynx cancer	Number	2021	648.263044	825.951191	496.93118
Incidence	China	Male	40-44 years	Larynx cancer	Rate	2021	1.16140373	1.53304472	0.83762245
Incidence	China	Female	40-44 years	Larynx cancer	Rate	2021	0.23150535	0.34753716	0.14174055
Incidence	China	Both	40-44 years	Larynx cancer	Rate	2021	0.70822348	0.90234671	0.54289433
Incidence	China	Male	45-49 years	Larynx cancer	Number	1990	711.282898	878.049219	545.576808
Incidence	China	Female	45-49 years	Larynx cancer	Number	1990	111.726161	151.409024	52.3573289
Incidence	China	Both	45-49 years	Larynx cancer	Number	1990	823.009059	991.340961	648.451886
Incidence	China	Male	45-49 years	Larynx cancer	Rate	1990	2.61128273	3.22352016	2.00293765
Incidence	China	Female	45-49 years	Larynx cancer	Rate	1990	0.45826739	0.62103466	0.21475415
Incidence	China	Both	45-49 years	Larynx cancer	Rate	1990	1.59439308	1.92049789	1.25622821
Incidence	China	Male	45-49 years	Larynx cancer	Number	2000	1073.70131	1250.7844	903.723348
Incidence	China	Female	45-49 years	Larynx cancer	Number	2000	159.878868	197.999796	91.7708506
Incidence	China	Both	45-49 years	Larynx cancer	Number	2000	1233.58017	1410.98694	1037.1183
Incidence	China	Male	45-49 years	Larynx cancer	Rate	2000	2.42510067	2.82506696	2.04118229
Incidence	China	Female	45-49 years	Larynx cancer	Rate	2000	0.38154554	0.47251985	0.21900805
Incidence	China	Both	45-49 years	Larynx cancer	Rate	2000	1.43144175	1.63730388	1.20346813
Incidence	China	Male	45-49 years	Larynx cancer	Number	2010	1423.94864	1673.22221	1195.67369
Incidence	China	Female	45-49 years	Larynx cancer	Number	2010	231.733856	286.820892	165.206513

Incidence	China	Both	45-49 years	Larynx cancer	Number	2010	1655.6825	1899.91506	1416.42655
Incidence	China	Male	45-49 years	Larynx cancer	Rate	2010	2.63243707	3.09326617	2.21042785
Incidence	China	Female	45-49 years	Larynx cancer	Rate	2010	0.44401437	0.54956406	0.31654445
Incidence	China	Both	45-49 years	Larynx cancer	Rate	2010	1.55780514	1.78759964	1.33269305
Incidence	China	Male	45-49 years	Larynx cancer	Number	2019	1433.90784	1959.88945	1046.50648
Incidence	China	Female	45-49 years	Larynx cancer	Number	2019	227.008084	318.685745	150.894244
Incidence	China	Both	45-49 years	Larynx cancer	Number	2019	1660.91592	2189.96584	1255.72708
Incidence	China	Male	45-49 years	Larynx cancer	Rate	2019	2.38078315	3.25409462	1.73756285
Incidence	China	Female	45-49 years	Larynx cancer	Rate	2019	0.3922589	0.55067343	0.2607379
Incidence	China	Both	45-49 years	Larynx cancer	Rate	2019	1.40635911	1.8543253	1.06327069
Incidence	China	Male	45-49 years	Larynx cancer	Number	2020	1414.67687	1885.86067	1002.44741
Incidence	China	Female	45-49 years	Larynx cancer	Number	2020	225.450832	331.888431	142.558261
Incidence	China	Both	45-49 years	Larynx cancer	Number	2020	1640.1277	2111.64891	1219.5119
Incidence	China	Male	45-49 years	Larynx cancer	Rate	2020	2.42005859	3.22610302	1.71486614
Incidence	China	Female	45-49 years	Larynx cancer	Rate	2020	0.39969277	0.58839174	0.25273585
Incidence	China	Both	45-49 years	Larynx cancer	Rate	2020	1.42790729	1.83841713	1.06171607
Incidence	China	Male	45-49 years	Larynx cancer	Number	2021	1368.23111	1838.73372	993.343868
Incidence	China	Female	45-49 years	Larynx cancer	Number	2021	219.780163	323.214103	138.475199
Incidence	China	Both	45-49 years	Larynx cancer	Number	2021	1588.01127	2076.49533	1177.72311
Incidence	China	Male	45-49 years	Larynx cancer	Rate	2021	2.44059036	3.27985218	1.77188301
Incidence	China	Female	45-49 years	Larynx cancer	Rate	2021	0.40504947	0.59567569	0.25520641
Incidence	China	Both	45-49 years	Larynx cancer	Rate	2021	1.43943873	1.88222076	1.06753666

Incidence	China	Male	50-54 years	Larynx cancer	Number	1990	1414.5177	1763.51852	1084.4251
Incidence	China	Female	50-54 years	Larynx cancer	Number	1990	191.497014	261.669016	95.9689328
Incidence	China	Both	50-54 years	Larynx cancer	Number	1990	1606.01471	1953.54806	1268.34241
Incidence	China	Male	50-54 years	Larynx cancer	Rate	1990	5.60081055	6.98268617	4.29380245
Incidence	China	Female	50-54 years	Larynx cancer	Rate	1990	0.85279253	1.1652891	0.42737789
Incidence	China	Both	50-54 years	Larynx cancer	Rate	1990	3.36613961	4.09455496	2.65839259
Incidence	China	Male	50-54 years	Larynx cancer	Number	2000	1630.99368	1909.81398	1370.10084
Incidence	China	Female	50-54 years	Larynx cancer	Number	2000	225.405642	280.058937	144.88531
Incidence	China	Both	50-54 years	Larynx cancer	Number	2000	1856.39932	2142.18752	1587.7047
Incidence	China	Male	50-54 years	Larynx cancer	Rate	2000	4.93474142	5.77834129	4.14538294
Incidence	China	Female	50-54 years	Larynx cancer	Rate	2000	0.73374593	0.91165467	0.47163419
Incidence	China	Both	50-54 years	Larynx cancer	Rate	2000	2.91103553	3.35918242	2.48969322
Incidence	China	Male	50-54 years	Larynx cancer	Number	2010	1894.93334	2254.63465	1563.6005
Incidence	China	Female	50-54 years	Larynx cancer	Number	2010	262.903452	333.314688	179.162467
Incidence	China	Both	50-54 years	Larynx cancer	Number	2010	2157.83679	2534.72199	1805.24373
Incidence	China	Male	50-54 years	Larynx cancer	Rate	2010	4.65879207	5.54313645	3.84419308
Incidence	China	Female	50-54 years	Larynx cancer	Rate	2010	0.67921555	0.86112418	0.46286929
Incidence	China	Both	50-54 years	Larynx cancer	Rate	2010	2.71831968	3.19309815	2.27414307
Incidence	China	Male	50-54 years	Larynx cancer	Number	2019	3064.83053	4110.09433	2215.10016
Incidence	China	Female	50-54 years	Larynx cancer	Number	2019	414.337614	567.6396	276.268307
Incidence	China	Both	50-54 years	Larynx cancer	Number	2019	3479.16815	4544.71345	2592.41547
Incidence	China	Male	50-54 years	Larynx cancer	Rate	2019	4.98390854	6.68367598	3.60210996

Incidence	China	Female	50-54 years	Larynx cancer	Rate	2019	0.68111233	0.93311908	0.45414595
Incidence	China	Both	50-54 years	Larynx cancer	Rate	2019	2.84415364	3.71521662	2.11925023
Incidence	China	Male	50-54 years	Larynx cancer	Number	2020	3097.76636	4173.3358	2174.03804
Incidence	China	Female	50-54 years	Larynx cancer	Number	2020	421.246478	609.039891	272.019466
Incidence	China	Both	50-54 years	Larynx cancer	Number	2020	3519.01284	4572.34312	2557.24574
Incidence	China	Male	50-54 years	Larynx cancer	Rate	2020	5.04607382	6.79811132	3.54137632
Incidence	China	Female	50-54 years	Larynx cancer	Rate	2020	0.69698521	1.00770409	0.45007746
Incidence	China	Both	50-54 years	Larynx cancer	Rate	2020	2.88850902	3.75311342	2.09906235
Incidence	China	Male	50-54 years	Larynx cancer	Number	2021	3088.64313	4193.4013	2151.65212
Incidence	China	Female	50-54 years	Larynx cancer	Number	2021	418.982645	613.490601	274.44434
Incidence	China	Both	50-54 years	Larynx cancer	Number	2021	3507.62577	4652.35762	2588.08553
Incidence	China	Male	50-54 years	Larynx cancer	Rate	2021	5.05166956	6.85857084	3.519162
Incidence	China	Female	50-54 years	Larynx cancer	Rate	2021	0.70160197	1.02731276	0.45956722
Incidence	China	Both	50-54 years	Larynx cancer	Rate	2021	2.90224543	3.84940826	2.14140843
Incidence	China	Male	55-59 years	Larynx cancer	Number	1990	2055.99806	2518.4215	1588.22401
Incidence	China	Female	55-59 years	Larynx cancer	Number	1990	338.866775	459.553599	174.113035
Incidence	China	Both	55-59 years	Larynx cancer	Number	1990	2394.86484	2878.82249	1897.43723
Incidence	China	Male	55-59 years	Larynx cancer	Rate	1990	9.04580498	11.0803362	6.98773256
Incidence	China	Female	55-59 years	Larynx cancer	Rate	1990	1.64176808	2.22648099	0.84355636
Incidence	China	Both	55-59 years	Larynx cancer	Rate	1990	5.52205307	6.63795731	4.37508995
Incidence	China	Male	55-59 years	Larynx cancer	Number	2000	1924.69332	2338.00128	1583.45897
Incidence	China	Female	55-59 years	Larynx cancer	Number	2000	327.908286	418.211894	206.214563

Incidence	China	Both	55-59 years	Larynx cancer	Number	2000	2252.60161	2692.6835	1866.10644
Incidence	China	Male	55-59 years	Larynx cancer	Rate	2000	7.92614125	9.62819801	6.52089314
Incidence	China	Female	55-59 years	Larynx cancer	Rate	2000	1.45812379	1.85968071	0.91698311
Incidence	China	Both	55-59 years	Larynx cancer	Rate	2000	4.81621254	5.75713699	3.98986009
Incidence	China	Male	55-59 years	Larynx cancer	Number	2010	3154.30859	3713.09466	2697.95377
Incidence	China	Female	55-59 years	Larynx cancer	Number	2010	519.681897	637.380795	351.017228
Incidence	China	Both	55-59 years	Larynx cancer	Number	2010	3673.99049	4265.00888	3153.23134
Incidence	China	Male	55-59 years	Larynx cancer	Rate	2010	7.6327492	8.98489145	6.52846857
Incidence	China	Female	55-59 years	Larynx cancer	Rate	2010	1.28383608	1.57460259	0.86716236
Incidence	China	Both	55-59 years	Larynx cancer	Rate	2010	4.49116639	5.2136402	3.85457901
Incidence	China	Male	55-59 years	Larynx cancer	Number	2019	3897.58303	5325.06132	2845.88656
Incidence	China	Female	55-59 years	Larynx cancer	Number	2019	616.048384	907.42398	396.616605
Incidence	China	Both	55-59 years	Larynx cancer	Number	2019	4513.63141	5976.56668	3412.58263
Incidence	China	Male	55-59 years	Larynx cancer	Rate	2019	8.33490408	11.3875381	6.08587202
Incidence	China	Female	55-59 years	Larynx cancer	Rate	2019	1.32932062	1.95805627	0.85582666
Incidence	China	Both	55-59 years	Larynx cancer	Rate	2019	4.84787898	6.41914888	3.66529433
Incidence	China	Male	55-59 years	Larynx cancer	Number	2020	4265.26679	5688.49825	3031.71544
Incidence	China	Female	55-59 years	Larynx cancer	Number	2020	676.83696	987.832459	409.722247
Incidence	China	Both	55-59 years	Larynx cancer	Number	2020	4942.10375	6367.97634	3624.74063
Incidence	China	Male	55-59 years	Larynx cancer	Rate	2020	8.34218204	11.1257959	5.92955219
Incidence	China	Female	55-59 years	Larynx cancer	Rate	2020	1.32834933	1.9387041	0.80411429
Incidence	China	Both	55-59 years	Larynx cancer	Rate	2020	4.84130081	6.23809021	3.55080764

Incidence	China	Male	55-59 years	Larynx cancer	Number	2021	4603.47062	6172.95485	3279.74577
Incidence	China	Female	55-59 years	Larynx cancer	Number	2021	740.670482	1063.34886	473.388811
Incidence	China	Both	55-59 years	Larynx cancer	Number	2021	5344.1411	6877.28819	4008.88195
Incidence	China	Male	55-59 years	Larynx cancer	Rate	2021	8.38770397	11.2473658	5.97582538
Incidence	China	Female	55-59 years	Larynx cancer	Rate	2021	1.34523509	1.93129635	0.85978752
Incidence	China	Both	55-59 years	Larynx cancer	Rate	2021	4.86085654	6.25535715	3.64634834
Incidence	China	Male	60-64 years	Larynx cancer	Number	1990	2183.86233	2694.76328	1702.81087
Incidence	China	Female	60-64 years	Larynx cancer	Number	1990	396.333206	521.579077	216.683152
Incidence	China	Both	60-64 years	Larynx cancer	Number	1990	2580.19554	3120.302	2076.15764
Incidence	China	Male	60-64 years	Larynx cancer	Rate	1990	11.9973207	14.804019	9.35460438
Incidence	China	Female	60-64 years	Larynx cancer	Rate	1990	2.31306527	3.04402062	1.26459824
Incidence	China	Both	60-64 years	Larynx cancer	Rate	1990	7.30158705	8.83001166	5.87523135
Incidence	China	Male	60-64 years	Larynx cancer	Number	2000	2325.60011	2717.3081	1972.45954
Incidence	China	Female	60-64 years	Larynx cancer	Number	2000	410.799091	501.397525	268.989513
Incidence	China	Both	60-64 years	Larynx cancer	Number	2000	2736.3992	3137.99608	2351.96195
Incidence	China	Male	60-64 years	Larynx cancer	Rate	2000	10.6582653	12.4534698	9.03981605
Incidence	China	Female	60-64 years	Larynx cancer	Rate	2000	2.03718506	2.48646983	1.33394019
Incidence	China	Both	60-64 years	Larynx cancer	Rate	2000	6.51760701	7.47413801	5.60194715
Incidence	China	Male	60-64 years	Larynx cancer	Number	2010	3609.4368	4170.96347	3024.7668
Incidence	China	Female	60-64 years	Larynx cancer	Number	2010	632.798249	781.222704	425.097845
Incidence	China	Both	60-64 years	Larynx cancer	Number	2010	4242.23504	4880.56455	3613.53287
Incidence	China	Male	60-64 years	Larynx cancer	Rate	2010	12.0296115	13.9010801	10.0810103

Incidence	China	Female	60-64 years	Larynx cancer	Rate	2010	2.18219469	2.69403406	1.46594315
Incidence	China	Both	60-64 years	Larynx cancer	Rate	2010	7.18988172	8.27174391	6.12433626
Incidence	China	Male	60-64 years	Larynx cancer	Number	2019	5062.4922	6834.41937	3722.37114
Incidence	China	Female	60-64 years	Larynx cancer	Number	2019	851.783668	1164.67558	531.766391
Incidence	China	Both	60-64 years	Larynx cancer	Number	2019	5914.27587	7703.69244	4515.0014
Incidence	China	Male	60-64 years	Larynx cancer	Rate	2019	13.0666816	17.6401619	9.60772614
Incidence	China	Female	60-64 years	Larynx cancer	Rate	2019	2.21884676	3.03391193	1.38522042
Incidence	China	Both	60-64 years	Larynx cancer	Rate	2019	7.66772371	9.98766147	5.85359629
Incidence	China	Male	60-64 years	Larynx cancer	Number	2020	4919.3801	6669.39681	3430.24213
Incidence	China	Female	60-64 years	Larynx cancer	Number	2020	830.712544	1150.71029	496.64197
Incidence	China	Both	60-64 years	Larynx cancer	Number	2020	5750.09264	7452.20764	4202.24919
Incidence	China	Male	60-64 years	Larynx cancer	Rate	2020	13.2167878	17.9185183	9.21595433
Incidence	China	Female	60-64 years	Larynx cancer	Rate	2020	2.2532581	3.12123285	1.34711165
Incidence	China	Both	60-64 years	Larynx cancer	Rate	2020	7.76118002	10.0586075	5.67198035
Incidence	China	Male	60-64 years	Larynx cancer	Number	2021	4806.49351	6540.35307	3408.45257
Incidence	China	Female	60-64 years	Larynx cancer	Number	2021	815.671443	1131.87604	509.189407
Incidence	China	Both	60-64 years	Larynx cancer	Number	2021	5622.16495	7505.86835	4248.7043
Incidence	China	Male	60-64 years	Larynx cancer	Rate	2021	13.1234072	17.8574498	9.30626684
Incidence	China	Female	60-64 years	Larynx cancer	Rate	2021	2.2420987	3.11127455	1.399648
Incidence	China	Both	60-64 years	Larynx cancer	Rate	2021	7.70105081	10.2812838	5.81973101
Incidence	China	Male	65-69 years	Larynx cancer	Number	1990	2121.18672	2610.68661	1675.66315
Incidence	China	Female	65-69 years	Larynx cancer	Number	1990	456.89795	578.224442	256.338966

Incidence	China	Both	65-69 years	Larynx cancer	Number	1990	2578.08467	3086.1849	2070.22216
Incidence	China	Male	65-69 years	Larynx cancer	Rate	1990	15.8195926	19.4702325	12.4969236
Incidence	China	Female	65-69 years	Larynx cancer	Rate	1990	3.29338398	4.16792221	1.84772693
Incidence	China	Both	65-69 years	Larynx cancer	Rate	1990	9.44982952	11.3122433	7.58828702
Incidence	China	Male	65-69 years	Larynx cancer	Number	2000	2545.27063	2930.13396	2172.07996
Incidence	China	Female	65-69 years	Larynx cancer	Number	2000	518.362288	630.500819	342.704427
Incidence	China	Both	65-69 years	Larynx cancer	Number	2000	3063.63292	3489.62774	2653.76135
Incidence	China	Male	65-69 years	Larynx cancer	Rate	2000	14.2945833	16.4560278	12.1986942
Incidence	China	Female	65-69 years	Larynx cancer	Rate	2000	2.97197064	3.61490403	1.96485647
Incidence	China	Both	65-69 years	Larynx cancer	Rate	2000	8.6917631	9.90034332	7.52892582
Incidence	China	Male	65-69 years	Larynx cancer	Number	2010	3154.48009	3720.52077	2667.48541
Incidence	China	Female	65-69 years	Larynx cancer	Number	2010	603.726788	758.820475	404.311641
Incidence	China	Both	65-69 years	Larynx cancer	Number	2010	3758.20688	4412.16691	3235.42651
Incidence	China	Male	65-69 years	Larynx cancer	Rate	2010	15.1223264	17.8358803	12.7877126
Incidence	China	Female	65-69 years	Larynx cancer	Rate	2010	2.94884527	3.70638542	1.97482122
Incidence	China	Both	65-69 years	Larynx cancer	Rate	2010	9.09249109	10.6746621	7.82769222
Incidence	China	Male	65-69 years	Larynx cancer	Number	2019	5496.64937	7332.24046	4091.0926
Incidence	China	Female	65-69 years	Larynx cancer	Number	2019	1059.05409	1410.34782	650.178545
Incidence	China	Both	65-69 years	Larynx cancer	Number	2019	6555.70346	8427.41745	5101.0473
Incidence	China	Male	65-69 years	Larynx cancer	Rate	2019	16.0754311	21.4437775	11.9647576
Incidence	China	Female	65-69 years	Larynx cancer	Rate	2019	2.98476852	3.97483171	1.83242052
Incidence	China	Both	65-69 years	Larynx cancer	Rate	2019	9.409001	12.0953578	7.3212218

Incidence	China	Male	65-69 years	Larynx cancer	Number	2020	5985.32474	7942.76821	4248.06621
Incidence	China	Female	65-69 years	Larynx cancer	Number	2020	1166.61525	1659.80836	720.463141
Incidence	China	Both	65-69 years	Larynx cancer	Number	2020	7151.93998	9343.23978	5315.86489
Incidence	China	Male	65-69 years	Larynx cancer	Rate	2020	16.368363	21.7214802	11.6173963
Incidence	China	Female	65-69 years	Larynx cancer	Rate	2020	3.07743832	4.37844258	1.90052452
Incidence	China	Both	65-69 years	Larynx cancer	Rate	2020	9.60313297	12.5454596	7.13777766
Incidence	China	Male	65-69 years	Larynx cancer	Number	2021	6215.83072	8297.49049	4394.68823
Incidence	China	Female	65-69 years	Larynx cancer	Number	2021	1210.48997	1670.56131	740.196723
Incidence	China	Both	65-69 years	Larynx cancer	Number	2021	7426.32069	9533.94286	5615.98276
Incidence	China	Male	65-69 years	Larynx cancer	Rate	2021	16.4701026	21.9858819	11.6446167
Incidence	China	Female	65-69 years	Larynx cancer	Rate	2021	3.10673384	4.28751123	1.89972181
Incidence	China	Both	65-69 years	Larynx cancer	Rate	2021	9.68185223	12.4296041	7.32167618
Incidence	China	Male	70-74 years	Larynx cancer	Number	1990	1741.74158	2092.29339	1367.46336
Incidence	China	Female	70-74 years	Larynx cancer	Number	1990	434.258488	551.11695	239.395101
Incidence	China	Both	70-74 years	Larynx cancer	Number	1990	2176.00007	2572.4063	1743.94826
Incidence	China	Male	70-74 years	Larynx cancer	Rate	1990	19.9619292	23.979569	15.6723632
Incidence	China	Female	70-74 years	Larynx cancer	Rate	1990	4.30285491	5.46074823	2.37204893
Incidence	China	Both	70-74 years	Larynx cancer	Rate	1990	11.5636116	13.6701776	9.26761937
Incidence	China	Male	70-74 years	Larynx cancer	Number	2000	2264.03886	2585.93057	1968.28954
Incidence	China	Female	70-74 years	Larynx cancer	Number	2000	494.86404	596.385611	316.772016
Incidence	China	Both	70-74 years	Larynx cancer	Number	2000	2758.9029	3117.46521	2404.35939
Incidence	China	Male	70-74 years	Larynx cancer	Rate	2000	18.0387265	20.6033982	15.6823442

Incidence	China	Female	70-74 years	Larynx cancer	Rate	2000	3.73262678	4.49837677	2.38932639
Incidence	China	Both	70-74 years	Larynx cancer	Rate	2000	10.6897816	12.0790848	9.31604978
Incidence	China	Male	70-74 years	Larynx cancer	Number	2010	3013.66965	3475.87883	2560.24883
Incidence	China	Female	70-74 years	Larynx cancer	Number	2010	636.742725	780.435903	414.931131
Incidence	China	Both	70-74 years	Larynx cancer	Number	2010	3650.41237	4145.00214	3136.12574
Incidence	China	Male	70-74 years	Larynx cancer	Rate	2010	18.2247044	21.0198434	15.4827116
Incidence	China	Female	70-74 years	Larynx cancer	Rate	2010	3.81368923	4.67432117	2.48517702
Incidence	China	Both	70-74 years	Larynx cancer	Rate	2010	10.9844919	12.4727669	9.43694691
Incidence	China	Male	70-74 years	Larynx cancer	Number	2019	4590.45656	6054.85033	3404.30028
Incidence	China	Female	70-74 years	Larynx cancer	Number	2019	943.460297	1277.52871	589.224672
Incidence	China	Both	70-74 years	Larynx cancer	Number	2019	5533.91685	7096.17564	4283.78119
Incidence	China	Male	70-74 years	Larynx cancer	Rate	2019	19.6606041	25.9325001	14.5803798
Incidence	China	Female	70-74 years	Larynx cancer	Rate	2019	3.86523452	5.23386949	2.41397709
Incidence	China	Both	70-74 years	Larynx cancer	Rate	2019	11.5875643	14.858805	8.96988356
Incidence	China	Male	70-74 years	Larynx cancer	Number	2020	4853.49774	6284.63608	3546.5768
Incidence	China	Female	70-74 years	Larynx cancer	Number	2020	1014.63649	1397.28312	616.572665
Incidence	China	Both	70-74 years	Larynx cancer	Number	2020	5868.13423	7364.58325	4514.25496
Incidence	China	Male	70-74 years	Larynx cancer	Rate	2020	19.9329922	25.8105821	14.5655549
Incidence	China	Female	70-74 years	Larynx cancer	Rate	2020	3.95918665	5.4523021	2.40591215
Incidence	China	Both	70-74 years	Larynx cancer	Rate	2020	11.7417954	14.7361028	9.03276168
Incidence	China	Male	70-74 years	Larynx cancer	Number	2021	5160.89546	6659.35889	3824.03813
Incidence	China	Female	70-74 years	Larynx cancer	Number	2021	1089.19023	1525.61927	637.639138

Incidence	China	Both	70-74 years	Larynx cancer	Number	2021	6250.0857	7943.55374	4882.62611
Incidence	China	Male	70-74 years	Larynx cancer	Rate	2021	19.9597665	25.7550747	14.7894699
Incidence	China	Female	70-74 years	Larynx cancer	Rate	2021	3.9693594	5.55984712	2.32376203
Incidence	China	Both	70-74 years	Larynx cancer	Rate	2021	11.7270224	14.9044728	9.16126089
Incidence	China	Male	75-79 years	Larynx cancer	Number	1990	1044.03194	1236.39652	845.261494
Incidence	China	Female	75-79 years	Larynx cancer	Number	1990	297.489321	375.276492	164.421839
Incidence	China	Both	75-79 years	Larynx cancer	Number	1990	1341.52126	1541.70082	1104.37583
Incidence	China	Male	75-79 years	Larynx cancer	Rate	1990	21.3196508	25.247831	17.2606595
Incidence	China	Female	75-79 years	Larynx cancer	Rate	1990	4.5882955	5.7880378	2.53594307
Incidence	China	Both	75-79 years	Larynx cancer	Rate	1990	11.7876897	13.546629	9.70393838
Incidence	China	Male	75-79 years	Larynx cancer	Number	2000	1380.08361	1559.67512	1202.48322
Incidence	China	Female	75-79 years	Larynx cancer	Number	2000	352.023664	422.813558	244.901584
Incidence	China	Both	75-79 years	Larynx cancer	Number	2000	1732.10727	1941.32875	1525.13324
Incidence	China	Male	75-79 years	Larynx cancer	Rate	2000	18.9716545	21.4404529	16.5302277
Incidence	China	Female	75-79 years	Larynx cancer	Rate	2000	3.96680674	4.7645083	2.75969304
Incidence	China	Both	75-79 years	Larynx cancer	Rate	2000	10.725997	12.0215916	9.4443195
Incidence	China	Male	75-79 years	Larynx cancer	Number	2010	2252.62717	2561.60622	1936.99457
Incidence	China	Female	75-79 years	Larynx cancer	Number	2010	545.482616	665.937487	356.950431
Incidence	China	Both	75-79 years	Larynx cancer	Number	2010	2798.10979	3167.61569	2392.2911
Incidence	China	Male	75-79 years	Larynx cancer	Rate	2010	19.9037573	22.6338335	17.1148915
Incidence	China	Female	75-79 years	Larynx cancer	Rate	2010	4.3094803	5.2611108	2.8200181
Incidence	China	Both	75-79 years	Larynx cancer	Rate	2010	11.6707859	13.2119778	9.97813498

Incidence	China	Male	75-79 years	Larynx cancer	Number	2019	2952.4257	3846.96741	2313.51302
Incidence	China	Female	75-79 years	Larynx cancer	Number	2019	702.280325	972.301658	405.221988
Incidence	China	Both	75-79 years	Larynx cancer	Number	2019	3654.70603	4583.22186	2979.41921
Incidence	China	Male	75-79 years	Larynx cancer	Rate	2019	20.5396993	26.7629272	16.0948544
Incidence	China	Female	75-79 years	Larynx cancer	Rate	2019	4.39627869	6.08661371	2.53669187
Incidence	China	Both	75-79 years	Larynx cancer	Rate	2019	12.0423939	15.1018885	9.81729844
Incidence	China	Male	75-79 years	Larynx cancer	Number	2020	3041.87621	3791.51573	2262.85896
Incidence	China	Female	75-79 years	Larynx cancer	Number	2020	732.130466	1023.91518	423.70635
Incidence	China	Both	75-79 years	Larynx cancer	Number	2020	3774.00668	4649.69828	2948.35241
Incidence	China	Male	75-79 years	Larynx cancer	Rate	2020	20.5130263	25.5682535	15.2596892
Incidence	China	Female	75-79 years	Larynx cancer	Rate	2020	4.4201799	6.18180705	2.55809365
Incidence	China	Both	75-79 years	Larynx cancer	Rate	2020	12.0220542	14.8115595	9.39194215
Incidence	China	Male	75-79 years	Larynx cancer	Number	2021	3180.68851	4088.79404	2427.97556
Incidence	China	Female	75-79 years	Larynx cancer	Number	2021	766.032212	1063.78695	433.488904
Incidence	China	Both	75-79 years	Larynx cancer	Number	2021	3946.72073	4951.46274	3170.77407
Incidence	China	Male	75-79 years	Larynx cancer	Rate	2021	20.3858521	26.2061344	15.5615208
Incidence	China	Female	75-79 years	Larynx cancer	Rate	2021	4.37320215	6.0730545	2.47474528
Incidence	China	Both	75-79 years	Larynx cancer	Rate	2021	11.9168098	14.9505485	9.57390052
Incidence	China	Male	<1 year	Larynx cancer	Number	1990	0	0	0
Incidence	China	Female	<1 year	Larynx cancer	Number	1990	0	0	0
Incidence	China	Both	<1 year	Larynx cancer	Number	1990	0	0	0
Incidence	China	Male	<1 year	Larynx cancer	Rate	1990	0	0	0

Incidence	China	Female	<1 year	Larynx cancer	Rate	1990	0	0	0
Incidence	China	Both	<1 year	Larynx cancer	Rate	1990	0	0	0
Incidence	China	Male	<1 year	Larynx cancer	Number	2000	0	0	0
Incidence	China	Female	<1 year	Larynx cancer	Number	2000	0	0	0
Incidence	China	Both	<1 year	Larynx cancer	Number	2000	0	0	0
Incidence	China	Male	<1 year	Larynx cancer	Rate	2000	0	0	0
Incidence	China	Female	<1 year	Larynx cancer	Rate	2000	0	0	0
Incidence	China	Both	<1 year	Larynx cancer	Rate	2000	0	0	0
Incidence	China	Male	<1 year	Larynx cancer	Number	2010	0	0	0
Incidence	China	Female	<1 year	Larynx cancer	Number	2010	0	0	0
Incidence	China	Both	<1 year	Larynx cancer	Number	2010	0	0	0
Incidence	China	Male	<1 year	Larynx cancer	Rate	2010	0	0	0
Incidence	China	Female	<1 year	Larynx cancer	Rate	2010	0	0	0
Incidence	China	Both	<1 year	Larynx cancer	Rate	2010	0	0	0
Incidence	China	Male	<1 year	Larynx cancer	Number	2019	0	0	0
Incidence	China	Female	<1 year	Larynx cancer	Number	2019	0	0	0
Incidence	China	Both	<1 year	Larynx cancer	Number	2019	0	0	0
Incidence	China	Male	<1 year	Larynx cancer	Rate	2019	0	0	0
Incidence	China	Female	<1 year	Larynx cancer	Rate	2019	0	0	0
Incidence	China	Both	<1 year	Larynx cancer	Rate	2019	0	0	0
Incidence	China	Male	<1 year	Larynx cancer	Number	2020	0	0	0
Incidence	China	Female	<1 year	Larynx cancer	Number	2020	0	0	0

Incidence	China	Both	<1 year	Larynx cancer	Number	2020	0	0	0
Incidence	China	Male	<1 year	Larynx cancer	Rate	2020	0	0	0
Incidence	China	Female	<1 year	Larynx cancer	Rate	2020	0	0	0
Incidence	China	Both	<1 year	Larynx cancer	Rate	2020	0	0	0
Incidence	China	Male	<1 year	Larynx cancer	Number	2021	0	0	0
Incidence	China	Female	<1 year	Larynx cancer	Number	2021	0	0	0
Incidence	China	Both	<1 year	Larynx cancer	Number	2021	0	0	0
Incidence	China	Male	<1 year	Larynx cancer	Rate	2021	0	0	0
Incidence	China	Female	<1 year	Larynx cancer	Rate	2021	0	0	0
Incidence	China	Both	<1 year	Larynx cancer	Rate	2021	0	0	0
Incidence	China	Male	80-84	Larynx cancer	Number	1990	448.831008	540.345668	368.846885
Incidence	China	Female	80-84	Larynx cancer	Number	1990	159.48062	200.612431	99.1484596
Incidence	China	Both	80-84	Larynx cancer	Number	1990	608.311628	708.753599	492.721962
Incidence	China	Male	80-84	Larynx cancer	Rate	1990	22.0031512	26.4894966	18.0820702
Incidence	China	Female	80-84	Larynx cancer	Rate	1990	4.89613357	6.15890042	3.04390653
Incidence	China	Both	80-84	Larynx cancer	Rate	1990	11.4838062	13.3799661	9.30168563
Incidence	China	Male	80-84	Larynx cancer	Number	2000	669.353901	809.418449	567.418932
Incidence	China	Female	80-84	Larynx cancer	Number	2000	208.401741	266.056974	144.425842
Incidence	China	Both	80-84	Larynx cancer	Number	2000	877.755642	1037.02542	748.643031
Incidence	China	Male	80-84	Larynx cancer	Rate	2000	20.3970551	24.6652072	17.2908161
Incidence	China	Female	80-84	Larynx cancer	Rate	2000	4.30380137	5.49446641	2.9826053
Incidence	China	Both	80-84	Larynx cancer	Rate	2000	10.8046202	12.7651311	9.21532513

Incidence	China	Male	80-84	Larynx cancer	Number	2010	1142.7269	1323.83624	958.61122
Incidence	China	Female	80-84	Larynx cancer	Number	2010	318.301009	386.605647	182.631406
Incidence	China	Both	80-84	Larynx cancer	Number	2010	1461.02791	1659.26321	1226.59568
Incidence	China	Male	80-84	Larynx cancer	Rate	2010	21.43954	24.8374656	17.9852102
Incidence	China	Female	80-84	Larynx cancer	Rate	2010	4.37682656	5.31605561	2.51128952
Incidence	China	Both	80-84	Larynx cancer	Rate	2010	11.5932397	13.1662345	9.73302267
Incidence	China	Male	80-84	Larynx cancer	Number	2019	1690.75882	2075.26844	1321.83626
Incidence	China	Female	80-84	Larynx cancer	Number	2019	466.992996	640.23067	256.078164
Incidence	China	Both	80-84	Larynx cancer	Number	2019	2157.75182	2599.22465	1770.59047
Incidence	China	Male	80-84	Larynx cancer	Rate	2019	20.5414052	25.2128981	16.0592829
Incidence	China	Female	80-84	Larynx cancer	Rate	2019	4.40239701	6.0355286	2.41407848
Incidence	China	Both	80-84	Larynx cancer	Rate	2019	11.453839	13.7972775	9.39869821
Incidence	China	Male	80-84	Larynx cancer	Number	2020	1721.08932	2100.83088	1332.41354
Incidence	China	Female	80-84	Larynx cancer	Number	2020	482.986665	687.949364	257.37784
Incidence	China	Both	80-84	Larynx cancer	Number	2020	2204.07599	2622.83333	1739.53887
Incidence	China	Male	80-84	Larynx cancer	Rate	2020	20.3536493	24.8444834	15.757159
Incidence	China	Female	80-84	Larynx cancer	Rate	2020	4.44593572	6.33263581	2.36918618
Incidence	China	Both	80-84	Larynx cancer	Rate	2020	11.408568	13.5761074	9.00406677
Incidence	China	Male	80-84	Larynx cancer	Number	2021	1751.04564	2145.38416	1354.30482
Incidence	China	Female	80-84	Larynx cancer	Number	2021	491.565179	693.280326	268.021147
Incidence	China	Both	80-84	Larynx cancer	Number	2021	2242.61082	2695.40929	1829.67979
Incidence	China	Male	80-84	Larynx cancer	Rate	2021	20.1850219	24.7307239	15.6116276

Incidence	China	Female	80-84	Larynx cancer	Rate	2021	4.42179051	6.23628462	2.41093839
Incidence	China	Both	80-84	Larynx cancer	Rate	2021	11.3309779	13.6187799	9.24460947
Incidence	China	Male	85-89	Larynx cancer	Number	1990	162.140759	195.01138	133.834432
Incidence	China	Female	85-89	Larynx cancer	Number	1990	55.1391935	70.3877636	35.2623303
Incidence	China	Both	85-89	Larynx cancer	Number	1990	217.279953	252.730536	180.80703
Incidence	China	Male	85-89	Larynx cancer	Rate	1990	29.5543681	35.5458932	24.3948042
Incidence	China	Female	85-89	Larynx cancer	Rate	1990	4.84426311	6.1839288	3.09797795
Incidence	China	Both	85-89	Larynx cancer	Rate	1990	12.8807681	14.9823459	10.718584
Incidence	China	Male	85-89	Larynx cancer	Number	2000	331.890207	390.223376	286.467889
Incidence	China	Female	85-89	Larynx cancer	Number	2000	86.2025627	116.780465	62.0693501
Incidence	China	Both	85-89	Larynx cancer	Number	2000	418.092769	489.658281	363.368694
Incidence	China	Male	85-89	Larynx cancer	Rate	2000	33.2668751	39.1138758	28.7139882
Incidence	China	Female	85-89	Larynx cancer	Rate	2000	4.48442309	6.07514438	3.22896696
Incidence	China	Both	85-89	Larynx cancer	Rate	2000	14.3186074	16.7695431	12.4444479
Incidence	China	Male	85-89	Larynx cancer	Number	2010	541.028827	622.347011	430.506341
Incidence	China	Female	85-89	Larynx cancer	Number	2010	138.977973	174.539305	83.9219091
Incidence	China	Both	85-89	Larynx cancer	Number	2010	680.006801	776.214435	560.08938
Incidence	China	Male	85-89	Larynx cancer	Rate	2010	32.1226632	36.9507916	25.5605793
Incidence	China	Female	85-89	Larynx cancer	Rate	2010	4.40331567	5.53002492	2.65894406
Incidence	China	Both	85-89	Larynx cancer	Rate	2010	14.0483606	16.0359283	11.570969
Incidence	China	Male	85-89	Larynx cancer	Number	2019	995.29517	1182.77537	808.975594
Incidence	China	Female	85-89	Larynx cancer	Number	2019	261.332294	378.91023	144.872009

Incidence	China	Both	85-89	Larynx cancer	Number	2019	1256.62746	1509.48435	1026.57681
Incidence	China	Male	85-89	Larynx cancer	Rate	2019	32.1098951	38.1583215	26.0989124
Incidence	China	Female	85-89	Larynx cancer	Rate	2019	4.82390298	6.99426067	2.67417589
Incidence	China	Both	85-89	Larynx cancer	Rate	2019	14.7541748	17.72299	12.0531296
Incidence	China	Male	85-89	Larynx cancer	Number	2020	1040.10895	1251.75424	833.557067
Incidence	China	Female	85-89	Larynx cancer	Number	2020	275.062367	399.029463	147.967844
Incidence	China	Both	85-89	Larynx cancer	Number	2020	1315.17132	1536.43364	1066.26905
Incidence	China	Male	85-89	Larynx cancer	Rate	2020	31.5851469	38.0122115	25.3127544
Incidence	China	Female	85-89	Larynx cancer	Rate	2020	4.79186643	6.95149943	2.57775046
Incidence	China	Both	85-89	Larynx cancer	Rate	2020	14.5592674	17.0086953	11.8038585
Incidence	China	Male	85-89	Larynx cancer	Number	2021	1098.11272	1325.84519	881.996159
Incidence	China	Female	85-89	Larynx cancer	Number	2021	289.393423	427.532494	154.430962
Incidence	China	Both	85-89	Larynx cancer	Number	2021	1387.50614	1650.80744	1120.13837
Incidence	China	Male	85-89	Larynx cancer	Rate	2021	31.5466655	38.088981	25.3380525
Incidence	China	Female	85-89	Larynx cancer	Rate	2021	4.78744895	7.07269007	2.55475863
Incidence	China	Both	85-89	Larynx cancer	Rate	2021	14.5658451	17.3299453	11.7590557
Incidence	China	Male	90-94	Larynx cancer	Number	1990	25.3689335	30.2374979	21.1812262
Incidence	China	Female	90-94	Larynx cancer	Number	1990	10.2974234	13.2996905	7.12758826
Incidence	China	Both	90-94	Larynx cancer	Number	1990	35.6663569	41.9651614	29.8724103
Incidence	China	Male	90-94	Larynx cancer	Rate	1990	31.1355182	37.1107506	25.9959076
Incidence	China	Female	90-94	Larynx cancer	Rate	1990	4.56966658	5.90197651	3.1629953
Incidence	China	Both	90-94	Larynx cancer	Rate	1990	11.624443	13.6773607	9.73606955

Incidence	China	Male	90-94	Larynx cancer	Number	2000	55.9439546	67.6996942	47.9301409
Incidence	China	Female	90-94	Larynx cancer	Number	2000	21.0918535	28.9447464	15.0589032
Incidence	China	Both	90-94	Larynx cancer	Number	2000	77.0358081	91.9816497	64.8434553
Incidence	China	Male	90-94	Larynx cancer	Rate	2000	33.2208713	40.2017134	28.4620752
Incidence	China	Female	90-94	Larynx cancer	Rate	2000	4.41874793	6.06393072	3.15484351
Incidence	China	Both	90-94	Larynx cancer	Rate	2000	11.9300988	14.2446765	10.0419382
Incidence	China	Male	90-94	Larynx cancer	Number	2010	103.309982	120.436406	79.8498485
Incidence	China	Female	90-94	Larynx cancer	Number	2010	40.4302821	50.5766155	24.8877519
Incidence	China	Both	90-94	Larynx cancer	Number	2010	143.740264	164.73904	117.097315
Incidence	China	Male	90-94	Larynx cancer	Rate	2010	32.184207	37.51961	24.8756608
Incidence	China	Female	90-94	Larynx cancer	Rate	2010	4.33142379	5.41843252	2.66630345
Incidence	China	Both	90-94	Larynx cancer	Rate	2010	11.4587605	13.1327518	9.3348241
Incidence	China	Male	90-94	Larynx cancer	Number	2019	209.047759	255.923062	164.618174
Incidence	China	Female	90-94	Larynx cancer	Number	2019	89.9104142	128.965891	47.7428925
Incidence	China	Both	90-94	Larynx cancer	Number	2019	298.958173	368.31374	233.874749
Incidence	China	Male	90-94	Larynx cancer	Rate	2019	29.5207979	36.1403204	23.2466488
Incidence	China	Female	90-94	Larynx cancer	Rate	2019	4.84512954	6.94976723	2.57278872
Incidence	China	Both	90-94	Larynx cancer	Rate	2019	11.6606369	14.3657982	9.12210727
Incidence	China	Male	90-94	Larynx cancer	Number	2020	219.716758	265.369206	170.633405
Incidence	China	Female	90-94	Larynx cancer	Number	2020	95.9400567	138.970615	51.6113682
Incidence	China	Both	90-94	Larynx cancer	Number	2020	315.656815	374.200925	241.200362
Incidence	China	Male	90-94	Larynx cancer	Rate	2020	29.0017047	35.0276393	22.5229049

Incidence	China	Female	90-94	Larynx cancer	Rate	2020	4.82790054	6.99328652	2.59718996
Incidence	China	Both	90-94	Larynx cancer	Rate	2020	11.5001762	13.6330862	8.78753929
Incidence	China	Male	90-94	Larynx cancer	Number	2021	233.367263	284.969591	183.157678
Incidence	China	Female	90-94	Larynx cancer	Number	2021	102.204931	145.904117	54.5695899
Incidence	China	Both	90-94	Larynx cancer	Number	2021	335.572194	401.237165	261.944331
Incidence	China	Male	90-94	Larynx cancer	Rate	2021	28.8522822	35.2321184	22.6446372
Incidence	China	Female	90-94	Larynx cancer	Rate	2021	4.81382831	6.87204972	2.57021489
Incidence	China	Both	90-94	Larynx cancer	Rate	2021	11.4452123	13.6848184	8.93401937
Incidence	China	Male	2-4 years	Larynx cancer	Number	1990	0	0	0
Incidence	China	Female	2-4 years	Larynx cancer	Number	1990	0	0	0
Incidence	China	Both	2-4 years	Larynx cancer	Number	1990	0	0	0
Incidence	China	Male	2-4 years	Larynx cancer	Rate	1990	0	0	0
Incidence	China	Female	2-4 years	Larynx cancer	Rate	1990	0	0	0
Incidence	China	Both	2-4 years	Larynx cancer	Rate	1990	0	0	0
Incidence	China	Male	2-4 years	Larynx cancer	Number	2000	0	0	0
Incidence	China	Female	2-4 years	Larynx cancer	Number	2000	0	0	0
Incidence	China	Both	2-4 years	Larynx cancer	Number	2000	0	0	0
Incidence	China	Male	2-4 years	Larynx cancer	Rate	2000	0	0	0
Incidence	China	Female	2-4 years	Larynx cancer	Rate	2000	0	0	0
Incidence	China	Both	2-4 years	Larynx cancer	Rate	2000	0	0	0
Incidence	China	Male	2-4 years	Larynx cancer	Number	2010	0	0	0
Incidence	China	Female	2-4 years	Larynx cancer	Number	2010	0	0	0

Incidence	China	Both	2-4 years	Larynx cancer	Number	2010	0	0	0
Incidence	China	Male	2-4 years	Larynx cancer	Rate	2010	0	0	0
Incidence	China	Female	2-4 years	Larynx cancer	Rate	2010	0	0	0
Incidence	China	Both	2-4 years	Larynx cancer	Rate	2010	0	0	0
Incidence	China	Male	2-4 years	Larynx cancer	Number	2019	0	0	0
Incidence	China	Female	2-4 years	Larynx cancer	Number	2019	0	0	0
Incidence	China	Both	2-4 years	Larynx cancer	Number	2019	0	0	0
Incidence	China	Male	2-4 years	Larynx cancer	Rate	2019	0	0	0
Incidence	China	Female	2-4 years	Larynx cancer	Rate	2019	0	0	0
Incidence	China	Both	2-4 years	Larynx cancer	Rate	2019	0	0	0
Incidence	China	Male	2-4 years	Larynx cancer	Number	2020	0	0	0
Incidence	China	Female	2-4 years	Larynx cancer	Number	2020	0	0	0
Incidence	China	Both	2-4 years	Larynx cancer	Number	2020	0	0	0
Incidence	China	Male	2-4 years	Larynx cancer	Rate	2020	0	0	0
Incidence	China	Female	2-4 years	Larynx cancer	Rate	2020	0	0	0
Incidence	China	Both	2-4 years	Larynx cancer	Rate	2020	0	0	0
Incidence	China	Male	2-4 years	Larynx cancer	Number	2021	0	0	0
Incidence	China	Female	2-4 years	Larynx cancer	Number	2021	0	0	0
Incidence	China	Both	2-4 years	Larynx cancer	Number	2021	0	0	0
Incidence	China	Male	2-4 years	Larynx cancer	Rate	2021	0	0	0
Incidence	China	Female	2-4 years	Larynx cancer	Rate	2021	0	0	0
Incidence	China	Both	2-4 years	Larynx cancer	Rate	2021	0	0	0

Incidence	China	Male	95+ years	Larynx cancer	Number	1990	1.10157413	1.48000364	0.84899225
Incidence	China	Female	95+ years	Larynx cancer	Number	1990	1.29826232	1.79082575	0.88518988
Incidence	China	Both	95+ years	Larynx cancer	Number	1990	2.39983645	3.06720109	1.8227351
Incidence	China	Male	95+ years	Larynx cancer	Rate	1990	14.1786366	19.0494977	10.927592
Incidence	China	Female	95+ years	Larynx cancer	Rate	1990	3.96745085	5.47270996	2.70511383
Incidence	China	Both	95+ years	Larynx cancer	Rate	1990	5.92668022	7.57481619	4.50146012
Incidence	China	Male	95+ years	Larynx cancer	Number	2000	1.68697924	2.35454032	1.24065241
Incidence	China	Female	95+ years	Larynx cancer	Number	2000	2.51836021	3.76505372	1.77458362
Incidence	China	Both	95+ years	Larynx cancer	Number	2000	4.20533945	5.55178811	3.13164932
Incidence	China	Male	95+ years	Larynx cancer	Rate	2000	10.9716713	15.3133138	8.0688784
Incidence	China	Female	95+ years	Larynx cancer	Rate	2000	3.59769472	5.37870391	2.53514573
Incidence	China	Both	95+ years	Larynx cancer	Rate	2000	4.9257243	6.50282288	3.66810844
Incidence	China	Male	95+ years	Larynx cancer	Number	2010	3.6610377	4.41192521	2.66643711
Incidence	China	Female	95+ years	Larynx cancer	Number	2010	5.14836183	6.59004752	3.04416045
Incidence	China	Both	95+ years	Larynx cancer	Number	2010	8.80939954	10.7332301	6.24410986
Incidence	China	Male	95+ years	Larynx cancer	Rate	2010	12.338439	14.8690821	8.98643344
Incidence	China	Female	95+ years	Larynx cancer	Rate	2010	3.14978165	4.03180884	1.86242557
Incidence	China	Both	95+ years	Larynx cancer	Rate	2010	4.56154402	5.55771154	3.23322627
Incidence	China	Male	95+ years	Larynx cancer	Number	2019	15.1341561	17.7632664	11.9495033
Incidence	China	Female	95+ years	Larynx cancer	Number	2019	13.1634025	18.9278377	6.44607156
Incidence	China	Both	95+ years	Larynx cancer	Number	2019	28.2975586	34.989345	20.6692395
Incidence	China	Male	95+ years	Larynx cancer	Rate	2019	14.4500177	16.9602792	11.4093269

Incidence	China	Female	95+ years	Larynx cancer	Rate	2019	3.02233222	4.34585311	1.48002538
Incidence	China	Both	95+ years	Larynx cancer	Rate	2019	5.23764631	6.47624115	3.82570693
Incidence	China	Male	95+ years	Larynx cancer	Number	2020	16.4208892	19.4119536	13.009394
Incidence	China	Female	95+ years	Larynx cancer	Number	2020	14.7634746	21.8806839	7.25619364
Incidence	China	Both	95+ years	Larynx cancer	Number	2020	31.1843638	39.441222	22.0367864
Incidence	China	Male	95+ years	Larynx cancer	Rate	2020	14.566087	17.2192992	11.5399332
Incidence	China	Female	95+ years	Larynx cancer	Rate	2020	3.09967937	4.59397984	1.52348105
Incidence	China	Both	95+ years	Larynx cancer	Rate	2020	5.2942426	6.69602879	3.74123693
Incidence	China	Male	95+ years	Larynx cancer	Number	2021	17.4486874	21.0202155	13.6620119
Incidence	China	Female	95+ years	Larynx cancer	Number	2021	15.8647121	24.0386164	7.70114269
Incidence	China	Both	95+ years	Larynx cancer	Number	2021	33.3133995	43.3062991	23.3183206
Incidence	China	Male	95+ years	Larynx cancer	Rate	2021	14.4271173	17.3801678	11.2961763
Incidence	China	Female	95+ years	Larynx cancer	Rate	2021	3.06177095	4.63927344	1.48626302
Incidence	China	Both	95+ years	Larynx cancer	Rate	2021	5.21256144	6.7761546	3.64862731

Appendix 24：Percentage contribution of risk factors of tracheal, bronchus, and lung cancer,2021

measure	location	sex	age	cause	rei	metric	year	val	upper	lower
Deaths	Inner Mongolia	Both	All ages	Tracheal, bronchus, and lung cancer	Occupational exposure to diesel engine exhaust	Percent	2021	0.01299522	0.01517597	0.01104222
Deaths	Inner Mongolia	Both	All ages	Tracheal, bronchus, and lung cancer	Occupational exposure to nickel	Percent	2021	0.00560324	0.01298215	0.00116935
Deaths	Inner Mongolia	Both	All ages	Tracheal, bronchus, and lung cancer	Occupational exposure to polycyclic aromatic hydrocarbons	Percent	2021	0.00409337	0.00488787	0.00333442
Deaths	Inner Mongolia	Both	All ages	Tracheal, bronchus, and lung cancer	Occupational exposure to silica	Percent	2021	0.02634467	0.04136513	0.01193503
Deaths	Inner Mongolia	Both	All ages	Tracheal, bronchus, and lung cancer	All risk factors	Percent	2021	0.80871645	0.94306905	0.56853213
Deaths	Inner Mongolia	Both	All ages	Tracheal, bronchus, and lung cancer	Environmental/occupational risks	Percent	2021	0.32885955	0.46841436	0.23164074
Deaths	Macao Special Administrative Region of China	Both	All ages	Larynx cancer	Occupational exposure to sulfuric acid	Percent	2021	0.04380378	0.08103837	0.01878436
Deaths	Macao Special Administrative Region of China	Both	All ages	Larynx cancer	All risk factors	Percent	2021	0.83051125	0.89855133	0.73488035
Deaths	Macao Special Administrative Region of China	Both	All ages	Larynx cancer	Environmental/occupational risks	Percent	2021	0.05137727	0.08907425	0.0268109
Deaths	Macao Special Administrative Region of China	Both	All ages	Larynx cancer	Behavioral risks	Percent	2021	0.82205673	0.89329284	0.72471732

Deaths	Macao Special Administrative Region of China	Both	All ages	Tracheal, bronchus, and lung cancer	Occupational exposure to chromium	Percent	2021	0.00143217	0.0016984 6	0.0011967
Deaths	Hainan	Both	All ages	Larynx cancer	Tobacco	Percent	2021	0.7560132 8	0.8610391 6	0.6359824 3
Deaths	Macao Special Administrative Region of China	Both	All ages	Tracheal, bronchus, and lung cancer	Occupational exposure to diesel engine exhaust	Percent	2021	0.0180037 7	0.0214192 5	0.0150682 7
Deaths	Macao Special Administrative Region of China	Both	All ages	Tracheal, bronchus, and lung cancer	Occupational exposure to nickel	Percent	2021	0.0058752 2	0.012778	0.0013090 4
Deaths	Macao Special Administrative Region of China	Both	All ages	Tracheal, bronchus, and lung cancer	Occupational exposure to polycyclic aromatic hydrocarbons	Percent	2021	0.0049077 3	0.0059341 4	0.0039420 5
Deaths	Macao Special Administrative Region of China	Both	All ages	Tracheal, bronchus, and lung cancer	Occupational exposure to silica	Percent	2021	0.0280400 2	0.0432415 7	0.0130994 3
Deaths	Macao Special Administrative Region of China	Both	All ages	Tracheal, bronchus, and lung cancer	All risk factors	Percent	2021	0.7292739 6	0.8891782 4	0.4986200 1
Deaths	Inner Mongolia	Both	All ages	Tracheal, bronchus, and lung cancer	Occupational risks	Percent	2021	0.1020157 9	0.1323722 5	0.0762593 6
Deaths	Inner Mongolia	Both	All ages	Tracheal, bronchus, and lung cancer	Occupational carcinogens	Percent	2021	0.1020157 9	0.1323722 5	0.0762593 6
Deaths	Inner Mongolia	Both	All ages	Tracheal, bronchus, and lung cancer	Occupational exposure to asbestos	Percent	2021	0.0484182 4	0.0730527 7	0.0299121 5
Deaths	Inner Mongolia	Both	All ages	Tracheal, bronchus, and lung cancer	Occupational exposure to arsenic	Percent	2021	0.0058475 9	0.0096312 4	0.0021670 1

Deaths	Inner Mongolia	Both	All ages	Tracheal, bronchus, and lung cancer	Occupational exposure to beryllium	Percent	2021	0.00020903	0.00025178	0.00017059
Deaths	Inner Mongolia	Both	All ages	Tracheal, bronchus, and lung cancer	Occupational exposure to cadmium	Percent	2021	0.005689	0.0069125	0.0045824
Deaths	Macao Special Administrative Region of China	Both	All ages	Tracheal, bronchus, and lung cancer	Environmental/occupational risks	Percent	2021	0.25794482	0.35039499	0.18809206
Deaths	Tibet	Both	All ages	Larynx cancer	Occupational exposure to sulfuric acid	Percent	2021	0.04704362	0.08825363	0.01914773
Deaths	Tibet	Both	All ages	Larynx cancer	All risk factors	Percent	2021	0.73353288	0.84591636	0.58186838
Deaths	China	Both	All ages	Tracheal, bronchus, and lung cancer	Occupational exposure to diesel engine exhaust	Percent	2021	0.01431082	0.01613146	0.01259098
Deaths	China	Both	All ages	Tracheal, bronchus, and lung cancer	Occupational exposure to nickel	Percent	2021	0.00617757	0.01378941	0.00130666
Deaths	China	Both	All ages	Tracheal, bronchus, and lung cancer	Occupational exposure to polycyclic aromatic hydrocarbons	Percent	2021	0.00452152	0.00529566	0.00383371
Deaths	China	Both	All ages	Tracheal, bronchus, and lung cancer	Occupational exposure to silica	Percent	2021	0.02880944	0.04579575	0.0133578
Deaths	China	Both	All ages	Tracheal, bronchus, and lung cancer	All risk factors	Percent	2021	0.78221965	0.90611291	0.56992118
Deaths	Hainan	Both	All ages	Larynx cancer	Smoking	Percent	2021	0.75601328	0.86103916	0.63598243

Deaths	Hainan	Both	All ages	Larynx cancer	High alcohol use	Percent	2021	0.14996792	0.22421991	0.07450194
Deaths	Hainan	Both	All ages	Larynx cancer	Occupational risks	Percent	2021	0.04442569	0.07876144	0.02128658
Deaths	Hainan	Both	All ages	Larynx cancer	Occupational carcinogens	Percent	2021	0.04442569	0.07876144	0.02128658
Deaths	Hainan	Both	All ages	Larynx cancer	Occupational exposure to asbestos	Percent	2021	0.00463135	0.00840409	0.00215133
Deaths	Chongqing	Both	All ages	Larynx cancer	Occupational exposure to sulfuric acid	Percent	2021	0.04073061	0.07558784	0.01710966
Deaths	Chongqing	Both	All ages	Larynx cancer	All risk factors	Percent	2021	0.7995097	0.87369375	0.70676603
Deaths	China	Both	All ages	Tracheal, bronchus, and lung cancer	Environmental/occupational risks	Percent	2021	0.35203313	0.4379123	0.25782159
Deaths	Inner Mongolia	Both	All ages	Tracheal, bronchus, and lung cancer	Tobacco	Percent	2021	0.70380289	0.90939918	0.32885485
Deaths	Inner Mongolia	Both	All ages	Tracheal, bronchus, and lung cancer	Smoking	Percent	2021	0.67463248	0.89998744	0.3213772
Deaths	Inner Mongolia	Both	All ages	Tracheal, bronchus, and lung cancer	Secondhand smoke	Percent	2021	0.08312798	0.27964877	-0.115021
Deaths	Inner Mongolia	Both	All ages	Tracheal, bronchus, and lung cancer	Metabolic risks	Percent	2021	0.02052663	0.04541333	-0.0042053

Deaths	Inner Mongolia	Both	All ages	Tracheal, bronchus, and lung cancer	High fasting plasma glucose	Percent	2021	0.02052663	0.04541333	-0.0042053
Deaths	Inner Mongolia	Both	All ages	Tracheal, bronchus, and lung cancer	Dietary risks	Percent	2021	0.02079788	0.03182004	0.01078711
Deaths	Chongqing	Both	All ages	Larynx cancer	Environmental/occupational risks	Percent	2021	0.04629317	0.08107849	0.02313472
Deaths	Chongqing	Both	All ages	Larynx cancer	Behavioral risks	Percent	2021	0.78865042	0.86687155	0.69123832
Deaths	Chongqing	Both	All ages	Tracheal, bronchus, and lung cancer	Occupational exposure to chromium	Percent	2021	0.00144934	0.00169129	0.00121304
Deaths	Inner Mongolia	Both	All ages	Tracheal, bronchus, and lung cancer	Diet low in fruits	Percent	2021	0.02079788	0.03182004	0.01078711
Deaths	Shaanxi	Both	All ages	Larynx cancer	Occupational exposure to sulfuric acid	Percent	2021	0.0395604	0.07207217	0.0163665
Deaths	Shaanxi	Both	All ages	Larynx cancer	All risk factors	Percent	2021	0.78626355	0.86865125	0.68861682
Deaths	Macao Special Administrative Region of China	Both	All ages	Larynx cancer	Tobacco	Percent	2021	0.79166815	0.8736045	0.68016653
Deaths	Chongqing	Both	All ages	Tracheal, bronchus, and lung cancer	Occupational exposure to diesel engine exhaust	Percent	2021	0.01538851	0.01803769	0.01318355
Deaths	Chongqing	Both	All ages	Tracheal, bronchus, and lung cancer	Occupational exposure to nickel	Percent	2021	0.00669673	0.01501534	0.00140972

Deaths	Chongqing	Both	All ages	Tracheal, bronchus, and lung cancer	Occupational exposure to polycyclic aromatic hydrocarbons	Percent	2021	0.0049104 6	0.0058875	0.0039925 7
Deaths	Chongqing	Both	All ages	Tracheal, bronchus, and lung cancer	Occupational exposure to silica	Percent	2021	0.0310522 4	0.0484397 7	0.0138901 5
Deaths	Chongqing	Both	All ages	Tracheal, bronchus, and lung cancer	All risk factors	Percent	2021	0.7749373 6	0.9006962	0.5563422 7
Deaths	Tibet	Both	All ages	Larynx cancer	Tobacco	Percent	2021	0.6894068 1	0.8190014 7	0.5200954 7
Deaths	China	Both	All ages	Tracheal, bronchus, and lung cancer	Smoking	Percent	2021	0.6157800 1	0.8295488	0.3000372 9
Deaths	China	Both	All ages	Tracheal, bronchus, and lung cancer	Secondhand smoke	Percent	2021	0.0737825 8	0.2470049 5	-0.1030242
Deaths	China	Both	All ages	Tracheal, bronchus, and lung cancer	Metabolic risks	Percent	2021	0.0213390 6	0.0473373 1	-0.0043 01
Deaths	China	Both	All ages	Tracheal, bronchus, and lung cancer	High fasting plasma glucose	Percent	2021	0.0213390 6	0.0473373 1	-0.0043 01
Deaths	China	Both	All ages	Tracheal, bronchus, and lung cancer	Dietary risks	Percent	2021	0.0234862 3	0.0351765	0.0124842 1
Deaths	China	Both	All ages	Tracheal, bronchus, and lung cancer	Diet low in fruits	Percent	2021	0.0234862 3	0.0351765	0.0124842 1
Deaths	Hainan	Both	All ages	Larynx cancer	Occupational exposure to sulfuric acid	Percent	2021	0.0398895 8	0.0738893 3	0.0169752 9

Deaths	Hainan	Both	All ages	Larynx cancer	All risk factors	Percent	2021	0.78863738	0.88358474	0.68563717
Deaths	China	Both	All ages	Tracheal, bronchus, and lung cancer	Occupational risks	Percent	2021	0.09125319	0.1184901	0.0688322
Deaths	Hainan	Both	All ages	Tracheal, bronchus, and lung cancer	Occupational risks	Percent	2021	0.07580031	0.10338235	0.05372352
Deaths	Hainan	Both	All ages	Tracheal, bronchus, and lung cancer	Occupational carcinogens	Percent	2021	0.07580031	0.10338235	0.05372352
Deaths	Hainan	Both	All ages	Tracheal, bronchus, and lung cancer	Occupational exposure to asbestos	Percent	2021	0.01427719	0.02276551	0.00773848
Deaths	Hainan	Both	All ages	Tracheal, bronchus, and lung cancer	Occupational exposure to arsenic	Percent	2021	0.00658751	0.01091069	0.00253288
Deaths	Hainan	Both	All ages	Tracheal, bronchus, and lung cancer	Occupational exposure to beryllium	Percent	2021	0.00022468	0.00026902	0.00018662
Deaths	Hainan	Both	All ages	Tracheal, bronchus, and lung cancer	Occupational exposure to cadmium	Percent	2021	0.00063942	0.00078325	0.00050215
Deaths	Hainan	Both	All ages	Tracheal, bronchus, and lung cancer	Tobacco	Percent	2021	0.61425131	0.82281255	0.26504255
Deaths	Hainan	Both	All ages	Tracheal, bronchus, and lung cancer	Smoking	Percent	2021	0.56850731	0.7936453	0.2707655
Deaths	Hainan	Both	All ages	Tracheal, bronchus, and lung cancer	Secondhand smoke	Percent	2021	0.09050898	0.29408099	-0.128312

Deaths	Hainan	Both	All ages	Tracheal, bronchus, and lung cancer	Metabolic risks	Percent	2021	0.02547436	0.05660011	-0.0051383
Deaths	Hainan	Both	All ages	Tracheal, bronchus, and lung cancer	High fasting plasma glucose	Percent	2021	0.02547436	0.05660011	-0.0051383
Deaths	Hainan	Both	All ages	Tracheal, bronchus, and lung cancer	Dietary risks	Percent	2021	0.02572799	0.03969403	0.01321933
Deaths	Chongqing	Both	All ages	Tracheal, bronchus, and lung cancer	Environmental/occupational risks	Percent	2021	0.32653043	0.45675275	0.2324431
Deaths	Shaanxi	Both	All ages	Larynx cancer	Environmental/occupational risks	Percent	2021	0.05267644	0.08513987	0.02937669
Deaths	Shaanxi	Both	All ages	Larynx cancer	Behavioral risks	Percent	2021	0.77388261	0.86063632	0.6755798
Deaths	Shaanxi	Both	All ages	Tracheal, bronchus, and lung cancer	Occupational exposure to chromium	Percent	2021	0.00133662	0.00158496	0.00114169
Deaths	Shaanxi	Both	All ages	Tracheal, bronchus, and lung cancer	Occupational exposure to diesel engine exhaust	Percent	2021	0.01448445	0.01693712	0.01230677
Deaths	Shaanxi	Both	All ages	Tracheal, bronchus, and lung cancer	Occupational exposure to nickel	Percent	2021	0.00619798	0.01378581	0.00136233
Deaths	Shaanxi	Both	All ages	Tracheal, bronchus, and lung cancer	Occupational exposure to polycyclic aromatic hydrocarbons	Percent	2021	0.00454338	0.00540025	0.00370613
Deaths	Shaanxi	Both	All ages	Tracheal, bronchus, and lung cancer	Occupational exposure to silica	Percent	2021	0.02906403	0.04569289	0.01346557

Deaths	Shaanxi	Both	All ages	Tracheal, bronchus, and lung cancer	All risk factors	Percent	2021	0.79250927	0.91385869	0.58466511
Deaths	Tibet	Both	All ages	Larynx cancer	Environmental/occupational risks	Percent	2021	0.06395148	0.10583828	0.03441865
Deaths	Tibet	Both	All ages	Larynx cancer	Behavioral risks	Percent	2021	0.71547937	0.83613459	0.54646232
Deaths	Tibet	Both	All ages	Tracheal, bronchus, and lung cancer	Occupational exposure to chromium	Percent	2021	0.00139359	0.00162076	0.00118205
Deaths	Tibet	Both	All ages	Larynx cancer	Smoking	Percent	2021	0.68940681	0.81900147	0.52009547
Deaths	Tibet	Both	All ages	Larynx cancer	High alcohol use	Percent	2021	0.09761998	0.16891744	0.03359953
Deaths	Tibet	Both	All ages	Larynx cancer	Occupational risks	Percent	2021	0.06395148	0.10583828	0.03441865
Deaths	Tibet	Both	All ages	Larynx cancer	Occupational carcinogens	Percent	2021	0.06395148	0.10583828	0.03441865
Deaths	Tibet	Both	All ages	Larynx cancer	Occupational exposure to asbestos	Percent	2021	0.01762653	0.03024302	0.00870833
Deaths	Hainan	Both	All ages	Tracheal, bronchus, and lung cancer	Diet low in fruits	Percent	2021	0.02572799	0.03969403	0.01321933
Deaths	Tibet	Both	All ages	Tracheal, bronchus, and lung cancer	Occupational exposure to diesel engine exhaust	Percent	2021	0.01309929	0.01495861	0.01128128

Deaths	Tibet	Both	All ages	Tracheal, bronchus, and lung cancer	Occupational exposure to nickel	Percent	2021	0.00741095	0.01594526	0.001555
Deaths	Tibet	Both	All ages	Tracheal, bronchus, and lung cancer	Occupational exposure to polycyclic aromatic hydrocarbons	Percent	2021	0.00479031	0.00575023	0.00390031
Deaths	Tibet	Both	All ages	Tracheal, bronchus, and lung cancer	Occupational exposure to silica	Percent	2021	0.03468209	0.05378084	0.01562522
Deaths	Tibet	Both	All ages	Tracheal, bronchus, and lung cancer	All risk factors	Percent	2021	0.72800872	0.8686206	0.52163878
Deaths	Shaanxi	Both	All ages	Tracheal, bronchus, and lung cancer	Environmental/occupational risks	Percent	2021	0.38163613	0.49994544	0.27571051
Deaths	Hainan	Both	All ages	Tracheal, bronchus, and lung cancer	Occupational exposure to chromium	Percent	2021	0.00136541	0.00159065	0.00113995
Deaths	Hainan	Both	All ages	Larynx cancer	Environmental/occupational risks	Percent	2021	0.04442569	0.07876144	0.02128658
Deaths	Hainan	Both	All ages	Larynx cancer	Behavioral risks	Percent	2021	0.77874741	0.87877644	0.66946041
Deaths	Beijing	Both	All ages	Tracheal, bronchus, and lung cancer	Particulate matter pollution	Percent	2021	0.28191943	0.37160139	0.18837178
Deaths	Tibet	Both	All ages	Tracheal, bronchus, and lung cancer	Occupational risks	Percent	2021	0.11386136	0.14907997	0.08277752
Deaths	Tibet	Both	All ages	Tracheal, bronchus, and lung cancer	Occupational carcinogens	Percent	2021	0.11386136	0.14907997	0.08277752

Deaths	Tibet	Both	All ages	Tracheal, bronchus, and lung cancer	Occupational exposure to asbestos	Percent	2021	0.04895854	0.07617042	0.02922494
Deaths	Tibet	Both	All ages	Tracheal, bronchus, and lung cancer	Occupational exposure to arsenic	Percent	2021	0.00734539	0.01194477	0.00262514
Deaths	Tibet	Both	All ages	Tracheal, bronchus, and lung cancer	Occupational exposure to beryllium	Percent	2021	0.00028706	0.00034125	0.00023831
Deaths	Tibet	Both	All ages	Tracheal, bronchus, and lung cancer	Occupational exposure to cadmium	Percent	2021	0.0007133	0.00086359	0.00056786
Deaths	Tibet	Both	All ages	Tracheal, bronchus, and lung cancer	Environmental/occupational risks	Percent	2021	0.40073016	0.54257381	0.26648258
Deaths	Macao Special Administrative Region of China	Both	All ages	Larynx cancer	Smoking	Percent	2021	0.79166815	0.8736045	0.68016653
Deaths	Macao Special Administrative Region of China	Both	All ages	Larynx cancer	High alcohol use	Percent	2021	0.19836128	0.28579047	0.09609158
Deaths	Macao Special Administrative Region of China	Both	All ages	Larynx cancer	Occupational risks	Percent	2021	0.05137727	0.08907425	0.0268109
Deaths	Macao Special Administrative Region of China	Both	All ages	Larynx cancer	Occupational carcinogens	Percent	2021	0.05137727	0.08907425	0.0268109
Deaths	Macao Special Administrative Region of China	Both	All ages	Larynx cancer	Occupational exposure to asbestos	Percent	2021	0.00779314	0.01399455	0.00336076
Deaths	Hainan	Both	All ages	Tracheal, bronchus, and lung cancer	Occupational exposure to diesel engine exhaust	Percent	2021	0.01462264	0.01730344	0.01236761

Deaths	Hainan	Both	All ages	Tracheal, bronchus, and lung cancer	Occupational exposure to nickel	Percent	2021	0.0063392	0.0138783	0.00130918
Deaths	Hainan	Both	All ages	Tracheal, bronchus, and lung cancer	Occupational exposure to polycyclic aromatic hydrocarbons	Percent	2021	0.00462473	0.00560389	0.00380826
Deaths	Hainan	Both	All ages	Tracheal, bronchus, and lung cancer	Occupational exposure to silica	Percent	2021	0.02968615	0.04627091	0.01319112
Deaths	Hainan	Both	All ages	Tracheal, bronchus, and lung cancer	All risk factors	Percent	2021	0.72544692	0.87579481	0.46433044
Deaths	Hainan	Both	All ages	Tracheal, bronchus, and lung cancer	Environmental/occupational risks	Percent	2021	0.246368	0.33825812	0.16462105
Deaths	Inner Mongolia	Both	All ages	Tracheal, bronchus, and lung cancer	Air pollution	Percent	2021	0.21043705	0.30797537	0.13258322
Deaths	Inner Mongolia	Both	All ages	Tracheal, bronchus, and lung cancer	Ambient particulate matter pollution	Percent	2021	0.18611621	0.25966061	0.10324023
Deaths	Inner Mongolia	Both	All ages	Tracheal, bronchus, and lung cancer	Household air pollution from solid fuels	Percent	2021	0.02419605	0.15951726	0.00028262
Deaths	Inner Mongolia	Both	All ages	Tracheal, bronchus, and lung cancer	Other environmental risks	Percent	2021	0.05335747	0.21564073	-0.0255825
Deaths	China	Both	All ages	Tracheal, bronchus, and lung cancer	Air pollution	Percent	2021	0.25983838	0.35000163	0.16943391
Deaths	China	Both	All ages	Tracheal, bronchus, and lung cancer	Ambient particulate matter pollution	Percent	2021	0.21966373	0.30524426	0.13504009

Deaths	China	Both	All ages	Tracheal, bronchus, and lung cancer	Household air pollution from solid fuels	Percent	2021	0.04006103	0.13697133	0.00560531
Deaths	China	Both	All ages	Tracheal, bronchus, and lung cancer	Other environmental risks	Percent	2021	0.03682672	0.09988872	-0.0179148
Deaths	China	Both	All ages	Tracheal, bronchus, and lung cancer	Occupational carcinogens	Percent	2021	0.09125319	0.1184901	0.0688322
Deaths	China	Both	All ages	Tracheal, bronchus, and lung cancer	Occupational exposure to asbestos	Percent	2021	0.03192851	0.04526533	0.02042233
Deaths	China	Both	All ages	Tracheal, bronchus, and lung cancer	Occupational exposure to arsenic	Percent	2021	0.00644827	0.0103724	0.00246115
Deaths	China	Both	All ages	Tracheal, bronchus, and lung cancer	Occupational exposure to beryllium	Percent	2021	0.00022505	0.00026508	0.0001881
Deaths	China	Both	All ages	Tracheal, bronchus, and lung cancer	Occupational exposure to cadmium	Percent	2021	0.0006277	0.00074296	0.00051376
Deaths	Tibet	Both	All ages	Tracheal, bronchus, and lung cancer	Air pollution	Percent	2021	0.28802009	0.44024992	0.15339124
Deaths	Tibet	Both	All ages	Tracheal, bronchus, and lung cancer	Ambient particulate matter pollution	Percent	2021	0.07859313	0.12700287	0.02862467
Deaths	Tibet	Both	All ages	Tracheal, bronchus, and lung cancer	Household air pollution from solid fuels	Percent	2021	0.20933877	0.38747578	0.0559794
Deaths	Tibet	Both	All ages	Tracheal, bronchus, and lung cancer	Other environmental risks	Percent	2021	0.0500291	0.19329093	-0.0196556

Deaths	Shaanxi	Both	All ages	Larynx cancer	Tobacco	Percent	2021	0.75697284	0.84837851	0.64679249
Deaths	Macao Special Administrative Region of China	Both	All ages	Tracheal, bronchus, and lung cancer	Tobacco	Percent	2021	0.61148721	0.83909313	0.26838915
Deaths	Macao Special Administrative Region of China	Both	All ages	Tracheal, bronchus, and lung cancer	Smoking	Percent	2021	0.57534933	0.81376732	0.26716642
Deaths	Macao Special Administrative Region of China	Both	All ages	Tracheal, bronchus, and lung cancer	Secondhand smoke	Percent	2021	0.07681123	0.26278227	-0.1062539
Deaths	Macao Special Administrative Region of China	Both	All ages	Tracheal, bronchus, and lung cancer	Metabolic risks	Percent	2021	0.01914578	0.04295228	-0.0038218
Deaths	Macao Special Administrative Region of China	Both	All ages	Tracheal, bronchus, and lung cancer	High fasting plasma glucose	Percent	2021	0.01914578	0.04295228	-0.0038218
Deaths	Macao Special Administrative Region of China	Both	All ages	Tracheal, bronchus, and lung cancer	Dietary risks	Percent	2021	0.05206163	0.08022323	0.02605051
Deaths	Shandong	Both	All ages	Larynx cancer	Occupational exposure to sulfuric acid	Percent	2021	0.0432786	0.07732657	0.0181093
Deaths	Shandong	Both	All ages	Larynx cancer	All risk factors	Percent	2021	0.80892879	0.89012935	0.70467616
Deaths	Chongqing	Both	All ages	Larynx cancer	Tobacco	Percent	2021	0.77039632	0.85624731	0.66512836
Deaths	Chongqing	Both	All ages	Tracheal, bronchus, and lung cancer	Tobacco	Percent	2021	0.64872785	0.83838546	0.31961759

Deaths	Chongqing	Both	All ages	Tracheal, bronchus, and lung cancer	Smoking	Percent	2021	0.61244097	0.81295661	0.32362882
Deaths	Chongqing	Both	All ages	Tracheal, bronchus, and lung cancer	Secondhand smoke	Percent	2021	0.07706629	0.25863165	-0.1089821
Deaths	Chongqing	Both	All ages	Tracheal, bronchus, and lung cancer	Metabolic risks	Percent	2021	0.0214101	0.04722165	-0.0042073
Deaths	Chongqing	Both	All ages	Tracheal, bronchus, and lung cancer	High fasting plasma glucose	Percent	2021	0.0214101	0.04722165	-0.0042073
Deaths	Chongqing	Both	All ages	Tracheal, bronchus, and lung cancer	Dietary risks	Percent	2021	0.02182941	0.03377861	0.01121806
Deaths	Macao Special Administrative Region of China	Both	All ages	Tracheal, bronchus, and lung cancer	Air pollution	Percent	2021	0.16444506	0.22884715	0.10427558
Deaths	Macao Special Administrative Region of China	Both	All ages	Tracheal, bronchus, and lung cancer	Ambient particulate matter pollution	Percent	2021	0.1641022	0.22804494	0.10329853
Deaths	Macao Special Administrative Region of China	Both	All ages	Tracheal, bronchus, and lung cancer	Household air pollution from solid fuels	Percent	2021	0.00030398	1.55E-05	0
Deaths	Macao Special Administrative Region of China	Both	All ages	Tracheal, bronchus, and lung cancer	Other environmental risks	Percent	2021	0.0286682	0.12702338	-0.0113053
Deaths	Chongqing	Both	All ages	Larynx cancer	Smoking	Percent	2021	0.77039632	0.85624731	0.66512836
Deaths	Chongqing	Both	All ages	Larynx cancer	High alcohol use	Percent	2021	0.13830852	0.20019802	0.07256221

Deaths	Chongqing	Both	All ages	Larynx cancer	Occupational risks	Percent	2021	0.04629317	0.08107849	0.02313472
Deaths	Chongqing	Both	All ages	Larynx cancer	Occupational carcinogens	Percent	2021	0.04629317	0.08107849	0.02313472
Deaths	Chongqing	Both	All ages	Larynx cancer	Occupational exposure to asbestos	Percent	2021	0.00569351	0.00968727	0.00293309
Deaths	Chongqing	Both	All ages	Tracheal, bronchus, and lung cancer	Occupational risks	Percent	2021	0.08116657	0.10983263	0.056935
Deaths	Chongqing	Both	All ages	Tracheal, bronchus, and lung cancer	Occupational carcinogens	Percent	2021	0.08116657	0.10983263	0.056935
Deaths	Chongqing	Both	All ages	Tracheal, bronchus, and lung cancer	Occupational exposure to asbestos	Percent	2021	0.01667856	0.0266295	0.00982966
Deaths	Chongqing	Both	All ages	Tracheal, bronchus, and lung cancer	Occupational exposure to arsenic	Percent	2021	0.0070088	0.01145668	0.00260512
Deaths	Chongqing	Both	All ages	Tracheal, bronchus, and lung cancer	Occupational exposure to beryllium	Percent	2021	0.00024326	0.00029165	0.00019449
Deaths	Chongqing	Both	All ages	Tracheal, bronchus, and lung cancer	Occupational exposure to cadmium	Percent	2021	0.00068223	0.00083852	0.00054775
Deaths	Hainan	Both	All ages	Tracheal, bronchus, and lung cancer	Air pollution	Percent	2021	0.16247599	0.26298047	0.08919787
Deaths	Hainan	Both	All ages	Tracheal, bronchus, and lung cancer	Ambient particulate matter pollution	Percent	2021	0.10249209	0.15736011	0.05052155

Deaths	Hainan	Both	All ages	Tracheal, bronchus, and lung cancer	Household air pollution from solid fuels	Percent	2021	0.05991664	0.18345358	0.00624736
Deaths	Hainan	Both	All ages	Tracheal, bronchus, and lung cancer	Other environmental risks	Percent	2021	0.02642005	0.10408015	-0.0104885
Deaths	Shaanxi	Both	All ages	Larynx cancer	Smoking	Percent	2021	0.75697284	0.84837851	0.64679249
Deaths	Shaanxi	Both	All ages	Larynx cancer	High alcohol use	Percent	2021	0.12415914	0.18480865	0.06028824
Deaths	Shaanxi	Both	All ages	Larynx cancer	Occupational risks	Percent	2021	0.05267644	0.08513987	0.02937669
Deaths	Shaanxi	Both	All ages	Larynx cancer	Occupational carcinogens	Percent	2021	0.05267644	0.08513987	0.02937669
Deaths	Shaanxi	Both	All ages	Larynx cancer	Occupational exposure to asbestos	Percent	2021	0.01344497	0.02147629	0.00723962
Deaths	Chongqing	Both	All ages	Tracheal, bronchus, and lung cancer	Air pollution	Percent	2021	0.24215175	0.32967576	0.15743363
Deaths	Chongqing	Both	All ages	Tracheal, bronchus, and lung cancer	Ambient particulate matter pollution	Percent	2021	0.21828876	0.30251964	0.12838961
Deaths	Chongqing	Both	All ages	Tracheal, bronchus, and lung cancer	Household air pollution from solid fuels	Percent	2021	0.02374089	0.13091166	0.00060335
Deaths	Chongqing	Both	All ages	Tracheal, bronchus, and lung cancer	Other environmental risks	Percent	2021	0.03372665	0.1585107	-0.013737

Deaths	Hebei	Both	All ages	Larynx cancer	Occupational exposure to sulfuric acid	Percent	2021	0.04346569	0.0783972	0.01788988
Deaths	Hebei	Both	All ages	Larynx cancer	All risk factors	Percent	2021	0.75501007	0.84699844	0.64396499
Deaths	Macao Special Administrative Region of China	Both	All ages	Tracheal, bronchus, and lung cancer	Occupational risks	Percent	2021	0.08570065	0.11161598	0.06398137
Deaths	Macao Special Administrative Region of China	Both	All ages	Tracheal, bronchus, and lung cancer	Occupational carcinogens	Percent	2021	0.08570065	0.11161598	0.06398137
Deaths	Macao Special Administrative Region of China	Both	All ages	Tracheal, bronchus, and lung cancer	Occupational exposure to asbestos	Percent	2021	0.02342407	0.03704304	0.01123229
Deaths	Macao Special Administrative Region of China	Both	All ages	Tracheal, bronchus, and lung cancer	Occupational exposure to arsenic	Percent	2021	0.00635357	0.01031588	0.00253673
Deaths	Macao Special Administrative Region of China	Both	All ages	Tracheal, bronchus, and lung cancer	Occupational exposure to beryllium	Percent	2021	0.00022845	0.00027662	0.00018416
Deaths	Macao Special Administrative Region of China	Both	All ages	Tracheal, bronchus, and lung cancer	Occupational exposure to cadmium	Percent	2021	0.00062909	0.00078235	0.00048252
Deaths	China	Both	All ages	Tracheal, bronchus, and lung cancer	Occupational exposure to chromium	Percent	2021	0.00133295	0.00151154	0.00117555
Deaths	Shaanxi	Both	All ages	Tracheal, bronchus, and lung cancer	Occupational risks	Percent	2021	0.09878881	0.13052576	0.07199127
Deaths	Shaanxi	Both	All ages	Tracheal, bronchus, and lung cancer	Occupational carcinogens	Percent	2021	0.09878881	0.13052576	0.07199127

Deaths	Shaanxi	Both	All ages	Tracheal, bronchus, and lung cancer	Occupational exposure to asbestos	Percent	2021	0.03925707	0.05998401	0.02350449
Deaths	Shaanxi	Both	All ages	Tracheal, bronchus, and lung cancer	Occupational exposure to arsenic	Percent	2021	0.00646722	0.0106016	0.00235527
Deaths	Shaanxi	Both	All ages	Tracheal, bronchus, and lung cancer	Occupational exposure to beryllium	Percent	2021	0.00022766	0.0002716	0.00018927
Deaths	Shaanxi	Both	All ages	Tracheal, bronchus, and lung cancer	Occupational exposure to cadmium	Percent	2021	0.00063051	0.00076718	0.00049896
Deaths	Macao Special Administrative Region of China	Both	All ages	Tracheal, bronchus, and lung cancer	Diet low in fruits	Percent	2021	0.05206163	0.08022323	0.02605051
Deaths	Inner Mongolia	Both	All ages	Tracheal, bronchus, and lung cancer	Residential radon	Percent	2021	0.05335747	0.21564073	-0.0255825
Deaths	Inner Mongolia	Both	All ages	Tracheal, bronchus, and lung cancer	Behavioral risks	Percent	2021	0.70993501	0.91151844	0.34167722
Deaths	Jiangsu	Both	All ages	Larynx cancer	Occupational exposure to sulfuric acid	Percent	2021	0.036284	0.06673387	0.0149367
Deaths	Jiangsu	Both	All ages	Larynx cancer	All risk factors	Percent	2021	0.8120116	0.89647719	0.70379765
Deaths	Fujian	Both	All ages	Larynx cancer	Tobacco	Percent	2021	0.77032914	0.85237591	0.65647527
Deaths	Chongqing	Both	All ages	Tracheal, bronchus, and lung cancer	Diet low in fruits	Percent	2021	0.02182941	0.03377861	0.01121806

Deaths	Shaanxi	Both	All ages	Tracheal, bronchus, and lung cancer	Air pollution	Percent	2021	0.28382196	0.380167	0.18504036
Deaths	Shaanxi	Both	All ages	Tracheal, bronchus, and lung cancer	Ambient particulate matter pollution	Percent	2021	0.24504507	0.34220882	0.14053604
Deaths	Shaanxi	Both	All ages	Tracheal, bronchus, and lung cancer	Household air pollution from solid fuels	Percent	2021	0.03863042	0.1646438	0.00255817
Deaths	Shaanxi	Both	All ages	Tracheal, bronchus, and lung cancer	Other environmental risks	Percent	2021	0.04201786	0.16310234	-0.0142446
Deaths	Tibet	Both	All ages	Tracheal, bronchus, and lung cancer	Residential radon	Percent	2021	0.0500291	0.19329093	-0.0196556
Deaths	Tibet	Both	All ages	Tracheal, bronchus, and lung cancer	Behavioral risks	Percent	2021	0.53712837	0.75802063	0.20906912
Deaths	Shandong	Both	All ages	Larynx cancer	Environmental/occupational risks	Percent	2021	0.05121539	0.08656221	0.02616996
Deaths	Shandong	Both	All ages	Larynx cancer	Behavioral risks	Percent	2021	0.79825602	0.88262388	0.68816507
Deaths	Hebei	Both	All ages	Larynx cancer	Environmental/occupational risks	Percent	2021	0.06374527	0.10134062	0.03624711
Deaths	Hebei	Both	All ages	Larynx cancer	Behavioral risks	Percent	2021	0.73814942	0.83698077	0.62319441
Deaths	Hebei	Both	All ages	Tracheal, bronchus, and lung cancer	Occupational exposure to chromium	Percent	2021	0.01458611	0.01678774	0.01246944

Deaths	Fujian	Both	All ages	Larynx cancer	Occupational exposure to sulfuric acid	Percent	2021	0.04047303	0.07444227	0.01641811
Deaths	Fujian	Both	All ages	Larynx cancer	All risk factors	Percent	2021	0.80072839	0.87316322	0.69877168
Deaths	Jiangsu	Both	All ages	Larynx cancer	Environmental/occupational risks	Percent	2021	0.04227045	0.07434537	0.0216147
Deaths	Jiangsu	Both	All ages	Larynx cancer	Behavioral risks	Percent	2021	0.80335729	0.89270411	0.68968671
Deaths	Jiangsu	Both	All ages	Tracheal, bronchus, and lung cancer	Occupational exposure to chromium	Percent	2021	0.01287022	0.01505944	0.01076822
Deaths	China	Both	All ages	Tracheal, bronchus, and lung cancer	Residential radon	Percent	2021	0.03682672	0.09988872	-0.0179148
Deaths	China	Both	All ages	Tracheal, bronchus, and lung cancer	Behavioral risks	Percent	2021	0.65628296	0.85163633	0.31040779
Deaths	Shaanxi	Both	All ages	Tracheal, bronchus, and lung cancer	Tobacco	Percent	2021	0.65015148	0.85200955	0.31476037
Deaths	Shaanxi	Both	All ages	Tracheal, bronchus, and lung cancer	Smoking	Percent	2021	0.61472048	0.82671024	0.3171509
Deaths	Shaanxi	Both	All ages	Tracheal, bronchus, and lung cancer	Secondhand smoke	Percent	2021	0.07485118	0.25497218	-0.1158278
Deaths	Shaanxi	Both	All ages	Tracheal, bronchus, and lung cancer	Metabolic risks	Percent	2021	0.01780808	0.03918167	-0.0035422

Deaths	Shaanxi	Both	All ages	Tracheal, bronchus, and lung cancer	High fasting plasma glucose	Percent	2021	0.01780808	0.03918167	-0.0035422
Deaths	Shaanxi	Both	All ages	Tracheal, bronchus, and lung cancer	Dietary risks	Percent	2021	0.02382543	0.03659458	0.01238823
Deaths	Taiwan (Province of China)	Both	All ages	Larynx cancer	Occupational exposure to sulfuric acid	Percent	2021	0.04033692	0.07234821	0.01696181
Deaths	Taiwan (Province of China)	Both	All ages	Larynx cancer	All risk factors	Percent	2021	0.80123933	0.87705065	0.70099887
Deaths	Hainan	Both	All ages	Tracheal, bronchus, and lung cancer	Residential radon	Percent	2021	0.02642005	0.10408015	-0.0104885
Deaths	Hainan	Both	All ages	Tracheal, bronchus, and lung cancer	Behavioral risks	Percent	2021	0.62414459	0.82801828	0.28117937
Deaths	Tibet	Both	All ages	Tracheal, bronchus, and lung cancer	Particulate matter pollution	Percent	2021	0.28802009	0.44024992	0.15339124
Deaths	Shaanxi	Both	All ages	Tracheal, bronchus, and lung cancer	Residential radon	Percent	2021	0.04201786	0.16310234	-0.0142446
Deaths	Shaanxi	Both	All ages	Tracheal, bronchus, and lung cancer	Behavioral risks	Percent	2021	0.65841742	0.85595948	0.32862696
Deaths	Hebei	Both	All ages	Tracheal, bronchus, and lung cancer	Occupational exposure to diesel engine exhaust	Percent	2021	0.0154868	0.01794549	0.01330329
Deaths	Hebei	Both	All ages	Tracheal, bronchus, and lung cancer	Occupational exposure to nickel	Percent	2021	0.00676038	0.01506512	0.00144193

**Deaths**	**Hebei**	**Both**	**All ages**	**Tracheal, bronchus, and lung cancer**	**Occupational exposure to polycyclic aromatic hydrocarbons**	**Percent**	**2021**	**0.0049426**	**0.00597049**	**0.00410994**
**Deaths**	**Hebei**	**Both**	**All ages**	**Tracheal, bronchus, and lung cancer**	**Occupational exposure to silica**	**Percent**	**2021**	**0.03114622**	**0.04809919**	**0.01466642**
**Deaths**	**Hebei**	**Both**	**All ages**	**Tracheal, bronchus, and lung cancer**	**All risk factors**	**Percent**	**2021**	**0.76891615**	**0.89772335**	**0.57311216**
**Deaths**	**Jiangsu**	**Both**	**All ages**	**Tracheal, bronchus, and lung cancer**	**Occupational exposure to diesel engine exhaust**	**Percent**	**2021**	**0.01405296**	**0.0163955**	**0.0119703**
**Deaths**	**Jiangsu**	**Both**	**All ages**	**Tracheal, bronchus, and lung cancer**	**Occupational exposure to nickel**	**Percent**	**2021**	**0.00581024**	**0.01316556**	**0.00127071**
**Deaths**	**Jiangsu**	**Both**	**All ages**	**Tracheal, bronchus, and lung cancer**	**Occupational exposure to polycyclic aromatic hydrocarbons**	**Percent**	**2021**	**0.00436331**	**0.00517118**	**0.00358748**
**Deaths**	**Jiangsu**	**Both**	**All ages**	**Tracheal, bronchus, and lung cancer**	**Occupational exposure to silica**	**Percent**	**2021**	**0.02707422**	**0.04276417**	**0.01294149**
**Deaths**	**Jiangsu**	**Both**	**All ages**	**Tracheal, bronchus, and lung cancer**	**All risk factors**	**Percent**	**2021**	**0.78997733**	**0.9310957**	**0.56361571**
**Deaths**	**Jiangsu**	**Both**	**All ages**	**Tracheal, bronchus, and lung cancer**	**Environmental/occupational risks**	**Percent**	**2021**	**0.34422694**	**0.44724943**	**0.25170819**
**Deaths**	**Jiangsu**	**Both**	**All ages**	**Larynx cancer**	**Tobacco**	**Percent**	**2021**	**0.77964101**	**0.87766889**	**0.65229993**
**Deaths**	**Fujian**	**Both**	**All ages**	**Larynx cancer**	**Environmental/occupational risks**	**Percent**	**2021**	**0.04423316**	**0.07842634**	**0.02044081**

Deaths	Fujian	Both	All ages	Larynx cancer	Behavioral risks	Percent	2021	0.7919747	0.86607746	0.6864019
Deaths	Fujian	Both	All ages	Tracheal, bronchus, and lung cancer	Occupational exposure to chromium	Percent	2021	0.001387	0.00162371	0.00116935
Deaths	Shandong	Both	All ages	Tracheal, bronchus, and lung cancer	Occupational exposure to diesel engine exhaust	Percent	2021	0.01555537	0.01799607	0.01324812
Deaths	Shandong	Both	All ages	Tracheal, bronchus, and lung cancer	Occupational exposure to nickel	Percent	2021	0.00666313	0.0153438	0.00142311
Deaths	Shandong	Both	All ages	Tracheal, bronchus, and lung cancer	Occupational exposure to polycyclic aromatic hydrocarbons	Percent	2021	0.00491296	0.0058883	0.00405374
Deaths	Shandong	Both	All ages	Tracheal, bronchus, and lung cancer	Occupational exposure to silica	Percent	2021	0.03095428	0.050401	0.01413155
Deaths	Shandong	Both	All ages	Tracheal, bronchus, and lung cancer	All risk factors	Percent	2021	0.78536533	0.91001824	0.56648299
Deaths	Hebei	Both	All ages	Tracheal, bronchus, and lung cancer	Environmental/occupational risks	Percent	2021	0.41043466	0.52629534	0.30197326
Deaths	Taiwan (Province of China)	Both	All ages	Larynx cancer	Environmental/occupational risks	Percent	2021	0.05444189	0.08865395	0.02920448
Deaths	Taiwan (Province of China)	Both	All ages	Larynx cancer	Behavioral risks	Percent	2021	0.79068793	0.87036986	0.68668305
Deaths	Taiwan (Province of China)	Both	All ages	Tracheal, bronchus, and lung cancer	Occupational exposure to chromium	Percent	2021	0.00122642	0.0013737	0.00109278

Deaths	Tibet	Both	All ages	Tracheal, bronchus, and lung cancer	Tobacco	Percent	2021	0.52386011	0.75222977	0.18296254
Deaths	Tibet	Both	All ages	Tracheal, bronchus, and lung cancer	Smoking	Percent	2021	0.48751126	0.72144827	0.18930619
Deaths	Tibet	Both	All ages	Tracheal, bronchus, and lung cancer	Secondhand smoke	Percent	2021	0.07274595	0.25927786	-0.0961643
Deaths	Tibet	Both	All ages	Tracheal, bronchus, and lung cancer	Metabolic risks	Percent	2021	0.01394987	0.03123138	-0.0028023
Deaths	Tibet	Both	All ages	Tracheal, bronchus, and lung cancer	High fasting plasma glucose	Percent	2021	0.01394987	0.03123138	-0.0028023
Deaths	Tibet	Both	All ages	Tracheal, bronchus, and lung cancer	Dietary risks	Percent	2021	0.02790875	0.04314539	0.01426645
Deaths	Jiangsu	Both	All ages	Tracheal, bronchus, and lung cancer	Tobacco	Percent	2021	0.66496069	0.88476953	0.29930521
Deaths	Jiangsu	Both	All ages	Tracheal, bronchus, and lung cancer	Smoking	Percent	2021	0.63734566	0.8710271	0.30073733
Deaths	Jiangsu	Both	All ages	Tracheal, bronchus, and lung cancer	Secondhand smoke	Percent	2021	0.06976685	0.248181	-0.0801869
Deaths	Jiangsu	Both	All ages	Tracheal, bronchus, and lung cancer	Metabolic risks	Percent	2021	0.02222517	0.04921495	-0.0044425
Deaths	Jiangsu	Both	All ages	Tracheal, bronchus, and lung cancer	High fasting plasma glucose	Percent	2021	0.02222517	0.04921495	-0.0044425

Deaths	Jiangsu	Both	All ages	Tracheal, bronchus, and lung cancer	Dietary risks	Percent	2021	0.01886726	0.0300031	0.00945626
Deaths	Shanghai	Both	All ages	Larynx cancer	Occupational exposure to sulfuric acid	Percent	2021	0.02677121	0.04958576	0.01067905
Deaths	Shanghai	Both	All ages	Larynx cancer	All risk factors	Percent	2021	0.81290358	0.89641359	0.71227655
Deaths	Xinjiang	Both	All ages	Tracheal, bronchus, and lung cancer	Tobacco	Percent	2021	0.51197243	0.7506506	0.17990481
Deaths	Xinjiang	Both	All ages	Tracheal, bronchus, and lung cancer	Smoking	Percent	2021	0.47023618	0.7192327	0.18023782
Deaths	Xinjiang	Both	All ages	Tracheal, bronchus, and lung cancer	Secondhand smoke	Percent	2021	0.07314948	0.25155014	-0.0953012
Deaths	Xinjiang	Both	All ages	Tracheal, bronchus, and lung cancer	Metabolic risks	Percent	2021	0.02356775	0.05292954	-0.0047277
Deaths	Xinjiang	Both	All ages	Tracheal, bronchus, and lung cancer	High fasting plasma glucose	Percent	2021	0.02356775	0.05292954	-0.0047277
Deaths	Xinjiang	Both	All ages	Tracheal, bronchus, and lung cancer	Dietary risks	Percent	2021	0.02548171	0.03800596	0.01276239
Deaths	Jiangsu	Both	All ages	Larynx cancer	Smoking	Percent	2021	0.77964101	0.87766889	0.65229993
Deaths	Jiangsu	Both	All ages	Larynx cancer	High alcohol use	Percent	2021	0.15631652	0.23482592	0.07740958

Deaths	Jiangsu	Both	All ages	Larynx cancer	Occupational risks	Percent	2021	0.04227045	0.07434537	0.0216147
Deaths	Jiangsu	Both	All ages	Larynx cancer	Occupational carcinogens	Percent	2021	0.04227045	0.07434537	0.0216147
Deaths	Jiangsu	Both	All ages	Larynx cancer	Occupational exposure to asbestos	Percent	2021	0.0609448	0.01041776	0.0031135 1
Deaths	Xinjiang	Both	All ages	Larynx cancer	Occupational exposure to sulfuric acid	Percent	2021	0.04163493	0.07734146	0.01683701
Deaths	Xinjiang	Both	All ages	Larynx cancer	All risk factors	Percent	2021	0.72466726	0.82865726	0.60244469
Deaths	Jiangsu	Both	All ages	Tracheal, bronchus, and lung cancer	Occupational risks	Percent	2021	0.07485092	0.09904961	0.05346598
Deaths	Jiangsu	Both	All ages	Tracheal, bronchus, and lung cancer	Occupational carcinogens	Percent	2021	0.07485092	0.09904961	0.05346598
Deaths	Jiangsu	Both	All ages	Tracheal, bronchus, and lung cancer	Occupational exposure to asbestos	Percent	2021	0.01772525	0.02850038	0.01027299
Deaths	Jiangsu	Both	All ages	Tracheal, bronchus, and lung cancer	Occupational exposure to arsenic	Percent	2021	0.00612904	0.01012928	0.00230858
Deaths	Jiangsu	Both	All ages	Tracheal, bronchus, and lung cancer	Occupational exposure to beryllium	Percent	2021	0.00020913	0.00025075	0.00016854
Deaths	Jiangsu	Both	All ages	Tracheal, bronchus, and lung cancer	Occupational exposure to cadmium	Percent	2021	0.00059622	0.00073351	0.00047904

Deaths	Shandong	Both	All ages	Tracheal, bronchus, and lung cancer	Environmental/occupational risks	Percent	2021	0.37037262	0.46413393	0.2707191
Deaths	Hebei	Both	All ages	Tracheal, bronchus, and lung cancer	Tobacco	Percent	2021	0.59135043	0.81759593	0.24804719
Deaths	Hebei	Both	All ages	Tracheal, bronchus, and lung cancer	Smoking	Percent	2021	0.55904253	0.79572636	0.24545693
Deaths	Hebei	Both	All ages	Tracheal, bronchus, and lung cancer	Secondhand smoke	Percent	2021	0.06716368	0.23769393	-0.0887277
Deaths	Hebei	Both	All ages	Tracheal, bronchus, and lung cancer	Metabolic risks	Percent	2021	0.0216596	0.04885188	-0.004283
Deaths	Hebei	Both	All ages	Tracheal, bronchus, and lung cancer	High fasting plasma glucose	Percent	2021	0.0216596	0.04885188	-0.004283
Deaths	Hebei	Both	All ages	Tracheal, bronchus, and lung cancer	Dietary risks	Percent	2021	0.02478799	0.03750808	0.01223833
Deaths	Xinjiang	Both	All ages	Larynx cancer	Environmental/occupational risks	Percent	2021	0.0511553	0.08701846	0.0271938
Deaths	Xinjiang	Both	All ages	Larynx cancer	Behavioral risks	Percent	2021	0.70973454	0.81991717	0.57979632
Deaths	Xinjiang	Both	All ages	Tracheal, bronchus, and lung cancer	Occupational exposure to chromium	Percent	2021	0.00135794	0.00159367	0.00115115
Deaths	Shanghai	Both	All ages	Larynx cancer	Environmental/occupational risks	Percent	2021	0.03810634	0.06233847	0.02117258

Deaths	Shanghai	Both	All ages	Larynx cancer	Behavioral risks	Percent	2021	0.80587332	0.8929477	0.70376856
Deaths	Shanghai	Both	All ages	Tracheal, bronchus, and lung cancer	Occupational exposure to chromium	Percent	2021	0.00091684	0.00106553	0.00077689
Deaths	Fujian	Both	All ages	Tracheal, bronchus, and lung cancer	Occupational exposure to diesel engine exhaust	Percent	2021	0.01500897	0.01775348	0.01272219
Deaths	Fujian	Both	All ages	Tracheal, bronchus, and lung cancer	Occupational exposure to nickel	Percent	2021	0.00643806	0.01442852	0.00135179
Deaths	Fujian	Both	All ages	Tracheal, bronchus, and lung cancer	Occupational exposure to polycyclic aromatic hydrocarbons	Percent	2021	0.00468219	0.0055566	0.00387887
Deaths	Fujian	Both	All ages	Tracheal, bronchus, and lung cancer	Occupational exposure to silica	Percent	2021	0.03029802	0.04838202	0.01383823
Deaths	Fujian	Both	All ages	Tracheal, bronchus, and lung cancer	All risk factors	Percent	2021	0.75038986	0.88598001	0.51995259
Deaths	Fujian	Both	All ages	Tracheal, bronchus, and lung cancer	Environmental/occupational risks	Percent	2021	0.26362278	0.36236513	0.18900709
Deaths	Hebei	Both	All ages	Larynx cancer	Tobacco	Percent	2021	0.71431268	0.81876992	0.58385614
Deaths	Shanghai	Both	All ages	Tracheal, bronchus, and lung cancer	Occupational exposure to diesel engine exhaust	Percent	2021	0.01079719	0.01263222	0.00900614
Deaths	Shanghai	Both	All ages	Tracheal, bronchus, and lung cancer	Occupational exposure to nickel	Percent	2021	0.00399206	0.00911747	0.00085383

Deaths	Shanghai	Both	All ages	Tracheal, bronchus, and lung cancer	Occupational exposure to polycyclic aromatic hydrocarbons	Percent	2021	0.00311619	0.00371586	0.00253018
Deaths	Shanghai	Both	All ages	Tracheal, bronchus, and lung cancer	Occupational exposure to silica	Percent	2021	0.01896327	0.03039882	0.00861283
Deaths	Shanghai	Both	All ages	Tracheal, bronchus, and lung cancer	All risk factors	Percent	2021	0.72843394	0.87076556	0.50940648
Deaths	Shanghai	Both	All ages	Tracheal, bronchus, and lung cancer	Environmental/occupational risks	Percent	2021	0.31088879	0.39843172	0.22115177
Deaths	Jiangsu	Both	All ages	Tracheal, bronchus, and lung cancer	Diet low in fruits	Percent	2021	0.01886726	0.0300031	0.00945626
Deaths	Macao Special Administrative Region of China	Both	All ages	Tracheal, bronchus, and lung cancer	Residential radon	Percent	2021	0.0286682	0.12702338	-0.0113053
Deaths	Macao Special Administrative Region of China	Both	All ages	Tracheal, bronchus, and lung cancer	Behavioral risks	Percent	2021	0.63148748	0.84704119	0.29848434
Deaths	Xinjiang	Both	All ages	Tracheal, bronchus, and lung cancer	Occupational exposure to diesel engine exhaust	Percent	2021	0.01432895	0.0166791	0.0122575
Deaths	Xinjiang	Both	All ages	Tracheal, bronchus, and lung cancer	Occupational exposure to nickel	Percent	2021	0.00636484	0.01428282	0.00133105
Deaths	Xinjiang	Both	All ages	Tracheal, bronchus, and lung cancer	Occupational exposure to polycyclic aromatic hydrocarbons	Percent	2021	0.00462529	0.00552604	0.0038314
Deaths	Xinjiang	Both	All ages	Tracheal, bronchus, and lung cancer	Occupational exposure to silica	Percent	2021	0.02942174	0.04605921	0.01369955

Deaths	Xinjiang	Both	All ages	Tracheal, bronchus, and lung cancer	All risk factors	Percent	2021	0.71498962	0.86060777	0.50926377
Deaths	Taiwan (Province of China)	Both	All ages	Tracheal, bronchus, and lung cancer	Occupational exposure to diesel engine exhaust	Percent	2021	0.01384894	0.01567633	0.01213137
Deaths	Taiwan (Province of China)	Both	All ages	Tracheal, bronchus, and lung cancer	Occupational exposure to nickel	Percent	2021	0.00546945	0.01186701	0.00112699
Deaths	Taiwan (Province of China)	Both	All ages	Tracheal, bronchus, and lung cancer	Occupational exposure to polycyclic aromatic hydrocarbons	Percent	2021	0.00424071	0.00490141	0.00364844
Deaths	Taiwan (Province of China)	Both	All ages	Tracheal, bronchus, and lung cancer	Occupational exposure to silica	Percent	2021	0.02760288	0.04292163	0.01279975
Deaths	Taiwan (Province of China)	Both	All ages	Tracheal, bronchus, and lung cancer	All risk factors	Percent	2021	0.63737838	0.78282351	0.39826022
Deaths	Shaanxi	Both	All ages	Tracheal, bronchus, and lung cancer	Diet low in fruits	Percent	2021	0.02382543	0.03659458	0.01238823
Deaths	Inner Mongolia	Both	All ages	Tracheal, bronchus, and lung cancer	Particulate matter pollution	Percent	2021	0.21043705	0.30797537	0.13258322
Deaths	Fujian	Both	All ages	Larynx cancer	Smoking	Percent	2021	0.77032914	0.85237591	0.65647527
Deaths	Fujian	Both	All ages	Larynx cancer	High alcohol use	Percent	2021	0.1538038	0.2310358	0.07878475
Deaths	Fujian	Both	All ages	Larynx cancer	Occupational risks	Percent	2021	0.04423316	0.07842634	0.02044081

Deaths	Fujian	Both	All ages	Larynx cancer	Occupational carcinogens	Percent	2021	0.04423316	0.07842634	0.02044081
Deaths	Fujian	Both	All ages	Larynx cancer	Occupational exposure to asbestos	Percent	2021	0.00383466	0.00671581	0.00181254
Deaths	Chongqing	Both	All ages	Tracheal, bronchus, and lung cancer	Residential radon	Percent	2021	0.03372665	0.1585107	-0.013737
Deaths	Chongqing	Both	All ages	Tracheal, bronchus, and lung cancer	Behavioral risks	Percent	2021	0.6562503	0.84180754	0.33399913
Deaths	Tibet	Both	All ages	Tracheal, bronchus, and lung cancer	Diet low in fruits	Percent	2021	0.02790875	0.04314539	0.01426645
Deaths	Xinjiang	Both	All ages	Tracheal, bronchus, and lung cancer	Diet low in fruits	Percent	2021	0.02548171	0.03800596	0.01276239
Deaths	Xinjiang	Both	All ages	Tracheal, bronchus, and lung cancer	Environmental/occupational risks	Percent	2021	0.38584708	0.48761182	0.27690121
Deaths	Jiangxi	Both	All ages	Tracheal, bronchus, and lung cancer	Tobacco	Percent	2021	0.65205411	0.84444393	0.31262424
Deaths	Jiangxi	Both	All ages	Tracheal, bronchus, and lung cancer	Smoking	Percent	2021	0.61835121	0.80768411	0.31808111
Deaths	Jiangxi	Both	All ages	Tracheal, bronchus, and lung cancer	Secondhand smoke	Percent	2021	0.07396295	0.24758448	-0.1057815
Deaths	Jiangxi	Both	All ages	Tracheal, bronchus, and lung cancer	Metabolic risks	Percent	2021	0.01918972	0.04389411	-0.0039202

Deaths	Jiangxi	Both	All ages	Tracheal, bronchus, and lung cancer	High fasting plasma glucose	Percent	2021	0.01918972	0.04389411	-0.0039202
Deaths	Jiangxi	Both	All ages	Tracheal, bronchus, and lung cancer	Dietary risks	Percent	2021	0.02627837	0.04052169	0.01343804
Deaths	Taiwan (Province of China)	Both	All ages	Tracheal, bronchus, and lung cancer	Environmental/occupational risks	Percent	2021	0.21767021	0.27351324	0.1652712
Deaths	Shaanxi	Both	All ages	Tracheal, bronchus, and lung cancer	Particulate matter pollution	Percent	2021	0.28382196	0.380167	0.18504036
Deaths	Jiangxi	Both	All ages	Larynx cancer	Occupational exposure to sulfuric acid	Percent	2021	0.03992339	0.0761884	0.01633642
Deaths	Jiangxi	Both	All ages	Larynx cancer	All risk factors	Percent	2021	0.7751577	0.86604851	0.6763107
Deaths	Hebei	Both	All ages	Tracheal, bronchus, and lung cancer	Air pollution	Percent	2021	0.30130865	0.39942441	0.19928108
Deaths	Hebei	Both	All ages	Tracheal, bronchus, and lung cancer	Ambient particulate matter pollution	Percent	2021	0.25667034	0.35420878	0.15131213
Deaths	Hebei	Both	All ages	Tracheal, bronchus, and lung cancer	Household air pollution from solid fuels	Percent	2021	0.04447511	0.16425701	0.00397969
Deaths	Hebei	Both	All ages	Tracheal, bronchus, and lung cancer	Other environmental risks	Percent	2021	0.0397328	0.16045945	-0.0145674
Deaths	Xinjiang	Both	All ages	Larynx cancer	Tobacco	Percent	2021	0.68169765	0.79896991	0.54061386

Deaths	Taiwan (Province of China)	Both	All ages	Larynx cancer	Tobacco	Percent	2021	0.76780653	0.85709197	0.64751932
Deaths	Fujian	Both	All ages	Tracheal, bronchus, and lung cancer	Occupational risks	Percent	2021	0.07410139	0.10097949	0.05215073
Deaths	Fujian	Both	All ages	Tracheal, bronchus, and lung cancer	Occupational carcinogens	Percent	2021	0.07410139	0.10097949	0.05215073
Deaths	Fujian	Both	All ages	Tracheal, bronchus, and lung cancer	Occupational exposure to asbestos	Percent	2021	0.01110912	0.01846878	0.00561873
Deaths	Fujian	Both	All ages	Tracheal, bronchus, and lung cancer	Occupational exposure to arsenic	Percent	2021	0.00668401	0.0110373	0.00263847
Deaths	Fujian	Both	All ages	Tracheal, bronchus, and lung cancer	Occupational exposure to beryllium	Percent	2021	0.00022301	0.0002676	0.00018217
Deaths	Fujian	Both	All ages	Tracheal, bronchus, and lung cancer	Occupational exposure to cadmium	Percent	2021	0.00064868	0.00080237	0.0005224
Deaths	Xinjiang	Both	All ages	Larynx cancer	Smoking	Percent	2021	0.68169765	0.79896991	0.54061386
Deaths	Xinjiang	Both	All ages	Larynx cancer	High alcohol use	Percent	2021	0.12369097	0.18353768	0.06343375
Deaths	Xinjiang	Both	All ages	Larynx cancer	Occupational risks	Percent	2021	0.0511553	0.08701846	0.0271938
Deaths	Xinjiang	Both	All ages	Larynx cancer	Occupational carcinogens	Percent	2021	0.0511553	0.08701846	0.0271938

Deaths	Xinjiang	Both	All ages	Larynx cancer	Occupational exposure to asbestos	Percent	2021	0.0098012 1	0.0161621	0.0053516 5
Deaths	Xinjiang	Both	All ages	Tracheal, bronchus, and lung cancer	Air pollution	Percent	2021	0.2892908 6	0.3865745	0.1895915 7
Deaths	Xinjiang	Both	All ages	Tracheal, bronchus, and lung cancer	Ambient particulate matter pollution	Percent	2021	0.2393890 2	0.3357057 4	0.1351152 6
Deaths	Xinjiang	Both	All ages	Tracheal, bronchus, and lung cancer	Household air pollution from solid fuels	Percent	2021	0.0497625 6	0.1690631 2	0.0051431 8
Deaths	Xinjiang	Both	All ages	Tracheal, bronchus, and lung cancer	Other environmental risks	Percent	2021	0.0504601 7	0.1868241 2	-0.0202851
Deaths	China	Both	All ages	Tracheal, bronchus, and lung cancer	Particulate matter pollution	Percent	2021	0.2598383 8	0.3500016 3	0.1694339 1
Deaths	Jiangxi	Both	All ages	Larynx cancer	Environmental/occupational risks	Percent	2021	0.0496547 9	0.0852977 1	0.0267392
Deaths	Jiangxi	Both	All ages	Larynx cancer	Behavioral risks	Percent	2021	0.7633584 8	0.8604873 1	0.6613100 4
Deaths	Jiangxi	Both	All ages	Tracheal, bronchus, and lung cancer	Occupational exposure to chromium	Percent	2021	0.0013143 9	0.0015266 5	0.0011133 5
Deaths	Xinjiang	Both	All ages	Tracheal, bronchus, and lung cancer	Occupational risks	Percent	2021	0.0898226 3	0.1172088 5	0.0664114
Deaths	Xinjiang	Both	All ages	Tracheal, bronchus, and lung cancer	Occupational carcinogens	Percent	2021	0.0898226 3	0.1172088 5	0.0664114

Deaths	Xinjiang	Both	All ages	Tracheal, bronchus, and lung cancer	Occupational exposure to asbestos	Percent	2021	0.02930187	0.04431483	0.0181005
Deaths	Xinjiang	Both	All ages	Tracheal, bronchus, and lung cancer	Occupational exposure to arsenic	Percent	2021	0.00663378	0.01068609	0.00252276
Deaths	Xinjiang	Both	All ages	Tracheal, bronchus, and lung cancer	Occupational exposure to beryllium	Percent	2021	0.00024138	0.00028756	0.0001988
Deaths	Xinjiang	Both	All ages	Tracheal, bronchus, and lung cancer	Occupational exposure to cadmium	Percent	2021	0.00064635	0.00078602	0.00051973
Deaths	Hebei	Both	All ages	Tracheal, bronchus, and lung cancer	Diet low in fruits	Percent	2021	0.02478799	0.03750808	0.01223833
Deaths	Shandong	Both	All ages	Larynx cancer	Tobacco	Percent	2021	0.77379878	0.86807896	0.654058
Deaths	Shandong	Both	All ages	Tracheal, bronchus, and lung cancer	Tobacco	Percent	2021	0.64287276	0.84866066	0.28378054
Deaths	Shandong	Both	All ages	Tracheal, bronchus, and lung cancer	Smoking	Percent	2021	0.61302139	0.83828413	0.28371396
Deaths	Shandong	Both	All ages	Tracheal, bronchus, and lung cancer	Secondhand smoke	Percent	2021	0.07139717	0.24751264	-0.0965095
Deaths	Shandong	Both	All ages	Tracheal, bronchus, and lung cancer	Metabolic risks	Percent	2021	0.0223334	0.04967901	-0.0046609
Deaths	Shandong	Both	All ages	Tracheal, bronchus, and lung cancer	High fasting plasma glucose	Percent	2021	0.0223334	0.04967901	-0.0046609

Deaths	Shandong	Both	All ages	Tracheal, bronchus, and lung cancer	Dietary risks	Percent	2021	0.02110617	0.03175124	0.010916
Deaths	Yunnan	Both	All ages	Tracheal, bronchus, and lung cancer	Tobacco	Percent	2021	0.6632987 1	0.8372624 6	0.3569775 6
Deaths	Yunnan	Both	All ages	Tracheal, bronchus, and lung cancer	Smoking	Percent	2021	0.6226535 8	0.7958227 3	0.3499668 7
Deaths	Yunnan	Both	All ages	Tracheal, bronchus, and lung cancer	Secondhand smoke	Percent	2021	0.0800900 1	0.2719306 3	-0.1171555
Deaths	Yunnan	Both	All ages	Tracheal, bronchus, and lung cancer	Metabolic risks	Percent	2021	0.0192094 5	0.0434517 2	-0.0038308
Deaths	Yunnan	Both	All ages	Tracheal, bronchus, and lung cancer	High fasting plasma glucose	Percent	2021	0.0192094 5	0.0434517 2	-0.0038308
Deaths	Yunnan	Both	All ages	Tracheal, bronchus, and lung cancer	Dietary risks	Percent	2021	0.0287803 9	0.0432555	0.0146650 7
Deaths	Chongqing	Both	All ages	Tracheal, bronchus, and lung cancer	Particulate matter pollution	Percent	2021	0.2421517 5	0.3296757 6	0.1574336 3
Deaths	Hainan	Both	All ages	Tracheal, bronchus, and lung cancer	Particulate matter pollution	Percent	2021	0.1624759 9	0.2629804 7	0.0891978 7
Deaths	Yunnan	Both	All ages	Larynx cancer	Occupational exposure to sulfuric acid	Percent	2021	0.0462367 8	0.0861318 3	0.0195854 8
Deaths	Yunnan	Both	All ages	Larynx cancer	All risk factors	Percent	2021	0.7973893 3	0.8684941 2	0.7089553 1

Deaths	Jiangxi	Both	All ages	Tracheal, bronchus, and lung cancer	Occupational exposure to diesel engine exhaust	Percent	2021	0.01386804	0.01622167	0.01197812
Deaths	Jiangxi	Both	All ages	Tracheal, bronchus, and lung cancer	Occupational exposure to nickel	Percent	2021	0.00620759	0.01397117	0.00131818
Deaths	Jiangxi	Both	All ages	Tracheal, bronchus, and lung cancer	Occupational exposure to polycyclic aromatic hydrocarbons	Percent	2021	0.00445103	0.00539065	0.00363653
Deaths	Jiangxi	Both	All ages	Tracheal, bronchus, and lung cancer	Occupational exposure to silica	Percent	2021	0.02906199	0.04588463	0.01325848
Deaths	Jiangxi	Both	All ages	Tracheal, bronchus, and lung cancer	All risk factors	Percent	2021	0.78367938	0.90042095	0.56618489
Deaths	Heilongjiang	Both	All ages	Larynx cancer	Occupational exposure to sulfuric acid	Percent	2021	0.03438429	0.06338616	0.01380886
Deaths	Heilongjiang	Both	All ages	Larynx cancer	All risk factors	Percent	2021	0.83288492	0.90662527	0.72764584
Deaths	Jiangxi	Both	All ages	Tracheal, bronchus, and lung cancer	Diet low in fruits	Percent	2021	0.02627837	0.04052169	0.01343804
Deaths	Macao Special Administrative Region of China	Both	All ages	Tracheal, bronchus, and lung cancer	Particulate matter pollution	Percent	2021	0.16444506	0.22884715	0.10427558
Deaths	Yunnan	Both	All ages	Larynx cancer	Environmental/occupational risks	Percent	2021	0.0632219	0.1045178	0.03536756
Deaths	Yunnan	Both	All ages	Larynx cancer	Behavioral risks	Percent	2021	0.78270204	0.85896544	0.68808648

Deaths	Yunnan	Both	All ages	Tracheal, bronchus, and lung cancer	Occupational exposure to chromium	Percent	2021	0.0015417 2	0.0017784 2	0.0013096 6
Deaths	Jiangxi	Both	All ages	Tracheal, bronchus, and lung cancer	Environmental/occupational risks	Percent	2021	0.3474179 6	0.4526987 2	0.2447795 6
Deaths	Fujian	Both	All ages	Tracheal, bronchus, and lung cancer	Tobacco	Percent	2021	0.6470695 8	0.8352038 9	0.3083235 7
Deaths	Fujian	Both	All ages	Tracheal, bronchus, and lung cancer	Smoking	Percent	2021	0.6099501 2	0.8047261 8	0.3052663 4
Deaths	Fujian	Both	All ages	Tracheal, bronchus, and lung cancer	Secondhand smoke	Percent	2021	0.0774132 1	0.2757526 9	-0.1125716
Deaths	Fujian	Both	All ages	Tracheal, bronchus, and lung cancer	Metabolic risks	Percent	2021	0.0219149 2	0.0492139 4	-0.0044136
Deaths	Fujian	Both	All ages	Tracheal, bronchus, and lung cancer	High fasting plasma glucose	Percent	2021	0.0219149 2	0.0492139 4	-0.0044136
Deaths	Fujian	Both	All ages	Tracheal, bronchus, and lung cancer	Dietary risks	Percent	2021	0.0205190 2	0.0309191	0.0106180 3
Deaths	Heilongjiang	Both	All ages	Larynx cancer	Environmental/occupational risks	Percent	2021	0.0472043 3	0.0769013 7	0.0262023 9
Deaths	Heilongjiang	Both	All ages	Larynx cancer	Behavioral risks	Percent	2021	0.8248290 7	0.9029778 4	0.7156355 9
Deaths	Heilongjiang	Both	All ages	Tracheal, bronchus, and lung cancer	Occupational exposure to chromium	Percent	2021	0.0009973 1	0.0011663 4	0.0008432 6

Deaths	Yunnan	Both	All ages	Tracheal, bronchus, and lung cancer	Occupational exposure to diesel engine exhaust	Percent	2021	0.01547593	0.01782921	0.01322569
Deaths	Yunnan	Both	All ages	Tracheal, bronchus, and lung cancer	Occupational exposure to nickel	Percent	2021	0.00745537	0.01636525	0.00157691
Deaths	Yunnan	Both	All ages	Tracheal, bronchus, and lung cancer	Occupational exposure to polycyclic aromatic hydrocarbons	Percent	2021	0.00522907	0.00628937	0.00432262
Deaths	Yunnan	Both	All ages	Tracheal, bronchus, and lung cancer	Occupational exposure to silica	Percent	2021	0.03447096	0.05273337	0.01585824
Deaths	Yunnan	Both	All ages	Tracheal, bronchus, and lung cancer	All risk factors	Percent	2021	0.79310666	0.90171556	0.59719033
Deaths	Hebei	Both	All ages	Tracheal, bronchus, and lung cancer	Residential radon	Percent	2021	0.0397328	0.16045945	-0.0145674
Deaths	Hebei	Both	All ages	Tracheal, bronchus, and lung cancer	Behavioral risks	Percent	2021	0.60146378	0.82096538	0.26808326
Deaths	Jilin	Both	All ages	Tracheal, bronchus, and lung cancer	Tobacco	Percent	2021	0.61192721	0.84487723	0.24427864
Deaths	Jilin	Both	All ages	Tracheal, bronchus, and lung cancer	Smoking	Percent	2021	0.57384531	0.8359024	0.23031574
Deaths	Jilin	Both	All ages	Tracheal, bronchus, and lung cancer	Secondhand smoke	Percent	2021	0.08431955	0.29306199	-0.1206308
Deaths	Jilin	Both	All ages	Tracheal, bronchus, and lung cancer	Metabolic risks	Percent	2021	0.02158516	0.0489013	-0.0042

Deaths	Jilin	Both	All ages	Tracheal, bronchus, and lung cancer	High fasting plasma glucose	Percent	2021	0.02158516	0.0489013	-0.0042
Deaths	Jilin	Both	All ages	Tracheal, bronchus, and lung cancer	Dietary risks	Percent	2021	0.02274956	0.03427509	0.01103229
Deaths	Yunnan	Both	All ages	Tracheal, bronchus, and lung cancer	Environmental/occupational risks	Percent	2021	0.35180243	0.45863033	0.25625343
Deaths	Zhejiang	Both	All ages	Larynx cancer	Occupational exposure to sulfuric acid	Percent	2021	0.03962967	0.0728734	0.01560558
Deaths	Zhejiang	Both	All ages	Larynx cancer	All risk factors	Percent	2021	0.83107807	0.89846144	0.7311587
Deaths	Heilongjiang	Both	All ages	Tracheal, bronchus, and lung cancer	Occupational exposure to diesel engine exhaust	Percent	2021	0.01078106	0.01272071	0.00908661
Deaths	Heilongjiang	Both	All ages	Tracheal, bronchus, and lung cancer	Occupational exposure to nickel	Percent	2021	0.00467449	0.01049343	0.00095375
Deaths	Heilongjiang	Both	All ages	Tracheal, bronchus, and lung cancer	Occupational exposure to polycyclic aromatic hydrocarbons	Percent	2021	0.00339944	0.00406528	0.0027866
Deaths	Heilongjiang	Both	All ages	Tracheal, bronchus, and lung cancer	Occupational exposure to silica	Percent	2021	0.02185896	0.03432866	0.01008985
Deaths	Heilongjiang	Both	All ages	Tracheal, bronchus, and lung cancer	All risk factors	Percent	2021	0.81061001	0.95370523	0.56393747
Deaths	Heilongjiang	Both	All ages	Tracheal, bronchus, and lung cancer	Environmental/occupational risks	Percent	2021	0.34379913	0.48119177	0.23552807

Deaths	Heilongjiang	Both	All ages	Larynx cancer	Tobacco	Percent	2021	0.79994533	0.88952361	0.67282311
Deaths	Zhejiang	Both	All ages	Larynx cancer	Environmental/occupational risks	Percent	2021	0.04662942	0.08036001	0.02384476
Deaths	Zhejiang	Both	All ages	Larynx cancer	Behavioral risks	Percent	2021	0.82318231	0.89405079	0.71725337
Deaths	Taiwan (Province of China)	Both	All ages	Tracheal, bronchus, and lung cancer	Tobacco	Percent	2021	0.52430308	0.71731802	0.20219137
Deaths	Taiwan (Province of China)	Both	All ages	Tracheal, bronchus, and lung cancer	Smoking	Percent	2021	0.4908839	0.69209698	0.20410591
Deaths	Taiwan (Province of China)	Both	All ages	Tracheal, bronchus, and lung cancer	Secondhand smoke	Percent	2021	0.06115593	0.23037932	-0.0734307
Deaths	Taiwan (Province of China)	Both	All ages	Tracheal, bronchus, and lung cancer	Metabolic risks	Percent	2021	0.02775098	0.06303015	-0.0056486
Deaths	Taiwan (Province of China)	Both	All ages	Tracheal, bronchus, and lung cancer	High fasting plasma glucose	Percent	2021	0.02775098	0.06303015	-0.0056486
Deaths	Taiwan (Province of China)	Both	All ages	Tracheal, bronchus, and lung cancer	Dietary risks	Percent	2021	0.01336566	0.01972804	0.00667606
Deaths	Taiwan (Province of China)	Both	All ages	Tracheal, bronchus, and lung cancer	Air pollution	Percent	2021	0.12616184	0.17716215	0.07912643
Deaths	Taiwan (Province of China)	Both	All ages	Tracheal, bronchus, and lung cancer	Ambient particulate matter pollution	Percent	2021	0.12517781	0.17583061	0.07777903

Deaths	Taiwan (Province of China)	Both	All ages	Tracheal, bronchus, and lung cancer	Household air pollution from solid fuels	Percent	2021	0.0009545456	0.0055489898	8.85E-11
Deaths	Taiwan (Province of China)	Both	All ages	Tracheal, bronchus, and lung cancer	Other environmental risks	Percent	2021	0.0106229224	0.0328417477	-0.0048141
Deaths	Shandong	Both	All ages	Tracheal, bronchus, and lung cancer	Diet low in fruits	Percent	2021	0.0211061617	0.0317512524	0.010916
Deaths	Zhejiang	Both	All ages	Tracheal, bronchus, and lung cancer	Occupational exposure to diesel engine exhaust	Percent	2021	0.0148467478	0.0174828855	0.01259737
Deaths	Zhejiang	Both	All ages	Tracheal, bronchus, and lung cancer	Occupational exposure to nickel	Percent	2021	0.0061999422	0.0136239838	0.00134158
Deaths	Zhejiang	Both	All ages	Tracheal, bronchus, and lung cancer	Occupational exposure to polycyclic aromatic hydrocarbons	Percent	2021	0.0045663838	0.0055654659	0.0036832
Deaths	Zhejiang	Both	All ages	Tracheal, bronchus, and lung cancer	Occupational exposure to silica	Percent	2021	0.0290938181	0.0464197177	0.0135222
Deaths	Zhejiang	Both	All ages	Tracheal, bronchus, and lung cancer	All risk factors	Percent	2021	0.77081097	0.90308768	0.54566869
Deaths	Fujian	Both	All ages	Tracheal, bronchus, and lung cancer	Diet low in fruits	Percent	2021	0.02051902	0.0309191	0.01061803
Deaths	Yunnan	Both	All ages	Tracheal, bronchus, and lung cancer	Diet low in fruits	Percent	2021	0.02878039	0.0432555	0.01466507
Deaths	Zhejiang	Both	All ages	Tracheal, bronchus, and lung cancer	Environmental/occupational risks	Percent	2021	0.30301894	0.38280064	0.22219884

Deaths	Jilin	Both	All ages	Tracheal, bronchus, and lung cancer	Diet low in fruits	Percent	2021	0.02274956	0.03427509	0.01103229
Deaths	Taiwan (Province of China)	Both	All ages	Tracheal, bronchus, and lung cancer	Diet low in fruits	Percent	2021	0.01336566	0.01972804	0.00667606
Deaths	Gansu	Both	All ages	Tracheal, bronchus, and lung cancer	Tobacco	Percent	2021	0.65473237	0.84213299	0.31959991
Deaths	Gansu	Both	All ages	Tracheal, bronchus, and lung cancer	Smoking	Percent	2021	0.61608737	0.81027192	0.3274449
Deaths	Gansu	Both	All ages	Tracheal, bronchus, and lung cancer	Secondhand smoke	Percent	2021	0.07916849	0.2631963	-0.1133974
Deaths	Gansu	Both	All ages	Tracheal, bronchus, and lung cancer	Metabolic risks	Percent	2021	0.01788249	0.03993435	-0.0034946
Deaths	Gansu	Both	All ages	Tracheal, bronchus, and lung cancer	High fasting plasma glucose	Percent	2021	0.01788249	0.03993435	-0.0034946
Deaths	Gansu	Both	All ages	Tracheal, bronchus, and lung cancer	Dietary risks	Percent	2021	0.02957024	0.04532196	0.0149358
Deaths	Jiangsu	Both	All ages	Tracheal, bronchus, and lung cancer	Air pollution	Percent	2021	0.26367309	0.3504463	0.17508913
Deaths	Jiangsu	Both	All ages	Tracheal, bronchus, and lung cancer	Ambient particulate matter pollution	Percent	2021	0.25912857	0.3449091	0.17204623
Deaths	Jiangsu	Both	All ages	Tracheal, bronchus, and lung cancer	Household air pollution from solid fuels	Percent	2021	0.00449116	0.04044682	6.29E-06

Deaths	Jiangsu	Both	All ages	Tracheal, bronchus, and lung cancer	Other environmental risks	Percent	2021	0.03733019	0.1652168	-0.0111118
Deaths	Xinjiang	Both	All ages	Tracheal, bronchus, and lung cancer	Residential radon	Percent	2021	0.05046017	0.18682412	-0.0202851
Deaths	Xinjiang	Both	All ages	Tracheal, bronchus, and lung cancer	Behavioral risks	Percent	2021	0.52431784	0.75910108	0.19961384
Deaths	Fujian	Both	All ages	Tracheal, bronchus, and lung cancer	Air pollution	Percent	2021	0.17925396	0.25104217	0.11373004
Deaths	Fujian	Both	All ages	Tracheal, bronchus, and lung cancer	Ambient particulate matter pollution	Percent	2021	0.1665489	0.23005565	0.10448755
Deaths	Fujian	Both	All ages	Tracheal, bronchus, and lung cancer	Household air pollution from solid fuels	Percent	2021	0.01263636	0.0929714	0.0013373
Deaths	Fujian	Both	All ages	Tracheal, bronchus, and lung cancer	Other environmental risks	Percent	2021	0.0310538	0.12659225	-0.0111897
Deaths	Heilongjiang	Both	All ages	Tracheal, bronchus, and lung cancer	Tobacco	Percent	2021	0.69727716	0.920051	0.29075197
Deaths	Heilongjiang	Both	All ages	Tracheal, bronchus, and lung cancer	Smoking	Percent	2021	0.67044653	0.91464672	0.28701705
Deaths	Heilongjiang	Both	All ages	Tracheal, bronchus, and lung cancer	Secondhand smoke	Percent	2021	0.08348463	0.2889546	-0.1223965
Deaths	Heilongjiang	Both	All ages	Tracheal, bronchus, and lung cancer	Metabolic risks	Percent	2021	0.02266977	0.05064211	-0.0045951

Deaths	Heilongjiang	Both	All ages	Tracheal, bronchus, and lung cancer	High fasting plasma glucose	Percent	2021	0.02266977	0.05064211	-0.0045951
Deaths	Heilongjiang	Both	All ages	Tracheal, bronchus, and lung cancer	Dietary risks	Percent	2021	0.0250706	0.03763286	0.01365111
Deaths	Shanxi	Both	All ages	Tracheal, bronchus, and lung cancer	Tobacco	Percent	2021	0.67693633	0.87937265	0.32505689
Deaths	Shanxi	Both	All ages	Tracheal, bronchus, and lung cancer	Smoking	Percent	2021	0.64705687	0.86810163	0.31832847
Deaths	Shanxi	Both	All ages	Tracheal, bronchus, and lung cancer	Secondhand smoke	Percent	2021	0.0709977	0.25580011	-0.0993209
Deaths	Shanxi	Both	All ages	Tracheal, bronchus, and lung cancer	Metabolic risks	Percent	2021	0.02408621	0.05342388	-0.0048102
Deaths	Shanxi	Both	All ages	Tracheal, bronchus, and lung cancer	High fasting plasma glucose	Percent	2021	0.02408621	0.05342388	-0.0048102
Deaths	Shanxi	Both	All ages	Tracheal, bronchus, and lung cancer	Dietary risks	Percent	2021	0.02631632	0.04006497	0.01324335
Deaths	Shanghai	Both	All ages	Tracheal, bronchus, and lung cancer	Tobacco	Percent	2021	0.58958239	0.80556505	0.25439682
Deaths	Shanghai	Both	All ages	Tracheal, bronchus, and lung cancer	Smoking	Percent	2021	0.55750067	0.77814889	0.26576337
Deaths	Shanghai	Both	All ages	Tracheal, bronchus, and lung cancer	Secondhand smoke	Percent	2021	0.06388125	0.22523718	-0.0806579

Deaths	Shanghai	Both	All ages	Tracheal, bronchus, and lung cancer	Metabolic risks	Percent	2021	0.0262894	0.06076835	-0.0052547
Deaths	Shanghai	Both	All ages	Tracheal, bronchus, and lung cancer	High fasting plasma glucose	Percent	2021	0.0262894	0.06076835	-0.0052547
Deaths	Shanghai	Both	All ages	Tracheal, bronchus, and lung cancer	Dietary risks	Percent	2021	0.01794331	0.02787573	0.00863297
Deaths	Shanxi	Both	All ages	Tracheal, bronchus, and lung cancer	Diet low in fruits	Percent	2021	0.02631632	0.04006497	0.01324335
Deaths	Jilin	Both	All ages	Larynx cancer	Occupational exposure to sulfuric acid	Percent	2021	0.03510892	0.06550549	0.01416704
Deaths	Jilin	Both	All ages	Larynx cancer	All risk factors	Percent	2021	0.77229702	0.87208107	0.65295406
Deaths	Heilongjiang	Both	All ages	Tracheal, bronchus, and lung cancer	Diet low in fruits	Percent	2021	0.0250706	0.03763286	0.01365111
Deaths	Henan	Both	All ages	Tracheal, bronchus, and lung cancer	Tobacco	Percent	2021	0.60786164	0.82571986	0.25420274
Deaths	Henan	Both	All ages	Tracheal, bronchus, and lung cancer	Smoking	Percent	2021	0.57426128	0.80752894	0.26245731
Deaths	Henan	Both	All ages	Tracheal, bronchus, and lung cancer	Secondhand smoke	Percent	2021	0.07247694	0.24462818	-0.0946937
Deaths	Henan	Both	All ages	Tracheal, bronchus, and lung cancer	Metabolic risks	Percent	2021	0.02097897	0.04665704	-0.0041678

Deaths	Henan	Both	All ages	Tracheal, bronchus, and lung cancer	High fasting plasma glucose	Percent	2021	0.02097897	0.04665704	-0.0041678
Deaths	Henan	Both	All ages	Tracheal, bronchus, and lung cancer	Dietary risks	Percent	2021	0.02536569	0.03802569	0.01188148
Deaths	Gansu	Both	All ages	Tracheal, bronchus, and lung cancer	Diet low in fruits	Percent	2021	0.02957024	0.04532196	0.0149358
Deaths	Gansu	Both	All ages	Larynx cancer	Tobacco	Percent	2021	0.75001195	0.83575858	0.63231912
Deaths	Jilin	Both	All ages	Larynx cancer	Environmental/occupational risks	Percent	2021	0.05312401	0.08389695	0.03085885
Deaths	Jilin	Both	All ages	Larynx cancer	Behavioral risks	Percent	2021	0.75906304	0.86545239	0.63505824
Deaths	Jilin	Both	All ages	Tracheal, bronchus, and lung cancer	Occupational exposure to chromium	Percent	2021	0.00117049	0.00139247	0.00098246
Deaths	Zhejiang	Both	All ages	Tracheal, bronchus, and lung cancer	Tobacco	Percent	2021	0.65720143	0.85382146	0.32515647
Deaths	Zhejiang	Both	All ages	Tracheal, bronchus, and lung cancer	Smoking	Percent	2021	0.62733984	0.82899576	0.32235134
Deaths	Zhejiang	Both	All ages	Tracheal, bronchus, and lung cancer	Secondhand smoke	Percent	2021	0.06696333	0.23659706	-0.0900082
Deaths	Zhejiang	Both	All ages	Tracheal, bronchus, and lung cancer	Metabolic risks	Percent	2021	0.02006565	0.04464597	-0.004038

Deaths	Zhejiang	Both	All ages	Tracheal, bronchus, and lung cancer	High fasting plasma glucose	Percent	2021	0.02006565	0.04464597	-0.004038
Deaths	Zhejiang	Both	All ages	Tracheal, bronchus, and lung cancer	Dietary risks	Percent	2021	0.02466461	0.0379406	0.0127605
Deaths	Jiangxi	Both	All ages	Tracheal, bronchus, and lung cancer	Air pollution	Percent	2021	0.25388975	0.35273638	0.16353345
Deaths	Jiangxi	Both	All ages	Tracheal, bronchus, and lung cancer	Ambient particulate matter pollution	Percent	2021	0.17712178	0.25858935	0.08664562
Deaths	Jiangxi	Both	All ages	Tracheal, bronchus, and lung cancer	Household air pollution from solid fuels	Percent	2021	0.07667934	0.2106207	0.01151956
Deaths	Jiangxi	Both	All ages	Tracheal, bronchus, and lung cancer	Other environmental risks	Percent	2021	0.04056823	0.16975941	-0.0147847
Deaths	Jilin	Both	All ages	Tracheal, bronchus, and lung cancer	Occupational exposure to diesel engine exhaust	Percent	2021	0.01269466	0.01504458	0.01071446
Deaths	Jilin	Both	All ages	Tracheal, bronchus, and lung cancer	Occupational exposure to nickel	Percent	2021	0.00528417	0.01178054	0.00109564
Deaths	Jilin	Both	All ages	Tracheal, bronchus, and lung cancer	Occupational exposure to polycyclic aromatic hydrocarbons	Percent	2021	0.00398821	0.0049157	0.00329219
Deaths	Jilin	Both	All ages	Tracheal, bronchus, and lung cancer	Occupational exposure to silica	Percent	2021	0.02428041	0.0387163	0.01125629
Deaths	Jilin	Both	All ages	Tracheal, bronchus, and lung cancer	All risk factors	Percent	2021	0.76037741	0.90868083	0.52487794

Deaths	Jilin	Both	All ages	Tracheal, bronchus, and lung cancer	Environmental/occupational risks	Percent	2021	0.35470425	0.46854261	0.24979661
Deaths	Zhejiang	Both	All ages	Tracheal, bronchus, and lung cancer	Diet low in fruits	Percent	2021	0.02466461	0.0379406	0.0127605
Deaths	Shanghai	Both	All ages	Tracheal, bronchus, and lung cancer	Diet low in fruits	Percent	2021	0.01794331	0.02787573	0.00863297
Deaths	Yunnan	Both	All ages	Larynx cancer	Tobacco	Percent	2021	0.76661661	0.8471209	0.67278868
Deaths	Henan	Both	All ages	Larynx cancer	Tobacco	Percent	2021	0.70693863	0.81229831	0.57380863
Deaths	Jiangxi	Both	All ages	Larynx cancer	Tobacco	Percent	2021	0.74125995	0.84605905	0.62904055
Deaths	Jilin	Both	All ages	Larynx cancer	Tobacco	Percent	2021	0.73750239	0.84910498	0.59927356
Deaths	Shandong	Both	All ages	Tracheal, bronchus, and lung cancer	Air pollution	Percent	2021	0.28761372	0.37883997	0.19030699
Deaths	Shandong	Both	All ages	Tracheal, bronchus, and lung cancer	Ambient particulate matter pollution	Percent	2021	0.27186936	0.36444501	0.17356169
Deaths	Shandong	Both	All ages	Tracheal, bronchus, and lung cancer	Household air pollution from solid fuels	Percent	2021	0.01560578	0.10883582	0.00022802
Deaths	Shandong	Both	All ages	Tracheal, bronchus, and lung cancer	Other environmental risks	Percent	2021	0.03058433	0.11927074	-0.0124945

Deaths	Hebei	Both	All ages	Larynx cancer	Smoking	Percent	2021	0.71431268	0.81876992	0.58385614
Deaths	Hebei	Both	All ages	Larynx cancer	High alcohol use	Percent	2021	0.13831785	0.20793516	0.07093207
Deaths	Hebei	Both	All ages	Larynx cancer	Occupational risks	Percent	2021	0.06374527	0.10134062	0.03624711
Deaths	Hebei	Both	All ages	Larynx cancer	Occupational carcinogens	Percent	2021	0.06374527	0.10134062	0.03624711
Deaths	Hebei	Both	All ages	Larynx cancer	Occupational exposure to asbestos	Percent	2021	0.02086897	0.03302817	0.0110232
Deaths	Taiwan (Province of China)	Both	All ages	Larynx cancer	Smoking	Percent	2021	0.76780653	0.85709197	0.64751932
Deaths	Taiwan (Province of China)	Both	All ages	Larynx cancer	High alcohol use	Percent	2021	0.1185457	0.17410317	0.06172071
Deaths	Taiwan (Province of China)	Both	All ages	Larynx cancer	Occupational risks	Percent	2021	0.05444189	0.08865395	0.02920448
Deaths	Taiwan (Province of China)	Both	All ages	Larynx cancer	Occupational carcinogens	Percent	2021	0.05444189	0.08865395	0.02920448
Deaths	Taiwan (Province of China)	Both	All ages	Larynx cancer	Occupational exposure to asbestos	Percent	2021	0.0145426	0.02695408	0.00653111
Deaths	Taiwan (Province of China)	Both	All ages	Tracheal, bronchus, and lung cancer	Occupational risks	Percent	2021	0.09513081	0.12319707	0.07021826

Deaths	Taiwan (Province of China)	Both	All ages	Tracheal, bronchus, and lung cancer	Occupational carcinogens	Percent	2021	0.09513081	0.12319707	0.07021826
Deaths	Taiwan (Province of China)	Both	All ages	Tracheal, bronchus, and lung cancer	Occupational exposure to asbestos	Percent	2021	0.0400699	0.06481519	0.02171582
Deaths	Taiwan (Province of China)	Both	All ages	Tracheal, bronchus, and lung cancer	Occupational exposure to arsenic	Percent	2021	0.00567004	0.00927239	0.00215598
Deaths	Taiwan (Province of China)	Both	All ages	Tracheal, bronchus, and lung cancer	Occupational exposure to beryllium	Percent	2021	0.0001846	0.00021731	0.00015348
Deaths	Taiwan (Province of China)	Both	All ages	Tracheal, bronchus, and lung cancer	Occupational exposure to cadmium	Percent	2021	0.00055146	0.00064491	0.00045688
Deaths	Hebei	Both	All ages	Tracheal, bronchus, and lung cancer	Occupational risks	Percent	2021	0.12138591	0.15542178	0.09019138
Deaths	Hebei	Both	All ages	Tracheal, bronchus, and lung cancer	Occupational carcinogens	Percent	2021	0.12138591	0.15542178	0.09019138
Deaths	Hebei	Both	All ages	Tracheal, bronchus, and lung cancer	Occupational exposure to asbestos	Percent	2021	0.0582829	0.08413799	0.03670073
Deaths	Hebei	Both	All ages	Tracheal, bronchus, and lung cancer	Occupational exposure to arsenic	Percent	2021	0.00706806	0.01156788	0.00256783
Deaths	Hebei	Both	All ages	Tracheal, bronchus, and lung cancer	Occupational exposure to beryllium	Percent	2021	0.00024812	0.00029316	0.00020555
Deaths	Hebei	Both	All ages	Tracheal, bronchus, and lung cancer	Occupational exposure to cadmium	Percent	2021	0.00068846	0.00083657	0.00055214

Deaths	Henan	Both	All ages	Tracheal, bronchus, and lung cancer	Diet low in fruits	Percent	2021	0.02536569	0.03802569	0.01188148
Deaths	Shanxi	Both	All ages	Larynx cancer	Occupational exposure to sulfuric acid	Percent	2021	0.03991825	0.07303272	0.01654526
Deaths	Shanxi	Both	All ages	Larynx cancer	All risk factors	Percent	2021	0.79890839	0.88250591	0.69886562
Deaths	Hebei	Both	All ages	Tracheal, bronchus, and lung cancer	Particulate matter pollution	Percent	2021	0.30130865	0.39942441	0.19928108
Deaths	Shanxi	Both	All ages	Larynx cancer	Environmental/occupational risks	Percent	2021	0.0519816	0.08577062	0.02850447
Deaths	Shanxi	Both	All ages	Larynx cancer	Behavioral risks	Percent	2021	0.78819456	0.87587188	0.68362688
Deaths	Shanxi	Both	All ages	Tracheal, bronchus, and lung cancer	Occupational exposure to diesel engine exhaust	Percent	2021	0.01352491	0.01586923	0.01153235
Deaths	Shanxi	Both	All ages	Tracheal, bronchus, and lung cancer	Occupational exposure to nickel	Percent	2021	0.0057792	0.01306615	0.00121819
Deaths	Shanxi	Both	All ages	Tracheal, bronchus, and lung cancer	Occupational exposure to polycyclic aromatic hydrocarbons	Percent	2021	0.00419115	0.00502512	0.00340214
Deaths	Shanxi	Both	All ages	Tracheal, bronchus, and lung cancer	Occupational exposure to silica	Percent	2021	0.02720957	0.04358924	0.01296327
Deaths	Shanxi	Both	All ages	Tracheal, bronchus, and lung cancer	All risk factors	Percent	2021	0.81221914	0.93243999	0.61309793

Deaths	Shanxi	Both	All ages	Tracheal, bronchus, and lung cancer	Environmental/occupational risks	Percent	2021	0.38963272	0.49005332	0.28506986
Deaths	Guangdong	Both	All ages	Tracheal, bronchus, and lung cancer	Tobacco	Percent	2021	0.66430315	0.86246506	0.31688623
Deaths	Guangdong	Both	All ages	Tracheal, bronchus, and lung cancer	Smoking	Percent	2021	0.63018841	0.84057735	0.31789944
Deaths	Guangdong	Both	All ages	Tracheal, bronchus, and lung cancer	Secondhand smoke	Percent	2021	0.07658936	0.26304499	-0.1066584
Deaths	Guangdong	Both	All ages	Tracheal, bronchus, and lung cancer	Metabolic risks	Percent	2021	0.02157945	0.04758412	-0.00432
Deaths	Guangdong	Both	All ages	Tracheal, bronchus, and lung cancer	High fasting plasma glucose	Percent	2021	0.02157945	0.04758412	-0.00432
Deaths	Guangdong	Both	All ages	Tracheal, bronchus, and lung cancer	Dietary risks	Percent	2021	0.02110737	0.03134763	0.0103821
Deaths	Guangdong	Both	All ages	Tracheal, bronchus, and lung cancer	Diet low in fruits	Percent	2021	0.02110737	0.03134763	0.0103821
Deaths	Xinjiang	Both	All ages	Tracheal, bronchus, and lung cancer	Particulate matter pollution	Percent	2021	0.28929086	0.3865745	0.18959157
Deaths	Yunnan	Both	All ages	Tracheal, bronchus, and lung cancer	Air pollution	Percent	2021	0.23781839	0.33610364	0.14899265
Deaths	Yunnan	Both	All ages	Tracheal, bronchus, and lung cancer	Ambient particulate matter pollution	Percent	2021	0.12599464	0.19188673	0.05816271

Deaths	Yunnan	Both	All ages	Tracheal, bronchus, and lung cancer	Household air pollution from solid fuels	Percent	2021	0.11169666	0.22816477	0.03000836
Deaths	Yunnan	Both	All ages	Tracheal, bronchus, and lung cancer	Other environmental risks	Percent	2021	0.03569654	0.12065006	-0.0131487
Deaths	Taiwan (Province of China)	Both	All ages	Tracheal, bronchus, and lung cancer	Residential radon	Percent	2021	0.01062294	0.03284177	-0.0048141
Deaths	Taiwan (Province of China)	Both	All ages	Tracheal, bronchus, and lung cancer	Behavioral risks	Percent	2021	0.53082704	0.72132463	0.21139038
Deaths	Henan	Both	All ages	Larynx cancer	Occupational exposure to sulfuric acid	Percent	2021	0.04443905	0.0803716	0.01824664
Deaths	Henan	Both	All ages	Larynx cancer	All risk factors	Percent	2021	0.74340667	0.83530083	0.63047077
Deaths	Shandong	Both	All ages	Larynx cancer	Smoking	Percent	2021	0.77379878	0.86807896	0.654058
Deaths	Shandong	Both	All ages	Larynx cancer	High alcohol use	Percent	2021	0.15675754	0.22903191	0.08200505
Deaths	Shandong	Both	All ages	Larynx cancer	Occupational risks	Percent	2021	0.05121539	0.08656221	0.02616996
Deaths	Shandong	Both	All ages	Larynx cancer	Occupational carcinogens	Percent	2021	0.05121539	0.08656221	0.02616996
Deaths	Shandong	Both	All ages	Larynx cancer	Occupational exposure to asbestos	Percent	2021	0.00813956	0.01350458	0.00430239

Deaths	Shandong	Both	All ages	Tracheal, bronchus, and lung cancer	Occupational risks	Percent	2021	0.08829897	0.11591215	0.06486814
Deaths	Shandong	Both	All ages	Tracheal, bronchus, and lung cancer	Occupational carcinogens	Percent	2021	0.08829897	0.11591215	0.06486814
Deaths	Shandong	Both	All ages	Tracheal, bronchus, and lung cancer	Occupational exposure to asbestos	Percent	2021	0.02404897	0.0366008	0.0139047
Deaths	Shandong	Both	All ages	Tracheal, bronchus, and lung cancer	Occupational exposure to arsenic	Percent	2021	0.00696498	0.01136038	0.00256267
Deaths	Shandong	Both	All ages	Tracheal, bronchus, and lung cancer	Occupational exposure to beryllium	Percent	2021	0.00024185	0.00028387	0.00019925
Deaths	Shandong	Both	All ages	Tracheal, bronchus, and lung cancer	Occupational exposure to cadmium	Percent	2021	0.00067861	0.00080788	0.00054327
Deaths	Heilongjiang	Both	All ages	Tracheal, bronchus, and lung cancer	Air pollution	Percent	2021	0.24007644	0.33364925	0.15125097
Deaths	Heilongjiang	Both	All ages	Tracheal, bronchus, and lung cancer	Ambient particulate matter pollution	Percent	2021	0.17690782	0.25779613	0.08892999
Deaths	Heilongjiang	Both	All ages	Tracheal, bronchus, and lung cancer	Household air pollution from solid fuels	Percent	2021	0.0630919	0.19087985	0.0072984
Deaths	Heilongjiang	Both	All ages	Tracheal, bronchus, and lung cancer	Other environmental risks	Percent	2021	0.05615894	0.21380614	-0.0276093
Deaths	Shandong	Both	All ages	Tracheal, bronchus, and lung cancer	Occupational exposure to chromium	Percent	2021	0.0014479	0.00166795	0.00123906

Deaths	Hubei	Both	All ages	Tracheal, bronchus, and lung cancer	Tobacco	Percent	2021	0.63945178	0.81980431	0.31031467
Deaths	Hubei	Both	All ages	Tracheal, bronchus, and lung cancer	Smoking	Percent	2021	0.60470174	0.79120786	0.31384644
Deaths	Hubei	Both	All ages	Tracheal, bronchus, and lung cancer	Secondhand smoke	Percent	2021	0.07468086	0.2594134	-0.1038687
Deaths	Hubei	Both	All ages	Tracheal, bronchus, and lung cancer	Metabolic risks	Percent	2021	0.01724766	0.03919341	-0.0036336
Deaths	Hubei	Both	All ages	Tracheal, bronchus, and lung cancer	High fasting plasma glucose	Percent	2021	0.01724766	0.03919341	-0.0036336
Deaths	Hubei	Both	All ages	Tracheal, bronchus, and lung cancer	Dietary risks	Percent	2021	0.02258851	0.03582214	0.0121958
Deaths	Liaoning	Both	All ages	Tracheal, bronchus, and lung cancer	Tobacco	Percent	2021	0.65340013	0.87925675	0.27859377
Deaths	Liaoning	Both	All ages	Tracheal, bronchus, and lung cancer	Smoking	Percent	2021	0.62475235	0.88010604	0.2919459
Deaths	Liaoning	Both	All ages	Tracheal, bronchus, and lung cancer	Secondhand smoke	Percent	2021	0.07543042	0.25214278	-0.103641
Deaths	Liaoning	Both	All ages	Tracheal, bronchus, and lung cancer	Metabolic risks	Percent	2021	0.025056	0.05546193	-0.0050983
Deaths	Liaoning	Both	All ages	Tracheal, bronchus, and lung cancer	High fasting plasma glucose	Percent	2021	0.025056	0.05546193	-0.0050983

Deaths	Liaoning	Both	All ages	Tracheal, bronchus, and lung cancer	Dietary risks	Percent	2021	0.02227788	0.03375939	0.0114972
Deaths	Liaoning	Both	All ages	Tracheal, bronchus, and lung cancer	Diet low in fruits	Percent	2021	0.02227788	0.03375939	0.0114972
Deaths	Fujian	Both	All ages	Tracheal, bronchus, and lung cancer	Residential radon	Percent	2021	0.0310538	0.12659225	-0.0111897
Deaths	Fujian	Both	All ages	Tracheal, bronchus, and lung cancer	Behavioral risks	Percent	2021	0.6542917	0.83872754	0.32197628
Deaths	Hubei	Both	All ages	Tracheal, bronchus, and lung cancer	Diet low in fruits	Percent	2021	0.02258851	0.03582214	0.0121958
Deaths	Gansu	Both	All ages	Larynx cancer	Occupational exposure to sulfuric acid	Percent	2021	0.04215076	0.07822351	0.01756375
Deaths	Gansu	Both	All ages	Larynx cancer	All risk factors	Percent	2021	0.77707673	0.85410224	0.66494981
Deaths	Shanghai	Both	All ages	Larynx cancer	Tobacco	Percent	2021	0.77858562	0.87675068	0.65781808
Deaths	Zhejiang	Both	All ages	Larynx cancer	Tobacco	Percent	2021	0.79805008	0.87743864	0.68328235
Deaths	Shanghai	Both	All ages	Tracheal, bronchus, and lung cancer	Air pollution	Percent	2021	0.23052976	0.30877298	0.15127252
Deaths	Shanghai	Both	All ages	Tracheal, bronchus, and lung cancer	Ambient particulate matter pollution	Percent	2021	0.22935949	0.30601221	0.1491536

Deaths	Shanghai	Both	All ages	Tracheal, bronchus, and lung cancer	Household air pollution from solid fuels	Percent	2021	0.00112564	0.00983717	3.77E-07
Deaths	Shanghai	Both	All ages	Tracheal, bronchus, and lung cancer	Other environmental risks	Percent	2021	0.03319356	0.13476499	-0.0127658
Deaths	Gansu	Both	All ages	Tracheal, bronchus, and lung cancer	Air pollution	Percent	2021	0.28453332	0.39021634	0.18514778
Deaths	Gansu	Both	All ages	Tracheal, bronchus, and lung cancer	Ambient particulate matter pollution	Percent	2021	0.14221256	0.2216179	0.06476671
Deaths	Gansu	Both	All ages	Tracheal, bronchus, and lung cancer	Household air pollution from solid fuels	Percent	2021	0.14221075	0.27894786	0.04704182
Deaths	Gansu	Both	All ages	Tracheal, bronchus, and lung cancer	Other environmental risks	Percent	2021	0.09760937	0.30118111	-0.0532428
Deaths	Shanxi	Both	All ages	Larynx cancer	Tobacco	Percent	2021	0.77194575	0.86363437	0.65803726
Deaths	Shandong	Both	All ages	Tracheal, bronchus, and lung cancer	Residential radon	Percent	2021	0.03058433	0.11927074	-0.0124945
Deaths	Shandong	Both	All ages	Tracheal, bronchus, and lung cancer	Behavioral risks	Percent	2021	0.65030579	0.85090465	0.29765415
Deaths	Jiangsu	Both	All ages	Tracheal, bronchus, and lung cancer	Residential radon	Percent	2021	0.03733019	0.1652168	-0.0111118
Deaths	Jiangsu	Both	All ages	Tracheal, bronchus, and lung cancer	Behavioral risks	Percent	2021	0.67123416	0.88748387	0.31202405

Deaths	Liaoning	Both	All ages	Larynx cancer	Tobacco	Percent	2021	0.7824864	0.87505561	0.66197686
Deaths	China	Both	All ages	Tracheal, bronchus, and lung cancer	Tobacco	Percent	2021	0.64806906	0.84747481	0.2961918
Deaths	Guangxi	Both	All ages	Tracheal, bronchus, and lung cancer	Tobacco	Percent	2021	0.60397321	0.80914025	0.25119819
Deaths	Guangxi	Both	All ages	Tracheal, bronchus, and lung cancer	Smoking	Percent	2021	0.56850903	0.78773697	0.26269261
Deaths	Guangxi	Both	All ages	Tracheal, bronchus, and lung cancer	Secondhand smoke	Percent	2021	0.07336709	0.24581189	-0.0889145
Deaths	Guangxi	Both	All ages	Tracheal, bronchus, and lung cancer	Metabolic risks	Percent	2021	0.02103601	0.04736014	-0.0044329
Deaths	Guangxi	Both	All ages	Tracheal, bronchus, and lung cancer	High fasting plasma glucose	Percent	2021	0.02103601	0.04736014	-0.0044329
Deaths	Guangxi	Both	All ages	Tracheal, bronchus, and lung cancer	Dietary risks	Percent	2021	0.02695191	0.04159291	0.01374018
Deaths	Sichuan	Both	All ages	Tracheal, bronchus, and lung cancer	Tobacco	Percent	2021	0.65109748	0.85606212	0.30733068
Deaths	Sichuan	Both	All ages	Tracheal, bronchus, and lung cancer	Smoking	Percent	2021	0.6174089	0.83533536	0.31099072
Deaths	Sichuan	Both	All ages	Tracheal, bronchus, and lung cancer	Secondhand smoke	Percent	2021	0.0717692	0.25181756	-0.1015381

Deaths	Sichuan	Both	All ages	Tracheal, bronchus, and lung cancer	Metabolic risks	Percent	2021	0.02004277	0.04501044	-0.0040694
Deaths	Sichuan	Both	All ages	Tracheal, bronchus, and lung cancer	High fasting plasma glucose	Percent	2021	0.02004277	0.04501044	-0.0040694
Deaths	Sichuan	Both	All ages	Tracheal, bronchus, and lung cancer	Dietary risks	Percent	2021	0.02590673	0.03967932	0.01348461
Deaths	Fujian	Both	All ages	Tracheal, bronchus, and lung cancer	Particulate matter pollution	Percent	2021	0.17925396	0.25104217	0.11373004
Deaths	Guangxi	Both	All ages	Tracheal, bronchus, and lung cancer	Diet low in fruits	Percent	2021	0.02695191	0.04159291	0.01374018
Deaths	Sichuan	Both	All ages	Tracheal, bronchus, and lung cancer	Diet low in fruits	Percent	2021	0.02590673	0.03967932	0.01348461
Deaths	Taiwan (Province of China)	Both	All ages	Tracheal, bronchus, and lung cancer	Particulate matter pollution	Percent	2021	0.12616184	0.17716215	0.07912643
Deaths	Anhui	Both	All ages	Tracheal, bronchus, and lung cancer	Tobacco	Percent	2021	0.68869223	0.88891425	0.3256407
Deaths	Anhui	Both	All ages	Tracheal, bronchus, and lung cancer	Smoking	Percent	2021	0.65720025	0.86963253	0.32023192
Deaths	Anhui	Both	All ages	Tracheal, bronchus, and lung cancer	Secondhand smoke	Percent	2021	0.07816167	0.26669763	-0.107624
Deaths	Anhui	Both	All ages	Tracheal, bronchus, and lung cancer	Metabolic risks	Percent	2021	0.02202957	0.04929685	-0.0043793

Deaths	Anhui	Both	All ages	Tracheal, bronchus, and lung cancer	High fasting plasma glucose	Percent	2021	0.02202957	0.04929685	-0.0043793
Deaths	Anhui	Both	All ages	Tracheal, bronchus, and lung cancer	Dietary risks	Percent	2021	0.02605353	0.03984484	0.01292946
Deaths	Ningxia	Both	All ages	Tracheal, bronchus, and lung cancer	Tobacco	Percent	2021	0.59490585	0.82758439	0.24363362
Deaths	Ningxia	Both	All ages	Tracheal, bronchus, and lung cancer	Smoking	Percent	2021	0.55718192	0.7927796	0.24698543
Deaths	Ningxia	Both	All ages	Tracheal, bronchus, and lung cancer	Secondhand smoke	Percent	2021	0.07851981	0.26966465	-0.1001589
Deaths	Ningxia	Both	All ages	Tracheal, bronchus, and lung cancer	Metabolic risks	Percent	2021	0.01854267	0.04130137	-0.0036579
Deaths	Ningxia	Both	All ages	Tracheal, bronchus, and lung cancer	High fasting plasma glucose	Percent	2021	0.01854267	0.04130137	-0.0036579
Deaths	Ningxia	Both	All ages	Tracheal, bronchus, and lung cancer	Dietary risks	Percent	2021	0.02474399	0.03958275	0.01249269
Deaths	Anhui	Both	All ages	Tracheal, bronchus, and lung cancer	Diet low in fruits	Percent	2021	0.02605353	0.03984484	0.01292946
Deaths	Ningxia	Both	All ages	Tracheal, bronchus, and lung cancer	Diet low in fruits	Percent	2021	0.02474399	0.03958275	0.01249269
Deaths	Yunnan	Both	All ages	Tracheal, bronchus, and lung cancer	Residential radon	Percent	2021	0.03569654	0.12065006	-0.0131487

Deaths	Yunnan	Both	All ages	Tracheal, bronchus, and lung cancer	Behavioral risks	Percent	2021	0.67291645	0.84090147	0.37479811
Deaths	Anhui	Both	All ages	Larynx cancer	Tobacco	Percent	2021	0.78514767	0.873031	0.67546124
Deaths	Heilongjiang	Both	All ages	Tracheal, bronchus, and lung cancer	Residential radon	Percent	2021	0.05615894	0.21380614	-0.0276093
Deaths	Heilongjiang	Both	All ages	Tracheal, bronchus, and lung cancer	Behavioral risks	Percent	2021	0.70493607	0.92196899	0.30688005
Deaths	Jiangxi	Both	All ages	Tracheal, bronchus, and lung cancer	Residential radon	Percent	2021	0.04056823	0.16975941	-0.0147847
Deaths	Jiangxi	Both	All ages	Tracheal, bronchus, and lung cancer	Behavioral risks	Percent	2021	0.66116248	0.84911379	0.32795665
Deaths	Liaoning	Both	All ages	Larynx cancer	Occupational exposure to sulfuric acid	Percent	2021	0.03404257	0.06199479	0.01393453
Deaths	Liaoning	Both	All ages	Larynx cancer	All risk factors	Percent	2021	0.81686455	0.89456955	0.71450083
Deaths	Hunan	Both	All ages	Tracheal, bronchus, and lung cancer	Tobacco	Percent	2021	0.69024889	0.8683098	0.34483322
Deaths	Hunan	Both	All ages	Tracheal, bronchus, and lung cancer	Smoking	Percent	2021	0.65881841	0.8490015	0.35036264
Deaths	Hunan	Both	All ages	Tracheal, bronchus, and lung cancer	Secondhand smoke	Percent	2021	0.07464276	0.26179773	-0.1083743

Deaths	Hunan	Both	All ages	Tracheal, bronchus, and lung cancer	Metabolic risks	Percent	2021	0.02025466	0.04446261	-0.0039895
Deaths	Hunan	Both	All ages	Tracheal, bronchus, and lung cancer	High fasting plasma glucose	Percent	2021	0.02025466	0.04446261	-0.0039895
Deaths	Hunan	Both	All ages	Tracheal, bronchus, and lung cancer	Dietary risks	Percent	2021	0.02446175	0.03745803	0.01277558
Deaths	Hunan	Both	All ages	Tracheal, bronchus, and lung cancer	Diet low in fruits	Percent	2021	0.02446175	0.03745803	0.01277558
Deaths	Guizhou	Both	All ages	Tracheal, bronchus, and lung cancer	Tobacco	Percent	2021	0.64858783	0.8484439	0.31103748
Deaths	Guizhou	Both	All ages	Tracheal, bronchus, and lung cancer	Smoking	Percent	2021	0.61253483	0.81186997	0.31844079
Deaths	Guizhou	Both	All ages	Tracheal, bronchus, and lung cancer	Secondhand smoke	Percent	2021	0.07401295	0.24362456	-0.0980891
Deaths	Guizhou	Both	All ages	Tracheal, bronchus, and lung cancer	Metabolic risks	Percent	2021	0.01830641	0.04129076	-0.0036527
Deaths	Guizhou	Both	All ages	Tracheal, bronchus, and lung cancer	High fasting plasma glucose	Percent	2021	0.01830641	0.04129076	-0.0036527
Deaths	Guizhou	Both	All ages	Tracheal, bronchus, and lung cancer	Dietary risks	Percent	2021	0.02838564	0.04414035	0.01483293
Deaths	Tianjin	Both	All ages	Tracheal, bronchus, and lung cancer	Tobacco	Percent	2021	0.65334217	0.88382513	0.2848208

Deaths	Tianjin	Both	All ages	Tracheal, bronchus, and lung cancer	Smoking	Percent	2021	0.62727108	0.87103083	0.27993762
Deaths	Tianjin	Both	All ages	Tracheal, bronchus, and lung cancer	Secondhand smoke	Percent	2021	0.06748767	0.23577628	-0.0888584
Deaths	Tianjin	Both	All ages	Tracheal, bronchus, and lung cancer	Metabolic risks	Percent	2021	0.02610876	0.05783258	-0.0053243
Deaths	Tianjin	Both	All ages	Tracheal, bronchus, and lung cancer	High fasting plasma glucose	Percent	2021	0.02610876	0.05783258	-0.0053243
Deaths	Tianjin	Both	All ages	Tracheal, bronchus, and lung cancer	Dietary risks	Percent	2021	0.01685222	0.02629936	0.00835459
Deaths	Guizhou	Both	All ages	Tracheal, bronchus, and lung cancer	Diet low in fruits	Percent	2021	0.02838564	0.04414035	0.01483293
Deaths	Tianjin	Both	All ages	Tracheal, bronchus, and lung cancer	Diet low in fruits	Percent	2021	0.01685222	0.02629936	0.00835459
Deaths	Jilin	Both	All ages	Tracheal, bronchus, and lung cancer	Air pollution	Percent	2021	0.24325571	0.33739643	0.15705726
Deaths	Jilin	Both	All ages	Tracheal, bronchus, and lung cancer	Ambient particulate matter pollution	Percent	2021	0.20386787	0.2883689	0.11181145
Deaths	Jilin	Both	All ages	Tracheal, bronchus, and lung cancer	Household air pollution from solid fuels	Percent	2021	0.03916151	0.167811	0.00192589
Deaths	Jilin	Both	All ages	Tracheal, bronchus, and lung cancer	Other environmental risks	Percent	2021	0.05015807	0.18689905	-0.0193016

Deaths	Beijing	Both	All ages	Tracheal, bronchus, and lung cancer	Tobacco	Percent	2021	0.6454764 8	0.8709427 8	0.2835799 4
Deaths	Beijing	Both	All ages	Tracheal, bronchus, and lung cancer	Smoking	Percent	2021	0.6127903 3	0.8551619 7	0.2862153 6
Deaths	Beijing	Both	All ages	Tracheal, bronchus, and lung cancer	Secondhand smoke	Percent	2021	0.0746926 6	0.2637757 6	-0.1003524
Deaths	Beijing	Both	All ages	Tracheal, bronchus, and lung cancer	Metabolic risks	Percent	2021	0.0229422 2	0.0516600 5	-0.0045427
Deaths	Beijing	Both	All ages	Tracheal, bronchus, and lung cancer	High fasting plasma glucose	Percent	2021	0.0229422 2	0.0516600 5	-0.0045427
Deaths	Beijing	Both	All ages	Tracheal, bronchus, and lung cancer	Dietary risks	Percent	2021	0.0176116 7	0.0267913 1	0.0086679 8
Deaths	Beijing	Both	All ages	Tracheal, bronchus, and lung cancer	Diet low in fruits	Percent	2021	0.0176116 7	0.0267913 1	0.0086679 8
Deaths	Hong Kong Special Administrative Region of China	Both	All ages	Tracheal, bronchus, and lung cancer	Tobacco	Percent	2021	0.4432857 5	0.7104326 8	0.1290359 7
Deaths	Hong Kong Special Administrative Region of China	Both	All ages	Tracheal, bronchus, and lung cancer	Smoking	Percent	2021	0.4154860 9	0.7006099 6	0.1340498 9
Deaths	Hong Kong Special Administrative Region of China	Both	All ages	Tracheal, bronchus, and lung cancer	Secondhand smoke	Percent	2021	0.0525426 9	0.1848738 4	-0.0609173
Deaths	Hong Kong Special Administrative Region of China	Both	All ages	Tracheal, bronchus, and lung cancer	Metabolic risks	Percent	2021	0.0178116 7	0.0409540 5	-0.003534

Deaths	Hong Kong Special Administrative Region of China	Both	All ages	Tracheal, bronchus, and lung cancer	High fasting plasma glucose	Percent	2021	0.01781167	0.04095405	-0.003534
Deaths	Hong Kong Special Administrative Region of China	Both	All ages	Tracheal, bronchus, and lung cancer	Dietary risks	Percent	2021	0.02699597	0.04187197	0.01366216
Deaths	Hong Kong Special Administrative Region of China	Both	All ages	Tracheal, bronchus, and lung cancer	Diet low in fruits	Percent	2021	0.02699597	0.04187197	0.01366216
Deaths	Shanghai	Both	All ages	Tracheal, bronchus, and lung cancer	Residential radon	Percent	2021	0.03319356	0.13476499	-0.0127658
Deaths	Shanghai	Both	All ages	Tracheal, bronchus, and lung cancer	Behavioral risks	Percent	2021	0.59694642	0.80882347	0.26793059
Deaths	Shandong	Both	All ages	Tracheal, bronchus, and lung cancer	Particulate matter pollution	Percent	2021	0.28761372	0.37883997	0.19030699
Deaths	Hubei	Both	All ages	Larynx cancer	Tobacco	Percent	2021	0.756495	0.84379595	0.6402704
Deaths	Heilongjiang	Both	All ages	Larynx cancer	Smoking	Percent	2021	0.79994533	0.88952361	0.67282311
Deaths	Heilongjiang	Both	All ages	Larynx cancer	High alcohol use	Percent	2021	0.15157142	0.2250183	0.07754219
Deaths	Heilongjiang	Both	All ages	Larynx cancer	Occupational risks	Percent	2021	0.04720433	0.07690137	0.02620239
Deaths	Heilongjiang	Both	All ages	Larynx cancer	Occupational carcinogens	Percent	2021	0.04720433	0.07690137	0.02620239

Deaths	Heilongjiang	Both	All ages	Larynx cancer	Occupational exposure to asbestos	Percent	2021	0.01311531	0.02128396	0.00721477
Deaths	Hubei	Both	All ages	Tracheal, bronchus, and lung cancer	Air pollution	Percent	2021	0.27304325	0.36253861	0.17738818
Deaths	Hubei	Both	All ages	Tracheal, bronchus, and lung cancer	Ambient particulate matter pollution	Percent	2021	0.24563587	0.33610165	0.14854353
Deaths	Hubei	Both	All ages	Tracheal, bronchus, and lung cancer	Household air pollution from solid fuels	Percent	2021	0.0272625	0.13632972	0.00113342
Deaths	Hubei	Both	All ages	Tracheal, bronchus, and lung cancer	Other environmental risks	Percent	2021	0.03295757	0.12459061	-0.0127428
Deaths	Inner Mongolia	Both	All ages	Larynx cancer	Tobacco	Percent	2021	0.80330814	0.887627	0.68948139
Deaths	Heilongjiang	Both	All ages	Tracheal, bronchus, and lung cancer	Occupational risks	Percent	2021	0.08489913	0.11082761	0.0619275
Deaths	Heilongjiang	Both	All ages	Tracheal, bronchus, and lung cancer	Occupational carcinogens	Percent	2021	0.08489913	0.11082761	0.0619275
Deaths	Heilongjiang	Both	All ages	Tracheal, bronchus, and lung cancer	Occupational exposure to asbestos	Percent	2021	0.03995137	0.05864786	0.0239005
Deaths	Heilongjiang	Both	All ages	Tracheal, bronchus, and lung cancer	Occupational exposure to arsenic	Percent	2021	0.00487463	0.00804394	0.00171306
Deaths	Heilongjiang	Both	All ages	Tracheal, bronchus, and lung cancer	Occupational exposure to beryllium	Percent	2021	0.0001769	0.00021041	0.00014591

Deaths	Heilongjiang	Both	All ages	Tracheal, bronchus, and lung cancer	Occupational exposure to cadmium	Percent	2021	0.0004749	0.0057506	0.0037662
Deaths	Jiangsu	Both	All ages	Tracheal, bronchus, and lung cancer	Particulate matter pollution	Percent	2021	0.26367309	0.3504463	0.17508913
Deaths	Yunnan	Both	All ages	Tracheal, bronchus, and lung cancer	Particulate matter pollution	Percent	2021	0.23781839	0.33610364	0.14899265
Deaths	Qinghai	Both	All ages	Tracheal, bronchus, and lung cancer	Tobacco	Percent	2021	0.59470802	0.79648511	0.25460871
Deaths	Qinghai	Both	All ages	Tracheal, bronchus, and lung cancer	Smoking	Percent	2021	0.54488809	0.75102238	0.26954511
Deaths	Qinghai	Both	All ages	Tracheal, bronchus, and lung cancer	Secondhand smoke	Percent	2021	0.09200012	0.31287692	-0.1458284
Deaths	Qinghai	Both	All ages	Tracheal, bronchus, and lung cancer	Metabolic risks	Percent	2021	0.01874149	0.04306224	-0.0037835
Deaths	Qinghai	Both	All ages	Tracheal, bronchus, and lung cancer	High fasting plasma glucose	Percent	2021	0.01874149	0.04306224	-0.0037835
Deaths	Qinghai	Both	All ages	Tracheal, bronchus, and lung cancer	Dietary risks	Percent	2021	0.02572811	0.0383305	0.01348327
Deaths	Qinghai	Both	All ages	Tracheal, bronchus, and lung cancer	Diet low in fruits	Percent	2021	0.02572811	0.0383305	0.01348327
Deaths	Zhejiang	Both	All ages	Tracheal, bronchus, and lung cancer	Air pollution	Percent	2021	0.21891974	0.29433975	0.14294645

Deaths	Zhejiang	Both	All ages	Tracheal, bronchus, and lung cancer	Ambient particulate matter pollution	Percent	2021	0.21215745	0.28768334	0.13839113
Deaths	Zhejiang	Both	All ages	Tracheal, bronchus, and lung cancer	Household air pollution from solid fuels	Percent	2021	0.0066917	0.05521067	3.01E-05
Deaths	Zhejiang	Both	All ages	Tracheal, bronchus, and lung cancer	Other environmental risks	Percent	2021	0.0285092	0.10541737	-0.0107338
Deaths	Heilongjiang	Both	All ages	Tracheal, bronchus, and lung cancer	Particulate matter pollution	Percent	2021	0.24007644	0.33364925	0.15125097
Deaths	Guangdong	Both	All ages	Tracheal, bronchus, and lung cancer	Air pollution	Percent	2021	0.19617267	0.26672833	0.12640139
Deaths	Guangdong	Both	All ages	Tracheal, bronchus, and lung cancer	Ambient particulate matter pollution	Percent	2021	0.18610976	0.2558157	0.11828974
Deaths	Guangdong	Both	All ages	Tracheal, bronchus, and lung cancer	Household air pollution from solid fuels	Percent	2021	0.00998675	0.07235594	0.0001156
Deaths	Guangdong	Both	All ages	Tracheal, bronchus, and lung cancer	Other environmental risks	Percent	2021	0.03642885	0.13905689	-0.0156484
Deaths	Shanxi	Both	All ages	Tracheal, bronchus, and lung cancer	Air pollution	Percent	2021	0.30094881	0.40139859	0.19590799
Deaths	Shanxi	Both	All ages	Tracheal, bronchus, and lung cancer	Ambient particulate matter pollution	Percent	2021	0.24144915	0.34029184	0.13766526
Deaths	Shanxi	Both	All ages	Tracheal, bronchus, and lung cancer	Household air pollution from solid fuels	Percent	2021	0.05934859	0.18364754	0.00794779

Deaths	Shanxi	Both	All ages	Tracheal, bronchus, and lung cancer	Other environmental risks	Percent	2021	0.0363953	0.14989316	-0.0129276
Deaths	Guangdong	Both	All ages	Larynx cancer	Occupational exposure to sulfuric acid	Percent	2021	0.04054614	0.07477192	0.01615543
Deaths	Guangdong	Both	All ages	Larynx cancer	All risk factors	Percent	2021	0.82560245	0.89626086	0.73565047
Deaths	Gansu	Both	All ages	Tracheal, bronchus, and lung cancer	Residential radon	Percent	2021	0.09760937	0.30118111	-0.0532428
Deaths	Gansu	Both	All ages	Tracheal, bronchus, and lung cancer	Behavioral risks	Percent	2021	0.66490091	0.84633882	0.34136739
Deaths	Jiangxi	Both	All ages	Tracheal, bronchus, and lung cancer	Particulate matter pollution	Percent	2021	0.25388975	0.35273638	0.16353345
Deaths	Hubei	Both	All ages	Larynx cancer	Occupational exposure to sulfuric acid	Percent	2021	0.03817725	0.06963587	0.01603433
Deaths	Hubei	Both	All ages	Larynx cancer	All risk factors	Percent	2021	0.78944766	0.8645247	0.69561303
Deaths	Beijing	Both	All ages	Larynx cancer	Tobacco	Percent	2021	0.78502389	0.87940453	0.65961407
Deaths	Ningxia	Both	All ages	Larynx cancer	Tobacco	Percent	2021	0.69692323	0.80230191	0.56531597
Deaths	Tianjin	Both	All ages	Larynx cancer	Tobacco	Percent	2021	0.78133099	0.86958502	0.66346733

Deaths	Sichuan	Both	All ages	Larynx cancer	Tobacco	Percent	2021	0.78017633	0.86767106	0.67243747
Deaths	Jiangxi	Both	All ages	Larynx cancer	Smoking	Percent	2021	0.74125995	0.84605905	0.62904055
Deaths	Jiangxi	Both	All ages	Larynx cancer	High alcohol use	Percent	2021	0.14001646	0.21319109	0.06898192
Deaths	Jiangxi	Both	All ages	Larynx cancer	Occupational risks	Percent	2021	0.04965479	0.08529771	0.0267392
Deaths	Jiangxi	Both	All ages	Larynx cancer	Occupational carcinogens	Percent	2021	0.04965479	0.08529771	0.0267392
Deaths	Jiangxi	Both	All ages	Larynx cancer	Occupational exposure to asbestos	Percent	2021	0.00996219	0.01643082	0.00521081
Deaths	Guangdong	Both	All ages	Larynx cancer	Tobacco	Percent	2021	0.79384537	0.87392673	0.68741746
Deaths	Sichuan	Both	All ages	Larynx cancer	Occupational exposure to sulfuric acid	Percent	2021	0.04326403	0.07905435	0.01805264
Deaths	Sichuan	Both	All ages	Larynx cancer	All risk factors	Percent	2021	0.81139405	0.88697345	0.71671705
Deaths	Hong Kong Special Administrative Region of China	Both	All ages	Larynx cancer	Tobacco	Percent	2021	0.61587625	0.76823241	0.44427086
Deaths	Shanghai	Both	All ages	Tracheal, bronchus, and lung cancer	Particulate matter pollution	Percent	2021	0.23052976	0.30877298	0.15127252

Deaths	Hubei	Both	All ages	Tracheal, bronchus, and lung cancer	Residential radon	Percent	2021	0.03295757	0.12459061	-0.0127428
Deaths	Hubei	Both	All ages	Tracheal, bronchus, and lung cancer	Behavioral risks	Percent	2021	0.64752118	0.82429202	0.3228297
Deaths	Henan	Both	All ages	Tracheal, bronchus, and lung cancer	Air pollution	Percent	2021	0.31494374	0.4118977	0.20767588
Deaths	Henan	Both	All ages	Tracheal, bronchus, and lung cancer	Ambient particulate matter pollution	Percent	2021	0.26021444	0.36291671	0.14898094
Deaths	Henan	Both	All ages	Tracheal, bronchus, and lung cancer	Household air pollution from solid fuels	Percent	2021	0.05459786	0.18560687	0.00666915
Deaths	Henan	Both	All ages	Tracheal, bronchus, and lung cancer	Other environmental risks	Percent	2021	0.03103388	0.12950577	-0.0116725
Deaths	Yunnan	Both	All ages	Larynx cancer	Smoking	Percent	2021	0.76661661	0.8471209	0.67278868
Deaths	Yunnan	Both	All ages	Larynx cancer	High alcohol use	Percent	2021	0.13099104	0.20833467	0.05890475
Deaths	Yunnan	Both	All ages	Larynx cancer	Occupational risks	Percent	2021	0.0632219	0.1045178	0.03536756
Deaths	Yunnan	Both	All ages	Larynx cancer	Occupational carcinogens	Percent	2021	0.0632219	0.1045178	0.03536756
Deaths	Yunnan	Both	All ages	Larynx cancer	Occupational exposure to asbestos	Percent	2021	0.01754825	0.02734672	0.00952762

Deaths	Jiangxi	Both	All ages	Tracheal, bronchus, and lung cancer	Occupational risks	Percent	2021	0.08830024	0.11673323	0.06462454
Deaths	Jiangxi	Both	All ages	Tracheal, bronchus, and lung cancer	Occupational carcinogens	Percent	2021	0.08830024	0.11673323	0.06462454
Deaths	Jiangxi	Both	All ages	Tracheal, bronchus, and lung cancer	Occupational exposure to asbestos	Percent	2021	0.02902601	0.04478872	0.0169162
Deaths	Jiangxi	Both	All ages	Tracheal, bronchus, and lung cancer	Occupational exposure to arsenic	Percent	2021	0.00644638	0.01072597	0.00234492
Deaths	Jiangxi	Both	All ages	Tracheal, bronchus, and lung cancer	Occupational exposure to beryllium	Percent	2021	0.00022309	0.00026799	0.00018035
Deaths	Jiangxi	Both	All ages	Tracheal, bronchus, and lung cancer	Occupational exposure to cadmium	Percent	2021	0.00062492	0.00076371	0.00049557
Deaths	Yunnan	Both	All ages	Tracheal, bronchus, and lung cancer	Occupational risks	Percent	2021	0.11815003	0.15132724	0.08557119
Deaths	Yunnan	Both	All ages	Tracheal, bronchus, and lung cancer	Occupational carcinogens	Percent	2021	0.11815003	0.15132724	0.08557119
Deaths	Yunnan	Both	All ages	Tracheal, bronchus, and lung cancer	Occupational exposure to asbestos	Percent	2021	0.05018401	0.07374047	0.02990082
Deaths	Yunnan	Both	All ages	Tracheal, bronchus, and lung cancer	Occupational exposure to arsenic	Percent	2021	0.00768399	0.01278169	0.00286711
Deaths	Yunnan	Both	All ages	Tracheal, bronchus, and lung cancer	Occupational exposure to beryllium	Percent	2021	0.00027324	0.00032464	0.00022675

Deaths	Yunnan	Both	All ages	Tracheal, bronchus, and lung cancer	Occupational exposure to cadmium	Percent	2021	0.00074586	0.00089903	0.00061344
Deaths	Jilin	Both	All ages	Tracheal, bronchus, and lung cancer	Residential radon	Percent	2021	0.05015807	0.18689905	-0.0193016
Deaths	Jilin	Both	All ages	Tracheal, bronchus, and lung cancer	Behavioral risks	Percent	2021	0.62067982	0.84781124	0.26047356
Deaths	Liaoning	Both	All ages	Tracheal, bronchus, and lung cancer	Air pollution	Percent	2021	0.25112666	0.33723886	0.16280071
Deaths	Liaoning	Both	All ages	Tracheal, bronchus, and lung cancer	Ambient particulate matter pollution	Percent	2021	0.23062516	0.31498491	0.14016118
Deaths	Liaoning	Both	All ages	Tracheal, bronchus, and lung cancer	Household air pollution from solid fuels	Percent	2021	0.02040113	0.11510435	0.00500015
Deaths	Liaoning	Both	All ages	Tracheal, bronchus, and lung cancer	Other environmental risks	Percent	2021	0.04768975	0.209007	-0.0160991
Deaths	Gansu	Both	All ages	Larynx cancer	Smoking	Percent	2021	0.75001195	0.83575858	0.63231912
Deaths	Gansu	Both	All ages	Larynx cancer	High alcohol use	Percent	2021	0.09891432	0.15403483	0.04738189
Deaths	Gansu	Both	All ages	Larynx cancer	Occupational risks	Percent	2021	0.05676745	0.09306269	0.03150311
Deaths	Gansu	Both	All ages	Larynx cancer	Occupational carcinogens	Percent	2021	0.05676745	0.09306269	0.03150311

Deaths	Gansu	Both	All ages	Larynx cancer	Occupational exposure to asbestos	Percent	2021	0.01503461	0.02442079	0.00804148
Deaths	Hunan	Both	All ages	Larynx cancer	Tobacco	Percent	2021	0.7857865	0.87718987	0.6690177
Deaths	Guangxi	Both	All ages	Larynx cancer	Tobacco	Percent	2021	0.73097126	0.83594488	0.61332815
Deaths	Zhejiang	Both	All ages	Larynx cancer	Smoking	Percent	2021	0.79805008	0.87743864	0.68328235
Deaths	Zhejiang	Both	All ages	Larynx cancer	High alcohol use	Percent	2021	0.1747541	0.25699728	0.0937643
Deaths	Zhejiang	Both	All ages	Larynx cancer	Occupational risks	Percent	2021	0.04662942	0.08036001	0.02384476
Deaths	Zhejiang	Both	All ages	Larynx cancer	Occupational carcinogens	Percent	2021	0.04662942	0.08036001	0.02384476
Deaths	Zhejiang	Both	All ages	Larynx cancer	Occupational exposure to asbestos	Percent	2021	0.00713226	0.01259458	0.00350434
Deaths	Gansu	Both	All ages	Tracheal, bronchus, and lung cancer	Occupational risks	Percent	2021	0.10419707	0.13679603	0.07532133
Deaths	Gansu	Both	All ages	Tracheal, bronchus, and lung cancer	Occupational carcinogens	Percent	2021	0.10419707	0.13679603	0.07532133
Deaths	Gansu	Both	All ages	Tracheal, bronchus, and lung cancer	Occupational exposure to asbestos	Percent	2021	0.04353009	0.06455638	0.02659715

Deaths	Gansu	Both	All ages	Tracheal, bronchus, and lung cancer	Occupational exposure to arsenic	Percent	2021	0.00677517	0.01133152	0.00241607
Deaths	Gansu	Both	All ages	Tracheal, bronchus, and lung cancer	Occupational exposure to beryllium	Percent	2021	0.00024895	0.00029705	0.0002066
Deaths	Zhejiang	Both	All ages	Tracheal, bronchus, and lung cancer	Occupational risks	Percent	2021	0.08145389	0.10856208	0.05903291
Deaths	Zhejiang	Both	All ages	Tracheal, bronchus, and lung cancer	Occupational carcinogens	Percent	2021	0.08145389	0.10856208	0.05903291
Deaths	Zhejiang	Both	All ages	Tracheal, bronchus, and lung cancer	Occupational exposure to asbestos	Percent	2021	0.02076976	0.03139854	0.01212193
Deaths	Zhejiang	Both	All ages	Tracheal, bronchus, and lung cancer	Occupational exposure to arsenic	Percent	2021	0.00646631	0.01067506	0.0023872
Deaths	Zhejiang	Both	All ages	Tracheal, bronchus, and lung cancer	Occupational exposure to beryllium	Percent	2021	0.00021461	0.0002594	0.00017366
Deaths	Zhejiang	Both	All ages	Tracheal, bronchus, and lung cancer	Occupational exposure to cadmium	Percent	2021	0.00062822	0.00078485	0.00050105
Deaths	Gansu	Both	All ages	Tracheal, bronchus, and lung cancer	Occupational exposure to cadmium	Percent	2021	0.00065856	0.00080234	0.00054446
Deaths	Zhejiang	Both	All ages	Tracheal, bronchus, and lung cancer	Residential radon	Percent	2021	0.0285092	0.10541737	-0.0107338
Deaths	Zhejiang	Both	All ages	Tracheal, bronchus, and lung cancer	Behavioral risks	Percent	2021	0.66553725	0.85791474	0.34006633

Deaths	Henan	Both	All ages	Tracheal, bronchus, and lung cancer	Residential radon	Percent	2021	0.03103388	0.12950577	-0.0116725
Deaths	Henan	Both	All ages	Tracheal, bronchus, and lung cancer	Behavioral risks	Percent	2021	0.61774785	0.82997691	0.27277711
Deaths	Gansu	Both	All ages	Tracheal, bronchus, and lung cancer	Particulate matter pollution	Percent	2021	0.28453332	0.39021634	0.18514778
Deaths	Shanxi	Both	All ages	Tracheal, bronchus, and lung cancer	Residential radon	Percent	2021	0.0363953	0.14989316	-0.0129276
Deaths	Shanxi	Both	All ages	Tracheal, bronchus, and lung cancer	Behavioral risks	Percent	2021	0.68542353	0.88219526	0.3401455
Deaths	Guangdong	Both	All ages	Tracheal, bronchus, and lung cancer	Residential radon	Percent	2021	0.03642885	0.13905689	-0.0156484
Deaths	Guangdong	Both	All ages	Tracheal, bronchus, and lung cancer	Behavioral risks	Percent	2021	0.67134567	0.86582657	0.32882211
Deaths	China	Both	All ages	Larynx cancer	All risk factors	Percent	2021	0.80012349	0.87051487	0.71023748
Deaths	Liaoning	Both	All ages	Tracheal, bronchus, and lung cancer	Residential radon	Percent	2021	0.04768975	0.209007	-0.0160991
Deaths	Liaoning	Both	All ages	Tracheal, bronchus, and lung cancer	Behavioral risks	Percent	2021	0.66110575	0.88213245	0.29472473
Deaths	Henan	Both	All ages	Larynx cancer	Environmental/occupational risks	Percent	2021	0.05358665	0.0897132	0.02782449

Deaths	Henan	Both	All ages	Larynx cancer	Behavioral risks	Percent	2021	0.72783591	0.82648104	0.60943272
Deaths	Henan	Both	All ages	Tracheal, bronchus, and lung cancer	Occupational exposure to chromium	Percent	2021	0.00143973	0.00168789	0.0012215
Deaths	Henan	Both	All ages	Tracheal, bronchus, and lung cancer	Occupational exposure to diesel engine exhaust	Percent	2021	0.01533179	0.01798563	0.01310842
Deaths	Henan	Both	All ages	Tracheal, bronchus, and lung cancer	Occupational exposure to nickel	Percent	2021	0.006697	0.01478127	0.00140605
Deaths	Henan	Both	All ages	Tracheal, bronchus, and lung cancer	Occupational exposure to polycyclic aromatic hydrocarbons	Percent	2021	0.00488325	0.00588474	0.00402372
Deaths	Henan	Both	All ages	Tracheal, bronchus, and lung cancer	Occupational exposure to silica	Percent	2021	0.03110243	0.04852209	0.01442287
Deaths	Henan	Both	All ages	Tracheal, bronchus, and lung cancer	All risk factors	Percent	2021	0.7754343	0.90605141	0.56653464
Deaths	Henan	Both	All ages	Tracheal, bronchus, and lung cancer	Environmental/occupational risks	Percent	2021	0.39805594	0.49034234	0.29629938
Deaths	China	Both	All ages	Larynx cancer	Tobacco	Percent	2021	0.76699656	0.85149558	0.6545722
Deaths	China	Both	All ages	Larynx cancer	Smoking	Percent	2021	0.76699656	0.85149558	0.6545722
Deaths	Jilin	Both	All ages	Larynx cancer	Smoking	Percent	2021	0.73750239	0.84910498	0.59927356

Deaths	Jilin	Both	All ages	Larynx cancer	High alcohol use	Percent	2021	0.13187926	0.20671963	0.06654823
Deaths	Jilin	Both	All ages	Larynx cancer	Occupational risks	Percent	2021	0.05312401	0.08389695	0.03085885
Deaths	Jilin	Both	All ages	Larynx cancer	Occupational carcinogens	Percent	2021	0.05312401	0.08389695	0.03085885
Deaths	Jilin	Both	All ages	Larynx cancer	Occupational exposure to asbestos	Percent	2021	0.01844321	0.02848044	0.00988551
Deaths	Hubei	Both	All ages	Tracheal, bronchus, and lung cancer	Particulate matter pollution	Percent	2021	0.27304325	0.36253861	0.17738818
Deaths	Liaoning	Both	All ages	Larynx cancer	Smoking	Percent	2021	0.7824864	0.87505561	0.66197686
Deaths	Liaoning	Both	All ages	Larynx cancer	High alcohol use	Percent	2021	0.15389504	0.22649453	0.08273476
Deaths	Liaoning	Both	All ages	Larynx cancer	Occupational risks	Percent	2021	0.05240321	0.08380891	0.02986207
Deaths	Liaoning	Both	All ages	Larynx cancer	Occupational carcinogens	Percent	2021	0.05240321	0.08380891	0.02986207
Deaths	Liaoning	Both	All ages	Larynx cancer	Occupational exposure to asbestos	Percent	2021	0.01875432	0.03126082	0.00961867
Deaths	Henan	Both	All ages	Larynx cancer	Smoking	Percent	2021	0.70693863	0.81229831	0.57380863

Deaths	Henan	Both	All ages	Larynx cancer	High alcohol use	Percent	2021	0.12441782	0.18829543	0.06149975
Deaths	Henan	Both	All ages	Larynx cancer	Occupational risks	Percent	2021	0.05358665	0.0897132	0.02782449
Deaths	Henan	Both	All ages	Larynx cancer	Occupational carcinogens	Percent	2021	0.05358665	0.0897132	0.02782449
Deaths	Henan	Both	All ages	Larynx cancer	Occupational exposure to asbestos	Percent	2021	0.00939714	0.01570448	0.00516989
Deaths	Guangxi	Both	All ages	Tracheal, bronchus, and lung cancer	Air pollution	Percent	2021	0.25411874	0.35132244	0.16405532
Deaths	Guangxi	Both	All ages	Tracheal, bronchus, and lung cancer	Ambient particulate matter pollution	Percent	2021	0.17125017	0.25565344	0.08506805
Deaths	Guangxi	Both	All ages	Tracheal, bronchus, and lung cancer	Household air pollution from solid fuels	Percent	2021	0.08279365	0.21468699	0.01492416
Deaths	Guangxi	Both	All ages	Tracheal, bronchus, and lung cancer	Other environmental risks	Percent	2021	0.03097751	0.11909514	-0.0108217
Deaths	Anhui	Both	All ages	Tracheal, bronchus, and lung cancer	Air pollution	Percent	2021	0.29641444	0.39358999	0.19606488
Deaths	Anhui	Both	All ages	Tracheal, bronchus, and lung cancer	Ambient particulate matter pollution	Percent	2021	0.22681284	0.32407304	0.11907872
Deaths	Anhui	Both	All ages	Tracheal, bronchus, and lung cancer	Household air pollution from solid fuels	Percent	2021	0.06947684	0.19719853	0.00973207

Deaths	Anhui	Both	All ages	Tracheal, bronchus, and lung cancer	Other environmental risks	Percent	2021	0.02994422	0.10747248	-0.0120123
Deaths	Inner Mongolia	Both	All ages	Larynx cancer	Smoking	Percent	2021	0.80330814	0.887627	0.68948139
Deaths	Inner Mongolia	Both	All ages	Larynx cancer	High alcohol use	Percent	2021	0.13968604	0.20891632	0.07198725
Deaths	Inner Mongolia	Both	All ages	Larynx cancer	Occupational risks	Percent	2021	0.05444686	0.08824743	0.03113779
Deaths	Inner Mongolia	Both	All ages	Larynx cancer	Occupational carcinogens	Percent	2021	0.05444686	0.08824743	0.03113779
Deaths	Inner Mongolia	Both	All ages	Larynx cancer	Occupational exposure to asbestos	Percent	2021	0.01638043	0.02561086	0.0089899
Deaths	Jilin	Both	All ages	Tracheal, bronchus, and lung cancer	Particulate matter pollution	Percent	2021	0.24325571	0.33739643	0.15705726
Deaths	Ningxia	Both	All ages	Tracheal, bronchus, and lung cancer	Air pollution	Percent	2021	0.24748086	0.33727528	0.15703205
Deaths	Ningxia	Both	All ages	Tracheal, bronchus, and lung cancer	Ambient particulate matter pollution	Percent	2021	0.20539485	0.29037308	0.11474823
Deaths	Ningxia	Both	All ages	Tracheal, bronchus, and lung cancer	Household air pollution from solid fuels	Percent	2021	0.04194982	0.16405401	0.0030732
Deaths	Ningxia	Both	All ages	Tracheal, bronchus, and lung cancer	Other environmental risks	Percent	2021	0.04588923	0.16947931	-0.0182679

Deaths	Hong Kong Special Administrative Region of China	Both	All ages	Larynx cancer	Occupational exposure to sulfuric acid	Percent	2021	0.03154891	0.05835145	0.01312087
Deaths	Hong Kong Special Administrative Region of China	Both	All ages	Larynx cancer	All risk factors	Percent	2021	0.68805966	0.81411289	0.54463015
Deaths	Shanxi	Both	All ages	Tracheal, bronchus, and lung cancer	Particulate matter pollution	Percent	2021	0.30094881	0.40139859	0.19590799
Deaths	Liaoning	Both	All ages	Tracheal, bronchus, and lung cancer	Particulate matter pollution	Percent	2021	0.25112666	0.33723886	0.16280071
Deaths	Shanxi	Both	All ages	Larynx cancer	Smoking	Percent	2021	0.77194575	0.86363437	0.65803726
Deaths	Shanxi	Both	All ages	Larynx cancer	High alcohol use	Percent	2021	0.12398012	0.18725886	0.06071821
Deaths	Shanxi	Both	All ages	Larynx cancer	Occupational risks	Percent	2021	0.0519816	0.08577062	0.02850447
Deaths	Shanxi	Both	All ages	Larynx cancer	Occupational carcinogens	Percent	2021	0.0519816	0.08577062	0.02850447
Deaths	Shanxi	Both	All ages	Larynx cancer	Occupational exposure to asbestos	Percent	2021	0.0123635	0.01948388	0.00675296
Deaths	Shanghai	Both	All ages	Larynx cancer	Smoking	Percent	2021	0.77858562	0.87675068	0.65781808
Deaths	Shanghai	Both	All ages	Larynx cancer	High alcohol use	Percent	2021	0.17000513	0.2437403	0.08767615

Deaths	Shanghai	Both	All ages	Larynx cancer	Occupational risks	Percent	2021	0.03810634	0.06233847	0.02117258
Deaths	Shanghai	Both	All ages	Larynx cancer	Occupational carcinogens	Percent	2021	0.03810634	0.06233847	0.02117258
Deaths	Shanghai	Both	All ages	Larynx cancer	Occupational exposure to asbestos	Percent	2021	0.01146651	0.02056574	0.00565683
Deaths	Henan	Both	All ages	Tracheal, bronchus, and lung cancer	Particulate matter pollution	Percent	2021	0.31494374	0.4118977	0.20767588
Deaths	Guizhou	Both	All ages	Larynx cancer	Tobacco	Percent	2021	0.75846921	0.86980277	0.62631247
Deaths	Zhejiang	Both	All ages	Tracheal, bronchus, and lung cancer	Occupational exposure to chromium	Percent	2021	0.00135277	0.00156682	0.00113784
Deaths	Gansu	Both	All ages	Larynx cancer	Environmental/occupational risks	Percent	2021	0.05676745	0.09306269	0.03150311
Deaths	Gansu	Both	All ages	Larynx cancer	Behavioral risks	Percent	2021	0.76249279	0.84378446	0.64526612
Deaths	Gansu	Both	All ages	Tracheal, bronchus, and lung cancer	Occupational exposure to chromium	Percent	2021	0.0013481	0.0016015	0.0011429
Deaths	Qinghai	Both	All ages	Larynx cancer	Occupational exposure to sulfuric acid	Percent	2021	0.04271514	0.07952183	0.01807938
Deaths	Qinghai	Both	All ages	Larynx cancer	All risk factors	Percent	2021	0.71877776	0.82005311	0.6042711

Deaths	Jilin	Both	All ages	Tracheal, bronchus, and lung cancer	Occupational risks	Percent	2021	0.10211634	0.130463	0.07586299
Deaths	Jilin	Both	All ages	Tracheal, bronchus, and lung cancer	Occupational carcinogens	Percent	2021	0.10211634	0.130463	0.07586299
Deaths	Jilin	Both	All ages	Tracheal, bronchus, and lung cancer	Occupational exposure to asbestos	Percent	2021	0.05155314	0.07322461	0.03272245
Deaths	Jilin	Both	All ages	Tracheal, bronchus, and lung cancer	Occupational exposure to arsenic	Percent	2021	0.00561048	0.00932522	0.00216842
Deaths	Jilin	Both	All ages	Tracheal, bronchus, and lung cancer	Occupational exposure to beryllium	Percent	2021	0.00020248	0.00024245	0.00016735
Deaths	Jilin	Both	All ages	Tracheal, bronchus, and lung cancer	Occupational exposure to cadmium	Percent	2021	0.00054791	0.00067731	0.00043785
Deaths	Gansu	Both	All ages	Tracheal, bronchus, and lung cancer	Occupational exposure to diesel engine exhaust	Percent	2021	0.01358217	0.01585398	0.01172277
Deaths	Gansu	Both	All ages	Tracheal, bronchus, and lung cancer	Occupational exposure to nickel	Percent	2021	0.00660494	0.01503013	0.00139342
Deaths	Gansu	Both	All ages	Tracheal, bronchus, and lung cancer	Occupational exposure to polycyclic aromatic hydrocarbons	Percent	2021	0.0045876	0.0054662	0.00375864
Deaths	Gansu	Both	All ages	Tracheal, bronchus, and lung cancer	Occupational exposure to silica	Percent	2021	0.03061951	0.04795884	0.0140885
Deaths	Gansu	Both	All ages	Tracheal, bronchus, and lung cancer	All risk factors	Percent	2021	0.81069989	0.91940756	0.61755904

Deaths	Henan	Both	All ages	Tracheal, bronchus, and lung cancer	Occupational risks	Percent	2021	0.09313532	0.12295667	0.0678494
Deaths	Henan	Both	All ages	Tracheal, bronchus, and lung cancer	Occupational carcinogens	Percent	2021	0.09313532	0.12295667	0.0678494
Deaths	Henan	Both	All ages	Tracheal, bronchus, and lung cancer	Occupational exposure to asbestos	Percent	2021	0.02916864	0.04314601	0.01712855
Deaths	Henan	Both	All ages	Tracheal, bronchus, and lung cancer	Occupational exposure to arsenic	Percent	2021	0.00698967	0.01130837	0.00282106
Deaths	Henan	Both	All ages	Tracheal, bronchus, and lung cancer	Occupational exposure to beryllium	Percent	2021	0.00024687	0.00029786	0.00019848
Deaths	Henan	Both	All ages	Tracheal, bronchus, and lung cancer	Occupational exposure to cadmium	Percent	2021	0.00068138	0.00083522	0.00055413
Deaths	Liaoning	Both	All ages	Tracheal, bronchus, and lung cancer	Occupational risks	Percent	2021	0.10015007	0.12885595	0.0727601
Deaths	Liaoning	Both	All ages	Tracheal, bronchus, and lung cancer	Occupational carcinogens	Percent	2021	0.10015007	0.12885595	0.0727601
Deaths	Liaoning	Both	All ages	Tracheal, bronchus, and lung cancer	Occupational exposure to asbestos	Percent	2021	0.05247239	0.0787624	0.03321781
Deaths	Liaoning	Both	All ages	Tracheal, bronchus, and lung cancer	Occupational exposure to arsenic	Percent	2021	0.00516707	0.00860032	0.00190399
Deaths	Liaoning	Both	All ages	Tracheal, bronchus, and lung cancer	Occupational exposure to beryllium	Percent	2021	0.00018142	0.00021936	0.00015095

Deaths	Liaoning	Both	All ages	Tracheal, bronchus, and lung cancer	Occupational exposure to cadmium	Percent	2021	0.00050332	0.0061524	0.0040018
Deaths	Gansu	Both	All ages	Tracheal, bronchus, and lung cancer	Environmental/occupational risks	Percent	2021	0.42155081	0.57632717	0.27660778
Deaths	Shanxi	Both	All ages	Tracheal, bronchus, and lung cancer	Occupational risks	Percent	2021	0.09371574	0.1202999	0.06820695
Deaths	Shanxi	Both	All ages	Tracheal, bronchus, and lung cancer	Occupational carcinogens	Percent	2021	0.09371574	0.1202999	0.06820695
Deaths	Shanxi	Both	All ages	Tracheal, bronchus, and lung cancer	Occupational exposure to asbestos	Percent	2021	0.03800343	0.05476965	0.02286537
Deaths	Shanxi	Both	All ages	Tracheal, bronchus, and lung cancer	Occupational exposure to arsenic	Percent	2021	0.00600165	0.00977948	0.00236542
Deaths	Shanxi	Both	All ages	Tracheal, bronchus, and lung cancer	Occupational exposure to beryllium	Percent	2021	0.00021093	0.00025901	0.00017326
Deaths	Shanxi	Both	All ages	Tracheal, bronchus, and lung cancer	Occupational exposure to cadmium	Percent	2021	0.00058426	0.00072196	0.00045502
Deaths	Shanghai	Both	All ages	Tracheal, bronchus, and lung cancer	Occupational risks	Percent	2021	0.0737727	0.09639843	0.05439074
Deaths	Shanghai	Both	All ages	Tracheal, bronchus, and lung cancer	Occupational carcinogens	Percent	2021	0.0737727	0.09639843	0.05439074
Deaths	Shanghai	Both	All ages	Tracheal, bronchus, and lung cancer	Occupational exposure to asbestos	Percent	2021	0.03282485	0.05136732	0.01925644

Deaths	Shanghai	Both	All ages	Tracheal, bronchus, and lung cancer	Occupational exposure to arsenic	Percent	2021	0.0042487<br>4	0.0071502<br>8	0.0015574<br>6
Deaths	Shanghai	Both	All ages	Tracheal, bronchus, and lung cancer	Occupational exposure to beryllium	Percent	2021	0.0001441<br>4	0.0001765<br>8	0.0001140<br>3
Deaths	Shanghai	Both	All ages	Tracheal, bronchus, and lung cancer	Occupational exposure to cadmium	Percent	2021	0.0004135<br>6	0.0005116<br>7	0.0003244<br>2
Deaths	Zhejiang	Both	All ages	Tracheal, bronchus, and lung cancer	Particulate matter pollution	Percent	2021	0.2189197<br>4	0.2943397<br>5	0.1429464<br>5
Deaths	Shanxi	Both	All ages	Tracheal, bronchus, and lung cancer	Occupational exposure to chromium	Percent	2021	0.0012380<br>5	0.0014599<br>6	0.0010399
Deaths	Qinghai	Both	All ages	Tracheal, bronchus, and lung cancer	Air pollution	Percent	2021	0.2367414<br>6	0.3336112<br>5	0.1490711<br>8
Deaths	Qinghai	Both	All ages	Tracheal, bronchus, and lung cancer	Ambient particulate matter pollution	Percent	2021	0.1755929	0.2531001<br>1	0.0911422<br>8
Deaths	Qinghai	Both	All ages	Tracheal, bronchus, and lung cancer	Household air pollution from solid fuels	Percent	2021	0.0609784<br>6	0.1912421	0.0065363<br>2
Deaths	Qinghai	Both	All ages	Tracheal, bronchus, and lung cancer	Other environmental risks	Percent	2021	0.0591459<br>8	0.2414507<br>6	-0.0249885
Deaths	Guangxi	Both	All ages	Tracheal, bronchus, and lung cancer	Residential radon	Percent	2021	0.0309775<br>1	0.1190951<br>4	-0.0108217
Deaths	Guangxi	Both	All ages	Tracheal, bronchus, and lung cancer	Behavioral risks	Percent	2021	0.6146498<br>1	0.8151269<br>9	0.2693868<br>6

Deaths	Liaoning	Both	All ages	Larynx cancer	Environmental/occupational risks	Percent	2021	0.05240321	0.08380891	0.02986207
Deaths	Liaoning	Both	All ages	Larynx cancer	Behavioral risks	Percent	2021	0.80712751	0.88964442	0.69969594
Deaths	Liaoning	Both	All ages	Tracheal, bronchus, and lung cancer	Occupational exposure to chromium	Percent	2021	0.0107921	0.01255833	0.00909399
Deaths	Ningxia	Both	All ages	Tracheal, bronchus, and lung cancer	Residential radon	Percent	2021	0.04588923	0.16947931	-0.0182679
Deaths	Ningxia	Both	All ages	Tracheal, bronchus, and lung cancer	Behavioral risks	Percent	2021	0.60486216	0.83229713	0.26420271
Deaths	Anhui	Both	All ages	Tracheal, bronchus, and lung cancer	Residential radon	Percent	2021	0.02994422	0.10747248	-0.0120123
Deaths	Anhui	Both	All ages	Tracheal, bronchus, and lung cancer	Behavioral risks	Percent	2021	0.69673586	0.89257521	0.33731565
Deaths	Guangdong	Both	All ages	Tracheal, bronchus, and lung cancer	Particulate matter pollution	Percent	2021	0.19617267	0.26672833	0.12640139
Deaths	Liaoning	Both	All ages	Tracheal, bronchus, and lung cancer	Occupational exposure to diesel engine exhaust	Percent	2021	0.01194158	0.01391449	0.01013997
Deaths	Liaoning	Both	All ages	Tracheal, bronchus, and lung cancer	Occupational exposure to nickel	Percent	2021	0.00493079	0.01080758	0.00102417
Deaths	Liaoning	Both	All ages	Tracheal, bronchus, and lung cancer	Occupational exposure to polycyclic aromatic hydrocarbons	Percent	2021	0.00367383	0.00435208	0.00304757

Deaths	Liaoning	Both	All ages	Tracheal, bronchus, and lung cancer	Occupational exposure to silica	Percent	2021	0.02309772	0.0368934	0.01078578
Deaths	Liaoning	Both	All ages	Tracheal, bronchus, and lung cancer	All risk factors	Percent	2021	0.78660255	0.92936843	0.56553511
Deaths	Liaoning	Both	All ages	Tracheal, bronchus, and lung cancer	Environmental/occupational risks	Percent	2021	0.35815425	0.4699745	0.25753091
Deaths	Anhui	Both	All ages	Larynx cancer	Occupational exposure to sulfuric acid	Percent	2021	0.04049058	0.07577447	0.01701477
Deaths	Anhui	Both	All ages	Larynx cancer	All risk factors	Percent	2021	0.81395959	0.89347584	0.71503364
Deaths	Guangxi	Both	All ages	Tracheal, bronchus, and lung cancer	Particulate matter pollution	Percent	2021	0.25411874	0.35132244	0.16405532
Deaths	Beijing	Both	All ages	Larynx cancer	Occupational exposure to sulfuric acid	Percent	2021	0.02866536	0.05293627	0.01155769
Deaths	Beijing	Both	All ages	Larynx cancer	All risk factors	Percent	2021	0.81841266	0.89930374	0.70428074
Deaths	Hong Kong Special Administrative Region of China	Both	All ages	Larynx cancer	Smoking	Percent	2021	0.61587625	0.76823241	0.44427086
Deaths	Hong Kong Special Administrative Region of China	Both	All ages	Larynx cancer	High alcohol use	Percent	2021	0.18090732	0.27410575	0.08346132
Deaths	Hong Kong Special Administrative Region of China	Both	All ages	Larynx cancer	Occupational risks	Percent	2021	0.039736	0.06722254	0.0206852

Deaths	Hong Kong Special Administrative Region of China	Both	All ages	Larynx cancer	Occupational carcinogens	Percent	2021	0.039736	0.0672225 4	0.0206852
Deaths	Hong Kong Special Administrative Region of China	Both	All ages	Larynx cancer	Occupational exposure to asbestos	Percent	2021	0.0083385 1	0.0145286 1	0.0039882 1
Deaths	Ningxia	Both	All ages	Tracheal, bronchus, and lung cancer	Particulate matter pollution	Percent	2021	0.2474808 6	0.3372752 8	0.1570320 5
Deaths	Hong Kong Special Administrative Region of China	Both	All ages	Tracheal, bronchus, and lung cancer	Occupational risks	Percent	2021	0.0733896 7	0.0972882 4	0.0555373 8
Deaths	Hong Kong Special Administrative Region of China	Both	All ages	Tracheal, bronchus, and lung cancer	Occupational carcinogens	Percent	2021	0.0733896 7	0.0972882 4	0.0555373 8
Deaths	Hong Kong Special Administrative Region of China	Both	All ages	Tracheal, bronchus, and lung cancer	Occupational exposure to asbestos	Percent	2021	0.0234389 2	0.0377943 3	0.0127839 2
Deaths	Hong Kong Special Administrative Region of China	Both	All ages	Tracheal, bronchus, and lung cancer	Occupational exposure to arsenic	Percent	2021	0.0050103	0.0081148 9	0.0019110 7
Deaths	Hong Kong Special Administrative Region of China	Both	All ages	Tracheal, bronchus, and lung cancer	Occupational exposure to beryllium	Percent	2021	0.0001775	0.0002160 2	0.0001430 2
Deaths	Hong Kong Special Administrative Region of China	Both	All ages	Tracheal, bronchus, and lung cancer	Occupational exposure to cadmium	Percent	2021	0.0004937 6	0.0006177	0.0003951 9
Deaths	Anhui	Both	All ages	Tracheal, bronchus, and lung cancer	Particulate matter pollution	Percent	2021	0.2964144 4	0.3935899 9	0.1960648 8
Deaths	Qinghai	Both	All ages	Tracheal, bronchus, and lung cancer	Residential radon	Percent	2021	0.0591459 8	0.2414507 6	-0.0249885

Deaths	Qinghai	Both	All ages	Tracheal, bronchus, and lung cancer	Behavioral risks	Percent	2021	0.6050286	0.8012966	0.26990959
Deaths	Qinghai	Both	All ages	Tracheal, bronchus, and lung cancer	Particulate matter pollution	Percent	2021	0.23674146	0.33361125	0.14907118
Deaths	Beijing	Both	All ages	Larynx cancer	Smoking	Percent	2021	0.78502389	0.87940453	0.65961407
Deaths	Beijing	Both	All ages	Larynx cancer	High alcohol use	Percent	2021	0.17477806	0.25281779	0.09317253
Deaths	Beijing	Both	All ages	Larynx cancer	Occupational risks	Percent	2021	0.04604097	0.07263756	0.02620835
Deaths	Beijing	Both	All ages	Larynx cancer	Occupational carcinogens	Percent	2021	0.04604097	0.07263756	0.02620835
Deaths	Beijing	Both	All ages	Larynx cancer	Occupational exposure to asbestos	Percent	2021	0.01762436	0.02822251	0.00889057
Deaths	Hubei	Both	All ages	Larynx cancer	Smoking	Percent	2021	0.756495	0.84379595	0.6402704
Deaths	Hubei	Both	All ages	Larynx cancer	High alcohol use	Percent	2021	0.15768848	0.23224074	0.08190261
Deaths	Hubei	Both	All ages	Larynx cancer	Occupational risks	Percent	2021	0.04431781	0.07601718	0.02237447
Deaths	Hubei	Both	All ages	Larynx cancer	Occupational carcinogens	Percent	2021	0.04431781	0.07601718	0.02237447

Deaths	Hubei	Both	All ages	Larynx cancer	Occupational exposure to asbestos	Percent	2021	0.00627368	0.01067133	0.00311779
Deaths	Guangdong	Both	All ages	Larynx cancer	Environmental/occupational risks	Percent	2021	0.04924214	0.08314176	0.02526307
Deaths	Guangdong	Both	All ages	Larynx cancer	Behavioral risks	Percent	2021	0.81670567	0.89018475	0.72271572
Deaths	Guangdong	Both	All ages	Tracheal, bronchus, and lung cancer	Occupational exposure to chromium	Percent	2021	0.0144639	0.01673866	0.01244478
Deaths	Sichuan	Both	All ages	Larynx cancer	Environmental/occupational risks	Percent	2021	0.05094258	0.08704038	0.02620849
Deaths	Sichuan	Both	All ages	Larynx cancer	Behavioral risks	Percent	2021	0.80058173	0.87871427	0.70429368
Deaths	Sichuan	Both	All ages	Tracheal, bronchus, and lung cancer	Occupational exposure to chromium	Percent	2021	0.01476866	0.01738255	0.01244399
Deaths	Anhui	Both	All ages	Larynx cancer	Smoking	Percent	2021	0.78514767	0.873031	0.67546124
Deaths	Anhui	Both	All ages	Larynx cancer	High alcohol use	Percent	2021	0.13254087	0.20093903	0.06466423
Deaths	Anhui	Both	All ages	Larynx cancer	Occupational risks	Percent	2021	0.04877126	0.0844279	0.02535178
Deaths	Anhui	Both	All ages	Larynx cancer	Occupational carcinogens	Percent	2021	0.04877126	0.0844279	0.02535178

Deaths	Anhui	Both	All ages	Larynx cancer	Occupational exposure to asbestos	Percent	2021	0.00847352	0.01373278	0.004188
Deaths	Guangdong	Both	All ages	Tracheal, bronchus, and lung cancer	Occupational exposure to diesel engine exhaust	Percent	2021	0.01608373	0.0188322	0.01371409
Deaths	Guangdong	Both	All ages	Tracheal, bronchus, and lung cancer	Occupational exposure to nickel	Percent	2021	0.00646448	0.01466095	0.0013895
Deaths	Guangdong	Both	All ages	Tracheal, bronchus, and lung cancer	Occupational exposure to polycyclic aromatic hydrocarbons	Percent	2021	0.00489271	0.00586256	0.00404426
Deaths	Guangdong	Both	All ages	Tracheal, bronchus, and lung cancer	Occupational exposure to silica	Percent	2021	0.03026024	0.04872231	0.01423259
Deaths	Guangdong	Both	All ages	Tracheal, bronchus, and lung cancer	All risk factors	Percent	2021	0.77253013	0.90933601	0.53637356
Deaths	Sichuan	Both	All ages	Tracheal, bronchus, and lung cancer	Occupational exposure to diesel engine exhaust	Percent	2021	0.01527873	0.01768817	0.01326479
Deaths	Sichuan	Both	All ages	Tracheal, bronchus, and lung cancer	Occupational exposure to nickel	Percent	2021	0.00698451	0.0156976	0.00142571
Deaths	Sichuan	Both	All ages	Tracheal, bronchus, and lung cancer	Occupational exposure to polycyclic aromatic hydrocarbons	Percent	2021	0.00500884	0.00618083	0.00413139
Deaths	Sichuan	Both	All ages	Tracheal, bronchus, and lung cancer	Occupational exposure to silica	Percent	2021	0.03229587	0.05101292	0.0147367
Deaths	Sichuan	Both	All ages	Tracheal, bronchus, and lung cancer	All risk factors	Percent	2021	0.78596756	0.91445335	0.5794707

Deaths	Guangdong	Both	All ages	Tracheal, bronchus, and lung cancer	Environmental/occupational risks	Percent	2021	0.29487592	0.39433469	0.2143369
Deaths	Hong Kong Special Administrative Region of China	Both	All ages	Tracheal, bronchus, and lung cancer	Air pollution	Percent	2021	0.14403602	0.20174794	0.09205739
Deaths	Hong Kong Special Administrative Region of China	Both	All ages	Tracheal, bronchus, and lung cancer	Ambient particulate matter pollution	Percent	2021	0.14345389	0.19964987	0.09116922
Deaths	Hong Kong Special Administrative Region of China	Both	All ages	Tracheal, bronchus, and lung cancer	Household air pollution from solid fuels	Percent	2021	0.00055504	0.00232756	1.41E-12
Deaths	Hong Kong Special Administrative Region of China	Both	All ages	Tracheal, bronchus, and lung cancer	Other environmental risks	Percent	2021	0.0390972	0.12956615	-0.0179194
Deaths	Sichuan	Both	All ages	Tracheal, bronchus, and lung cancer	Environmental/occupational risks	Percent	2021	0.35329951	0.45965427	0.25327229
Deaths	Beijing	Both	All ages	Tracheal, bronchus, and lung cancer	Occupational risks	Percent	2021	0.09055967	0.11606038	0.06654652
Deaths	Beijing	Both	All ages	Tracheal, bronchus, and lung cancer	Occupational carcinogens	Percent	2021	0.09055967	0.11606038	0.06654652
Deaths	Beijing	Both	All ages	Tracheal, bronchus, and lung cancer	Occupational exposure to asbestos	Percent	2021	0.05118332	0.07460202	0.03087988
Deaths	Beijing	Both	All ages	Tracheal, bronchus, and lung cancer	Occupational exposure to arsenic	Percent	2021	0.00414099	0.00672479	0.00154069
Deaths	Beijing	Both	All ages	Tracheal, bronchus, and lung cancer	Occupational exposure to beryllium	Percent	2021	0.00014473	0.00017087	0.00011804

Deaths	Beijing	Both	All ages	Tracheal, bronchus, and lung cancer	Occupational exposure to cadmium	Percent	2021	0.0004053 2	0.0005019 3	0.0003185 2
Deaths	Anhui	Both	All ages	Tracheal, bronchus, and lung cancer	Occupational risks	Percent	2021	0.0847508 9	0.1151575	0.0607674 7
Deaths	Anhui	Both	All ages	Tracheal, bronchus, and lung cancer	Occupational carcinogens	Percent	2021	0.0847508 9	0.1151575	0.0607674 7
Deaths	Anhui	Both	All ages	Tracheal, bronchus, and lung cancer	Occupational exposure to asbestos	Percent	2021	0.0253439 6	0.0401529 2	0.0145729 4
Deaths	Anhui	Both	All ages	Tracheal, bronchus, and lung cancer	Occupational exposure to arsenic	Percent	2021	0.0064872 6	0.0109063 8	0.0024579 9
Deaths	Anhui	Both	All ages	Tracheal, bronchus, and lung cancer	Occupational exposure to beryllium	Percent	2021	0.0002312 1	0.0002804	0.0001878 3
Deaths	Anhui	Both	All ages	Tracheal, bronchus, and lung cancer	Occupational exposure to cadmium	Percent	2021	0.0006307 5	0.0007662 5	0.0005077 4
Deaths	Hubei	Both	All ages	Tracheal, bronchus, and lung cancer	Occupational risks	Percent	2021	0.0782153 1	0.1065198 7	0.0555375 7
Deaths	Hubei	Both	All ages	Tracheal, bronchus, and lung cancer	Occupational carcinogens	Percent	2021	0.0782153 1	0.1065198 7	0.0555375 7
Deaths	Hubei	Both	All ages	Tracheal, bronchus, and lung cancer	Occupational exposure to asbestos	Percent	2021	0.0178420 6	0.0288343 2	0.0100231 7
Deaths	Hubei	Both	All ages	Tracheal, bronchus, and lung cancer	Occupational exposure to arsenic	Percent	2021	0.0064763 4	0.0106657	0.0025316

Deaths	Hubei	Both	All ages	Tracheal, bronchus, and lung cancer	Occupational exposure to beryllium	Percent	2021	0.00022566	0.00027096	0.00018306
Deaths	Hubei	Both	All ages	Tracheal, bronchus, and lung cancer	Occupational exposure to cadmium	Percent	2021	0.00062946	0.00076552	0.00050397
Deaths	Sichuan	Both	All ages	Tracheal, bronchus, and lung cancer	Air pollution	Percent	2021	0.26464643	0.36070326	0.17193361
Deaths	Sichuan	Both	All ages	Tracheal, bronchus, and lung cancer	Ambient particulate matter pollution	Percent	2021	0.19382919	0.28154919	0.09895932
Deaths	Sichuan	Both	All ages	Tracheal, bronchus, and lung cancer	Household air pollution from solid fuels	Percent	2021	0.0706842	0.20488836	0.01013393
Deaths	Sichuan	Both	All ages	Tracheal, bronchus, and lung cancer	Other environmental risks	Percent	2021	0.03463953	0.14706964	-0.019115
Deaths	Hong Kong Special Administrative Region of China	Both	All ages	Tracheal, bronchus, and lung cancer	Residential radon	Percent	2021	0.0390972	0.12956615	-0.0179194
Deaths	Hong Kong Special Administrative Region of China	Both	All ages	Tracheal, bronchus, and lung cancer	Behavioral risks	Percent	2021	0.45826846	0.71832362	0.15514683
Deaths	Hong Kong Special Administrative Region of China	Both	All ages	Tracheal, bronchus, and lung cancer	Particulate matter pollution	Percent	2021	0.14403602	0.20174794	0.09205739
Deaths	Sichuan	Both	All ages	Tracheal, bronchus, and lung cancer	Residential radon	Percent	2021	0.03463953	0.14706964	-0.019115
Deaths	Sichuan	Both	All ages	Tracheal, bronchus, and lung cancer	Behavioral risks	Percent	2021	0.66006851	0.8600074	0.32274682

Deaths	Sichuan	Both	All ages	Tracheal, bronchus, and lung cancer	Particulate matter pollution	Percent	2021	0.26464643	0.36070326	0.17193361
Deaths	Ningxia	Both	All ages	Larynx cancer	Smoking	Percent	2021	0.69692323	0.80230191	0.56531597
Deaths	Ningxia	Both	All ages	Larynx cancer	High alcohol use	Percent	2021	0.11327113	0.18082058	0.04887497
Deaths	Ningxia	Both	All ages	Larynx cancer	Occupational risks	Percent	2021	0.04751412	0.08196959	0.02210897
Deaths	Ningxia	Both	All ages	Larynx cancer	Occupational carcinogens	Percent	2021	0.04751412	0.08196959	0.02210897
Deaths	Ningxia	Both	All ages	Larynx cancer	Occupational exposure to asbestos	Percent	2021	0.00471277	0.00775601	0.00239038
Deaths	Hubei	Both	All ages	Larynx cancer	Environmental/occupational risks	Percent	2021	0.04431781	0.07601718	0.02237447
Deaths	Hubei	Both	All ages	Larynx cancer	Behavioral risks	Percent	2021	0.77933852	0.85780281	0.68070892
Deaths	Hubei	Both	All ages	Tracheal, bronchus, and lung cancer	Occupational exposure to chromium	Percent	2021	0.00133509	0.00157656	0.00112022
Deaths	Hubei	Both	All ages	Tracheal, bronchus, and lung cancer	Occupational exposure to diesel engine exhaust	Percent	2021	0.01433241	0.01668017	0.01224884
Deaths	Hubei	Both	All ages	Tracheal, bronchus, and lung cancer	Occupational exposure to nickel	Percent	2021	0.00621086	0.01394674	0.00137229

Deaths	Hubei	Both	All ages	Tracheal, bronchus, and lung cancer	Occupational exposure to polycyclic aromatic hydrocarbons	Percent	2021	0.0045267 4	0.0055289 4	0.0036126 8
Deaths	Hubei	Both	All ages	Tracheal, bronchus, and lung cancer	Occupational exposure to silica	Percent	2021	0.0291677 1	0.0469552	0.0140545 9
Deaths	Hubei	Both	All ages	Tracheal, bronchus, and lung cancer	All risk factors	Percent	2021	0.7760424 1	0.8889146 7	0.5646219
Deaths	Hubei	Both	All ages	Tracheal, bronchus, and lung cancer	Environmental/occupational risks	Percent	2021	0.3519744 1	0.4485333 9	0.2532950 2
Deaths	Tianjin	Both	All ages	Larynx cancer	Smoking	Percent	2021	0.7813309 9	0.8695850 2	0.6634673 3
Deaths	Tianjin	Both	All ages	Larynx cancer	High alcohol use	Percent	2021	0.1556141 6	0.2337161 2	0.0869456 7
Deaths	Tianjin	Both	All ages	Larynx cancer	Occupational risks	Percent	2021	0.0360384 9	0.0606075 6	0.0196245
Deaths	Tianjin	Both	All ages	Larynx cancer	Occupational carcinogens	Percent	2021	0.0360384 9	0.0606075 6	0.0196245
Deaths	Tianjin	Both	All ages	Larynx cancer	Occupational exposure to asbestos	Percent	2021	0.0085719 9	0.0136691 1	0.0045040 7
Deaths	Qinghai	Both	All ages	Larynx cancer	Tobacco	Percent	2021	0.6846686 6	0.7937301 8	0.5589763 7
Deaths	Tianjin	Both	All ages	Larynx cancer	Occupational exposure to sulfuric acid	Percent	2021	0.0275909 2	0.0504777 7	0.0110212

Deaths	Tianjin	Both	All ages	Larynx cancer	All risk factors	Percent	2021	0.81064852	0.88885854	0.70719129
Deaths	Sichuan	Both	All ages	Larynx cancer	Smoking	Percent	2021	0.78017633	0.86767106	0.67243747
Deaths	Sichuan	Both	All ages	Larynx cancer	High alcohol use	Percent	2021	0.14325276	0.21342157	0.07127215
Deaths	Sichuan	Both	All ages	Larynx cancer	Occupational risks	Percent	2021	0.05094258	0.08704038	0.02620849
Deaths	Sichuan	Both	All ages	Larynx cancer	Occupational carcinogens	Percent	2021	0.05094258	0.08704038	0.02620849
Deaths	Sichuan	Both	All ages	Larynx cancer	Occupational exposure to asbestos	Percent	2021	0.0078866	0.01325294	0.00402325
Deaths	Inner Mongolia	Both	All ages	Larynx cancer	Occupational exposure to sulfuric acid	Percent	2021	0.03846423	0.07102907	0.01599714
Deaths	Inner Mongolia	Both	All ages	Larynx cancer	All risk factors	Percent	2021	0.83280285	0.90769941	0.73303523
Deaths	Hunan	Both	All ages	Larynx cancer	Smoking	Percent	2021	0.7857865	0.87718987	0.6690177
Deaths	Hunan	Both	All ages	Larynx cancer	High alcohol use	Percent	2021	0.13688437	0.20132336	0.07273682
Deaths	Hunan	Both	All ages	Larynx cancer	Occupational risks	Percent	2021	0.05734318	0.09119657	0.03293308

Deaths	Hunan	Both	All ages	Larynx cancer	Occupational carcinogens	Percent	2021	0.05734318	0.09119657	0.03293308
Deaths	Hunan	Both	All ages	Larynx cancer	Occupational exposure to asbestos	Percent	2021	0.01610206	0.02597913	0.00886895
Deaths	Guangxi	Both	All ages	Larynx cancer	Occupational exposure to sulfuric acid	Percent	2021	0.04521099	0.08278102	0.01869122
Deaths	Guangxi	Both	All ages	Larynx cancer	All risk factors	Percent	2021	0.77203622	0.86272127	0.66621012
Deaths	Guangxi	Both	All ages	Larynx cancer	Smoking	Percent	2021	0.73097126	0.83594488	0.61332815
Deaths	Guangxi	Both	All ages	Larynx cancer	High alcohol use	Percent	2021	0.15206972	0.22905588	0.07452171
Deaths	Guangxi	Both	All ages	Larynx cancer	Occupational risks	Percent	2021	0.05889467	0.09607641	0.03222492
Deaths	Guangxi	Both	All ages	Larynx cancer	Occupational carcinogens	Percent	2021	0.05889467	0.09607641	0.03222492
Deaths	Guangxi	Both	All ages	Larynx cancer	Occupational exposure to asbestos	Percent	2021	0.01409689	0.02339584	0.00721259
Deaths	Guangdong	Both	All ages	Larynx cancer	Smoking	Percent	2021	0.79384537	0.87392673	0.68741746
Deaths	Guangdong	Both	All ages	Larynx cancer	High alcohol use	Percent	2021	0.15674603	0.2223984	0.08245917

Deaths	Guangdong	Both	All ages	Larynx cancer	Occupational risks	Percent	2021	0.04924214	0.08314176	0.02526307
Deaths	Guangdong	Both	All ages	Larynx cancer	Occupational carcinogens	Percent	2021	0.04924214	0.08314176	0.02526307
Deaths	Guangdong	Both	All ages	Larynx cancer	Occupational exposure to asbestos	Percent	2021	0.00889697	0.01487388	0.00457145
Deaths	Ningxia	Both	All ages	Tracheal, bronchus, and lung cancer	Occupational risks	Percent	2021	0.07611374	0.10283299	0.05380269
Deaths	Ningxia	Both	All ages	Tracheal, bronchus, and lung cancer	Occupational carcinogens	Percent	2021	0.07611374	0.10283299	0.05380269
Deaths	Ningxia	Both	All ages	Tracheal, bronchus, and lung cancer	Occupational exposure to asbestos	Percent	2021	0.01500848	0.02416493	0.00867924
Deaths	Ningxia	Both	All ages	Tracheal, bronchus, and lung cancer	Occupational exposure to arsenic	Percent	2021	0.00664427	0.01088561	0.00255135
Deaths	Ningxia	Both	All ages	Tracheal, bronchus, and lung cancer	Occupational exposure to beryllium	Percent	2021	0.00023563	0.00028231	0.0001932
Deaths	Ningxia	Both	All ages	Tracheal, bronchus, and lung cancer	Occupational exposure to cadmium	Percent	2021	0.00064612	0.00079079	0.00052652
Deaths	Tianjin	Both	All ages	Tracheal, bronchus, and lung cancer	Occupational risks	Percent	2021	0.0648551	0.08489597	0.04756028
Deaths	Tianjin	Both	All ages	Tracheal, bronchus, and lung cancer	Occupational carcinogens	Percent	2021	0.0648551	0.08489597	0.04756028

Deaths	Tianjin	Both	All ages	Tracheal, bronchus, and lung cancer	Occupational exposure to asbestos	Percent	2021	0.02417831	0.03756417	0.01462309
Deaths	Tianjin	Both	All ages	Tracheal, bronchus, and lung cancer	Occupational exposure to arsenic	Percent	2021	0.0042329	0.00690014	0.0015152 6
Deaths	Tianjin	Both	All ages	Tracheal, bronchus, and lung cancer	Occupational exposure to beryllium	Percent	2021	0.0014875	0.0018268	0.0012126
Deaths	Tianjin	Both	All ages	Tracheal, bronchus, and lung cancer	Occupational exposure to cadmium	Percent	2021	0.0041425	0.0050728	0.0032337
Deaths	Ningxia	Both	All ages	Tracheal, bronchus, and lung cancer	Occupational exposure to chromium	Percent	2021	0.0135604	0.0159011	0.0115312
Deaths	Sichuan	Both	All ages	Tracheal, bronchus, and lung cancer	Occupational risks	Percent	2021	0.08906159	0.11585907	0.06416496
Deaths	Sichuan	Both	All ages	Tracheal, bronchus, and lung cancer	Occupational carcinogens	Percent	2021	0.08906159	0.11585907	0.06416496
Deaths	Sichuan	Both	All ages	Tracheal, bronchus, and lung cancer	Occupational exposure to asbestos	Percent	2021	0.02315538	0.03587588	0.01353406
Deaths	Sichuan	Both	All ages	Tracheal, bronchus, and lung cancer	Occupational exposure to arsenic	Percent	2021	0.00726339	0.01222819	0.00274721
Deaths	Sichuan	Both	All ages	Tracheal, bronchus, and lung cancer	Occupational exposure to beryllium	Percent	2021	0.0025697	0.0030555	0.002154
Deaths	Sichuan	Both	All ages	Tracheal, bronchus, and lung cancer	Occupational exposure to cadmium	Percent	2021	0.0070695	0.0085365	0.0057015

Deaths	Hunan	Both	All ages	Tracheal, bronchus, and lung cancer	Occupational risks	Percent	2021	0.10864689	0.14252583	0.08061239
Deaths	Hunan	Both	All ages	Tracheal, bronchus, and lung cancer	Occupational carcinogens	Percent	2021	0.10864689	0.14252583	0.08061239
Deaths	Hunan	Both	All ages	Tracheal, bronchus, and lung cancer	Occupational exposure to asbestos	Percent	2021	0.04567652	0.06921207	0.02756147
Deaths	Hunan	Both	All ages	Tracheal, bronchus, and lung cancer	Occupational exposure to arsenic	Percent	2021	0.00688442	0.01140853	0.00256704
Deaths	Hunan	Both	All ages	Tracheal, bronchus, and lung cancer	Occupational exposure to beryllium	Percent	2021	0.00024054	0.00029277	0.00019575
Deaths	Hunan	Both	All ages	Tracheal, bronchus, and lung cancer	Occupational exposure to cadmium	Percent	2021	0.00066958	0.00082371	0.00054303
Deaths	Hunan	Both	All ages	Larynx cancer	Occupational exposure to sulfuric acid	Percent	2021	0.04166133	0.07559769	0.01680487
Deaths	Hunan	Both	All ages	Larynx cancer	All risk factors	Percent	2021	0.8144829	0.89440868	0.71689745
Deaths	Guangxi	Both	All ages	Tracheal, bronchus, and lung cancer	Occupational risks	Percent	2021	0.10881277	0.14134958	0.0807991
Deaths	Guangxi	Both	All ages	Tracheal, bronchus, and lung cancer	Occupational carcinogens	Percent	2021	0.10881277	0.14134958	0.0807991
Deaths	Guangxi	Both	All ages	Tracheal, bronchus, and lung cancer	Occupational exposure to asbestos	Percent	2021	0.04025227	0.06253881	0.02324597

Deaths	Guangxi	Both	All ages	Tracheal, bronchus, and lung cancer	Occupational exposure to arsenic	Percent	2021	0.00753558	0.01235305	0.00283206
Deaths	Guangxi	Both	All ages	Tracheal, bronchus, and lung cancer	Occupational exposure to beryllium	Percent	2021	0.00026392	0.00031871	0.00021453
Deaths	Guangxi	Both	All ages	Tracheal, bronchus, and lung cancer	Occupational exposure to cadmium	Percent	2021	0.0007332	0.0008968 6	0.0005847 6
Deaths	Guangdong	Both	All ages	Tracheal, bronchus, and lung cancer	Occupational risks	Percent	2021	0.0896699 6	0.1168575 6	0.0651751 2
Deaths	Guangdong	Both	All ages	Tracheal, bronchus, and lung cancer	Occupational carcinogens	Percent	2021	0.0896699 6	0.1168575 6	0.0651751 2
Deaths	Guangdong	Both	All ages	Tracheal, bronchus, and lung cancer	Occupational exposure to asbestos	Percent	2021	0.0259436 1	0.0411251 4	0.0151880 1
Deaths	Guangdong	Both	All ages	Tracheal, bronchus, and lung cancer	Occupational exposure to arsenic	Percent	2021	0.0068162 5	0.0111844 5	0.0026632 7
Deaths	Guangdong	Both	All ages	Tracheal, bronchus, and lung cancer	Occupational exposure to beryllium	Percent	2021	0.0002275 1	0.0002706 8	0.0001876 2
Deaths	Guangdong	Both	All ages	Tracheal, bronchus, and lung cancer	Occupational exposure to cadmium	Percent	2021	0.0006639 5	0.0008014 7	0.0005411 3
Deaths	China	Both	All ages	Larynx cancer	High alcohol use	Percent	2021	0.1452316 4	0.2098665 5	0.0770268 6
Deaths	China	Both	All ages	Larynx cancer	Occupational risks	Percent	2021	0.0517584 8	0.0855219 7	0.0281666 9

Deaths	China	Both	All ages	Larynx cancer	Occupational carcinogens	Percent	2021	0.05175848	0.08552197	0.02816669
Deaths	China	Both	All ages	Larynx cancer	Occupational exposure to asbestos	Percent	2021	0.01184061	0.01864789	0.0066204
Deaths	China	Both	All ages	Larynx cancer	Occupational exposure to sulfuric acid	Percent	2021	0.04021019	0.0738571	0.01690033
Deaths	Hong Kong Special Administrative Region of China	Both	All ages	Larynx cancer	Environmental/occupational risks	Percent	2021	0.039736	0.06722254	0.0206852
Deaths	Hong Kong Special Administrative Region of China	Both	All ages	Larynx cancer	Behavioral risks	Percent	2021	0.67648359	0.80450075	0.52609025
Deaths	Hong Kong Special Administrative Region of China	Both	All ages	Tracheal, bronchus, and lung cancer	Occupational exposure to chromium	Percent	2021	0.00111608	0.00131639	0.00094419
Deaths	Ningxia	Both	All ages	Larynx cancer	Occupational exposure to sulfuric acid	Percent	2021	0.04292378	0.07764443	0.01770769
Deaths	Ningxia	Both	All ages	Larynx cancer	All risk factors	Percent	2021	0.73058674	0.82241858	0.61274057
Deaths	Hong Kong Special Administrative Region of China	Both	All ages	Tracheal, bronchus, and lung cancer	Occupational exposure to diesel engine exhaust	Percent	2021	0.0141977	0.01699406	0.01188399
Deaths	Hong Kong Special Administrative Region of China	Both	All ages	Tracheal, bronchus, and lung cancer	Occupational exposure to nickel	Percent	2021	0.00468594	0.01082451	0.00098562
Deaths	Hong Kong Special Administrative Region of China	Both	All ages	Tracheal, bronchus, and lung cancer	Occupational exposure to polycyclic aromatic hydrocarbons	Percent	2021	0.00380184	0.0045532	0.00310778

Deaths	Hong Kong Special Administrative Region of China	Both	All ages	Tracheal, bronchus, and lung cancer	Occupational exposure to silica	Percent	2021	0.02278762	0.03611479	0.01066492
Deaths	Hong Kong Special Administrative Region of China	Both	All ages	Tracheal, bronchus, and lung cancer	All risk factors	Percent	2021	0.59209718	0.7929683	0.35720173
Deaths	Hong Kong Special Administrative Region of China	Both	All ages	Tracheal, bronchus, and lung cancer	Environmental/occupational risks	Percent	2021	0.23792488	0.31918688	0.16911324
Deaths	Tianjin	Both	All ages	Tracheal, bronchus, and lung cancer	Air pollution	Percent	2021	0.28991414	0.38278822	0.19374212
Deaths	Tianjin	Both	All ages	Tracheal, bronchus, and lung cancer	Ambient particulate matter pollution	Percent	2021	0.28839341	0.38092094	0.19340691
Deaths	Tianjin	Both	All ages	Tracheal, bronchus, and lung cancer	Household air pollution from solid fuels	Percent	2021	0.00143717	0.01385026	1.74E-07
Deaths	Tianjin	Both	All ages	Tracheal, bronchus, and lung cancer	Other environmental risks	Percent	2021	0.03456224	0.13422858	-0.0136824
Deaths	Hunan	Both	All ages	Tracheal, bronchus, and lung cancer	Air pollution	Percent	2021	0.2655851	0.36397027	0.17322699
Deaths	Hunan	Both	All ages	Tracheal, bronchus, and lung cancer	Ambient particulate matter pollution	Percent	2021	0.21122692	0.298969	0.1140761
Deaths	Hunan	Both	All ages	Tracheal, bronchus, and lung cancer	Household air pollution from solid fuels	Percent	2021	0.05420814	0.19627548	0.00494087
Deaths	Hunan	Both	All ages	Tracheal, bronchus, and lung cancer	Other environmental risks	Percent	2021	0.02585524	0.09438347	-0.0110605

Deaths	Qinghai	Both	All ages	Larynx cancer	Environmental/occupational risks	Percent	2021	0.05589784	0.09279642	0.0307033 6
Deaths	Qinghai	Both	All ages	Larynx cancer	Behavioral risks	Percent	2021	0.7014828 8	0.8067474	0.5795872 2
Deaths	Qinghai	Both	All ages	Tracheal, bronchus, and lung cancer	Occupational exposure to chromium	Percent	2021	0.0013273 9	0.0015716 2	0.0011043 7
Deaths	Qinghai	Both	All ages	Tracheal, bronchus, and lung cancer	Occupational exposure to diesel engine exhaust	Percent	2021	0.0132516	0.0153299 7	0.0112552 3
Deaths	Qinghai	Both	All ages	Tracheal, bronchus, and lung cancer	Occupational exposure to nickel	Percent	2021	0.0064023 9	0.0140123 6	0.0013470 3
Deaths	Qinghai	Both	All ages	Tracheal, bronchus, and lung cancer	Occupational exposure to polycyclic aromatic hydrocarbons	Percent	2021	0.0045208 1	0.0054348 1	0.0036779 4
Deaths	Qinghai	Both	All ages	Tracheal, bronchus, and lung cancer	Occupational exposure to silica	Percent	2021	0.0294744 3	0.0460002 5	0.0137118 6
Deaths	Qinghai	Both	All ages	Tracheal, bronchus, and lung cancer	All risk factors	Percent	2021	0.7498282 8	0.8862302 7	0.5461930 9
Deaths	Beijing	Both	All ages	Larynx cancer	Environmental/occupational risks	Percent	2021	0.0460409 7	0.0726375 6	0.0262083 5
Deaths	Beijing	Both	All ages	Larynx cancer	Behavioral risks	Percent	2021	0.8102715 2	0.8946738	0.6931082 5
Deaths	Beijing	Both	All ages	Tracheal, bronchus, and lung cancer	Occupational exposure to chromium	Percent	2021	0.0009022 6	0.0010586 6	0.0007669 6

Deaths	Guizhou	Both	All ages	Larynx cancer	Occupational exposure to sulfuric acid	Percent	2021	0.04514148	0.08117647	0.0189751
Deaths	Guizhou	Both	All ages	Larynx cancer	All risk factors	Percent	2021	0.7914491	0.8891617	0.66812293
Deaths	Qinghai	Both	All ages	Tracheal, bronchus, and lung cancer	Environmental/occupational risks	Percent	2021	0.35359758	0.47048275	0.24508816
Deaths	Beijing	Both	All ages	Tracheal, bronchus, and lung cancer	Occupational exposure to diesel engine exhaust	Percent	2021	0.01070843	0.01267488	0.00907361
Deaths	Beijing	Both	All ages	Tracheal, bronchus, and lung cancer	Occupational exposure to nickel	Percent	2021	0.00385016	0.00882225	0.00083175
Deaths	Beijing	Both	All ages	Tracheal, bronchus, and lung cancer	Occupational exposure to polycyclic aromatic hydrocarbons	Percent	2021	0.00307821	0.00368108	0.00254159
Deaths	Beijing	Both	All ages	Tracheal, bronchus, and lung cancer	Occupational exposure to silica	Percent	2021	0.01820498	0.02897639	0.0085712
Deaths	Beijing	Both	All ages	Tracheal, bronchus, and lung cancer	All risk factors	Percent	2021	0.78236495	0.92375945	0.56405644
Deaths	Beijing	Both	All ages	Tracheal, bronchus, and lung cancer	Environmental/occupational risks	Percent	2021	0.36638443	0.45427292	0.26818643
Deaths	Anhui	Both	All ages	Larynx cancer	Environmental/occupational risks	Percent	2021	0.04877126	0.0844279	0.02535178
Deaths	Anhui	Both	All ages	Larynx cancer	Behavioral risks	Percent	2021	0.80378874	0.886643	0.70313395

Deaths	Anhui	Both	All ages	Tracheal, bronchus, and lung cancer	Occupational exposure to chromium	Percent	2021	0.00131371	0.0015421	0.00111586
Deaths	Anhui	Both	All ages	Tracheal, bronchus, and lung cancer	Occupational exposure to diesel engine exhaust	Percent	2021	0.01362401	0.01578806	0.01168075
Deaths	Anhui	Both	All ages	Tracheal, bronchus, and lung cancer	Occupational exposure to nickel	Percent	2021	0.00628394	0.0141483	0.00126776
Deaths	Anhui	Both	All ages	Tracheal, bronchus, and lung cancer	Occupational exposure to polycyclic aromatic hydrocarbons	Percent	2021	0.00445891	0.00530331	0.00359517
Deaths	Anhui	Both	All ages	Tracheal, bronchus, and lung cancer	Occupational exposure to silica	Percent	2021	0.02926703	0.04554361	0.01319764
Deaths	Anhui	Both	All ages	Tracheal, bronchus, and lung cancer	All risk factors	Percent	2021	0.81563594	0.93682334	0.59282665
Deaths	Guizhou	Both	All ages	Larynx cancer	Smoking	Percent	2021	0.75846921	0.86980277	0.62631247
Deaths	Guizhou	Both	All ages	Larynx cancer	High alcohol use	Percent	2021	0.13161144	0.21117756	0.06030797
Deaths	Guizhou	Both	All ages	Larynx cancer	Occupational risks	Percent	2021	0.06123721	0.09869017	0.03395836
Deaths	Guizhou	Both	All ages	Larynx cancer	Occupational carcinogens	Percent	2021	0.06123721	0.09869017	0.03395836
Deaths	Guizhou	Both	All ages	Larynx cancer	Occupational exposure to asbestos	Percent	2021	0.01657567	0.02595994	0.00884305

Deaths	Guizhou	Both	All ages	Tracheal, bronchus, and lung cancer	Occupational risks	Percent	2021	0.11267509	0.1448517	0.08333449
Deaths	Guizhou	Both	All ages	Tracheal, bronchus, and lung cancer	Occupational carcinogens	Percent	2021	0.11267509	0.1448517	0.08333449
Deaths	Guizhou	Both	All ages	Tracheal, bronchus, and lung cancer	Occupational exposure to asbestos	Percent	2021	0.04842781	0.07354603	0.02817917
Deaths	Guizhou	Both	All ages	Tracheal, bronchus, and lung cancer	Occupational exposure to arsenic	Percent	2021	0.00726079	0.01180915	0.00275667
Deaths	Guizhou	Both	All ages	Tracheal, bronchus, and lung cancer	Occupational exposure to beryllium	Percent	2021	0.00026249	0.00031452	0.00021429
Deaths	Guizhou	Both	All ages	Tracheal, bronchus, and lung cancer	Occupational exposure to cadmium	Percent	2021	0.00070624	0.00085739	0.0005815
Deaths	Guizhou	Both	All ages	Tracheal, bronchus, and lung cancer	Air pollution	Percent	2021	0.25040928	0.35000579	0.15867451
Deaths	Guizhou	Both	All ages	Tracheal, bronchus, and lung cancer	Ambient particulate matter pollution	Percent	2021	0.13773901	0.20985018	0.06422302
Deaths	Guizhou	Both	All ages	Tracheal, bronchus, and lung cancer	Household air pollution from solid fuels	Percent	2021	0.11256	0.23721112	0.02992737
Deaths	Guizhou	Both	All ages	Tracheal, bronchus, and lung cancer	Other environmental risks	Percent	2021	0.03800565	0.13573738	-0.0150104
Deaths	Anhui	Both	All ages	Tracheal, bronchus, and lung cancer	Environmental/occupational risks	Percent	2021	0.37523064	0.4742704	0.27545524

Deaths	Tianjin	Both	All ages	Tracheal, bronchus, and lung cancer	Residential radon	Percent	2021	0.0345622 4	0.1342285 8	-0.0136824
Deaths	Tianjin	Both	All ages	Tracheal, bronchus, and lung cancer	Behavioral risks	Percent	2021	0.6591321 8	0.8853540 5	0.2956577 5
Deaths	Hunan	Both	All ages	Tracheal, bronchus, and lung cancer	Residential radon	Percent	2021	0.0258552 4	0.0943834 7	-0.0110605
Deaths	Hunan	Both	All ages	Tracheal, bronchus, and lung cancer	Behavioral risks	Percent	2021	0.6978077 2	0.8722585 6	0.3595008 9
Deaths	Tianjin	Both	All ages	Larynx cancer	Environmental/occupational risks	Percent	2021	0.0360384 9	0.0606075 6	0.0196245
Deaths	Tianjin	Both	All ages	Larynx cancer	Behavioral risks	Percent	2021	0.8040634 7	0.8848874 6	0.6966694 3
Deaths	Tianjin	Both	All ages	Tracheal, bronchus, and lung cancer	Occupational exposure to chromium	Percent	2021	0.0009177 5	0.0010825 2	0.0007801 6
Deaths	Tianjin	Both	All ages	Tracheal, bronchus, and lung cancer	Occupational exposure to diesel engine exhaust	Percent	2021	0.0107423 3	0.0125710 3	0.0089615 9
Deaths	Tianjin	Both	All ages	Tracheal, bronchus, and lung cancer	Occupational exposure to nickel	Percent	2021	0.0039555	0.0088738 7	0.0008099 2
Deaths	Tianjin	Both	All ages	Tracheal, bronchus, and lung cancer	Occupational exposure to polycyclic aromatic hydrocarbons	Percent	2021	0.0031412 8	0.0037857 4	0.0025922 8
Deaths	Tianjin	Both	All ages	Tracheal, bronchus, and lung cancer	Occupational exposure to silica	Percent	2021	0.0186247 4	0.0298845 5	0.0084585

Deaths	Tianjin	Both	All ages	Tracheal, bronchus, and lung cancer	All risk factors	Percent	2021	0.78582855	0.9289351	0.56357994
Deaths	Tianjin	Both	All ages	Tracheal, bronchus, and lung cancer	Particulate matter pollution	Percent	2021	0.28991414	0.38278822	0.19374212
Deaths	Hunan	Both	All ages	Tracheal, bronchus, and lung cancer	Particulate matter pollution	Percent	2021	0.2655851	0.36397027	0.17322699
Deaths	Tianjin	Both	All ages	Tracheal, bronchus, and lung cancer	Environmental/occupational risks	Percent	2021	0.35891963	0.45111611	0.25664711
Deaths	Guangxi	Both	All ages	Larynx cancer	Environmental/occupational risks	Percent	2021	0.05889467	0.09607641	0.03222492
Deaths	Guangxi	Both	All ages	Larynx cancer	Behavioral risks	Percent	2021	0.7578196	0.85382981	0.64801059
Deaths	Guangxi	Both	All ages	Tracheal, bronchus, and lung cancer	Occupational exposure to chromium	Percent	2021	0.00154292	0.0017909	0.00130493
Deaths	Inner Mongolia	Both	All ages	Larynx cancer	Environmental/occupational risks	Percent	2021	0.05444686	0.08824743	0.03113779
Deaths	Inner Mongolia	Both	All ages	Larynx cancer	Behavioral risks	Percent	2021	0.82318982	0.90278573	0.71771426
Deaths	Inner Mongolia	Both	All ages	Tracheal, bronchus, and lung cancer	Occupational exposure to chromium	Percent	2021	0.00120183	0.00141204	0.00101614
Deaths	Qinghai	Both	All ages	Larynx cancer	Smoking	Percent	2021	0.68466866	0.79373018	0.55897637

Deaths	Qinghai	Both	All ages	Larynx cancer	High alcohol use	Percent	2021	0.11053289	0.17216202	0.05235963
Deaths	Qinghai	Both	All ages	Larynx cancer	Occupational risks	Percent	2021	0.05589784	0.09279642	0.03070336
Deaths	Qinghai	Both	All ages	Larynx cancer	Occupational carcinogens	Percent	2021	0.05589784	0.09279642	0.03070336
Deaths	Qinghai	Both	All ages	Larynx cancer	Occupational exposure to asbestos	Percent	2021	0.01360061	0.02114291	0.00762903
Deaths	Guangxi	Both	All ages	Tracheal, bronchus, and lung cancer	Occupational exposure to diesel engine exhaust	Percent	2021	0.01632793	0.01897022	0.01386802
Deaths	Guangxi	Both	All ages	Tracheal, bronchus, and lung cancer	Occupational exposure to nickel	Percent	2021	0.00728637	0.01618876	0.00154284
Deaths	Guangxi	Both	All ages	Tracheal, bronchus, and lung cancer	Occupational exposure to polycyclic aromatic hydrocarbons	Percent	2021	0.00523195	0.00628612	0.00429586
Deaths	Guangxi	Both	All ages	Tracheal, bronchus, and lung cancer	Occupational exposure to silica	Percent	2021	0.03401049	0.05285653	0.0159997
Deaths	Guangxi	Both	All ages	Tracheal, bronchus, and lung cancer	All risk factors	Percent	2021	0.75658336	0.88627628	0.54352315
Deaths	Qinghai	Both	All ages	Tracheal, bronchus, and lung cancer	Occupational risks	Percent	2021	0.09938727	0.12908819	0.07530535
Deaths	Qinghai	Both	All ages	Tracheal, bronchus, and lung cancer	Occupational carcinogens	Percent	2021	0.09938727	0.12908819	0.07530535

Deaths	Qinghai	Both	All ages	Tracheal, bronchus, and lung cancer	Occupational exposure to asbestos	Percent	2021	0.04044607	0.0583658 6	0.0254668 9
Deaths	Qinghai	Both	All ages	Tracheal, bronchus, and lung cancer	Occupational exposure to arsenic	Percent	2021	0.0066279 3	0.0109888 9	0.0025496 1
Deaths	Qinghai	Both	All ages	Tracheal, bronchus, and lung cancer	Occupational exposure to beryllium	Percent	2021	0.0002373 9	0.0002806 3	0.0001958 3
Deaths	Qinghai	Both	All ages	Tracheal, bronchus, and lung cancer	Occupational exposure to cadmium	Percent	2021	0.0006426 3	0.0007864 1	0.0005179 5
Deaths	Hunan	Both	All ages	Larynx cancer	Environmental/occupational risks	Percent	2021	0.0573431 8	0.0911965 7	0.0329330 8
Deaths	Hunan	Both	All ages	Larynx cancer	Behavioral risks	Percent	2021	0.8030805 6	0.8886965 1	0.7022294 5
Deaths	Hunan	Both	All ages	Tracheal, bronchus, and lung cancer	Occupational exposure to chromium	Percent	2021	0.0014137 1	0.0016505 4	0.0011938 1
Deaths	Guizhou	Both	All ages	Tracheal, bronchus, and lung cancer	Residential radon	Percent	2021	0.0380056 5	0.1357373 8	-0.0150104
Deaths	Guizhou	Both	All ages	Tracheal, bronchus, and lung cancer	Behavioral risks	Percent	2021	0.6583761 3	0.8527625 9	0.3299765 6
Deaths	Beijing	Both	All ages	Tracheal, bronchus, and lung cancer	Air pollution	Percent	2021	0.2819194 3	0.3716013 9	0.1883717 8
Deaths	Beijing	Both	All ages	Tracheal, bronchus, and lung cancer	Ambient particulate matter pollution	Percent	2021	0.2806816 5	0.3704503 2	0.1869828 1

Deaths	Beijing	Both	All ages	Tracheal, bronchus, and lung cancer	Household air pollution from solid fuels	Percent	2021	0.00120338	0.0113546 5	2.60E-07
Deaths	Beijing	Both	All ages	Tracheal, bronchus, and lung cancer	Other environmental risks	Percent	2021	0.0298883 8	0.0977709 8	-0.0128287
Deaths	Guizhou	Both	All ages	Tracheal, bronchus, and lung cancer	Particulate matter pollution	Percent	2021	0.2504092 8	0.3500057 9	0.1586745 1
Deaths	Ningxia	Both	All ages	Larynx cancer	Environmental/occupational risks	Percent	2021	0.0475141 2	0.0819695 9	0.0221089 7
Deaths	Ningxia	Both	All ages	Larynx cancer	Behavioral risks	Percent	2021	0.716551	0.8152128 8	0.5934523
Deaths	China	Both	All ages	Larynx cancer	Environmental/occupational risks	Percent	2021	0.0517584 8	0.0855219 7	0.0281666 9
Deaths	China	Both	All ages	Larynx cancer	Behavioral risks	Percent	2021	0.7889065 3	0.8640829 5	0.6897392 5
Deaths	Guangxi	Both	All ages	Tracheal, bronchus, and lung cancer	Environmental/occupational risks	Percent	2021	0.3556685 2	0.4484251 7	0.2559466 6
Deaths	Ningxia	Both	All ages	Tracheal, bronchus, and lung cancer	Occupational exposure to diesel engine exhaust	Percent	2021	0.0141751 8	0.0162955 1	0.0122262 3
Deaths	Ningxia	Both	All ages	Tracheal, bronchus, and lung cancer	Occupational exposure to nickel	Percent	2021	0.0063835 6	0.0142368 3	0.0013314 2
Deaths	Ningxia	Both	All ages	Tracheal, bronchus, and lung cancer	Occupational exposure to polycyclic aromatic hydrocarbons	Percent	2021	0.0046084 1	0.0055101 1	0.0037726 1

Deaths	Ningxia	Both	All ages	Tracheal, bronchus, and lung cancer	Occupational exposure to silica	Percent	2021	0.02958096	0.04648245	0.01391657
Deaths	Ningxia	Both	All ages	Tracheal, bronchus, and lung cancer	All risk factors	Percent	2021	0.74389642	0.89512206	0.51350832
Deaths	Hunan	Both	All ages	Tracheal, bronchus, and lung cancer	Occupational exposure to diesel engine exhaust	Percent	2021	0.01515755	0.01753555	0.01275442
Deaths	Hunan	Both	All ages	Tracheal, bronchus, and lung cancer	Occupational exposure to nickel	Percent	2021	0.00663671	0.01490272	0.00136441
Deaths	Hunan	Both	All ages	Tracheal, bronchus, and lung cancer	Occupational exposure to polycyclic aromatic hydrocarbons	Percent	2021	0.00479079	0.00582838	0.00389576
Deaths	Hunan	Both	All ages	Tracheal, bronchus, and lung cancer	Occupational exposure to silica	Percent	2021	0.03123784	0.05012837	0.01479215
Deaths	Hunan	Both	All ages	Tracheal, bronchus, and lung cancer	All risk factors	Percent	2021	0.81109158	0.92288231	0.59280193
Deaths	Ningxia	Both	All ages	Tracheal, bronchus, and lung cancer	Environmental/occupational risks	Percent	2021	0.33642164	0.45812768	0.23216504
Deaths	Hunan	Both	All ages	Tracheal, bronchus, and lung cancer	Environmental/occupational risks	Percent	2021	0.36231806	0.45270478	0.27207367
Deaths	Guizhou	Both	All ages	Larynx cancer	Environmental/occupational risks	Percent	2021	0.06123721	0.09869017	0.03395836
Deaths	Guizhou	Both	All ages	Larynx cancer	Behavioral risks	Percent	2021	0.77672801	0.88319102	0.65103944

Deaths	Guizhou	Both	All ages	Tracheal, bronchus, and lung cancer	Occupational exposure to chromium	Percent	2021	0.0014494 2	0.0017306 9	0.0012074 8
Deaths	Guizhou	Both	All ages	Tracheal, bronchus, and lung cancer	Occupational exposure to diesel engine exhaust	Percent	2021	0.0143623 9	0.0166473 6	0.0121931 6
Deaths	Guizhou	Both	All ages	Tracheal, bronchus, and lung cancer	Occupational exposure to nickel	Percent	2021	0.0070662 8	0.0155194 1	0.0014146 4
Deaths	Guizhou	Both	All ages	Tracheal, bronchus, and lung cancer	Occupational exposure to polycyclic aromatic hydrocarbons	Percent	2021	0.0049321 2	0.0059652 8	0.0040910 6
Deaths	Guizhou	Both	All ages	Tracheal, bronchus, and lung cancer	Occupational exposure to silica	Percent	2021	0.0325312	0.0508647 8	0.0144791 7
Deaths	Guizhou	Both	All ages	Tracheal, bronchus, and lung cancer	All risk factors	Percent	2021	0.7863529	0.9116820 7	0.5752608
Deaths	Guizhou	Both	All ages	Tracheal, bronchus, and lung cancer	Environmental/occupational risks	Percent	2021	0.3601458	0.4640691 5	0.2619010 3
Deaths	Beijing	Both	All ages	Tracheal, bronchus, and lung cancer	Residential radon	Percent	2021	0.0298883 8	0.0977709 8	-0.0128287
Deaths	Beijing	Both	All ages	Tracheal, bronchus, and lung cancer	Behavioral risks	Percent	2021	0.6516828 3	0.8733187 3	0.2968537 1

Appendix 25：Percentage contribution of risk factors of larynx cancer,2021

measure	location	sex	age	cause	rei	metric	year	val	upper	lower
Deaths	Macao Special Administrative Region of China	Both	All ages	Larynx cancer	Occupational exposure to sulfuric acid	Percent	2021	0.0438037 8	0.0810383 7	0.0187843 6
Deaths	Macao Special Administrative Region of China	Both	All ages	Larynx cancer	All risk factors	Percent	2021	0.8305112 5	0.8985513 3	0.7348803 5
Deaths	Macao Special Administrative Region of China	Both	All ages	Larynx cancer	Environmental/occupational risks	Percent	2021	0.0513772 7	0.0890742 5	0.0268109
Deaths	Macao Special Administrative Region of China	Both	All ages	Larynx cancer	Behavioral risks	Percent	2021	0.8220567 3	0.8932928 4	0.7247173 2
Deaths	Hainan	Both	All ages	Larynx cancer	Tobacco	Percent	2021	0.7560132 8	0.8610391 6	0.6359824 3
Deaths	Tibet	Both	All ages	Larynx cancer	Occupational exposure to sulfuric acid	Percent	2021	0.0470436 2	0.0882536 3	0.0191477 3
Deaths	Tibet	Both	All ages	Larynx cancer	All risk factors	Percent	2021	0.7335328 8	0.8459163 6	0.5818683 8
Deaths	Hainan	Both	All ages	Larynx cancer	Smoking	Percent	2021	0.7560	0.8610	0.6359

								1328	3916	8243
Deaths	Hainan	Both	All ages	Larynx cancer	High alcohol use	Percent	2021	0.14996792	0.2242 1991	0.07450194
Deaths	Hainan	Both	All ages	Larynx cancer	Occupational risks	Percent	2021	0.04442569	0.07876144	0.02128658
Deaths	Hainan	Both	All ages	Larynx cancer	Occupational carcinogens	Percent	2021	0.04442569	0.07876144	0.02128658
Deaths	Hainan	Both	All ages	Larynx cancer	Occupational exposure to asbestos	Percent	2021	0.00463135	0.00840409	0.00215133
Deaths	Chongqing	Both	All ages	Larynx cancer	Occupational exposure to sulfuric acid	Percent	2021	0.04073061	0.07558784	0.01710966
Deaths	Chongqing	Both	All ages	Larynx cancer	All risk factors	Percent	2021	0.7995097	0.87369375	0.70676603
Deaths	Chongqing	Both	All ages	Larynx cancer	Environmental/occupational risks	Percent	2021	0.04629317	0.08107849	0.02313472
Deaths	Chongqing	Both	All ages	Larynx cancer	Behavioral risks	Percent	2021	0.78865042	0.86687155	0.69123832

Deaths	Shaanxi	Both	All ages	Larynx cancer	Occupational exposure to sulfuric acid	Percent	2021	0.0395604	0.07207217	0.0163665
Deaths	Shaanxi	Both	All ages	Larynx cancer	All risk factors	Percent	2021	0.78626355	0.86865125	0.68861682
Deaths	Macao Special Administrative Region of China	Both	All ages	Larynx cancer	Tobacco	Percent	2021	0.79166815	0.8736045	0.68016653
Deaths	Tibet	Both	All ages	Larynx cancer	Tobacco	Percent	2021	0.68940681	0.81900147	0.52009547
Deaths	Hainan	Both	All ages	Larynx cancer	Occupational exposure to sulfuric acid	Percent	2021	0.03988958	0.07388933	0.01697529
Deaths	Hainan	Both	All ages	Larynx cancer	All risk factors	Percent	2021	0.78863738	0.88358474	0.68563717
Deaths	Shaanxi	Both	All ages	Larynx cancer	Environmental/occupational risks	Percent	2021	0.05267644	0.08513987	0.02937669
Deaths	Shaanxi	Both	All ages	Larynx cancer	Behavioral risks	Percent	2021	0.77388261	0.86063632	0.6755798
Deaths	Tibet	Both	All ages	Larynx cancer	Environmental/occupational risks	Percent	2021	0.0639	0.1058	0.0344

								5148	3828	1865
Deaths	Tibet	Both	All ages	Larynx cancer	Behavioral risks	Percent	2021	0.7154793 7	0.8361345 9	0.5464623 2
Deaths	Tibet	Both	All ages	Larynx cancer	Smoking	Percent	2021	0.6894068 1	0.8190014 7	0.5200954 7
Deaths	Tibet	Both	All ages	Larynx cancer	High alcohol use	Percent	2021	0.0976199 8	0.1689174 4	0.0335995 3
Deaths	Tibet	Both	All ages	Larynx cancer	Occupational risks	Percent	2021	0.0639514 8	0.1058382 8	0.0344186 5
Deaths	Tibet	Both	All ages	Larynx cancer	Occupational carcinogens	Percent	2021	0.0639514 8	0.1058382 8	0.0344186 5
Deaths	Tibet	Both	All ages	Larynx cancer	Occupational exposure to asbestos	Percent	2021	0.0176265 3	0.0302430 2	0.0087083 3
Deaths	Hainan	Both	All ages	Larynx cancer	Environmental/occupational risks	Percent	2021	0.0444256 9	0.0787614 4	0.0212865 8
Deaths	Hainan	Both	All ages	Larynx cancer	Behavioral risks	Percent	2021	0.7787474 1	0.8787764 4	0.6694604 1

Deaths	Macao Special Administrative Region of China	Both	All ages	Larynx cancer	Smoking	Percent	2021	0.7916681 5	0.8736045	0.68016653
Deaths	Macao Special Administrative Region of China	Both	All ages	Larynx cancer	High alcohol use	Percent	2021	0.19836128	0.28579047	0.09609158
Deaths	Macao Special Administrative Region of China	Both	All ages	Larynx cancer	Occupational risks	Percent	2021	0.05137727	0.08907425	0.0268109
Deaths	Macao Special Administrative Region of China	Both	All ages	Larynx cancer	Occupational carcinogens	Percent	2021	0.05137727	0.08907425	0.0268109
Deaths	Macao Special Administrative Region of China	Both	All ages	Larynx cancer	Occupational exposure to asbestos	Percent	2021	0.00779314	0.01399455	0.00336076
Deaths	Shaanxi	Both	All ages	Larynx cancer	Tobacco	Percent	2021	0.75697284	0.84837851	0.64679249
Deaths	Shandong	Both	All ages	Larynx cancer	Occupational exposure to sulfuric acid	Percent	2021	0.0432786	0.07732657	0.0181093
Deaths	Shandong	Both	All ages	Larynx cancer	All risk factors	Percent	2021	0.80892879	0.89012935	0.70467616
Deaths	Chongqing	Both	All ages	Larynx cancer	Tobacco	Percent	2021	0.7703	0.8562	0.6651

								9632	4731	2836
Deaths	Chongqing	Both	All ages	Larynx cancer	Smoking	Percent	2021	0.7703963 2	0.8562473 1	0.6651283 6
Deaths	Chongqing	Both	All ages	Larynx cancer	High alcohol use	Percent	2021	0.1383085 2	0.2001980 2	0.0725622 1
Deaths	Chongqing	Both	All ages	Larynx cancer	Occupational risks	Percent	2021	0.0462931 7	0.0810784 9	0.0231347 2
Deaths	Chongqing	Both	All ages	Larynx cancer	Occupational carcinogens	Percent	2021	0.0462931 7	0.0810784 9	0.0231347 2
Deaths	Chongqing	Both	All ages	Larynx cancer	Occupational exposure to asbestos	Percent	2021	0.0056935 1	0.0096872 7	0.0029330 9
Deaths	Shaanxi	Both	All ages	Larynx cancer	Smoking	Percent	2021	0.7569728 4	0.8483785 1	0.6467924 9
Deaths	Shaanxi	Both	All ages	Larynx cancer	High alcohol use	Percent	2021	0.1241591 4	0.1848086 5	0.0602882 4
Deaths	Shaanxi	Both	All ages	Larynx cancer	Occupational risks	Percent	2021	0.0526764 4	0.0851398 7	0.0293766 9

Deaths	Shaanxi	Both	All ages	Larynx cancer	Occupational carcinogens	Percent	2021	0.05267644	0.08513987	0.02937669
Deaths	Shaanxi	Both	All ages	Larynx cancer	Occupational exposure to asbestos	Percent	2021	0.01344497	0.02147629	0.00723962
Deaths	Hebei	Both	All ages	Larynx cancer	Occupational exposure to sulfuric acid	Percent	2021	0.04346569	0.0783972	0.01788988
Deaths	Hebei	Both	All ages	Larynx cancer	All risk factors	Percent	2021	0.75501007	0.84699844	0.64396499
Deaths	Jiangsu	Both	All ages	Larynx cancer	Occupational exposure to sulfuric acid	Percent	2021	0.03626284	0.06673387	0.0149367
Deaths	Jiangsu	Both	All ages	Larynx cancer	All risk factors	Percent	2021	0.8120116	0.89647719	0.70379765
Deaths	Fujian	Both	All ages	Larynx cancer	Tobacco	Percent	2021	0.77032914	0.85237591	0.65647527
Deaths	Shandong	Both	All ages	Larynx cancer	Environmental/occupational risks	Percent	2021	0.05121539	0.08656221	0.02616996
Deaths	Shandong	Both	All ages	Larynx cancer	Behavioral risks	Percent	2021	0.7982	0.8826	0.6881

								5602	2388	6507
Deaths	Hebei	Both	All ages	Larynx cancer	Environmental/occupational risks	Percent	2021	0.06374527	0.10134062	0.03624711
Deaths	Hebei	Both	All ages	Larynx cancer	Behavioral risks	Percent	2021	0.73814942	0.83698077	0.62319441
Deaths	Fujian	Both	All ages	Larynx cancer	Occupational exposure to sulfuric acid	Percent	2021	0.04047303	0.07444227	0.01641811
Deaths	Fujian	Both	All ages	Larynx cancer	All risk factors	Percent	2021	0.80072839	0.87316322	0.69877168
Deaths	Jiangsu	Both	All ages	Larynx cancer	Environmental/occupational risks	Percent	2021	0.04227045	0.07434537	0.0216147
Deaths	Jiangsu	Both	All ages	Larynx cancer	Behavioral risks	Percent	2021	0.80335729	0.89270411	0.68968671
Deaths	Taiwan (Province of China)	Both	All ages	Larynx cancer	Occupational exposure to sulfuric acid	Percent	2021	0.04033692	0.07234821	0.01696181
Deaths	Taiwan (Province of China)	Both	All ages	Larynx cancer	All risk factors	Percent	2021	0.80123933	0.87705065	0.70099887

Deaths	Jiangsu	Both	All ages	Larynx cancer	Tobacco	Percent	2021	0.7796410 1	0.8776688 9	0.6522999 3
Deaths	Fujian	Both	All ages	Larynx cancer	Environmental/occupational risks	Percent	2021	0.0442331 6	0.0784263 4	0.0204408 1
Deaths	Fujian	Both	All ages	Larynx cancer	Behavioral risks	Percent	2021	0.7919747	0.8660774 6	0.6864019
Deaths	Taiwan (Province of China)	Both	All ages	Larynx cancer	Environmental/occupational risks	Percent	2021	0.0544418 9	0.0886539 5	0.0292044 8
Deaths	Taiwan (Province of China)	Both	All ages	Larynx cancer	Behavioral risks	Percent	2021	0.7906879 3	0.8703698 6	0.6866830 5
Deaths	Shanghai	Both	All ages	Larynx cancer	Occupational exposure to sulfuric acid	Percent	2021	0.0267712 1	0.0495857 6	0.0106790 5
Deaths	Shanghai	Both	All ages	Larynx cancer	All risk factors	Percent	2021	0.8129035 8	0.8964135 9	0.7122765 5
Deaths	Jiangsu	Both	All ages	Larynx cancer	Smoking	Percent	2021	0.7796410 1	0.8776688 9	0.6522999 3
Deaths	Jiangsu	Both	All ages	Larynx cancer	High alcohol use	Percent	2021	0.1563	0.2348	0.0774

								1652	2592	0958
Deaths	Jiangsu	Both	All ages	Larynx cancer	Occupational risks	Percent	2021	0.0422704 5	0.0743453 7	0.0216147
Deaths	Jiangsu	Both	All ages	Larynx cancer	Occupational carcinogens	Percent	2021	0.04227045	0.07434537	0.0216147
Deaths	Jiangsu	Both	All ages	Larynx cancer	Occupational exposure to asbestos	Percent	2021	0.00609448	0.01041776	0.00311351
Deaths	Xinjiang	Both	All ages	Larynx cancer	Occupational exposure to sulfuric acid	Percent	2021	0.04163493	0.07734146	0.01683701
Deaths	Xinjiang	Both	All ages	Larynx cancer	All risk factors	Percent	2021	0.72466726	0.82865726	0.60244469
Deaths	Xinjiang	Both	All ages	Larynx cancer	Environmental/occupational risks	Percent	2021	0.0511553	0.08701846	0.0271938
Deaths	Xinjiang	Both	All ages	Larynx cancer	Behavioral risks	Percent	2021	0.70973454	0.81991717	0.57979632
Deaths	Shanghai	Both	All ages	Larynx cancer	Environmental/occupational risks	Percent	2021	0.03810634	0.06233847	0.02117258

Deaths	Shanghai	Both	All ages	Larynx cancer	Behavioral risks	Percent	2021	0.805873332	0.892947 7	0.70376856
Deaths	Hebei	Both	All ages	Larynx cancer	Tobacco	Percent	2021	0.71431268	0.81876992	0.58385614
Deaths	Fujian	Both	All ages	Larynx cancer	Smoking	Percent	2021	0.77032914	0.85237591	0.65647527
Deaths	Fujian	Both	All ages	Larynx cancer	High alcohol use	Percent	2021	0.1538038	0.2310358	0.07878475
Deaths	Fujian	Both	All ages	Larynx cancer	Occupational risks	Percent	2021	0.04423316	0.07842634	0.02044081
Deaths	Fujian	Both	All ages	Larynx cancer	Occupational carcinogens	Percent	2021	0.04423316	0.07842634	0.02044081
Deaths	Fujian	Both	All ages	Larynx cancer	Occupational exposure to asbestos	Percent	2021	0.00383466	0.00671581	0.00181254
Deaths	Jiangxi	Both	All ages	Larynx cancer	Occupational exposure to sulfuric acid	Percent	2021	0.03992339	0.0761884	0.01633642
Deaths	Jiangxi	Both	All ages	Larynx cancer	All risk factors	Percent	2021	0.7751	0.8660	0.6763

								577	4851	107
Deaths	Xinjiang	Both	All ages	Larynx cancer	Tobacco	Percent	2021	0.6816976 5	0.7989699 1	0.5406138 6
Deaths	Taiwan (Province of China)	Both	All ages	Larynx cancer	Tobacco	Percent	2021	0.7678065 3	0.8570919 7	0.6475193 2
Deaths	Xinjiang	Both	All ages	Larynx cancer	Smoking	Percent	2021	0.6816976 5	0.7989699 1	0.5406138 6
Deaths	Xinjiang	Both	All ages	Larynx cancer	High alcohol use	Percent	2021	0.1236909 7	0.1835376 8	0.0634337 5
Deaths	Xinjiang	Both	All ages	Larynx cancer	Occupational risks	Percent	2021	0.0511553	0.0870184 6	0.0271938
Deaths	Xinjiang	Both	All ages	Larynx cancer	Occupational carcinogens	Percent	2021	0.0511553	0.0870184 6	0.0271938
Deaths	Xinjiang	Both	All ages	Larynx cancer	Occupational exposure to asbestos	Percent	2021	0.0098012 1	0.0161621	0.0053516 5
Deaths	Jiangxi	Both	All ages	Larynx cancer	Environmental/occupational risks	Percent	2021	0.0496547 9	0.0852977 1	0.0267392

Deaths	Jiangxi	Both	All ages	Larynx cancer	Behavioral risks	Percent	2021	0.7633585848	0.8604873131	0.6613100404
Deaths	Shandong	Both	All ages	Larynx cancer	Tobacco	Percent	2021	0.7737987878	0.8680787896	0.65404058
Deaths	Yunnan	Both	All ages	Larynx cancer	Occupational exposure to sulfuric acid	Percent	2021	0.0462367878	0.0861318383	0.0195854848
Deaths	Yunnan	Both	All ages	Larynx cancer	All risk factors	Percent	2021	0.7973893333	0.8684941212	0.7089553131
Deaths	Heilongjiang	Both	All ages	Larynx cancer	Occupational exposure to sulfuric acid	Percent	2021	0.0343842929	0.0633861616	0.0138080886
Deaths	Heilongjiang	Both	All ages	Larynx cancer	All risk factors	Percent	2021	0.8328849292	0.9066252727	0.7276458484
Deaths	Yunnan	Both	All ages	Larynx cancer	Environmental/occupational risks	Percent	2021	0.063221 9	0.104517 8	0.0353675656
Deaths	Yunnan	Both	All ages	Larynx cancer	Behavioral risks	Percent	2021	0.7827020404	0.8589654444	0.6880864848
Deaths	Heilongjiang	Both	All ages	Larynx cancer	Environmental/occupational risks	Percent	2021	0.0472	0.0769	0.0262

								0433	0137	0239
Deaths	Heilongjiang	Both	All ages	Larynx cancer	Behavioral risks	Percent	2021	0.8248290 7	0.9029778 4	0.7156355 9
Deaths	Zhejiang	Both	All ages	Larynx cancer	Occupational exposure to sulfuric acid	Percent	2021	0.0396296 7	0.0728734	0.0156055 8
Deaths	Zhejiang	Both	All ages	Larynx cancer	All risk factors	Percent	2021	0.8310780 7	0.8984614 4	0.7311587
Deaths	Heilongjiang	Both	All ages	Larynx cancer	Tobacco	Percent	2021	0.7999453 3	0.8895236 1	0.6728231 1
Deaths	Zhejiang	Both	All ages	Larynx cancer	Environmental/occupational risks	Percent	2021	0.0466294 2	0.0803600 1	0.0238447 6
Deaths	Zhejiang	Both	All ages	Larynx cancer	Behavioral risks	Percent	2021	0.8231823 1	0.8940507 9	0.7172533 7
Deaths	Jilin	Both	All ages	Larynx cancer	Occupational exposure to sulfuric acid	Percent	2021	0.0351089 2	0.0655054 9	0.0141670 4
Deaths	Jilin	Both	All ages	Larynx cancer	All risk factors	Percent	2021	0.7722970 2	0.8720810 7	0.6529540 6

Deaths	Gansu	Both	All ages	Larynx cancer	Tobacco	Percent	2021	0.75001195	0.83575858	0.63231912
Deaths	Jilin	Both	All ages	Larynx cancer	Environmental/occupational risks	Percent	2021	0.05312401	0.08389695	0.03085885
Deaths	Jilin	Both	All ages	Larynx cancer	Behavioral risks	Percent	2021	0.75906304	0.86545239	0.63505824
Deaths	Yunnan	Both	All ages	Larynx cancer	Tobacco	Percent	2021	0.76661661	0.8471209	0.67278868
Deaths	Henan	Both	All ages	Larynx cancer	Tobacco	Percent	2021	0.70693863	0.81229831	0.57380863
Deaths	Jiangxi	Both	All ages	Larynx cancer	Tobacco	Percent	2021	0.74125995	0.84605905	0.62904055
Deaths	Jilin	Both	All ages	Larynx cancer	Tobacco	Percent	2021	0.73750239	0.84910498	0.59927356
Deaths	Hebei	Both	All ages	Larynx cancer	Smoking	Percent	2021	0.71431268	0.81876992	0.58385614
Deaths	Hebei	Both	All ages	Larynx cancer	High alcohol use	Percent	2021	0.1383	0.2079	0.0709

								1785	3516	3207
Deaths	Hebei	Both	All ages	Larynx cancer	Occupational risks	Percent	2021	0.06374527	0.10134062	0.03624711
Deaths	Hebei	Both	All ages	Larynx cancer	Occupational carcinogens	Percent	2021	0.06374527	0.10134062	0.03624711
Deaths	Hebei	Both	All ages	Larynx cancer	Occupational exposure to asbestos	Percent	2021	0.02086897	0.03302817	0.0110232
Deaths	Taiwan (Province of China)	Both	All ages	Larynx cancer	Smoking	Percent	2021	0.76780653	0.85709197	0.64751932
Deaths	Taiwan (Province of China)	Both	All ages	Larynx cancer	High alcohol use	Percent	2021	0.1185457	0.17410317	0.06172071
Deaths	Taiwan (Province of China)	Both	All ages	Larynx cancer	Occupational risks	Percent	2021	0.05444189	0.08865395	0.02920448
Deaths	Taiwan (Province of China)	Both	All ages	Larynx cancer	Occupational carcinogens	Percent	2021	0.05444189	0.08865395	0.02920448
Deaths	Taiwan (Province of China)	Both	All ages	Larynx cancer	Occupational exposure to asbestos	Percent	2021	0.0145426	0.02695408	0.00653111

Deaths	Shanxi	Both	All ages	Larynx cancer	Occupational exposure to sulfuric acid	Percent	2021	0.0399182 5	0.0730327 2	0.0165452 6
Deaths	Shanxi	Both	All ages	Larynx cancer	All risk factors	Percent	2021	0.7989083 9	0.8825059 1	0.6988656 2
Deaths	Shanxi	Both	All ages	Larynx cancer	Environmental/occupational risks	Percent	2021	0.0519816	0.0857706 2	0.0285044 7
Deaths	Shanxi	Both	All ages	Larynx cancer	Behavioral risks	Percent	2021	0.7881945 6	0.8758718 8	0.6836268 8
Deaths	Henan	Both	All ages	Larynx cancer	Occupational exposure to sulfuric acid	Percent	2021	0.0444390 5	0.0803716	0.0182466 4
Deaths	Henan	Both	All ages	Larynx cancer	All risk factors	Percent	2021	0.7434066 7	0.8353008 3	0.6304707 7
Deaths	Shandong	Both	All ages	Larynx cancer	Smoking	Percent	2021	0.7737987 8	0.8680789 6	0.654058
Deaths	Shandong	Both	All ages	Larynx cancer	High alcohol use	Percent	2021	0.1567575 4	0.2290319 1	0.0820050 5
Deaths	Shandong	Both	All ages	Larynx cancer	Occupational risks	Percent	2021	0.0512	0.0865	0.0261

								1539	6221	6996
Deaths	Shandong	Both	All ages	Larynx cancer	Occupational carcinogens	Percent	2021	0.0512151539	0.0865626221	0.0261696996
Deaths	Shandong	Both	All ages	Larynx cancer	Occupational exposure to asbestos	Percent	2021	0.0081393956	0.0135045858	0.0043023 9
Deaths	Gansu	Both	All ages	Larynx cancer	Occupational exposure to sulfuric acid	Percent	2021	0.0421507 6	0.0782235 1	0.0175637 5
Deaths	Gansu	Both	All ages	Larynx cancer	All risk factors	Percent	2021	0.7770767 3	0.8541022 4	0.6649498 1
Deaths	Shanghai	Both	All ages	Larynx cancer	Tobacco	Percent	2021	0.7785856 2	0.8767506 8	0.6578180 8
Deaths	Zhejiang	Both	All ages	Larynx cancer	Tobacco	Percent	2021	0.7980500 8	0.8774386 4	0.6832823 5
Deaths	Shanxi	Both	All ages	Larynx cancer	Tobacco	Percent	2021	0.7719457 5	0.8636343 7	0.6580372 6
Deaths	Liaoning	Both	All ages	Larynx cancer	Tobacco	Percent	2021	0.782486 4	0.8750556 1	0.6619768 6

Deaths	Anhui	Both	All ages	Larynx cancer	Tobacco	Percent	2021	0.7851476 7	0.87303031	0.67546124
Deaths	Liaoning	Both	All ages	Larynx cancer	Occupational exposure to sulfuric acid	Percent	2021	0.0340425 7	0.06199479	0.01393453
Deaths	Liaoning	Both	All ages	Larynx cancer	All risk factors	Percent	2021	0.8168645 5	0.89456955	0.71450083
Deaths	Hubei	Both	All ages	Larynx cancer	Tobacco	Percent	2021	0.756495	0.8437959 5	0.6402704
Deaths	Heilongjiang	Both	All ages	Larynx cancer	Smoking	Percent	2021	0.7999453 3	0.88952361	0.67282311
Deaths	Heilongjiang	Both	All ages	Larynx cancer	High alcohol use	Percent	2021	0.1515714 2	0.2250183	0.07754219
Deaths	Heilongjiang	Both	All ages	Larynx cancer	Occupational risks	Percent	2021	0.0472043 3	0.07690137	0.02620239
Deaths	Heilongjiang	Both	All ages	Larynx cancer	Occupational carcinogens	Percent	2021	0.0472043 3	0.07690137	0.02620239
Deaths	Heilongjiang	Both	All ages	Larynx cancer	Occupational exposure to asbestos	Percent	2021	0.0131	0.0212	0.0072

								1531	8396	1477
Deaths	Inner Mongolia	Both	All ages	Larynx cancer	Tobacco	Percent	2021	0.8033081 4	0.88762 7	0.68948139
Deaths	Guangdong	Both	All ages	Larynx cancer	Occupational exposure to sulfuric acid	Percent	2021	0.04054614	0.07477192	0.01615543
Deaths	Guangdong	Both	All ages	Larynx cancer	All risk factors	Percent	2021	0.82560245	0.89626086	0.73565047
Deaths	Hubei	Both	All ages	Larynx cancer	Occupational exposure to sulfuric acid	Percent	2021	0.03817725	0.06963587	0.01603433
Deaths	Hubei	Both	All ages	Larynx cancer	All risk factors	Percent	2021	0.78944766	0.8645247	0.69561303
Deaths	Beijing	Both	All ages	Larynx cancer	Tobacco	Percent	2021	0.78502389	0.87940453	0.65961407
Deaths	Ningxia	Both	All ages	Larynx cancer	Tobacco	Percent	2021	0.69692323	0.80230191	0.56531597
Deaths	Tianjin	Both	All ages	Larynx cancer	Tobacco	Percent	2021	0.78133099	0.86958502	0.66346733

Deaths	Sichuan	Both	All ages	Larynx cancer	Tobacco	Percent	2021	0.7801763 3	0.8676710 6	0.6724374 7
Deaths	Jiangxi	Both	All ages	Larynx cancer	Smoking	Percent	2021	0.7412599 5	0.8460590 5	0.6290405 5
Deaths	Jiangxi	Both	All ages	Larynx cancer	High alcohol use	Percent	2021	0.1400164 6	0.2131910 9	0.0689819 2
Deaths	Jiangxi	Both	All ages	Larynx cancer	Occupational risks	Percent	2021	0.0496547 9	0.0852977 1	0.0267392
Deaths	Jiangxi	Both	All ages	Larynx cancer	Occupational carcinogens	Percent	2021	0.0496547 9	0.0852977 1	0.0267392
Deaths	Jiangxi	Both	All ages	Larynx cancer	Occupational exposure to asbestos	Percent	2021	0.0099621 9	0.0164308 2	0.0052108 1
Deaths	Guangdong	Both	All ages	Larynx cancer	Tobacco	Percent	2021	0.7938453 7	0.8739267 3	0.6874174 6
Deaths	Sichuan	Both	All ages	Larynx cancer	Occupational exposure to sulfuric acid	Percent	2021	0.0432640 3	0.0790543 5	0.0180526 4
Deaths	Sichuan	Both	All ages	Larynx cancer	All risk factors	Percent	2021	0.8113	0.8869	0.7167

								9405	7345	1705
Deaths	Hong Kong Special Administrative Region of China	Both	All ages	Larynx cancer	Tobacco	Percent	2021	0.6158762 5	0.7682324 1	0.4442708 6
Deaths	Yunnan	Both	All ages	Larynx cancer	Smoking	Percent	2021	0.7666166 1	0.8471209	0.6727886 8
Deaths	Yunnan	Both	All ages	Larynx cancer	High alcohol use	Percent	2021	0.1309910 4	0.2083346 7	0.0589047 5
Deaths	Yunnan	Both	All ages	Larynx cancer	Occupational risks	Percent	2021	0.0632219	0.1045178	0.0353675 6
Deaths	Yunnan	Both	All ages	Larynx cancer	Occupational carcinogens	Percent	2021	0.0632219	0.1045178	0.0353675 6
Deaths	Yunnan	Both	All ages	Larynx cancer	Occupational exposure to asbestos	Percent	2021	0.0175482 5	0.0273467 2	0.0095276 2
Deaths	Gansu	Both	All ages	Larynx cancer	Smoking	Percent	2021	0.7500119 5	0.8357585 8	0.6323191 2
Deaths	Gansu	Both	All ages	Larynx cancer	High alcohol use	Percent	2021	0.0989143 2	0.1540348 3	0.0473818 9

Deaths	Gansu	Both	All ages	Larynx cancer	Occupational risks	Percent	2021	0.0567676745	0.0930626969	0.0315030311
Deaths	Gansu	Both	All ages	Larynx cancer	Occupational carcinogens	Percent	2021	0.0567676745	0.0930626969	0.0315030311
Deaths	Gansu	Both	All ages	Larynx cancer	Occupational exposure to asbestos	Percent	2021	0.0150346 1	0.0244207 9	0.0080414 8
Deaths	Hunan	Both	All ages	Larynx cancer	Tobacco	Percent	2021	0.78578 65	0.8771898 7	0.6690177
Deaths	Guangxi	Both	All ages	Larynx cancer	Tobacco	Percent	2021	0.7309712 6	0.8359448 8	0.6133281 5
Deaths	Zhejiang	Both	All ages	Larynx cancer	Smoking	Percent	2021	0.7980500 8	0.8774386 4	0.6832823 5
Deaths	Zhejiang	Both	All ages	Larynx cancer	High alcohol use	Percent	2021	0.1747541	0.2569972 8	0.0937643
Deaths	Zhejiang	Both	All ages	Larynx cancer	Occupational risks	Percent	2021	0.0466294 2	0.0803600 1	0.0238447 6
Deaths	Zhejiang	Both	All ages	Larynx cancer	Occupational carcinogens	Percent	2021	0.0466	0.0803	0.0238

								2942	6001	4476
Deaths	Zhejiang	Both	All ages	Larynx cancer	Occupational exposure to asbestos	Percent	2021	0.0071 3226	0.0125 9458	0.0035 0434
Deaths	China	Both	All ages	Larynx cancer	All risk factors	Percent	2021	0.8001 2349	0.8705 1487	0.7102 3748
Deaths	Henan	Both	All ages	Larynx cancer	Environmental/occupational risks	Percent	2021	0.0535 8665	0.0897 132	0.0278 2449
Deaths	Henan	Both	All ages	Larynx cancer	Behavioral risks	Percent	2021	0.7278 3591	0.8264 8104	0.6094 3272
Deaths	China	Both	All ages	Larynx cancer	Tobacco	Percent	2021	0.7669 9656	0.8514 9558	0.6545 722
Deaths	China	Both	All ages	Larynx cancer	Smoking	Percent	2021	0.7669 9656	0.8514 9558	0.6545 722
Deaths	Jilin	Both	All ages	Larynx cancer	Smoking	Percent	2021	0.7375 0239	0.8491 0498	0.5992 7356
Deaths	Jilin	Both	All ages	Larynx cancer	High alcohol use	Percent	2021	0.1318 7926	0.2067 1963	0.0665 4823

Deaths	Jilin	Both	All ages	Larynx cancer	Occupational risks	Percent	2021	0.0531240 1	0.0838969 5	0.0308588 5
Deaths	Jilin	Both	All ages	Larynx cancer	Occupational carcinogens	Percent	2021	0.0531240 1	0.0838969 5	0.0308588 5
Deaths	Jilin	Both	All ages	Larynx cancer	Occupational exposure to asbestos	Percent	2021	0.0184432 1	0.0284804 4	0.0098855 1
Deaths	Liaoning	Both	All ages	Larynx cancer	Smoking	Percent	2021	0.7824864	0.8750556 1	0.6619768 6
Deaths	Liaoning	Both	All ages	Larynx cancer	High alcohol use	Percent	2021	0.1538950 4	0.2264945 3	0.0827347 6
Deaths	Liaoning	Both	All ages	Larynx cancer	Occupational risks	Percent	2021	0.0524032 1	0.0838089 1	0.0298620 7
Deaths	Liaoning	Both	All ages	Larynx cancer	Occupational carcinogens	Percent	2021	0.0524032 1	0.0838089 1	0.0298620 7
Deaths	Liaoning	Both	All ages	Larynx cancer	Occupational exposure to asbestos	Percent	2021	0.0187543 2	0.0312608 2	0.0096186 7
Deaths	Henan	Both	All ages	Larynx cancer	Smoking	Percent	2021	0.7069	0.8122	0.5738

								3863	9831	0863
Deaths	Henan	Both	All ages	Larynx cancer	High alcohol use	Percent	2021	0.1244178 2	0.1882954 3	0.0614997 5
Deaths	Henan	Both	All ages	Larynx cancer	Occupational risks	Percent	2021	0.0535866 5	0.0897132	0.0278244 9
Deaths	Henan	Both	All ages	Larynx cancer	Occupational carcinogens	Percent	2021	0.0535866 5	0.0897132	0.0278244 9
Deaths	Henan	Both	All ages	Larynx cancer	Occupational exposure to asbestos	Percent	2021	0.0093971 4	0.0157044 8	0.0051698 9
Deaths	Inner Mongolia	Both	All ages	Larynx cancer	Smoking	Percent	2021	0.8033081 4	0.887627	0.6894813 9
Deaths	Inner Mongolia	Both	All ages	Larynx cancer	High alcohol use	Percent	2021	0.1396860 4	0.2089163 2	0.0719872 5
Deaths	Inner Mongolia	Both	All ages	Larynx cancer	Occupational risks	Percent	2021	0.0544468 6	0.0882474 3	0.0311377 9
Deaths	Inner Mongolia	Both	All ages	Larynx cancer	Occupational carcinogens	Percent	2021	0.0544468 6	0.0882474 3	0.0311377 9

Deaths	Inner Mongolia	Both	All ages	Larynx cancer	Occupational exposure to asbestos	Percent	2021	0.0163804 3	0.0256108 6	0.0089 99
Deaths	Hong Kong Special Administrative Region of China	Both	All ages	Larynx cancer	Occupational exposure to sulfuric acid	Percent	2021	0.0315489 1	0.0583514 5	0.0131208 7
Deaths	Hong Kong Special Administrative Region of China	Both	All ages	Larynx cancer	All risk factors	Percent	2021	0.6880596 6	0.8141128 9	0.5446301 5
Deaths	Shanxi	Both	All ages	Larynx cancer	Smoking	Percent	2021	0.7719457 5	0.8636343 7	0.6580372 6
Deaths	Shanxi	Both	All ages	Larynx cancer	High alcohol use	Percent	2021	0.1239801 2	0.1872588 6	0.0607182 1
Deaths	Shanxi	Both	All ages	Larynx cancer	Occupational risks	Percent	2021	0.0519816	0.0857706 2	0.0285044 7
Deaths	Shanxi	Both	All ages	Larynx cancer	Occupational carcinogens	Percent	2021	0.0519816	0.0857706 2	0.0285044 7
Deaths	Shanxi	Both	All ages	Larynx cancer	Occupational exposure to asbestos	Percent	2021	0.0123635	0.0194838 8	0.0067529 6
Deaths	Shanghai	Both	All ages	Larynx cancer	Smoking	Percent	2021	0.7785	0.8767	0.6578

								8562	5068	1808
Deaths	Shanghai	Both	All ages	Larynx cancer	High alcohol use	Percent	2021	0.1700051 3	0.2437403	0.0876761 5
Deaths	Shanghai	Both	All ages	Larynx cancer	Occupational risks	Percent	2021	0.0381063 4	0.0623384 7	0.0211725 8
Deaths	Shanghai	Both	All ages	Larynx cancer	Occupational carcinogens	Percent	2021	0.0381063 4	0.0623384 7	0.0211725 8
Deaths	Shanghai	Both	All ages	Larynx cancer	Occupational exposure to asbestos	Percent	2021	0.0114665 1	0.0205657 4	0.0056568 3
Deaths	Guizhou	Both	All ages	Larynx cancer	Tobacco	Percent	2021	0.7584692 1	0.8698027 7	0.6263124 7
Deaths	Gansu	Both	All ages	Larynx cancer	Environmental/occupational risks	Percent	2021	0.0567674 5	0.0930626 9	0.0315031 1
Deaths	Gansu	Both	All ages	Larynx cancer	Behavioral risks	Percent	2021	0.7624927 9	0.8437844 6	0.6452661 2
Deaths	Qinghai	Both	All ages	Larynx cancer	Occupational exposure to sulfuric acid	Percent	2021	0.0427151 4	0.0795218 3	0.0180793 8

Deaths	Qinghai	Both	All ages	Larynx cancer	All risk factors	Percent	2021	0.7187777 6	0.8200531 1	0.6042711
Deaths	Liaoning	Both	All ages	Larynx cancer	Environmental/occupational risks	Percent	2021	0.0524032 1	0.0838089 1	0.0298620 7
Deaths	Liaoning	Both	All ages	Larynx cancer	Behavioral risks	Percent	2021	0.8071275 1	0.8896444 2	0.6996959 4
Deaths	Anhui	Both	All ages	Larynx cancer	Occupational exposure to sulfuric acid	Percent	2021	0.0404905 8	0.0757744 7	0.0170147 7
Deaths	Anhui	Both	All ages	Larynx cancer	All risk factors	Percent	2021	0.8139595 9	0.8934758 4	0.7150336 4
Deaths	Beijing	Both	All ages	Larynx cancer	Occupational exposure to sulfuric acid	Percent	2021	0.0286653 6	0.0529362 7	0.0115576 9
Deaths	Beijing	Both	All ages	Larynx cancer	All risk factors	Percent	2021	0.8184126 6	0.8993037 4	0.7042807 4
Deaths	Hong Kong Special Administrative Region of China	Both	All ages	Larynx cancer	Smoking	Percent	2021	0.6158762 5	0.7682324 1	0.4442708 6
Deaths	Hong Kong Special Administrat	Both	All ages	Larynx cancer	High alcohol use	Percent	2021	0.1809	0.2741	0.0834

	ive Region of China							0732	0575	6132
Deaths	Hong Kong Special Administrative Region of China	Both	All ages	Larynx cancer	Occupational risks	Percent	2021	0.03973 6	0.06722254	0.0206852
Deaths	Hong Kong Special Administrative Region of China	Both	All ages	Larynx cancer	Occupational carcinogens	Percent	2021	0.039736	0.06722254	0.0206852
Deaths	Hong Kong Special Administrative Region of China	Both	All ages	Larynx cancer	Occupational exposure to asbestos	Percent	2021	0.00833851	0.01452861	0.00398821
Deaths	Beijing	Both	All ages	Larynx cancer	Smoking	Percent	2021	0.78502389	0.87940453	0.65961407
Deaths	Beijing	Both	All ages	Larynx cancer	High alcohol use	Percent	2021	0.17477806	0.25281779	0.09317253
Deaths	Beijing	Both	All ages	Larynx cancer	Occupational risks	Percent	2021	0.04604097	0.07263756	0.02620835
Deaths	Beijing	Both	All ages	Larynx cancer	Occupational carcinogens	Percent	2021	0.04604097	0.07263756	0.02620835
Deaths	Beijing	Both	All ages	Larynx cancer	Occupational exposure to asbestos	Percent	2021	0.01762436	0.02822251	0.00889057

Deaths	Hubei	Both	All ages	Larynx cancer	Smoking	Percent	2021	0.75646495	0.8437959595	0.64027044
Deaths	Hubei	Both	All ages	Larynx cancer	High alcohol use	Percent	2021	0.15768848	0.23224074	0.08190261
Deaths	Hubei	Both	All ages	Larynx cancer	Occupational risks	Percent	2021	0.04431781	0.07601718	0.02237447
Deaths	Hubei	Both	All ages	Larynx cancer	Occupational carcinogens	Percent	2021	0.04431781	0.07601718	0.02237447
Deaths	Hubei	Both	All ages	Larynx cancer	Occupational exposure to asbestos	Percent	2021	0.00627368	0.01067133	0.00311779
Deaths	Guangdong	Both	All ages	Larynx cancer	Environmental/occupational risks	Percent	2021	0.04924214	0.08314176	0.02526307
Deaths	Guangdong	Both	All ages	Larynx cancer	Behavioral risks	Percent	2021	0.81670567	0.89018475	0.72271572
Deaths	Sichuan	Both	All ages	Larynx cancer	Environmental/occupational risks	Percent	2021	0.05094258	0.08704038	0.02620849
Deaths	Sichuan	Both	All ages	Larynx cancer	Behavioral risks	Percent	2021	0.8005	0.8787	0.7042

								8173	1427	9368
Deaths	Anhui	Both	All ages	Larynx cancer	Smoking	Percent	2021	0.7851476 7	0.8730311	0.67546124
Deaths	Anhui	Both	All ages	Larynx cancer	High alcohol use	Percent	2021	0.13254087	0.20093903	0.06466423
Deaths	Anhui	Both	All ages	Larynx cancer	Occupational risks	Percent	2021	0.04877126	0.0844279	0.02535178
Deaths	Anhui	Both	All ages	Larynx cancer	Occupational carcinogens	Percent	2021	0.04877126	0.0844279	0.02535178
Deaths	Anhui	Both	All ages	Larynx cancer	Occupational exposure to asbestos	Percent	2021	0.00847352	0.01373278	0.004188
Deaths	Ningxia	Both	All ages	Larynx cancer	Smoking	Percent	2021	0.69692323	0.80230191	0.56531597
Deaths	Ningxia	Both	All ages	Larynx cancer	High alcohol use	Percent	2021	0.11327113	0.18082058	0.04887497
Deaths	Ningxia	Both	All ages	Larynx cancer	Occupational risks	Percent	2021	0.04751412	0.08196959	0.02210897

Deaths	Ningxia	Both	All ages	Larynx cancer	Occupational carcinogens	Percent	2021	0.0475141 2	0.0819695 9	0.0221089 7
Deaths	Ningxia	Both	All ages	Larynx cancer	Occupational exposure to asbestos	Percent	2021	0.0047127 7	0.0077560 1	0.0023903 8
Deaths	Hubei	Both	All ages	Larynx cancer	Environmental/occupational risks	Percent	2021	0.0443178 1	0.0760171 8	0.0223744 7
Deaths	Hubei	Both	All ages	Larynx cancer	Behavioral risks	Percent	2021	0.7793385 2	0.8578028 1	0.6807089 2
Deaths	Tianjin	Both	All ages	Larynx cancer	Smoking	Percent	2021	0.7813309 9	0.8695850 2	0.6634673 3
Deaths	Tianjin	Both	All ages	Larynx cancer	High alcohol use	Percent	2021	0.1556141 6	0.2337161 2	0.0869456 7
Deaths	Tianjin	Both	All ages	Larynx cancer	Occupational risks	Percent	2021	0.0360384 9	0.0606075 6	0.0196245
Deaths	Tianjin	Both	All ages	Larynx cancer	Occupational carcinogens	Percent	2021	0.0360384 9	0.0606075 6	0.0196245
Deaths	Tianjin	Both	All ages	Larynx cancer	Occupational exposure to asbestos	Percent	2021	0.0085	0.0136	0.0045

								7199	6911	0407
Deaths	Qinghai	Both	All ages	Larynx cancer	Tobacco	Percent	2021	0.68466866	0.79373018	0.55897637
Deaths	Tianjin	Both	All ages	Larynx cancer	Occupational exposure to sulfuric acid	Percent	2021	0.02759092	0.05047777	0.0110212
Deaths	Tianjin	Both	All ages	Larynx cancer	All risk factors	Percent	2021	0.81064852	0.88885854	0.70719129
Deaths	Sichuan	Both	All ages	Larynx cancer	Smoking	Percent	2021	0.78017633	0.86767106	0.67243747
Deaths	Sichuan	Both	All ages	Larynx cancer	High alcohol use	Percent	2021	0.14325276	0.21342157	0.07127215
Deaths	Sichuan	Both	All ages	Larynx cancer	Occupational risks	Percent	2021	0.05094258	0.08704038	0.02620849
Deaths	Sichuan	Both	All ages	Larynx cancer	Occupational carcinogens	Percent	2021	0.05094258	0.08704038	0.02620849
Deaths	Sichuan	Both	All ages	Larynx cancer	Occupational exposure to asbestos	Percent	2021	0.0078866	0.01325294	0.00402325

Deaths	Inner Mongolia	Both	All ages	Larynx cancer	Occupational exposure to sulfuric acid	Percent	2021	0.03846423	0.07102907	0.01599714
Deaths	Inner Mongolia	Both	All ages	Larynx cancer	All risk factors	Percent	2021	0.83280285	0.90769941	0.73303523
Deaths	Hunan	Both	All ages	Larynx cancer	Smoking	Percent	2021	0.7857865	0.87718987	0.6690177
Deaths	Hunan	Both	All ages	Larynx cancer	High alcohol use	Percent	2021	0.13688437	0.20132336	0.07273682
Deaths	Hunan	Both	All ages	Larynx cancer	Occupational risks	Percent	2021	0.05734318	0.09119657	0.03293308
Deaths	Hunan	Both	All ages	Larynx cancer	Occupational carcinogens	Percent	2021	0.05734318	0.09119657	0.03293308
Deaths	Hunan	Both	All ages	Larynx cancer	Occupational exposure to asbestos	Percent	2021	0.01610206	0.02597913	0.00886895
Deaths	Guangxi	Both	All ages	Larynx cancer	Occupational exposure to sulfuric acid	Percent	2021	0.04521099	0.08278102	0.01869122
Deaths	Guangxi	Both	All ages	Larynx cancer	All risk factors	Percent	2021	0.7720	0.8627	0.6662

								3622	2127	1012
Deaths	Guangxi	Both	All ages	Larynx cancer	Smoking	Percent	2021	0.73097126	0.83594488	0.61332815
Deaths	Guangxi	Both	All ages	Larynx cancer	High alcohol use	Percent	2021	0.15206972	0.22905588	0.07452171
Deaths	Guangxi	Both	All ages	Larynx cancer	Occupational risks	Percent	2021	0.05889467	0.09607641	0.03222492
Deaths	Guangxi	Both	All ages	Larynx cancer	Occupational carcinogens	Percent	2021	0.05889467	0.09607641	0.03222492
Deaths	Guangxi	Both	All ages	Larynx cancer	Occupational exposure to asbestos	Percent	2021	0.01409689	0.02339584	0.00721259
Deaths	Guangdong	Both	All ages	Larynx cancer	Smoking	Percent	2021	0.79384537	0.87392673	0.68741746
Deaths	Guangdong	Both	All ages	Larynx cancer	High alcohol use	Percent	2021	0.15674603	0.2223984	0.08245917
Deaths	Guangdong	Both	All ages	Larynx cancer	Occupational risks	Percent	2021	0.04924214	0.08314176	0.02526307

Deaths	Guangdong	Both	All ages	Larynx cancer	Occupational carcinogens	Percent	2021	0.04924214	0.08314176	0.02526307
Deaths	Guangdong	Both	All ages	Larynx cancer	Occupational exposure to asbestos	Percent	2021	0.00889697	0.01487388	0.00457145
Deaths	Hunan	Both	All ages	Larynx cancer	Occupational exposure to sulfuric acid	Percent	2021	0.04166133	0.07559769	0.01680487
Deaths	Hunan	Both	All ages	Larynx cancer	All risk factors	Percent	2021	0.8144829	0.89440868	0.71689745
Deaths	China	Both	All ages	Larynx cancer	High alcohol use	Percent	2021	0.14523164	0.20986655	0.07702686
Deaths	China	Both	All ages	Larynx cancer	Occupational risks	Percent	2021	0.05175848	0.08552197	0.02816669
Deaths	China	Both	All ages	Larynx cancer	Occupational carcinogens	Percent	2021	0.05175848	0.08552197	0.02816669
Deaths	China	Both	All ages	Larynx cancer	Occupational exposure to asbestos	Percent	2021	0.01184061	0.01864789	0.0066204
Deaths	China	Both	All ages	Larynx cancer	Occupational exposure to sulfuric acid	Percent	2021	0.0402	0.0738	0.0169

								1019	571	0033
Deaths	Hong Kong Special Administrative Region of China	Both	All ages	Larynx cancer	Environmental/occupational risks	Percent	2021	0.03979736	0.0672225 4	0.0206852
Deaths	Hong Kong Special Administrative Region of China	Both	All ages	Larynx cancer	Behavioral risks	Percent	2021	0.67648359	0.80450075	0.52609025
Deaths	Ningxia	Both	All ages	Larynx cancer	Occupational exposure to sulfuric acid	Percent	2021	0.04292378	0.07764443	0.01770769
Deaths	Ningxia	Both	All ages	Larynx cancer	All risk factors	Percent	2021	0.73058674	0.82241858	0.61274057
Deaths	Qinghai	Both	All ages	Larynx cancer	Environmental/occupational risks	Percent	2021	0.05589784	0.09279642	0.03070336
Deaths	Qinghai	Both	All ages	Larynx cancer	Behavioral risks	Percent	2021	0.70148288	0.8067474	0.57958722
Deaths	Beijing	Both	All ages	Larynx cancer	Environmental/occupational risks	Percent	2021	0.04604097	0.07263756	0.02620835
Deaths	Beijing	Both	All ages	Larynx cancer	Behavioral risks	Percent	2021	0.81027152	0.8946738	0.69310825

Deaths	Guizhou	Both	All ages	Larynx cancer	Occupational exposure to sulfuric acid	Percent	2021	0.0451414148	0.0811764 7	0.0189751
Deaths	Guizhou	Both	All ages	Larynx cancer	All risk factors	Percent	2021	0.7914491	0.8891617	0.66812293
Deaths	Anhui	Both	All ages	Larynx cancer	Environmental/occupational risks	Percent	2021	0.0487712 6	0.0844279	0.02535178
Deaths	Anhui	Both	All ages	Larynx cancer	Behavioral risks	Percent	2021	0.80378874	0.886643	0.70313395
Deaths	Guizhou	Both	All ages	Larynx cancer	Smoking	Percent	2021	0.75846921	0.86980277	0.62631247
Deaths	Guizhou	Both	All ages	Larynx cancer	High alcohol use	Percent	2021	0.13161144	0.21117756	0.06030797
Deaths	Guizhou	Both	All ages	Larynx cancer	Occupational risks	Percent	2021	0.06123721	0.09869017	0.03395836
Deaths	Guizhou	Both	All ages	Larynx cancer	Occupational carcinogens	Percent	2021	0.06123721	0.09869017	0.03395836
Deaths	Guizhou	Both	All ages	Larynx cancer	Occupational exposure to asbestos	Percent	2021	0.0165	0.0259	0.0088

								7567	5994	4305
Deaths	Tianjin	Both	All ages	Larynx cancer	Environmental/occupational risks	Percent	2021	0.0360603849	0.0606075 6	0.019624 5
Deaths	Tianjin	Both	All ages	Larynx cancer	Behavioral risks	Percent	2021	0.8040634 7	0.8848874 6	0.6966694 3
Deaths	Guangxi	Both	All ages	Larynx cancer	Environmental/occupational risks	Percent	2021	0.0588946 7	0.0960764 1	0.0322249 2
Deaths	Guangxi	Both	All ages	Larynx cancer	Behavioral risks	Percent	2021	0.7578196	0.8538298 1	0.6480105 9
Deaths	Inner Mongolia	Both	All ages	Larynx cancer	Environmental/occupational risks	Percent	2021	0.0544468 6	0.0882474 3	0.0311377 9
Deaths	Inner Mongolia	Both	All ages	Larynx cancer	Behavioral risks	Percent	2021	0.8231898 2	0.9027857 3	0.7177142 6
Deaths	Qinghai	Both	All ages	Larynx cancer	Smoking	Percent	2021	0.6846686 6	0.7937301 8	0.5589763 7
Deaths	Qinghai	Both	All ages	Larynx cancer	High alcohol use	Percent	2021	0.1105328 9	0.1721620 2	0.0523596 3

Deaths	Qinghai	Both	All ages	Larynx cancer	Occupational risks	Percent	2021	0.0558979784	0.0927964 2	0.03070336
Deaths	Qinghai	Both	All ages	Larynx cancer	Occupational carcinogens	Percent	2021	0.0558979784	0.0927964 2	0.03070336
Deaths	Qinghai	Both	All ages	Larynx cancer	Occupational exposure to asbestos	Percent	2021	0.0136006 1	0.0211429 1	0.00762903
Deaths	Hunan	Both	All ages	Larynx cancer	Environmental/occupational risks	Percent	2021	0.0573431 8	0.0911965 7	0.03293308
Deaths	Hunan	Both	All ages	Larynx cancer	Behavioral risks	Percent	2021	0.8030805 6	0.8886965 1	0.7022294 5
Deaths	Ningxia	Both	All ages	Larynx cancer	Environmental/occupational risks	Percent	2021	0.0475141 2	0.0819695 9	0.0221089 7
Deaths	Ningxia	Both	All ages	Larynx cancer	Behavioral risks	Percent	2021	0.716551	0.8152128 8	0.5934523
Deaths	China	Both	All ages	Larynx cancer	Environmental/occupational risks	Percent	2021	0.0517584 8	0.0855219 7	0.0281666 9
Deaths	China	Both	All ages	Larynx cancer	Behavioral risks	Percent	2021	0.7889	0.8640	0.6897

								0653	8295	3925
Deaths	Guizhou	Both	All ages	Larynx cancer	Environmental/occupational risks	Percent	2021	0.0612 3721	0.0986 9017	0.0339 5836
Deaths	Guizhou	Both	All ages	Larynx cancer	Behavioral risks	Percent	2021	0.7767 2801	0.8831 9102	0.6510 3944
